# A systematic review on the impact of social support on college students’ wellbeing and mental health

**DOI:** 10.1371/journal.pone.0325212

**Published:** 2025-07-11

**Authors:** Li Ruihua, Norlizah Che Hassan, Zhu Qiuxia, Ouyang Sha, Dong Jingyi

**Affiliations:** 1 Faculty of Basic Education, Putian University, Putian, Fujian, China; 2 Faculty of Educational Studies, Universiti Putra Malaysia, Serdang, Selangor, Malaysia; 3 Faculty of Art, Lanzhou University of Finance and Economics, Lan zhou, Gansu, China; 4 Faculty of Marxism, Shaanxi Railway Institute, Weinan, Shaanxi, China; The Open University of Israel, ISRAEL

## Abstract

In recent years, growing attention has been directed toward undergraduate students’ mental health and overall well-being. The transition to university life, coupled with academic and social demands, has been shown to strain students’ psychological functioning considerably. Social support is often cited as a protective factor that can help mitigate these pressures; however, in-depth investigations focusing specifically on this relationship within the college student demographic remain relatively limited. This review examines how social support influences university students’ mental health and well-being outcomes. A systematic analysis was carried out, encompassing 51 empirical studies published between 2010 and 2024. These studies were identified through a comprehensive search of six major academic databases: Web of Science (WOS), Scopus, ProQuest, APA PsycINFO, PubMed, and Cochrane. The review highlights the nuanced relationship between social support and student well-being, underscoring the significance of social resources in shaping psychological outcomes during the higher education experience. The review identified both direct and indirect effects of social support. Direct effects include improved psychological and emotional well-being, reduced stress, and better health behaviours. Indirect effects highlight social support’s role as a mediator, enhancing resilience, self-esteem, and life satisfaction by providing emotional and informational resources. These results emphasize the importance of nurturing supportive relationships to promote student well-being.

## 1. Introduction

### 1.1. Research background

Entering university is a significant milestone in a young person’s life, with considerable academic, social, and emotional changes. This stage is exciting and challenging for many students as they adjust to newfound independence, increased workloads, and pressure to develop personal and professional identities [1,2]. University students’ overall well-being and mental health can be significantly impacted by various factors. Maintaining good mental health is crucial for their academic success, positive social relationships, and smooth transition into adulthood [3,4]. However, research suggests that college students are at higher risk for mental health problems such as depression, anxiety, and stress compared to their non-student peers [5]. Many researchers have emphasized the importance of social support, showing how it significantly benefits college students by improving their overall mental health and well-being [6–8]. Students who feel they have strong social support generally experience better mental health, face fewer psychological difficulties, and tend to perform better academically [8]. Existing studies indicate that having good social support helps university students feel like they belong, manage challenging situations more efficiently, and become stronger emotionally, ultimately boosting their mental health and overall well-being.

### 1.2. Related literature and research perspective

#### 1.2.1. Well-being.

The idea of “well-being” has been around in health conversations since the World Health Organization introduced its definition in 1948. However, it started gaining more attention after 2000, especially with the rise of positive psychology. This field aims to understand and enhance the factors that help individuals and communities thrive [9]. Although there is no universally accepted definition for well-being, it has often been described simply as the experience of feeling happy and being able to manage daily life effectively [10]. Well-being is often described as multidimensional, with various theoretical models proposed to capture its different aspects. For example, the PERMA model identifies five key elements of well-being: positive emotions, engagement, relationships, meaning, and accomplishment [9]. Huppert and So [11] have also proposed ten flourishing features, including emotional stability, vitality, optimism, resilience, and competence. From the perspective of positive psychology, high levels of well-being are associated with various positive outcomes, such as life satisfaction, positive emotions, higher productivity, increased life meaning, and lower levels of psychological distress [12]. Alongside the concept of well-being, the term “flourishing” is also frequently used in literature. Flourishing is “the experience of life going well” characterized by feeling good and functioning effectively [12]. Researchers have conceptualized well-being from two main perspectives: hedonic and eudaimonia. The hedonic view focuses on positive and negative emotions (subjective well-being), while the eudaimonia view emphasizes psychological functioning (psychological well-being) [13]. Subjective well-being (SWB), often equated with happiness, is typically defined as encompassing positive emotions, the absence of negative emotions, and overall life satisfaction [14].

Many researchers have investigated what student well-being means in higher education, but there is still no complete agreement. Some have suggested it includes how students feel emotionally, how satisfied they are with their learning environment, and the quality of their interactions with classmates and teachers [15,16]. Other researchers break down student well-being into areas like emotional health, feeling connected to the school, and having good relationships with teachers and friends [17]. Researchers have also looked at college students’ sense of well-being by examining things like their goals for the future, how involved they feel in their studies, their attendance habits, how well they perform academically, and whether they stay enrolled [18–20]. In this review, We will examine psychological well-being and how people genuinely feel about themselves and their lives. This includes being happy in the moment and having a more profound sense of meaning, purpose, and fulfilment.

#### 1.2.2. Social support.

Social support encompasses the emotional, practical, and motivational aid people obtain from their interpersonal connections, highlighting the nature and diversity of these relationships [21]. This concept has been widely explored concerning mental health and overall well-being. Researchers often distinguish various types of social support, including tangible help with everyday activities, informational support such as advice or guidance, emotional support characterized by empathy and care, and the simple presence of companionship or friendship [22–24]. Individuals may receive support from different sources, such as family, friends, or romantic partners, each offering unique contributions to their overall support network [21]. Friendships are crucial in enhancing an individual’s emotional well-being and providing tangible assistance, significantly impacting their happiness and quality of life. According to the existing research, friends play a significant role in providing emotional comfort and practical help, influencing how happy someone feels. When we talk about “perceived social support,” we describe how a person feels about the support they get from people around them—like their family, friends, or romantic partners, especially when things get tough. This support can mean emotional encouragement, sound advice, practical help with everyday tasks, or simply getting honest and helpful feedback [25].

#### 1.2.3. The role of social support in psychological well-being.

Good social support is essential for our emotional health, and it is well-known to help people deal better with stress and stay healthier overall. Researchers have proposed different ideas about why this happens. For example, the Main Effect Model theory explains that social support helps people feel connected, boosts their confidence, and gives them the necessary resources [26]. Similarly, Ryff and Keyes pointed out that having good relationships with others is crucial to feeling genuinely happy and fulfilled, emphasizing how meaningful social connections are for our well-being [27]. Studies consistently find that social support impacts our emotional well-being more than physical health. It is strongly linked to feeling happier and more satisfied with life, largely because how we see our lives often depends on the quality of our relationships and the support, we feel from those around us [28,29]. When college students feel supported by their teachers and friends, they adapt to university life, feel more motivated in their studies, and achieve better academic results. This support can also improve how they feel socially and emotionally during college [30,31]. Feeling that emotional support is available when needed can make a big difference in how happy and satisfied you feel [32].

Studies also point out how meaningful good relationships are for college students, especially emphasizing that when students feel supported by their families, they tend to be happier and more satisfied with their lives [33]. College students often find it easier to manage difficulties and feel more content with their lives when they believe they have someone who genuinely cares, offers comfort during stressful moments, and gives helpful guidance [34,35]. Researchers have also noticed that social support can help soften the impact of stress on mental health. Having supportive people around can change how someone sees a stressful situation, making it feel less overwhelming and leading to better emotional outcomes [36–38]. In summary, supportive relationships are essential because they help people deal more effectively with life’s ups and downs. This kind of support can boost students’ academic success and personal development and positively affects people’s emotional health and well-being across many groups.

### 1.3. Review aims and gaps

While research has established links between social support and undergraduate well-being, the field lacks a comprehensive synthesis of existing knowledge. This gap highlights the need for a systematic literature review to consolidate past findings, identify research trends, and guide future investigations in this critical area of student mental health [39,40]. This systematic review integrates recent empirical studies to examine the impact of social support on college students’ academic success, social integration, and mental health. It examines peer-reviewed, English-language studies published between 2010 and 2024, addressing the question: How does contemporary research describe the connection between social support and well-being or mental health outcomes among college students?

Four specific sub-questions support this primary question:

Research question 1: What major research directions have emerged from 2010 to 2024 regarding the connection between social support and college students’ well-being and mental health?

Research question 2: How do researchers measure well-being and social support in studies?

Research question 3: What are the direct effects of social support on the well-being of college students?

Research question 4: What are the indirect effects of social support on well-being, including mediating and moderating factors?

## 2. Methodology

The systematic literature review (SLR) approach was first introduced within the medical field, valued for its methodological precision and clarity. This review follows the PRISMA-P 2015 checklist (S1 File) [41]. Due to the diverse types of studies and sample heterogeneity, results will be synthesized thematically. A predefined protocol covering search methods, criteria for inclusion and exclusion, screening, data management, and article coding was registered with PROSPERO (ID: CRD42024535966).

### 2.1. Search strategy

A scoping search was performed in the spring of 2024 to identify appropriate search keywords using the Education Source via EBSCO and SCOPUS databases. The initial literature search used criteria including the topic area (social support and well-being), empirical study designs, and college student populations. These parameters later served as formal inclusion criteria for the selected studies. To ensure the identification of relevant and impactful keywords, consultations were conducted with a subject librarian and two psychology experts. The literature search was carried out across six major databases—Web of Science (WOS), Scopus, APA PsycINFO, ProQuest, PubMed, and the Cochrane Library—utilizing a systematic approach to maximize the breadth and depth of the search results.

A backward snowballing technique was employed to ensure comprehensive coverage of the literature, following the methodology outlined by Jalali and Wohlin [42]. This approach systematically examined key reference lists to identify additional relevant studies not captured through the initial keyword-based search. The review focused on studies published between 2010 and 2024, emphasizing recent and pertinent research addressing contemporary factors influencing college student’s mental health, such as the impact of social media and significant global events like the COVID-19 pandemic. This period captures recent trends, ensuring the analysis aligns with the current challenges and supports dynamics impacting student well-being. With the help of the subject librarian, search strings were tailored to meet the specific requirements of each database, as outlined in Table 1. For the total number of hits across all databases for the search strategy, see S2 File.

**Table 1:** Keywords Used in Database Searches.

Search Criteria	Search terms embedded in titles, abstracts, and keywords
1	“Social support” OR “social relation*” OR “social network*”OR” family support “OR “family relation*”OR “emotional support “OR” financial support”OR”instrumental support”OR”tangible support “OR” informational support “OR” appraisal support”
2	“wellbeing” OR “wellbeing” OR “psychological wellbeing” OR “psychological wellbeing” OR “happiness” OR “flourish” OR “flourishing” OR “psychological flourish” OR “psychological flourishing” OR “subjective wellbeing” OR “subjective wellbeing” OR “positive emotions” OR “positive emotion” OR “positive affect” OR “engagement” OR “flow” OR “psychological flow” OR “positive relationship” OR “positive relationships” OR “social support” OR “meaning” OR “meaning of life” OR “meaning in life” OR “life meaning” OR “life purpose” OR “purpose of life” OR “purpose in life” OR “achievement” OR “achievements” OR “accomplishment” OR “accomplishments” OR “performance” OR “success.”
3	“College students” OR”undergraduates”
Final Search Query	Combination of Stages 1, 2, and 3
Databases Searched	Web of Science (WOS), Scopus, APA PsycINFO, ProQuest, PubMed, and Cochrane Library
Date Range	From January 1, 2010, to March 28, 2024.

### 2.2. Inclusion\exclusion criteria for studies

This systematic review used PICO (Population, Intervention, Comparison, Outcome) and SPIDER (Sample, Phenomenon of Interest, Design, Evaluation, Research type) frameworks to guide the literature search. These tools are recognized for their effectiveness in conducting comprehensive research, particularly for systematic reviews [43,44]. The study established two sets of eligibility criteria: inclusion and exclusion. These criteria, detailed in Table 2, were applied to filter and select relevant papers for the investigation.

**Table 2:** Inclusion and Exclusion Parameters for Study Selection.

Criterion	Included	Excluded
Target Population	Research involving college or undergraduate students.	Studies focusing on primary/secondary school students or adult populations.
Intervention	No specific intervention is required.	Not applicable.
Comparison Groups	Not necessary for inclusion.	Not applicable.
Geographic Scope	Studies conducted in any national context.	No geographic limitations were applied.
Measured Outcomes	Articles exploring links between social support and mental health or well-being.	Studies where outcomes pertain to mental health disorders without connection to well-being or support.
Context/Setting	Research that broadly addresses social support, mental health, and well-being.	Works unrelated to these key areas.
Language	Publications written in English or translated into English.	Non-English language studies without translation.
Study Design	Empirical research using quantitative, qualitative, or mixed methods.	Publications lacking empirical data.

### 2.3. Data extraction

**Fig 1:**
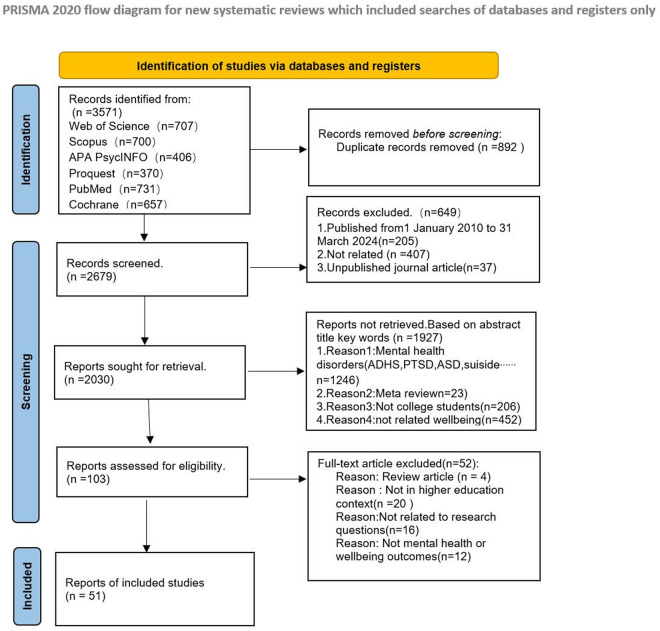
Flow chart of literature search. PRISMA flow diagram showing records identified, screened, excluded, and included in the review.

The process began with an extensive database search, resulting in 3,571 articles: 707 from Web of Science (WOS), 700 from Scopus, 406 from APA PsycINFO, 370 from ProQuest, 731 from PubMed, and 657 from Cochrane. After removing 892 duplicates, 2,679 articles remained for further assessment. These were then screened based on their titles, abstracts, and keywords to determine their relevance, excluding many unrelated studies. The diagram indicates that 1,927 reports were excluded from detailed reviews for various reasons: focus on specific mental health conditions (e.g., ADHD, PTSD, ASD, suicide; 1,246 records), meta-review articles (23 records), non-college student samples (206 records), and topics not directly related to well-being (452 records). Of the retrieved records, only 103 were assessed for eligibility through a full-text review. This review was critical in determining the studies’ pertinence to the research question. In total, 51 full-text articles were removed from consideration due to specific criteria: four were literature reviews, 20 did not involve higher education settings, 16 lacked relevance to the study objectives, and 12 did not address outcomes related to mental health or well-being. Two independent reviewers screened the full text of all records for eligibility, categorizing them as include, unsure, or exclude. Disagreements were resolved through discussion, with a third reviewer consulting for unresolved cases. A numbered table of all 2679 studies identified in the literature search, with the full title of each article, including those that were excluded from the analyses with the reasons for their exclusion, see S3 File. The screening process’s reliability was assessed using per cent agreement and Cohen’s Kappa, which accounts for chance agreement. The average interrater agreement for the initial screening was high at 92%. After this rigorous process, 51 articles were selected for further analysis, as illustrated in Fig 1.

### 2.4. Evaluation of study quality

The Crowe Critical Appraisal Tool (CCAT) was employed to evaluate the research quality included. This tool was chosen for its versatility in assessing various research designs, including quantitative, qualitative, and mixed-method studies, which is crucial given the heterogeneous nature of the studies in this review. The CCAT is also noted for its high reliability. Each category is scored out of 5, with a maximum total score of 40 (S4 File) [45]. Two reviewers independently performed the quality evaluation, and any discrepancies were settled through discussion until consensus was achieved. CCAT scores and results are detailed in the S5 File. S6 File was developed presenting the completed risk of bias (using the JBI checklist) and GRADE quality/certainty assessments for each study and outcome, ensuring a transparent evaluation of methodological quality and evidence strength across the included studies.

### 2.5. Synthesis and analysis of results

A meta-analysis was initially considered but ultimately determined inappropriate due to substantial methodological heterogeneity among the included studies. Specifically, the studies varied considerably regarding their research designs (e.g., cross-sectional surveys, longitudinal studies), populations, social support and well-being measurement instruments, and outcome reporting methods (e.g., correlations, group comparisons, mediation analyses). Because a meta-analysis requires comparable quantitative metrics, attempting to statistically combine these diverse results into a single summary effect would have been misleading and invalid [46]. Given the exploratory nature of our research objectives, thematic analysis was strategically employed as a methodological framework for data synthesis. This approach facilitated a rigorous process of iterative coding and thematic organization, allowing systematic extraction and categorization of critical insights from the reviewed studies. Through this narrative synthesis methodology, we achieved three key analytical outcomes: (1) identification of emergent trends and conceptual patterns within the literature, (2) comprehensive documentation of measurement methodologies employed across empirical investigations, and (3) nuanced evaluation of both direct causal relationships and indirect mediating pathways linking social support mechanisms to psychological well-being outcomes in collegiate populations. The thematic synthesis framework proved particularly effective in accommodating the complexity of our multi-faceted research aims while maintaining analytical coherence. This methodological choice aligns with established best practices for evidence synthesis when statistical aggregation is not feasible [47].

We carefully reviewed each article during the coding phase to identify key points about social support, mental health, and well-being. Two researchers went through each paper, sorting the information into clear categories. For every study, we recorded the authors, year of publication, country, title, purpose, methods, types of variables involved (including independent, mediator, moderator, and dependent), participant information, and main findings. We used descriptive coding to summarize the information within each category, which helped us recognize patterns and generate themes directly from the articles. To analyze the qualitative data, we mainly followed [48,49] thematic analysis approach, which involves several steps: getting familiar with the data, creating initial codes, finding and reviewing themes, refining and defining them, and clearly explaining each one. Both researchers coded the data separately and then came together to discuss and agree on the final themes, ensuring the analysis was thorough and accurate. The first and third authors independently coded and qualitatively analyzed all articles, achieving high inter-rater reliability (Cohen’s Kappa = .80). Through an iterative categorization process and discussion among the authors, initial codes were grouped into broader themes. The resulting themes and their analysis are presented in the results part: 1) the conceptualization of well-being and social support as they presented in the selected studies;2) the various determinants that influence wellbeing according to the reviewed research;3) dynamics between social support and wellbeing, probing into the nature of their interconnection, aims to elucidate the mechanism by which social support contributes to wellbeing.

## 3. Results

### 3.1. Summary of research contexts and study attributes

This paper presents both quantitative and qualitative findings. The authors independently categorized the selected journal articles based on thematic content. Analysis of the chosen publications included examination by year, location, journal, research objectives, methodology, and key findings. Detailed characteristics of the 51 included studies are provided in the S7 File.

**Fig 2:**
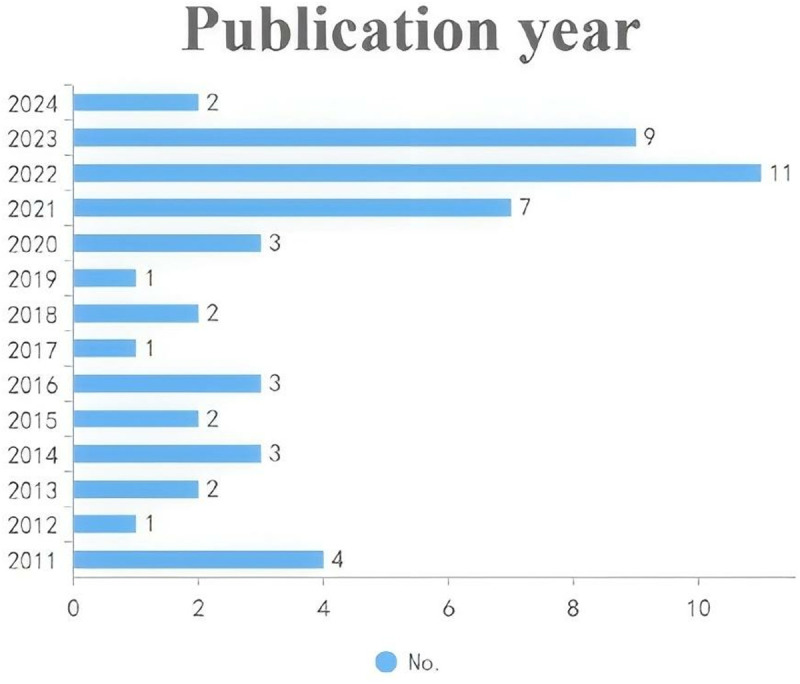
Publications by year. Annual distribution of included studies showing trends in publication volume over time.

As shown in Fig 2, the yearly distribution of the 51 articles reviewed between 2011 and 2024 reveals initial fluctuations, starting with four studies in 2011. The publication rate remained modest until a noticeable increase in 2017 (five articles), followed by a consistent upward trend from 2018 onward, highlighting increased scholarly attention. This trend peaked in 2023, with eleven articles illustrating a substantial rise in interest regarding the relationship between social support and college student’s mental health and well-being. Early data from 2024 currently shows two publications, reflecting continued academic engagement with the topic. Fig 2 demonstrates the growing research emphasis on social support within higher education settings.

**Fig 3:**
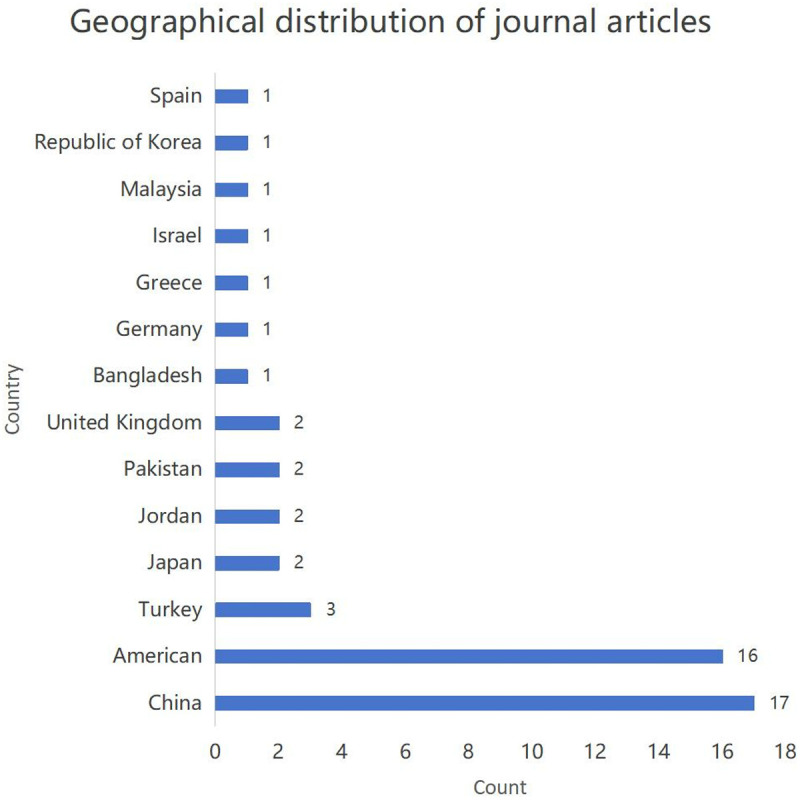
Geographical distribution of studies. Map showing countries where included studies were conducted based on first author affiliation.

Additionally, Fig 3 summarizes country-specific contributions, showing China and the United States as dominant contributors, with 17 and 16 publications, respectively, highlighting their leading roles in advancing this research area. Turkey, Japan, Jordan, United Kingdom, Germany, Israel, Malaysia, the Republic of Korea, Greece, Spain, Bangladesh, and Pakistan contribute to a lesser extent, with 1–3 publications each. The review revealed a strong preference for quantitative research designs among the analyzed studies. Fifty of the included articles employed quantitative methods, utilizing diverse data collection techniques such as pre-and post-workshop surveys, assessments, and tests. In contrast, only one article adopted a qualitative approach, gathering data through observational methods and interviews.

### 3.2. Themes

This research explores the connections among social support, well-being, and mental health in college students, focusing on measurement approaches, determinants of well-being, and the impact social support has on students’ psychological health and overall wellness.

#### 3.2.1. Instrument of well-being and social support.

In psychology, well-being is a positive state encompassing mental health and overall life satisfaction. A widely accepted definition within the field of positive psychology describes it as “the combination of feeling good and functioning well.” Practically, this entails experiencing positive emotions such as happiness and contentment while striving to fulfil one’s potential—cultivating a sense of purpose, effectively managing daily responsibilities, and fostering meaningful relationships [12]. It aligns with the World Health Organization’s view of mental health as a state of well-being in which individuals realize their abilities, can cope with everyday stresses, work productively, and contribute to their community [50].

To empirically investigate happiness and well-being, researchers use multiple validated measures that reflect the multidimensional nature of these concepts. Wellbeing is measured through 7 different methods, including subjective well-being, satisfaction with life, mental health inventory, spiritual well-being, quality of life, depression, and health behaviours. Table 3 also indicates that depression, anxiety, and stress were assessed as a combined construct. Moreover, social support is evaluated using two main methods: general and perceived support. For detailed information on well-being and social support measures, see S8 File.

**Table 3:** Wellbeing Measurement.

Variables	Measurement	Amount
wellbeing	Subjective wellbeing	26
	Satisfaction with life	14
	Mental health inventory	5
	Spiritual wellbeing	2
	Quality of life	2
	Health behaviours	2

Well-being is a complex and multidimensional construct commonly assessed through various validated instruments reflecting subjective, psychological, and social dimensions. Among the 51 studies reviewed, 26 employed measures of subjective well-being. For example, Kong and You utilized the Satisfaction with Life Scale (SWLS) and the Positive and Negative Affect Scale (PANAS) to assess participants’ emotional and cognitive evaluations of life [51]. In contrast, psychological well-being was explored in greater depth using instruments such as the Ryff Psychological Well-Being Scale and the Flourishing Scale (FS), as evidenced in the study by Brunsting, Zachry [52]. School-related well-being, focusing on students’ emotional experiences and satisfaction within the educational setting, was addressed by Sun, Jiang [53]. Personal well-being was assessed by Wang, Chua [54] using a scale developed by Diener et al., integrating both emotional states and overall life satisfaction. Broader assessments of happiness and mental health were conducted using the Oxford Happiness Questionnaire (OHQ) by Wang, Chua [55] and the Rand Mental Health Inventory (MHI-5) by Lee, Chung [56] and Johnson [57]. Notably, 17 studies in the review utilized the SWLS to evaluate life satisfaction. The SWLS, developed by Diener [58], remains one of the most widely used tools, with applications in studies such as Cahuas, Marenus [59], and Kong [60]. To assess well-being specifically among student populations, tools such as the Student Life Satisfaction Scale (SLSS) referenced in Roming and Howard [61] and the Brief Multidimensional Student Life Satisfaction Scale (BMSLSS) by Huebner et al. (2006), as cited in Holliman, Waldeck [62], were employed. These instruments provide a more nuanced evaluation of student satisfaction across home, school, and peer relationships. Collectively, these tools contribute to a robust framework for examining student well-being across diverse contexts.

Social support broadly refers to providing comfort, care, and resources that individuals receive from their social network, helping them manage biological, psychological, and social stressors [63]. It includes practical or tangible assistance, such as financial or physical help, and emotional reassurance that individuals are loved, valued, and integrated into a supportive network characterized by mutual obligations [64]. Social support thus involves both actual supportive behaviours and the perception or belief that such support is available when needed [65]. According to House’s influential framework, social support can manifest emotional support (empathy, trust), instrumental support (concrete help), informational support (advice), and appraisal support (feedback for self-evaluation).

Various instruments have been developed to measure social support, each designed to capture different aspects and sources of support. These instruments assess perceived support from family, friends, significant others, and other social networks. Among the many available instruments, the Multidimensional Scale of Perceived Social Support is the most frequently used tool, appearing in 18 studies.

Researchers have utilized various standardized instruments to comprehensively assess social support, each capturing distinct dimensions of the construct. Table 4 summarizes the various measurement tools used to assess social support across the reviewed studies. Among the most widely used measures is the Multidimensional Scale of Perceived Social Support (MSPSS), which evaluates perceived support from family, friends, and significant others through 12 items rated on a 7-point Likert scale [53,66–68]. Similarly, the Perceived Social Support Scale has been employed in six studies to measure perceived support across different sources using Likert-scale responses [69–71]. Additional tools include the Social Support Rating Scale (SSRS), utilized in two studies [51,67], and the Brief Perceived Social Support Questionnaire (BPSSQ), featured in three studies [72–74]. The Inventory of Socially Supportive Behaviors (ISSB) stands out for its focus on the frequency of received supportive actions rather than perceptions alone and has been applied in two studies [75]. These scales reflect the multifaceted nature of social support, addressing various dimensions such as emotional comfort, practical assistance, and relational dynamics. Collectively, they underscore the complexity of understanding social support’s impact on well-being across diverse populations and contexts.

**Table 4:** Social support Measurement.

Variables	Measurement	Amount
Social support	Multidimensional Scale of Perceived Social Support (MSPSS)	18
	Perceived Social Support Scale (PSSS)	6
	Brief Perceived Social Support Questionnaire (BPSSQ)	3
	Inventory of Socially Supportive Behaviors (ISSB)	2
	Social Support Rating Scale (SSRS)	2
	Social Provisions Scale (SPS)	1
	Social Support Questionnaire (SSQ)	1
	Other Scales	17

#### 3.2.2. Direct effects of social support on well-being.

Social support is widely acknowledged as a vital determinant of happiness and life satisfaction across various domains of life. It encompasses emotional encouragement, practical assistance, and valuable advice, all of which make individuals feel appreciated and better equipped to navigate life’s challenges. This review synthesizes findings from 51 research articles to explore the impact of social support on well-being, emphasizing the distinct roles played by various types of support in shaping psychological outcomes.

There is substantial evidence that social support markedly benefits emotional and psychological well-being. Individuals who perceive their support networks as reliable tend to report more positive emotions, fewer negative emotions, and greater life satisfaction [56,67,76]. Likewise, strong family support is associated with lower anxiety and sadness and higher levels of happiness and comfort [77–79]. Social support is vital in mitigating stress helping individuals maintain emotional stability during adversity. Research has consistently demonstrated that reliable social networks can buffer the adverse effects of stress by reducing emotional distress and physiological responses [80].

Social support helps individuals cope with adverse life events by providing emotional comfort, practical assistance, and informational resources, which can be crucial in navigating challenges and uncertainties [67,81]. Additionally, social support enhances various aspects of life quality, including emotional well-being, social relationships, and physical health [74,76,82]. Daily social support positively correlates with daily well-being among emerging adults, suggesting that consistent, everyday interactions with supportive individuals contribute significantly to overall happiness and life satisfaction [83]. Moreover, social support positively influences health behaviours like physical activity and nutrition. This support often comes as motivation to exercise regularly, maintain a balanced diet, and adhere to medical advice, collectively contributing to improved physical health [57,84]. Overall, the substantial body of research underscores the profound impact of social support on multiple dimensions of well-being. It highlights the importance of fostering supportive relationships to enhance psychological health, manage stress, improve quality of life, and promote healthier behaviours.

The distinct contributions of various types of social support—emotional, informational, instrumental, and appraisal support—to enhance well-being across different dimensions. Emotional support, including empathy, love, trust, and caring, significantly improves well-being by reducing negative and increasing positive emotions [53,85]. Perceived social support from family and friends reduces negative emotions and increases positive emotions like excitement and happiness [72,79]. Furthermore, informational support involves providing advice, suggestions, and information that help individuals cope with problems. This type of support helps students manage stress and maintain mental health by offering practical solutions and resources [81,83]. Instrumental support, encompassing tangible assistance and services, is crucial in enhancing well-being by addressing practical needs. This form of support enables individuals to manage daily responsibilities effectively and alleviates stress, thereby contributing to improved life satisfaction and mental health outcomes [54,82,85]. These findings emphasize the crucial role of fostering supportive relationships from family, friends, significant others, and faculty in enhancing life satisfaction, psychological well-being, and reducing stress.

#### 3.2.3. Indirect effects of social support on well-being.

The intricate relationship between social support and psychological well-being has been extensively examined, revealing its pivotal role in enhancing mental health, life satisfaction, and resilience across diverse contexts, including education and personal development. Social support is a mediator that fosters emotional stability, reduces stress, and enables individuals to navigate challenges more effectively, contributing to improved psychological outcomes. This collection of research elucidates how social support impacts well-being, emphasizing the importance of social connections and emotional intelligence in enhancing mental health across diverse demographics and various stressors, including those related to the COVID-19 pandemic. For example, Kim & Lee [86] showed that meaningful Facebook friendships can provide the same level of social support as traditional ones, enhancing well-being if the relationships are nurtured with time and effort. Kong and You [51] revealed the multifaceted role of social support in improving self-esteem and reducing loneliness, thereby leading to increased life satisfaction. Self-esteem and loneliness mediate the complex interplay between social support and life satisfaction, demonstrating how social networks influence emotional states. The impact of gratitude on social dynamics is profound. In another two studies, researchers explored how gratitude fosters interpersonal relationships and enhances perceived social support, subsequently improving school well-being and life satisfaction [51,53]. Deichert, Fekete [80] and Yang, Zhang [83] further expand on this by illustrating how gratitude combined with social support can reduce stress and build resilience, promoting overall psychological well-being. Adopting a similar position, Kase, Endo [87] illustrated the specificity of social support in different contexts, noting that in Japanese university students, support from friends and family significantly bolsters mental health by strengthening their Sense of Coherence (SOC). [56] further highlight how network density and offline bonding capital can indirectly affect well-being through perceived social support, showcasing how structured relationships contribute to supportive environments. Zeidner and Matthews [88]and Shuo, Xuyang [89] discuss how social support acts as a bridge between emotional intelligence and psychological health, with emotional intelligence enhancing the perception and utilization of social support. This, in turn, promotes greater well-being, indicating the intricate linkages between emotional competencies and social interactions.

Yildirim and Tanrýverdi [74] examined how social support facilitates resilience and self-esteem, influencing life satisfaction and happiness. Ma [90] observed that teenagers who feel well-supported by others tend to hold stronger positive beliefs about themselves and feel more satisfied with life, showing how the social environment can positively shape young people. This is particularly important in times of crisis, like the COVID-19 pandemic. For instance, Fan and Liu [91] found that emotional support from teachers and personal resilience helped reduce students’ anxiety caused by COVID-19, improving their overall mental health. However, Xin [92] cautioned that social support can sometimes become stressful if it is forced or feels overwhelming, suggesting that support needs to be thoughtfully balanced. Additionally, Liu [93] highlighted that social support can help individuals grow personally and forgive others more efficiently, ultimately boosting their well-being and improving relationships.

Together, these studies emphasize that strong social connections significantly influence people’s happiness, resilience, and sense of community. These findings show that social support bridges personal experiences, environmental influences, and emotional well-being. They underscore the importance of nurturing social networks, particularly in educational settings and during challenging times such as global health crises.

## 4. Discussion

### 4.1. Social support and student well-being in context

The present review highlights that social support broadly benefits college students’ well-being, aligning with extensive prior research. Across the studies, support from family, friends, and significant others was consistently linked to higher life satisfaction and positive mental health outcomes. This finding resonates with decades of evidence that individuals with close, supportive relationships experience greater life satisfaction and fewer psychological problems [94,95]. Social support is a multidimensional construct – it encompasses different types (emotional, instrumental, informational/companionship) and sources (e.g., family, friends, and romantic partners) [96]. Our synthesis encompassed this breadth, revealing that all forms of support helped students cope with challenges and added meaning to their lives. Multiple studies in the review reported that robust support networks correspond to lower stress and psychological distress among students. This aligns with the classic stress-buffering model, in which social support mitigates the impact of stress on well-being [26]. Recent evidence in student populations confirms that high perceived support can buffer against depression, anxiety, and even suicidal risk [97]. Such converging findings underscore that social support correlates with improved well-being and is a protective factor during adversity [98]. Essentially, our review reaffirms that having an adequate support system is fundamental to thriving in the college years, which is consistent with the broader psychological literature on social relationships and health.

### 4.2. Offline versus online support

A notable contribution to this review is its examination of offline (face-to-face) and online social support. We found that support obtained through in-person interactions and digital platforms each positively affected student well-being, particularly life satisfaction. This dual influence mirrors recent findings by Han Mo, Ma [99], who observed in a survey of over 26,000 students that offline and online social support were independently associated with greater life satisfaction. Such parallels suggest that online-mediated support (for example, encouragement in social media or virtual forums) can supplement traditional in-person support in buffering stress and enhancing happiness. However, our review also points to significant qualitative differences. Consistent with results from Han Mo, Ma [99], strong in-person ties often provided a deeper level of support that online networks could not fully replace. Likewise, Hossain, Islam [17] noted that during the COVID-19 pandemic, active social interactions online had a more significant impact on young people’s well-being than passive online behaviours. This finding underscores that the quality of online engagement matters: using online platforms to connect with others genuinely can buffer stress, much like face-to-face support does. Our review suggests that while virtual support cannot entirely substitute for in-person connection, it is a valuable complement.

### 4.3. Role of individual traits and mediators

Our results also identify multiple individual characteristics that influence how social support affects well-being, either as mediators or moderators. Emotional intelligence (EI) emerged as one such key factor. This observation is consistent with recent work showing that emotional intelligence correlates positively with perceived social support and predicts greater happiness and life satisfaction in college populations [100]. In other words, emotionally intelligent students may be more adept at forming supportive relationships and utilizing them to cope with academic or personal stress. Another vital mechanism identified in our review is resilience. In several studies, social support contributed to building students’ resilience and enhancing their well-being. This aligns with findings by Yıldırım and Tanrıverdi [96], who demonstrated that social support significantly predicted higher resilience, which then mediated increases in life satisfaction among college students. In our synthesis, students with ample support were more likely to bounce back from setbacks, partly because supportive interactions help develop optimism, coping skills, and a sense of belonging – all core components of resilience [52,101,102]. The role of resilience as a mediator is well-supported in the specialized literature and provides a deeper explanation for how social support exerts its beneficial effects by fostering internal strengths that carry students through difficulties. Beyond emotional intelligence and resilience, other personal factors like gratitude [103] and forgiveness [104] may condition the support–well–being linked. Recognizing these mediators emphasizes that students are not just passive recipients of support; their traits critically shape how support is perceived and translated into well-being outcomes.

### 4.4. Self-disclosure and support dynamics

Another theme in our review is the importance of self-disclosure – the willingness to communicate one’s feelings, challenges, or needs. Several studies suggested that students who openly disclose their struggles tend to receive more effective support from peers and family, thereby improving their well-being. Recent evidence supports this idea: it was found that college students’ self-disclosure on social media positively influenced the social support they received, which in turn boosted their subjective well-being [17]. This helps explain findings in our review that students who were more communicative about stress (whether online or offline) often experienced stress relief and higher life satisfaction – their openness likely elicited emotional or informational support that buffered their distress. However, a critical interpretation is that the benefits of self-disclosure can depend on individual and contextual factors. For instance, Desjarlais [105] observed that online self-disclosure improved well-being primarily for students with high social anxiety by increasing their feelings of social connectedness.

In contrast, more socially confident students did not experience the same benefit and could even feel worse with excessive sharing. Such nuances suggest that while encouraging students to seek help and communicate is generally beneficial, it must be tailored to individual needs. In our review, no uniform “dose” of self-disclosure was ideal for all; adequate support often arose when students disclosed to responsive, caring others in their network. The specialized literature thus reinforces that self-disclosure is a double-edged sword – it can significantly enhance well-being if met with understanding and support but may yield frustration or “oversharing” fatigue if the audience or context is not supportive. These insights highlight the complex interplay between personal communication habits and the social support process among college students.

This systematic review has some limitations that might affect how thoroughly it covers the topic. For example, the authors focused only on empirical research, meaning I did not include other types of literature, such as review papers, theoretical discussions, books, or book chapters. The review looked exclusively at peer-reviewed journal articles, leaving out newer insights from recent conference presentations or book chapters. Moreover, while our review provides a comprehensive synthesis of existing literature, it is essential to note that our search was limited to English-language studies. Expanding the search to include studies published in other languages, especially those relevant to East Asian contexts, could reveal additional insights and cultural nuances regarding the role of social support in college students’ well-being. Since this review was limited to studies published in English, future research could benefit from international partnerships to explore and include sources in other languages, helping to create a broader, more global understanding of the topic. Finally, employing descriptive and thematic analyses rather than meta-analytical methods allowed this study to uncover nuanced insights and detailed narratives; however, this method limits the ability to draw generalizable conclusions about intervention effectiveness or other explored phenomena.

Despite these limitations, this commentary offers guidance for future research. Most reviewed studies used cross-sectional designs, limiting the ability to establish causality. Future research should employ longitudinal and experimental designs to understand better the directional effects of social support, individual traits, and well-being over time. Such studies would also enable testing interventions to enhance social support and related factors. Future studies should examine how cultural differences and identity factors influence the impact of social support. This would help us understand how these factors shape the experiences and effectiveness of social support on well-being outcomes.

Additionally, while some research has examined the influence of online social networks on students’ well-being, further investigation is necessary to clarify the specific mechanisms, strengths, and limitations associated with online forms of social support, particularly given the fast-paced development of digital platforms. Moreover, future research should consider institutional and contextual variables, including university policies, campus culture, resource availability, community engagement, and socioeconomic factors, to understand better their impact on the accessibility, utilization, and effectiveness of social support for college students. To advance our understanding of the relationship between social support and well-being among college students, future research should prioritize qualitative methodologies that delve into students’ lived experiences and perceptions. While quantitative studies have provided valuable insights, qualitative approaches can offer a more nuanced understanding of how students perceive and utilize social support in their daily lives. Finally, drawing from current research’s theoretical and practical insights, future studies should emphasize developing, implementing, and thoroughly evaluating interventions and programs to strengthen social support systems, foster personal attributes such as resilience and emotional intelligence, and improve overall well-being among college students.

## 5. Conclusions

This in-depth review of studies on social support, individual characteristics, and subjective well-being in college students offers essential theoretical insights. It builds upon and enriches existing theories that emphasize the pivotal role of social support in managing stress and improving emotional health. It also clarifies how various forms of support—such as emotional encouragement, practical help, or advice—from different sources, including family, peers, and romantic partners, influence student well-being across diverse cultural contexts. The review highlights the multifaceted benefits of social support and emphasizes the need to cultivate supportive social environments within educational settings. The findings further suggest that strengthening family relationships and fostering healthy romantic partnerships can enhance student well-being, positively affecting their emotional health. For stakeholders in higher education, including policymakers, university administrators, mental health professionals, and educators—these findings underscore the critical role of social support in promoting student well-being and provide a framework for developing and implementing effective support strategies and interventions.

## Supporting information

S1 File PRISMA checklist. (PDF)

S2 File Full search strategy and results. (PDF)

S3 File Numbered table of all 2679 studies. (PDF)

S4 File Crowe Critical Appraisal Tool (CCAT) form. (PDF)

S5 File Critical appraisal of included studies. (PDF)

S6 File Risk_Bias_Quality_Table. (PDF)

S7 File The characteristics of the included studies. (PDF)

S8 File Wellbeing and Social Support Measurement. (PDF)
