# Supplementary material for: A systematic review on the impact of social support on college students’ wellbeing and mental health
Source: PLoS One. 2025 Jul 11;20(7):e0325212. doi: 10.1371/journal.pone.0325212 (PMC12250717; doi:10.1371/journal.pone.0325212)
Supplement: S1 File — (PDF) [file pone.0325212.s001.pdf]

Supporting information

S1 File: PRISMA checklist

Section and Topic	Item #	Checklist item	A location where the item is reported
**TITLE**			
Title	1	Identify the report as a systematic review.	Title: Page 1
**ABSTRACT**			
Abstract	2	See the PRISMA 2020 for Abstracts checklist.	In abstract; Pages 1-2
**INTRODUCTION**			
Rationale	3	Describe the rationale for the review in the context of existing knowledge.	In the introduction, Pages 3-8
Objectives	4	Provide an explicit statement of the objective(s) or question(s) the review addresses.	In the introduction, Pages 9-10
**METHODS**			
Eligibility criteria	5	Specify the inclusion and exclusion criteria for the review and how studies were grouped for the syntheses.	In Methods 2.2; Page 12-13
Information sources	6	Specify all databases, registers, websites, organizations, reference lists, and other sources searched or consulted to identify studies. Specify the date when each source was last searched or consulted.	In Methods 2.1; Page 10-12
Search strategy	7	Present the complete search strategies for all databases, registers, and websites, including any filters and limits used.	In Methods 2.1; Page 10-12
Selection process	8	Specify the methods used to decide whether a study met the review's inclusion criteria, including how many reviewers screened each record and each report retrieved, whether they worked independently, and, if applicable, details of automation tools used in the process.	In Methods 2.3; Page 13-14
Data collection	9	Specify the methods used to collect data from reports, including how	In Methods 2.3; Page 13-14

process		many reviewers collected data from each report, whether they worked independently, any processes for obtaining or confirming data from study investigators, and, if applicable, details of automation tools used in the process.	
Data items	10a	List and define all outcomes for which data were sought. Specify whether all results compatible with each outcome domain in each study were sought (e.g., for all measures, time points, analyses), and if not, the methods used to decide which results to collect.	In Methods 2.5; Page 15-16<br>In the results, Pages 17-28
	10b	List and define all other variables for which data were sought (e.g., participant and intervention characteristics, funding sources). Describe any assumptions made about any missing or unclear information.	In the S6 File: Wellbeing and Social Support measurement<br>In S5 File: The characteristics of the included studies.
Study risk of bias assessment	11	Specify the methods used to assess the risk of bias in the included studies, including details of the tool(s) used, how many reviewers assessed each study, whether they worked independently, and, if applicable, details of automation tools used in the process.	In Methods 2.4; Page 15
Effect measures	12	Specify the effect measure(s) (e.g., risk ratio and mean difference) used in synthesizing or presenting results for each outcome.	In Table 3, Page 20<br>In Table 4, Page 22
Synthesis methods	13a	Describe the processes to decide which studies were eligible for each synthesis (e.g., tabulating the study intervention characteristics and comparing against the planned groups for each synthesis (item #5)).	In Methods 2.4; Page 15
	13b	Describe any methods required to prepare the data for presentation or synthesis, such as handling missing summary statistics or data conversions.	n/a
	13c	Describe any methods used to tabulate or visually display the results of individual studies and syntheses.	In The S5 File, the characteristics of the included studies are described.

	13d	Describe any methods used to synthesize results and provide a rationale for the choice(s). If meta-analysis was performed, describe the model(s), method(s) to identify the presence and extent of statistical heterogeneity, and software package(s) used.	In Methods 2.4 Page 15;<br>In S5 File: The characteristics of the included studies.<br>In S3 File: Crowe Critical Appraisal Tool (CCAT) form
	13e	Describe any methods to explore possible causes of heterogeneity among study results (e.g., subgroup analysis, meta-regression).	n/a
	13f	Describe any sensitivity analyses conducted to assess the robustness of the synthesized results.	n/a
Reporting bias assessment	14	Describe any methods used to assess the risk of bias due to missing results in a synthesis (arising from reporting biases).	In Methods 2.4 Page 15;<br>In S5 File: The characteristics of the included studies.<br>In S3 File: Crowe Critical Appraisal Tool (CCAT) form
Certainty assessment	15	Describe any methods used to assess certainty (or confidence) in the body of evidence for an outcome.	In Methods 2.4 Page 15;<br>In S5 File: The characteristics of the included studies.<br>In S3 File: Crowe Critical Appraisal Tool (CCAT) form
**RESULTS**			
Study selection	16a	Describe the search and selection process results, from the number of records identified in the search to the number of studies included in the review, ideally using a flow diagram.	In results 2.3, Pages 13-14<br>In figure1：PRISMA flow diagram; Page 15
	16b	Cite studies that might appear to meet the inclusion criteria but which were excluded, and explain why they were excluded.	In figure1：PRISMA flow diagram; Page 15
Study characteristics	17	Cite each included study and present its characteristics.	In S5 File: The characteristics of the

			included studies.
Risk of bias in studies	18	Present assessments of risk of bias for each included study.	In Methods 2.4 Page 15;<br>In S5 File: The characteristics of the included studies.<br>In S3 File: Crowe Critical Appraisal Tool (CCAT) form
Results of individual studies	19	For all outcomes, present, for each study: (a) summary statistics for each group (where appropriate) and (b) an effect estimate and its precision (e.g., confidence/credible interval), ideally using structured tables or plots.	In the results, Pages 17-31
Results of syntheses	20a	For each synthesis, briefly summarise the characteristics and risk of bias among contributing studies.	In the results, Pages 17-31
	20b	Present results of all statistical syntheses conducted. If meta-analysis was done, present for each the summary estimate and its precision (e.g., confidence/credible interval) and measures of statistical heterogeneity. If comparing groups, describe the direction of the effect.	n/a
	20c	Present results of all investigations of possible causes of heterogeneity among study results.	In the results, Pages 17-31
	20d	Present all sensitivity analyses conducted to assess the robustness of the synthesized results.	n/a
Reporting biases	21	Present assessments of risk of bias due to missing results (arising from reporting biases) for each synthesis assessed.	n/a
Certainty of evidence	22	Present assessments of certainty (or confidence) in the body of evidence for each outcome assessed.	n/a
**DISCUSSION**			
Discussion	23a	Provide a general interpretation of the results in the context of other evidence.	In discussion: Pages 31-32
	23b	Discuss any limitations of the evidence included in the review.	In discussion: Pages 33-34

	23c	Discuss any limitations of the review processes used.	In discussion: Pages 33-34
	23d	Discuss the implications of the results for practice, policy, and future research.	In discussion: Pages 34-35
**OTHER INFORMATION**			
Registration and protocol	24a	Provide registration information for the review, including the register name and registration number, or state that the review was not registered.	In Methods 2, Page 10
	24b	Indicate where the review protocol can be accessed or state that a protocol was not prepared.	In Methods 2, Page 10
	24c	Describe and explain any amendments to information provided at registration or in the protocol.	n/a
Support	25	Describe sources of financial or non-financial support for the review and the role of the funders or sponsors.	In Author statements; Page 36
Competing interests	26	Declare any competing interests of review authors.	In the Author's statements, Page 37
Availability of data, code, and other materials	27	Report which of the following are publicly available and where they can be found: template data collection forms; data extracted from included studies; data used for all analyses; analytic code; any other materials used in the review.	In Data Availability Statement; Page 37
