# Supplementary material for: A systematic review on the impact of social support on college students’ wellbeing and mental health
Source: PLoS One. 2025 Jul 11;20(7):e0325212. doi: 10.1371/journal.pone.0325212 (PMC12250717; doi:10.1371/journal.pone.0325212)
Supplement: S2 File — (PDF) [file pone.0325212.s002.pdf]

Supporting information

S2 File: Full search strategy and results

No.	Database	Complete Search Strategy	Results
1	Web of Science	"Social support"OR"social relation*" OR "social network*"OR “family support “OR "family relation*"OR"emotional support"OR"financial support"OR"instrumental support"OR"tangible support"OR"informational support"OR"appraisal support" (Topic) and “wellbeing” OR “wellbeing” OR “psychological wellbeing” OR “psychological wellbeing” OR “happiness” OR “flourish” OR “flourishing” OR “psychological flourish” OR “psychological flourishing” OR “subjective wellbeing” OR “subjective wellbeing” OR “positive emotions” OR “positive emotion” OR “positive affect” OR “engagement” OR “flow” OR “psychological flow” OR “positive relationship” OR “positive relationships” OR “social support” OR “meaning” OR “meaning of life” OR “meaning in life” OR “life meaning” OR “life purpose” OR “purpose of life” OR “purpose in life” OR “achievement” OR “achievements” OR “accomplishment” OR “accomplishments” OR “performance” OR “success” (Title) and "college students"OR"undergraduates" (Topic) and Article (Document Types) and English (Languages) and 2023 or 2022 or 2024 or 2021 or 2020 or 2019 or 2018 or 2012 or 2013 or 2014 or 2015 or 2016 or 2017 or 2011 or 2010 (Final Publication Year)	707
2	Scopus	(TITLE-ABS-KEY ("Social support" OR "social relation*" OR "social network*" OR "family support" OR "family relation*" OR "emotional support" OR "financial support" OR "instrumental support" OR "tangible support" OR "informational support" OR "appraisal support") AND TITLE ("wellbeing" OR "wellbeing" OR "psychological wellbeing" OR "psychological wellbeing" OR "happiness" OR "flourish" OR "flourishing" OR "psychological flourish" OR "psychological flourishing" OR "subjective	700

		wellbeing" OR "subjective wellbeing" OR "positive emotions" OR "positive emotion" OR "positive affect" OR "engagement" OR "flow" OR "psychological flow" OR "positive relationship" OR "positive relationships" OR "social support" OR "meaning" OR "meaning of life" OR "meaning in life" OR "life meaning" OR "life purpose" OR "purpose of life" OR "purpose in life" OR "achievement" OR "achievements" OR "accomplishment" OR "accomplishments" OR "performance" OR "success") AND TITLE-ABS-KEY ("college students" OR "undergraduates")) AND PUBYEAR > 2009 AND PUBYEAR < 2025 AND (LIMIT-TO (DOCTYPE , "are")) AND (LIMIT-TO (LANGUAGE , "English"))	
3	PsycINFO	SU ("Social support" OR “social relation*" OR "social network*"OR" family support"OR "family relation*"OR “emotional support” OR" financial support"OR"instrumental support"OR"tangible support” OR" informational support “OR" appraisal support") AND TI (“wellbeing” OR “wellbeing” OR “psychological wellbeing” OR “psychological wellbeing” OR “happiness” OR “flourish” OR “flourishing” OR “psychological flourish” OR “psychological flourishing” OR “subjective wellbeing” OR “subjective wellbeing” OR “positive emotions” OR “positive emotion” OR “positive affect” OR “engagement” OR “flow” OR “psychological flow” OR “positive relationship” OR “positive relationships” OR “social support” OR “meaning” OR “meaning of life” OR “meaning in life” OR “life meaning” OR “life purpose” OR “purpose of life” OR “purpose in life” OR “achievement” OR “achievements” OR “accomplishment” OR “accomplishments” OR “performance” OR “success”) AND SU "college students"OR"undergranduates"	406
4	ProQuest	abstract("Social support" OR "social relation*" OR "social network*" OR "family support" OR "family relation*" OR "emotional support" OR "financial	370

		support" OR "instrumental support" OR "tangible support" OR "informational support" OR "appraisal support") AND title("wellbeing" OR "wellbeing" OR "psychological wellbeing" OR "psychological wellbeing" OR "happiness" OR "flourish" OR "flourishing" OR "psychological flourish" OR "psychological flourishing" OR "subjective wellbeing" OR "subjective wellbeing" OR "positive emotions" OR "positive emotion" OR "positive affect" OR "engagement" OR "flow" OR "psychological flow" OR "positive relationship" OR "positive relationships" OR "social support" OR "meaning" OR "meaning of life" OR "meaning in life" OR "life meaning" OR "life purpose" OR "purpose of life" OR "purpose in life" OR "achievement" OR "achievements" OR "accomplishment" OR "accomplishments" OR "performance" OR "success") AND abstract("college students" OR "undergraduates")Limits applied	
5	PubMed	(("Social support"OR"social relation*"[Title/Abstract] OR "social network*"OR “family support” OR "family relation*"OR"emotional support"OR"financial support"OR"instrumental support"OR"tangible support"OR"informational support"OR"appraisal support"[Title/Abstract]) AND ("wellbeing"[Title/Abstract] OR "wellbeing"[Title/Abstract] OR "psychological wellbeing"[Title/Abstract] OR "psychological wellbeing"[Title/Abstract] OR "happiness"[Title/Abstract] OR "flourish"[Title/Abstract] OR "flourishing"[Title/Abstract] OR "psychological flourish"[Title/Abstract] OR "psychological flourishing"[Title/Abstract] OR "subjective wellbeing"[Title/Abstract] OR "subjective wellbeing"[Title/Abstract] OR "positive emotions"[Title/Abstract] OR "positive emotion"[Title/Abstract] OR "positive affect"[Title/Abstract] OR "engagement"[Title/Abstract] OR "flow"[Title/Abstract] OR "psychological flow"[Title/Abstract] OR "positive relationship"[Title/Abstract] OR "positive	731

		relationships"[Title/Abstract] OR "social support"[Title/Abstract] OR "meaning"[Title/Abstract] OR "meaning of life"[Title/Abstract] OR "meaning in life"[Title/Abstract] OR "life meaning"[Title/Abstract] OR "life purpose"[Title/Abstract] OR "purpose of life"[Title/Abstract] OR "purpose in life"[Title/Abstract] OR "achievement"[Title/Abstract] OR "achievements"[Title/Abstract] OR "accomplishment"[Title/Abstract] OR "accomplishments"[Title/Abstract] OR "performance"[Title/Abstract] OR "success"[Title/Abstract])) AND ("college students"OR"undergranduates"[Title/Abstract])	
6	Cochrane	"Social support"OR"social relation" OR "social network"OR"family support “OR "family relation"OR"emotional support"OR"financial support"OR"instrumental support"OR"tangible support"OR"informational support"OR"appraisal support" in Title Abstract Keyword AND “wellbeing” OR “wellbeing” OR “psychological wellbeing” OR “psychological wellbeing” OR “happiness” OR “flourish” OR “flourishing” OR “psychological flourish” OR “psychological flourishing” OR “subjective wellbeing” OR “subjective wellbeing” OR “positive emotions” OR “positive emotion” OR “positive affect” OR “engagement” OR “flow” OR “psychological flow” OR “positive relationship” OR “positive relationships” OR “social support” OR “meaning” OR “meaning of life” OR “meaning in life” OR “life meaning” OR “life purpose” OR “purpose of life” OR “purpose in life” OR “achievement” OR “achievements” OR “accomplishment” OR “accomplishments” OR “performance” OR “success” in Title Abstract Keyword AND "college students"OR"undergranduates" in Title Abstract Keyword - (Word variations have been searched)	657
