# Supplementary material for: A systematic review on the impact of social support on college students’ wellbeing and mental health
Source: PLoS One. 2025 Jul 11;20(7):e0325212. doi: 10.1371/journal.pone.0325212 (PMC12250717; doi:10.1371/journal.pone.0325212)
Supplement: S3 File — (PDF) [file pone.0325212.s003.pdf]

# Supporting information

**S3 File: Numbered table of all 2679 studies**

| No. | Author(s) | Year | Title | Journal | Reason for Exclusion |
|---|---|---|---|---|---|
| 1 | Greger, H. K.;Stuifbergen, M. C.;Jozefiak, T.;Kayed, N. S.;Lydersen, S.;Rimehaug, T.;Schalinski, I.;Seim, A. R.;Singstad, M. T.;Wallander, J.;Wichstrøm, L.;Lehmann, S.; | 2024 | Young Adults with a History of Residential Youth Care: A Cohort Profile of a Hard-to-Reach Population | International journal of environmental research and public health | The population is not college students |
| 2 | Bridges, Frank;Appel, Lora;Grossklags, Jens; | 2012 | Young Adults' Online Participation Behaviors: An Exploratory Study of Web 2.0 Use for Political Engagement | Information Polity | The population is not college students |
| 3 | Anderson, C.;Butt, C.; | 2017 | Young Adults on the Autism Spectrum at College: Successes and Stumbling Blocks | Journal of Autism and Developmental Disorders | The population is not college students |
| 4 | Nandi, S.;Singh, S.; | 2021 | Young adults' engagement with Facebook advertisements: An exploratory study using a theory of planned behavior | Indian Journal of Marketing | The population is not college students |
| 5 | Wei, Z. Y.;Li, Y. F.;Liu, L. M.;Wu, X. C.;Qiao, Z. H.;Wang, W. C.; | 2024 | You are worth it: Social support buffered the relationship between impostor syndrome and suicidal ideation. | Journal of Pacific Rim Psychology | Mental health disorders |
| 6 | Shin, J. E.; | 2019 | You are my happiness: Socially enriched happiness belief predicts life satisfaction, | Cogn Emot | Not mental health or wellbeing outcomes |

| | | | | | |
|---|---|---|---|---|---|
| | | | especially among the poor. | | |
| 7 | Pacanowski, C. R.;Diers, L.;Crosby, R. D.;Mackenzie, M.;Neumark-Sztainer, D.; | 2020 | Yoga's impact on risk and protective factors for disordered eating: a pilot prevention trial | Eating disorders | Mental health disorders |
| 8 | Li, J.;Yang, D.;Hu, Z.; | 2022 | Wuhan College Students' Self-Directed Learning and Academic Performance: Chain-Mediating Roles of Optimism and Mental Health | Frontiers in Psychology | Not related wellbeing |
| 9 | Mawjee, K.;Woltering, S.;Tannock, R.; | 2015 | Working Memory Training in Post-Secondary Students with ADHD: a Randomized Controlled Study | PLoS ONE | Mental health disorders |
| 10 | Gropper, R. J.;Gotlieb, H.;Kronitz, R.;Tannock, R.; | 2014 | Working memory training in college students with ADHD or LD | Journal of Attention Disorders | Mental health disorders |
| 11 | van der Schaaf, M. E.;Fallon, S. J.;Ter Huurne, N.;Buitelaar, J.;Cools, R.; | 2013 | Working memory capacity predicts the effects of methylphenidate on reversal learning. | Neuropsychopharmacology | Mental health disorders |
| 12 | D'Orta, I.;Weber, K.;Herrmann, F. R.;Giannakopoulos, P.; | 2024 | Women in acute forensic psychiatric care: comparison of clinical, sociodemographic, and detention-related characteristics in pretrial detention, sentence execution, and court-ordered treatment | BMC Psychiatry | Mental health disorders |
| 13 | Ward, J.;McBride, A.;Gudka, R.;Becker, K.;Newlove-Delgado, T.;Price, A.; | 2024 | Wider health needs in attention deficit hyperactivity disorder from lived and professional experience: A qualitative framework analysis | BMJ Open | Mental health disorders |
| 14 | Mizusaki, Y.;Ikudome, S.;Ishii, Y.;Unenaka, S.;Funo, T.;Takeuchi, T.;Ogasa, K.;Mori, S.;Nakamoto, H.; | 2019 | Why does the Quiet Eye improve aiming accuracy? Testing a motor preparation hypothesis with brain potential | Cognitive processing | Mental health disorders |

| | | | | | |
|---|---|---|---|---|---|
| 15 | Domínguez, T.;Puebla, D. P.;Fresán, A.;Sheinbaum, T.;Nieto, L.;Robles, R.;López, S. R.;de la Fuente-Sandoval, C.;Lara Muñoz, M. D. C.;Barrantes-Vidal, N.;Celada-Borja, C. A.;Rosel-Vales, M.;Saracco, R.; | 2024 | Why do some Mexicans with psychosis risk symptoms seek mental health care and others do not? | Psychiatry Research | Not related wellbeing |
| 16 | Fritz, H. L.; | 2020 | Why are humor styles associated with well-being, and does social competence matter? Examining relations to psychological and physical well-being, reappraisal, and social support | Personality and Individual Differences | Not related to research questions |
| 17 | Liew, S. L.;Ma, Y.;Han, S.;Aziz-Zadeh, L.; | 2011 | Who's Afraid of the Boss: Cultural Differences in Social hierarchies modulate self-face recognition in Chinese and Americans. | PLoS ONE | Not related to research questions |
| 18 | Alfaro, E. C.;Weimer, A. A.;Castillo, E.; | 2018 | Who Helps Build Mexican-Origin Female College Students' Self-Efficacy? The Role of Important Others in Student Success | Hispanic Journal of Behavioral Sciences | Not related to research questions |
| 19 | Ostafin, B. D.;Palfai, T. P.; | 2012 | When wanting to change is not enough: automatic appetitive processes moderate the effects of a brief alcohol intervention in hazardous-drinking college students. | Addiction science & clinical practice | Mental health disorders |
| 20 | Park, J.;Lee, D. S.;Shablack, H.;Verduyn, P.;Deldin, P.;Ybarra, O.;Jonides, J.;Kross, E.; | 2016 | When perceptions defy reality: The relationships between depression and actual and perceived Facebook social support | Journal of Affective Disorders | Mental health disorders |
| 21 | Alshahrani, A.;Siddiqui, A.;Khalil, S.;Farag, S.;Alshahrani, | 2021 | WhatsApp-based intervention for promoting physical activity among female college students, | La revue de sante de la Mediterranee | Mental health disorders |

| | | | | | |
|---|---|---|---|---|---|
| | N.;Alsabaani, A.;Korairi, H.; | | Saudi Arabia: a randomized controlled trial | orientale / al-Majallah al-sihhiyah li-sharq al-mutawassit [Eastern Mediterranean health journal] | |
| 22 | Skurka, C.;Reynolds-Tylus, T.;Quick, B.;Hartman, D.; | 2020 | What's at Stake: evaluating a Run-Hide-Fight® Intervention Video through the Lens of Vested Interest Theory | Journal of Health Communication | Mental health disorders |
| 23 | Christie, Charlene; | 2021 | What Is Hidden Can Still Hurt: Concealable Stigma, Psychological Well-Being, and Social Support Among LGB College Students | Sexuality Research & Social Policy | Not related |
| 24 | Lu, Guoqing;Xie, Kui;Liu, Qingtang; | 2022 | What influences student situational engagement in smart classrooms: Perception of the learning environment and students' motivation | British Journal of Educational Technology | Not related |
| 25 | Li, X. Q.;Chen, W. H.;Popiel, P.; | 2015 | What happens on Facebook stays on Facebook? The implications of Facebook interaction for perceived, receiving, and giving social support | Computers in Human Behavior | Not related |
| 26 | Choi, J.;Moon, H.;Park, M.; | 2023 | What factors influence continuous usage intention of head-mounted display-based virtual reality content?: a cross-sectional survey | Korean J Women Health Nurs | Not related |
| 27 | Doyle, A.;Healy, O.;Paterson, J.;Lewis, K.;Treanor, D.; | 2024 | What does an ADHD-friendly university look like? A case study from Ireland | International Journal of Educational Research Open | Mental health disorders |

| | | | | | |
|---|---|---|---|---|---|
| 28 | Wang, N.;Ye, J. H.;Gao, W.;Lee, Y. S.;Zeng, L.;Wang, L.; | 2024 | What do they Need?-The academic counseling needs of students majoring in art and design in a higher vocational college in China | Heliyon | Not related |
| 29 | Niu, X.;Niu, Z.;Wang, M.;Wu, X.; | 2022 | What are the key drivers to promote the entrepreneurial intention of vocational college students? An empirical study based on structural equation modeling | Front Psychol | Mental health disorders |
| 30 | Meredith, C.;McKerchar, C.;Haitana, T.;Pitama, S.; | 2024 | Whāia te it kahurangi: Seeking perinatal mental health equity. Māori offer solutions for the health system. | Mental Health and Prevention | Not related |
| 31 | Lillehei, A. S.;Halcon, L.;Gross, C. R.;Savik, K.;Reis, R.; | 2016 | Well-Being and Self-Assessment of Change: secondary Analysis of an RCT That Demonstrated Benefit of Inhaled Lavender and Sleep Hygiene in College Students with Sleep Problems | Explore (New York, N.Y.) | Mental health disorders |
| 32 | Lytle, L. A.;Laska, M. N.;Linde, J. A.;Moe, S. G.;Nanney, M. S.;Hannan, P. J.;Erickson, D. J.; | 2017 | Weight-Gain Reduction Among 2-Year College Students: the CHOICES RCT | American journal of preventive medicine | Mental health disorders |
| 33 | Hou, J.;Ndasauka, Y.;Pan, X.;Chen, S.;Xu, F.;Zhang, X.; | 2018 | Weibo or WeChat? Assessing Preference for Social Networking Sites and Role of Personality Traits and Psychological Factors | Front Psychol | Not related |
| 34 | Pang, H.; | 2018 | WeChat use is significantly correlated with college students' quality of friendships but not with perceived well-being. | Heliyon | Not related |
| 35 | Chen, Y.; | 2017 | WeChat use among Chinese college students: Exploring gratifications and political engagement in China | Journal of International and Intercultural | Not related |

| | | | | | |
|---|---|---|---|---|---|
| | | | | Communication | |
| 36 | Chen, Y. X.;Li, R. X.;Zhang, P.;Liu, X. P.; | 2021 | WeChat engagement styles: Measuring the two processes of relatedness need, moderated by personality differences | Current Psychology | Not related |
| 37 | Poddar, K. H.;Hosig, K. W.;Anderson, E. S.;Nickols-Richardson, S. M.;Duncan, S. E.; | 2010 | Web-based nutrition education intervention improves self-efficacy and self-regulation related to increased dairy intake in college students. | J Am Diet Assoc | Published not from January 2010 to 31 March 2024 |
| 38 | Gilbertson, R. J.;Norton, T. R.;Beery, S. H.;Lee, K. R.; | 2018 | Web-Based Alcohol Intervention in First-Year College Students: efficacy of Full-Program Administration Prior to Second Semester | Substance use & misuse | Mental health disorders |
| 39 | Levin, M. E.;Haeger, J. A.;Pierce, B. G.;Twohig, M. P.; | 2017 | Web-Based Acceptance and Commitment Therapy for Mental Health Problems in College Students: a Randomized Controlled Trial | Behavior Modification | Not related |
| 40 | Tutlam, N. T.;Chang, J. J.;Byansi, W.;Flick, L. H.;Ssewamala, F. M.;Betancourt, T. S.; | 2024 | War-Affected South Sudanese in Settings of Preflight, Flight, and Resettlement: a Systematic Review and Meta-analysis of Trauma-Associated Mental Disorders | Global Social Welfare | Meta review |
| 41 | Galovski, T. E.;Nixon, R. D. V.;Kehle-Forbes, S.; | 2024 | Walking the line between fidelity and flexibility: A conceptual review of personalized approaches to manualized treatments for posttraumatic stress disorder | Journal of Traumatic Stress | Mental health disorders |
| 42 | Ryan, J. J.;Swopes-Willhite, N.;Franklin, C.;Kreiner, D. S.; | 2015 | WAIS-IV administration errors: effects of altered response requirements on Symbol Search and violation of standard surface-variety patterns on Block Design | Applied neuropsychology. Adult | Not related to research questions |

| | | | | | |
|---|---|---|---|---|---|
| 43 | Geng, Y.;Huang, C. C.;Deng, G. S.;Cheung, S. P.;Liao, J. Y.; | 2022 | Volunteering and Psychological well-being in college students in China | Asian Social Work and Policy Review | Not related to research questions |
| 44 | Xie, Qiuzhu;Liu, Xiaobin;Zhang, Nanyan;Zhang, Qianqian;Jiang, Xijuan;Wen, Lijun; | 2021 | Vlog-Based Multimodal Composing: Enhancing EFL Learners' Writing Performance | Applied Sciences | Not related to research questions |
| 45 | Woltering, S.;Gu, C.;Liu, Z. X.;Tannock, R.; | 2021 | Visuospatial Working Memory Capacity in the Brain After Working Memory Training in College Students With ADHD: a Randomized Controlled Trial | Journal of Attention Disorders | Mental health disorders |
| 46 | Kurane, K.;Lin, N.;Dan, I.;Tanaka, H.;Tsuji, Y.;Ito, W.;Yanagida, S.;Monden, Y.; | 2024 | Visualizing changes in cerebral hemodynamics in children with ADHD who have discontinued methylphenidate: A pilot study on using brain function for medication discontinuation decisions | Brain and Development | Mental health disorders |
| 47 | Bagheri, S.;Good, J.;Alavi, H. S.; | 2024 | Visual and acoustic discomfort: A comparative study of impacts on individuals with and without ADHD using electroencephalogram (EEG) | Building and Environment | Mental health disorders |
| 48 | Wiebe, A.;Selaskowski, B.;Paskin, M.;Asché, L.;Pakos, J.;Aslan, B.;Lux, S.;Philipsen, A.;Braun, N.; | 2024 | Virtual reality-assisted prediction of adult ADHD based on eye tracking, EEG, actigraphy and behavioral indices: a machine learning analysis of independent training and test samples | Translational Psychiatry | Mental health disorders |
| 49 | Binfet, J. T.;Tardif-Williams, C.;Draper, Z. A.;Green, F. L. L.;Singal, A.;Rousseau, C. X.;Roma, R.; | 2022 | Virtual Canine Comfort: A Randomized Controlled Trial of the Effects of a Canine-Assisted Intervention Supporting Undergraduate Wellbeing | Anthrozoos | Mental health disorders |
| 50 | Hust, S. J. T.;Rodgers, K. B.;Cameron, N.;Li, J.; | 2019 | Viewers' Perceptions of Objectified Images of Women in Alcohol Advertisements and Their | Journal of Health Communication | Mental health disorders |

| | | | | | |
|---|---|---|---|---|---|
| | | | Intentions to Intervene in Alcohol-Facilitated Sexual Assault Situations | | |
| 51 | Rutakumwa, R.;Knizek, B. L.;Tusiime, C.;Mpango, R. S.;Birungi, C.;Kinyanda, E.; | 2024 | Victimisation in the life of persons with severe mental illness in Uganda: a pluralistic qualitative study | BMC Psychiatry | Not related |
| 52 | Weber, M. C.;Jendro, A. M.;Fischer, E. P.;Drummond, K. L.;Haltom, T. M.;Hundt, N. E.;Cucciare, M. A.;Pyne, J. M.; | 2024 | Veterans' Experiences of and Preferences for Patient-Centered, Measurement-Based PTSD Care | Medical Care | Mental health disorders |
| 53 | Liu, W.;Wang, W.;Wang, Z.;Xing, Y.; | 2024 | Ventral tegmental area dopaminergic circuits participate in stress-induced chronic postsurgical pain in male mice. | BMC Neuroscience | Mental health disorders |
| 54 | Anderson, J. R.;Maki, K. C.;Palacios, O. M.;Edirisinghe, I.;Burton-Freeman, B.;Spitznagel, M. B.; | 2021 | Varying roles of glucoregulatory function measures in postprandial cognition following milk consumption | European journal of nutrition | Not related |
| 55 | Souza, A. S.;Pontes, T. N.;Abreu-Rodrigues, J.; | 2012 | Varied but not necessarily random: human performance under variability contingencies is affected by instructions. | Learning & behavior | Not related |
| 56 | Ramírez-Martínez, F. R.;Villanos, M. T.;Sharma, S.;Leiner, M.; | 2024 | Variations in anxiety and emotional support among first-year college students across different learning modes (distance and face-to-face) during COVID-19 | PLoS ONE | Mental health disorders |
| 57 | Babarczy, A.;Dobó, D.;Nagy, P.;Mészáros, A.;Lukács, Á; | 2024 | Variability of theory of mind versus pragmatic ability in typical and atypical development | Journal of Communication Disorders | Not related |

| | | | | | |
|---|---|---|---|---|---|
| 58 | Dietch, J. R.;Sethi, K.;Slavish, D. C.;Taylor, D. J.; | 2019 | Validity of two retrospective questionnaire versions of the Consensus Sleep Diary: the whole week and split week Self-Assessment of Sleep Surveys | Sleep medicine | Not related |
| 59 | Tsai, J. D.;Sun, H. Y.;Kuo, H. Y.;Chu, S. Y.;Lee, Y. W.;Lu, H. H.; | 2024 | Validity of specific CPT indices in differentiating school-aged children previously diagnosed with attention-deficit/hyperactivity disorder from school-aged children with non-attention deficit/hyperactivity disorder in general education classrooms: a case | BMC Pediatrics | The population is not college students |
| 60 | Soares, A. B.;dos Santos, A. A.;Jardim, M. E. D.;Queluz, Fnfr; | 2023 | Validity Evidences of the Social Support Scale for University Students | Revista Iberoamericana De Diagnostico Y Evaluacion-E Avaliacao Psicologica | Not related |
| 61 | Hupfeld, K. E.;Osborne, J. B.;Tran, Q. T.;Hyatt, H. W.;Abagis, T. R.;Shah, P.; | 2024 | Validation of the dispositional adult hyperfocus questionnaire (AHQ-D) | Scientific Reports | Not related |
| 62 | Arenas Dávila, A. M.;Pastrana Arias, K.;Castaño Ramírez, Ó M.;Van den Enden, P.;Castro Navarro, J. C.;González Giraldo, S.;Vera Higuera, D. M.;Harris, K. M.; | 2024 | Validation of the Colombian–Spanish Suicidality Scale for Screening Suicide Risk in Clinical and Community Settings | Journal of Clinical Medicine | Mental health disorders |
| 63 | Harman, E.;Perkins, S. M.;Pai, A.;Robb, S. L.; | 2024 | Validation of the Abbreviated PTSD Checklist–Civilian as a Traumatic Stress Screener | Journal of Pediatric Hematology/Oncolo | Mental health disorders |

| | | | | | |
|---|---|---|---|---|---|
| | | | for Parents of Children With Cancer | gy Nursing | |
| 64 | Stockton, M. A.;Mazinyo, E. W.;Mlanjeni, L.;Sweetland, A. C.;Scharf, J. Y.;Nogemane, K.;Ngcelwane, N.;Basaraba, C.;Bezuidenhout, C.;Sansbury, G.;Olivier, D.;Grobler, C.;Wall, M. M.;Medina-Marino, A.;Nobatyi, P.;Wainberg, M. L.; | 2024 | Validation of screening instruments for common mental disorders and suicide risk in South African primary care settings | Journal of Affective Disorders | Mental health disorders |
| 65 | Huang, M.;Sun, H.;Chen, H.;Zhang, Y.;Adams, K.;Gao, Z.; | 2023 | Validation of Physical Activity Correlates Questionnaire from Social Ecological Model in College Students | J Clin Med | Not related |
| 66 | Young-Jones, A.;Hart, B.;Yadon, C. A.;Buchanan, E. M.; | 2022 | Validation of a Four-Factor Measure: Scale of Perceived Instructor Support | Psychol Rep | Not related |
| 67 | Duan, W.;Mu, W.; | 2018 | Validation of a Chinese version of the stress overload scale-short and its use as a screening tool for mental health status | Qual Life Res | Mental health disorders |
| 68 | Stockton, M. A.;Mazinyo, E. W.;Mlanjeni, L.;Nogemane, K.;Ngcelwane, N.;Sweetland, A. C.;Basaraba, C. N.;Bezuidenhout, C.;Sansbury, G.;Lovero, K. L.;Olivier, D.;Grobler, C.;Wall, M. M.;Medina-Marino, A.;Nobatyi, P.;Wainberg, M. L.; | 2024 | Validation of a brief screener for broad-spectrum mental and substance-use disorders in South Africa | Global Mental Health | Mental health disorders |

| | | | | | |
|---|---|---|---|---|---|
| 69 | Kleiman, E. M.;Riskind, J. H.; | 2013 | Utilized social support and self-esteem mediate the relationship between perceived social support and suicide ideation. A test of a multiple mediator model | Crisis | Mental health disorders |
| 70 | Salah, E.;Shokair, M.;El-Samie, F. E. A.;Shalaby, W. A.; | 2024 | Utilization of fMRI with optical amplification to diagnose attention deficit hyperactivity disorder | Journal of Optics (India) | Mental health disorders |
| 71 | Moon, I.;Yun, K. K.;Jennings, M.; | 2024 | Utilization and Perceived Need for Mental Health Services Among Homeless Seniors | Community Mental Health Journal | Not related |
| 72 | Lazard, A. J.;Bamgbade, B. A.;Sontag, J. M.;Brown, C.; | 2016 | Using Visual Metaphors in Health Messages: a Strategy to Increase Effectiveness for Mental Illness Communication | Journal of Health Communication | Not related |
| 73 | Magoc, D.;Tomaka, J.;Bridges-Arzaga, A.; | 2011 | Using the web to increase physical activity in college students | American Journal of Health Behavior | Not related |
| 74 | Cleary, T. J.;Bryer, J. M.;Andrade, H. L.; | 2023 | Using the Diagnostic Assessment and achievement of college success (DAACS) to promote SRL skills among entry-level college students: Challenges and recommendations | New Directions for Teaching and Learning | Not related |
| 75 | Brandon-Friedman, R. A.;Kim, H. W.; | 2016 | Using social support levels to predict sexual identity development among college students who identify as a sexual minority | Journal of Gay and Lesbian Social Services | Not related |
| 76 | Godino, J. G.;Merchant, G.;Norman, G. J.;Donohue, M. C.;Marshall, S. J.;Fowler, J. H.;Calfas, K. J.;Huang, J. S.;Rock, C. L.;Griswold, W. G.;et al.,; | 2016 | Using social and mobile tools for weight loss in overweight and obese young adults (Project SMART): a 2-year, parallel-group, randomized, controlled trial | The Lancet. Diabetes & endocrinology | Not related |
| 77 | Fucito, L. M.;DeMartini, K. | 2017 | Using Sleep Interventions to Engage and Treat | Alcoholism, clinical | Mental health disorders |

| | | | | | |
|---|---|---|---|---|---|
| | S.;Hanrahan, T. H.;Yaggi, H. K.;Heffern, C.;Redeker, N. S.; | | Heavy-Drinking College Students: a Randomized Pilot Study | and experimental research | |
| 78 | Nguyen-Feng, V. N.;Greer, C. S.;Frazier, P.; | 2017 | Using online interventions to deliver college student mental health resources: evidence from randomized clinical trials | Psychological services | Mental health disorders |
| 79 | Lai, Jocelyn;Rahmani, Amir;Asal, Yunusova;Rivera, Alexander P.;Labbaf, Sina;Hu, Sirui;Dutt, Nikil;Jain, Ramesh;Borelli, Jessica L.; | 2021 | Using Multimodal Assessments to Capture Personalized Contexts of College Student Well-being in 2020: Case Study | JMIR formative research | Not related |
| 80 | Tran, N. A.;Jean-Marie, G.;Powers, K.;Bell, S.;Sanders, K.; | 2016 | Using Institutional Resources and Agency to Support Graduate Students′ Success at a Hispanic Serving Institution | Education Sciences | Not related |
| 81 | Mukherjee, A.;Yatirajula, S. K.;Kallakuri, S.;Paslawar, S.;Lempp, H.;Raman, U.;Essue, B. M.;Sagar, R.;Singh, R.;Peiris, D.;Norton, R.;Thornicroft, G.;Maulik, P. K.; | 2024 | Using Formative Research to inform a mental health intervention for adolescents living in Indian slums: the ARTEMIS study | Child and Adolescent Psychiatry and Mental Health | Mental health disorders |
| 82 | Knapp, Kyler S.;Cleveland, H. Harrington;Apsley, Hannah B.;Harris, Kitty S.; | 2021 | Using daily diary methods to understand how college students in recovery use social support | Journal of Substance Abuse Treatment | Not related |
| 83 | De Leon, A. N.;Dvorak, R. D.;Smallman, R.;Arthur, K.;Piercey, C.; | 2022 | Using counterfactual thinking theory to change alcohol protective behavioral strategy use intentions | British journal of health psychology | Mental health disorders |
| 84 | Ackland, P. E.;Hagedorn, H. J.;Kenny, M. E.;Salameh, H. | 2024 | Using brief reflections to capture and evaluate end-user engagement: a case example using the | BMC Medical Research | Not related |

| | | | | | |
|---|---|---|---|---|---|
| | A.;Kehle-Forbes, S. M.;Gustavson, A. M.;Karimzadeh, L. E.;Meis, L. A.; | | COMPASS study | Methodology | |
| 85 | Schwebel, D. C.;Hasan, R.;Griffin, R.; | 2020 | Using Bluetooth beacon technology to reduce distracted pedestrian behavior: a cross-over trial study protocol | Injury prevention | Mental health disorders |
| 86 | Johnston, J. D.;Massey, A. P.;Marker-Hoffman, R. L.; | 2012 | Using an alternate reality game to increase physical activity and decrease the obesity risk of college students | Journal of diabetes science and technology | Not related |
| 87 | Rainisch, B. K. W.;Dahlman, L.;Vigil, J.;Forster, M.; | 2022 | Using a multi-module web app to prevent substance use among students at a Hispanic Serving Institution: development and evaluation design | BMC Public Health | Not related |
| 88 | Nagar, R.;Quirk, H. D.;Anderson, P. L.; | 2023 | User Experiences of college students using mental health applications to improve self-care: Implications for improving engagement | Internet Interv | Not related |
| 89 | Pope, Z. C.;Barr-Anderson, D. J.;Lewis, B. A.;Pereira, M. A.;Gao, Z.; | 2019 | Use of Wearable Technology and Social Media to Improve Physical Activity and Dietary Behaviors among College Students: a 12-Week Randomized Pilot Study | International journal of environmental research and public health | Not related |
| 90 | Cerretani, P. I.;Bernaras, E.;Garay, P. B.; | 2016 | Use of information and communications technology, academic performance and psychosocial distress in university students | Computers in Human Behavior | Mental health disorders |
| 91 | Alberth, | 2019 | Use of Facebook, students' intrinsic motivation to study writing, writing self-efficacy, and writing | Technology Pedagogy and | Not related |

| | | | | | |
|---|---|---|---|---|---|
| | | | performance | Education | |
| 92 | Hübner, I. B.;Scheibe, D. B.;Marchezan, J.;Bücker, J.; | 2024 | Use of Citicoline in Attention-Deficit/Hyperactivity Disorder: A Pilot Study | Clinical Neuropharmacology | Mental health disorders |
| 93 | Freichel, R.;Herzog, P.;Billings, J.;Bloomfield, M. A. P.;McNally, R. J.;Greene, T.; | 2024 | Unveiling temporal dynamics of PTSD and its functional impairments: A longitudinal study in UK healthcare workers | Journal of Anxiety Disorders | Mental health disorders |
| 94 | Stephens, N. M.;Fryberg, S. A.;Markus, H. R.;Johnson, C. S.;Covarrubias, R.; | 2012 | Unseen disadvantage: how American universities' focus on independence undermines the academic performance of first-generation college students | J Pers Soc Psychol | Not related |
| 95 | Ramakrishnan, C.;Widjaja, N.;Malhotra, C.;Finkelstein, E.;Khan, B. A.;Ozdemir, S.; | 2024 | Unraveling complex choices: multi-stakeholder perceptions on dialysis withdrawal and end-of-life care in kidney disease | BMC Nephrology | Not related |
| 96 | Rankin, James A.;Paisley, Courtney A.;Mulla, Mazheruddin M.;Tomeny, Theodore S.; | 2018 | Unmet social support needs among college students: Relations between social support discrepancy and depressive and anxiety symptoms | Journal of Counseling Psychology | Mental health disorders |
| 97 | Cerel, J.;Fruhbauerova, M.;Edwards, A.;Murphy, L.;Salt, E.;Whipple, B.;Clark, P. M.;Ackerman, J.; | 2024 | Universal Safety Planning for Suicide Prevention: CODE RED Initial Feasibility and Acceptability | International journal of environmental research and public health | Mental health disorders |
| 98 | Zhao, Y.;Tran, A.;Mattie, H.; | 2024 | Unhealthy weight control behaviors and health risk behaviors in American youth: a repeated cross-sectional study | Journal of Eating Disorders | Not related |
| 99 | Kim, T.;Hong, H.; | 2021 | Understanding University Students' Experiences, Perceptions, and Attitudes Toward Peers | JMIR Ment Health | Not related |

| | | | | | |
|---|---|---|---|---|---|
| | | | Displaying Mental Health-Related Problems on Social Networking Sites: Online Survey and Interview Study | | |
| 100 | Vornholt, P.;De Choudhury, M.; | 2021 | Understanding the Role of Social Media-Based Mental Health Support Among College Students: Survey and Semistructured Interviews | JMIR Ment Health | Not related |
| 101 | Cerezo, A.;Ramirez, A.;O'Shaughnessy, T.;Sanchez, A.;Mattis, S.;Ross, A.; | 2021 | Understanding the Power of Social Media during COVID-19: Forming Social Norms for Drinking among Sexual Minority Gender Expansive College Women | J Homosex | Not related |
| 102 | Pang, Hua; | 2018 | Understanding the effects of WeChat on perceived social capital and psychological well-being among Chinese international college students in Germany | Aslib Journal of Information Management | Not related |
| 103 | Frazier, Patricia B. S.;Gabriel, Abigail PhD;Merians, Addie B. S.;Lust, Katherine PhD; | 2019 | Understanding stress as an impediment to academic performance | Journal of American College Health | Mental health disorders |
| 104 | Bouchard, Elizabeth G.;Prince, Mark A.;McCarty, Christopher;Vincent, Paula C.;Patel, Hital;LaValley, Susan A.;Collins, R. Lorraine;Sahler, Olle Jane Z.;Krenz, Till;Kelly, Kara M.; | 2023 | Understanding social network support, composition, and structure among cancer caregivers | Psycho-Oncology | Not related |
| 105 | Xin, Y.;Ren, T.;Chen, X.;Liu, X.;Wu, Y.;Jing, S.;Zhang, L.;Dai, Z.;Wang, Y.;Su, X.; | 2024 | Understanding psychological symptoms among Chinese college students during the COVID-19 Omicron pandemic: Findings from a national cross-sectional survey in 2023 | Comprehensive Psychoneuroendocrinology | Not related |
| 106 | Chiu, C. M.;Huang, H. Y.;Cheng, H. | 2015 | Understanding online community citizenship | International | Not related |

| | | | | | |
|---|---|---|---|---|---|
| | L.;Sun, P. C.; | | behaviors through social support and social identity | Journal of Information Management | |
| 107 | Van Eekert, N.;De Bruyn, S.;Wouters, E.;Van de Velde, S.; | 2023 | Understanding Mental Wellbeing amongst Potentially Vulnerable Higher Education Students during the COVID-19 Pandemic | Social Sciences-Basel | Mental health disorders |
| 108 | Borghouts, J.;Eikey, E. V.;Mark, G.;De Leon, C.;Schueller, S. M.;Schneider, M.;Stadnick, N.;Zheng, K.;Mukamel, D. B.;Sorkin, D. H.; | 2021 | Understanding mental health app use among community college students: web-based survey study | Journal of medical Internet research | Not related |
| 109 | Vela, J. C.;Smith, W. D.;Guerra, F.;Hinojosa, K.;Aquila, J. D.;Ortega, K.; | 2018 | Understanding Humanistic and Family Predictors of Mexican American College Students' Subjective Happiness | Journal of Humanistic Counseling | Not related |
| 110 | Pardavila-Belio, M. I.;Canga-Armayor, A.;Duaso, M. J.;Pueyo-Garrigues, S.;Pueyo-Garrigues, M.;Canga-Armayor, N.; | 2019 | Understanding how a smoking cessation intervention changes beliefs, self-efficacy, and intention to quit: a secondary analysis of a pragmatic randomized controlled trial | Translational Behavioral Medicine | Mental health disorders |
| 111 | Wilson, V. E.;Le Brocque, R.;Drayton, J.;Hammer, S.; | 2024 | Understanding and responsiveness in the trauma-informed adult ESL classroom | Australian Educational Researcher | Not related |
| 112 | Hong, Y.;Gardner, L.; | 2019 | Undergraduates' perception and engagement in Facebook learning groups | British Journal of Educational Technology | Not related |
| 113 | Kaljo, K.;Braun, M. T.;Maddula, | 2023 | Undergraduate College Pathway Programs | South Med J | Not related |

| | | | | | |
|---|---|---|---|---|---|
| | R.;Ferguson, C. C.;Bonifacino, E.;Farkas, A.; | | Designed to Attract and Matriculate Students from Underrepresented Groups into Medicine | | |
| 114 | Lewandowski, G. W.;Mattingly, B. A.;Pedreiro, A.; | 2014 | Under pressure: the effects of stress on positive and negative relationship behaviors | Journal of Social Psychology | Mental health disorders |
| 115 | Raynal, P.;Goutaudier, N.;Nidetch, V.;Chabrol, H.; | 2016 | Typology of schizotypy in non-clinical young adults: Psychopathological and personality disorder traits correlates | Psychiatry Res | The population is not college students |
| 116 | Hombrados-Mendieta, Ma Isabel;Gomez-Jacinto, Luis;Dominguez-Fuentes, Juan Manuel;Garcia-Leiva, Patricia;Castro-Travé, Margarita; | 2012 | TYPES OF SOCIAL SUPPORT PROVIDED BY PARENTS, TEACHERS, AND CLASSMATES DURING ADOLESCENCE | Journal of Community Psychology | Not related |
| 117 | Duffy, M. E.;Twenge, J. M.;Joiner, T. E.; | 2019 | Trends in Mood and Anxiety Symptoms and Suicide-Related Outcomes Among U.S. Undergraduates, 2007-2018: Evidence from Two National Surveys | Journal of Adolescent Health | Mental health disorders |
| 118 | Fleming, A. R.;Edwin, M.;Hayes, J. A.;Locke, B. D.;Lockard, A. J.; | 2018 | Treatment-seeking college students with disabilities: Presenting concerns, protective factors, and academic distress | Rehabil Psychol | Mental health disorders |
| 119 | Boykin, D. M.;Orcutt, H. K.; | 2019 | Treatment Use Among College Women Following a Campus Shooting | J Aggress Maltreat Trauma | Not related |
| 120 | Horstmann, R. H.;Seefeld, L.;Schellong, J.;Garthus-Niegel, S.; | 2024 | Treatment and counseling preferences of postpartum women with and without symptoms of (childbirth-related) PTSD: findings of the cross-sectional study INVITE | BMC Pregnancy and Childbirth | Mental health disorders |
| 121 | Babakhanyan, I.;Sedigh, | 2024 | Traumatic brain injury rehabilitation for | NeuroRehabilitation | Mental health disorders |

| | | | | | |
|---|---|---|---|---|---|
| | R.;Remigio-Baker, R.;Hungerford, L.;Bailie, J. M.; | | warfighters with post-traumatic stress | | |
| 122 | Zeligman, M.;Varney, M.;Gheesling, S.;Placeres, V.; | 2019 | Trauma, Meaning-Making, and Loneliness in College Students | Journal of College Student Psychotherapy | Mental health disorders |
| 123 | Oliveira, Jscd;Freitas, L.;Tomlinson, G. M.;Petursdottir, A. I.; | 2021 | Translational evaluation of training structures in equivalence-based instruction | Journal of the experimental analysis of behavior | Not related |
| 124 | Li, L.;Peng, W.; | 2019 | Transitioning through social media: International students' SNS use, perceived social support, and acculturative stress | Computers in Human Behavior | Mental health disorders |
| 125 | Martins, T. H. S.;Rodrigues, R. M.;Araujo, F. C. O.;Cedro, A. M.;Bortoloti, R.;Varella, A. A. B.;Huziwara, E. M.; | 2023 | Transfer of functions based on equivalence class formation using musical stimuli | Journal of the experimental analysis of behavior | Not related |
| 126 | Nochaiwong, S.;Ruengorn, C.;Awiphan, R.;Phosuya, C.;Ruanta, Y.;Kanjanarat, P.;Wongpakaran, N.;Wongpakaran, T.;Thavorn, K.; | 2022 | Transcultural adaptation and psychometric validation of the Thai-Brief Resilient Coping Scale: a cross-sectional study during the coronavirus disease 2019 pandemic in Thailand | Sci Rep | Not related |
| 127 | Nejati, V.;Dehghan, M.;Shahidi, S.;Estaji, R.;Nitsche, M. A.; | 2024 | Transcranial random noise stimulation (tRNS) improves hot and cold executive functions in children with attention deficit hyperactivity disorder (ADHD) | Scientific Reports | Mental health disorders |
| 128 | Moghadas Tabrizi, Y.;Yavari, M.;Shahrbanian, S.;Gharayagh Zandi, H.; | 2019 | Transcranial direct current stimulation on prefrontal and parietal areas enhances motor imagery. | Neuroreport | Not related |

| | | | | | |
|---|---|---|---|---|---|
| 129 | Liu, X.;Li, J.;Wang, G.;Yin, X.;Li, S.;Fu, X.; | 2020 | Transcranial direct current stimulation of the rLPFC shifts normative judgments in voluntary cooperation. | Neuroscience letters | Not related |
| 130 | Estaji, R.;Hosseinzadeh, M.;Arabgol, F.;Nejati, V.; | 2024 | Transcranial direct current stimulation (tDCS) improves emotion regulation in children with attention-deficit hyperactivity disorder (ADHD) | Scientific Reports | Mental health disorders |
| 131 | Rogers, A. A.;Updegraff, K. A.;Iida, M.;Dishion, T. J.;Doane, L. D.;Corbin, W. C.;Van Lenten, S. A.;Ha, T.; | 2018 | Trajectories of positive and negative affect across the transition to college: The role of daily interactions with parents and friends | Dev Psychol | Not related |
| 132 | Wang, Z.;Xu, Y.;Zeng, H.; | 2023 | Trait rumination in post-stress growth among Chinese college students: the chain mediating effect of distress disclosure and perceived social support | Front Public Health | Mental health disorders |
| 133 | Krysinska, K.;Lester, D.;Lyke, J.;Corveleyn, J.; | 2015 | Trait gratitude and suicidal ideation and behavior: an exploratory study | Crisis | Mental health disorders |
| 134 | Mofidi, T.;El-Alayli, A.;Brown, A.; | 2014 | Trait gratitude and grateful coping as they relate to college student persistence, success, and integration in school | Journal of College Student Retention: Research, Theory and Practice | Not related |
| 135 | Świątek, A. H.;Szcześniak, M.;Bielecka, G.; | 2021 | Trait Anxiety and Social Media Fatigue: Fear of Missing Out as a Mediator | Psychol Res Behav Manag | Mental health disorders |
| 136 | Mihailidis, P.;Fincham, K.;Cohen, J. N.; | 2014 | Toward a Media Literate Model for Civic Engagement in Digital Culture: Exploring the Civic Habits and Dispositions of College Students on Facebook | Atlantic Journal of Communication | Not related |

| | | | | | |
|---|---|---|---|---|---|
| 137 | Junco, R.; | 2012 | Too much face and not enough books: The relationship between multiple indices of Facebook use and academic performance | Computers in Human Behavior | Not related |
| 138 | Berg, C. J.;Haardörfer, R.;Lanier, A.;Childs, D.;Foster, B.;Getachew, B.;Windle, M.; | 2020 | Tobacco Use Trajectories in Young Adults: Analyses of Predictors Across Systems Levels | Nicotine Tob Res | The population is not college students |
| 139 | Tran, V.;Szabó, A.;Ward, C.;Jose, P. E.; | 2023 | To vent or not to vent? The impact of venting on psychological symptoms varies by levels of social support. | International Journal of Intercultural Relations | Not related |
| 140 | Krauss, B. J.; | 2024 | To Calm and to Commend: Veterans' Musical Preferences Anticipating End of Life | Military Medicine | Not related |
| 141 | Manrique, M.;Allwood, M. A.;Pugach, C. P.;Amoh, N.;Cerbone, A.; | 2020 | Time and support do not heal all wounds: Mental health correlates of past bullying among college students. | J Am Coll Health | Not related |
| 142 | Seo, J. W.; | 2020 | Thwarted belongingness and perceived burdensomeness in Korean college students: Psychometric properties and cultural considerations for the interpersonal needs Questionnaire-10 | Death Stud | Not related |
| 143 | Wurtz, H. M.;Hernandez, M.;Baird, M.; | 2023 | Thriving Despite the Odds: Digital Capital and Reimagined Life Projects Among Mexican College Students During COVID-19 | Cult Med Psychiatry | Not related |
| 144 | Beerse, M. E.;Van Lith, T.;Stanwood, G.; | 2020 | Therapeutic psychological and biological responses to mindfulness-based art therapy | Stress and Health | Not related |
| 145 | Berzosa-Gonzalez, | 2024 | Therapeutic Approach to Primary Tic Disorders | Brain Sciences | Mental health disorders |

| | | | | | |
|---|---|---|---|---|---|
| | I.;Martinez-Horta, S.;Pérez-Pérez, J.;Kulisevsky, J.;Pagonabarraga, J.; | | and Associated Psychiatric Comorbidities | | |
| 146 | Savage, M. W.;Deiss, D. M.;Roberto, A. J.;Aboujaoude, E.; | 2017 | Theory-Based Formative Research on an Anti-Cyberbullying Victimization Intervention Message | Journal of Health Communication | Mental health disorders |
| 147 | Lundqvist, J.;Lindberg, M. S.;Brattmyr, M.;Havnen, A.;Hjemdal, O.;Solem, S.; | 2024 | The Work and Social Adjustment Scale (WSAS): An investigation of reliability, validity, and associations with clinical characteristics in psychiatric outpatients | PLoS ONE | Mental health disorders |
| 148 | Smith, R. A.;Brown, M. G.;Schiltz, J. J.;Sowl, S.;Schulz, J. M.;Grady, K. A.; | 2023 | The Value of Interpersonal Network Continuity for College Students in Disruptive Times | Innov High Educ | Not related |
| 149 | Chen, I. C.;Chang, C. L.;Chang, M. H.;Ko, L. W.; | 2024 | The utility of wearable electroencephalography combined with behavioral measures to establish a practical multi-domain model for facilitating the diagnosis of young children with attention-deficit/hyperactivity disorder. | Journal of Neurodevelopmental Disorders | The population is not college students. |
| 150 | You, J. S.;Chung, S. P.;Park, J. Y.;Park, S.;Chung, T. N.;Park, I.;Kim, J. H.;Park, J. W.;Hwang, T. S.; | 2012 | The utility of the HeartSaver Sticker for maintaining correct hand position during chest compressions | Journal of Emergency Medicine | Not related |
| 151 | Apgar, D.; | 2020 | The use of group text messaging to enhance social support of social work students | Social Work Education | Not related |
| 152 | Joseph, J.;Vinay, M.; | 2024 | The use of augmented reality in assessing and training children with attention deficit hyperactivity disorder | IAES International Journal of Artificial Intelligence | The population is not college students |

| | | | | | |
|---|---|---|---|---|---|
| 153 | Wimberley, D. W.;Raonka, P.;Rose, T.;Sabirova, S.;Gheesling, S.; | 2024 | The US Student Antisweatshop Movement's Presence and Success at the Campus Level: Impacts of Collective Identity Strength and Network Density1 | Sociological Inquiry | Not related |
| 154 | Wu, Z. M.;Wang, P.;Zhong, Y. Y.;Liu, Y.;Liu, X. C.;Wang, J. J.;Cao, X. L.;Liu, L.;Sun, L.;Yang, L.;Zang, Y. F.;Qian, Y.;Cao, Q. J.;Wang, Y. F.;Yang, B. R.; | 2024 | The underlying neuropsychological and neural correlates of the impaired Chinese reading skills in children with attention deficit hyperactivity disorder | European Child and Adolescent Psychiatry | The population is not college students |
| 155 | Park, C. S.;Kaye, B. K.; | 2017 | The tweet goes on: Interconnection of Twitter opinion leadership, network size, and civic engagement. | Computers in Human Behavior | Not related |
| 156 | Mandali, H.;Ghorbani, M.;Molamehdizadeh, N.;Abolghasemi, J.;Ebrahimi, H.; | 2024 | The synergistic effect of attention-deficit hyperactivity disorder (ADHD) and technology-based inattention on reduction of driving performance | Transportation Research Part F: Traffic Psychology and Behaviour | Mental health disorders |
| 157 | Liu, J.; | 2014 | The study of the relationship between students' perception of teacher's function and students' English learning achievement based on a multimedia application environment | BioTechnology: An Indian Journal | Not related |
| 158 | Zhang, R. W.; | 2017 | The stress-buffering effect of self-disclosure on Facebook: An examination of stressful life events, social support, and mental health among college students | Computers in Human Behavior | Mental health disorders |
| 159 | Chen, Z. W.;Liu, Y.; | 2020 | The state of leisure life situation and the meaning of leisure education for college students in China | International Journal of | Not related |

| | | | | | |
|---|---|---|---|---|---|
| | | | | Educational Research | |
| 160 | Terrana, A.;Bruno, W.;Ibrahim, N.;Kaiser, B. N.;Wei, J.;Al-Delaimy, W.; | 2024 | The Somali Distress and Resilience Scale: Development of a novel measure for Somali adults | Transcultural Psychiatry | The population is not college students |
| 161 | Zanini, D. S.;Peixoto, E. M.;Nakano, T. D. C.; | 2018 | The Social Support Scale (MOS-SSS): Standardizing with item references | Trends in Psychology | Not related |
| 162 | Qu, Yan;Saffer, Adam J.;Riffe, Daniel; | 2022 | The Social network antecedents to consumer engagement: revealing how consumers' conversations Influence Online Engagement behaviors | Corporate Communications | Not related |
| 163 | Bellet, Benjamin W.;Holland, Jason M.;Neimeyer, Robert A.; | 2019 | The Social Meaning in Life Events Scale (SMILES): A preliminary psychometric evaluation in a bereaved sample | Death Studies | Not related |
| 164 | Medina, Maria Sheila G.; | 2018 | The Self-esteem, Social Support and College Adjustment of Business and Accountancy Students | Review of Integrative Business and Economics Research, suppl. Supplementary Issue 2 | Not related |
| 165 | Wittrup, Audrey R.;Hurd, Noelle M.; | 2022 | The Role of Trajectories of Stress and Social Support in Underrepresented Students' Educational Outcomes | Applied Developmental Science | Mental health disorders |
| 166 | Perera, H. N.;DiGiacomo, M.; | 2015 | The role of trait emotional intelligence in academic performance during the University transition: An integrative model of mediation via social support, | Personality and Individual Differences | Not related |

| | | | | | |
|---|---|---|---|---|---|
| | | | coping, and adjustment | | |
| 167 | Stallman, H. M.;Ohan, J. L.;Chiera, B.; | 2018 | The Role of Social Support, Being Present, and Self-kindness in University Student Psychological Distress | Australian Psychologist | Mental health disorders |
| 168 | Kahn, Jeffrey H.;Cantwell, Kaitlyn E.; | 2017 | The role of social support in the disclosure of every day unpleasant emotional events | Counselling Psychology Quarterly | Not related |
| 169 | Mo, P. K. H.;Chan, V. W. Y.;Chan, S. W.;Lau, J. T. F.; | 2018 | The role of social support on emotion dysregulation and Internet addiction among Chinese adolescents: A structural equation model | Addictive behaviors | Not related |
| 170 | Motsabi, S.;Diale, B. M.;Van Zyl, A.; | 2020 | THE ROLE OF SOCIAL SUPPORT IN THE PERSISTENCE OF FIRST-YEAR FIRST-GENERATION AFRICAN STUDENTS IN A HIGHER EDUCATION INSTITUTION IN SOUTH AFRICA | South African Journal of Higher Education | Not related |
| 171 | Rice, Lindsay;Barth, Joan M.;Guadagno, Rosanna E.;Smith, Gabrielle P.;McCallum, Debra M.; | 2013 | The Role of Social Support in Students' Perceived Abilities and Attitudes Toward Math and Science | Journal of Youth and Adolescence | Not related |
| 172 | Agbaria, Q.;Bdier, D.; | 2021 | The Role of Social Support and Subjective Well-Being as Predictors of Internet Addiction among Israeli-Palestinian College Students in Israel | International Journal of Mental Health and Addiction | Not related |
| 173 | Zhao, J.;Kong, F.;Wang, Y.; | 2013 | The role of social support and self-esteem in the relationship between shyness and loneliness | Personality and Individual Differences | Not related |

| | | | | | |
|---|---|---|---|---|---|
| 174 | Cho, H.;Hussain, R. S. B.;Kang, H. K.; | 2023 | The role of social support and its influence on exercise Participation: The perspective of self-determination theory and the theory of planned behavior | Social Science Journal | Not related |
| 175 | Miao, C.;Zhou, C.;Sun, H.;Xu, J.;Zheng, J.;Huang, X.;Xia, Y.; | 2020 | The Role of Social Support and Environment: The Mediating Effect of College Students' Psychology and Behavior | Environment and Social Psychology | Not related |
| 176 | Kiema-Junes, H.;Hintsanen, M.;Soini, H.;Pyhältö, K.; | 2020 | The role of social skills in burnout and engagement among university students | Electronic Journal of Research in Educational Psychology | Not related |
| 177 | Felton, J. W.;Triemstra, J. D.;Reynolds, E. K.;Hale, N.;Magidson, J. F.;Lejuez, C. W.; | 2022 | The Role of Social Adjustment in a Collegiate Behavioral Activation Program | Behav Modif | Not related |
| 178 | Maples, A. E.;Williams-Wengerd, A.;Braughton, J. E.;Henry, K. L.;Haddock, S. A.;Weiler, L. M.; | 2022 | The role of service-learning experiences in promoting flourishing among college-student youth mentors | Journal of Positive Psychology | Not related |
| 179 | Lavin, K.;Goeke-Morey, M. C.;Degnan, K. A.; | 2020 | The role of self- compassion in college students' perceived social support | Journal of Positive Psychology and Wellbeing | Not related |
| 180 | Park, BoKyung;Kim, Minjae;Young, Liane; | 2022 | The role of relational mobility in relationship quality and well-being | Journal of Social and Personal Relationships | Not related |
| 181 | Campbell, R.;Riggs, S. A.; | 2015 | The Role of Psychological Symptomatology and Social Support in the Academic Adjustment of Previously Deployed Student Veterans | Journal of American College Health | Not related |

| | | | | | |
|---|---|---|---|---|---|
| 182 | Atay, E.;Ermiş, Ç;Gökbayrak Atay, İ N.;Aydemir, Ö;Özmen, E.; | 2024 | The role of predominant polarity on cognitive dysfunctions in patients with bipolar disorder | International Journal of Bipolar Disorders | Mental health disorders |
| 183 | Garro, M.;Novara, C.;Di Napoli, G.;Scandurra, C.;Bochicchio, V.;Lavanco, G.; | 2022 | The Role of Internalized Transphobia, Loneliness, and Social Support in the Psychological Well-Being of a Group of Italian Transgender and Gender Non-Conforming Youths | Healthcare | Not related |
| 184 | McCallen, Leigh S.;Johnson, Helen L.; | 2020 | The Role of Institutional Agents in Promoting Higher Education Success among First-Generation College Students at a Public Urban University | Journal of Diversity in Higher Education | Not related |
| 185 | Wong, W. L.;Cheung, S. H.; | 2024 | The role of hope in College transition: Its cross-lagged relationships with psychosocial resources and emotional well-being in first-year college students | J Adolesc | Not related |
| 186 | Roksa, Josipa;Kinsley, Peter; | 2019 | The Role of Family Support in Facilitating Academic Success of Low-Income Students | Research in Higher Education | Not related |
| 187 | Huang, C. Q.;Zhang, L. J.;He, T.;Wu, X. M.;Pan, Y. F.;Han, Z. M.;Zhao, W. Z.; | 2023 | The role of emotion regulation in predicting emotional engagement mediated by meta-emotion in online learning environments: a two-stage SEM-ANN approach | Educational Psychology | Meta review |
| 188 | Agoes Salim, R. M.;Istiasih, M. R.;Rumalutur, N. A.;Biondi Situmorang, D. D.; | 2023 | The role of career decision self-efficacy as a mediator of peer support on students' career adaptability | Heliyon | Not related |
| 189 | Katsiaficas, D.;Volpe, V.;Raza, S. S.;Garcia, Y.; | 2019 | The Role of Campus Support, Undocumented Identity, and Deferred Action for Childhood Arrivals on Civic Engagement for Latinx | Child Dev | The population is not college students |

| | | | | | |
|---|---|---|---|---|---|
| | | | Undocumented Undergraduates | | |
| 190 | Dattilo, T. M.;Roberts, C. M.;Fisher, R. S.;Traino, K. A.;Edwards, C. S.;Pepper-Davis, M.;Chaney, J. M.;Mullins, L. L.; | 2021 | The Role of Avoidance Coping and Illness Uncertainty in the Relationship Between Transition Readiness and Health Anxiety | J Pediatr Nurs | Mental health disorders |
| 191 | Kunst, L. E.;Bekker, M. H. J.;Maas, J.;van Assen, Malm;Duijndam, S.;Riem, M. M. E.; | 2021 | The role of autonomy-connectedness in stress-modulating effects of social support in women: An experimental study using a virtual Trier Social Stress Test | International journal of psychophysiology | Mental health disorders |
| 192 | Makaruk, H.;Porter, J. M.;Czaplicki, A.;Sadowski, J.;Sacewicz, T.; | 2012 | The role of attentional focus in plyometric training | Journal of sports medicine and physical fitness | Not related |
| 193 | Suryaratri, Ratna Dyah;Komalasari, Gantina;Medellu, Gita Irianda; | 2022 | The Role of Academic Self-Efficacy and Social Support in Achieving Academic Flow in Online Learning | International Journal of Technology in Education and Science | Not related |
| 194 | Wibrowski, Connie R.;Matthews, Wendy K.;Kitsantas, Anastasia; | 2017 | The Role of a Skills Learning Support Program on First-Generation College Students' Self-Regulation, Motivation, and Academic Achievement: A Longitudinal Study | Journal of College Student Retention: Research, Theory & Practice | Not related |
| 195 | Stoppelbein, L.;McRae, E.;Smith, S.; | 2024 | The ripple effect of trauma: Evaluating vulnerability, post-traumatic stress symptoms, and aggression within a child and adolescent population | Child Abuse and Neglect | The population is not college students |

| | | | | | |
|---|---|---|---|---|---|
| 196 | Mahler, H. I. M.; | 2018 | The relative role of cognitive and emotional reactions in mediating the effects of a social comparison sun protection intervention | Psychology & health | Mental health disorders |
| 197 | Kim, B.;Rho, M.; | 2023 | The Relationships of SNS Use, Social Achievement Goals, and Life Satisfaction: A Study of Korean College Students | Journal of Higher Education Theory and Practice | Not related |
| 198 | Park, I. J.;Kim, M.;Kwon, S.;Lee, H. G.; | 2018 | The Relationships of Self-Esteem, Future Time Perspective, Positive Affect, Social Support, and Career Decision: A Longitudinal Multilevel Study | Frontiers in Psychology | Not related |
| 199 | Mahmoud, J. S.;Staten, R. T.;Lennie, T. A.;Hall, L. A.; | 2015 | The relationships of coping, negative thinking, life satisfaction, social support, and selected demographics with the anxiety of young adult college students | J Child Adolesc Psychiatr Nurs | Mental health disorders |
| 200 | Koç, T.;Turan, A. H.; | 2021 | The Relationships Among Social Media Intensity, Smartphone Addiction, and Subjective Wellbeing of Turkish College Students | Applied Research in Quality of Life | Mental health disorders |
| 201 | Chen, Y. X.;Li, R. X.;Liu, X. P.; | 2021 | The relationships among relatedness frustration, affiliation motivation, and WeChat engagement, moderated by relatedness satisfaction | Cyberpsychology-Journal of Psychosocial Research on Cyberspace | Not related |
| 202 | Kong, F.;Ding, K.;Zhao, J. J.; | 2015 | The Relationships Among Gratitude, Self-esteem, Social Support, and Life Satisfaction Among Undergraduate Students | Journal of Happiness Studies | Not related |
| 203 | Wu, A.; | 2024 | The relationship of social support to posttraumatic growth in COVID-19 among college students after | Front Psychiatry | Not related |

| | | | | | |
|---|---|---|---|---|---|
| | | | experiencing campus lockdown: the effects of belief in a just world and meaning in life | | |
| 204 | Brodar, K. E.;Crosskey, L. B.;Thompson, R. J.; | 2015 | The Relationship of Self-Compassion with Perfectionistic Self-Presentation, Perceived Forgiveness, and Perceived Social Support in an Undergraduate Christian Community | Journal of Psychology and Theology | Not related |
| 205 | Kujala, J.;Matveinen, S.;van Bijnen, S.;Parviainen, T.; | 2024 | The relationship between structural properties of frontal cortical regions and response inhibition in 6–14-year-old children | Brain and Cognition | The population is not college students |
| 206 | Chen, D.;Miao, X.;Ma, Y.;Tang, Y.; | 2022 | The relationship between social support and goal pursuit among Chinese college students: The mediating role of just-world beliefs | Front Psychol | Not related |
| 207 | Tian, Y.;Shi, Z.; | 2022 | The Relationship between Social Support and Exercise Adherence among Chinese College Students during the COVID-19 Pandemic: The Mediating Effects of Subjective Exercise Experience and Commitment | Int J Environ Res Public Health | Not related |
| 208 | Dong, Yuanyuan;Li, Hongyu; | 2020 | The relationship between social support and depressive symptoms among the college students of Liaoning, China: a moderated mediated analysis | Psychology, health & medicine | Mental health disorders |
| 209 | Li, Tiantian;Tien, Hsiu-Lan Shelley;Gu, Jiyou;Wang, Juanjuan; | 2023 | The relationship between social support and career adaptability: the chain mediating role of perceived career barriers and career maturity | International Journal for Educational and Vocational Guidance | Not related |
| 210 | Chen, C. M.;Bian, F.;Zhu, Y. J.; | 2023 | The relationship between social support and | BMC Public Health | Not related |

| | | | | | |
|---|---|---|---|---|---|
| | | | academic engagement among university students: the chain mediating effects of life satisfaction and academic motivation | | |
| 211 | Azizi, S. M.;Soroush, A.;Khatony, A.; | 2019 | The relationship between social networking addiction and academic performance in Iranian students of medical sciences: a cross-sectional study | BMC Psychology | Not related |
| 212 | Sabbah, Wael;Tsakos, Georgios;Chandola, Tarani;Newton, Tim;Kawachi, Ichiro;Sheiham, Aubrey;Marmot, Michael G.;Watt, Richard G.; | 2011 | The relationship between social network, social support and periodontal disease among older Americans | Journal of Clinical Periodontology | Not related |
| 213 | Bailey, K. M.;Frost, K. M.;Casagrande, K.;Ingersoll, B.; | 2020 | The relationship between social experience and subjective well-being in autistic college students: A mixed methods study | Autism | Not related |
| 214 | Zheng, X. L.;Wang, Z. Y.;Chen, H. P.;Xie, F. W.; | 2021 | The relationship between self-esteem and Internet altruistic behavior: The mediating effect of online social support and its gender differences | Personality and Individual Differences | Not related |
| 215 | Ye, Yuan;Wu, Daili;Chen, Zhongnong;Chen, Daile;Zhou, Qiang;Jeong, Jaesik;Tu, Yanling; | 2024 | The relationship between self-consciousness and depression in college students: the chain mediating effect of meaning life and self-efficacy, with the moderating effect of social support | BMC Public Health | Mental health disorders |
| 216 | Liu, Y. H.; | 2024 | The Relationship Between Resilience, Interactive Distance, and College Students' Online Mathematics Learning Engagement: A Longitudinal Study | Psychology Research and Behavior Management | Not related |

| | | | | | |
|---|---|---|---|---|---|
| 217 | Liao, T.;Yin, Y.;Hu, X.;Tang, S.;Shim, Y.; | 2023 | The relationship between physical activity and subjective well-being in Chinese university students: the mediating roles of perceived health, social support and self-esteem | Front Sports Act Living | Not related |
| 218 | Hou, X. L.;Wang, H. Z.;Hu, T. Q.;Gentile, D. A.;Gaskin, J.;Wang, J. L.; | 2019 | The relationship between perceived stress and problematic social networking site use among Chinese college students | J Behav Addict | Mental health disorders |
| 219 | Zhao, G.;Xie, F.;Li, S.;Ding, Y.;Li, X.;Liu, H.; | 2022 | The relationship between perceived social support with anxiety, depression, and insomnia among Chinese college students during the COVID-19 pandemic: The mediating role of self-control | Front Psychiatry | Mental health disorders |
| 220 | Harrell, Z. A.;Powell, K.; | 2014 | The relationship between parent and student religious coping and college alcohol use | J Relig Health | Mental health disorders |
| 221 | Zhang, S. W.;Wu, Q.;Liu, R. F.; | 2023 | The relationship between neuroticism and passive use of mobile social networks among Chinese young adults: The mediating role of fear of missing out and online social support | Acta Psychologica | The population is not college students |
| 222 | Li, Guangming; | 2023 | The Relationship between Mobile Phone Dependence and Subjective Well-Being of College Students in China: A Moderated Mediation Model | Healthcare | Not related |
| 223 | Choi, Y.;Choi, S. H.;Yun, J. Y.;Lim, J. A.;Kwon, Y.;Lee, H. Y.;Jang, J. H.; | 2019 | The relationship between levels of self-esteem and the development of depression in young adults with mild depressive symptoms | Medicine (Baltimore) | The population is not college students |
| 224 | Luo, Q.;Huang, L.;Wu, N.; | 2022 | The relationship between Internet use preference and loneliness among college students during COVID-19: The chain mediating effect of online | Front Psychol | Not related |

| | | | | | |
|---|---|---|---|---|---|
| | | | social support and self-esteem | | |
| 225 | Yang, M.;Haydon, K. C.;Miller, M. J.; | 2013 | The relationship between intergenerational cultural conflict and social support among Asian American and Asian international female college students and their parents | Asian American Journal of Psychology | Not related |
| 226 | Junco, R.; | 2012 | The relationship between frequency of Facebook use, participation in Facebook activities, and student engagement | Computers and Education | Not related |
| 227 | Gao, H.;Ou, Y.;Zhang, Z.;Ni, M.;Zhou, X.;Liao, L.; | 2021 | The Relationship Between Family Support and e-Learning Engagement in College Students: The Mediating Role of e-Learning Normative Consciousness and Behaviors and Self-Efficacy | Front Psychol | Not related |
| 228 | Kalpidou, M.;Costin, D.;Morris, J.; | 2011 | The relationship between Facebook and the well-being of undergraduate college students | Cyberpsychol Behav Soc Netw | Not related |
| 229 | Haj-Yahia, Muhammad M.;Sokar, Shireen;Hassan-Abbas, Niveen;Malka, Menny; | 2019 | The relationship between exposure to family violence in childhood and post-traumatic stress symptoms in young adulthood: The mediating role of social support | Child Abuse & Neglect | The population is not college students |
| 230 | Brailovskaia, J.;Rohmann, E.;Bierhoff, H. W.;Schillack, H.;Margraf, J.; | 2019 | The relationship between daily stress, social support, and Facebook Addiction Disorder | Psychiatry Research | Mental health disorders |
| 231 | Wen, H.;Kong, X.;Feng, Y.; | 2022 | The relationship between cyber upward social comparison and cyberbullying behaviors: A moderated mediating model | Front Psychol | Not related |
| 232 | Gong, Z.;Lv, Y.;Jiao, X.;Liu, J.;Sun, Y.;Qu, Q.; | 2022 | The relationship between COVID-19-related restrictions and fear of missing out, problematic | Front Public Health | Not related |

| | | | | | |
|---|---|---|---|---|---|
| | | | smartphone use, and mental health in college students: The moderated moderation effect of resilience and social support | | |
| 233 | Wang, Peipei;Sun, Wenmei;Zhong, Qiqi; | 2023 | The relationship between college students' extraversion and entrepreneurial intention: The mediating role of perceived social support | Social Behavior and Personality | Not related |
| 234 | Xiang, Y. H.;Wang, W. X.;Guan, F.; | 2018 | The Relationship Between Child Maltreatment and Dispositional Envy and the Mediating Effect of Self-Esteem and Social Support in Young Adults | Frontiers in Psychology | The population is not college students |
| 235 | Munguia, A.;Ostrosky, F.;Lozano, A.;Castañeda, D.;Lujan, A.;Diaz, K.;Perez, M.;Lara, R.;Sacristan, E.; | 2024 | The relationship between changes in functional networks and cognitive changes and PTSD symptoms in maltreated children before and after TF-CBT | Behavioural Brain Research | Mental health disorders |
| 236 | Xia, T. S.;Gu, H. L.;Huang, Y. M.;Zhu, Q.;Cheng, Y. F.; | 2020 | The Relationship Between Career Social Support and Employability of College Students: A Moderated Mediation Model | Frontiers in Psychology | Not related |
| 237 | Wang, H.;Jiao, R. K.; | 2023 | The relationship between career social support and career management competency: The mediating role of career decision-making self-efficacy | Current Psychology | Not related |
| 238 | Li, N.;Yang, Y.;Zhao, X.;Li, Y.; | 2023 | The relationship between achievement motivation and college students’ general self-efficacy: A moderated mediation model | Frontiers in Psychology | Not related |
| 239 | Ye, J. H.;Zhang, M.;Yang, X.;Wang, M.; | 2023 | The Relation between Intergroup Contact and Subjective Well-Being among College Students at Minzu Universities: The Moderating Role of Social Support | Int J Environ Res Public Health | Not related |

| | | | | | |
|---|---|---|---|---|---|
| 240 | Jin-Liang, Wang;Gaskin, James;Rost, Detlef H.;Gentile, Douglas A.; | 2018 | The Reciprocal Relationship Between Passive Social Networking Site (SNS) Usage and Users' Subjective Well-Being | Social Science Computer Review | Not related |
| 241 | Akanni, O. O.;Edeh, A. N.;Agbir, M. T.;Olashore, A. A.; | 2024 | The Quality of Life and its inter-relationship with posttraumatic stress disorder and social support in two post-conflict communities in Nigeria | Journal of Health Psychology | Mental health disorders |
| 242 | Cao, W.;Fang, Z.;Hou, G.;Han, M.;Xu, X.;Dong, J.;Zheng, J.; | 2020 | The psychological impact of the COVID-19 epidemic on college students in China | Psychiatry Res | Not related |
| 243 | Ravan, J. R.;Panda, U. K.;Pattnaik, J. I.;Banerjee, S.;Chaudhuri, T.;Patil, S.;Das, R. C.; | 2024 | The psychological impact of disaster on first responders in the aftermath of Bahanaga train tragedy: A mixed methods approach | Indian Journal of Psychiatry | Not related |
| 244 | Marchetti, S.;Feinstein, A.; | 2024 | The Psychological Health of Iranian Citizens Protesting the Actions of Their Country's Morality Police: La santé psychologique des citoyens iraniens qui manifestent contre les actions de la police des mœurs de leur pays | Canadian Journal of Psychiatry | Not related |
| 245 | Stock, M. L.;Litt, D. M.;Arlt, V.;Peterson, L. M.;Sommerville, J.; | 2013 | The prototype/willingness model, academic versus health-risk information, and risk cognitions associated with nonmedical prescription stimulant use among college students | British journal of health psychology | Not related |
| 246 | Benson, O. M.;Whitson, M. L.; | 2022 | The protective role of a sense of community and access to resources on college student stress and COVID-19-related daily life disruptions | J Community Psychol | Mental health disorders |
| 247 | Gan, J.;Guo, Y.;Wang, E.; | 2024 | The processing mechanism of mixed prospective memory: changes in internal and external attention | Quarterly journal of experimental psychology (2006) | Not related |

| | | | | | |
|---|---|---|---|---|---|
| 248 | Abuhamdah, S. M. A.;Naser, A. Y.;Abdelwahab, G. M.;Alqatawneh, A.; | 2021 | The prevalence of mental distress and social support among university students in Jordan: A cross-sectional study | International journal of environmental research and public health | Mental health disorders |
| 249 | Uysal, R.; | 2015 | The predictive roles of social safeness and flourishing on problematic Facebook use | South African Journal of Psychology | Not related |
| 250 | Sun, M.;Wang, D.;Jing, L.;Zhou, L.; | 2023 | The predictive role of psychotic-like experiences in suicidal ideation among technical secondary school and college students during the COVID-19 pandemic | BMC Psychiatry | Mental health disorders |
| 251 | Wang, S. Z.;Li, J.;Li, Y. B.;Xia, Y. W.;Gong, Y.;Mao, F. Q.; | 2023 | The predictive role of impulsivity and perceived social support in psychiatric symptoms of women with methamphetamine use disorder | Frontiers in Psychiatry | Mental health disorders |
| 252 | Zhu, X. Q.;Chu, C. K. M.;Lam, Y. C.; | 2022 | The Predictive Effects of Family and Individual Wellbeing on University Students' Online Learning During the COVID-19 Pandemic | Frontiers in Psychology | Not related to research questions |
| 253 | Andrieieva, O.;Byshevets, N.;Kashuba, V.;Loshytska, T.;Golovanova, N.; | 2024 | The potential of adventure tourism as a means of preventing stress-related states in students during wartime | Fizicna Reabilitacia ta Rekreacijno-Ozdorovci Tehnologii | Mental health disorders |
| 254 | Langdon, R. R.;Bradley, L.;Newton, C. J.;Sawang, S.; | 2024 | The Potential for Workplaces to Provide Social Support for Distressed Infrastructure Workers | Journal of Construction Engineering and Management | Mental health disorders |

| | | | | | |
|---|---|---|---|---|---|
| 255 | Baker, C.;Kirby, J. B.;O'Connor, J.;Lindsay, K. G.;Hutchins, A.;Harris, M.; | 2022 | The Perceived Impact of Ashwagandha on Stress, Sleep Quality, Energy, and Mental Clarity for College Students: qualitative Analysis of a Double-Blind Randomized Control Trial | Journal of Medicinal Food | Mental health disorders |
| 256 | Fort, I.;Murariu, A.; | 2018 | The Paths Between Gender, Barriers, Social Support, Coping Efficacy, and Educational Goals | Journal of Career Assessment | Not related |
| 257 | Fort, I.;Murariu, A.; | 2018 | The paths between gender, barriers, social support, coping efficacy, and vocational indecision | International Journal for Educational and Vocational Guidance | Not related |
| 258 | Chrysomalidou, A.;Takos, I.;Spiliotis, I.;Xofis, P.; | 2024 | The Participation of Teachers in Greece in Outdoor Education Activities and the Schools' Perceptions of the Benefits to Students | Education Sciences | Not related |
| 259 | Schmiedehaus, E.;Snyder, E.;Perrotte, J.;Deason, R.;Howard, K.;Cordaro, M.; | 2023 | The Ongoing Mental Health Plight of Depressed College Students: Clinical Recommendations and the Importance of Early Screening and Detection | Issues Ment Health Nurs | Mental health disorders |
| 260 | Szuhany, K. L.;Otto, M. W.; | 2020 | The new TV Dinner: effects of television programming content on eating and attitudes towards exercise | Psychology, health & medicine | Not related |
| 261 | Ray, C. D.;Mikkelson, A. C.; | 2023 | The multidimensional evaluation of enacted social support: Best practices for use based on issues of factor structure, study design, and scale instructions | Journal of Social and Personal Relationships | Not related |
| 262 | Siewert, K.;Antoniw, K.;Kubiak, T.;Weber, H.; | 2011 | The more the better. The relationship between mismatches in social support and subjective | Journal of Health Psychology | Not related |

| | | | | | |
|---|---|---|---|---|---|
| | | | well-being in daily life | | |
| 263 | Li, Xu;Wang, Yu-Wei;Kim, Young Hwa; | 2022 | The Moderation of Parental Support on the Relationship between Race-Related Career Barriers and Academic Achievement | Journal of Career Development | Not related |
| 264 | Kleiman, E. M.;Riskind, J. H.;Schaefer, K. E.;Weingarden, H.; | 2012 | The moderating role of social support on the relationship between impulsivity and suicide risk | Crisis | Mental health disorders |
| 265 | Çivitci, Asim; | 2015 | The Moderating Role of Positive and Negative Affect on the Relationship between Perceived Social Support and Stress in College Students | Educational Sciences: Theory and Practice | Mental health disorders |
| 266 | Hazzam, J.; | 2022 | The moderating role of age on social media marketing activities and customer brand engagement on the Instagram social network | Young Consumers | Not related |
| 267 | Kautish, P.;Walia, S.;Kaur, P.; | 2021 | The moderating influence of social support on career anxiety and career commitment: an empirical investigation from India | Journal of Travel & Tourism Marketing | Mental health disorders |
| 268 | Matijczak, A.;McDonald, S. E.;Tomlinson, C. A.;Murphy, J. L.;O'Connor, K.; | 2021 | The Moderating Effect of Comfort from Companion Animals and Social Support on the Relationship between Microaggressions and Mental Health in LGBTQ+ Emerging Adults | Behavioral Sciences | The population is not college students |
| 269 | Ke, T. H.;Li, W. J.;Sanci, L.;Reavley, N.;Williams, I.;Russell, M. A.; | 2023 | The mental health of international university students from China during the COVID-19 pandemic and the protective effect of social support: A longitudinal study | Journal of Affective Disorders | Not related |
| 270 | Dong, X.;Yang, K.;Zhang, R.;Lv, Y.; | 2021 | The Mental Health and Grade Point Average among College Students from Lower Socioeconomic Status Based on Healthcare Data | J Healthc Eng | Not related |

| | | | | | |
|---|---|---|---|---|---|
| | | | Analysis | | |
| 271 | Demir, B.;Sümer, Z. H.; | 2023 | The mediator role of perceived social support in the relationship between difficulties in emotion regulation and suicide tendency | Current Psychology | Mental health disorders |
| 272 | Konan, N.;Çelik, O. T.; | 2019 | The Mediator Role of Interaction Anxiety in the Relationship between Social Support Perception and Smartphone Addiction | Journal of Education and Future-Egitim Ve Gelecek Dergisi | Mental health disorders |
| 273 | Wang, J.;Chen, Y.;Chen, H.;Hua, L.;Wang, J.;Jin, Y.;He, L.;Chen, Y.;Yao, Y.; | 2023 | The mediating role of coping strategies between depression and social support and the moderating effect of the parent-child relationship in college students returning to school: During the period of the regular prevention and control of COVID-19 | Front Psychol | The population is not college students |
| 274 | Park, S.;Choi, M.;Lee, S.; | 2019 | The mediating effects on the relationship between campus life adaptation and clinical competence | Nurse Educ Today | Mental health disorders |
| 275 | Zhou, M.;Liu, X.;Guo, J.; | 2023 | The mediating effect of self-efficacy between teacher emotional support and interaction engagement in EFL learning | Journal of Multilingual and Multicultural Development | Not related |
| 276 | Feng, S.;Zhang, L.;Lin, J.;Sun, R. W.;Wang, R. N.;Qu, H. F.;Fang, B. X.;Wang, J. N.;Yao, P.; | 2024 | The mediating effect of positive expectations in the relationship between social support and post-traumatic stress disorder symptoms among parents of children with acute lymphoblastic leukemia | Journal of Psychiatric Research | The population is not college students |
| 277 | Yi, Y.;Tan, Q.;Liu, J.;Liang, F.;Liu, C.;Yin, Z.; | 2022 | The Mechanism of Cumulative Ecological Risk Affecting College Students' Sense of Social | Int J Environ Res Public Health | Not related |

| | | | | | |
|---|---|---|---|---|---|
| | | | Responsibility: The Double Fugue Effect of Belief in a Just World and Empathy | | |
| 278 | Hasan, S.;Bagde, S.; | 2013 | The Mechanics of Social Capital and Academic Performance in an Indian College | American Sociological Review | Not related |
| 279 | Marván, Ma Luisa;Chrisler, Joan C.;Gorman, Jennifer A.;Barney, Angela; | 2017 | The meaning of menarche: A cross-cultural semantic network analysis | Health Care for Women International | Not related |
| 280 | Tomchuk, D.;Rubley, M. D.;Holcomb, W. R.;Guadagnoli, M.;Tarno, J. M.; | 2010 | The magnitude of tissue cooling during cryotherapy with varied types of compression | Journal of Athletic Training | Published not from January 2010 to 31 March 2024 |
| 281 | Moran, R.;Sperandei, S.;Peel, N.;Gray, T.;Reis, A.; | 2024 | The long-term effect of surf therapy on posttraumatic stress, depression, and anxiety symptomology among current and former Australian defense force members - A nonrandomized controlled longitudinal study in a community setting | Mental Health and Physical Activity | Mental health disorders |
| 282 | Yang, M.;Perez-Rojas, A.;Miller, M. J.; | 2024 | The interplay of friendship stress, social support, and optimism on psychological distress in college students | Counselling Psychology Quarterly | Mental health disorders |
| 283 | Hakyemez, T. C.;Mardikyan, S.; | 2021 | The interplay between institutional integration and self-efficacy in the academic performance of first-year university students: A multigroup approach | International Journal of Management Education | Not related |
| 284 | Chang, J.; | 2015 | The interplay between collectivism and social support processes among Asian and Latino | Asian American Journal of | Not related |

| | | | | | |
|---|---|---|---|---|---|
| | | | American College students | Psychology | |
| 285 | Love, H. A.;Morgan, P.; | 2024 | The Interpersonal Theory of Suicide and Relationship Satisfaction: A Daily Diary Study | Behavioral Sciences | Mental health disorders |
| 286 | Kumar, A.;Gupta, V.; | 2024 | The Intelligent, Responsible, and Humane Side of Social Media: A Case Study of a Partnership Between UP Police India and Facebook Saving Lives | Journal of Police and Criminal Psychology | Not related |
| 287 | Wenzler, S.;Keeley, J.; | 2022 | The initiation or continuation of mental health services in the transition to college | J Am Coll Health | Not related |
| 288 | Shima, T.;Nakao, H.;Tai, K.;Shimofure, T.;Jesmin, S.;Arai, Y.;Kiyama, K.;Onizawa, Y.; | 2022 | The Influences of Changes in Physical Activity Levels With Easing Restriction of Access to the University Campus on Empathy and Social Supports in College Students During the COVID-19 Pandemic | Asia Pac J Public Health | Not related |
| 289 | Belanger, N. M. S.;Patrick, J. H.; | 2018 | The Influence of Source and Type of Support on College Students' Physical Activity Behavior | J Phys Act Health | Not related |
| 290 | Guo, Yuan; | 2017 | The Influence of Social Support on the Prosocial Behavior of College Students: The Mediating Effect Based on Interpersonal Trust | English Language Teaching | Not related |
| 291 | Zhang, Y.;Hasibagen,;Zhang, C.; | 2022 | The influence of social support on the physical exercise behavior of college students: The mediating role of self-efficacy | Front Psychol | Not related |
| 292 | Abomah, P. W.; | 2021 | The Influence of Social Support on the Academic Performance/Self-Efficacy of Students in Methodist University College Students, Ghana | Texila International Journal of Public Health | Not related |

| | | | | | |
|---|---|---|---|---|---|
| 293 | Kim, M.;Jeong, Y.; | 2018 | The Influence of social support on stress coping among nursing students | Indian Journal of Public Health Research and Development | Mental health disorders |
| 294 | Wang, Y. H.;Shi, Z. T.; | 2018 | The influence of social support on the sexual and mental health of female college students | Medicine (Baltimore) | Not related |
| 295 | Lin, X. J.;Hu, Y. J.;Chen, C. M.;Zhu, Y. J.; | 2023 | The Influence of Social Support on Higher Vocational Students? Learning Motivation: The Mediating Role of Belief in a Just World and the Role of Gender | Psychology Research and Behavior Management | Not related |
| 296 | Guo, Qiang;Wang, Xiaozan;Gu, Xiangli; | 2016 | The Influence of Social Support on College Students' Physical Activity | Research Quarterly for Exercise and Sport, suppl. Supplement | Not related |
| 297 | Leite, S. V.;França, L. H. F. P.;Leite, S. B. F.; | 2021 | The influence of social support and social skills on the academic performance of younger individuals and older adult college students | Estudos de Psicologia (Campinas) | Not related |
| 298 | Choi, D. H.;Noh, G. Y.; | 2020 | The influence of social media use on attitude toward suicide through psychological well-being, social isolation, and social support | Information Communication & Society | Mental health disorders |
| 299 | Hong, M.;Dyakov, D. G.;Zheng, J.; | 2021 | The influence of self-identity on social support, loneliness, and internet addiction among Chinese college students | Journal of Psychology in Africa | Mental health disorders |
| 300 | Wu, S.;Liu, H.;Li, Y.;Teng, Y.; | 2024 | The Influence of Self-Esteem on Sociocultural Adaptation of College Students of Hong Kong, Macao, and Taiwan: The Chain Mediating Role of | Psychol Res Behav Manag | Not related |

| | | | | | |
|---|---|---|---|---|---|
| | | | Social Support and School Belonging | | |
| 301 | Wang, Rui;Wang, Mengru;Georgiev, Georgi V.; | 2023 | The Influence of Personal Evaluation and Social Support on Career Expectations of College Students | Behavioral Sciences | Not related |
| 302 | Xiang, G. C.;Teng, Z. J.;Li, Q. Q.;Chen, H.;Guo, C.; | 2020 | The influence of perceived social support on hope: A longitudinal study of older-aged adolescents in China | Children and Youth Services Review | Not related |
| 303 | Kutsuna, T.;Sugawara, H.;Kurita, H.;Kusaka, S.;Takahashi, T.; | 2021 | The influence of low-intensity resistance training combined with neuromuscular electrical stimulation on autonomic activity in healthy adults: a randomized controlled cross-over trial | Hong Kong Physiotherapy Journal: official publication of the Hong Kong Physiotherapy Association Limited = wu li chih Liao | The population is not college students. |
| 304 | Liu, H.;Zhou, Z.;Fan, X.;Wang, J.;Sun, H.;Shen, C.;Zhai, X.; | 2020 | The Influence of Left-Behind Experience on College Students' Mental Health: A Cross-Sectional Comparative Study | Int J Environ Res Public Health | Not related |
| 305 | Mao, E. R.;Zhao, L. S.; | 2023 | The influence of job search stress on college students' addictive social media use: Seeking social support and perceived social support as serial mediators and sense of coherence as a moderator | Frontiers in Psychology | Mental health disorders |
| 306 | Zhang, X.;Huang, P. F.;Li, B. Q.;Xu, W. J.;Li, W.;Zhou, B.; | 2021 | The Influence of interpersonal relationships on school adaptation among Chinese university students during COVID-19 control period: | J Affect Disord | Not related |

| | | | | | |
|---|---|---|---|---|---|
| | | | Multiple mediating roles of social support and resilience | | |
| 307 | Jiang, M. M.;Wang, D. W.;Wu, Z. Y.;Gao, K.;Guo, P. P.;Kong, Y.; | 2022 | The influence of internet use frequency, family atmosphere, and academic performance on adolescent depression: Based on the chain mediating effect of self-adjustment and campus deviant behavior | Frontiers in Psychology | Mental health disorders |
| 308 | Lipinski, D.;Whelan, J. P.;Stiglets, B. E.;Ginley, M. K.;Pfund, R. A.; | 2023 | The Influence of Gambling on Mood State and Alcohol Cravings | Journal of Gambling Studies | Mental health disorders |
| 309 | García, J. A.;Carcedo, R. J.;Castaño, J. L.; | 2019 | The Influence of Feedback on Competence, Motivation, Vitality, and Performance in a Throwing Task | Research quarterly for exercise and sport | Not related |
| 310 | Chen, Q.;Zhao, W.;Li, Q.;Sagi, H.; | 2021 | The influence of family therapy on psychological stress and social adaptability of depressed patients | Work | Unpublished journal article |
| 311 | He, Shuang;Jiang, Shouwen;Zhu, Ruilin;Hu, Xuan; | 2023 | The influence of educational and emotional support on e-learning acceptance: An integration of social support theory and TAM | Education and Information Technologies | Not related |
| 312 | Darlow, S. D.;Xu, X. M.; | 2011 | The influence of close others' exercise habits and perceived social support on exercise | Psychology of Sport and Exercise | Not related |
| 313 | Ceballos, N. A.;Watt, T. T.; | 2023 | The Influence of Adverse Childhood Experiences on Malevolent Creativity in Young Adulthood | Behav Sci (Basel) | The population is not college students |
| 314 | Liu, Y. B.;Ni, X. L.;Niu, G. F.; | 2020 | The influence of active social networking services use and social capital on flourishing in Chinese adolescents. | Children and Youth Services Review | Not related |
| 315 | Sanchez-Garciaguirre, A. I.;Najera, S. N.;Portillo, E. M.;Field, C. A.; | 2024 | The indirect effects of self-regulation on the association of social support with increased | Alcohol Clin Exp Res (Hoboken) | Mental health disorders |

| | | | | | |
|---|---|---|---|---|---|
| | | | protective drinking behavior and decreased alcohol problems in a predominantly Hispanic college student sample | | |
| 316 | Hollingsworth, David W.;Slish, Meredith L.;Wingate, LaRicka R.;Davidson, Collin L.;Rasmussen, Kathy A.;O'Keefe, Victoria M.;Tucker, Raymond P.;Grant, DeMond M.; | 2018 | The Indirect Effect of Perceived Burdensomeness on the Relationship between Indices of Social Support and Suicide Ideation in College Students | Journal of American College Health | Mental health disorders |
| 317 | Llamas, J. D.;Morgan Consoli, M.; | 2012 | The importance of family for Latina/o college students: examining the role of familial support in intragroup marginalization | Cultur Divers Ethnic Minor Psychol | Not related |
| 318 | Ou, H.;Zheng, Y.;Li, M.;Liang, J.;Chen, H.;Lang, S.;Li, Q.;Chen, D.;Lin, Y.;Chen, Q.;et al.,; | 2022 | The impacts of surgical mask in young healthy subjects on cardiopulmonary function and muscle performance: a randomized crossover trial | Archives of public health [Archives belges de sante publique] | Not related |
| 319 | Suchy, Y.;Holmes, L. G.;Strassberg, D. S.;Gillespie, A. A.;Nilssen, A. R.;Niermeyer, M. A.;Huntbach, B. A.; | 2019 | The Impacts of Sexual Arousal and Its Suppression on Executive Functioning | Journal of sex research | Not related |
| 320 | Huang, Yiman;Su, Xiaoyou;Si, Mingyu;Xiao, Weijun;Wang, Hao;Wang, Wenjun;Gu, Xiaofen;Li, Ma;Li, Jing;Zhang, Shaokai;Ren, Zefang;Qiao, Youlin; | 2021 | The impacts of coping style and perceived social support on the mental health of undergraduate students during the early phases of the COVID-19 pandemic in China: a multicenter survey | BMC Psychiatry | Not related |
| 321 | Wiegers, E.;Garner, A.;Jusko, | 2024 | The Impact of Stimulant Medication on the | Research on Child | Mental health disorders |

| | | | | | |
|---|---|---|---|---|---|
| | M.;Smith, J. N.;Campez, M.;Greiner, A.;Gnagy, E.;Pelham, W. E.;Raiker, J.; | | Relation Between Working Memory and Activity Level in ADHD | and Adolescent Psychopathology | |
| 322 | D'Alessandro, A. M.;Peltier, J. W.;Dahl, A. J.; | 2012 | The Impact of social, cognitive and attitudinal dimensions on college students' support for organ donation | Am J Transplant | Not related |
| 323 | Wei, P. J.; | 2022 | The impact of social support on students' mental health: A new perspective based on fine art majors | Frontiers in Psychology | Not related |
| 324 | Dhruve, D. M.;Russo, J. E.;Oliveros, A. D.; | 2023 | The impact of social support and emotion dysregulation on COVID-19 depressive symptoms | Front Psychol | Mental health disorders |
| 325 | Wang, C.;Mattingly, S.;Payne, J.;Lizardo, O.;Hachen, D. S.; | 2021 | The impact of social networks on sleep among a cohort of college students | SSM Popul Health | Not related |
| 326 | Jackson, Theodore;Kim, Don; | 2023 | The Impact of Social Networking Sites on Social Well-Being During the Pandemic | The Journal of Applied Business and Economics | Not related |
| 327 | Xu, Y.;Razak, R. R. A.;Xiang, M.; | 2024 | The impact of social media on the happiness of Chinese college students | Heliyon | Not related |
| 328 | Levi-Belz, Y.;Blank, C.;Groweiss, Y.;Neria, Y.; | 2024 | The impact of potentially morally injurious experience of betrayal on PTSD and depression following the October 7th terror attack | Scientific Reports | Mental health disorders |
| 329 | Salami, T.;Lawson, E.;Metzger, I. W.; | 2021 | The impact of Microaggressions on Black college students' worry about their future employment: The moderating role of social support and academic achievement | Cultur Divers Ethnic Minor Psychol | Not related |
| 330 | Figueiro, M. G.;Wood, B.;Plitnick, B.;Rea, M. S.; | 2011 | The impact of light from computer monitors on melatonin levels in college students | Neuro endocrinology | Not related |

| | | | | | |
|---|---|---|---|---|---|
| | | | | letters | |
| 331 | Chen, J.;Shi, L.;Xiao, S.;Zheng, X.;Xue, Y.;Xue, B.;Zhang, J.;Li, X.;Chen, Y.;Wu, Y.;Zhang, C.; | 2024 | The impact of intimate partner violence on depressive symptoms among college students: A moderated mediation model of the big five personality traits and perceived social support | J Affect Disord | Mental health disorders |
| 332 | Vasquez-Salgado, Y.;Ramirez, G.;Greenfield, P. M.; | 2018 | The impact of home-school cultural value conflicts and President Trump on Latina/o first-generation college students' attentional control | International Journal of Psychology | Not related |
| 333 | Perrine, B. L.;Monzón, K.;Weber, L. M.;Funderburk, L. K.; | 2021 | The Impact of Fish Oil Supplementation on Self-Perception of the Voice in Vocal Performers: a Randomized, Single-Blind, Placebo-Controlled Study | Journal of Speech, Language, and Hearing Research | Not related |
| 334 | Suwinyattichaiporn, T.;Johnson, Z. D.; | 2022 | The Impact of Family and Friends Social Support on Latino/ First-Generation College Students' Perceived Stress, Depression, and Social Isolation | Journal of Hispanic Higher Education | Mental health disorders |
| 335 | Yousef, S.;Yousef, K.; | 2022 | The impact of Facebook usage in education on students' academic performance at the University of Jordan | Journal of E-Learning and Knowledge Society | Not related |
| 336 | Al-Fadhli, Salah;Al-Saleh, Yaser; | 2012 | The Impact of Facebook on Political Engagement in Kuwait | Journal of the Social Sciences | Not related |
| 337 | Busalim, A. H.;Masrom, M.;Zakaria, Wnbw; | 2019 | The impact of Facebook Addiction and self-esteem on students' academic performance: A multi-group analysis | Computers & Education | Mental health disorders |
| 338 | Frison, E.;Eggermont, S.; | 2015 | The impact of daily stress on adolescents' depressed mood: The role of social support seeking through Facebook | Computers in Human Behavior | Mental health disorders |

| | | | | | |
|---|---|---|---|---|---|
| 339 | Bhat, R. M.;Rangaiah, B.; | 2015 | The impact of conflict exposure and social support on posttraumatic growth among young adults in Kashmir | Cogent Psychology | The population is not college students |
| 340 | Gasik, R. E.;Madkour, A. S.;Skeen, S. J.;Clum, G.;Francis, T.;Felker-Kantor, E.;Ferguson, T.;Welsh, D. A.;Molina, P. E.;Theall, K. P.; | 2024 | The Impact of Childhood Adversity on Life Course Alcohol Use Patterns and Health Status Among People Living with HIV | AIDS and Behavior | The population is not college students |
| 341 | Park, Chulwoo;Shimada, Shannon; | 2022 | The impact of changing nonimmigrant visa policies on international students' psychological adjustment and well-being in the United States during the COVID-19 pandemic: a qualitative study | BMC Public Health | Not related |
| 342 | Zhao, A.; | 2023 | The impact of career expectation on employment anxiety of art students in higher vocational colleges during the COVID-19: A chain mediating role of social support and psychological capital | Front Psychol | Mental health disorders |
| 343 | Byrow, Y.;Nickerson, A.;Specker, P.;Bryant, R.;O'Donnell, M.;McMahon, T.;Mau, V.;Liddell, B.; | 2024 | The impact of age-related differences in emotion dysregulation on refugee mental health and social outcomes | Journal of Traumatic Stress | Not related |
| 344 | Gonzalez, C.;Finley, J. C. A.;Khalid, E.;Basurto, K. S.;Vanlandingham, H. B.;Frick, L. A.;Brooks, J. M.;Ellison, R. L.;Ulrich, D. M.;Soble, J. R.;Resch, Z. J.; | 2024 | The Impact of Adverse Childhood Experiences on Symptom and Performance Validity Tests Among a Multiracial Sample Presenting for ADHD Evaluation | Archives of Clinical Neuropsychology | Mental health disorders |

| | | | | | |
|---|---|---|---|---|---|
| 345 | Arabyat, R. M.;Borrego, M.;Hamidovic, A.;Sleath, B.;Raisch, D. W.; | 2019 | The impact of a theory-based web intervention on the intention to use prescription drugs for non-medical purposes among college students: a randomized controlled trial | Health education research | Mental health disorders |
| 346 | Hershner, S.;O'Brien, L. M.; | 2018 | The Impact of a Randomized Sleep Education Intervention for College Students | Journal of Clinical Sleep Medicine | Mental health disorders |
| 347 | Daddario, N. B.;Felipes, R. C. S.;Cooney, J. V.;Stephenson, K. M.;Shleiwet, N. H.;Liang, T.;Jafri, F. N.; | 2021 | The Impact of a Mobile Phone Application for Retention of Bleeding Control Skills | Journal of Surgical Research | Not related |
| 348 | Yang, X. H.;Yu, H. J.;Liu, M. W.;Zhang, J.;Tang, B. W.;Yuan, S.;Gasevic, D.;Paul, K.;Wang, P. G.;He, Q. Q.; | 2020 | The impact of a health education intervention on health behaviors and mental health among Chinese college students | Journal of American College Health | Mental health disorders |
| 349 | Manboard, M.;Johnson, C. M.;Thornton, H.;Biediger-Friedman, L.; | 2021 | The HOME Study: Understanding How College Students at a Hispanic Serving Institution Coped with Food Insecurity in a Pandemic | Int J Environ Res Public Health | Not related |
| 350 | Boerchi, D.;Magnano, P.;Lodi, E.; | 2021 | The High School Competencies Scale (H-Comp Scale): A First Validation Study | Eur J Investig Health Psychol Educ | The population is not college students |
| 351 | Vidal, C.;Silverman, J.;Petrillo, E. K.;Lilly, F. R. W.; | 2022 | The health-promoting effects of social flourishing in young adults: A broad view on the relevance of social relationships | Social Science Journal | The population is not college students |
| 352 | Ibrahim, Ali; | 2018 | The happiness of undergraduate students at one university in the United Arab Emirates | International Journal of Research Studies in Language | Not related |

| | | | | | |
|---|---|---|---|---|---|
| | | | | Learning | |
| 353 | Renn, T.;Dowdy-Hazlett, T.;Collins, C.;Killian, M.;Alani, D.; | 2024 | The Feasibility and Acceptability of Cognitive Behavioral Intervention for Trauma in Schools (CBITS) in a Rural Community Impacted by Environmental Trauma and COVID-19 | School Mental Health | Mental health disorders |
| 354 | Sakthivelrani, S.;Vijayalakshmi, K.; | 2017 | The factors influencing the academic achievement of college students | Journal of Advanced Research in Dynamical and Control Systems | Not related |
| 355 | McLeod, J. D.;Meanwell, E.;Hawbaker, A.; | 2019 | The Experiences of College Students on the Autism Spectrum: A Comparison to Their Neurotypical Peers | J Autism Dev Disord | Not related |
| 356 | Fried, A. B.;Dunn, M. E.; | 2012 | The Expectancy Challenge alcohol literacy curriculum (ECALC): a single session group intervention to reduce alcohol use. | Psychology of addictive behaviors | Mental health disorders |
| 357 | Wang, P. C.;Lei, L.;Wang, X. C.;Nie, J.;Chu, X. Y.;Jin, S. N.; | 2018 | The exacerbating role of perceived social support and the "buffering" role of depression in the relation between sensation seeking and adolescent smartphone addiction | Personality and Individual Differences | Mental health disorders |
| 358 | Meng, X.;Zhang, J.;Ren, G.; | 2021 | The Evaluation Model of College Students' Mental Health in the Environment of Independent Entrepreneurship Using Neural Network Technology | J Healthc Eng | Not related |
| 359 | Mrakotsky, C.;Walsh, K. S.;Buranahirun Burns, C.;Croteau, S. E.;Markert, A.;Geybels, | 2024 | The Thing Study: Cognitive and Behavioral Outcomes in Children with Hemophilia | Journal of Pediatrics | The population is not college students |

| | | | | | |
|---|---|---|---|---|---|
| | M.;Hannemann, C.;Rajpurkar, M.;Shapiro, K. A.;Wilkening, G. N.;Ventola, P.;Cooper, D. L.; | | | | |
| 360 | Zinn, T. E.;Newland, M. C.;Ritchie, K. E.; | 2015 | The efficiency and efficacy of equivalence-based learning: a randomized controlled trial | Journal of Applied Behavior Analysis | Not related |
| 361 | Tomoiaga, C.;David, O.; | 2022 | The Efficacy of Guided and Unguided Game-Based Cognitive-Behavioral Therapy in Reducing Distress in College Students | Games for Health Journal | Mental health disorders |
| 362 | Canale, N.;Vieno, A.;Santinello, M.;Chieco, F.;Andriolo, S.; | 2015 | The efficacy of computerized alcohol intervention tailored to drinking motives among college students: a quasi-experimental pilot study | American journal of drug and alcohol abuse | Mental health disorders |
| 363 | Donath, L.;Roth, R.;Hohn, Y.;Zahner, L.;Faude, O.; | 2014 | The effects of Zumba training on cardiovascular and neuromuscular function in female college students | European journal of sport science | Not related |
| 364 | Kattelmann, K. K.;Bredbenner, C. B.;White, A. A.;Greene, G. W.;Hoerr, S. L.;Kidd, T.;Colby, S.;Horacek, T. M.;Phillips, B. W.;Koenings, M. M.;et al.,; | 2014 | The effects of Young Adults Eating and Active for Health (YEAH): a theory-based Web-delivered intervention | Journal of nutrition education and behavior | The population is not college students |
| 365 | Albracht-Schulte, K.;Robert-McComb, J.; | 2018 | The effects of yoga and quiet rest on subjective levels of anxiety and physiological correlates: a 2-way crossover randomized trial | BMC complementary and alternative medicine | Mental health disorders |
| 366 | Zhang, W.;Lang, S.;Zheng, Y.;Qin, X.;Chen, H.;You, Y.;Ou, H.; | 2019 | The Effects of Transcranial Direct Current Stimulation Versus Electroacupuncture on Working Memory in Healthy Subjects | Journal of alternative and complementary medicine (New | Not related |

| | | | | | |
|---|---|---|---|---|---|
| | | | | York, N.Y.) | |
| 367 | Edmundson, M.;Berry, D. T. R.;Combs, H. L.;Brothers, S. L.;Harp, J. P.;Williams, A.;Rojas, S. L.;Saleh, A. K.;Scott, A. B.; | 2017 | The effects of symptom information coaching on the feigning of adult ADHD | Psychological assessment | Mental health disorders |
| 368 | Kwan, M. Y.;Gordon, K. H.; | 2016 | The effects of social support and stress perception on bulimic behaviors and unhealthy food consumption | Eat Behav | Mental health disorders |
| 369 | Wang, H. I.; | 2013 | The effects of social network sites on learning performance: The study of college students in Taiwan | International Journal of Computational Science and Engineering | Not related |
| 370 | Singh, J. S.;Capozzoli, M. C.;Dodd, M. D.;Hope, D. A.; | 2015 | The Effects of Social Anxiety and State Anxiety on Visual Attention: testing the Vigilance-Avoidance Hypothesis | Cognitive behavior therapy | Mental health disorders |
| 371 | Kingsbury, J. H.;Gibbons, F. X.;Gerrard, M.; | 2015 | The Effects of social and health consequence framing on heavy drinking intentions among college students | British journal of health psychology | Not related |
| 372 | Austin, Williamson J.; | 2024 | The effects of relationship history on social support perceived from friendships | Journal of Social and Personal Relationships | Not related |
| 373 | Chan, D. M.;Broda, M. D.;Winslow, J.;Jones, Q.;Luce, C.;McGinnis, H. A.;Tomlinson, C. A.;Hamid, H.;Ma, J.; | 2022 | The Effects of Prime Supporters within a College Student's Support Network | Nonlinear Dynamics Psychol Life Sci | Not related |

| | | | | | |
|---|---|---|---|---|---|
| 374 | Niyonsenga, J.;Uwingeneye, L.;Musabyemariya, I.;Nteziryayo, J. P.;Siboyintore, T.;Sagahutu, J. B.;Cavallini, F.;Eugene, R.;Jansen, S.;Monacelli, N.;Caricati, L.;Mutabaruka, J.; | 2024 | The effects of perceived therapist guidance and advice on adherence to home-based exercise programs in mothers of children with cerebral palsy in Rwanda | Heliyon | The population is not college students |
| 375 | Ergin, D. A.; | 2021 | The effects of perceived discrimination, social support, and ethnic identity on the mental health of immigrant adolescents | Scandinavian Journal of Child and Adolescent Psychiatry and Psychology | Not related |
| 376 | Liu, C.;Chen, C. S.;Ho, W. H.;Füle, R. J.;Chung, P. H.;Shiang, T. Y.; | 2013 | The effects of passive leg press training on jumping performance, speed, and muscle power | Journal of strength and conditioning research | Not related |
| 377 | Yang, S. Y.;Oh, Y. H.; | 2022 | The effects of neonatal resuscitation gamification program using immersive virtual reality: a quasi-experimental study | Nurse education today | Not related |
| 378 | Basala, T.;Morin, B.;Durocher, J.; | 2022 | The Effects of Morning vs. Evening Mindfulness Meditation on Sleep, Anxiety, and Decentering: a Pilot Analysis | FASEB journal | Mental health disorders |
| 379 | Heinrich, D. S.;O'Connell, K. A.; | 2024 | The Effects of Mindfulness Meditation on Nursing Students' Stress and Anxiety Levels | Nursing education perspectives | Mental health disorders |
| 380 | Zheng, G.;Li, K.;Bu, W.;Wang, Y.; | 2019 | The effects of indoor high temperature on circadian rhythms of human work efficiency | International journal of environmental research and public | Not related |

| | | | | | |
|---|---|---|---|---|---|
| | | | | health | |
| 381 | Zhang, J.;Zhang, N.;Du, S.;He, H.;Xu, Y.;Cai, H.;Guo, X.;Ma, G.; | 2018 | The effects of hydration status on cognitive performances among young adults in Hebei, China: a randomized controlled trial (RCT) | International journal of environmental research and public health | The population is not college students |
| 382 | Chang, Y. H.;Chou, Y. C.;Chang, Y. C.;Tan, K. H.;Wu, M. H.; | 2022 | The Effects of High-Intensity Power Training versus Traditional Resistance Training on Exercise Performance | International journal of environmental research and public health | Not related |
| 383 | Hsu, C. L.;Lin, J. C. C.; | 2023 | The effects of gratifications, flow, and satisfaction on the usage of live streaming services | Library Hi Tech | Not related |
| 384 | Brown, A. L.; | 2019 | The Effects of Exposure to Negative Social Reactions and Participant Gender on Attitudes and Behavior Toward a Rape Victim | Violence Against Women | Not related |
| 385 | Nagurney, Alexander; | 2013 | The Effects of Emotional Writing on Psychological Well-Being | North American Journal of Psychology | Not related |
| 386 | Kwan, R. Y. C.;Ng, F.;Lai, M.;Wong, D.;Chan, S.; | 2023 | The effects of Digital Buddy program on older adults' mental well-being: study protocol for a multi-center, cluster randomized controlled trial | Trials | The population is not college students |
| 387 | Rudisill, T. M.;Innes, K. K.;Wen, S.;Haggerty, T.;Smith, G. S.; | 2023 | The effects of cannabidiol on subjective states, cognition, and psychomotor function in healthy adults: a randomized clinical trial | Fundamental & clinical pharmacology | The population is not college students |

| | | | | | |
|---|---|---|---|---|---|
| 388 | Csepregi, E.;Szekanecz, Z.;Szanto, S.; | 2020 | The effects of breathing exercises in comparison with other exercise programs on cardiorespiratory fitness among healthy female college students | Journal of sports medicine and physical fitness | Not related |
| 389 | Frampton, S. E.;Linehan, E.; | 2024 | The effects of a training package to teach note-taking on the formation of equivalence classes | Journal of the experimental analysis of behavior | Not related |
| 390 | Grimes, A.;Baker, M.; | 2020 | The Effects of a Citywide Bike Share System on Active Transportation Among College Students: a Randomized Controlled Pilot Study | Health education & behavior | Not related |
| 391 | Rekenyi, V.;Garbóczy, S.;Szemán-Nagy, A.;Al-Tammemi, A. B.;Sayed-Ahmad, M.;Kolozsvári, L. R.; | 2023 | The Effects and Differences of Social Support, Depression, and Vital Exhaustion during the COVID-19 Pandemic among International and Domestic University Students | International journal of environmental research and public health | Mental health disorders |
| 392 | Zheng, G.;Lan, X.;Li, M.;Ling, K.;Lin, H.;Chen, L.;Tao, J.;Li, J.;Zheng, X.;Chen, B.;et al.,; | 2014 | The effectiveness of Tai Chi on the physical and psychological well-being of college students: a study protocol for a randomized controlled trial | Trials | Not related |
| 393 | Duan, Y.;Liang, W.;Wang, Y.;Lippke, S.;Lin, Z.;Shang, B.;Baker, J. S.; | 2022 | The Effectiveness of Sequentially Delivered Web-Based Interventions on Promoting Physical Activity and Fruit-Vegetable Consumption Among Chinese College Students: mixed Methods Study | Journal of medical Internet research | Mental health disorders |
| 394 | Bodley-Scott, E.;Ward, R. J.;Tarabay, J.;Fagbamigbe, A. F.;Barker, S.;Maguire, N.; | 2024 | The effectiveness of psychological interventions for people experiencing homelessness: A systematic review and meta-analysis | Journal of Community and Applied Social Psychology | Meta review |
| 395 | Bernstein, M. H.;Wood, M. | 2016 | The Effectiveness of Message Framing and | Alcohol and | Mental health disorders |

| | | | | | |
|---|---|---|---|---|---|
| | D.;Erickson, L. R.; | | Temporal Context on College Student Alcohol Use and Problems: a Selective E-Mail Intervention | alcoholism (Oxford, Oxfordshire) | |
| 396 | Amzajerdi, A.;Keshavarz, M.;Ghorbali, E.;Pezaro, S.;Sarvi, F.; | 2023 | The effect of vitamin D on the severity of dysmenorrhea and menstrual blood loss: a randomized clinical trial | BMC women's health | Mental health disorders |
| 397 | Junco, R.;Heiberger, G.;Loken, E.; | 2011 | The effect of Twitter on college student engagement and grades | Journal of Computer-Assisted Learning | Not related |
| 398 | Guo, Y.;Xu, M.;Zhang, J.;Hu, Q.;Zhou, Z.;Wei, Z.;Yan, J.;Chen, Y.;Lyu, J.;Shao, X.;et al.,; | 2018 | The effect of Three-Circle Post Standing (Zhan Zhuang) Qigong on the physical and psychological well-being of College students: study protocol for a randomized controlled trial | Medicine | Not related |
| 399 | Lee, J. H.;Cho, Y.;Kang, K. H.;Cho, G. C.;Song, K. J.;Lee, C. H.; | 2016 | The Effect of the Duration of Basic Life Support Training on the Learners' Cardiopulmonary and Automated External Defibrillator Skills | BioMed research international | Not related |
| 400 | Craig, D. J.;Fardouly, J.;Rapee, R. M.; | 2022 | The Effect of Spirituality on Mood: Mediation by Self-Esteem, Social Support, and Meaning in Life | Journal of Religion & Health | Not related |
| 401 | Zhang, N.;Ding, K. L.;Park, C.;Vo, J.;Cantos, K. M.; | 2023 | The Effect of Social Support on Social Media on Asian College Students' Intention to Participate in Physical Activity in the United States | Health Equity | Not related |
| 402 | Shi, H.; | 2024 | The effect of social support on home isolation anxiety and depression among college students in the post-pandemic era: the mediating effect of perceived loss of control and the moderating role of family socioeconomic status | Front Public Health | Mental health disorders |
| 403 | Yu, Xianglian;Xiong, Fen;Zhang, | 2023 | The Effect of Social Support on Depression among | International | Mental health disorders |

| | | | | | |
|---|---|---|---|---|---|
| | Hanbing;Ren, Zhihong;Liu, Lianzhong;Zhang, Lin;Zhou, Zongkui; | | Economically Disadvantaged College Students: The Mediating Role of Psychological Resilience and the Moderating Role of Geography | journal of environmental research and public health | |
| 404 | O'Neill, M.; | 2019 | The effect of social support on community college students experiencing food insecurity: An overlooked population | Social Work and Social Sciences Review | Not related |
| 405 | Tafesse, Wondwesen; | 2020 | The effect of social networking site use on college students' academic performance: the mediating role of student engagement | Education and Information Technologies | Not related |
| 406 | Bhaskara, N. V.;Nandanur, B. S.;Chakraborty, A.;Ghosh, S.; | 2020 | The effect of social media usage on the mental well-being of medical college students in Bangalore, Karnataka | J Family Med Prim Care | Not related |
| 407 | Fruehwirth, J. C.;Gorman, B. L.;Perreira, K. M.; | 2021 | The Effect of Social and Stress-Related Factors on Alcohol Use Among College Students During the Covid-19 Pandemic | J Adolesc Health | Mental health disorders |
| 408 | Shim, Jihyun;Lee, Jiyeon; | 2023 | The Effect of Positive Psychological Capital of College Students on College Adaptation in the COVID-19 Situation: The Moderating Effect of Social Support | Review of Integrative Business and Economics Research | Not related |
| 409 | Yu, S. C.;Chang, C. W.; | 2022 | The Effect of Positive Intervention Dosing Frequency: fixed Intervals May Decrease More Depression than Flexible Ones | International journal of environmental research and public health | Mental health disorders |
| 410 | Liu, M.;Shi, B.; | 2023 | The effect of physical exercise on the anxiety of | Front Psychol | Mental health disorders |

| | | | | | |
|---|---|---|---|---|---|
| | | | college students in the post-pandemic era: The mediating role of social support and proactive personality | | |
| 411 | Zhang, J.;Zheng, S.;Hu, Z.; | 2022 | The Effect of Physical Exercise on Depression in College Students: The Chain Mediating Role of Self-Concept and Social Support | Front Psychol | Mental health disorders |
| 412 | Chang, C. M.;Chou, Y. H.;Hsieh, H. H.;Huange, C. K.; | 2020 | The effect of participation motivations on interpersonal relationships and learning achievement of female college students in sports club: The moderating role of club involvement | International journal of environmental research and public health | Not related |
| 413 | Cheuk Yan, Sing;Wong, W. S.; | 2011 | The effect of optimism on depression: the mediating and moderating role of insomnia | J Health Psychol | Mental health disorders |
| 414 | Hussien, F. M.;Hassen, A. M.;Asfaw, Z. A.;Ahmed, A. Y.;Hassen, H. Y.; | 2021 | The effect of mobile text messages on knowledge and perception towards cancer and behavioral risks among college students, Northeast Ethiopia: a randomized controlled trial protocol | PLoS ONE | Not related |
| 415 | Sun, Y.; | 2022 | The Effect of Mental Health Intervention Before English Test on College Students' English Test Scores | Occup Ther Int | Mental health disorders |
| 416 | Nasiri, M. A.;Sajadi, S. A.;Farsi, Z.;Heidarieh, M.; | 2024 | The effect of mandala coloring and free coloring on the happiness in veterans with post-traumatic stress disorder in the Covid-19 pandemic: a randomized clinical trial | BMC Psychiatry | Mental health disorders |
| 417 | Xu, C.;Zhang, Z.; | 2021 | The Effect of Law Students in Entrepreneurial Psychology Under Artificial Intelligence | Front Psychol | Not related |

| | | | | | |
|---|---|---|---|---|---|
| | | | Technology | | |
| 418 | Wang, Z.;An, G.;Zhang, W.;Yang, G.; | 2019 | The effect of jazz dance on the physical and mental health of students with different physical fitness | Journal of sports medicine and physical fitness | Not related |
| 419 | Guo, Y.;Xu, M.;Ji, M.;Wei, Z.;Zhang, J.;Hu, Q.;Yan, J.;Chen, Y.;Lyu, J.;Shao, X.;et al.,; | 2018 | The effect of Imaginary Working Qigong on the psychological well-being of college students: study protocol for a randomized controlled trial | Medicine | Not related |
| 420 | Stark, A. M.;Tousignant, O. H.;Fireman, G. D.; | 2022 | The Effect of Frames on Personal Narratives of Bullying Memories | Psychological reports | Not related |
| 421 | Susanty, E.;Sijbrandij, M.;Srisayekti, W.;Suparman, Y.;Huizink, A. C.; | 2024 | The effect of eye movement desensitization on neurocognitive functioning compared to retrieval-only in PTSD patients: a randomized controlled trial | BMC Psychiatry | Mental health disorders |
| 422 | Jimenez, J.;Erdman, N. K.;Hart, J. M.;Resch, J. E.; | 2023 | The Effect of Exercise on a Novel Dual-Task Assessment for Sport Concussion | Medicine and science in sports and exercise | Not related |
| 423 | Bolinski, F.;Boumparis, N.;Kleiboer, A.;Cuijpers, P.;Ebert, D. D.;Riper, H.; | 2020 | The effect of e-mental health interventions on academic performance in university and college students: a meta-analysis of randomized controlled trials | Internet interventions | Meta review |
| 424 | Li, M.;Fang, Q.;Li, J.;Zheng, X.;Tao, J.;Yan, X.;Lin, Q.;Lan, X.;Chen, B.;Zheng, G.;et al.,; | 2015 | The Effect of Chinese Traditional Exercise-Baduanjin on Physical and Psychological Well-Being of College Students: a Randomized Controlled Trial | PLoS ONE | Not related |
| 425 | Wu, R.;Zhong, S. Y.;Wang, G. H.;Wu, M. Y.;Xu, J. F.;Zhu, H.;Liu, | 2023 | The Effect of Brief Mindfulness Meditation on Suicidal Ideation, Stress and Sleep Quality | Archives of suicide research | Mental health disorders |

| | | | | | |
|---|---|---|---|---|---|
| | L. L.;Su, W. J.;Cao, Z. Y.;Jiang, C. L.; | | | | |
| 426 | Li, X. S.;Jia, X. M.; | 2022 | The Effect of Boredom on College Students' Meaning in Life: A Longitudinal Mediation Model | International journal of environmental research and public health | Not related |
| 427 | Leisman, G.;Wallach, J.;Machado-Ferrer, Y.;Chinchilla-Acosta, M.;Meyer, A. G.;Lebovits, R.;Donkin, S.; | 2024 | The effect of binaural pulse modulation (B.P.M) on brain state in depression and anxiety: a case series | Journal of Medical Case Reports | Mental health disorders |
| 428 | Zheng, G.;Li, M.;Lan, X.;Yan, X.;Lin, Q.;Chen, L.;Tao, J.;Zheng, X.;Li, J.;Chen, B.;et al.,; | 2013 | The effect of Baduanjin exercise for the physical and psychological well-being of college students: study protocol for a randomized controlled trial | Trials | Mental health disorders |
| 429 | Zhang, Shuhua;Li, Hong;Li, Hai;Zhao, Shuo; | 2024 | The effect of autistic traits on prosocial behavior: The chain mediating role of received social support and perceived social support | Autism | Not related |
| 430 | Mohebi, S.;Sharifirad, G. H.;Shahsiah, M.;Botlani, S.;Matlabi, M.;Rezaeian, M.; | 2012 | The Effect of assertiveness training on student's academic anxiety | JPMA. The Journal of the Pakistan Medical Association | Mental health disorders |
| 431 | Lee, L. L.;Kuo, Y. C.;Fanaw, D.;Perng, S. J.;Juang, I. F.; | 2012 | The effect of an intervention combining self-efficacy theory and pedometers on promoting physical activity among adolescents | Journal of Clinical Nursing | Mental health disorders |
| 432 | Rezaei, F.;Hosseini Ramaghani, N. A.;Fazio, R. L.; | 2017 | The effect of a third party observer and trait anxiety on neuropsychological performance: the | Clinical neuropsychologist | Mental health disorders |

| | | | | | |
|---|---|---|---|---|---|
| | | | Attentional Control Theory (ACT) perspective | | |
| 433 | D'Aiello, B.;Di Vara, S.;De Rossi, P.;Vicari, S.;Menghini, D.; | 2024 | The effect of a single dose of methylphenidate on attention in children and adolescents with ADHD and comorbid Oppositional Defiant Disorder | PLoS ONE | Mental health disorders |
| 434 | Park, Y. H.;Park, Y. S.;Lee, Y. T.;Shin, H. S.;Oh, M. K.;Hong, J.;Lee, K. Y.; | 2016 | The effect of a core exercise program on Cobb angle and back muscle activity in male students with functional scoliosis: a prospective, randomized, parallel-group, comparative study | Journal of International Medical Research | Not related |
| 435 | Monroy, M.;Garcia, S. B.;Mendoza-Denton, R.;Keltner, D.; | 2021 | The Dynamics of Coping, Positive Emotions, and Well-Being: Evidence From Latin American Immigrant Farmworkers and College Students During a Time of Political Strife | Emotion | Not related |
| 436 | Li, T.;Wang, S. W.;Zhou, J. J.;Ren, Q. Z.;Gao, Y. L.; | 2021 | The Direct and Indirect Effect of Event Severity, Social Support, and optimism on stress-related growth in emerging adults | Psychol Health Med | The population is not college students |
| 437 | Zhan, Q.;Wang, Q.; | 2021 | The Development of the College Students' Experience of Family Harmony Questionnaire (CSEFHQ) | Front Psychol | Not related |
| 438 | Tennant, J. E.;Demaray, M. K.;Coyle, S.;Malecki, C. K.; | 2015 | The dangers of the web: Cyber victimization, depression, and social support in college students | Computers in Human Behavior | Mental health disorders |
| 439 | Tan, M.;Barkus, E.;Favelle, S.; | 2021 | The cross-lagged relationship between loneliness, social support, and psychotic-like experiences in young adults | Cognitive Neuropsychiatry | The population is not college students |
| 440 | Champ, R. E.;Adamou, M.;Gillibrand, W.;Arrey, S.;Tolchard, B.; | 2024 | The Creative Awareness Theory: A Grounded Theory Study of Inherent Self-Regulation in Attention Deficit Hyperactivity Disorder | Journal of Clinical Medicine | Mental health disorders |

| | | | | | |
|---|---|---|---|---|---|
| 441 | Totonchi, D. A.;Tibbetts, Y.;Williams, C. L.;Francis, M. K.;DeCoster, J.;Lee, G. A.;Hull, J. W.;Hulleman, C. S.; | 2023 | The cost of being first: Belonging uncertainty predicts math motivation and achievement for first-generation, but not continuing-generation, students | Learning and Individual Differences | Not related |
| 442 | Liu, H.;Dong, Y.;Li, L.; | 2022 | The Correlation Between College Students' Interpersonal Skills and Their Entrepreneurial Performance | International Journal of Emerging Technologies in Learning | Not related |
| 443 | Özdemir, M.;Yazıcı, H.;Tan, Y. W.; | 2022 | The contribution of self-compassion in the relationship between social support and posttraumatic growth | Revue Europeenne de Psychologie Appliquee | Not related |
| 444 | Tullis, K. A.;Kowalske, M. G.; | 2021 | The composition of social support networks of URM graduate students at predominantly white institutions | Social Network Analysis and Mining | Not related |
| 445 | Schmitz, R. M.;Tyler, K. A.; | 2018 | The Complexity of Family Reactions to Identity among Homeless and College Lesbian, Gay, Bisexual, Transgender, and Queer Young Adults | Arch Sex Behav | The population is not college students |
| 446 | Chang, J.;Wang, S. W.;Mancini, C.;McGrath-Mahrer, B.;Orama de Jesus, S.; | 2020 | The complexity of cultural mismatch in higher education: Norms affecting first-generation college students' coping and help-seeking behaviors | Cultur Divers Ethnic Minor Psychol | Not related |
| 447 | Inci Izmir, S. B.;Aktan, Z. D.;Ercan, E. S.; | 2024 | The Comparison of Psychological Factors and Executive Functions of Children with Attention Deficit Hyperactivity Disorder and Cognitive Disengagement Syndrome to ADHD and ADHD Comorbid with Oppositional Defiant Disorder | Journal of Attention Disorders | Mental health disorders |

| | | | | | |
|---|---|---|---|---|---|
| 448 | Thomas, J. J.;Borrayo, E. A.; | 2014 | The combined influence of psychosocial factors on illness behavior among women | Women Health | Not related |
| 449 | Hester, R. K.;Delaney, H. D.;Campbell, W.; | 2012 | The College Drinker's check-up: outcomes of two randomized clinical trials of a computer-delivered intervention | Psychology of addictive behaviors | Mental health disorders |
| 450 | Vishnevsky, G.;Fisher, T.;Specktor, P.; | 2024 | The clock drawing test (CDT) in the digital era: Underperformance of Generation Z adults | Journal of the Neurological Sciences | The population is not college students |
| 451 | Mihailidis, P.; | 2014 | The civic-social media disconnect: exploring perceptions of social media for engagement in the daily life of college students | Information Communication and Society | Not related |
| 452 | Sabella, Mel S.;Mardis, Kristy L.; | 2017 | The Chi-Sci Scholars Program: "Developing Community and Challenging Racially Inequitable Measures of Success at a Minority-Serving Institution on Chicago's Southside" | Physics Teacher | Not related |
| 453 | Kardas, Ferhat;Yalcin, Ilhan; | 2021 | The Broaden-and-Built Theory of Gratitude: Testing a Model of Wellbeing and Resilience on Turkish College Students | Participatory Educational Research | Not related to research questions |
| 454 | Koenig Kellas, J.;Horstman, H. K.;Willer, E. K.;Carr, K.; | 2015 | The benefits and risks of telling and listening to stories of difficulty over time: experimentally testing the expressive writing paradigm in the context of interpersonal communication between friends | Health communication | Not related |
| 455 | Cao, Juan;Wang, Kun;Shi, YuHui;Pan, YuQing;Lyu, MoHan;Ji, Ying; | 2023 | The associations between social support change and physical activity trajectory from late adolescence to young adulthood | BMC Public Health | Not related |

| | | | | | |
|---|---|---|---|---|---|
| 456 | Ding, Y.;Wan, X.;Lu, G.;Huang, H.;Liang, Y.;Yu, J.;Chen, C.; | 2022 | The associations between smartphone addiction and self-esteem, self-control, and social support among Chinese adolescents: A meta-analysis | Front Psychol | Meta review |
| 457 | Delaney, Eryn N.;Williams, Chelsea Derlan;Mosley, Della V.;Hawn, Sage E.;Dick, Danielle M.; | 2022 | The Associations Between Sexual Victimization and Health Outcomes Among LGBQA College Students: Examining the Moderating Role of Social Support | Journal of Interpersonal Violence | Not related |
| 458 | Liu, Y. L.;Chen, J. J.;Chen, K.;Liu, J.;Wang, W.; | 2023 | The associations between academic stress and depression among college students: A moderated chain mediation model of negative affect, sleep quality, and social support | Acta Psychologica | Mental health disorders |
| 459 | Chen, Y.;Zhu, L. J.;Fang, Z. M.;Wu, N.;Du, M. X.;Jiang, M. M.;Wang, J.;Yao, Y. S.;Zhou, C. C.; | 2021 | The Association of Suicidal Ideation With Family Characteristics and Social Support of the First Batch of Students Returning to a College During the COVID-19 Epidemic Period: A Cross-Sectional Study in China | Front Psychiatry | Mental health disorders |
| 460 | Lee, J. W.;Park, S. J.;Kim, S.;Chung, U. S.;Han, D. H.; | 2022 | The Association Between Temperament and Characteristics, Smartphone App Use Patterns and Academic Performance of University Students | Journal of Korean Medical Science | Not related |
| 461 | Yin, Y.;Yang, X. J.;Gao, L.;Zhang, S. Y.;Qi, M.;Zhang, L. G.;Tan, Y. L.;Chen, J. X.; | 2021 | The Association Between Social Support, COVID-19 Exposure, and Medical Students' Mental Health | Frontiers in Psychiatry | Not related |
| 462 | Xin, Z. Y.; | 2023 | The association between social support provision, psychological capital, subjective well-being, and sense of indebtedness among undergraduates with low socioeconomic status | BMC Psychology | Not related |

| | | | | | |
|---|---|---|---|---|---|
| 463 | Chen, J.;Wang, S.;Lai, X.;Zou, L.;Wen, S. W.;Krewski, D.;Xie, R. H.; | 2024 | The association between social support and postpartum post-traumatic stress disorder | BMC Pregnancy and Childbirth | Mental health disorders |
| 464 | Corcoran, E.;Bird, M.;Batchelor, R.;Ahmed, N.;Nowland, R.;Pitman, A.; | 2024 | The association between social connectedness and euthanasia and assisted suicide and related constructs: systematic review | BMC Public Health | Mental health disorders |
| 465 | Wang, S.;Eklund, L.;Yang, X.; | 2022 | The Association Between Sexual Harassment and Mental Health Among Chinese College Students: Do Gender and Social Support Matter? | Int J Public Health | Not related |
| 466 | Cao, J.;Liu, Y.;Yan, S.;Xiong, Z.;Wen, J.;Chen, Z.;Zhang, P.;Tao, J.;Zhang, J.;Wang, Y.;Zou, L.;Fu, W.; | 2024 | The association between perceived social support and post-traumatic stress disorder symptoms among medical staff in Hubei, China: a chain mediating effect of resilience and positive coping | BMC Public Health | Mental health disorders |
| 467 | Levy, M.;Yatziv, T.;Levavi, K.;Yakov, P.;Pike, A.;Deater-Deckard, K.;Hadar, A.;Bar, G.;Froimovici, M.;Atzaba-Poria, N.; | 2024 | The association between maternal and child posttraumatic stress symptoms among families living in southern Israel: The buffering role of maternal executive functions | Stress and Health | The population is not college students |
| 468 | Wang, Taotao;Ren, Mengyuan;Shen, Ying;Zhu, Xiaorou;Zhang, Xing;Gao, Min;Chen, Xueying;Zhao, Ai;Shi, Yuhui;Chai, Weizhong;Liu, Xinchuan;Sun, Xinying; | 2019 | The Association Among Social Support, Self-Efficacy, Use of Mobile Apps, and Physical Activity: Structural Equation Models With Mediating Effects | JMIR mHealth and uHealth | Not related |
| 469 | Ashurst, J.;van Woerden, I.;Dunton, G.;Todd, M.;Ohri-Vachaspati, P.;Swan, P.;Bruening, M.; | 2018 | The Association among Emotions and Food Choices in First-Year College Students Using mobile-Ecological Momentary Assessments | BMC Public Health | Not related |

| | | | | | |
|---|---|---|---|---|---|
| 470 | Zhang, T.;Fu, H.;Wan, Y.; | 2014 | The application of group forgiveness intervention for courtship-hurt college students: a Chinese perspective | International journal of group psychotherapy | Mental health disorders |
| 471 | Volk, Fred;Brown, Joshua Travis;Gibson, Daniel J.;Kush, Joseph M.; | 2023 | The Anatomy of Room Change: Architecture, Academic Performance, and Differences in Race and Socioeconomic Status | Journal of College and University Student Housing | Not related |
| 472 | Howland, J.;Rohsenow, D. J.;Arnedt, J. T.;Bliss, C. A.;Hunt, S. K.;Calise, T. V.;Heeren, T.;Winter, M.;Littlefield, C.;Gottlieb, D. J.; | 2011 | The acute effects of caffeinated versus non-caffeinated alcoholic beverages on driving performance and attention/reaction time | Addiction (Abingdon, England) | Mental health disorders |
| 473 | Brooks, C. P.;Hopewell-Kelly, N.;Lewis, N. V.; | 2024 | The Acceptability, Safety, and Impact of a Play Co-Developed With Public Contributors as a Format for Disseminating Research on a Sensitive Subject | Health Expectations | Not related |
| 474 | Yasuike, Akiko; | 2019 | The Academic Success of Undocumented Latino Students: School Programs, Non-Profit Organizations, and Social Capital | Journal of Latinos and Education | Not related |
| 475 | Lee, J.;Xiang, P.;Liu, J.;McBride, R. E.; | 2022 | The 3 × 2 achievement goal model and its relation to students' persistence/effort in college physical activity classes | Journal of American College Health | Not related |
| 476 | Navarro-Mateos, C.;Mora-Gonzalez, J.;Perez-Lopez, I. J.; | 2023 | The "STAR WARS: The First Jedi" Program. Effects of Gamification on Psychological Well-Being of College Students | Games for Health Journal | Not related |
| 477 | Mason, M.;Benotsch, E. G.;Way, T.;Kim, H.;Snipes, D.; | 2014 | Text messaging to increase readiness to change alcohol use in college students | Journal of primary prevention | Mental health disorders |
| 478 | Tahaney, K. D.;Palfai, T. P.; | 2017 | Text messaging as an adjunct to a web-based | Addictive behaviors | Mental health disorders |

| | | | | | |
|---|---|---|---|---|---|
| | | | intervention for college student alcohol use: a preliminary study | | |
| 479 | Eddy, R.;Goetschius, J.;Hertel, J.;Resch, J.; | 2020 | Test-Retest Reliability and the Effects of Exercise on the King-Devick Test | Clinical journal of sport medicine | Not related |
| 480 | Ayres, C.;Mahat, G.;Atkins, R.; | 2013 | Testing theoretical relationships: factors influencing positive health practices (PHP) in Filipino college students | J Am Coll Health | Not related |
| 481 | Gómez-López, C.;Osorio-Cock, L. M.;Yepes-Delgado, C. E.;Palacio-Ortiz, J. D.; | 2024 | Testimonies of Parents with Children Diagnosed with Attention Deficit/Hyperactivity Disorder, the Challenge of Re-meaning Experiences | Revista Colombiana de Psiquiatria | The population is not college students. |
| 482 | Potts, H.;Shaligram, D.;Ashraf, R.;Diekroger, E.;Fogler, J.; | 2024 | Teletherapy and Medication Management of Attention-Deficit/Hyperactivity Disorder with Co-occurring Internalizing Symptoms and Suicidality during the Pandemic | Journal of Developmental and Behavioral Pediatrics | Mental health disorders |
| 483 | Yao, N.;Wang, Q.; | 2023 | Technostress from Smartphone Use and Its Impact on University Students' Sleep Quality and Academic Performance | Asia-Pacific Education Researcher | Mental health disorders |
| 484 | Frenzel, Svenja B.;Junker, Nina M.;Häusser, Jan A.;Erkens, Valerie A.;van Dick, Rolf; | 2023 | Team identification relates to lower burnout—Emotional and instrumental support as two different social cure mechanisms. | British Journal of Social Psychology | Not related |
| 485 | Popa-Velea, O.;Pristavu, C. A.;Ionescu, C. G.;Diaconescu, L. V.;Mihailescu, A. I.; | 2021 | Teaching Style, Coping Strategies, Stress and Social Support: Associations to the Medical Students' Perception of Learning during the SARS-CoV-2 Pandemic | Education Sciences | Mental health disorders |
| 486 | Yeager, D. S.;Walton, G. M.;Brady, S. T.;Akcinar, E. N.;Paunesku, | 2016 | Teaching a lay theory before college narrows achievement gaps at scale | Proc Natl Acad Sci U S A | Not related |

| | | | | | |
|---|---|---|---|---|---|
| | D.;Keane, L.;Kamentz, D.;Ritter, G.;Duckworth, A. L.;Urstein, R.;Gomez, E. M.;Markus, H. R.;Cohen, G. L.;Dweck, C. S.; | | | | |
| 487 | Garcia, S.;Nalven, M.;Ault, A.;Eskenazi, M. A.; | 2020 | tDCS as a treatment for anxiety and related cognitive deficits | International journal of psychophysiology | Mental health disorders |
| 488 | Voogt, C. V.;Poelen, E. A.;Kleinjan, M.;Lemmers, L. A.;Engels, R. C.; | 2011 | Targeting Young Drinkers Online: the effectiveness of a web-based brief alcohol intervention in reducing heavy drinking among college students: study protocol of a two-arm parallel group randomized controlled trial | BMC Public Health | Mental health disorders |
| 489 | Meluch, A. L.;Starcher, S.;Hannah, M.;LeBlanc, S. S.; | 2022 | Talking to Instructors about Mental Health during the COVID-19 Pandemic: Investigating the Associations between College Students' Willingness to Communicate about Mental Health, Perceptions of Social Support Availability, and the Risk of Disclosure | Southern Communication Journal | Not related |
| 490 | Mrazek, A. J.;Mrazek, M. D.;Maul, A.;Mrazek, K. L.;Schooler, J. W.; | 2021 | Taking charge: characterizing the rapid development of self-regulation through intensive training. | Journal of Health Psychology | Not related |
| 491 | Converse, A. K.;Barrett, B. P.;Chewning, B. A.;Wayne, P. M.; | 2020 | Tai Chi training for attention deficit hyperactivity disorder: a feasibility trial in college students | Complementary therapies in medicine | Mental health disorders |
| 492 | Xu, K. R.;Shah, S.;Rohr, B.; | 2024 | Systemic hydro is an aciniform lymphoproliferative disorder in a patient with | BMJ Case Reports | Mental health disorders |

| | | | | | |
|---|---|---|---|---|---|
| | | | chronic active EBV infection | | |
| 493 | Kelly, N. R.;Mazzeo, S. E.;Bean, M. K.; | 2013 | Systematic review of dietary interventions with college students: directions for future research and practice | J Nutr Educ Behav | Mental health disorders |
| 494 | Bao, Y.;Liu, Y.;Wang, C.;Wang, Y.;Yuan, D.;Xu, J.;Zhu, Z.;He, Y.;Liu, J.; | 2022 | Synergistic removal of U(VI) from aqueous solution by TAC material: Adsorption behavior and mechanism | Appl Radiat Isot | Not related |
| 495 | Van der Merwe, E.;Stroud, L.;Sharp, G.;Van Vuuren, N.;Mosola, M.;Fodo, T.;Paruk, F.; | 2024 | Symptoms of anxiety, depression, and post-traumatic stress disorder 6 weeks and 6 months after ICU: Six out of 10 survivors affected | South African medical journal = Suid-Afrikaanse tydskrif vir geneeskunde | Mental health disorders |
| 496 | Sewell, M. N.;Soto, C. J.;Napolitano, C. M.;Yoon, H. J.;Roberts, B. W.; | 2022 | Survey data of social, emotional, and behavioral skills among seven independent samples | Data Brief | Not related |
| 497 | Marek, F.;Oexle, N.; | 2024 | Supportive and non-supportive social experiences following suicide loss: a qualitative study | BMC Public Health | Mental health disorders |
| 498 | Hensley, L.;Kulesza, A.;Peri, J.;Brady, A. C.;Wolters, C. A.;Sovic, D.;Breitenberger, C.; | 2021 | Supporting Undergraduate Biology Students' Academic Success: comparing Two Workshop Interventions | CBE life sciences education | Mental health disorders |
| 499 | Cook, D. A.;Aljamal, Y.;Pankratz, V. S.;Sedlack, R. E.;Farley, D. R.;Brydges, R.; | 2019 | Supporting self-regulation in simulation-based education: a randomized experiment of practice schedules and goals | Advances in health sciences education | Not related |
| 500 | Diamond, R.;Waite, F.;Boylan, A. M.;Hicks, A.;Kabir, T.;Shiers, D.;Freeman, D.; | 2024 | Supporting movement and physical activity in people with psychosis: A qualitative exploration of the carer perspective | International Journal of Social Psychiatry | Not related |

| | | | | | |
|---|---|---|---|---|---|
| 501 | Eveland, Thomas J.; | 2020 | Supporting first-generation college students: analyzing academic and social support's effects on academic performance. | Journal of Further and Higher Education | Not related |
| 502 | Locke, J.;Osuna, A.;Myrvold, R. J.;Closson, J. S.; | 2023 | Supporting Autistic College Students: Examining the Mentoring, Organization and Social Support for Autism Inclusion on Campus (MOSAIC) Program | J Autism Dev Disord | Not related |
| 503 | Washington-Nortey, M.;Angwenyi, V.;Demissie, M.;Mwangome, E.;Eshetu, T.;Negussie, H.;Goldsmith, K.;Healey, A.;Feyasa, M.;Medhin, G.;Belay, A.;Azmeraw, T.;Getachew, M.;Birhane, R.;Nasambu, C.;Kifle, T. H.;Kairu, A.;Mkubwa, B.;Girma, F.;Abdurahman, R.;Tsigeb | 2024 | Supporting African communities to increase resilience and mental health of kids with developmental disabilities and their caregivers using the World Health Organization's Caregiver Skills Training Programme (SPARK trial): study protocol for a cluster rando | Trials | Not related |
| 504 | Pfender, E.;Weir, S.;White, A.; | 2023 | Support marshaling strategies among college students with anxiety and depression. | J Am Coll Health | Mental health disorders |
| 505 | Bellar, D.;Murphy, K.;Davis, G. R.;Judge, L. W.; | 2021 | Supplemental vitamin D3 does not affect musculoskeletal or psychomotor performance in college-aged males. | International journal for vitamin and nutrition research. Internationale Zeitschrift fur Vitamin- und Ernahrungsforschun | Not related |

| | | | | | |
|---|---|---|---|---|---|
| | | | | g. Journal international de vitaminologie et de nutrition | |
| 506 | Aburto-Corona, J.;Aragón-Vargas, L.; | 2016 | Sunscreen Use and Sweat Production in Men and Women | Journal of Athletic Training | Not related |
| 507 | Saito, M.;Klibert, J.;Langhinrichsen-Rohling, J.; | 2013 | Suicide proneness in American and Japanese college students: associations with suicide acceptability and emotional expressivity | Death Stud | Mental health disorders |
| 508 | Testoni, I.;Piol, S.;De Leo, D.; | 2021 | Suicide Prevention: University Students' Narratives on Their Reasons for Living and Dying | Int J Environ Res Public Health | Mental health disorders |
| 509 | Altavini, C. S.;Asciutti, A. P. R.;Santana, G. L.;Solis, A. C. O.;Andrade, L. H.;Oliveira, L. G.;Andrade, A. G.;Gorenstein, C.;Wang, Y. P.; | 2023 | Suicide ideation among Brazilian college students: Relationship with academic factors, mental health, and sexual abuse | J Affect Disord | Mental health disorders |
| 510 | Men, V. Y.;Chan, P. P. M.;Schaffer, A.;Sanchez Morales, D.;Steinberg, R.;Mitchell, R. H.;Sinyor, M.; | 2024 | Suicide by different methods in Toronto: A quantitative study examining 23 23-years of coronial records | Journal of Affective Disorders | Mental health disorders |
| 511 | Gaily-Luoma, S.;Valkonen, J.;Holma, J.;Laitila, A.; | 2024 | Suicide attempt survivors' recovery-related agency in the relational context of services: a qualitative analysis | BMC Public Health | Mental health disorders |
| 512 | Werbart Törnblom, A.;Werbart, A.;Sorjonen, K.;Runeson, B.; | 2024 | Suicide and sudden violent death among young people: Two sides of the same coin? | PLoS ONE | Mental health disorders |
| 513 | Khalil, M. A.;Khalifa, D.;Allam, R. M.;Abdalgeleel, S. A.;Khalaf, O. O.; | 2024 | Suicide and depressive symptoms possible correlates among a sample of Egyptian physicians: | BMC Psychiatry | Mental health disorders |

| | | | | | |
|---|---|---|---|---|---|
| | | | observational cross-sectional study (online survey) | | |
| 514 | Yin, Y.;Tong, J.;Huang, J.;Tian, B.;Chen, S.;Tan, S.;Wang, Z.;Tong, Y.;Fan, F.;Kochunov, P.;Hong, L. E.;Tan, Y.; | 2024 | Suicidality, perceived chronic stress, and stress-induced cortisol changes of individuals with schizophrenia | Stress and Health | Mental health disorders |
| 515 | Johnson, A. M.;Smith, D. L.; | 2022 | Suicidality in Rural College Students: An Integrative Review | J Psychosoc Nurs Ment Health Serv | Mental health disorders |
| 516 | Aizpurua, Eva;Caravaca-Sánchez, Francisco;Taliaferro, Lindsay A.; | 2022 | Suicidality Among College Students in Spain: Prevalence and Associations With Substance Use, Social Support, and Resilience | Death Studies | Mental health disorders |
| 517 | Phalen, P.;Jones, N.;Davis, B.;Sarpal, D.;Dickerson, F.;Vatza, C.;Jumper, M.;Kuczynski, A.;Thompson, E.;Jay, S.;Buchanan, R.;Chengappa, K. N. R.;Goldberg, R.;Kreyenbuhl, J.;Margolis, R.;Dong, F.;Riggs, J.;Moxam, A.;Burris, E.;Campbell, P.;Cooke, A.;Ered, A | 2024 | Suicidality among clients in a network of coordinated specialty care (CSC) programs for first-episode psychosis: Rates, changes in rates, and their predictors | Schizophrenia research | Mental health disorders |
| 518 | Zhao, Q.;Gui, H. Y.;Mao, Y. C.;Tam, C. C.; | 2022 | Suicidal Ideation, Suicide Attempts, Parenting Styles, Social Support and School Conditions among College Students in China | Smith College Studies in Social Work | Mental health disorders |
| 519 | Yu, Y.;Luo, B.;Qin, L.;Gong, H.;Chen, Y.; | 2023 | Suicidal ideation of people living with HIV and its relations to depression, anxiety, and social support | BMC Psychol | Mental health disorders |
| 520 | Huang, Q.;Lin, S.;Li, Y.;Huang, S.;Liao, Z.;Chen, X.;Shao, T.;Li, | 2021 | Suicidal Ideation Is Associated With Excessive Smartphone Use Among Chinese College Students | Front Public Health | Mental health disorders |

| | | | | | |
|---|---|---|---|---|---|
| | Y.;Cai, Y.;Qi, J.;Shen, H.; | | | | |
| 521 | Liu, H. Y.;Wang, W.;Qi, Y. Y.;Zhang, L.; | 2022 | Suicidal ideation among Chinese survivors of childhood sexual abuse: Associations with rumination and perceived social support | Child Abuse & Neglect | The population is not college students |
| 522 | Mahesar, R. A.;Mughal, M.;Abro, S.;Ventriglio, A.;Ali, S. A. E. Z.;Shoib, S.; | 2024 | Suicidal behaviors in Pakistani Urdu television dramas: a 12-year media content analysis | East Asian Archives of Psychiatry | Mental health disorders |
| 523 | Fox, M. L.;James, T. G.;Barnett, S. L.; | 2020 | Suicidal Behaviors and Help-Seeking Attitudes Among Deaf and Hard-of-Hearing College Students | Suicide & life-threatening behavior | Mental health disorders |
| 524 | Brooks, M. A.;Dasgupta, A.;Khadra, M.;Bawaneh, A.;Kaushal, N.;El-Bassel, N.; | 2024 | Suicidal behaviors among refugee women in Jordan: post-traumatic stress disorder, social support and post-displacement stressors | BMC Public Health | Mental health disorders |
| 525 | Abdu, Z.;Hajure, M.;Desalegn, D.; | 2020 | Suicidal Behavior and Associated Factors Among Students in Mettu University, South West Ethiopia, 2019: An Institutional Based Cross-Sectional Study | Psychol Res Behav Manag | Mental health disorders |
| 526 | Eskin, M.;Sakarya, S.;Okyay, P.;Karkın, A. N.;Devrimci Özgüven, H.;Yapici Eser, H.;Abdollahpour Ranjbar, H.;Şar, V.; | 2024 | Suicidal behavior among Turkish physicians: Associations with negative life events, psychological distress, and attitudes towards suicide | Journal of Psychiatric Research | Mental health disorders |
| 527 | Tran, K. K.;Wong, Y. J.;Cokley, K. O.;Brownson, C.;Drum, D.;Awad, G.;Wang, M. C.; | 2015 | Suicidal Asian American College Students' Perceptions of Protective Factors: A Qualitative Study | Death Stud | Mental health disorders |
| 528 | Watts, J. R.;Chowdhury, D.;Holloway, L.; | 2019 | Success in collegiate recovery programs: results from a phenomenological investigation | Alcoholism Treatment | Not related |

| | | | | | |
|---|---|---|---|---|---|
| | | | | Quarterly | |
| 529 | Hyde, C.;Fuelscher, I.;Rosch, K. S.;Seymour, K. E.;Crocetti, D.;Silk, T.;Singh, M.;Mostofsky, S. H.; | 2024 | Subtle motor signs in children with ADHD and their white matter correlates | Human Brain Mapping | Mental health disorders |
| 530 | Denenny, Danielle;Thompson, Elizabeth;Pitts, Steven C.;Dixon, Lisa B.;Schiffman, Jason; | 2015 | Subthreshold psychotic symptom distress, self-stigma, and peer social support among college students with mental health concerns | Psychiatric rehabilitation journal | Mental health disorders |
| 531 | Daou, L.;Rached, A.;Jrad, M.;Naja, W.;Haddad, R.; | 2024 | Substance use disorder in the context of the economic crisis, COVID-19 pandemic and social unrest: a cross-sectional study across Beirut (2020–2021) | Encephale | Mental health disorders |
| 532 | Martínez, L.;Valencia, I.;Trofimoff, V.; | 2020 | Subjective well-being and mental health during the COVID-19 pandemic: Data from three population groups in Colombia | Data Brief | Not related to research questions |
| 533 | Wang, X.;Wang, Y.;Ye, Y.; | 2023 | Subjective socioeconomic status predicts e-learning engagement in college students: the mediating role of perceived social support and self-efficacy. | European Journal of Psychology of Education | Not related |
| 534 | Neef, N. A.;Perrin, C. J.;Haberlin, A. T.;Rodrigues, L. C.; | 2011 | Studying as fun and games: effects on college students' quiz performance | Journal of Applied Behavior Analysis | Not related |
| 535 | Owaki, Y.;Yoshimoto, H.;Saito, G.;Goto, T.;Kushio, S.;Nakamura, A.;Togo, Y.;Mori, K.;Hokazono, H.; | 2022 | Study protocol of brief intervention using gene polymorphism information for excessive drinking among Japanese college students and adults aged 20-30 years: a randomized controlled trial | Trials | The population is not college students |
| 536 | Benjet, C.;Kessler, R. C.;Kazdin, A. E.;Cuijpers, P.;Albor, Y.;Carrasco | 2022 | Study protocol for pragmatic trials of Internet-delivered guided and unguided cognitive | Trials | Mental health disorders |

| | | | | | |
|---|---|---|---|---|---|
| | Tapias, N.;Contreras-Ibanez, C. C.;Duran Gonzalez, M. S.;Gildea, S. M.;Gonzalez, N.;et al.,; | | behavior therapy for treating depression and anxiety in university students of two Latin American countries: the Yo Puedo Sentirme Bien study | | |
| 537 | Pedersen, E. R.;Marshall, G. N.;Schell, T. L.; | 2016 | Study protocol for a web-based personalized normative feedback alcohol intervention for young adult veterans | Addiction science & clinical practice | Mental health disorders |
| 538 | Renn, T.;Griffin, B.;Kumaravelu, V.;Ventuneac, A.;Santacatterina, M.;Bunting, A. M.; | 2024 | Study protocol for a randomized controlled trial to adapt a posttraumatic stress disorder intervention of patients with opioid-stimulant polysubstance use receiving methadone maintenance treatment | BMC Psychiatry | Mental health disorders |
| 539 | Chao, B.;Fan, J.; | 2021 | Study on Mental Health Problems and Countermeasures of College Students under the Background of Epidemic Prevention and Control | Psychiatr Danub | Not related |
| 540 | Orel, O.; | 2024 | Study of effectiveness of NUMO application in lowering ADHD symptoms in adults | BPA Applied Psychology Bulletin | Mental health disorders |
| 541 | Houser, C.;Brannstrom, C.;Quiring, S. M.;Lemmons, K. K.; | 2011 | Study Abroad Field Trip Improves Test Performance through Engagement and New Social Networks | Journal of Geography in Higher Education | Not related |
| 542 | Mutter, A.;Küchler, A. M.;Idrees, A. R.;Kählke, F.;Terhorst, Y.;Baumeister, H.; | 2023 | StudiCare procrastination - Randomized controlled non-inferiority trial of a persuasive design-optimized internet- and mobile-based intervention with digital coach targeting procrastination in college students | BMC Psychology | Mental health disorders |
| 543 | Küchler, A. M.;Schultchen, D.;Pollatos, O.;Moshagen, M.;Ebert, | 2020 | StudiCare mindfulness-study protocol of a randomized controlled trial evaluating an internet- | Trials | Mental health disorders |

| | | | | | |
|---|---|---|---|---|---|
| | D. D.;Baumeister, H.; | | and mobile-based intervention for college students with no and "on demand" guidance | | |
| 544 | Smith, R.;Morgan, J.;Monks, C.; | 2017 | Students' perceptions of the effect of social media ostracism on wellbeing | Computers in Human Behavior | Mental health disorders |
| 545 | Annunziato, R. A.;Tutino, R.;Campagna, B. R.;Duncan-Park, S.;Dunphy, C.;Flood, J.;Riklin, E.;Stevanovic, K.;Mast, M. B.; | 2022 | Students for others: Correlates of adherence to COVID-19 guidelines | J Am Coll Health | Not related |
| 546 | Dangal, M. R.;Bajracharya, L. S.; | 2020 | Students Anxiety Experiences during COVID-19 in Nepal | Kathmandu Univ Med J (KUMJ) | Mental health disorders |
| 547 | Beasley, S. T.; | 2021 | Student-Faculty Interactions and Psychosociocultural Influences as Predictors of Engagement Among Black College Students | Journal of Diversity in Higher Education | Not related |
| 548 | Kumar, S.;Patel, A. B.; | 2024 | Student Suicide in India: Unintended Consequences of Socio-Ecological Factors | Omega (United States) | Mental health disorders |
| 549 | Li, H. M.;Ma, X. Y.;Fang, J.;Liang, G. T.;Lin, R. S.;Liao, W. Y.;Yang, X. S.; | 2023 | Student Stress and Online Shopping Addiction Tendency among College Students in Guangdong Province, China: The Mediating Effect of the Social Support | International journal of environmental research and public health | Mental health disorders |
| 550 | Chaudhry, S.;Tandon, A.;Shinde, S.;Bhattacharya, A.; | 2024 | Student psychological well-being in higher education: The role of internal team environment, institutional, friends and family support and academic engagement | PLoS ONE | Not related |
| 551 | Hoi, Vo Ngoc;Hang, Ho Le; | 2022 | Student Engagement in the Facebook Learning Environment: A Person-Centred Study | Journal of Educational | Not related |

| | | | | | |
|---|---|---|---|---|---|
| | | | | Computing Research | |
| 552 | Alarcon, G. M.;Edwards, J. M.;Menke, L. E.; | 2011 | Student burnout and engagement: a test of the conservation of resources theory | J Psychol | Not related |
| 553 | Bum, C. H.;Jeon, I. K.; | 2016 | Structural relationships between students' social support and self-esteem, depression, and happiness | Social Behavior and Personality | Mental health disorders |
| 554 | Bum, Chul-Ho;Jeon, Ik-Ki; | 2016 | STRUCTURAL RELATIONSHIPS BETWEEN STUDENTS' SOCIAL SUPPORT AND SELF-ESTEEM, DEPRESSION, AND HAPPINESS | Social Behavior and Personality | Mental health disorders |
| 555 | Kalkbrenner, M. T.;Carlisle, K. L.; | 2023 | Structural Pathways Between Social Support and Mental Health Among STEM Students: Implications for College Student Psychotherapy | Journal of College Student Psychotherapy | Not related |
| 556 | Mahoney, C. T.;Dixon, K. E.;Daugherty, Y. T.;Bindbeutel, K. M.;Horne, S. D.;Littleton, H.;Dworkin, E. R.;Livingston, N. A.;Galano, M. M.; | 2024 | Structural Pathways Between PTSD Symptoms and Alcohol Use Consequences Among Women | International Journal of Mental Health and Addiction | Mental health disorders |
| 557 | Bastien, G.;Seifen-Adkins, T.;Johnson, L. R.; | 2018 | Striving for Success: Academic Adjustment of International Students in the U.S | Journal of International Students | Not related |
| 558 | Szkody, E.;Stearns, M.;Stanhope, L.;McKinney, C.; | 2021 | Stress-Buffering Role of Social Support during COVID-19 | Fam Process | Mental health disorders |
| 559 | Farrell, Michelle;Langrehr, Kimberly J.; | 2017 | Stress, Social Support, and Psychosocial Functioning of Ethnically Diverse Students | Journal of College Counseling | Mental health disorders |

| | | | | | |
|---|---|---|---|---|---|
| 560 | Hernández, A. L.;Escobar, S. G.;Fuentes, Nigal;Eguiarte, B. E. B.; | 2019 | Stress, self-efficacy, academic achievement and resilience in emerging adults | Electronic Journal of Research in Educational Psychology | The population is not college students |
| 561 | Kroshus, Emily;Hawrilenko, Matt;Browning, Anne; | 2021 | Stress, self-compassion, and well-being during the transition to college | Social Science & Medicine | Mental health disorders |
| 562 | Gustems-Carnicer, J.;Calderón, C.;Calderón-Garrido, D.; | 2019 | Stress, coping strategies and academic achievement in teacher education students | European Journal of Teacher Education | Mental health disorders |
| 563 | Sontag-Padilla, L.;Dunbar, M. S.;Ye, F.;Kase, C.;Fein, R.;Abelson, S.;Seelam, R.;Stein, B. D.; | 2018 | Strengthening College Students' Mental Health Knowledge, Awareness, and Helping Behaviors: The Impact of Active Minds, a Peer Mental Health Organization | J Am Acad Child Adolesc Psychiatry | Not related |
| 564 | Tucker, R. P.;Haydel, R.;Zielinski, M.;Niederkrotenthaler, T.; | 2022 | Storytelling of suicide attempt recovery and its relationship with mental health treatment-seeking attitudes and behaviors: an experimental study | Journal of American College Health | Mental health disorders |
| 565 | Rijmen, J.;Wiersema, J. R.; | 2024 | Stochastic resonance is not required for pink noise to have beneficial effects on ADHD-related performance. The moderate brain arousal model challenged | Neuropsychologia | Mental health disorders |
| 566 | Tan, S. H.;Pang, J. S.; | 2012 | Sticks and stones will break my bones but failure feedback may not hurt me: gender differences in the relationship between achievement motive, coping strategies, and environmental mastery. | Educational Psychology | Not related |
| 567 | Dilweg, M. A.;Mocking, T. A. M.;Maragkoudakis, P.;van Westen, G. J. P.;Heitman, L. H.;Ijzerman, A. | 2024 | Stereochemical optimization of N,2-substituted cycloalkyl amines as norepinephrine reuptake inhibitors | RSC Medicinal Chemistry | Not related |

| | | | | | |
|---|---|---|---|---|---|
| | P.;Jespers, W.;van der Es, D.; | | | | |
| 568 | Ghetti, C. M.;Hjelmbrekke, S.;Morken, K.;Dahl, T.;Stige, B.; | 2024 | Steering the energy with music: a hermeneutic phenomenological study of user perspectives of music and music therapy for co-occurring ADHD and substance use problems | Substance Abuse: Treatment, Prevention, and Policy | Mental health disorders |
| 569 | Zhang, W.;Zeng, W.;Chen, H.;Liu, J.;Yan, H.;Zhang, K.;Tao, R.;Siok, W. T.;Wang, N.; | 2024 | STANet: A Novel Spatio-Temporal Aggregation Network for Depression Classification with Small and Unbalanced FMRI Data | Tomography | Mental health disorders |
| 570 | Deng, S.;Zhan, X.;Lyu, C.;Saibon, J. B.; | 2023 | Sports motivation in Chinese college students: A systematic review | Heliyon | Not related |
| 571 | Kobrinsky, V.;Siedlecki, K. L.; | 2024 | Specific emotion regulation difficulties mediating the associations between post-traumatic stress disorder symptoms and suicidal behaviors | Current Psychology | Mental health disorders |
| 572 | Bhochhibhoya, A.;Dong, Y.;Branscum, P.; | 2017 | Sources of social support among international college students in the United States | Journal of International Students | Not related |
| 573 | Beauchemin, J. D.; | 2018 | Solution-Focused Wellness: a Randomized Controlled Trial of College Students | Health & social work | Not related |
| 574 | Jerome, L.;Masood, S.;Henden, J.;Bird, V.;Ougrin, D.; | 2024 | Solution-focused approaches for treating self-injurious thoughts and behaviors: a scoping review | BMC Psychiatry | Mental health disorders |
| 575 | Jnaneswar, A.;Jha, K.;Barman, D.;Singh, A.;Pathak, M.;Kumar, G.; | 2020 | Software Intervention in Smoking Cessation among Engineering Students in Bhubaneswar City: a Randomized Controlled Trial | Indian journal of community medicine | Mental health disorders |
| 576 | Huang, L.;Wang, D. S.;Loscalzo, | 2023 | Socioeconomic Status and Students' Mental Health | Behavioral Sciences | Not related |

| | | | | | |
|---|---|---|---|---|---|
| | Y.;Giannini, M.; | | during the COVID-19 University Closure: Mediating Roles of Perceived Social Support and Self-Efficacy | | |
| 577 | Watkins, N. K.;Dubar, R. T.; | 2022 | Socio-demographic factors and COVID-19 experiences predict perceived social support and social media engagement among college students in the U.S. | J Am Coll Health | Not related |
| 578 | Serrano, I. M. A.;Cuyugan, A. M. N.;Cruz, K.;Mahusay, J. M. A.;Alibudbud, R.; | 2023 | Sociodemographic characteristics, social support, and family history as factors of depression, anxiety, and stress among young adult senior high school students in metro Manila, Philippines, during the COVID-19 pandemic | Frontiers in Psychiatry | The population is not college students |
| 579 | Wang, Chao;Shijiao, Yan;Jiang, Heng;Guo, Yingying;Gan, Yong;Lv, Chuanzhu;Lu, Zuxun; | 2022 | Socio-demographic characteristics, lifestyles, social support quality, and mental health in college students: a cross-sectional study | BMC Public Health | Not related |
| 580 | Lent, R. W.;Morris, T. R.;Penn, L. T.;Ireland, G. W.; | 2019 | Social-cognitive predictors of career exploration and decision-making: Longitudinal test of the career self-management model | J Couns Psychol | Not related |
| 581 | Wang, Hua;Chua, Vincent;Stefanone, Michael A.; | 2015 | Social Ties, Communication Channels, and Personal Well-Being: A Study of the Networked Lives of College Students in Singapore: PROD | The American Behavioral Scientist | Not related |
| 582 | Hua, J.;Johnson, A. E.;Pino, S.;Olson, D.;Nguyen, T.;Lawson, L.;Bedolla, B.;Howell, J. L.; | 2022 | Social Threat Reduces Alcohol Consumption among College Students | Alcohol and alcoholism (Oxford, Oxfordshire) | Mental health disorders |
| 583 | Durocher, J. J.;Lufkin, K. M.;King, M. E.;Carter, J. R.; | 2011 | Social technology restriction alters state anxiety but not autonomic activity in humans. | American journal of physiology. | Mental health disorders |

| | | | | | |
|---|---|---|---|---|---|
| | | | | Regulatory, integrative, and comparative physiology | |
| 584 | Gu, Y.;Hu, J.;Hu, Y.;Wang, J.; | 2016 | Social supports and mental health: a cross-sectional study on the correlation of self-consistency and congruence in China | BMC Health Serv Res | Not related |
| 585 | Ullah, M. S.;Akhter, S.;Aziz, M. A.;Islam, M.; | 2023 | Social support: mediating the emotional intelligence-academic stress link | Frontiers in Psychology | Mental health disorders |
| 586 | Murray, Christopher;Lombardi, Allison;Bender, Franklin;Gerdes, Hillary; | 2013 | Social support: main and moderating effects on the relation between financial stress and adjustment among college students with disabilities | Social Psychology of Education: An International Journal | Mental health disorders |
| 587 | Chambi-Martínez, C. A. A.;Moraga-Escobar, E. I.;Peralta-Jiménez, G. A.;Vera-Calzaretta, A.;Barrientos, J.;Pihán, R.;Klaassen, G.;Páez, D.; | 2022 | Social Support, Stress, and Emotional Symptoms Among LGBTQ+ College Students in Chile | International Journal of Sexual Health | Mental health disorders |
| 588 | Chambi-Martínez, C. A. A.;Moraga-Escobar, E. I.;Peralta-Jiménez, G. A.;Vera-Calzaretta, A.;Barrientos, J.;Pihán, R.;Klaassen, G.;Páez, D.; | 2022 | Social Support, Stress, and Emotional Symptoms Among LGBTQ Plus College Students in Chile | International Journal of Sexual Health | Mental health disorders |
| 589 | Yildirim, M.;Tanrýverdi, F. C.; | 2020 | Social Support, Resilience, and Subjective Well-being in College Students | Journal of Positive Psychology and Wellbeing | Not related |

| | | | | | |
|---|---|---|---|---|---|
| 590 | Thompson, Wendy E.; | 2017 | Social Support, Religious Involvement, and Alcohol Use among Students at a Conservative Religious University | Behavioral Sciences | Mental health disorders |
| 591 | Wang, Wei;Zhang, Jie; | 2024 | Social Support, Psychological Strain, and Suicidality: Evidence from Chinese Universities | Psychology in the Schools | Mental health disorders |
| 592 | Wei, Z.; | 2024 | Social support, psychological responses, and mental health among college students during online learning | Interactive Learning Environments | Not related |
| 593 | Wang, Y.;Zhu, J.;Xu, Z. L.;Dai, X. Y.;Chen, K. D.;Wang, Y.; | 2023 | Social Support, Oral Health Knowledge, attitudes, practice, self-efficacy and Oral health-related quality of life in Chinese college students | Scientific Reports | Not related |
| 594 | Hofman, N. L.;Hahn, A. M.;Tirabassi, C. K.;Gaher, R. M.; | 2016 | Social Support, Emotional Intelligence, and Posttraumatic Stress Disorder Symptoms A Mediation Analysis | Journal of Individual Differences | Mental health disorders |
| 595 | Ayres, C. G.;Mahat, G.; | 2012 | Social support, acculturation, and optimism: understanding positive health practices in Asian American college students | J Transcult Nurs | Not related |
| 596 | Turkpour, A.;Mehdinezhad, V.; | 2016 | Social support, academic support and Adaptation to college: Exploring the relationships between indicators of college students | New Educational Review | Not related |
| 597 | Liu, C.;Ma, J.; | 2020 | Social support through online social networking sites and addiction among college students: The mediating roles of fear of missing out and problematic smartphone use | Current Psychology | Mental health disorders |
| 598 | Johnson-Esparza, Y.;Espinosa, P. R.;Verney, S. P.;Boursaw, B.;Smith, | 2021 | Social Support Protects Against Symptoms of Anxiety and Depression: Key Variations in Latinx | J Lat Psychol | Mental health disorders |

| | | | | | |
|---|---|---|---|---|---|
| | B. W.; | | and Non-Latinx White College Students | | |
| 599 | Dworkin, E. R.;Ojalehto, H.;Bedard-Gilligan, M. A.;Cadigan, J. M.;Kaysen, D.; | 2018 | Social support predicts reductions in PTSD symptoms when substances are not used to cope: A longitudinal study of sexual assault survivors. | Journal of Affective Disorders | Mental health disorders |
| 600 | Leite, S. V.;de Freitas Pinho França, L. H.; | 2022 | Social support perceived by the older college students compared to the younger ones | Avaliacao Psicologica | Not related |
| 601 | Restrepo, Danielle Marie;Spokas, Megan; | 2023 | Social support moderates the relationship between interpersonal trauma and suicidal behaviors among college students. | Journal of American College Health | Mental health disorders |
| 602 | Dai, P.;Yi, G.;Qian, D.;Wu, Z.;Fu, M.;Peng, H.; | 2023 | Social Support Mediates the Relationship Between Coping Styles and the Mental Health of Medical Students | Psychol Res Behav Manag | Not related |
| 603 | Liu, J.; | 2021 | Social support mediates the effect of forgiveness on subjective well-being in college students. | Social Behavior and Personality | Not related |
| 604 | McClay, Michael M.;Brausch, Amy M.;O'Connor, Stephen S.; | 2020 | Social Support Mediates the Association between Disclosure of Suicide Attempts and Depression, Perceived Burdensomeness, and Thwarted Belongingness | Suicide & Life-Threatening Behavior | Mental health disorders |
| 605 | Huang, W. L.;Yu, H.; | 2022 | Social support in university music students' coping with performance anxiety: people, strategies and performance situations | Music Education Research | Mental health disorders |
| 606 | Morling, Beth;Uchida, Yukiko;Frentrup, Sandra; | 2015 | Social Support in Two Cultures: Everyday Transactions in the U.S. and Empathic Assurance in Japan | PLoS ONE | Not related |
| 607 | Ncube, B. L.;Shaikh, K. T.;Ames, M. | 2019 | Social Support in Postsecondary Students with | International | Mental health disorders |

| | | | | | |
|---|---|---|---|---|---|
| | E.;McMorris, C. A.;Bebko, J. M.; | | Autism Spectrum Disorder | Journal of Mental Health and Addiction | |
| 608 | Manrique-Millones, D.;Millones-Rivalles, R.;Dominguez-Lara, S.;Pineda-Marín, C.;Manrique-Pino, O.; | 2020 | Social Support in Higher Education: Evidence of Validity and Reliability in the Peruvian Context* | Universitas Psychologica | Not related |
| 609 | Manrique-Millones, D.;Millones-Rivalles, R.;Dominguez-Lara, S.;Pineda-Marín, C.;Manrique-Pino, O.; | 2020 | Social Support in Higher Education: Evidence of Validity and Reliability in the Peruvian Context | Universitas Psychologica | Not related |
| 610 | Reeble, C. J.;Lefler, E. K.;Abu-Ramadan, T.;Bodalski, E. A.;Canu, W. H.; | 2023 | Social Support in College Students with ADHD Symptoms: Quantity Beats Quality in Moderating Impairment | Journal of College Student Psychotherapy | Mental health disorders |
| 611 | Chiu, C. H. M.;Ma, H. W.;Boddez, Y.;Raes, F.;Barry, T. J.; | 2019 | Social support from friends predicts changes in memory specificity following a stressful life event. | Memory | Mental health disorders |
| 612 | Arnoso, A.;Sancho, J.;Elgorriaga, E.;Arnoso, M.; | 2022 | Social Support as an Explanatory Mechanism of the Relationship Between Social Class and Mental Health in University Students: A Structural Mediation Model | Journal of Social Inclusion | Not related |
| 613 | Carmeli, A.;Peng, A. C.;Schaubroeck, J. M.;Amir, I.; | 2021 | Social support as a source of vitality among college students: The moderating role of social self-efficacy | Psychology in the Schools | Not related |
| 614 | Asensio-Martinez, | 2023 | Social support as a mediator in the relationship | Frontiers in | Mental health disorders |

| | | | | | |
|---|---|---|---|---|---|
| | A.;Aguilar-Latorre, A.;Masluk, B.;Gascón-Santos, S.;Sánchez-Calavera, M. A.;Sánchez-Recio, R.; | | between technostress or academic stress and health: analysis by gender among university students | Psychology | |
| 615 | Sullivan, M. D.;Wilson, L.;Amick, M.;Miller-Matero, L. R.;Chrusciel, T.;Salas, J.;Zabel, C.;Lustman, P. J.;Ahmedani, B.;Carpenter, R. W.;Scherrer, J. F.; | 2024 | Social support and the association between post-traumatic stress disorder and risk for long-term prescription opioid use | Pain | Mental health disorders |
| 616 | Baker, C. N.; | 2013 | Social Support and Success in Higher Education: The Influence of On-Campus Support on African American and Latino College Students | Urban Review | Not related |
| 617 | Rahming, S.; | 2019 | Social support and stress-related acculturative experiences of an English-speaking afro-caribbean female student in U.S. higher education | Journal of International Students | Mental health disorders |
| 618 | Fontanini, Humberto;Marshman, Zoe;Vettore, Mario; | 2015 | Social support and social network as intermediary social determinants of dental caries in adolescents | Community Dentistry and Oral Epidemiology | Not related |
| 619 | Ruppel, E. K.;McKinley, C. J.; | 2015 | Social Support and Social Anxiety in Use and Perceptions of Online Mental Health Resources: Exploring Social Compensation and Enhancement | Cyberpsychol Behav Soc Netw | Mental health disorders |
| 620 | Lee, Kyunghee; | 2020 | Social Support and self-esteem on the association between stressful life events and mental health outcomes among college students | Social Work in Health Care | Mental health disorders |
| 621 | Wu, J.;Xiao, Q. X.;Zhang, X.; | 2023 | Social support and self-concept clarity as mediators between college students' passive social | Journal of Psychology in | Not related |

| | | | | | |
|---|---|---|---|---|---|
| | | | network site use and loneliness | Africa | |
| 622 | Ilevbare, F. M.;Ilevbare, O. E.;Adelowo, C. M.;Oshorenua, F. P.; | 2022 | Social support and Risk-taking Propensity as predictors of entrepreneurial intention among undergraduates in Nigeria | Asia Pacific Journal of Innovation and Entrepreneurship | Not related |
| 623 | Huang, Z.;Gan, Q.;Luo, M.;Zhang, Y.;Ge, J.;Fu, Y.;Chen, Z.; | 2023 | Social support and prosocial behavior in Chinese college students during the COVID-19 outbreak: a moderated mediation model of positive affect and parental care | Front Psychol | Not related |
| 624 | Sullivan, L.;Ding, K.;Tattersall, H.;Brown, S.;Yang, J.; | 2022 | Social Support and Post-Injury Depressive and Anxiety Symptoms among College-Student Athletes | International journal of environmental research and public health | Mental health disorders |
| 625 | Gage, Gale S.; | 2017 | Social Support and Positive Health Practices in Black Late Adolescents: The Role of Mediating Variables | Clinical Nursing Research | Not related |
| 626 | Gage, G. S.; | 2017 | Social Support and Positive Health Practices in Black Late Adolescents | Clin Nurs Res | Not related |
| 627 | Kleiman, E. M.;Riskind, J. H.;Schaefer, K. E.; | 2014 | Social Support and Positive Events as Suicide Resiliency Factors: Examination of Synergistic Buffering Effects | Archives of suicide research | Mental health disorders |
| 628 | Schneider, E.;Chesky, K.; | 2011 | Social support and performance anxiety of college music students | Medical problems of performing artists | Mental health disorders |
| 629 | Yalçin, Ilhan; | 2011 | Social Support and Optimism as Predictors of Life Satisfaction of College Students | International Journal for the | Not related |

| | | | | | |
|---|---|---|---|---|---|
| | | | | Advancement of Counselling | |
| 630 | Yu, H.;Zhou, J.; | 2022 | Social support and online self-regulated learning during the COVID-19 pandemic | Asia Pacific Journal of Education | Not related |
| 631 | Kim, Na-Rae;Kim, Haram J.;Lee, Ki-Hak; | 2018 | Social Support and Occupational Engagement among Korean Undergraduates: The Moderating and Mediating Effect of Work Volition | Journal of Career Development | Not related |
| 632 | Na-Rae, Kim;Kim, Haram J.;Ki-Hak, Lee; | 2018 | Social Support and Occupational Engagement Among Korean Undergraduates | Journal of Career Development | Not related |
| 633 | Yang, D.;Liu, L. J.;Wei, Y. L.; | 2023 | Social support and learning burnout in art education students: The mediating role of resilience | Social Behavior and Personality | Not related |
| 634 | Pagán, I.;Fabián, C.;Ríos, J. L.;Betancourt, J.;Cruz, S. Y.;González, A. M.;Rivera-Soto, W. T.; | 2013 | Social Support and its Association with Socio-demographic Characteristics, Dietary Patterns, and Perceived Academic Stress among College Students in Puerto Rico | Puerto Rico Health Sciences Journal | Mental health disorders |
| 635 | Pagán, I.;Fabián, C.;Ríos, J. L.;Betancourt, J.;Cruz, S. Y.;González, A. M.;Rivera-Soto, W. T.; | 2013 | Social Support and its Association with sociodemographic characteristics, dietary patterns, and perceived academic stress among college students in Puerto Rico | P R Health Sci J | Mental health disorders |
| 636 | Lei, H.;Li, S. Y.;Chiu, M. M.;Lu, M. H.; | 2018 | Social support and Internet addiction among mainland Chinese teenagers and young adults: A meta-analysis | Computers in Human Behavior | Meta review |
| 637 | Dunn, L. C.;Alexander, S. M.;Howard, A. L.; | 2022 | Social Support and end-of-semester Depression, burnout, and adjustment in students making the transition to university | Infant and Child Development | Mental health disorders |

| | | | | | |
|---|---|---|---|---|---|
| 638 | Liu, Y.;Hu, J. S.;Liu, J.; | 2022 | Social Support and Depressive Symptoms Among Adolescents During the COVID-19 Pandemic: The Mediating Roles of Loneliness and Meaning in Life | Frontiers in Public Health | Mental health disorders |
| 639 | Liu, X.;Li, H.;Zeng, S.;Luo, X.; | 2023 | Social support and cigarette smoking among homosexual college students in China: a moderated mediation model examining the roles of depression and disclosure | Current Psychology | Mental health disorders |
| 640 | Rodriguez, Stefanie; | 2012 | Social Support and Career Thoughts in College Athletes and Non-Athletes | Professional Counselor | Not related |
| 641 | Bertram, D. M.;Poulakis, M.;Elsasser, B. S.;Kumar, E.; | 2014 | Social Support and Acculturation in Chinese International Students | Journal of Multicultural Counseling and Development | Not related |
| 642 | Lombardi, A.;Murray, C.;Kowitt, J.; | 2016 | Social support and academic success for college students with disabilities: Do relationship types matter? | Journal of Vocational Rehabilitation | Not related |
| 643 | Cai, J.;Lian, R.; | 2021 | Social Support and a Sense of Purpose: The Role of Personal Growth Initiative and Academic Self-Efficacy | Front Psychol | Not related |
| 644 | Bromley, Katherine W.;Murray, Christopher;Rochelle, Jonathan;Lombardi, Allison; | 2021 | Social Support Among College Students With Disabilities: Structural Patterns and Satisfaction | Journal of Student Affairs Research and Practice | Not related |
| 645 | Gawry, M.;Cichon, E.;Kiejna, A.; | 2023 | Social support against depression in young adults group during COVID-19 pandemic | Psychiatria Polska | The population is not college students |
| 646 | Faw, Meara H.;Matter, Michelle M.; | 2023 | Social Support | The International | Not related |

| | | | | | |
|---|---|---|---|---|---|
| | | | | Encyclopedia of Health Communication | |
| 647 | Newhart, S.; | 2023 | Social predictors of psychological well-being and symptoms of college students | J Am Coll Health | Not related |
| 648 | Patterson, M. S.;Gagnon, L. R.;Vukelich, A.;Brown, S. E.;Nelon, J. L.;Prochnow, T.; | 2021 | Social networks, group exercise, and anxiety among college students | J Am Coll Health | Mental health disorders |
| 649 | Fernández-Martínez, E.;Andina-Díaz, E.;Fernández-Peña, R.;García-López, R.;Fulgueiras-Carril, I.;Liébana-Presa, C.; | 2017 | Social Networks, Engagement and Resilience in University Students | International journal of environmental research and public health | Not related |
| 650 | Liu, Yan;Yi, Hongfa; | 2022 | Social networking smartphone applications and emotional health among college students: The moderating role of social support | Science Progress | Not related |
| 651 | Tafesse, Wondwesen; | 2022 | Social networking sites use and college student's academic performance: testing for an inverted U-shaped relationship using automated mobile app usage data: Revista de Universidad y Sociedad del Conocimiento. | International Journal of Educational Technology in Higher Education | Not related |
| 652 | Tafesse, Wondwesen; | 2022 | Social Networking Sites Use and College Students' Academic Performance: Testing for an Inverted U-Shaped Relationship Using Automated Mobile App Usage Data | International Journal of Educational Technology in Higher Education | Not related |

| | | | | | |
|---|---|---|---|---|---|
| 653 | Tuck, Alison B.;Thompson, Renee J.; | 2021 | Social Networking Site Use During the COVID-19 Pandemic and Its Associations With Social and Emotional Well-being in College Students: Survey Study | JMIR formative research | Not related |
| 654 | Doleck, Tenzin;Lajoie, Susanne P.;Bazelais, Paul; | 2019 | Social networking and academic performance: A net benefits perspective | Education and Information Technologies | Not related |
| 655 | Arampatzi, E.;Burger, M. J.;Novik, N.; | 2018 | Social Network Sites, Individual Social Capital, and Happiness | Journal of Happiness Studies | Not related |
| 656 | Hofhuis, J.;Hanke, K.;Rutten, T.; | 2019 | Social network sites and acculturation of international sojourners in the Netherlands: The mediating role of psychological alienation and online social support | International Journal of Intercultural Relations | Not related |
| 657 | Yang, Q.;Xu, Y.;van den Bos, K.; | 2024 | Social network site use and materialistic values: the roles of self-control and self-acceptance. | BMC Psychol | Not related |
| 658 | Reid, A. E.;Carey, K. B.;Merrill, J. E.;Carey, M. P.; | 2015 | Social network influences on initiation and maintenance of reduced drinking among college students. | Journal of consulting and clinical psychology | Not related |
| 659 | Pokhrel, Pallav;Fagan, Pebbles;Cassel, Kevin;Trinidad, Dennis R.;Kaholokula, Joseph Keawe'aimoku;Herzog, Thaddeus A.; | 2016 | Social Network Characteristics, Social Support, and Cigarette Smoking among Asian/Pacific Islander Young Adults | American Journal of Community Psychology | The population is not college students |
| 660 | Courtney, A. L.;Baltiansky, D.;Fang, W. M.;Roshanaei, M.;Aybas, Y. C.;Samuels, N. A.;Wetchler, E.;Wu, | 2023 | Social microclimates and well-being | Emotion | Not related |

| | | | | | |
|---|---|---|---|---|---|
| | Z.;Jackson, M. O.;Zaki, J.; | | | | |
| 661 | Tuck, A. B.;Long, K. A.;Thompson, R. J.; | 2023 | Social media's influence on momentary emotion based on people's initial mood: an experimental design | Cognition & emotion | Not related |
| 662 | Spence, A.;Beasley, K.;Gravenkemper, H.;Hoefler, A.;Ngo, A.;Ortiz, D.;Campisi, J.; | 2020 | Social media use while listening to new material negatively affects short-term memory in college students. | Physiology & behavior | Not related |
| 663 | Lee, H. E.;Cho, J.; | 2019 | Social Media Use and Well-Being in People with Physical Disabilities: Influence of SNS and Online Community Uses on Social Support, Depression, and Psychological Disposition | Health communication | Mental health disorders |
| 664 | Su, X.;Huang, J.; | 2021 | Social media use and college students' academic performance: Student engagement as a mediator | Social Behavior and Personality | Not related |
| 665 | Shafiq, M.;Parveen, K.; | 2023 | Social media usage: Analyzing its effect on academic performance and engagement of higher education students | International Journal of Educational Development | Not related |
| 666 | Qi, C.; | 2019 | Social Media Usage of Students, Role of Tie Strength, and Perceived Task Performance | Journal of Educational Computing Research | Not related |
| 667 | Park, N.;Lee, H.; | 2012 | Social implications of smartphone use: Korean college students' smartphone use and psychological well-being | Cyberpsychol Behav Soc Netw | Not related |
| 668 | Gore, Jonathan S.;Thomas, Jessica;Jones, Stevy;Mahoney, | 2016 | Social Factors That Predict Fear of Academic Success | Educational Review | Not related |

| | | | | | |
|---|---|---|---|---|---|
| | Lauren;Dukes, Kristina;Treadway, Jodi; | | | | |
| 669 | Becerra, E. G. T.;Reynoso-Alcántara, V.; | 2019 | Social engagement as a measurement of cognitive reserve and how it relates to different cognitive skills in college students | Acta Colombiana de Psicologia | Not related |
| 670 | Thomas, J. L.;Bengtson, J. E.;Ghidei, W.;Schreier, M.;Wang, Q.;Luo, X.;Lust, K.;Ahluwalia, J. S.; | 2015 | Social contingencies and college quit and win contest: a qualitative inquiry. | Am J Health Behav | Not related |
| 671 | Bourassa, K. J.;Dennis, P. A.;Patel, P.;Qin, X. J.;Sbarra, D. A.;Hauser, E. R.;Ashley-Koch, A. E.;Program, M. V.;Beckham, J. C.;Kimbrel, N. A.; | 2024 | Social connection and suicidal thoughts and behaviors in the Million Veteran Program cohort | Journal of Psychiatric Research | Mental health disorders |
| 672 | Wu, C. L.;Horng, S. M.; | 2022 | Social Commerce Intention, Social Interaction, and Social Support: Moderating Role of Social Anxiety | Journal of Organizational and End User Computing | Mental health disorders |
| 673 | Lent, R. W.;Miller, M. J.;Smith, P. E.;Watford, B. A.;Lim, R. H.;Hui, K.; | 2016 | Social cognitive predictors of academic persistence and performance in engineering: Applicability across gender and race/ethnicity | Journal of Vocational Behavior | Not related |
| 674 | Han, Seung-hyun;Oh, Eunjung Grace;Kang, Sung; | 2022 | Social Capital Leveraging Knowledge-Sharing Ties and Learning Performance in Higher Education: Evidence from Social Network Analysis in an Engineering Classroom | AERA Open | Not related |
| 675 | Zhou, H. L.;Jiang, H. B.;Zhang, B.;Liang, H. Y.; | 2021 | Social anxiety, maladaptive cognition, mobile phone addiction, and perceived social support: A moderated mediation model | Journal of Psychology in Africa | Mental health disorders |

| | | | | | |
|---|---|---|---|---|---|
| 676 | Potter, C. M.;Galbraith, T.;Jensen, D.;Morrison, A. S.;Heimberg, R. G.; | 2016 | Social anxiety and vulnerability for problematic drinking in college students: the moderating role of post-event processing | Cognitive behavior therapy | Mental health disorders |
| 677 | Porter, E.;Chambless, D. L.; | 2017 | Social Anxiety and Social Support in Romantic Relationships | Behavior therapy | Mental health disorders |
| 678 | Labrague, L. J.;De Los Santos, J. A. A.;Falguera, C. C.; | 2021 | Social and emotional loneliness among college students during the COVID-19 pandemic: The predictive role of coping behaviors, social support, and personal resilience | Perspect Psychiatr Care | Not related |
| 679 | Langer Á, I.;Crockett, M. A.;Bravo-Contreras, M.;Carrillo-Naipayan, C.;Chaura-Marió, M.;Gómez-Curumilla, B.;Henríquez-Pacheco, C.;Vergara, R. C.;Santander, J.;Antúnez, Z.;Baader, T.; | 2022 | Social and Economic Factors Associated With Subthreshold and Major Depressive Episodes in University Students During the COVID-19 Pandemic | Front Public Health | Mental health disorders |
| 680 | Zhao, Y. H.; | 2022 | Social Achievement Goals in Chinese Undergraduates: Associations With Self-Esteem and Symptoms of Social Anxiety and Depression | Frontiers in Psychology | Mental health disorders |
| 681 | Graupensperger, S.;Benson, A. J.;Kilmer, J. R.;Evans, M. B.; | 2020 | Social (Un)distancing: Teammate Interactions, Athletic Identity, and Mental Health of Student-Athletes During the COVID-19 Pandemic | J Adolesc Health | Not related |
| 682 | Dopico-Casal, C.;Montes, C.;Fraga, I.;Vieitez, L.;Padrón, I.;Romero, E.; | 2023 | So far but yet so near: Examining the buffering effect of perceived social support on the psychological impact of Spanish lockdown | Journal of Community Psychology | Not related |

| | | | | | |
|---|---|---|---|---|---|
| 683 | Mussener, U.;Bendtsen, M.;Karlsson, N.;White, I. R.;McCambridge, J.;Bendtsen, P.; | 2015 | SMS-based smoking cessation intervention among university students: study protocol for a randomized controlled trial (NEXit trial) | Trials | Mental health disorders |
| 684 | Aalbers, G.;Hendrickson, A. T.;Vanden Abeele, M. M.;Keijsers, L.; | 2023 | Smartphone-Tracked Digital Markers of Momentary Subjective Stress in College Students: Idiographic Machine Learning Analysis | JMIR Mhealth Uhealth | Mental health disorders |
| 685 | Lapierre, Matthew A.;Zhao, Pengfei; | 2022 | Smartphones and Social Support: Longitudinal Associations Between Smartphone Use and Types of Support | Social Science Computer Review | Not related |
| 686 | Xin, M.;Mo, P. K. H.;Li, J.;Liu, X.;Jiang, H.;Chen, Y.;Ma, L.;Lau, J. T. F.; | 2022 | Smartphone non-users experience disproportionately higher psychological distress than their counterparts: Mediations via psychosocial resources in a large sample of college students in China. | J Affect Disord | Mental health disorders |
| 687 | Bruehlman-Senecal, E.;Hook, C. J.;Pfeifer, J. H.;FitzGerald, C.;Davis, B.;Delucchi, K. L.;Haritatos, J.;Ramo, D. E.; | 2020 | Smartphone app to address loneliness among college students: pilot randomized controlled trial | JMIR mental health | Not related |
| 688 | Gökçearslan, S.;Uluyol, Ç;Sahin, S.; | 2018 | Smartphone addiction, cyberloafing, stress and social support among university students: A path analysis | Children and Youth Services Review | Mental health disorders |
| 689 | Lee, S.;Kim, H. J.;Choi, H. G.;Yoo, Y. S.; | 2018 | Smartphone Addiction and Interpersonal Competence of Nursing Students | Iran J Public Health | Mental health disorders |
| 690 | Guan, S. S. A.;Xie, H.;Boyns, D.; | 2020 | Sleep, stress, or social support?: Exploring the mechanisms that explain the relationship between student recreation center use and well-being | Journal of American College Health | Mental health disorders |

| | | | | | |
|---|---|---|---|---|---|
| 691 | Zhou, S. J.;Wang, L. L.;Yang, R.;Yang, X. J.;Zhang, L. G.;Guo, Z. C.;Chen, J. C.;Wang, J. Q.;Chen, J. X.; | 2020 | Sleep problems among Chinese adolescents and young adults during the coronavirus-2019 pandemic | Sleep Med | The population is not college students |
| 692 | Goodman, M. L.;Lee, M.;Springer, A.;Schick, V.;Vaughan, E.;Markham, C.;Gitari, S.;Mukiri, F.; | 2024 | Sleep disturbance as a precursor to anxiety, depression, and PTSD among rural Kenyans: A cross-lagged panel analysis from a rural Kenyan interventional cohort | Journal of Sleep Research | Mental health disorders |
| 693 | Chen, J.;Liang, J.;Lin, X.;Zhang, Y.;Zhang, Y.;Lu, L.;Shi, J.; | 2017 | Sleep Deprivation Promotes Habitual Control over Goal-Directed Control: behavioral and Neuroimaging Evidence. | Journal of neuroscience | Not related |
| 694 | Chen, Y. M.;Wang, I. L.;Zhou, S.;Tsai, T. Y.;Chiu, Y. S.;Chiu, W. C.; | 2021 | Six weeks of Jilin ginseng root supplementation attenuates drop jump-related muscle injury markers in healthy female college students. | Food & function | Not related |
| 695 | Pasco, D.;Roure, C.; | 2022 | Situational interest impacts college students' physical activity in a design-based bike exergame. | Journal of sport and health science | Not related |
| 696 | Lin, W.;Chen, Q.;Jiang, M.;Tao, J.;Liu, Z.;Zhang, X.;Wu, L.;Xu, S.;Kang, Y.;Zeng, Q.; | 2020 | Sitting or Walking? Analyzing the Neural Emotional Indicators of Urban Green Space Behavior with Mobile EEG | Journal of Urban Health | Not related |
| 697 | Bi, S. S.;Lam, C. B.;Chung, K. K. H.; | 2021 | Sibling relationships and civic engagement: A longitudinal study of Chinese young adults | Int J Psychol | The population is not college students |
| 698 | Latifi, B.;Amini, A.;Motie Nasrabadi, A.; | 2024 | Siamese-based deep neural network for ADHD detection using EEG signal | Computers in Biology and Medicine | Mental health disorders |
| 699 | Zhao, Jingjing;Kong, Feng;Wang, Yonghui; | 2013 | Shyness and Subjective Well-being: The Role of Emotional Intelligence and Social Support | Social Indicators Research | Not related |

| | | | | | |
|---|---|---|---|---|---|
| 700 | Zhao, J. J.;Tan, M. G.;Gao, L.;Wang, Y. H.; | 2019 | Shyness and Loneliness: Contributions of Emotional Intelligence and Social Support | Current Psychology | Not related |
| 701 | Howe, E. G.; | 2024 | Should Providers Engage in Religious Discussions, and If They Should, Then with Whom? | The Journal of Clinical Ethics | Not related |
| 702 | Abraham, A. E.;Busch, C. A.;Brownell, S. E.;Cooper, K. M.; | 2022 | Should I write about mental health on my med school app? Examining medical school admissions committee members' biases regarding mental health conditions | Advances in physiology education | Not related |
| 703 | Preti, A.;Sheehan, D. V.;Coric, V.;Distinto, M.;Pitanti, M.;Vacca, I.;Siddi, A.;Masala, C.;Petretto, D. R.; | 2013 | Sheehan Suicidality Tracking Scale (S-STS): reliability, convergent and discriminative validity in young Italian adults | Compr Psychiatry | The population is not college students |
| 704 | Fahey, L.;Lopez, L. M.; | 2024 | Shared Genetic Links Between Sleep, Neurodevelopmental and Neuropsychiatric Conditions: A Genome-Wide and Pathway-Based Polygenic Score Analysis | Genes, Brain and Behavior | Not related |
| 705 | Halstead, V.;Williams, J. R.;Gonzalez-Guarda, R.; | 2017 | Sexual violence in the college population: a systematic review of disclosure and campus resources and services | J Clin Nurs | Not related |
| 706 | Delaney, E. N.;Williams, C. D.;Jones, S. C. T.;Hood, K. B.;Cage, J.;Coston, B. E.;Hawn, S. E.;Santana, A.;Dick, D. M.; | 2023 | Sexual Victimization and Mental Health Among LGBQ + College Students: Examining Social Support and Trauma-Related Drinking as Mediators | International Journal of Mental Health and Addiction | Not related |
| 707 | Jones, Cassandra A.;Raghavan, Chitra; | 2012 | Sexual Orientation, Social Support Networks, and Dating Violence in an Ethnically Diverse Group of | Journal of Gay & Lesbian Social | Not related |

| | | | | | |
|---|---|---|---|---|---|
| | | | College Students | Services | |
| 708 | Chang, C. J.;Fehling, K. B.;Selby, E. A.; | 2020 | Sexual Minority Status and Psychological Risk for Suicide Attempt: A Serial Multiple Mediation Model of Social Support and Emotion Regulation | Frontiers in Psychiatry | Mental health disorders |
| 709 | Gacusan, C. G. D.;Uy, D. M. S.;Yu, D. A. B.;Hechanova, M. R. M.; | 2021 | Sexual identity management of GLB emerging adults in social support contexts | Journal of GLBT Family Studies | The population is not college students |
| 710 | Stappenbeck, C. A.;Hassija, C. M.;Zimmerman, L.;Kaysen, D.; | 2015 | Sexual assault related distress and drinking: The influence of daily reports of social support and coping control | Addictive behaviors | Mental health disorders |
| 711 | Halbe, E.;Heger, A. S.;Kolf, F.;Hüpen, P.;Bergmann, M.;Harrison, B. J.;Davey, C. G.;Philipsen, A.;Lux, S.; | 2024 | Sex differences in physiological Correlates of effectively driven decision-making behavior in Adult ADHD | BMC Psychiatry | Mental health disorders |
| 712 | Nordin, L.;Bothe, S. K.;Perrin, S.;Rorsman, I.; | 2024 | Severe Cognitive Impairment in Trauma-Affected Refugees—Exploring the Impact of Traumatic Brain Injury | Journal of Clinical Medicine | Mental health disorders |
| 713 | Jiang, M.;Zhao, Y.;Wang, J.;Hua, L.;Chen, Y.;Yao, Y.;Jin, Y.; | 2022 | Serial Multiple Mediation of the Correlation Between Internet Addiction and Depression by Social Support and Sleep Quality of College Students During the COVID-19 Epidemic | Psychiatry Investig | Mental health disorders |
| 714 | Doumit, R.;Afifi, R. A.;Devon, H. A.; | 2015 | Serenity in political uncertainty | Holist Nurs Pract | Not related |
| 715 | Ye, J. W.;Yeung, D. Y.;Liu, E. S. C.;Rochelle, T. L.; | 2019 | Sequential mediating effects of provided and received social support on trait emotional intelligence and subjective happiness: A longitudinal examination in Hong Kong Chinese | International Journal of Psychology | Not related |

| | | | | | |
|---|---|---|---|---|---|
| | | | university students | | |
| 716 | Koeber, Charles S.;Wright, David W.;Dingler, Elizabeth; | 2012 | Self-Service in the Labor Process: Control and Consent in the Performance of "Consumptive Labor" | Humanity & Society | Not related |
| 717 | Carpenter, S. M.;Yap, J.;Patrick, M. E.;Morrell, N.;Dziak, J. J.;Almirall, D.;Yoon, C.;Nahum-Shani, I.; | 2023 | Self-Relevant Appeals to Engage in Self-Monitoring of Alcohol Use: a Microrandomized Trial | Psychology of addictive behaviors | Mental health disorders |
| 718 | May, R. W.;Seibert, G. S.;Sanchez-Gonzalez, M. A.;Fincham, F. D.; | 2019 | Self-regulatory biofeedback training: an intervention to reduce school burnout and improve cardiac functioning in college students | Stress (Amsterdam, Netherlands) | Mental health disorders |
| 719 | Yang, D.;Ge, Y.;Sun, Y.;Collins, P.;Jaeggi, S. M.;Xu, Y.;Shea, Z. M.;Warschauer, M.; | 2024 | Self-regulation and comprehension in shared reading: The moderating effects of verbal interactions and E-book discussion prompts | Child Development | Not related |
| 720 | Masilla, A.;Jacquin, K. M.; | 2015 | Self-monitoring, concern for appropriateness, and social support moderate the relationship between childhood exposure to violence and young adult aggression | American Journal of Forensic Psychology | The population is not college students |
| 721 | Scheithauer, M. C.;Kelley, M. L.; | 2017 | Self-Monitoring by College Students With ADHD: the Impact on Academic Performance | Journal of Attention Disorders | Mental health disorders |
| 722 | Gould, O. N.;Doucette, C.; | 2018 | Self-Management of Adherence to Prescribed Stimulants in College Students With ADD/ADHD | J Atten Disord | Mental health disorders |
| 723 | Huang, X. P.;Li, Z. Q.;Feng, X. M.;Wang, X. C.;Jiang, Z. L.; | 2024 | Self-injury and suicide among people living with HIV/AIDS in China: a systematic review and meta-analysis | BMC Public Health | Mental health disorders |
| 724 | Zukerman, G.;Icht, M.;Zigdon, | 2024 | Self- inefficacy's impact on well-being indices in | Journal of | Not related |

| | | | | | |
|---|---|---|---|---|---|
| | A.;Korn, L.; | | students self-identifying with cluttering characteristics | Communication Disorders | |
| 725 | Cordero, E. D.; | 2011 | Self-esteem, social support, collectivism, and the thin ideal in Latina undergraduates | Body image | Not related |
| 726 | Rosenthal, S. R.;Tobin, A. P.; | 2023 | Self-esteem only goes so far: the moderating effect of social media screen time on self-esteem and depressive symptoms. | Behav Inf Technol | Mental health disorders |
| 727 | Acharya Pandey, R.;Chalise, H. N.; | 2015 | Self-Esteem and Academic Stress among Nursing Students | Kathmandu Univ Med J (KUMJ) | Mental health disorders |
| 728 | Yuan, Shu;Weiser, Dana A.;Fischer, Judith L.; | 2016 | Self-efficacy, parent-child relationships, and academic performance: a comparison of European American and Asian American college students | Social Psychology of Education: An International Journal | The population is not college students |
| 729 | Ghozali, G.;Ningsih, A. S.;Oktaviani, L. W.;Masnina, R.; | 2024 | Self-Efficacy, Achievement Motivation, and Anxiety Among Junior High School Students in Selected Sub-urban Areas of Indonesia | Universal Journal of Public Health | The population is not college students |
| 730 | Thériault, ÉR;Walsh, A.;MacIntyre, P.;O'Brien, C.; | 2023 | Self-efficacy in health among university students: the role of social support and place | Journal of American College Health | Not related |
| 731 | Liu, Y. C.;Hung, Y. Y.; | 2016 | Self-efficacy as the Moderator: Exploring Driving Factors of perceived social support for mainland Chinese students in Taiwan | Computers in Human Behavior | Not related |
| 732 | Marr, Joni;Wilcox, Sara; | 2015 | Self-efficacy and Social Support Mediate the Relationship Between Internal Health Locus of Control and Health Behaviors in College Students | American Journal of Health Education | Not related |

| | | | | | |
|---|---|---|---|---|---|
| 733 | Jia, L.;Wang, X.; | 2024 | Self-Efficacy and Life Satisfaction Mediate the Relationship between Perceived Social Support and Career Exploration among College Students: A Cross-Sectional Study | J Psychol | Not related |
| 734 | Ranjit, Y. S.;Snyder, L. B.;Hamilton, M. A.;Rimal, R. N.; | 2017 | Self-Determination Theory and Risk Behavior in a Collectivistic Society: preventing Reckless Driving in Urban Nepal | Journal of Health Communication | Not related |
| 735 | Lindgren, K. P.;Baldwin, S. A.;Ramirez, J. J.;Olin, C. C.;Peterson, K. P.;Wiers, R. W.;Teachman, B. A.;Norris, J.;Kaysen, D.;Neighbors, C.; | 2019 | Self-control, implicit alcohol associations, and the (lack of) prediction of consumption in an alcohol taste test with college student heavy episodic drinkers | PLoS ONE | Mental health disorders |
| 736 | Thomas, J. J.;Lutes, L.;Smirnova, E.;Das, B. M.;Huzurbazar, S.;Aldrich, L.;Lee, M.; | 2019 | Self-Concept in the Context of Diabetes Prevention: Development of the Lifestyle Health-Related Self-Concept Questionnaire | Am J Health Promot | Not related |
| 737 | Dupasquier, J. R.;Kelly, A. C.;Waring, S. V.;Moscovitch, D. A.; | 2020 | Self-compassionate college women report receiving more social support in the face of distress: Evidence from a daily diary study. | Personality and Individual Differences | Mental health disorders |
| 738 | Barankevich, R.;Loebach, J.; | 2022 | Self-Care and Mental Health Among College Students During the COVID-19 Pandemic: Social and Physical Environment Features of Interactions Which Impact Meaningfulness and Mitigate Loneliness | Front Psychol | Not related |
| 739 | Cheng, Y. H.;Xu, J. P.;Wu, H.; | 2024 | Seismic performance of FRP-repaired RC piers after blast loading | Engineering Structures | Not related |
| 740 | Choi, B.;Kim, H.;Huh-Yoo, J.; | 2021 | Seeking Mental Health Support Among College | JMIR Form Res | Not related |

| | | | | | |
|---|---|---|---|---|---|
| | | | Students in Video-Based Social Media: Content and Statistical Analysis of YouTube Videos | | |
| 741 | Grasso, D. J.;Cohen, L. H.;Moser, J. S.;Hajcak, G.;Foa, E. B.;Simons, R. F.; | 2012 | Seeing the silver lining: potential benefits of trauma exposure in college students | Anxiety Stress Coping | Not related |
| 742 | Rzepka, I.;Kindermann, D.;Friederich, H. C.;Nikendei, C.; | 2024 | Secondary traumatization in refugee care—EMDR intervention for interpreters (STEIN): a study protocol for a quasi-randomized controlled trial | Trials | Mental health disorders |
| 743 | Zamboanga, B. L.;Merrill, J. E.;Olthuis, J. V.;Milroy, J. J.;Sokolovsky, A. W.;Wyrick, D. L.; | 2019 | Secondary effects of my playbook on college athletes' avoidance of drinking games or pregaming as a protective behavior strategy: a multisite randomized controlled study | Social science & medicine (1982) | Not related |
| 744 | To, S.;Messias, E.;Burch, L.;Chibnall, J.; | 2024 | Seasonal variation in suicide: age group and summer effects in the United States (2015–2020) | BMC Psychiatry | Mental health disorders |
| 745 | Tanabe, A.;Masuki, S.;Nemoto, K. I.;Nose, H.; | 2018 | Seasonal influence on adherence to and effects of an interval walking training program on sedentary female college students in Japan | International journal of biometeorology | Not related |
| 746 | Palmer, M.;Fang, Z.;Hollocks, M. J.;Charman, T.;Pickles, A.;Baird, G.;Simonoff, E.; | 2024 | Screening for Attention Deficit Hyperactivity Disorder in Young Autistic Adults: The Diagnostic Accuracy of Three Commonly Used Questionnaires | Journal of Autism and Developmental Disorders | The population is not college students |
| 747 | Rogers, M. R.;Churchill, E. D.;Shahid, M.;Davis, T. O.;Mandojana-Ducot, C.; | 2022 | School psychologists' supporting American Indian students on the path to academic success: a community cultural wealth approach | Journal of Mental Health Training Education and Practice | Not related |
| 748 | Çam, Z.;Deniz, K. Z.;Kurnaz, A.; | 2014 | School Burnout: Testing a Structural Equation | Egitim Ve | Mental health disorders |

| | | | | | |
|---|---|---|---|---|---|
| | | | Model Based on Perceived Social Support, Perfectionism, and Stress Variables | Bilim-Education and Science | |
| 749 | Chui, R. C. F.;Chan, C. K.; | 2017 | School Adjustment, Social Support, and Mental Health of Mainland Chinese College Students in Hong Kong | Journal of College Student Development | Not related |
| 750 | Walsh, C. G.;Wilimitis, D.;Chen, Q.;Wright, A.;Kolli, J.;Robinson, K.;Ripperger, M. A.;Johnson, K. B.;Carrell, D.;Desai, R. J.;Mosholder, A.;Dharmarajan, S.;Adimadhyam, S.;Fabbri, D.;Stojanovic, D.;Matheny, M. E.;Bejan, C. A.; | 2024 | Scalable incident detection via natural language processing and probabilistic language models | Scientific Reports | Not related |
| 751 | Petrofes, C.;Howard, K.;Mayberry, A.;Bitney, C.;Ceballos, N.; | 2022 | Sad-fishing: Understanding a maladaptive social media behavior in college students | J Am Coll Health | Not related |
| 752 | Sedgwick, M.;Brassolotto, J.;Manduca-Barone, A.; | 2024 | Rural healthcare professionals’ participation in Medical Assistance in Dying (MAiD): beyond a binary decision | BMC Palliative Care | Not related |
| 753 | Birmachu, A. M.;Heidelberger, L.;Klem, J.; | 2021 | Rumination and perceived social support from significant others interact to predict eating disorder attitudes and behaviors in university students. | Journal of American College Health | Mental health disorders |
| 754 | Zawadzki, M. J.;Graham, J. E.;Gerin, W.; | 2013 | Rumination and anxiety mediate the effect of loneliness on depressed mood and sleep quality in college students. | Health Psychol | Mental health disorders |
| 755 | McKillip, M. E. M.;Godfrey, K. E.;Rawls, A.; | 2013 | Rules of Engagement: Building a College-Going Culture in an Urban School | Urban Education | Not related |

| | | | | | |
|---|---|---|---|---|---|
| 756 | Chen, H. Y.; | 2018 | Roles of mindfulness and perceived social support in mediating the effect of psychological distress on sleep quality of college students | NeuroQuantology | Mental health disorders |
| 757 | Abu-Kaf, S.;Shahar, G.;Noyman-Veksler, G.;Priel, B.; | 2019 | Role of perceived social support in depressive and somatic symptoms experienced by Bedouin Arab and Jewish Israeli undergraduates | Transcult Psychiatry | Mental health disorders |
| 758 | Ehtemam, H.;Sadeghi Esfahlani, S.;Sanaei, A.;Ghaemi, M. M.;Hajesmaeel-Gohari, S.;Rahimisadegh, R.;Bahaadinbeigy, K.;Ghasemian, F.;Shirvani, H.; | 2024 | Role of machine learning algorithms in suicide risk prediction: a systematic review-meta analysis of clinical studies | BMC Medical Informatics and Decision-Making | Mental health disorders |
| 759 | Daghmash, R. M.;Khanfar, M. S.;Darweesh, R. S.; | 2024 | Risperidone Pellets, Pycnogenol®, and Glucomannan Gummy Formulation for Managing Weight Gain and ADHD in Autistic Children | Pharmaceutics | Mental health disorders |
| 760 | Kennedy, S.;Balderrama-Durbin, C.; | 2021 | Risky Casual Sex and Posttraumatic Stress in College Females: An Examination of Assault History, Self-Esteem, and Social Support | Violence Against Women | Mental health disorders |
| 761 | Yao, J.;Xiao, T.;Hou, S.; | 2021 | Risk perceptions and DUI decisions of drivers in different legal environments: new evidence on differential deterrence from a Chinese sample | Accident; analysis and prevention | Not related |
| 762 | Ren, J.;Zhang, Z.;Mei, Y.;Wang, W.;Sun, Q.;Wang, M.;Hui, Z.; | 2022 | Risk perception of COVID-19 among college students in China: Latent profile analysis | Front Public Health | Not related |
| 763 | Counts, C. J.;John-Henderson, N. A.; | 2020 | Risk in childhood family environments and loneliness in college students: Implications for health | J Am Coll Health | The population is not college students |
| 764 | Li, N.;Li, S.;Fan, L.; | 2021 | Risk Factors of Psychological Disorders After the | J Adolesc Health | Mental health disorders |

| | | | | | |
|---|---|---|---|---|---|
| | | | COVID-19 Outbreak: The Mediating Role of Social Support and Emotional Intelligence | | |
| 765 | Stewart, S. A.;Copeland, A. L.;Cherry, K. E.; | 2023 | Risk Factors for Substance Use across the Lifespan | J Genet Psychol | Not related |
| 766 | Hanyu, T.;Ichikawa, H.;Kano, Y.;Ishikawa, T.;Muneoka, Y.;Hirose, Y.;Miura, K.;Tajima, Y.;Shimada, Y.;Sakata, J.;Wakai, T.; | 2024 | Risk factors for death from other diseases after curative gastrectomy and lymph node dissection for gastric cancer | BMC Surgery | Not related |
| 767 | Zhou, G.;Gou, M.;Gan, Y.;Schwarzer, R.; | 2020 | Risk Awareness, Self-Efficacy, and Social Support Predict Secure Smartphone Usage | Front Psychol | Not related |
| 768 | El Keshky, M. E. S.; | 2024 | Risk and protective factors for suicidal ideation among Saudi adolescents: A network analysis | International Journal of Social Psychiatry | Mental health disorders |
| 769 | Ellis, B. H.;Miller, A. B.;Sideridis, G.;Frounfelker, R.;Miconi, D.;Abdi, S.;Aw-Osman, F.;Rousseau, C.; | 2021 | Risk and Protective Factors Associated With Support of Violent Radicalization: Variations by Geographic Location | Int J Public Health | Not related |
| 770 | Gonzalez, J. C.;Feinberg, D. K.;Stewart, R. W.;Young, J.;Orengo-Aguayo, R.; | 2024 | Risk and protective factors associated with substance use among Puerto Rican youths after Hurricane María: a cross-sectional study | BMC Public Health | Not related |
| 771 | Parker, M.;Duran, B.;Rhew, I.;Magarati, M.;Larimer, M.;Donovan, D.; | 2021 | Risk and Protective Factors Associated with Moderate and Acute Suicidal Ideation among a National Sample of Tribal College and University Students 2015-2016 | J Rural Health | Mental health disorders |
| 772 | Wang, L.;Zhang, X.;Zhang, M.;Wang, L.;Tong, X.;Song, N.;Hou, J.;Xiao, J.;Xiao, H.;Hu, T.; | 2024 | Risk and prediction of job burnout in responding nurses to public health emergencies | BMC Nursing | Not related |

| | | | | | |
|---|---|---|---|---|---|
| 773 | Lin, Y.;Liu, Y.;Fan, W.;Tuunainen, V. K.;Deng, S.; | 2021 | Revisiting the relationship between smartphone use and academic performance: A large-scale study | Computers in Human Behavior | Not related |
| 774 | Pittman, Delishia M.;Quayson, Alicia A.;Cassandra Riedy, Rush;Minges, Melanie L.; | 2022 | Revisiting resilience: Examining the relationships between stress, social support, and drinking behavior among black college students with parental substance use disorder histories. | Journal of Ethnicity in Substance Abuse | Mental health disorders |
| 775 | Ellard, O. B.;Dennison, C.;Tuomainen, H.; | 2023 | Review: Interventions addressing loneliness amongst university students: a systematic review | Child Adolesc Ment Health | Mental health disorders |
| 776 | Lacy, E. S.;Miller, B. H.;Hornback, S. A.;McCann, A. L.;Reuben, J. S.; | 2011 | Retention of underrepresented minority students in dental school: one dental school's story | J Am Coll Dent | Not related |
| 777 | Quintiliani, L. M.;Whiteley, J. A.; | 2016 | Results of a Nutrition and Physical Activity Peer Counseling Intervention among Nontraditional College Students | Journal of Cancer Education | Mental health disorders |
| 778 | Joseph, R. P.;Pekmezi, D.;Dutton, G. R.;Cherrington, A. L.;Kim, Y. I.;Allison, J. J.;Durant, N. H.; | 2016 | Results of a Culturally Adapted Internet-Enhanced Physical Activity Pilot Intervention for Overweight and Obese Young Adult African American Women | J Transcult Nurs | Mental health disorders |
| 779 | McGuire, A. P.;Rodenbaugh, M.;Howard, B. A. N.;Contractor, A. A.; | 2024 | Response Styles to Positive Affect During a Positive Psychology Intervention for Veterans With PTSD and Moral Injury: Preliminary Results From a Moral Elevation Intervention Pilot Trial | Psychological Trauma: Theory, Research, Practice, and Policy | Mental health disorders |
| 780 | Yoo, S. H.;Clark, M. S.;Lemay, E. P., Jr.;Salovey, P.;Monin, J. K.; | 2011 | Responding to partners' expression of anger: the role of communal motivation | Pers Soc Psychol Bull | Not related |
| 781 | Garcia-Williams, A. G.;McGee, R. E.; | 2016 | Responding to a suicidal friend or family member: A qualitative study of college students | Death Stud | Mental health disorders |

| | | | | | |
|---|---|---|---|---|---|
| 782 | Arunkumar, N.;Nagaraj, B.;Ruth Keziah, M.; | 2024 | RESNET34 with Synchrosqueezing Transform for ADHD Disorder Detection Using EEG Signals | Fluctuation and Noise Letters | Mental health disorders |
| 783 | Mak, W. W.;Ng, I. S.;Wong, C. C.; | 2011 | Resilience: enhancing well-being through the positive cognitive triad | J Couns Psychol | Not related |
| 784 | Ye, Z.;Yang, X.;Zeng, C.;Wang, Y.;Shen, Z.;Li, X.;Lin, D.; | 2020 | Resilience, Social Support, and Coping as Mediators between COVID-19-related Stressful Experiences and Acute Stress Disorder among College Students in China | Appl Psychol Health Well-Being | Mental health disorders |
| 785 | Khalid, N. M.; | 2021 | RESILIENCE, PERCEIVED SOCIAL SUPPORT, AND LIFE SATISFACTION AMONG MALAYSIAN COLLEGE STUDENTS | Journal of Nusantara Studies-Jonus | Not in a higher education context |
| 786 | Satici, S. A.;Kayis, A. R.;Satici, B.;Griffiths, M. D.;Can, G.; | 2023 | Resilience, Hope, and Subjective Happiness Among the Turkish Population: Fear of COVID-19 as a Mediator | International Journal of Mental Health and Addiction | Not related |
| 787 | Khailenko, O.;Bacon, A. M.; | 2024 | Resilience, avoidant coping, and post-traumatic stress symptoms among female Ukrainian refugees and internally displaced people | International Journal of Social Psychiatry | Mental health disorders |
| 788 | Shi, J.;Chen, Z.;Yin, F.;Zhao, J.;Zhao, X.;Yao, Y.; | 2016 | Resilience as moderator of the relationship between left-behind experience and mental health of Chinese adolescents | Int J Soc Psychiatry | Not in a higher education context |
| 789 | Wilmshurst, Linda;Peele, Marella;Wilmshurst, Luke; | 2011 | Resilience and Well-Being in College Students with and without a Diagnosis of ADHD | Journal of Attention Disorders | Mental health disorders |
| 790 | Selak, Š;Crnkovič, N.;Šorgo, A.;Gabrovec, B.;Cesar, K.;Žmavc, M.; | 2024 | Resilience and social support as protective factors against suicidal ideation among tertiary students during COVID-19: a cross-sectional study | BMC Public Health | Mental health disorders |

| | | | | | |
|---|---|---|---|---|---|
| 791 | Wang, J.;Wu, Y.;Zhou, J.;Li, S.;She, L.; | 2024 | Resilience and its influencing factors after emergency percutaneous coronary intervention in young and middle-aged patients with first acute myocardial infarction | Scientific Reports | Mental health disorders |
| 792 | Zhang, X. N.;Liu, X.;Mi, Y. Y.;Wang, W.;Xu, H. B.; | 2022 | Resilience and Depressive Symptoms Mediated Pathways from Social Support to Suicidal Ideation Among Undergraduates During the COVID-19 Campus Lockdown in China | Psychology Research and Behavior Management | Mental health disorders |
| 793 | Mathieu, A.;Reignier, J.;Le Gouge, A.;Plantefeve, G.;Mira, J. P.;Argaud, L.;Asfar, P.;Badie, J.;Botoc, N. V.;Bui, H. N.;Chatellier, D.;Chauvelot, L.;Cracco, C.;Darmon, M.;Delbove, A.;Devaquet, J.;Dumont, L. M.;Gontier, O.;Groyer, S.;Hourmant, Y.;Jaber, S.; | 2024 | Resilience after severe critical illness: a prospective, multicentre, observational study (RESIREA) | Critical Care | Not related |
| 794 | Linlin, W.;Wanyu, H.;Yuting, L.;Huimin, Q.;Zhi, L.;Qinchen, J.;Tingting, W.;Fan, W.;Minghao, P.;Wei, Z.; | 2023 | Research on the mechanism of short video information interaction behavior of college students with psychological disorders based on grounded theory | BMC Public Health | Mental health disorders |
| 795 | Yu, J.; | 2022 | Research on the Intervention and Prevention of College Students' Mental Health Crisis From the Perspective of Ideological and Physical Education | Front Public Health | Mental health disorders |
| 796 | Graupensperger, S.;Schultz, N. R.;Lewis, M.;Kilmer, J.;Larimer, M.; | 2022 | Repeated Assessment of Alcohol Use and Perceived Norms Among College Students Who | Journal of studies on alcohol and | Mental health disorders |

| | | | | | |
|---|---|---|---|---|---|
| | | | Drink: comparisons to a Minimal Assessment at 12-Month Follow-Up | drugs | |
| 797 | Mirabito, G.;Verhaeghen, P.; | 2022 | Remote delivery of a Koru Mindfulness intervention for college students during the COVID-19 pandemic | Journal of American College Health | Mental health disorders |
| 798 | Xu, L.;Fields, N. L.;Daniel, K. M.;Cipher, D. J.;Troutman, B. A.; | 2023 | Reminiscence and Digital Storytelling to Improve the Social and Emotional Well-Being of Older Adults With Alzheimer's Disease and Related Dementias: protocol for a Mixed Methods Study Design and a Randomized Controlled Trial | JMIR research protocols | The population is not college students |
| 799 | Lew, B.;Kõlves, K.;Zhang, J.;Zhizhong, W.;Koenig, H. G.;Yip, P. S. F.;Abu Talib, M.;Osman, A.;Siau, C. S.;Chan, C. M. H.; | 2021 | Religious affiliation and suicidality among college students in China: A cross-sectional study across six provinces | PLoS ONE | Mental health disorders |
| 800 | Milevsky, Avidan; | 2017 | Religiosity and social support: A mediational model of adjustment in emerging adults | Mental Health, Religion & Culture | The population is not college students |
| 801 | Reyes, M. E. S.;Davis, R. D.;Chua, Capq;Olaveria, G. L.;Pamintuan, L. J. E.;Serrano, M. K. B.;Tan, Jlec; | 2020 | Relative Importance of Social Support and Social Connectedness as Protective Factors of Suicidal Ideation Among Selected Filipino Late Adolescents | Suicidology Online-Sol | Mental health disorders |
| 802 | Zhang, S. J.;Tian, Y.;Sui, Y.;Zhang, D. H.;Shi, J. R.;Wang, P.;Meng, W. X.;Si, Y. D.; | 2018 | Relationships Between Social Support, Loneliness, and Internet Addiction in Chinese Postsecondary Students: A Longitudinal Cross-Lagged Analysis | Frontiers in Psychology | Mental health disorders |
| 803 | Lee, C. K. K.;Chan, K. N. J.;Wong, S. M. C.;Wong, H. S. G.;Lei, H. C. J.;So, Y. K.;Fung, S. C. V.;Chu, S. | 2024 | Relationships between psychopathological symptoms, pandemic-related stress, perceived social support, and COVID-19 infection history: | Front Psychiatry | Mental health disorders |

| | | | | | |
|---|---|---|---|---|---|
| | T. R.;Chung, K. K. A.;Cheng, P. W. C.;Lo, K. Y. H.;Chan, W. C.;Chang, W. C.; | | network analysis in Chinese college students | | |
| 804 | Mao, Y.;Liu, L.;Lu, Z.;Wang, W.; | 2022 | Relationships between Perceived Discrimination and Suicidal Ideation among Impoverished Chinese College Students: The Mediating Roles of Social Support and Loneliness | Int J Environ Res Public Health | Mental health disorders |
| 805 | Davila, E. P.;Kolodziejczyk, J. K.;Norman, G. J.;Calfas, K.;Huang, J. S.;Rock, C. L.;Griswold, W.;Fowler, J. H.;Marshall, S. J.;Gupta, A.;et al.,; | 2014 | Relationships between depression, gender, and unhealthy weight loss practices among overweight or obese college students | Eating Behaviors | Mental health disorders |
| 806 | Merians, A. N.;Mischel, E.;Frazier, P.;Lust, K.; | 2024 | Relationships between childhood adversity and life functioning in US college students: Risk and resilience | J Am Coll Health | The population is not college students |
| 807 | De Pasquale, C.;Pistorio, M. L.;Sciacca, F.;Hichy, Z.; | 2021 | Relationships Between Anxiety, Perceived Vulnerability to Disease, and Smartphone Use During Coronavirus Disease 2019 Pandemic in a Sample of Italian College Students | Front Psychol | Mental health disorders |
| 808 | Fernández-González, L.;González-Hernández, A.;Trianes-Torres, M. V.; | 2015 | Relationships between academic stress, social support, optimism-pessimism and self-esteem in college students | Electronic Journal of Research in Educational Psychology | Mental health disorders |
| 809 | Hagiwara, G.;Iwatsuki, T.;Isogai, H.;Van Raalte, J. L.;Brewer, B. W.; | 2017 | Relationships among sports helplessness, depression, and social support in American college student-athletes | Journal of Physical Education and Sport | Mental health disorders |

| | | | | | |
|---|---|---|---|---|---|
| 810 | Kwon, S. J.;Kim, Y.;Kwak, Y.; | 2020 | Relationship of sleep quality and attention deficit hyperactivity disorder symptoms with quality of life in college students | J Am Coll Health | Mental health disorders |
| 811 | Wang, P. J.;Xiong, Z.;Yang, H.; | 2018 | Relationship of Mental Health, Social Support, and Coping Styles among Graduate Students: Evidence from Chinese Universities | Iranian Journal of Public Health | Mental health disorders |
| 812 | Horiuchi, S.;Tsuda, A.;Toyoshima, N.;Liu, T.;Iwano, S.;Doi, S.;Sakano, Y.; | 2015 | Relationship of cognitive appraisal and coping with subjective happiness in Japanese college students | Hellenic Journal of Psychology | Mental health disorders |
| 813 | Tariq, H.;Bilqees, U.;Fatima, S.;Mukhtar, S.;Usman, H.;Butt, A. N. A.; | 2023 | Relationship Between Social Support, Social Media Usage, and Psychological well-being among Undergraduates in Different Institutions of Punjab | Annals of King Edward Medical University Lahore Pakistan | Mental health disorders |
| 814 | Jeong, Y.; | 2019 | Relationship between social support perceived by nursing students and stress coping: Focusing on the moderating effect of major satisfaction | International Journal of Innovative Technology and Exploring Engineering | Mental health disorders |
| 815 | Hu, S. Y.;Cai, D.;Zhang, X. C.;Margraf, J.; | 2022 | Relationship between social support and positive mental health: A three-wave longitudinal study on college students | Current Psychology | Mental health disorders |
| 816 | Guo, Y.; | 2017 | Relationship between Social Support and Life Satisfaction of College Students: Resilience As a Mediator and Moderator | Ethics in Progress | Mental health disorders |
| 817 | Zhang, Bingyu;Wang, Hongyue;Mei, | 2023 | Relationship between social support and | Social Behavior and | Mental health disorders |

| | | | | | |
|---|---|---|---|---|---|
| | Rong; | | employment anxiety among art education students: Self-efficacy as a mediator | Personality | |
| 818 | Ma, J.;Xiao, Q.; | 2024 | Relationship Between Self-Compassion and Compassion for Others: The Mediated Effect of Perceived Social Support and Psychological Resilience | Psychol Rep | Mental health disorders |
| 819 | Chen, G.;Zhang, G.;Yang, Y.;Zhang, J.;Hu, Y.; | 2023 | Relationship Between Negative Life Events and Depressive Symptoms for Chinese College Students: The Mediating Role of Rumination and Moderating Role of Perceived Social Support and Psychological Capital | Psychology Research and Behavior Management | Mental health disorders |
| 820 | Yang, L.;Yang, Z.;Xia, Y.; | 2022 | Relationship between negative coping style and fear of COVID-19 among Wuhan college students during the post-pandemic period: A moderated chain mediation model | Front Psychiatry | Mental health disorders |
| 821 | Fang, S.;Huang, M.; | 2023 | Relationship between Moral Elevation and Prosocial Behavior among College Students: The Mediating Role of Perceived Social Support and Moderating Role of Moral Identity | International Journal of Mental Health Promotion | Mental health disorders |
| 822 | Newman, J.;Gozu, H.;Guan, S. Y.;Lee, J. E.;Li, X.;Sasaki, Y.; | 2015 | Relationship between Maternal Parenting Style and High School Achievement and Self-Esteem in China, Turkey, and USA | Journal of Comparative Family Studies | The population is not college students |
| 823 | Liu, J.;Cheng, X.;Li, J.; | 2022 | Relationship Between Hardiness and the Mental Health of Funded Chinese College Students: The Mediating Role of Social Support and the Moderating Role of an Only-Child Status | Front Psychol | The population is not college students |

| | | | | | |
|---|---|---|---|---|---|
| 824 | Cheng, X.;Liu, J.;Li, J.;Hu, Z.; | 2022 | Relationship Between Hardiness and Social Anxiety in Chinese Impoverished College Students During the COVID-19 Pandemic: Moderation by Perceived Social Support and Gender | Front Psychol | Mental health disorders |
| 825 | Dager, A. D.;Tice, M. R.;Book, G. A.;Tennen, H.;Raskin, S. A.;Austad, C. S.;Wood, R. M.;Fallahi, C. R.;Hawkins, K. A.;Pearlson, G. D.; | 2018 | Relationship between fMRI response during a nonverbal memory task and marijuana use in college students | Drug and alcohol dependence | Mental health disorders |
| 826 | Wang, T.;Li, S.; | 2022 | Relationship between employment values and college students' choice intention of slow employment: A moderated mediation model | Front Psychol | Mental health disorders |
| 827 | Tang, Y.;He, W.; | 2023 | Relationship between emotional intelligence and learning motivation among college students during the COVID-19 pandemic: A serial mediation model | Front Psychol | Mental health disorders |
| 828 | Hui, Z.;Guo, K. L.;Huang, W. B.;Wu, J. S.;Ma, X. H.;Jia, S. B.;Xing, Z.; | 2023 | Relationship between college students' exercise motivation and mental health: Chain mediating effect of perceived social support and resilience | Social Behavior and Personality | Mental health disorders |
| 829 | Liu, D.;Zhang, M.;Ding, L.;Huang, J.;Wang, Y.;Su, Y.;Chen, Z.;Cai, Y.;He, S.;Peng, D.; | 2024 | Relationship between biological rhythm dysregulation and suicidal ideation in patients with major depressive disorder | BMC Psychiatry | Mental health disorders |
| 830 | Li, X.;Wu, H.;Meng, F.;Li, L.;Wang, Y.;Zhou, M.; | 2020 | Relations of COVID-19-Related Stressors and Social Support With Chinese College Students' Psychological Response During the COVID-19 Pandemic | Front Psychiatry | Mental health disorders |
| 831 | Li, Yaoshan I.;Hazler, Richard | 2017 | Relational Self-Construal as a Moderator of Social | The Career | Mental health disorders |

| | | | | | |
|---|---|---|---|---|---|
| | J.;Trusty, Jerry; | | Support in Career Decision Making | Development Quarterly | |
| 832 | Butz, K. H. G.;Mueller, S. M.;Spille, J. L.;Martin, S.;Grunwald, M.; | 2024 | Refraining from spontaneous face touch is linked to personality traits, reduced memory performance, and EEG changes. | Scientific Reports | Mental health disorders |
| 833 | Ophir, Y.; | 2024 | REEVALUATING ADHD AND ITS FIRST-LINE TREATMENT: INSIGHTS FROM DSM-5-TR AND MODERN APPROACHES | Clinical Neuropsychiatry | Mental health disorders |
| 834 | Umucu, E.;Lee, B.;Berwick, A.;O'Neill, L. E.;Chan, F.;Chen, X. L.; | 2023 | Reducing the Influence of Perceived Stress on Subjective Well-Being of Student Veterans With and Without Disabilities: The Protective Role of Positive Traits and Social Support | Rehabilitation Counseling Bulletin | Mental health disorders |
| 835 | Bluth, K.;Bryce, A.;Lathren, C. R.;Park, J.;Pflum, S.;Clayton, M.; | 2024 | Reducing Suicide Ideation in Transgender Adolescents with Mindful Self-Compassion: An Open Trial | Mindfulness | Mental health disorders |
| 836 | Stormshak, E.;Caruthers, A.;Chronister, K.;DeGarmo, D.;Stapleton, J.;Falkenstein, C.;DeVargas, E.;Nash, W.; | 2019 | Reducing Risk Behavior with Family-Centered Prevention During the Young Adult Years | Prevention science | Mental health disorders |
| 837 | Broda, M.;Yun, J.;Schneider, B.;Yeager, D. S.;Walton, G. M.;Diemer, M.; | 2018 | Reducing Inequality in Academic Success for Incoming College Students: a Randomized Trial of Growth Mindset and Belonging Interventions | Journal of research on educational effectiveness | Mental health disorders |
| 838 | Gilmore, A. K.;Bountress, K. E.; | 2016 | Reducing drinking to cope among heavy episodic drinking college women: secondary outcomes of a web-based combined alcohol use and sexual assault risk reduction intervention | Addictive behaviors | Mental health disorders |

| | | | | | |
|---|---|---|---|---|---|
| 839 | Mautone, J. A.;Holdaway, A.;Chan, W.;Michel, J. J.;Guevara, J. P.;Davis, A.;Desrochers, C.;Evans, E.;Gajary, Z.;Leavy, S.;Rios, D.;Tremont, K. L.;Cacia, J.;Schwartz, B. S.;Jawad, A. F.;Power, T. J.; | 2024 | Reducing disparities in behavioral health treatment in pediatric primary care: a randomized controlled trial comparing Partnering to Achieve School Success (PASS) to usual ADHD care for children ages 5 to 11 – study protocol | BMC Primary Care | Mental health disorders |
| 840 | Sladek, M. R.;Doane, L. D.;Luecken, L. J.;Gonzales, N. A.;Grimm, K. J.; | 2020 | Reducing cultural mismatch: Latino students' neuroendocrine and affective stress responses following cultural diversity and inclusion reminder | Hormones and behavior | Mental health disorders |
| 841 | Dunn, M. E.;Fried-Somerstein, A.;Flori, J. N.;Hall, T. V.;Dvorak, R. D.; | 2020 | Reducing alcohol use in mandated college students: a comparison of a Brief Motivational Intervention (BMI) and the Expectancy Challenge Alcohol Literacy Curriculum (ECALC) | Experimental and clinical psychopharmacology | Mental health disorders |
| 842 | Brooks, S. K.;Greenberg, N.; | 2024 | Recurrence of post-traumatic stress disorder: a systematic review of definitions, prevalence, and predictors | BMC Psychiatry | Mental health disorders |
| 843 | Hennessy, E. A.;Tanner-Smith, E. E.;Finch, A. J.;Sathe, N.;Kugley, S.; | 2018 | Recovery Schools for improving behavioral and academic outcomes among students in recovery from substance use disorders: a systematic review | Campbell Syst Rev | Mental health disorders |
| 844 | Gartner, S. P.;Olesen, T. B.;Jensen, H.;Mortensen, L. M.;Baandrup, L.; | 2024 | Recognition of schizophrenia and quality of treatment during the COVID-19 pandemic: A Danish nationwide study | Schizophrenia research | Mental health disorders |
| 845 | Fernández-Sevillano, J.;González-Ortega, I.;Zorrilla, I.;López, M. P.;Courtet, P.;Leza, J. C.;González-Pinto, A.; | 2024 | Recent suicide: Inflammation and attention | Psiquiatria Biologica | Mental health disorders |

| | | | | | |
|---|---|---|---|---|---|
| 846 | Wang, Mei-Chuan;Wong, Y. Joel;Tran, Kimberly K.;Nyutu, Pius N.;Spears, Angela; | 2013 | Reasons for Living, Social Support, and Afrocentric Worldview: Assessing Buffering Factors Related to Black Americans' Suicidal Behavior | Archives of suicide research | Mental health disorders |
| 847 | Jamieson, J. P.;Black, A. E.;Pelaia, L. E.;Gravelding, H.;Gordils, J.;Reis, H. T.; | 2022 | Reappraising stress arousal improves affective, neuroendocrine, and academic performance outcomes in community college classrooms. | Journal of experimental psychology. General | Mental health disorders |
| 848 | Stamatis, C. A.;Heusser, A. C.;Simon, T. J.;Ala'ilima, T.;Kollins, S. H.; | 2024 | Real-time cognitive performance metrics derived from a digital therapeutic for inattention predict ADHD-related clinical outcomes: Replication across three independent trials of AKL-T01. | Translational Psychiatry | Mental health disorders |
| 849 | Berman, Arielle;Bevan, Jennifer L.;Sparks, Lisa; | 2020 | Readiness to Visit University Counseling Centers: Social Support, Stigma, and Communication Efficacy | Journal of Student Affairs Research and Practice | Mental health disorders |
| 850 | Abouelnaga, K. H.;Huff, A. E.;Jardine, K. H.;O'Neill, O. S.;Winters, B. D.; | 2024 | Reactivation-dependent transfer of fear memory between contexts requires M1 muscarinic receptor stimulation in the dorsal hippocampus of male rats. | Learning and Memory | Mental health disorders |
| 851 | Cooley, E.;Toray, T.;Roscoe, L.; | 2010 | Reactions to Loss Scale: assessing grief in college students | Omega (Westport) | Published not from January 2010 to 31 March 2024 |
| 852 | Pelizza, L.;Leuci, E.;Quattrone, E.;Azzali, S.;Pupo, S.;Paulillo, G.;Pellegrini, P.;Menchetti, M.; | 2024 | Rates and predictors of service disengagement in first episode psychosis: Results from a 2-year follow-up study in an Italian real-world care setting | Schizophrenia research | Not in the higher education context |
| 853 | Edwards, K. M.;Lim, S.;Huff, M.;Herrington, R.;Leader Charge, | 2023 | Rates and Correlates of Intimate Partner Violence Among Indigenous College Students: A | J Interpers Violence | Mental health disorders |

| | | | | | |
|---|---|---|---|---|---|
| | L.;Littleton, H.; | | Multi-Campus Study | | |
| 854 | Lau, E. Y.;Wong, M. L.;Lau, K. N.;Hui, F. W.;Tseng, C. H.; | 2015 | Rapid-Eye-Movement-Sleep (REM) Associated Enhancement of Working Memory Performance after a Daytime Nap | PLoS ONE | Mental health disorders |
| 855 | Woods, K. J. P.;Sampaio, G.;James, T.;Przysinda, E.;Hewett, A.;Spencer, A. E.;Morillon, B.;Loui, P.; | 2024 | Rapid modulation in music supports attention in listeners with attentional difficulties. | Communications Biology | Mental health disorders |
| 856 | Fernandez, A. C.;Wood, M. D.;Laforge, R.;Black, J. T.; | 2011 | Randomized Trials of alcohol-use interventions with college students and their parents: lessons from the Transitions Project | Clinical trials (London, England) | Mental health disorders |
| 857 | Pendry, P.;Carr, A. M.;Gee, N. R.;Vandagriff, J. L.; | 2020 | Randomized Trial Examining Effects of Animal-Assisted Intervention and Stress-Related Symptoms on College Students' Learning and Study Skills | International journal of environmental research and public health | Mental health disorders |
| 858 | Zhang, Y.;Yang, X. X.;Luo, J. Y.;Liang, M.;Li, N.;Tao, Q.;Ma, L. J.;Li, X. M.; | 2022 | Randomized trial estimating effects of hypnosis versus progressive muscle relaxation on medical students' test anxiety and attentional bias | World journal of psychiatry | Mental health disorders |
| 859 | Frazier, P.;Liu, Y.;Selvey, A.;Meredith, L.;Nguyen-Feng, V. N.; | 2023 | Randomized controlled trials assessing the efficacy of brief web-based stress management interventions for college students during the COVID pandemic | Journal of Counseling Psychology | Mental health disorders |
| 860 | Goodness, P. A.;Svingos, A. M.;Gerish, S.;Park, A.;Gellis, L. A.; | 2022 | Randomized controlled trial of cognitive refocusing versus stimulus control treatment for college insomnia: feasibility of a brief, electronic-based, and peer-led approach | Journal of American College Health | Not mental health or wellbeing outcomes |

| | | | | | |
|---|---|---|---|---|---|
| 861 | Hill, R. M.;Picou, P.;Hussain, Z.;Vieyra, B. A.;Perkins, K. M.; | 2024 | Randomized Controlled Trial of an Online Suicide Prevention Gatekeeper Training Program | Crisis | Mental health disorders |
| 862 | Lewis, M. A.;Patrick, M. E.;Litt, D. M.;Atkins, D. C.;Kim, T.;Blayney, J. A.;Norris, J.;George, W. H.;Larimer, M. E.; | 2014 | Randomized controlled trial of a web-delivered personalized normative feedback intervention to reduce alcohol-related risky sexual behavior among college students | Journal of consulting and clinical psychology | Mental health disorders |
| 863 | Frazier, P.;Meredith, L.;Greer, C.;Paulsen, J. A.;Howard, K.;Dietz, L. R.;Qin, K.; | 2015 | Randomized controlled trial evaluating the effectiveness of a web-based stress management program among community college students | Anxiety, stress, and coping | Mental health disorders |
| 864 | Rizvi, S. L.;Finkelstein, J.;Wacha-Montes, A.;Yeager, A. L.;Ruork, A. K.;Yin, Q.;Kellerman, J.;Kim, J. S.;Stern, M.;Oshin, L. A.;et al.,; | 2022 | Randomized clinical trial of a brief, scalable intervention for mental health sequelae in college students during the COVID-19 pandemic | Behavior research and therapy | Mental health disorders |
| 865 | Almeida, M. N.;Alper, D. P.;Barrero, C.;Parikh, N.;Hauc, S. C.;Moscarelli, J.;Golinko, M.;Persing, J.;Swanson, J.;Alperovich, M.; | 2024 | Radiographic severity is associated with worse executive function in metopic craniosynostosis. | Child's Nervous System | Mental health disorders |
| 866 | Baker, C. N.;Robnett, B.; | 2012 | Race, social support and college student retention: A case study | Journal of College Student Development | Not mental health or wellbeing outcomes |
| 867 | Kim, N. E.;Cho, S. M.; | 2012 | Quality of Life of Medical Students during Clinical Clerkship | Korean J Med Educ | Mental health disorders |
| 868 | Chen, C. C. J. J.;Lim, S.;Clardy, G. A.; | 2023 | Qualitative exploration of physical activity engagement among college students with intellectual disabilities | International Journal of Developmental | Mental health disorders |

| | | | | | |
|---|---|---|---|---|---|
| | | | | Disabilities | |
| 869 | Zhang, M. X.;Wang, X. R.;Yu, S. M.;Wu, A. M. S.; | 2019 | Purpose in life, social support, and internet gaming disorder among Chinese university students: A 1-year follow-up study | Addictive behaviors | Mental health disorders |
| 870 | Bianchini, V.;Roncone, R.;Giusti, L.;Casacchia, M.;Cifone, M. G.;Pollice, R.; | 2015 | PTSD Growth and Substance Abuse Among a College Student Community: Coping Strategies After 2009 L'Aquila Earthquake | Clin Pract Epidemiol Ment Health | Mental health disorders |
| 871 | Ducharme, L.;Lo, C.;Hier, M.;Zeitouni, A.;Kost, K.;Mlynarek, A.;Antoni, M.;Kuhn, E.;Owen, J. E.;Heyland, D.;Platt, R.;Fuehrmann, F.;Sadeghi, N.;Rosberger, Z.;Frenkiel, S.;Sultanem, K.;Shenouda, G.;Cury, F.;Henry, M.; | 2024 | PTSD Coach as an Early Mobile Intervention to improve cancer-related anxiety and psychosocial oncology uptake in Patients newly diagnosed with Head and neck cancer: pilot randomized controlled trial | Pilot and Feasibility Studies | Mental health disorders |
| 872 | Wang, D.;Zhou, L.;Chen, C.;Sun, M.; | 2023 | Psychotic-like experiences during COVID-19 lockdown among adolescents: Prevalence, risk and protective factors | Schizophr Res | Mental health disorders |
| 873 | Qeadan, F.;Ross, S.;Barbeau, W. A.;Madden, E. F.;Venner, K. L.;English, K.; | 2023 | Psychostimulant Misuse Among American Indian, Alaskan Native, or Native Hawaiian College Students in the U.S. From 2015 to 2019 | Subst Abuse | Mental health disorders |
| 874 | Liang, W.;Duan, Y.;Wang, Y.;Lippke, S.;Shang, B.;Lin, Z.;Wulff, H.;Baker, J. S.; | 2022 | Psychosocial Mediators of Web-Based Interventions for Promoting a Healthy Lifestyle Among Chinese College Students: Secondary Analysis of a Randomized Controlled Trial | Journal of medical Internet research | Mental health disorders |
| 875 | Johnston, J. D.;Roberts, E. I.;Brenman, A.;Lockard, A. | 2023 | Psychosocial functioning and adjustment to university settings: comparing students with | J Am Coll Health | Mental health disorders |

| | | | | | |
|---|---|---|---|---|---|
| | M.;Baker, A. M.; | | chronic illnesses to healthy peers | | |
| 876 | Ruan, S.;Wang, X.;Zhao, C.;Li, Q.;Li, W. M.;Zhang, G.;Pan, J.;Yang, X.; | 2024 | Psychosocial Correlates of Motivation for Abstinence Among People Who Used Drugs After Community Rehabilitation Treatment in China: A Structural Equation Modelling | Psychol Res Behav Manag | Mental health disorders |
| 877 | Li, X.;Ding, C.;Li, G.;Duan, Z.; | 2024 | Psychosocial characteristics pattern correlated with suicidal ideation and non-suicidal self-injury among nurse staff: a latent profile analysis. | BMC Nursing | Mental health disorders |
| 878 | Okado, Y.;Scaramella, C.;Nguyen, H. M.;Mendoza, B.;Watarastaporn, T.; | 2023 | Psychosocial adjustment of U.S. college students in the early months of the COVID-19 pandemic | J Am Coll Health | Mental health disorders |
| 879 | Zeng, C.;Lin, W.;Chen, Q.; | 2023 | Psychophysiological Responses of College Students to Audio-Visual Forest Trail Landscapes | Journal of Urban Health | Mental health disorders |
| 880 | Palmer, M.;Fang, Z.;Carter Leno, V.;Simonoff, E.; | 2024 | Psychometric properties of two ADHD rating scales used in children with ADHD and intellectual disability | Journal of Intellectual Disability Research | Mental health disorders |
| 881 | Chi, L. C.;Tang, T. C.;Tang, E.; | 2023 | Psychometric properties of the Utrecht Work Engagement Scale for Students (UWES-S) in the Taiwanese context | Current Psychology | Mental health disorders |
| 882 | Cénat, J. M.;Hébert, M.;Karray, A.;Derivois, D.; | 2018 | Psychometric properties of the Resilience Scale - 14 in a sample of college students from France | Encephale | Mental health disorders |
| 883 | Matel-Anderson, D. M.;Bekhet, A. K.; | 2019 | Psychometric properties of the positive thinking skills scale among college students | Arch Psychiatr Nurs | Mental health disorders |
| 884 | Ermis-Demirtas, Hulya;Watson, Joshua C.;Karaman, Mehmet | 2018 | Psychometric Properties of the Multidimensional Scale of Perceived Social Support within Hispanic | Hispanic Journal of Behavioral Sciences | Mental health disorders |

| | | | | | |
|---|---|---|---|---|---|
| | A.;Freeman, Paula;Kumaran, Ajitha;Haktanir, Abdulkadir;Streeter, Ashley M.; | | College Students | | |
| 885 | Shi, Z.;Chen, H.;Guan, J.;Xie, Y.;Huang, S.;Chen, X.;Liu, B.;Shu, Y.; | 2021 | Psychometric properties of the metacognitions about smartphone use questionnaire (MSUQ) in Chinese college students | Addictive behaviors | Meta review |
| 886 | Hallgren, K. A.;Ladd, B. O.;Greenfield, B. L.; | 2013 | Psychometric properties of the Important People Instrument with college student drinkers | Psychol Addict Behav | Mental health disorders |
| 887 | Chen, C.;Zhang, S.;Hong, H.;Qiu, S.;Zhou, Y.;Zhao, M.;Pan, M.;Si, F.;Dong, M.;Li, H.;Wang, Y.;Liu, L.;Sonuga-Barke, E. J. S.;Qian, Q.; | 2024 | Psychometric properties of the Chinese version of the Quick Delay Questionnaire (C-QDQ) and ecological characteristics of reward-delay impulsivity of adults with ADHD | BMC Psychiatry | Mental health disorders |
| 888 | Li, S.;Schulte, E. M.;Cui, G.;Li, Z.;Cheng, Z.;Xu, H.; | 2022 | Psychometric properties of the Chinese version of the modified Yale Food Addiction Scale version 2.0 (C-mYFAS 2.0): Prevalence of food addiction and relationship with resilience and social support | Eat Weight Disord | Mental health disorders |
| 889 | Bremner, J. D.;Williamson, D.;Vaccarino, V.; | 2024 | Psychometric properties of the 23-Item Clinician-Administered Dissociative States Scale (CADSS) in a psychological trauma population | Journal of Affective Disorders | Mental health disorders |
| 890 | Search, F.;Davies, A.;Hennessy, E.; | 2022 | Psychometric evaluation of the Multi-Dimensional Scale of Perceived Social Support in young adults with chronic health conditions | Irish Journal of Psychological Medicine | The population is not college students |
| 891 | Ho, Yi Ming;Prihadi, Kususanto Ditto;Chan, Po Yi;Ahsan Kaz, Kazi Sumaiya;Velayutam, Hirosharani; | 2023 | Psychological Wellbeing of Students with Trait Narcissism | Journal of Education and Learning (EduLearn) | Mental health disorders |

| | | | | | |
|---|---|---|---|---|---|
| 892 | McNally, Rachel D. S.;Winterowd, Carrie L.;Farra, Aisha; | 2021 | Psychological Sense of Community, Perceived Social Support, and Grief Experiences among Bereaved College Students | College Student Journal | Not mental health or wellbeing outcomes |
| 893 | Governale, A.;McTighe, K.;Cechova, V.; | 2024 | Psychological reactions to COVID-19: Ambiguous loss, posttraumatic growth, and coronavirus impact among college students | Psychol Trauma | Not related wellbeing |
| 894 | Lommen, M. J. J.; | 2024 | Psychological predictors of posttraumatic stress and depression in firefighters: A 2-year longitudinal study | Mental Health and Prevention | Mental health disorders |
| 895 | Guerra, F.;Cilli, E.;Gentili, N.;Cogodi, E.;Ranieri, J.; | 2024 | Psychological modeling in youth behind parental cancer experience: the predictive role of emotion regulation | Journal of Psychopathology | Not in a higher education context |
| 896 | Wu, J.;Zhang, S.; | 2017 | Psychological measurement pattern based on social support data flow | Boletin Tecnico/Technical Bulletin | Not in a higher education context |
| 897 | Arslan, G.; | 2022 | Psychological maltreatment and substance use among college students: Psychological distress, belongingness, and social support | J Ethn Subst Abuse | Mental health disorders |
| 898 | Arslan, G.; | 2021 | Psychological Maltreatment and Spiritual Wellbeing in Turkish College Young Adults: Exploring the Mediating Effect of College Belonging and Social Support | J Relig Health | The population is not college students |
| 899 | Zhang, J.;Qi, Q.;Delprino, R. P.; | 2017 | Psychological health among Chinese college students: a rural/urban comparison | J Child Adolesc Ment Health | Not related wellbeing |
| 900 | Ye, B.;Chen, X.;Zhang, Y.;Yang, Q.; | 2022 | Psychological flexibility and COVID-19 burnout in Chinese college students: A moderated | J Contextual Behav Sci | Not related wellbeing |

| | | | | | |
|---|---|---|---|---|---|
| | | | mediation model | | |
| 901 | Vungkhanching, M.;Tonsing, J. C.;Tonsing, K. N.; | 2017 | Psychological Distress, Coping, and Perceived Social Support in Social Work Students | British Journal of Social Work | Mental health disorders |
| 902 | Xu, T.; | 2021 | Psychological Distress of International Students during the COVID-19 Pandemic in China: Multidimensional Effects of External Environment, Individuals' Behavior, and Their Values | Int J Environ Res Public Health | Mental health disorders |
| 903 | Sintos, M. L.; | 2020 | Psychological distress of Filipino deaf: Role of environmental vulnerabilities, self-efficacy, and perceived functional social support | Asia-Pacific Social Science Review | Mental health disorders |
| 904 | Bazarova, N. N.;Choi, Y. H.;Whitlock, J.;Cosley, D.;Sosik, V.; | 2017 | Psychological Distress and Emotional Expression on Facebook | Cyberpsychol Behav Soc Netw | Mental health disorders |
| 905 | Xu, T.;Zhu, P. T.;Ji, Q. Y.;Wang, W.;Qian, M. Y.;Shi, G. H.; | 2023 | Psychological distress and academic self-efficacy of nursing undergraduates under the normalization of COVID-19: multiple mediating roles of social support and mindfulness | BMC medical education | Mental health disorders |
| 906 | Lew, B.;Osman, A.;Chan, C. M. H.;Chen, W. S.;Ibrahim, N.;Jia, C. X.;Siau, C. S.; | 2021 | Psychological characteristics of suicide attempters among undergraduate college students in China: a cross-sectional study | BMC Public Health | Mental health disorders |
| 907 | Destin, M.;Debrosse, R.;Rheinschmidt-Same, M.;Richeson, J. A.; | 2022 | Psychological Challenges and Social Supports That Shape the Pursuit of Socioeconomic Mobility | RSF | Mental health disorders |
| 908 | Zwolinski, J.; | 2012 | Psychological and neuroendocrine reactivity to ostracism | Aggressive behavior | Mental health disorders |

| | | | | | |
|---|---|---|---|---|---|
| 909 | Zhu, R.;Fang, Y.;Li, H.;Liu, Y.;Wei, J.;Zhang, S.;Wang, L.;Fan, R.;Wang, L.;Li, S.;et al.,; | 2023 | Psychobiotic Lactobacillus plantarum JYLP-326 relieves anxiety, depression, and insomnia symptoms in test anxious college via modulating the gut microbiota and its metabolism. | Frontiers in immunology | Meta review |
| 910 | Regli, J.;Sadeghi-Bahmani, D.;Rigotti, V.;Stanga, Z.;Ülgür, I. I.;Fichter, C.;Lang, U. E.;Brühl, A. B.;Brand, S.; | 2024 | Psychiatric Characteristics, Symptoms of Insomnia and Depression, Emotion Regulation, and Social Activity among Swiss Medical Students | Journal of Clinical Medicine | Mental health disorders |
| 911 | Hao, F.;Pang, G. Y.;Wu, Y. L.;Pi, Z. L.;Xia, L. R.;Min, G. Y.; | 2019 | Providing Appropriate Social Support to Prevention of Depression for Highly Anxious Sufferers | Ieee Transactions on Computational Social Systems | Mental health disorders |
| 912 | Coffin, J.;Vaz, S.;Kickett-Tucker, C.;Milroy, H.;Olsson, C.;Kirby, M.;Nelson, L.;McPhee, R.;Cross, D.; | 2024 | Protocol to implement and evaluate a culturally secure, strength-based, equine-assisted learning program, "Yawardani Jan-ga" (horses helping), to support the social and emotional well-being of Australian aboriginal children and young people | PLoS ONE | The population is not college students |
| 913 | Bergmans, R. S.;Wegryn-Jones, R.;Klida, C.;Kurtz, V.;Thomas, L.;Williams, D. A.;Clauw, D. J.;Kidwell, K. M.;Bohnert, A. S. B.;Boehnke, K. F.; | 2024 | Protocol for a pragmatic trial of Cannabidiol (CBD) to improve chronic pain symptoms among United States Veterans | BMC complementary medicine and therapies | Not in a higher education context |
| 914 | O'Connor, J.;Smith, L.;Woerner, J.;Khan, A.; | 2024 | Protective Factors for Sexual Violence Perpetration Among High School and College Students: A Systematic Review | Trauma Violence Abuse | The population is not college students |
| 915 | Howell, K. H.;Miller-Graff, L. E.; | 2014 | Protective factors associated with resilient functioning in young adulthood after childhood | Child Abuse Negl | The population is not college students |

| | | | | | |
|---|---|---|---|---|---|
| | | | exposure to violence | | |
| 916 | Mushonga, D. R.;Henneberger, A. K.; | 2020 | Protective factors associated with positive mental health in traditional and nontraditional Black students | Am J Orthopsychiatry | Not related wellbeing |
| 917 | Cusack, S. E.;Bourdon, J. L.;Bountress, K.;Saunders, T. R.;Kendler, K. S.;Dick, D. M.;Amstadter, A. B.; | 2021 | Prospective Predictors of Sexual Revictimization Among College Students | J Interpers Violence | Mental health disorders |
| 918 | Liu, H. X.;Chow, B. C.;Hassel, H.;Huang, Y. W.;Liang, W.;Wang, R. B.; | 2024 | Prospective association of eHealth literacy and health literacy with physical activity among Chinese college students: a multiple mediation analysis | Front Public Health | Not mental health or wellbeing outcomes |
| 919 | He, Y.;Liu, Q.;Turel, O.;He, Q.;Zhang, S.; | 2023 | Prosocial behavior predicts meaning in life during the COVID-19 pandemic: The longitudinal mediating role of perceived social support. | Front Public Health | Not related wellbeing |
| 920 | Chinawa, A.;Aronu, A.;Ossai, E.;Chinawa, J.; | 2022 | Promotive factors associated with internalizing symptoms amongst college students during the COVID-19 lockdown in Enugu metropolis, Nigeria | S Afr J Psychiatr | Not related wellbeing |
| 921 | Chang, S. M.;Lin, Y. H.;Lin, C. W.;Chang, H. K.;Chong, P. P.; | 2014 | Promoting positive psychology using social networking sites: a study of new college entrants on Facebook | Int J Environ Res Public Health | Mental health disorders |
| 922 | Dvořáková, K.;Kishida, M.;Li, J.;Elavsky, S.;Broderick, P. C.;Agrusti, M. R.;Greenberg, M. T.; | 2017 | Promoting healthy transition to college through mindfulness training with first-year college students: pilot randomized controlled trial | Journal of American College Health | Mental health disorders |

| | | | | | |
|---|---|---|---|---|---|
| 923 | Wang, Y. H.;Liao, H. C.; | 2014 | Promoting English oral communication and higher-order thinking in Taiwanese ESL students through the use of knowledge visualization techniques | Perceptual and motor skills | Mental health disorders |
| 924 | Yan, X.;Zhang, J.;Gong, Q.;Weng, X.; | 2011 | Prolonged high-altitude residence impacts verbal working memory: an fMRI study. | Experimental brain research | Mental health disorders |
| 925 | Al-Gamal, E.;Saeed, S. B.;Victor, A.;Long, T.; | 2019 | Prolonged grief disorder and its relationship with perceived social support and depression among university students | J Psychosoc Nurs Ment Health Serv | Mental health disorders |
| 926 | Milman, E.;Neimeyer, R. A.;Fitzpatrick, M.;MacKinnon, C. J.;Muis, K. R.;Cohen, S. R.; | 2019 | Prolonged Grief and the Disruption of Meaning: Establishing a Mediation Model | Journal of Counseling Psychology | Mental health disorders |
| 927 | Suárez-Perdomo, A.;Ruiz-Alfonso, Z.;Garcés-Delgado, Y.; | 2022 | Profiles of undergraduates' networks addiction: Difference in academic procrastination and performance | Computers & Education | Mental health disorders |
| 928 | Metzger, Isha W.;Cooper, Shauna M.;Ritchwood, Tierney D.;Onyeuku, Chisom;Griffin, Charity Brown; | 2017 | Profiles of African American College Students' Alcohol Use and Sexual Behaviors: Associations With Stress, Racial Discrimination, and Social Support | The Journal of Sex Research | Mental health disorders |
| 929 | Liu, Xinqiao;Ji, Xinyu;Zhang, Yifan;Gao, Wenjuan; | 2023 | Professional Identity and Career Adaptability among Chinese Engineering Students: The Mediating Role of Learning Engagement | Behavioral Sciences | Mental health disorders |
| 930 | Campo-Arias, A.;Herazo, E.;Caballero-Domínguez, C. C.; | 2024 | Prodromal questionnaire (PQ-16) dimensionality among Colombian adolescent school students | Early Intervention in Psychiatry | Mental health disorders |
| 931 | Dour, C. A.;Horacek, T. M.;Schembre, S. M.;Lohse, | 2013 | Process evaluation of Project WebHealth: a non-dieting Web-based intervention for obesity | Journal of nutrition education and | Mental health disorders |

| | | | | | |
|---|---|---|---|---|---|
| | B.;Hoerr, S.;Kattelmann, K.;White, A. A.;Shoff, S.;Phillips, B.;Greene, G.; | | prevention in college students | behavior | |
| 932 | Tomás, C. C.;Oliveira, E.;Sousa, D.;Uba-Chupel, M.;Furtado, G.;Rocha, C.;Teixeira, A.;Ferreira, P.;Alves, C.;Gisin, S.;Catarino, E.;Carvalho, N.;Coucelo, T.;Bonfim, L.;Silva, C.;Franco, D.;González, J. A.;Jardim, H. G.;Silva, R.;Baixinho, C. L.;Presado Mª, | 2016 | Proceedings of the 3rd IPLeiria's International Health Congress: Leiria, Portugal. 6-7 May 2016 | BMC Health Serv Res | Mental health disorders |
| 933 | Khan, Aqeel;Hamdan, Abdul Rahim;Ahmad, Roslee;Mustaffa, Mohamed Sharif;Mahalle, Salwa; | 2016 | Problem-Solving Coping and Social Support as Mediators of Academic Stress and Suicidal Ideation Among Malaysian and Indian Adolescents | Community Mental Health Journal | Mental health disorders |
| 934 | Zhang, M.;Sun, X.;Qin, X.;Ren, X.;Wen, C.;Xie, F.;Chen, B.;Dai, Q.; | 2022 | Problematic utilization of online social networking site in Chinese college students: prediction of personality and dynamic mediators | Curr Psychol | Mental health disorders |
| 935 | Faílde Garrido, J. M.;Dapía Conde, M. D.;Isorna Folgar, M.;Braña Rey, F.; | 2024 | Problematic Use of Video Games in Schooled Adolescents: The Role of Passion | Behavioral Sciences | Mental health disorders |
| 936 | Chen, Y.;Liu, X.;Chiu, D. T.;Li, Y.;Mi, B.;Zhang, Y.;Ma, L.;Yan, H.; | 2022 | Problematic Social Media Use and Depressive Outcomes among College Students in China: observational and Experimental Findings | International journal of environmental research and public health | Mental health disorders |

| | | | | | |
|---|---|---|---|---|---|
| 937 | Sun, Changkang;Sun, Binghai;Lin, Yishan;Zhou, Hui; | 2022 | Problematic Mobile Phone Use Increases with the Fear of Missing Out Among College Students: The Effects of Self-Control, Perceived Social Support and Future Orientation | Psychology Research and Behavior Management | Mental health disorders |
| 938 | Mekonnen, Y. S.;Tessema, S. A.;Bedane, S. D.;Ali, A. B.; | 2024 | Problematic Internet use among resident physicians at St. Paul's Hospital Millennium Medical College in Addis Ababa, Ethiopia | BMC Psychiatry | Mental health disorders |
| 939 | Qin, Q.;Liu, H.;Yang, Y.;Wang, Y.;Xia, C.;Tian, P.;Wei, J.;Li, S.;Chen, T.; | 2021 | Probiotic Supplement Preparation Relieves Test Anxiety by Regulating Intestinal Microbiota in College Students | Disease markers | Mental health disorders |
| 940 | Malan-Müller, S.;Vidal, R.;O'Shea, E.;Montero, E.;Figuero, E.;Zorrilla, I.;de Diego-Adeliño, J.;Cano, M.;García-Portilla, M. P.;González-Pinto, A.;Leza, J. C.; | 2024 | Probing the oral-brain connection: oral microbiome patterns in a large community cohort with anxiety, depression, and trauma symptoms, and periodontal outcomes | Translational Psychiatry | Mental health disorders |
| 941 | Salis, A. S.; | 2013 | Proactive and reactive effects of vigorous exercise on learning and vocabulary comprehension | Perceptual and motor skills | Mental health disorders |
| 942 | Clark, C. J.;Al-Hamdan, Z.;Bawadi, H.;Alsalem, H.;Hamadneh, J.;Abu Al-Haija, A.;Hadd, A. R.;Spencer, R. A.;Bergenfield, I.;Hall-Clifford, R.; | 2024 | Preventing violence and enhancing mental health among clients of an invitro fertilization clinic in Jordan: results of a pre/post pilot test of the use of cognitive behavioral therapy | Reproductive Health | Mental health disorders |
| 943 | Webermann, A. R.;Murphy, C. M.;Singh, R.;Schacht, R. L.; | 2022 | Preventing Relationship Abuse Among College Students: a Controlled Trial of the Skills for Healthy Adult Relationships (SHARe) Program | Journal of Interpersonal Violence | Mental health disorders |
| 944 | Donaldson, C. D.;Siegel, J. T.;Crano, W. D.; | 2020 | Preventing college student nonmedical prescription stimulant use: development of vested | Addictive behaviors | Not related wellbeing |

| | | | | | |
|---|---|---|---|---|---|
| | | | interest theory-based persuasive messages | | |
| 945 | Testa, M.;Livingston, J. A.;Wang, W.;Lewis, M. A.; | 2020 | Preventing College Sexual Victimization by Reducing Hookups: a Randomized Controlled Trial of a Personalized Normative Feedback Intervention | Prevention science | Mental health disorders |
| 946 | Davico, C.;Graziano, F.;Rossi Ghiglione, A.;Amianto, F.;Begotti, T.;Calandri, E.;Copetto, G.;Di Franco, F.;Lonardelli, E.;Marcotulli, D.;Olcuire, L.;Ricci, F.;Vitiello, B.; | 2024 | Preventing Adolescent Suicide: Feasibility and Preliminary Outcome Evaluation of a Theatre-Based Gatekeeper Training for Teachers | International journal of environmental research and public health | Mental health disorders |
| 947 | Zhang, M.;Zhang, J.;Zhang, F.;Zhang, L.;Feng, D.; | 2018 | Prevalence of psychological distress and the effects of resilience and perceived social support among Chinese college students: Does gender make a difference? | Psychiatry Res | Mental health disorders |
| 948 | Silveira, R.;Eleti, S.;Saruchera, E.;Mwamuka, R.;Whitwell, S.;Abas, M. A.;Jack, H. E.; | 2024 | Prevalence of probable post-traumatic stress disorder and experiences of trauma in emerging adults living with HIV in Zimbabwe | BJPsych Open | The population is not college students |
| 949 | Aslan, B.;Önal, Ö; | 2024 | Prevalence of probable post-traumatic stress disorder among survivors of the 2023 earthquakes in Türkiye | Eastern Mediterranean Health Journal | Mental health disorders |
| 950 | Parker, M.;Duran, B.;Rhew, I.;Magarati, M.;Egashira, L.;Larimer, M.;Donovan, D.; | 2021 | Prevalence of Moderate and Acute Suicidal Ideation among a National Sample of Tribal College and University Students 2014-2015 | Arch Suicide Res | Mental health disorders |
| 951 | Wu, X. S.;Zhang, Z. H.;Zhao, F.;Wang, W. J.;Li, Y. F.;Bi, L. D.;Qian, Z. Z.;Lu, S. S.;Feng, F.;Hu, | 2016 | Prevalence of Internet addiction and its association with social support and other related factors among adolescents in China | Journal of Adolescence | Mental health disorders |

| | | | | | |
|---|---|---|---|---|---|
| | C. Y.;Gong, F. F.;Sun, Y. H.; | | | | |
| 952 | Racine, N.;Pitt, T.;Premji, S.;McDonald, S. W.;Patten, S. B.;Tough, S.;Madigan, S.; | 2024 | Prevalence of Common Child Mental Health Disorders Using Administrative Health Data and Parent Report in a Prospective Community-Based Cohort from Alberta, Canada: Prévalence des troubles communs de santé mentale de l'enfant à l'aide des données de santé a | Canadian Journal of Psychiatry | The population is not college students |
| 953 | Ssirimuzaawo, J.;Musoke, M.;Kiyingi, P. F.; | 2024 | Prevalence of attention deficit hyperactive disorder (ADHD) symptoms in selected government primary schools in Wakiso District, Uganda | Quality Education for All | Mental health disorders |
| 954 | Al-Saadi, L. S.;Chan, M. F.;Al Sabahi, A.;Alkendi, J.;Al-Mashaikhi, N.;Sumri, H. A.;Al-Fahdi, A.;Al-Azri, M.; | 2024 | Prevalence of anxiety, depression, and post-traumatic stress disorder among Omani children and adolescents diagnosed with cancer: a prospective cross-sectional study | BMC Cancer | The population is not college students |
| 955 | Jimènez-Barragan, M.;Falguera-Puig, G.;Curto-Garcia, J. J.;Monistrol, O.;Coll-Navarro, E.;Tarragó-Grima, M.;Ezquerro-Rodriguez, O.;Ruiz, A. C.;Codina-Capella, L.;Urquizu, X.;Pino Gutierrez, A. D.; | 2024 | Prevalence of anxiety and depression and their associated risk factors throughout pregnancy and postpartum: a prospective cross-sectional descriptive multicentre study | BMC Pregnancy and Childbirth | Mental health disorders |
| 956 | Lilhore, U. K.;Dalal, S.;Varshney, N.;Sharma, Y. K.;Rao, K. B. V. B.;Rao, V. V. R. M.;Alroobaea, R.;Simaiya, S.;Margala, M.;Chakrabarti, P.; | 2024 | Prevalence and risk factors analysis of postpartum depression at an early stage using a hybrid deep learning model | Scientific Reports | Mental health disorders |

| | | | | | |
|---|---|---|---|---|---|
| 957 | Frey, L. M.;Venugopal, D.;Dev, V. S.; | 2024 | Prevalence and predictors of suicide ideation among university and high-school students during India's 2nd wave of the COVID-19 pandemic | PLoS ONE | Mental health disorders |
| 958 | Si, M. Y.;Su, X. Y.;Jiang, Y.;Wang, W. J.;Gu, X. F.;Ma, L.;Li, J.;Zhang, S. K.;Ren, Z. F.;Liu, Y. L.;Qiao, Y. L.; | 2021 | Prevalence and Predictors of PTSD During the Initial Stage of COVID-19 Epidemic among Female College Students in China | Inquiry | Mental health disorders |
| 959 | Wilcox, H. C.;Arria, A. M.;Caldeira, K. M.;Vincent, K. B.;Pinchevsky, G. M.;O'Grady, K. E.; | 2010 | Prevalence and predictors of persistent suicide ideation, plans, and attempts during college | J Affect Disord | Published not from January 2010 to 31 March 2024 |
| 960 | Yuan, L. L.;Lu, L.;Wang, X. H.;Guo, X. X.;Ren, H.;Gao, Y. Q.;Pan, B. C.; | 2021 | Prevalence and Predictors of Anxiety and Depressive Symptoms Among International Medical Students in China During COVID-19 Pandemic | Front Psychiatry | Mental health disorders |
| 961 | Sun, H. L.;Chen, P.;Bai, W.;Zhang, L.;Feng, Y.;Su, Z.;Cheung, T.;Ungvari, G. S.;Cui, X. L.;Ng, C. H.;An, F. R.;Xiang, Y. T.; | 2024 | Prevalence and network structure of depression, insomnia, and suicidality among mental health professionals who recovered from COVID-19: a national survey in China | Translational Psychiatry | Mental health disorders |
| 962 | Yu, M.;Tian, F.;Cui, Q.;Wu, H.; | 2021 | Prevalence and its associated factors of depressive symptoms among Chinese college students during the COVID-19 pandemic | BMC Psychiatry | Mental health disorders |
| 963 | Lasong, J.;Salifu, Y.;Kakungu, J. A. W. M.; | 2024 | Prevalence and factors associated with tramadol use among university students in Ghana: a cross-sectional survey | BMC Psychiatry | Not mental health or wellbeing outcomes |
| 964 | Walsh, K.;Sarvet, A. L.;Wall, M.;Gilbert, L.;Santelli, J.;Khan, | 2021 | Prevalence and Correlates of Sexual Assault Perpetration and Ambiguous Consent in a | J Interpers Violence | Mental health disorders |

| | | | | | |
|---|---|---|---|---|---|
| | S.;Thompson, M. P.;Reardon, L.;Hirsch, J. S.;Mellins, C. A.; | | Representative Sample of College Students | | |
| 965 | Xu, Y.;Chi, X.;Chen, S.;Qi, J.;Zhang, P.;Yang, Y.; | 2014 | Prevalence and correlates of depression among college nursing students in China | Nurse Educ Today | Mental health disorders |
| 966 | Liu, J.;Tai, Z.;Hu, F.; | 2024 | Prevalence and coping of depression and anxiety among college students during COVID-19 lockdowns in China | J Affect Disord | Mental health disorders |
| 967 | Fung, H. W.;Yuan, G. F.;Liu, C.;Lin, E. S. S.;Lam, S. K. K.;Wong, J. Y. H.; | 2024 | Prevalence and clinical correlates of dissociative symptoms in people with complex PTSD: Is complex PTSD a dissociative disorder? | Psychiatry Research | Mental health disorders |
| 968 | Yaghubi, H.;Soleimani, L.;Abedi Yarandi, M. S.;Mollaei, A.;Mahdavinoor, S. M. M.; | 2024 | Prevalence and associated factors of suicide-related behaviors in Iranian students: a large sample cross-sectional study | Middle East Current Psychiatry | Mental health disorders |
| 969 | Cabriales, J. A.;Cooper, T. V.;Taylor, T.; | 2013 | Prescription drug misuse, illicit drug use, and their potential risk and protective correlates in a Hispanic college student sample | Exp Clin Psychopharmacol | Mental health disorders |
| 970 | Lai, I. J.;Chang, L. C.;Lee, C. K.;Liao, L. L.; | 2023 | Preliminary evaluation of a scenario-based nutrition literacy online program for college students: a pilot study | Public health nutrition | Not related wellbeing |
| 971 | Rodríguez, M. S.;Tinajero, C.;Páramo, M. F.; | 2017 | Pre-entry Characteristics, Perceived Social Support, Adjustment and Academic Achievement in First-Year Spanish University Students: A Path Model | Journal of Psychology | Mental health disorders |
| 972 | Williston, Sarah Krill;Roemer, Lizabeth; | 2017 | Predictors of well-being in the lives of student service members and veterans | Journal of American College Health | Mental health disorders |

| | | | | | |
|---|---|---|---|---|---|
| 973 | van den End, A.;Snoek, A.;Aarts, I.;Beekman, A. T. F.;Dekker, J.;Blankers, M.;Lommerse, N.;Thomaes, K.; | 2024 | Predictors of treatment attendance in patients with posttraumatic stress disorder and comorbid personality disorders | Comprehensive Psychiatry | Mental health disorders |
| 974 | Kurniawan, D.;Fitriawan, A. S.;Susanti, B. A. D.;Firdaus, I.;Suparmanto, G.;Kafil, R. F.;Wulandari, A. N.;Setyaningsih, W. A. W.;Puspitarini, Z.;Wijoyo, E. B.; | 2024 | Predictors of suicidal behaviors among school-going adolescents: a cross-sectional study in Indonesia | Middle East Current Psychiatry | Mental health disorders |
| 975 | Wright, S.;Karyotaki, E.;Cuijpers, P.;Bisson, J.;Papola, D.;Witteveen, A. B.;Back, S. E.;Bichescu-Burian, D.;Capezzani, L.;Cloitre, M.;Devilly, G. J.;Elbert, T.;Mello, M.;Ford, J. D.;Grasso, D.;Gamito, P.;Gray, R.;Haller, M.;Hunt, N.;Kleber, R. J.;König, J | 2024 | Predictors of study dropout in cognitive-behavioral therapy with a trauma focus for post-traumatic stress disorder in adults: An individual participant data meta-analysis | BMJ Mental Health | Meta review |
| 976 | Brewer, S. E.;Nicotera, N.;Veeh, C.;Laser-Maira, J. A.; | 2018 | Predictors of positive development in first-year college students | J Am Coll Health | Mental health disorders |
| 977 | Samek, D. R.;Akua, B. A.; | 2022 | Predictors of persistent alcohol use disorder and co-occurring depressive symptoms: Insights from the longitudinal college experiences study | J Adolesc | Mental health disorders |
| 978 | Wen, F. H.;Prigerson, H. G.;Chuang, L. P.;Chou, W. C.;Huang, C. C.;Hu, T. H.;Tang, S. T.; | 2024 | Predictors of ICU Surrogates' States of Concurrent Prolonged Grief, Posttraumatic Stress, and Depression Symptoms* | Critical Care Medicine | Mental health disorders |

| | | | | | |
|---|---|---|---|---|---|
| 979 | Liu, Y.;Zhang, N.;Bao, G.;Huang, Y.;Ji, B.;Wu, Y.;Liu, C.;Li, G.; | 2019 | Predictors of depressive symptoms in college students: A systematic review and meta-analysis of cohort studies | J Affect Disord | Meta review |
| 980 | Blow, J.;Cooper, T. V.; | 2014 | Predictors of body dissatisfaction in a Hispanic college student sample | Eating Behaviors | Mental health disorders |
| 981 | Marôco, J.;Assunção, H.;Harju-Luukkainen, H.;Lin, S. W.;Sit, P. S.;Cheung, K. C.;Maloa, B.;Ilic, I. S.;Smith, T. J.;Campos, Jadb; | 2020 | Predictors of academic efficacy and dropout intention in university students: Can engagement suppress burnout? | PLoS ONE | Mental health disorders |
| 982 | Vindbjerg, E.;Sandahl, H.;Lindberg, L.;Attardo, H.;Mortensen, E.;Carlsson, J.; | 2024 | Predictors and Patterns of Dropout From Psychiatric Treatment Among Trauma-Affected Refugees: A Large Data Pool Analysis | Clinical Psychology and Psychotherapy | Mental health disorders |
| 983 | Quinn, P. D.;Fromme, K.; | 2011 | Predictors and outcomes of variability in subjective alcohol intoxication among college students: an event-level analysis across 4 years | Alcoholism, clinical and experimental research | Mental health disorders |
| 984 | Giumetti, G. W.;Kowalski, R. M.;Feinn, R. S.; | 2022 | Predictors and outcomes of cyberbullying among college students: A two-wave study | Aggress Behav | Mental health disorders |
| 985 | Mohajeri, M.;Towsyfyan, N.;Tayim, N.;Faroji, B. B.;Davoudi, M.; | 2024 | Prediction of Suicidal Thoughts and Suicide Attempts in People Who Gamble Based on Biological-Psychological-Social Variables: A Machine Learning Study | Psychiatric quarterly | Mental health disorders |
| 986 | Jekauc, D.;Völkle, M.;Wagner, M. O.;Mess, F.;Reiner, M.;Renner, B.; | 2015 | Prediction of attendance at fitness center: a comparison between the theory of planned behavior, the social cognitive theory, and the physical activity maintenance theory | Front Psychol | Mental health disorders |

| | | | | | |
|---|---|---|---|---|---|
| 987 | Zheng, W.;Chen, Q.;Yao, L.;Zhuang, J.;Huang, J.;Hu, Y.;Yu, S.;Chen, T.;Wei, N.;Zeng, Y.;Zhang, Y.;Fan, C.;Wang, Y.; | 2023 | Prediction Models for Sleep Quality Among College Students During the COVID-19 Outbreak: Cross-sectional Study Based on the Internet New Media | J Med Internet Res | Mental health disorders |
| 988 | Zhu, L.; | 2022 | Prediction model and case analysis of college students' psychological depression based on multi-source online comment mining | Front Public Health | Mental health disorders |
| 989 | Kiekens, G.;Hasking, P.;Claes, L.;Boyes, M.;Mortier, P.;Auerbach, R. P.;Cuijpers, P.;Demyttenaere, K.;Green, J. G.;Kessler, R. C.;Myin-Germeys, I.;Nock, M. K.;Bruffaerts, R.; | 2019 | Predicting the incidence of non-suicidal self-injury in college students | Eur Psychiatry | Mental health disorders |
| 990 | Hemal, S. H.;Khan, M. A. R.;Ahammad, I.;Rahman, M.;Khan, M. A. S.;Ejaz, S.; | 2024 | Predicting the Impact of Internet Usage on student's academic performance using machine learning techniques in Bangladesh Perspective | Social Network Analysis and Mining | Not related wellbeing |
| 991 | Kennedy, C. J.;Kearns, J. C.;Geraci, J. C.;Gildea, S. M.;Hwang, I. H.;King, A. J.;Liu, H.;Luedtke, A.;Marx, B. P.;Papini, S.;Petukhova, M. V.;Sampson, N. A.;Smoller, J. W.;Wolock, C. J.;Zainal, N. H.;Stein, M. B.;Ursano, R. J.;Wagner, J. R.;Kessler, R. C.; | 2024 | Predicting Suicides among US Army Soldiers after Leaving Active Service | JAMA Psychiatry | Mental health disorders |
| 992 | Bazrafshan, M.;Sayehmiri, K.; | 2024 | Predicting suicidal behavior outcomes: an analysis of key factors and machine learning models | BMC Psychiatry | Mental health disorders |

| | | | | | |
|---|---|---|---|---|---|
| 993 | Klaw, E. L.;Demers, A. L.;Da Silva, N.; | 2016 | Predicting Risk Factors for Intimate Partner Violence Among Post-9/11 College Student Veterans | J Interpers Violence | Mental health disorders |
| 994 | Domino, J. L.;Whiteman, S. E.;Weathers, F. W.;Blevins, C. T.;Davis, M. T.; | 2020 | Predicting PTSD and Depression Following Sexual Assault: The Role of Perceived Life Threat, Post-Traumatic Cognitions, Victim-Perpetrator Relationship, and Social Support | Journal of Aggression Maltreatment & Trauma | Mental health disorders |
| 995 | Rohde, P.;Stice, E.;Gau, J. M.; | 2017 | Predicting persistence of eating disorder compensatory weight control behaviors | International journal of eating disorders | Mental health disorders |
| 996 | Xie, W.;Karan, K.; | 2019 | Predicting Facebook addiction and state anxiety without Facebook by gender, trait anxiety, Facebook intensity, and different Facebook activities | J Behav Addict | Mental health disorders |
| 997 | Rice, K. G.;Arana, F.;Wetstone, H.;Aiello, M.;Durán, B.; | 2023 | Predicting and Moderating COVID-Fear and Stress among College Students in Argentina and the USA | Int J Environ Res Public Health | Mental health disorders |
| 998 | Calear, A. L.;Macleod, E.;Hoye, A. M.;McCallum, S.;Morse, A.;Farrer, L. M.;Batterham, P. J.; | 2024 | Pragmatic controlled trial of a school-based emotion literacy program for 8- to 10-year-old children: study protocol | BMC Psychiatry | The population is not college students |
| 999 | Hailu, G. N.; | 2020 | Practice of stress management behaviors and associated factors among undergraduate students of Mekelle University, Ethiopia: a cross-sectional study | BMC Psychiatry | Mental health disorders |
| 1000 | Boyraz, Güler;Horne, Sharon G.;Armstrong, Aisha P.;Owens, | 2015 | Posttraumatic stress predicting depression and social support among college students: moderating | Psychological Trauma: Theory, | Mental health disorders |

| | | | | | |
|---|---|---|---|---|---|
| | Archandria C.; | | effects of race and gender | Research, Practice, and Policy | |
| 1001 | Karaman, H. B.;Bulut, S.; | 2024 | Post-Traumatic Stress Disorder, Depression, and Perceived Social Support among Iraqi and Syrian Immigrant and Refugee Adolescents in Türkiye | Revista Colombiana de Psicologia | Mental health disorders |
| 1002 | Sharp, T. H.;Chideya, Y.;Giuliani, A.;Hunt, X.;Tomlinson, M.;Seedat, S.;Creswell, C.;Fearon, P.;Hamilton-Giachritsis, C.;Hiller, R.;Meiser-Stedman, R.;Toit, S. D.;Stewart, J.;Halligan, S. L.; | 2024 | Post-traumatic stress disorder symptoms following exposure to acute psychological trauma in children aged 8–16 years in South Africa: protocol for the Sinethemba longitudinal study | BMJ Open | The population is not college students |
| 1003 | Gebreyesus, A.;Gebremariam, A. G.;Kidanu, K. G.;Gidey, S.;Haftu, H.;Nigusse, A. T.;Shishay, F.;Mamo, L.; | 2024 | Post-traumatic stress disorder symptoms among internally displaced persons: unveiling the impact of the war of Tigray | Discover Mental Health | Mental health disorders |
| 1004 | Wang, J. Y.;Li, Q.;Liu, W.;Yang, Y.;Wang, X. G.;Liu, C. Y.;Shu, X. J.;Xue, L.;Shi, Y. W.; | 2023 | Posttraumatic stress disorder symptoms among Chinese college students following the COVID-19 outbreak | Front Neurosci | Mental health disorders |
| 1005 | Potik, D.;Einat, T.;Idisis, Y.; | 2024 | Posttraumatic Stress Disorder Symptom Clusters, Exposure to Potentially Morally Injurious Events, and Aggression Among Army Veterans | Clinical Psychology and Psychotherapy | Mental health disorders |
| 1006 | van de Vyver, M.;Benecke, R. M.;van den Heuvel, L.;Kruger, M. J.;Powrie, Y.;Seedat, S.;Smith, C.; | 2024 | Posttraumatic stress disorder is characterized by functional dysregulation of dermal fibroblasts. | Biochimie | Mental health disorders |
| 1007 | Moreland, A. D.;Rancher, C.;Davies, F.;Bottomley, J.;Galea, S.;Abba-Aji, | 2024 | Posttraumatic Stress Disorder among Adults in Communities with Mass Violence Incidents | JAMA network open | The population is not college students |

| | | | | | |
|---|---|---|---|---|---|
| | M.;Abdalla, S. M.;Schmidt, M. G.;Vena, J. E.;Kilpatrick, D. G.; | | | | |
| 1008 | He, W.;Xu, L.;Hu, Y.;Xu, Y.;Dong, T.;Zhao, H.; | 2023 | Positive valence ≠ positive effect: impact of positive meta-stereotypes on the cognitive performance | Journal of Social Psychology | Meta review |
| 1009 | Hirsch, Jameson K. PhD;Barton, Alison L. PhD; | 2011 | Positive Social Support, Negative Social Exchanges, and Suicidal Behavior in College Students | Journal of American College Health | Mental health disorders |
| 1010 | Caredda, M.;Vescera, L.;Picardi, A.;Tarolla, E.;Pancheri, C.;Biondi, M.;Tondo, L.; | 2024 | Positive psychological functioning, resilience, and styles of coping as buffers against suicidal behaviors. A case-control study | Journal of Affective Disorders | Mental health disorders |
| 1011 | Casagrande, K.;Frost, K. M.;Bailey, K. M.;Ingersoll, B. R.; | 2020 | Positive Predictors of Life Satisfaction for Autistic College Students and Their Neurotypical Peers | Autism Adulthood | Not related wellbeing |
| 1012 | Li, Q.;Hu, G.; | 2023 | Positive impacts of perceived social support on prosocial behavior: the chain mediating role of moral identity and moral sensitivity | Front Psychol | Not in a higher education context |
| 1013 | Fagbenro, R. K.;Sunindijo, R. Y.;Illankoon, C.; | 2024 | Positive Impact of Prefabrication on the Mental Health of Construction Workers | Buildings | Mental health disorders |
| 1014 | McQuade, J. D.;Taubin, D.;Mordy, A. E.; | 2024 | Positive Emotion Dysregulation and Social Impairments in Adolescents with and without ADHD | Research on Child and Adolescent Psychopathology | Mental health disorders |
| 1015 | Lowe, C. T.;Bath, A. C.;Callahan, B. L.;Climie, E. A.; | 2024 | Positive Childhood Experiences and the Indirect Relationship With Improved Emotion Regulation in Adults With ADHD Through Social Support | Journal of Attention Disorders | Mental health disorders |
| 1016 | Wang, C.;Zhou, R.;Zhang, X.; | 2023 | Positive Childhood Experiences and Depression Among College Students During the COVID-19 | Psychol Res Behav Manag | The population is not college students |

| | | | | | |
|---|---|---|---|---|---|
| | | | Pandemic: A Moderated Mediation Model | | |
| 1017 | Green, M.;Decourville, N.;Sadava, S.; | 2012 | Positive Affect, Negative Affect, Stress, and Social Support as Mediators of the Forgiveness-Health Relationship | Journal of Social Psychology | Mental health disorders |
| 1018 | Alacha, H. F.;Rosen, P. J.;Bufferd, S. J.; | 2024 | Positive Affect Variability is Associated with Homework Management Difficulties in Children with ADHD | Journal of Child and Family Studies | Mental health disorders |
| 1019 | Fernández-Delgado, M.;Cruz, S.;Cernadas, E.;Alateyat, H.;Tubío-Fungueiriño, M.;Sampaio, A.;Carracedo, A.;Fernández-Prieto, M.; | 2024 | Population-based detection of children ASD/ADHD comorbidity from atypical sensory processing | Applied Intelligence | Mental health disorders |
| 1020 | Varshney, D. K. S.;Agrawal, M.;Tripathi, R. K.;Rasaily, S.; | 2024 | Pioneering approaches: Navigating mind wandering and self-silencing in dissociated adolescent female sexual trauma survivors - An interpretative phenomenological analysis | European Journal of Trauma and Dissociation | Mental health disorders |
| 1021 | Moore, M. J.;Werch, C. E.;Bian, H.; | 2012 | The pilot of a computer-based brief multiple-health behavior intervention for college students | Journal of American College Health | Mental health disorders |
| 1022 | Jensen, M. L.;Storebø, O. J.;Bjerrum, M. B.;Vamosi, M.; | 2024 | Physical activity for children and adolescents with attention deficit hyperactivity disorder: a protocol of a systematic review and meta-analysis | BMJ Open | Meta review |
| 1023 | Oh, H.;Landré, B.;Yon, D. K.;Frajerman, A.;Gyasi, R. M.;Jacob, L.; | 2024 | Physical activity and suicidal behavior in medical students from the United States | Journal of Affective Disorders | Mental health disorders |

| | | | | | |
|---|---|---|---|---|---|
| 1024 | Joseph, R. P.;Pekmezi, D. W.;Lewis, T.;Dutton, G.;Turner, L. W.;Durant, N. H.; | 2013 | Physical Activity and Social Cognitive Theory Outcomes of an Internet-Enhanced Physical Activity Intervention for African American Female College Students | J Health Dispar Res Pract | Mental health disorders |
| 1025 | Alkatan, M.;Alsharji, K.;Akbar, A.;Alshareefi, A.;Alkhalaf, S.;Alabduljader, K.;Al-Hazzaa, H. M.; | 2021 | Physical activity and sedentary behaviors among active college students in Kuwait relative to gender status | Journal of preventive medicine and hygiene | Mental health disorders |
| 1026 | Abrantes, L. C. S.;de Souza de Morais, N.;Gonçalves, V. S. S.;Ribeiro, S. A. V.;de Oliveira Sediyama, C. M. N.;do Carmo Castro Franceschini, S.;Dos Santos Amorim, P. R.;Priore, S. E.; | 2022 | Physical activity and quality of life among college students without comorbidities for cardiometabolic diseases: systematic review and meta-analysis | Qual Life Res | Meta review |
| 1027 | Niu, L.;Xu, J.;E, Y.; | 2023 | Physical Activity and Habitus: Parental Support or Peer Support? | Int J Environ Res Public Health | Mental health disorders |
| 1028 | Zhang, J.;Uscola, C.;Abrutyn, S.;Mueller, A. S.; | 2024 | Phenomenology, Cultural Meaning, and the Curious Case of Suicide: Localizing the Structure-Culture Dialectic | Philosophy of the Social Sciences | Mental health disorders |
| 1029 | Galovski, T. E.;McSweeney, L. B.;Nixon, R. D. V.;Wachen, J. S.;Smith, B. N.;Noorbaloochi, S.;Vogt, D.;Niles, B. L.;Kehle-Forbes, S. M.; | 2024 | Personalizing cognitive processing therapy with a case formulation approach to intentionally target impairment in psychosocial functioning associated with PTSD | Contemporary clinical trial communications | Mental health disorders |
| 1030 | Zhao, Y.;Wang, W.;Wang, M.;Gao, F.;Hu, C.;Cui, B.;Yu, W.;Ren, H.; | 2022 | Personalized individual-based exercise prescriptions are effective in treating depressive | Frontiers in Psychiatry | Mental health disorders |

| | | | | | |
|---|---|---|---|---|---|
| | | | symptoms of college students during the COVID-19: a randomized controlled trial in China. | | |
| 1031 | Kim, S. C.;Song, J. H.;Kong, N. Y.; | 2024 | Personalized Game-Based Content and Performance: A Pilot Study on a Digital Intervention for Children with ADHD | Bioengineering | Mental health disorders |
| 1032 | Braitman, A. L.;Lau-Barraco, C.; | 2018 | Personalized Boosters After a Computerized Intervention Targeting College Drinking: a Randomized Controlled Trial | Alcoholism, clinical and experimental research | Mental health disorders |
| 1033 | Nikbin, D.;Iranmanesh, M.;Foroughi, B.; | 2021 | Personality traits, psychological well-being, Facebook addiction, health and performance: testing their relationships | Behavior & Information Technology | Mental health disorders |
| 1034 | Tang, J. H.;Chen, M. C.;Yang, C. Y.;Chung, T. Y.;Lee, Y. A.; | 2016 | Personality traits, interpersonal relationships, online social support, and Facebook addiction | Telematics and Informatics | Mental health disorders |
| 1035 | Laranjeira, C.;Querido, A.; | 2021 | Personality and coping as gendered predictors of distress and well-being in nursing students | European Psychiatry | Mental health disorders |
| 1036 | Carlson, E. B.;Barlow, M. R.;Palmieri, P. A.;Shieh, L.;Mellman, T. A.;Cooksey, E.;Parker, J.;Williams, M.;Spain, D. A.; | 2024 | Performance replication of the Hospital Mental Health Risk Screen in ethnoracial diverse U.S. patients admitted through emergency care. | PLoS ONE | Mental health disorders |
| 1037 | Stewart, D. W.;Thomas, J. L.;Copeland, A. L.; | 2010 | Perceptions of social support provided to smokers | Journal of Smoking Cessation | Published not from January 2010 to 31 March 2024 |
| 1038 | Stewart, C. M.;Master, A.;Mire, S. S.;Hassett, K. S.;Smith, B. H.; | 2024 | Perceptions of Academic Performance, Impairment, and Mental Health in University Students With and Without ADHD | Journal of Attention Disorders | Mental health disorders |

| | | | | | |
|---|---|---|---|---|---|
| 1039 | Panteli, M.;Vaiouli, P.;Leonidou, C.;Panayiotou, G.; | 2021 | Perceived Stress of Cypriot College Students during COVID-19: The Predictive Role of Social Skills and Social Support | European Journal of Psychology Open | Mental health disorders |
| 1040 | Bareket-Bojmel, L.;Shahar, G.;Abu-Kaf, S.;Margalit, M.; | 2021 | Perceived social support, loneliness, and hope during the COVID-19 Pandemic: Testing a mediating model in the UK, USA, and Israel | British Journal of Clinical Psychology | Mental health disorders |
| 1041 | Yang, Xiaofan;Ma, Hang;Zhang, Ling;Xue, Jinyang;Hu, Ping; | 2023 | Perceived Social Support, Depressive Symptoms, Self-Compassion, and Mobile Phone Addiction: A Moderated Mediation Analysis | Behavioral Sciences | Mental health disorders |
| 1042 | Tinajero, C.;Cadaveira, F.;Rodríguez, M. S.;Páramo, M. F.; | 2019 | Perceived Social Support from Significant Others among Binge Drinking and Polyconsuming Spanish University Students | International journal of environmental research and public health | Not in a higher education context |
| 1043 | Catu, L. R. E.; | 2021 | Perceived Social Support from Family, Friends, and Spiritual Experiences as Correlates of Depression | Philippine Journal of Nursing | Mental health disorders |
| 1044 | Kähkönen, Outi;Kankkunen, Päivi;Miettinen, Heikki;Lamidi, Marja-Leena;Saaranen, Terhi; | 2017 | Perceived social support following percutaneous coronary intervention is a crucial factor in patients with coronary heart disease. | Journal of Clinical Nursing | Mental health disorders |
| 1045 | Zhou, Xueting;Zhu, Hong;Zhang, Bin;Cai, Taisheng; | 2013 | PERCEIVED SOCIAL SUPPORT AS MODERATOR OF PERFECTIONISM, DEPRESSION, AND ANXIETY IN COLLEGE STUDENTS | Social Behavior and Personality | Mental health disorders |
| 1046 | Tinajero, C.;Martínez-López, Z.;Rodríguez, M. S.;Páramo, M. F.; | 2020 | Perceived social support as a predictor of academic success in Spanish university students | Anales De Psicologia | Not in a higher education context |

| | | | | | |
|---|---|---|---|---|---|
| 1047 | Shi, Bin; | 2021 | Perceived social support as a moderator of depression and stress in college students | Social Behavior and Personality | Mental health disorders |
| 1048 | Yang, X.;Zhu, J.;Hu, P.; | 2023 | Perceived social support and procrastination in college students: A sequential mediation model of self-compassion and negative emotions | Current Psychology | Mental health disorders |
| 1049 | Shelton, A. J.;Wang, C. D. C.;Zhu, W. Z.; | 2017 | Perceived Social Support and Mental Health: Cultural Orientations as Moderators | Journal of College Counseling | Mental health disorders |
| 1050 | Jibeen, T.; | 2016 | Perceived Social Support and Mental Health Problems Among Pakistani University Students | Community Mental Health Journal | Mental health disorders |
| 1051 | Lozano-Verduzco, I.;Vega-Cauich, J.;Mendoza-Pérez, J. C.;Craig, S. L.; | 2024 | Perceived Social Support and Mental Health Indicators of a Mexican LGBT Sample During the COVID-19 Pandemic | International Journal of Mental Health and Addiction | Mental health disorders |
| 1052 | Xu, QiongYing;Li, ShiFeng;Yang, Ling; | 2019 | Perceived social support and mental health for college students in mainland China: the mediating effects of self-concept | Psychology, health & medicine | Mental health disorders |
| 1053 | Reid, Gerald;Holt, Melissa;Bowman, Chelsey;Espelage, Dorothy;Green, Jennifer; | 2016 | Perceived Social Support and Mental Health among First-Year College Students with Histories of Bullying Victimization | Journal of Child and Family Studies | Mental health disorders |
| 1054 | Xin, Zhongyi; | 2022 | Perceived social support and college student engagement: moderating effects of a grateful disposition on the satisfaction of basic psychological needs as a mediator | BMC Psychology | Mental health disorders |
| 1055 | Iglesia, Guadalupe de la;Stover, Juliana Beatriz;Liporace, Mercedes Fernandez; | 2014 | Perceived social support and academic achievement in Argentinean college students | Europe's Journal of Psychology | Not mental health or wellbeing outcomes |

| | | | | | |
|---|---|---|---|---|---|
| 1056 | de la Iglesia, Guadalupe;Agustin Freiberg, Hoffmann;Mercedes Fernández, Liporace; | 2014 | Perceived parenting and social support: can they predict academic achievement in Argentinean college students? | Psychology Research and Behavior Management | Not related wellbeing |
| 1057 | Benau, E. M.;Jenkins, A. L.;Conner, B. T.; | 2017 | Perceived Parental Monitoring and Sexual Orientation Moderate Lifetime Acts of Non-Suicidal Self-Injury | Arch Suicide Res | Mental health disorders |
| 1058 | Nam, B.;Wilcox, H. C.;Hilimire, M.;DeVylder, J. E.; | 2018 | Perceived need for care and mental health service utilization among college students with suicidal ideation | J Am Coll Health | Mental health disorders |
| 1059 | Pike, C. K.;Burdick, K. E.;Millett, C.;Lipschitz, J. M.; | 2024 | Perceived loneliness and social support in bipolar disorder: relation to suicidal ideation and attempts | International Journal of Bipolar Disorders | Mental health disorders |
| 1060 | Roca-Campos, E.;Zubiri-Esnaola, H.;León-Jiménez, S.;Aubert, A.; | 2024 | Perceived Improvement of Literacy Skills of Students with and Without Special Educational Needs Through Dialogic Literary Gatherings | Disabilities | Not in a higher education context |
| 1061 | Blessing, A.;Russell, P.;DeBeer, B. B.;Morissette, S. B.; | 2023 | Perceived Family Support Buffers the Impact of PTSD-Depression Symptoms on Suicidal Ideation in College Students | Psychol Rep | Mental health disorders |
| 1062 | Liu, L.;Han, Y.;Lian, Y.;Wu, X.;Qiao, Z.;Wang, W.; | 2023 | Perceived discrimination and suicidal ideation among impoverished and non impoverished college students: Different mechanisms via social support, depressive symptoms, and nonsuicidal self-injury | Suicide Life Threat Behav | Mental health disorders |
| 1063 | Schmidt, Christa K.;Miles, Joseph R.;Welsh, Anne C.; | 2011 | Perceived Discrimination and Social Support: The Influences on Career Development and College | Journal of Career Development | Not related wellbeing |

| | | | | | |
|---|---|---|---|---|---|
| | | | Adjustment of LGBT College Students | | |
| 1064 | Morton, S. C. M.;Everhart, R.;Dautovich, N.;Chukmaitov, A.; | 2023 | Perceived discrimination and mental health outcomes in college students: the mediating effect of preventive health behaviors and social support | J Am Coll Health | Mental health disorders |
| 1065 | Pebole, M. M.;Singleton, C. R.;Hall, K. S.;Petruzzello, S. J.;Alston, R.;Gobin, R. L.; | 2024 | Perceived Barriers and Benefits of Exercise Among Women Survivors of Sexual Violence by Physical Activity Level and Posttraumatic Stress Disorder Status | Violence Against Women | Mental health disorders |
| 1066 | Malinauskas, R. K.;Saulius, T.; | 2022 | Perceived Academic Stress and Social Support among University Undergraduate Students During COVID-19 Pandemic | European Journal of Contemporary Education | Mental health disorders |
| 1067 | Watt, T.;Kim, S.;Ceballos, N.;Norton, C.; | 2022 | People who need people: the relationship between adverse childhood experiences and mental health among college students | J Am Coll Health | The population is not college students |
| 1068 | Hecht, C. A.;Latham, A. G.;Buskirk, R. E.;Hansen, D. R.;Yeager, D. S.; | 2022 | Peer-Modeled Mindsets: an Approach to Customizing Life Sciences Studying Interventions | CBE life sciences education | Mental health disorders |
| 1069 | Pueyo-Garrigues, S.;Pardavila-Belio, M. I.;Pueyo-Garrigues, M.;Canga-Armayor, N.; | 2023 | Peer-led alcohol intervention for college students: a pilot randomized controlled trial | Nursing & health sciences | Mental health disorders |
| 1070 | Roberson, A. A.;McKinney, C.;Walker, C.;Coleman, A.; | 2018 | Peer, social media, and alcohol marketing influences on college student drinking | J Am Coll Health | Mental health disorders |
| 1071 | Dia, M.;Khodabandelou, G.;Othmani, A.; | 2024 | Paying attention to uncertainty: A stochastic multimodal transformer for post-traumatic stress disorder detection using video | Computer Methods and Programs in Biomedicine | Mental health disorders |
| 1072 | Han, F. F.;Ellis, R. A.;Guan, E. J.; | 2023 | Patterns of students' collaborations by variations in | Journal of | Not mental health or |

| | | | | | |
|---|---|---|---|---|---|
| | | | their learning orientations in blended course designs: How is it associated with academic achievement? | Computer-Assisted Learning | wellbeing outcomes |
| 1073 | Wan, J.;Cao, C.;Fang, R.;Chen, C.;Wang, L.; | 2024 | Patterns and transitions of posttraumatic stress symptoms and posttraumatic growth in trauma-exposed youth: A latent transition analysis | Stress and Health | Mental health disorders |
| 1074 | Kaggwa, M. M.;Abaatyo, J.;Opiro, K.;Sikoti, M.;Bongomin, F.; | 2024 | Patterns and outcomes of individuals admitted at emergency units following intentional self-harm in Northern Uganda | Discover Mental Health | Not related wellbeing |
| 1075 | Arch, J. J.;Kirk, M. H.;Finkelstein, L. B.; | 2024 | Patient-Reported Worst-Case Scenarios in Advanced Cancer: Presence, Contents, and Predictors | Psycho-Oncology | Not related wellbeing |
| 1076 | Kim, E. J.;Yu, J. H.;Kim, E. Y.; | 2020 | Pathways linking mental health literacy to professional help-seeking intentions in Korean college students | J Psychiatr Ment Health Nurs | Not related wellbeing |
| 1077 | Li, H.;Cai, J.;Chen, R.;Zhao, Z.;Ying, Z.;Wang, L.;Chen, J.;Hao, K.;Kinney, P. L.;Chen, H.;et al.,; | 2017 | Particulate Matter Exposure and Stress Hormone Levels: A randomized, Double-blind, Crossover Trial of Air Purification | Circulation | Mental health disorders |
| 1078 | Abplanalp, S. J.;Mote, J.;Uhlman, A. C.;Weizenbaum, E.;Alvi, T.;Tabak, B. A.;Fulford, D.; | 2022 | Parsing social motivation: development and validation of a self-report measure of social effort | J Ment Health | Not mental health or wellbeing outcomes |
| 1079 | Altpeter, A.;Dixius, A.;Möhler, E.; | 2024 | Parents of Child Psychiatric Patients Report More Adverse Childhood Experiences Compared with Community Samples | Children | The population is not college students |
| 1080 | Hadar, T.; | 2024 | Parenting in the Face of Trauma: Music Therapy to Support Parent–Child Dyads Affected by War | Children | The population is not college students |

| | | | | | |
|---|---|---|---|---|---|
| | | | and Displacement | | |
| 1081 | Guerrero, N.;Ouyang, F.;Monahan, P.;Brown, S. A.;Zapolski, T. C.;Aalsma, M. C.; | 2024 | Parental support in adolescence: A potential moderator of the relationship between racial discrimination and adult suicidality | Public Health | Mental health disorders |
| 1082 | Yu, S.;Zhang, C.;Wang, Y.;Liu, T.;Chen, X.;Guo, J.;Zhang, G.;Xu, W.; | 2023 | Parental neglect, anxious attachment, perceived social support, and mental health among Chinese college students with left-behind experience: A longitudinal study. | Psych J | Mental health disorders |
| 1083 | Riggio, H. R.;Valenzuela, A. M.; | 2011 | Parental marital conflict and divorce, parent-child relationships, and social support among Latino-American young adults | Personal Relationships | The population is not college students |
| 1084 | Fingerman, K. L.;Cheng, Y. P.;Kim, K.;Fung, H. H.;Han, G.;Lang, F. R.;Lee, W.;Wagner, J.; | 2016 | Parental Involvement with College Students in Germany, Hong Kong, Korea, and the United States | J Fam Issues | Mental health disorders |
| 1085 | Serido, Joyce;Li, Lijun;Vosylis, Rimantas;Vasquez, Katherine;Sorgente, Angela;Lep, Žan;Fonseca, Gabriela;Crespo, Carla;Relvas, Ana Paula;Zupančič, Maja;Lanz, Margherita; | 2025 | Parental financial support and family emotional support to young adults during COVID-19: A help or a hindrance? | Family Process | The population is not college students |
| 1086 | Trujillo, Natasha P.;Servaty-Seib, Heather; | 2018 | Parental Absence and Non-Suicidal Self-Injury: Social Support, Social Constraints and Sense-Making | Journal of Child and Family Studies | Mental health disorders |
| 1087 | Schaefer, M. R.;Wagoner, S. T.;Young, M. E.;Kavookjian, J.;Shapiro, S. K.;Gray, W. N.; | 2018 | Parent Perceptions of Their College Students' Self-Management of Attention-Deficit/Hyperactivity Disorder | J Adolesc Health | Mental health disorders |

| | | | | | |
|---|---|---|---|---|---|
| 1088 | Jafari, A.;Tehrani, H.;Naddafi, F.;Nejatian, M.;Talebi, M.; | 2024 | Page: investigating the predictors of general psychological help-seeking intention among people who attempted suicide by using structural equation modeling | BMC Psychology | Mental health disorders |
| 1089 | Zhang, J.;Zhou, C.;Yu, R.; | 2020 | Oxytocin amplifies the influence of good intentions on social judgments | Hormones and behavior | Mental health disorders |
| 1090 | Norton, B.;Sheen, J.;Burns, L.;Enticott, P. G.;Fuller-Tyszkiewicz, M.;Kirkovski, M.; | 2024 | Overlap of eating disorders and neurodivergence: the role of inhibitory control | BMC Psychiatry | Mental health disorders |
| 1091 | Mancini, A. D.;Westphal, M.;Griffin, P.; | 2021 | Outside the Eye of the Storm: Can Moderate Hurricane Exposure Improve Social, Psychological, and Attachment Functioning? | Pers Soc Psychol Bull | Mental health disorders |
| 1092 | Hertweck, S. P.;Ziegler, C. H.;Logsdon, M. C.; | 2010 | Outcome of exposure to community violence in female adolescents | J Pediatr Adolesc Gynecol | Published not from January 2010 to 31 March 2024 |
| 1093 | Kroska, E. B.;Hoel, S.;Victory, A.;Murphy, S. A.;McInnis, M. G.;Stowe, Z. N.;Cochran, A.; | 2020 | Optimizing an Acceptance and Commitment Therapy Microintervention Via a Mobile App With Two Cohorts: protocol for Micro-Randomized Trials | JMIR research protocols | Mental health disorders |
| 1094 | Li, Y.;Ye, Y.;Zhou, X.; | 2024 | Optimism, Posttraumatic Stress Disorder, and Posttraumatic Growth Among Adolescents: A Longitudinal Analysis of the Mediating Effects of Adversity Belief and Affect | Journal of Happiness Studies | Mental health disorders |
| 1095 | Demirtas, A. S.; | 2020 | Optimism and happiness in undergraduate students: Cognitive flexibility and adjustment to university life as mediators | Anales De Psicologia | Not in a higher education context |

| | | | | | |
|---|---|---|---|---|---|
| 1096 | Shafna, V.;S.D, M. K.; | 2024 | Optimal interval and feature selection in activity data for detecting attention deficit hyperactivity disorder | Computers in Biology and Medicine | Mental health disorders |
| 1097 | King, C. A.;Eisenberg, D.;Zheng, K.;Czyz, E.;Kramer, A.;Horwitz, A.;Chermack, S.; | 2015 | Online suicide risk screening and intervention with college students: a pilot randomized controlled trial | Journal of consulting and clinical psychology | Mental health disorders |
| 1098 | Cole, D. A.;Nick, E. A.;Zelkowitz, R. L.;Roeder, K. M.;Spinelli, T.; | 2017 | Online social support for young people: Does it recapitulate in-person social support; can it help? | Computers in Human Behavior | Not in a higher education context |
| 1099 | Zhang, H. P.;Peng, S.;Li, S. Q.;Li, J. Z.;Yu, Q. L.; | 2023 | Online Social Support and Depressive Symptoms: Mediating Effect of Self-esteem and Gender Differences | International Journal of Mental Health and Addiction | Mental health disorders |
| 1100 | Velezmoro, R.;Negy, C.;Livia, J.; | 2012 | Online sexual activity: cross-national comparison between United States and Peruvian college students | Arch Sex Behav | Mental health disorders |
| 1101 | Meng, J.;Peng, W.;Shin, S. Y.;Chung, M.; | 2017 | Online Self-Tracking Groups to Increase Fruit and Vegetable Intake: a Small-Scale Study on Mechanisms of Group Effect on Behavior Change | Journal of medical Internet research | Not in a higher education context |
| 1102 | Walukevich-Dienst, K.;Neighbors, C.;Buckner, J. D.; | 2019 | Online personalized feedback intervention for cannabis-using college students reduces cannabis-related problems among women. | Addictive behaviors | Mental health disorders |
| 1103 | Wong, W. L.;Yuen, K. A.; | 2023 | Online Learning Stress and Chinese College Students' Academic Coping during COVID-19: The Role of Academic Hope and Academic Self-Efficacy | J Psychol | Mental health disorders |
| 1104 | Chang, T. F. H.;Ley, B. L.;Ramburn, | 2022 | Online Isha Upa Yoga for student mental health | Applied | Not in a higher education |

| | | | | | |
|---|---|---|---|---|---|
| | T. T.;Srinivasan, S.;Hariri, S.;Purandare, P.;Subramaniam, B.; | | and well-being during COVID-19: a randomized control trial | psychology. Health and well-being | context |
| 1105 | Jiang, Y.;Chi, J. M.;Wang, L.;Geng, X. M.; | 2023 | Online communication and positive psychological capital of college students in China: the mediating role of online social support | BMC Psychology | Mental health disorders |
| 1106 | Blair, J.;Luo, Y.;Ma, N. F.;Lee, S.;Choe, E. K.; | 2018 | OneNote Meal: a Photo-Based Diary Study for Reflective Meal Tracking | Amia ... Annual symposium proceedings. AMIA symposium | Mental health disorders |
| 1107 | Li, L.;Wang, H.;Lin, Y.;Li, X.; | 2023 | One single-person bicycling enhances interpersonal cooperation via increasing interpersonal neural synchronization in the left frontal cortex. | Human Brain Mapping | Mental health disorders |
| 1108 | D'Amico Guthrie, Deanna;Fruiht, Veronica; | 2020 | On-Campus Social Support and Hope as Unique Predictors of Perceived Ability to Persist in College | Journal of College Student Retention: Research, Theory & Practice | Mental health disorders |
| 1109 | McLean, K. C.;Pasupathi, M.; | 2011 | Old, new, borrowed, blue? The emergence and retention of personal meaning in autobiographical storytelling | Journal of Personality | Mental health disorders |
| 1110 | Xu, D.;Chen, B.;Yang, M.;Lin, G.;Zhang, M.;Wu, Z.;Zhou, H.;Shi, X.;Peng, Q.;Zeng, Y.;Lao, J.;Wang, Q.;Liang, S.;Li, J.;Yao, K.;Liu, Q.;Ou, Y.;Zhong, X.;Ning, Y.; | 2024 | Odor identification dysfunction in late-life depression with suicidal ideation | Journal of Affective Disorders | Mental health disorders |
| 1111 | Khoshakhlagh, A. H.;Al Sulaie, | 2024 | Occupational stress and musculoskeletal disorders | Scientific Reports | Mental health disorders |

| | | | | | |
|---|---|---|---|---|---|
| | S.;Mirzahosseininejad, M.;Yazdanirad, S.;Orr, R. M.;Laal, F.;Bamel, U.; | | in firefighters: the mediating effect of depression and job burnout | | |
| 1112 | Chiu, Y. H.;Lee, Y. H.;Wang, S. Y.;Ouyang, C. S.;Wu, R. C.;Yang, R. C.;Lin, L. C.; | 2024 | Objective approach to diagnosing attention deficit hyperactivity disorder by using pixel subtraction and machine learning classification of outpatient consultation videos | Journal of Neurodevelopmental Disorders | Mental health disorders |
| 1113 | Savage, L. R.;Couture Bue, A. C.; | 2023 | Objectifying the classroom: examining self-objectification and its effects on cognitive resources within virtual class environments. | Body image | Mental health disorders |
| 1114 | Singh, N.;Baumbach, C.;Compa, M.;Buczyłowska, D.;Bratkowski, J.;Mysak, Y.;Wierzba-Łukaszyk, M.;Sitnik-Warchulska, K.;Skotak, K.;Lipowska, M.;Izydorczyk, B.;Szwed, M.;Markevych, I.; | 2024 | Nurturing attention through nature | Environmental Research | Mental health disorders |
| 1115 | Kamal, K.;Sunita, S.;Karobi, D.;Abhishek, G.; | 2020 | Nurse-Delivered Screening and Brief Intervention Among College Students with Hazardous Alcohol Use: a Double-Blind Randomized Clinical Trial from India | Alcohol and alcoholism (Oxford, Oxfordshire) | Mental health disorders |
| 1116 | Mielicki, M. K.;Fitzsimmons, C. J.;Schiller, L. K.;Scheibe, D.;Taber, J. M.;Sidney, P. G.;Matthews, P. G.;Waters, E. A.;Coifman, K. G.;Thompson, C. A.; | 2023 | Number lines can be more effective at facilitating adults' performance on health-related ratio problems than risk ladders and icon arrays. | Journal of experimental psychology. Applied | The population is not college students. |
| 1117 | Tilstra-Ferrell, E. L.;Rheingold, A. | 2024 | Novel Application of Skills for Psychological | Cognitive and | Mental health disorders |

| | | | | | |
|---|---|---|---|---|---|
| | A.;Mai, K.;Hahn, C. K.; | | Recovery as an Early Intervention for Posttraumatic Stress Disorder, Depression, and Alcohol Misuse in Survivors of Recent Sexual Assault: A Case Series | Behavioral Practice | |
| 1118 | Rozzell, Bobby;Piercy, Cameron W.;Carr, Caleb T.;King, Shawn;Lane, Brianna L.;Tornes, Michael;Johnson, Amy Janan;Wright, Kevin B.; | 2014 | Notification pending: Online social support from close and nonclose relational ties via Facebook | Computers in Human Behavior | Mental health disorders |
| 1119 | Sattler, S.; | 2019 | Nonmedical use of prescription drugs for cognitive enhancement as a response to chronic stress especially when social support is lacking | Stress and Health | Mental health disorders |
| 1120 | Liu, Z.;Liu, Y.;Li, A.;Zhu, T.; | 2023 | Nonlinear effects of pandemic uncertainty on depression, pandemic preventive behavior intentions, and positive life attitudes: Moderating effects of high and low uncertainty grouping | Front Public Health | Mental health disorders |
| 1121 | Ramos-Martín, J.;Pérez-Berlanga, J. M.;Oliver, J.;Moreno-Küstner, B.; | 2023 | Non-lethal suicidal behavior in university students of Spain during COVID-19 | Front Psychiatry | Mental health disorders |
| 1122 | Jostrup, E.;Claesdotter-Knutsson, E.;Tallberg, P.;Söderlund, G.;Gustafsson, P.;Nyström, M.; | 2024 | No Effects of Auditory and Visual White Noise on Oculomotor Control in Children with ADHD | Journal of Attention Disorders | Mental health disorders |
| 1123 | Junco, R.;Cotten, S. R.; | 2012 | No, A 4 U: The relationship between multitasking and academic performance | Computers and Education | Mental health disorders |
| 1124 | Li, H. P.;Cheng, H. L.;Ding, K.;Zhang, Y.;Gao, F.;Zhu, G.;Zhang, Z.; | 2024 | New recognition of the heart-brain axis and its implication in the pathogenesis and treatment of PTSD | European Journal of Neuroscience | Mental health disorders |

| | | | | | |
|---|---|---|---|---|---|
| 1125 | Yu, Y.;Li, Y.;Li, T.;Xi, S.;Xiao, X.;Xiao, S.;Tebes, J. K.; | 2020 | New Path to Recovery and Well-Being: cross-Sectional Study on WeChat Use and Endorsement of WeChat-Based mHealth Among People Living With Schizophrenia in China | Journal of medical Internet research | Not in a higher education context |
| 1126 | Turel, Ofir;Natalie “Tasha”, Poppa;Gil-Or, Oren; | 2018 | Neuroticism Magnifies the Detrimental Association between Social Media Addiction Symptoms and Wellbeing in Women, but Not in Men: a three-Way Moderation Model. | Psychiatric quarterly | Mental health disorders |
| 1127 | Lookatch, S. J.;Fivecoat, H. C.;Moore, T. M.; | 2017 | Neuropsychological Effects of Placebo Stimulants on College Students | Journal of Psychoactive Drugs | Not related wellbeing |
| 1128 | Rahimpour Jounghani, A.;Gozdas, E.;Dacorro, L.;Avelar-Pereira, B.;Reitmaier, S.;Fingerhut, H.;Hong, D. S.;Elliott, G.;Hardan, A. Y.;Hinshaw, S. P.;Hosseini, S. M. H.; | 2024 | Neuromonitoring-guided working memory intervention in children with ADHD | iScience | Mental health disorders |
| 1129 | Lee, D. Y.;Byeon, G.;Kim, N.;Son, S. J.;Park, R. W.;Park, B.; | 2024 | Neuroimaging and natural language processing-based classification of suicidal thoughts in major depressive disorder | Translational Psychiatry | Mental health disorders |
| 1130 | Christensen, N.;Linden, M.;Muschalla, B.; | 2024 | Neurodevelopmental Impairments in Adult Psychosomatic Patients | Journal of Clinical Medicine | Mental health disorders |
| 1131 | Orak, S. A.;Bilaç, Ö;Polat, M.;Sobay, N. S.;Yalçin, A. H.;Korkmaz, R.;Kubur, Ç Ç;Atasever, A. K.;Yilmaz, C.;Özyurt, B. C.; | 2024 | Neurocognitive effects and electrophysiological findings in ADHD and self-limiting centrotemporal spike-wave epilepsy (SeLECTS) − A prospective tertiary care study | Epilepsy and Behavior | Mental health disorders |

| | | | | | |
|---|---|---|---|---|---|
| 1132 | Yu, Q.;Herold, F.;Ludyga, S.;Cheval, B.;Zhang, Z.;Mucke, M.;Kramer, A. F.;Li, J.;Kong, Z.;Zou, L.; | 2022 | Neurobehavioral mechanisms underlying the effects of physical exercise break on episodic memory during prolonged sitting. | Complementary therapies in clinical practice | Mental health disorders |
| 1133 | Song, X.;Liu, Q.;Zhang, X.;Liu, C.;Lan, C.;Zhang, X.;Xu, T.;Zhang, R.;Kendrick, K. M.;Becker, B.;Zhao, W.; | 2024 | Neural underpinnings of a two-phase memory suppression process in the neural response to self-related and observed perspective views | International Journal of Clinical and Health Psychology | Mental health disorders |
| 1134 | Dobbertin, M.;Blair, K. S.;Aloi, J.;Bajaj, S.;Bashford-Largo, J.;Mathur, A.;Zhang, R.;Carollo, E.;Schwartz, A.;Elowsky, J.;Ringle, J. L.;Tyler, P.;Blair, R. J.; | 2024 | Neural correlates of automatic emotion regulation and their association with suicidal ideation in adolescents during the first 90 days of residential care | Translational Psychiatry | Mental health disorders |
| 1135 | Kirk, R.;Watt, K. M.; | 2018 | Networks for Success: Preparing Mexican American AVID College Students for Credentials, Completion, and the Workforce | Journal of Latinos and Education | Mental health disorders |
| 1136 | Ruiz-Ordóñez, Y.;Sesé, A.; | 2024 | Network-based evidence of suicidal ideation among teachers | Scientific Reports | Mental health disorders |
| 1137 | Fung, V. S. C.;Chan, J. K. N.;Chui, E. M. C.;Wong, C. S. M.;Chu, R. S. T.;So, Y. K.;Chan, J. M. T.;Chung, A. K. K.;Lee, K. C. K.;Lo, H. K. Y.;Cheng, C. P. W.;Law, C. W.;Chan, W. C.;Chang, W. C.; | 2024 | Network analysis on psychopathological symptoms, psychological measures, quality of life, and COVID-19-related factors in Chinese psychiatric patients in Hong Kong | BMC Psychiatry | Not mental health or wellbeing outcomes |
| 1138 | Peng, L.;Zhang, J.;Li, M.;Li, P.;Zhang, Y.;Zuo, X.;Miao, Y.;Xu, Y.; | 2012 | Negative life events and mental health of Chinese medical students: the effect of resilience, personality, and social support | Psychiatry Res | Mental health disorders |

| | | | | | |
|---|---|---|---|---|---|
| 1139 | Liu, W.;Huang, J.;Lin, Y.;Cai, C.;Zhao, Y.;Teng, Y.;Mo, J.;Xue, L.;Liu, L.;Xu, W.;et al.,; | 2021 | Negative ions offset cardiorespiratory benefits of PM2.5 reduction from residential use of negative ion air purifiers. | Indoor air | Unpublished journal article |
| 1140 | Agnoli, S.;Mahncke, H.;Grant, S. J.;Goodman, Z. T.;Milberg, W. P.;Esterman, M.;DeGutis, J.; | 2024 | Negative Global Metacognitive Biases Are Associated With Depressive and Posttraumatic Stress Disorder Symptoms and Improve With Targeted or Game-Based Cognitive Training | Neuropsychology | Meta review |
| 1141 | Gao, Lingfeng;Chen, Yang;Yang, Xiujuan;Chu, Xiaowei;Liu, Qingqi;Zhou, Zongkui; | 2022 | Negative emotion and problematic mobile phone use: The mediating role of rumination and the moderating role of social support | Asian Journal of Social Psychology | Mental health disorders |
| 1142 | Fuse, A.; | 2018 | Needs of students seeking careers in communication sciences and disorders and barriers to their success | Journal of Communication Disorders | Mental health disorders |
| 1143 | Annamalai, N.;Foroughi, B.;Iranmanesh, M.;Buathong, S.; | 2020 | Needs and Facebook addiction: How important are psychological well-being and performance-approach goals? | Current Psychology | Mental health disorders |
| 1144 | Karaşar, B.;Baytemir, K.; | 2018 | Need for social approval and happiness in college students: The mediation role of social anxiety. | Universal Journal of Educational Research | Mental health disorders |
| 1145 | Fiset, J.; Robertson, M. C. S.; | 2023 | Navigating the support landscape: Bridging the divide between social support in business schools and student mental health | International Journal of Management Education | Mental health disorders |
| 1146 | Conley, Colleen S.;Shapiro, Jenna B.;Huguenel, Brynn M.;Kirsch, Alexandra C.; | 2020 | Navigating the College Years: Developmental Trajectories and Gender Differences in Psychological Functioning, Cognitive-Affective | Emerging Adulthood | Not related wellbeing |

| | | | | | |
|---|---|---|---|---|---|
| | | | Strategies, and Social Well-Being | | |
| 1147 | Anderson, A.;Lazarus, J.;Anderson Steeves, E.; | 2022 | Navigating Hidden Hunger: An Exploratory Analysis of the Lived Experience of Food Insecurity among College Students | Int J Environ Res Public Health | Not mental health or wellbeing outcomes |
| 1148 | Shrestha, T.;Di Blasi, Z.;Cassarino, M.; | 2021 | Natural or Urban Campus Walks and Vitality in University Students: exploratory Qualitative Findings from a Pilot Randomised Controlled Study | International journal of environmental research and public health | Mental health disorders |
| 1149 | Jo, H.;Park, J.;Lee, H.;Lee, K.;Lee, H.;Son, Y.;Kang, J.;Lee, S.;Choi, Y.;Lee, J. H.;Fond, G.;Boyer, L.;Smith, L.;Lee, J.;López Sánchez, G. F.;Dragioti, E.;Tully, M. A.;Rahmati, M.;Woo, H. G.;Woo, S.;Yon, D. K.; | 2024 | Nationwide trends in sadness, suicidal ideation, and suicide attempts among multicultural and monocultural adolescents in South Korea during the COVID-19 pandemic, 2011–2022 | World Journal of Pediatrics | Mental health disorders |
| 1150 | Kattih, M.;Lee, H.;Jo, H.;Jeong, J.;Kim, H.;Park, J.;Yang, H.;Nguyen, A.;Kim, H. J.;Lee, H.;Kim, M.;Lee, M.;Kwon, R.;Kim, S.;Koyanagi, A.;Kim, M. S.;Rahmati, M.;López Sánchez, G. F.;Dragioti, E.;Kim, J. H.;Woo, S.;Cho, S. H.;Smith, L.;Yon, D. K.; | 2024 | National prevalence of atopic dermatitis in Korean adolescents from 2009 to 2022 | Scientific Reports | Mental health disorders |
| 1151 | Shamsudeen, S.;Gupta, P.;Sayeed, N.;Munda, S. K.; | 2024 | Narrative exposure therapy for the treatment of trauma-related symptoms among adolescent | Indian Journal of Psychiatry | Not related wellbeing |

| | | | | | |
|---|---|---|---|---|---|
| | | | survivors of sex trafficking: A pilot study | | |
| 1152 | Paz, G. A.;Almeida, L.;Ruiz, L.;Casseres, S.;Xavier, G.;Lucas, J.;Santana, H. G.;Miranda, H.;Bonnette, S.;Willardson, J.; | 2020 | Myoelectric Responses of Lower-Body Muscles Performing Squat and Lunge Exercise Variations Adopting Visual Feedback With a Laser Sensor | Journal of Sport Rehabilitation | Not related wellbeing |
| 1153 | Demir, M.;Haynes, A.;Potts, S. K.; | 2017 | My Friends Are My Estate: Friendship Experiences Mediate the Relationship Between Perceived Responses to Capitalization Attempts and Happiness | Journal of Happiness Studies | Not mental health or wellbeing outcomes |
| 1154 | Lee, M. W.;Yang, N. J.;Mok, H. K.;Yang, R. C.;Chiu, Y. H.;Lin, L. C.; | 2024 | Music and movement therapy improves quality of life and attention and associated electroencephalogram changes in patients with attention-deficit/hyperactivity disorder. | Pediatrics and Neonatology | Mental health disorders |
| 1155 | Taniguchi, E.;Lee, H. E.; | 2019 | Muscle Talk Online and Impression Formation Based on Body Type: comparisons Between Asian American and Caucasian American Males | American journal of men's health | Not related wellbeing |
| 1156 | Sharma, M.;Arora, S.; | 2024 | Multimodality model investigating the impact of brain atlases, connectivity measures, and dimensionality reduction techniques on Attention Deficit Hyperactivity Disorder diagnosis using resting state functional connectivity | Journal of Medical Imaging | Mental health disorders |
| 1157 | Young, J. R.;Polick, C. S.;Michael, A. M.;Dannhauer, M.;Galla, J. T.;Evans, M. K.;Troutman, A.;Kirby, A. C.;Dennis, M. F.;Papanikolas, C. W.;Deng, Z. D.;Moore, S. D.;Dedert, | 2024 | Multimodal smoking cessation treatment combining repetitive transcranial magnetic stimulation, cognitive behavioral therapy, and nicotine replacement in veterans with posttraumatic stress disorder: A feasibility | PLoS ONE | Mental health disorders |

| | | | | | |
|---|---|---|---|---|---|
| | E. A.;Addicott, M. A.;Appelbaum, L. G.;Beckham, J. C.; | | randomized controlled trial protocol | | |
| 1158 | Zhang, R.;Luo, Q.;Gong, J.;Chen, Z.;Wu, Z.;Li, S.;Zheng, Q.;Wu, X.;Lam, K. H.;Lin, D.; | 2023 | Multilevel spatial confinement of transition metal selenides porous microcubes for efficient and stable potassium storage | J Colloid Interface Sci | Meta review |
| 1159 | Yu, L.;Zhang, N.;Zhang, Y. Y.; | 2023 | Multilevel Analysis of the effects of participation, peer support, SRL, and course disciplines on academic performance in SPOCs | Interactive Learning Environments | Not in a higher education context |
| 1160 | Graupensperger, S.;Cadigan, J. M.;Einberger, C.;Lee, C. M.; | 2023 | Multifaceted COVID-19-Related Stressors and Associations with Indices of Mental Health, Well-being, and Substance Use Among Young Adults | Int J Ment Health Addict | The population is not college students |
| 1161 | Jamil, B.;Su, J.; | 2023 | Multidimensional social support and associations between COVID-19 stress and depressive/anxiety outcomes among Hispanic/Latinx and White first-year college students | Journal of American College Health | Mental health disorders |
| 1162 | Covarrubias, R.;Fryberg, S. A.; | 2015 | Movin' on up (to college): first-generation college students' experiences with family achievement guilt | Cultural diversity & ethnic minority psychology | Not related wellbeing |
| 1163 | Peng, J.;Li, A.;Zhu, Q.; | 2018 | Motor expertise interacts with physical enactment to enhance action memory | Journal of Sports Sciences | Mental health disorders |
| 1164 | Dionisio, J.;de Sá, C. D. S. C.;Lúcio, S.;de Almeida, G. N.;Cordovil, R.; | 2024 | Motor Competence in Autistic Children with Attention-Deficit Hyperactivity Disorder | Children | The population is not college students |
| 1165 | Sanders, M.;Balcanoff, S.; | 2022 | Motivations for volunteering in an adapted skiing program: implications for volunteer program development | Disabil Rehabil | Not related wellbeing |

| | | | | | |
|---|---|---|---|---|---|
| 1166 | Magill, M.;Mastroleo, N. R.;Apodaca, T. R.;Barnett, N. P.;Colby, S. M.;Monti, P. M.; | 2010 | Motivational interviewing with significant other participation Assessing therapeutic alliance and patient satisfaction and engagement | Journal of Substance Abuse Treatment | Published not from January 2010 to 31 March 2024 |
| 1167 | Meinzer, M. C.;Oddo, L. E.;Vasko, J. M.;Murphy, J. G.;Iwamoto, D.;Lejuez, C. W.;Chronis-Tuscano, A.; | 2021 | Motivational interviewing plus behavioral activation for alcohol misuse in college students with ADHD | Psychology of addictive behaviors | Mental health disorders |
| 1168 | Samendinger, S.;Bruneau, M.;Hill, C. R.;Rowe, T. A.; | 2021 | Motivation in Team Exergames: testing the Köhler Discrepancy Effect with a Software-Generated Partner During Plank Exercise | Games for Health Journal | Not related wellbeing |
| 1169 | Egbert, Nichole;Wright, Kevin B.;Zhang, Xueying; | 2023 | Motivated and able: when is Facebook used to seek friendship and social support? | Journal of American College Health | Mental health disorders |
| 1170 | Archer, C.;Kao, K. T.; | 2018 | Mother, baby, and Facebook make three: does social media provide social support for new mothers? | Media International Australia | Mental health disorders |
| 1171 | Nguyen, A. W.; | 2017 | Mosque-Based Social Support and Collective and Personal Self-Esteem Among Young Muslim American Adults | Race and Social Problems | The population is not college students |
| 1172 | Topper, Amelia Marcetti; | 2019 | More than a Number: A Capabilities Framework for Conceptualizing Community College Success | Teachers College Record | Mental health disorders |
| 1173 | Pang, H.;Qiao, Y. X.;Xiao, Y.;Hu, X.; | 2023 | More Happiness or Less Comparison? Unpacking Associations Between Life Satisfaction, Negative Comparison, and Mobile Social Networking Use Among Sojourning Students | Sage Open | Mental health disorders |
| 1174 | Liu, S.;Zhu, M.;Young, S. D.; | 2018 | Monitoring Freshman College Experience | JMIR Public Health | Mental health disorders |

| | | | | | |
|---|---|---|---|---|---|
| | | | Through Content Analysis of Tweets: Observational Study | Surveill | |
| 1175 | Chang, C.;Palermo, E.;Deswert, S.;Brown, A.;Nuske, H. J.; | 2023 | Money can't buy happiness: a randomized controlled trial of a digital mental health app with versus without financial incentives. | Digital health | Mental health disorders |
| 1176 | Karalunas, S. L.;Dude, J.;Figuracion, M.;Lane, S. P.; | 2024 | Momentary Dynamics Implicate Emotional Features in the ADHD Phenotype | Research on Child and Adolescent Psychopathology | Mental health disorders |
| 1177 | Hilgeman, M. M.;Cramer, R. J.;Kaniuka, A. R.;Robertson, R. A.;Bishop, T.;Wilson, S. M.;Sperry, H. A.;Lange, T. M.; | 2024 | Moderators of treatment outcomes for LGBTQ+ military veterans in the PRIDE in All Who Served health promotion group | PLoS ONE | Mental health disorders |
| 1178 | Küchler, A. M.;Kählke, F.;Bantleon, L.;Terhorst, Y.;Ebert, D. D.;Baumeister, H.; | 2023 | Moderators and mediators of change of an internet-based mindfulness intervention for college students: secondary analysis from a randomized controlled trial | Frontiers in digital health | Mental health disorders |
| 1179 | Nguyen, H. V.;Huang, H. C.;Wong, M. K.;Yang, Y. H.;Huang, T. L.;Teng, C. I.; | 2018 | Moderator Roles of Optimism and Weight Control on the Impact of Playing Exergames on Happiness: the Perspective of Social Cognitive Theory Using a Randomized Controlled Trial | Games for Health Journal | Mental health disorders |
| 1180 | Chen, X.;Ma, Y.;Wu, R.;Liu, X.; | 2021 | Moderating Roles of Social Support in the Association between Hope and Life Satisfaction among Ethnic Minority College Students in China | Int J Environ Res Public Health | Not related wellbeing |
| 1181 | Arai, T.;Fuji, K.;Yanagida, M.; | 2018 | Moderating effect of social support on the relationship between psychopathic traits and immoral behaviors | Shinrigaku Kenkyu | Not in a higher education context |

| | | | | | |
|---|---|---|---|---|---|
| 1182 | Johnson, M. L.;Taasoobshirazi, G.;Kestler, J. L.;Cordova, J. R.; | 2015 | Models and messengers of resilience: a theoretical model of college students' resilience, regulatory Strategy use, and academic achievement | Educational Psychology | Not related wellbeing |
| 1183 | Ojo, K. S.;Volkova, N. V.; | 2023 | Modeling Innovation competence profiles: the empowering roles of self-monitoring and resilience | BMC Psychol | Not related wellbeing |
| 1184 | Ai, Amy L.;Tice, Terrence N.;Lemieux, Catherine M.;Huang, Bu; | 2011 | Modeling the post-9/11 meaning-laden paradox: from deep connection and deep struggle to posttraumatic stress and growth | Archive for the Psychology of Religion | Mental health disorders |
| 1185 | Liu, B.;Wei, L.; | 2018 | Modeling social support on social media: Effect of publicness and the underlying mechanisms | Computers in Human Behavior | Not related wellbeing |
| 1186 | Lee, S.;Ahn, H. Y.; | 2020 | Model Construction for Undergraduate Student College Adjustment | Int J Environ Res Public Health | Not related wellbeing |
| 1187 | Prokhorov, A. V.;Khalil, G. E.;Calabro, K. S.;Machado, T. C.;Russell, S.;Czerniak, K. W.;Botello, G. C.;Chen, M.;Perez, A.;Vidrine, D. J.;et al.,; | 2018 | Mobile Phone Text Messaging for Tobacco Risk Communication Among Young Adult Community College Students: Protocol and Baseline Overview for a Randomized Controlled Trial | JMIR research protocols | Not related wellbeing |
| 1188 | Yang, L. L.;Guo, C.;Li, G. Y.;Gan, K. P.;Luo, J. H.; | 2023 | Mobile phone addiction and mental health: the roles of sleep quality and perceived social support | Frontiers in Psychology | Mental health disorders |
| 1189 | Tran, D. T.;Martinez, I.;Cross, C. L.;Earley, Y. F.; | 2022 | MOBILE Intervention Pilot Study in College Students With Elevated Blood Pressure | Journal of Cardiovascular Nursing | Mental health disorders |
| 1190 | Kietrys, D. M.;Gerg, M. J.;Dropkin, J.;Gold, J. E.; | 2015 | Mobile input device type, texting style, and screen size influence upper extremity and trapezius muscle activity, and cervical posture while texting | Applied ergonomics | Mental health disorders |

| | | | | | |
|---|---|---|---|---|---|
| 1191 | Babalola, O.;Van Hedger, K.;Van Hedger, S. C.; | 2024 | Misophonia is associated with heightened emotion evocation by music | Current Psychology | Mental health disorders |
| 1192 | Lee, B.;Pescosolido, B. A.; | 2024 | Misery Needs Company: Contextualizing the Geographic and Temporal Link between Unemployment and Suicide | American Sociological Review | Mental health disorders |
| 1193 | Fujii, H.;Honoki, K.;Ishihara, T.;Shinomiya, T.;Tsukamoto, S.;Kido, A.;Kondoh, Y.;Kishi, S.;Shima, M.;Tanaka, Y.; | 2019 | Miscorrelation of Functional Outcome and Sociooccupational Status of Childhood, Adolescent, and Young Adult Generation With Bone and Soft Tissue Sarcoma Patients | J Pediatr Hematol Oncol | The population is not college students. |
| 1194 | Li, Huijun;Liu, Xiaoling;Zheng, Qingyong;Zeng, Siyuan;Luo, Xiaofeng; | 2023 | Minority stress, social support and mental health among lesbian, gay, and bisexual college students in China: a moderated mediation analysis | BMC Psychiatry | Mental health disorders |
| 1195 | Edwards, K. M.;Siller, L.;Littleton, H.;Wheeler, L.;Chen, D.;Sall, K.;Lim, S.; | 2021 | Minority Stress and Sexual Partner Violence Victimization and Perpetration Among LGBQ+ College Students: The Moderating Roles of Hazardous Drinking and Social Support | Psychology of Violence | Mental health disorders |
| 1196 | Edwards, K. M.;Siller, L.;Littleton, H.;Wheeler, L.;Chen, D.;Sall, K.;Lim, S.; | 2021 | Minority Stress and Sexual Partner Violence Victimization and Perpetration Among LGBQ plus College Students: The Moderating Roles of Hazardous Drinking and Social Support | Psychology of Violence | Mental health disorders |
| 1197 | Fine, L. E.; | 2011 | Minimizing heterosexism and homophobia: constructing the meaning of our campus LGB life | J Homosex | Mental health disorders |
| 1198 | Huang, C. L.;Shaw, F. F. T.;Hsu, W. Y.;Yu, H. T.;Chang, S. S.;Li, M. N.; | 2024 | Mindsets of suicide trajectories: An Linguistic Inquiry and Word Count analysis of suicide hotline conversations | Suicide and Life-Threatening Behavior | Mental health disorders |
| 1199 | Elzohairy, N. W.;Elzlbany, G. A. | 2024 | Mindfulness-based training effect on attention, | Archives of | Mental health disorders |

| | | | | | |
|---|---|---|---|---|---|
| | M.;Khamis, B. I.;El-Monshed, A. H.;Atta, M. H. R.; | | impulsivity, and emotional regulation among children with ADHD: The role of family engagement in randomized controlled trials | Psychiatric Nursing | |
| 1200 | Wingert, J. R.;Jones, J. C.;Swoap, R. A.;Wingert, H. M.; | 2022 | Mindfulness-based strengths practice improves well-being and retention in undergraduates: a preliminary randomized controlled trial. | Journal of American College Health | Mental health disorders |
| 1201 | Wilson, Jenna M.;Weiss, Audrey;Shook, Natalie J.; | 2020 | Mindfulness, self-compassion, and savoring: Factors that explain the relation between perceived social support and well-being | Personality and Individual Differences | Mental health disorders |
| 1202 | Kuhl, Megan;Boyraz, Güler; | 2017 | Mindfulness, General Trust, and Social Support Among Trauma-Exposed College Students | Journal of Loss & Trauma | Mental health disorders |
| 1203 | Zhang, N.;Fan, F. M.;Huang, S. Y.;Rodriguez, M. A.; | 2018 | Mindfulness training for loneliness among Chinese college students: a pilot randomized controlled trial | International Journal of Psychology | Mental health disorders |
| 1204 | Rosenstreich, E.; | 2016 | Mindfulness and False Memories: The Impact of Mindfulness Practice on the DRM Paradigm | Journal of Psychology | Mental health disorders |
| 1205 | Liu, C. M.;Wang, L. J.;Liu, Z.;Li, Y.;Yuan, G. Z.; | 2020 | Mindfulness and Cyberbullying Among Chinese Adolescents: The Mediating Roles of Perceived Social Support and Empathy | Violence and Victims | Mental health disorders |
| 1206 | Barrett, G. A.;Currin, H.; | 2024 | Mind Force Retreat: Improving the Subjective Well-being of Military Veterans Through Alternative Mental Health Therapies | Illness Crisis and Loss | Mental health disorders |
| 1207 | Kennedy, S. R.;Buck-Atkinson, J.;Moceri-Brooks, J.;Johnson, M. L.;Anestis, M. D.;Carrington, M.;Baker, J. C.;Fisher, M. E.;Nease, | 2024 | Military community engagement to prevent firearm-related violence: adaptation of project safeguard for service members | Injury Epidemiology | Mental health disorders |

| | | | | | |
|---|---|---|---|---|---|
| | D. E.;Bryan, A. O.;Bryan, C. J.;Betz, M. E.; | | | | |
| 1208 | Ivins, B.;Risling, M.;Wisén, N.;Schwab, K.;Rostami, E.; | 2024 | Mild Traumatic Brain Injury in the Maturing Brain: An Investigation of Symptoms and Cognitive Performance in Soldiers Returning From Afghanistan and Iraq | Journal of Head Trauma Rehabilitation | Mental health disorders |
| 1209 | Sajedi, S. S.;Navvabi-Rigi, S. D.;Navidian, A.; | 2024 | Midwifery-led brief counseling on the severity of posttraumatic stress symptoms of postpartum hemorrhage: quasi-experimental study | BMC Pregnancy and Childbirth | Mental health disorders |
| 1210 | Al-Rabiaah, A.;Temsah, M. H.;Al-Eyadhy, A. A.;Hasan, G. M.;Al-Zamil, F.;Al-Subaie, S.;Alsohime, F.;Jamal, A.;Alhaboob, A.;Al-Saadi, B.;et al.,; | 2020 | Middle East Respiratory Syndrome-Corona Virus (MERS-CoV) associated stress among medical students at a university teaching hospital in Saudi Arabia | Journal of infection and public health | Mental health disorders |
| 1211 | Williams, P. E.;Wall, N.;Fish, W. W.; | 2019 | Mid-Career Adult Learners in an Online Doctoral Program and the Drivers of Their Academic Self-Regulation: The Importance of Social Support and Parent Education Level | International Review of Research in Open and Distributed Learning | Mental health disorders |
| 1212 | Bonomi, R.;Hillmer, A. T.;Woodcock, E.;Bhatt, S.;Rusowicz, A.;Angarita, G. A.;Carson, R. E.;Davis, M. T.;Esterlis, I.;Nabulsi, N.;Huang, Y.;Krystal, J. H.;Pietrzak, R. H.;Cosgrove, K. P.; | 2024 | Microglia-mediated neuroimmune suppression in PTSD is associated with anhedonia. | Proceedings of the National Academy of Sciences of the United States of America | Mental health disorders |
| 1213 | Izadifar, M.;Massumi, M.;Prentice, | 2024 | Microfluidic chip systems for characterizing | Stem Cell Research | Mental health disorders |

| | | | | | |
|---|---|---|---|---|---|
| | K. J.;Oussenko, T.;Li, B.;Elbaz, J.;Puri, M.;Wheeler, M. B.;Nagy, A.; | | glucose-responsive insulin-secreting cells equipped with FailSafe kill-switch | and Therapy | |
| 1214 | Gonzales, L.;Davidoff, K. C.;Nadal, K. L.;Yanos, P. T.; | 2015 | Microaggressions experienced by persons with mental illnesses: An exploratory study | Psychiatr Rehabil J | Not in the higher education context |
| 1215 | Conover, K. J.;Israel, T.; | 2019 | Microaggressions and Social Support Among Sexual Minorities With Physical Disabilities | Rehabilitation Psychology | Mental health disorders |
| 1216 | Killoren, S. E.;Streit, C.;Alfaro, E. C.;Delgado, M. Y.;Johnson, N.; | 2017 | Mexican American college students' perceptions of youth success | Journal of Latina/o Psychology | Not related wellbeing |
| 1217 | Montoya-Londoño, D.;Landínez-Martínez, D.;Aguirre-Aldana, L.;Dussán-Lubert, C.;Partida-Gutierrez de Blume, A.; | 2024 | Metalinguistic and Reading Skills in a Sample of Colombian Children with Attention Deficit Hyperactivity Disorder | Children | Meta review |
| 1218 | James, A.;Adams-Huet, B.;Shah, M.; | 2015 | Menu labels displaying the kilocalorie content or the exercise equivalent: effects on energy ordered and consumed in young adults | American journal of health promotion | The population is not college students |
| 1219 | Hagler, M. A.; | 2023 | Mentoring first-generation college students: Examining distinct relationship profiles based on interpersonal characteristics, support provision, and educational capital | J Community Psychol | Not related wellbeing |
| 1220 | Taniguchi, Emiko;Thompson, Charee M.; | 2021 | Mental illness self-disclosure among college students: a pre-requisite of social support or a booster of social support benefits? | Journal of Mental Health | Not related wellbeing |
| 1221 | Webb, J. R.;Robinson, E. A. R.;Brower, K. J.; | 2011 | Mental Health, Not Social Support, Mediates the Forgiveness-Alcohol Outcome Relationship | Psychology of addictive behaviors | Mental health disorders |

| | | | | | |
|---|---|---|---|---|---|
| 1222 | Bottomley, J. S.;Abrutyn, S.;Smigelsky, M. A.;Neimeyer, R. A.; | 2017 | Mental Health Symptomatology and Exposure to Non-Fatal Suicidal Behavior: Factors That Predict Vulnerability and Resilience Among College Students | Arch Suicide Res | Mental health disorders |
| 1223 | Kirschner, B.;Goetzl, M.;Curtin, L.; | 2022 | Mental health stigma among college students: test of an interactive online intervention | Journal of American College Health | Mental health disorders |
| 1224 | Wu, M.;Xu, W.;Yao, Y.;Zhang, L.;Guo, L.;Fan, J.;Chen, J.; | 2020 | Mental health status of students' parents during COVID-19 pandemic and its influence factors | Gen Psychiatr | Not related wellbeing |
| 1225 | Torres, C.;Otero, P.;Bustamante, B.;Blanco, V.;Díaz, O.;Vázquez, F. L.; | 2017 | Mental Health Problems and Related Factors in Ecuadorian College Students | Int J Environ Res Public Health | Mental health disorders |
| 1226 | Ma, Z.;Zhao, J.;Li, Y.;Chen, D.;Wang, T.;Zhang, Z.;Chen, Z.;Yu, Q.;Jiang, J.;Fan, F.;Liu, X.; | 2020 | Mental health problems and correlates among 746 217 college students during the coronavirus disease 2019 outbreak in China | Epidemiol Psychiatr Sci | Mental health disorders |
| 1227 | Zhao, S.;Zhang, J.;Peng, L.;Yang, W.; | 2021 | Mental Health Outcomes among Chinese College Students over a Decade | Int J Environ Res Public Health | Not related wellbeing |
| 1228 | Fu, W.;Yan, S.;Zong, Q.;Anderson-Luxford, D.;Song, X.;Lv, Z.;Lv, C.; | 2021 | Mental health of college students during the COVID-19 epidemic in China | J Affect Disord | Not related wellbeing |
| 1229 | Kovess-Masfety, V.;Leray, E.;Denis, L.;Husky, M.;Pitrou, I.;Bodeau-Livinec, F.; | 2016 | Mental health of college students and their non-college-attending peers: results from a large French cross-sectional survey | BMC Psychol | Not related wellbeing |
| 1230 | Lei, X.;Liu, C.;Jiang, H.; | 2021 | The mental health of college students and associated factors in Hubei China | PLoS ONE | Mental health disorders |

| | | | | | |
|---|---|---|---|---|---|
| 1231 | Tandon, T.;Piccolo, M.;Ledermann, K.;McNally, R. J.;Gupta, R.;Morina, N.;Martin-Soelch, C.; | 2024 | Mental health markers and protective factors in students with symptoms of physical pain across WEIRD and non-WEIRD samples – a network analysis | BMC Psychiatry | Mental health disorders |
| 1232 | Johnson, A. P.;Lester, R. J.; | 2022 | Mental health in academia: Hacks for cultivating and sustaining wellbeing | American Journal of Human Biology | Not related to research questions |
| 1233 | Lian, Z.;Wallace, B. C.;Fullilove, R. E.; | 2020 | Mental Health Help-Seeking Intentions Among Chinese International Students in the US Higher Education System: The Role of Coping Self-Efficacy, Social Support, and Stigma for Seeking Psychological Help | Asian American Journal of Psychology | Mental health disorders |
| 1234 | Tahara, M.;Mashizume, Y.;Takahashi, K.; | 2021 | Mental Health Crisis and Stress Coping among Healthcare College Students Momentarily Displaced from Their Campus Community Because of COVID-19 Restrictions in Japan | Int J Environ Res Public Health | Mental health disorders |
| 1235 | Miller, R. L.;Moran, M.;Lucas-Thompson, R. G.;Sanchez, N.;Seiter, N.;Rayburn, S.;Verros, M.;Haddock, S. A.;Zimmerman, T. S.;Johnson, S. A.;et al.,; | 2022 | Mental health and health behaviors among college student mentors in a randomized controlled trial interrupted by COVID-19 | Journal of American College Health | Mental health disorders |
| 1236 | Bíró, É;Adány, R.;Kósa, K.; | 2011 | Mental health and behavior of students of public health and their correlation with social support: a cross-sectional study | BMC Public Health | Not related wellbeing |
| 1237 | Li, Y.;Zhao, J.;Ma, Z.;McReynolds, L. S.;Lin, D.;Chen, Z.;Wang, T.;Wang, D.;Zhang, Y.;Zhang, | 2021 | Mental Health Among College Students During the COVID-19 Pandemic in China: A 2-Wave Longitudinal Survey | J Affect Disord | Not related wellbeing |

| | | | | | |
|---|---|---|---|---|---|
| | J.;Fan, F.;Liu, X.; | | | | |
| 1238 | Siraji, A.;Molla, A.;Ayele, W. M.;Kebede, N.; | 2022 | Mental distress and associated factors among college students in Kemisie district, Ethiopia | Sci Rep | Mental health disorders |
| 1239 | Scarabelot, L. F.;Orellana, J. D. Y.;Cardoso, V. C.;Barbieri, M. A.;Cavalli, R. C.;Bettiol, H.;Horta, B. L.;Del-Ben, C. M.; | 2024 | Mental disorders in adults from Ribeirão Preto, Brazil: a cross-sectional analysis of two birth cohorts | BMC Public Health | The population is not college students |
| 1240 | Ezerceli, Ö;Dehkharghani, R.; | 2024 | Mental disorder and suicidal ideation detection from social media using deep neural networks | Journal of Computational Social Science | Mental health disorders |
| 1241 | Mathe, J. R.;Kelly, W. E.; | 2023 | Mental Boundaries Relationship with Self-Esteem and Social Support: New Findings for Mental Boundaries Research | Imagination, Cognition and Personality | Not related wellbeing |
| 1242 | Adler, R. H.; | 2024 | Men, masculinities, and mental health: Key issues for nurse practitioners | Journal of the American Association of Nurse Practitioners | Not related wellbeing |
| 1243 | Lagacé-Séguin, D. G.;DeLeavey, A. E.; | 2011 | Memories of adolescence: Can perceptions of social support be predicted from Baumrind's traditional parenting typologies? | International Journal of Adolescence and Youth | Not related wellbeing |
| 1244 | Smeets, E.;Neff, K.;Alberts, H.;Peters, M.; | 2014 | Meeting suffering with kindness: effects of a brief self-compassion intervention for female college students | Journal of Clinical Psychology | Mental health disorders |
| 1245 | Mao, X.;Fei, Y.;Deng, W.;Zhang, F.;Zhang, J.;Ni, C.;Hou, T.; | 2024 | Mediating Role of Resilience between Social Support and PTSD of Nursing Staff during Public | American Journal of Health Behavior | Mental health disorders |

| | | | | | |
|---|---|---|---|---|---|
| | | | Health Emergency in China: A Structural Equation Model | | |
| 1246 | Walter, O.;Hazan-Liran, B.; | 2022 | The mediating role of psychological capital in relations between social support and subjective well-being among students with learning disabilities and attention deficit hyperactivity disorder | European Journal of Special Needs Education | Mental health disorders |
| 1247 | Zhang, J.;Chen, Z. K.;Triatin, R. D.;Snieder, H.;Thio, C. H. L.;Hartman, C. A.; | 2024 | Mediating pathways between attention deficit hyperactivity disorder and type 2 diabetes mellitus: Evidence from a two-step and multivariable Mendelian randomization study | Epidemiology and Psychiatric Sciences | Mental health disorders |
| 1248 | Matel-Anderson, Denise M.;Bekhet, Abir K.;Garnier-Villarreal, Mauricio; | 2019 | Mediating Effects of Positive Thinking and Social Support on Suicide Resilience | Western Journal of Nursing Research | Mental health disorders |
| 1249 | Ma, Z. W.;Quan, P.;Liu, T.; | 2014 | MEDIATING EFFECT OF SOCIAL SUPPORT ON THE RELATIONSHIP BETWEEN SELF-EVALUATION AND DEPRESSION | Social Behavior and Personality | Mental health disorders |
| 1250 | Xu, H.;Song, X.;Wang, S.;Zhang, S.;Xu, S.;Wan, Y.; | 2019 | Mediating Effect of Social Support in the Relationship Between Childhood Abuse and Non-Suicidal Self-Injury Among Chinese Undergraduates: The Role of Only-Child Status | Int J Environ Res Public Health | The population is not college students |
| 1251 | Meng, J.;Rheu, M. M. J.;Zhang, Y.;Dai, Y.;Peng, W.; | 2023 | Mediated Social Support for Distress Reduction: AI Chatbots vs. Human | Proceedings of the ACM on Human-Computer Interaction | Mental health disorders |
| 1252 | Ikizer, E. G.;Blanton, H.; | 2016 | Media coverage of "wise" interventions can reduce concern for the disadvantaged. | Journal of experimental | Mental health disorders |

| | | | | | |
|---|---|---|---|---|---|
| | | | | psychology. Applied | |
| 1253 | Prince, M. A.;Tyskiewicz, A. J.;Conner, B. T.;Parnes, J. E.;Shillington, A. M.;George, M. W.;Riggs, N. R.; | 2021 | Mechanisms of change in an adapted marijuana e-CHECKUP TO GO intervention on decreased college student cannabis use | Journal of Substance Abuse Treatment | Mental health disorders |
| 1254 | Hernandez, D.;Jacomino, G.;Swamy, U.;Donis, K.;Eddy, S. L.; | 2021 | Measuring supports from learning assistants that promote engagement in active learning: evaluating a novel Social Support instrument. | International Journal of Stem Education | Not related wellbeing |
| 1255 | Schwebel, F. J.;Chavez, J. G.;Pearson, M. R.; | 2023 | Measuring Readiness to Change Substance Use, Alcohol Use, and Cannabis Use: an Experimental Manipulation of Cognitive Effort | Substance use & misuse | Mental health disorders |
| 1256 | Kathem, S. H.;Al-Jumail, A. A.;Noor-Aldeen, M.;Najah, N.;Khalid, D. A.; | 2021 | Measuring depression and anxiety prevalence among Iraqi healthcare college students using hospital anxiety and depression scale | Pharm Pract (Granada) | Mental health disorders |
| 1257 | Datta, N.;Bidopia, T.;Datta, S.;Mittal, G.;Alphin, F.;Marsh, E. J.;Fitzsimons, G. J.;Strauman, T. J.;Zucker, N. L.; | 2020 | Meal skipping and cognition along a spectrum of restrictive eating | Eating Behaviors | Unpublished journal article |
| 1258 | Manago, Adriana M.;Taylor, Tamara;Greenfield, Patricia M.; | 2012 | Me and My 400 Friends: The Anatomy of College Students' Facebook Networks, Their Communication Patterns, and Well-Being | Developmental Psychology | Not related wellbeing |
| 1259 | Michalek, J. E.;Qtaishat, L.;von Stumm, S.;El Kharouf, A.;Dajani, R.;Hadfield, K.;Mareschal, I.; | 2024 | Maternal Trauma and Psychopathology Symptoms Affect Refugee Children’s Mental Health But Not Their Emotion Processing | Research on Child and Adolescent Psychopathology | The population is not college students |
| 1260 | Phillips, K. T.;Phillips, M. | 2015 | Marijuana use, craving, and academic motivation | Addictive behaviors | Not related wellbeing |

| | | | | | |
|---|---|---|---|---|---|
| | M.;Lalonde, T. L.;Tormohlen, K. N.; | | and performance among college students: an in-the-moment study | | |
| 1261 | Palace, M.;Zamazii, O.;Terbeck, S.;Bokszczanin, A.;Berezovski, T.;Gurbisz, D.;Szwejka, L.; | 2024 | Mapping the factors behind ongoing war stress in Ukraine-based young civilian adults | Applied Psychology: Health and Well-Being | The population is not college students |
| 1262 | Buelow, M. T.;Jungers, M. K.;Chadwick, K. R.; | 2019 | Manipulating the decision-making process: influencing a "gut" reaction | Journal of clinical and experimental neuropsychology | Not in a higher education context |
| 1263 | Moser, J. S.;Moran, T. P.;Leber, A. B.; | 2015 | Manipulating Attention to Nonemotional Distractors Influences State Anxiety: a Proof-of-Concept Study in Low- and High-Anxious College Students | Behavior therapy | Mental health disorders |
| 1264 | Chao, R. C. L.; | 2011 | Managing Stress and Maintaining Well-Being: Social Support, Problem-Focused Coping, and Avoidant Coping | Journal of Counseling and Development | Mental health disorders |
| 1265 | Chao, R. C. L.; | 2012 | Managing Perceived Stress among College Students: The Roles of Social Support and Dysfunctional Coping | Journal of College Counseling | Mental health disorders |
| 1266 | Liu, Y. X.;Usman, M.;Zhang, J. W.;Raza, J.;Gul, H.; | 2021 | Making Sense of Chinese Employees' Suicide Ideation: Does Meaning in Life Matter? | Omega-Journal of Death and Dying | Mental health disorders |
| 1267 | van Ee, E.;de Beijer, D.;Florisson, D.;Geuskens, F.; | 2024 | Making Sense of Change after Intensive Trauma Treatment: a mixed-methods Study into Adolescents' Experience of Efficacy | Child and Adolescent Psychiatry and Mental Health | Not related wellbeing |
| 1268 | Laiduc, G.;Covarrubias, R.; | 2022 | Making Meaning of the Hidden Curriculum: Translating Wise Interventions to Usher University | Translational Issues in Psychological | Mental health disorders |

| | | | | | |
|---|---|---|---|---|---|
| | | | Change | Science | |
| 1269 | Bellur, S.;Nowak, K. L.;Hull, K. S.; | 2015 | Make it our time: In-class multitaskers have lower academic performance | Computers in Human Behavior | Not related wellbeing |
| 1270 | Tilahun, W. M.;Wolde, H. F.;Gebreegziabher, Z. A.;Abebaw, W. A.;Simegn, M. B.;Tadesse, A. A.; | 2023 | Magnitude, relationship, and determinants of attention deficit hyperactivity disorder and depression among University of Gondar undergraduate students, Northwest Ethiopia, 2022: Non-recursive structural equation modeling | PLoS ONE | Mental health disorders |
| 1271 | Andrikopoulos, D.;Vassiliou, G.;Fatouros, P.;Tsirmpas, C.;Pehlivanidis, A.;Papageorgiou, C.; | 2024 | Machine learning-enabled detection of attention-deficit/hyperactivity disorder with multimodal physiological data: a case-control study | BMC Psychiatry | Mental health disorders |
| 1272 | Zhu, X. L.;Wang, F.;Geng, Y. G.; | 2021 | Machiavellianism on quality of life: The role of lifestyle, age, gender, social support | Personality and Individual Differences | Not in a higher education context |
| 1273 | Wang, M.;Xie, Z.;Wang, T.;Dong, S.;Ma, Z.;Zhang, X.;Li, X.;Yuan, Y.; | 2024 | Low-intensity transcranial ultrasound stimulation improves memory behavior in an ADHD rat model by modulating cortical functional network connectivity. | NeuroImage | Mental health disorders |
| 1274 | Christie, L.;Smith-Spark, J. H.;Teodorini, R. D.; | 2024 | Loss of Control Eating in Adults With Impulsive and/or Inattentive Tendencies | Brain and Behavior | The population is not college students |
| 1275 | Yan, R.;Wang, X.;Zhou, H.;Li, X.;Diao, Y.;Xing, Y.;Zhu, Y.;Jiang, X.;Sun, J.;Zhang, Z.;et al.,; | 2019 | Long-term Low-Dose Sucrose May Prevent Migraine: two Double-Blinded Randomized Controlled Pilot Trials. | Journal of oral & facial pain and headache | Not related wellbeing |
| 1276 | Klaiber, P.;Whillans, A. V.;Chen, F. S.; | 2018 | Long-Term Health Implications of Students' Friendship Formation during the Transition to | Appl Psychol Health Well-Being | Not related wellbeing |

| | | | | | |
|---|---|---|---|---|---|
| | | | University | | |
| 1277 | Yamaguchi, S.;Ojio, Y.;Ando, S.;Bernick, P.;Ohta, K.;Watanabe, K. I.;Thornicroft, G.;Shiozawa, T.;Koike, S.; | 2019 | Long-term effects of filmed social contact or internet-based self-study on mental health-related stigma: a 2-year follow-up of a randomized controlled trial | Social Psychiatry and Psychiatric Epidemiology | Not related wellbeing |
| 1278 | Berger, K.;Schiefner, F.;Rudolf, M.;Awiszus, F.;Junne, F.;Vogel, M.;Lohmann, C. H.; | 2024 | Long-term effects of doping with anabolic steroids during adolescence on physical and mental health | Orthopadie | Unpublished journal article |
| 1279 | Chen, Z.;Shen, S.;Dai, Q.; | 2023 | Long-term and short-term psycho-social predictors of early-adulthood depression: role of childhood trauma, neuroticism, social support, resilience, and life-events | Current Psychology | The population is not college students |
| 1280 | Wang, D.;Zhao, J.;Zhai, S.;Huang, S.;Yang, Z.;Pan, Y.;Liu, X.;Fan, F.; | 2022 | Longitudinal trajectories of insomnia symptoms among college students during the COVID-19 lockdown in China | J Psychosom Res | Not related wellbeing |
| 1281 | Wang, D.;Zhao, J.;Ross, B.;Ma, Z.;Zhang, J.;Fan, F.;Liu, X.; | 2022 | Longitudinal trajectories of depression and anxiety among adolescents during COVID-19 lockdown in China | J Affect Disord | Mental health disorders |
| 1282 | Glass, N. E.;Clough, A.;Messing, J. T.;Bloom, T.;Brown, M. L.;Eden, K. B.;Campbell, J. C.;Gielen, A.;Laughon, K.;Grace, K. T.;et al.,; | 2022 | Longitudinal Impact of the myPlan App on Health and Safety Among College Women Experiencing Partner Violence | Journal of Interpersonal Violence | Mental health disorders |
| 1283 | Huang, C. Q.;Tu, Y. X.;He, T.;Han, Z. M.;Wu, X. M.; | 2024 | Longitudinal exploration of online learning burnout: the role of social support and cognitive engagement | European Journal of Psychology of Education | Not related wellbeing |

| | | | | | |
|---|---|---|---|---|---|
| 1284 | Haardörfer, R.;Windle, M.;Fairman, R. T.;Berg, C. J.; | 2021 | Longitudinal changes in alcohol use and binge-drinking among young-adult college students: Analyses of predictors across system levels | Addict Behav | Mental health disorders |
| 1285 | Martinez-Torteya, C.;Ramírez Hernández, L. I.;la Garza, B. T. D.;Pérez Tello, K. P.;Díaz Delgado, B. A.; | 2024 | Longitudinal change in symptoms of depression, anxiety, and post-traumatic stress disorder during the COVID-19 pandemic among Mexican college students | International Journal of Psychology | Mental health disorders |
| 1286 | Wright, G.;Volodarsky, S.;Hecht, S.;Saxe, L.; | 2021 | Lonely in Lockdown: Predictors of Emotional and Mental Health Difficulties Among Jewish Young Adults during the COVID-19 Pandemic | Contemp Jew | The population is not college students |
| 1287 | Öztürk, A.;Kundakçi, N.; | 2021 | Loneliness, Perceived Social Support, and Psychological Resilience as Predictors of Internet Addiction: A Cross-Sectional Study with a Sample of Turkish Undergraduates | Psychiatry and Clinical Psychopharmacolog y | Mental health disorders |
| 1288 | Ganson, K. T.;Cuccolo, K.;Nagata, J. M.; | 2023 | Loneliness is associated with eating disorders among a national sample of U.S. college students during the COVID-19 pandemic. | J Am Coll Health | Mental health disorders |
| 1289 | Lisitsa, E.;Benjamin, K. S.;Chun, S. K.;Skalisky, J.;Hammond, L. E.;Mezulis, A. H.; | 2020 | LONELINESS AMONG YOUNG ADULTS DURING COVID-19 PANDEMIC: THE MEDIATIONAL ROLES OF SOCIAL MEDIA USE AND SOCIAL SUPPORT SEEKING | Journal of Social and Clinical Psychology | The population is not college students |
| 1290 | Cheng, C. F.;Kuo, Y. H.;Hsu, W. C.;Chen, C.;Pan, C. H.; | 2021 | Local and remote ischemic preconditioning improves sprint interval exercise performance in team sport athletes. | International journal of environmental research and public | Not related wellbeing |

| | | | | | |
|---|---|---|---|---|---|
| | | | | health | |
| 1291 | Maxwell, C.;Houghton, S.;Chapman, E.; | 2024 | Links Between Attention-Deficit/Hyperactivity Disorder Symptoms, Peer Relationships and Mental Health Outcomes in Western Australian Youth | Children | Mental health disorders |
| 1292 | Redmond, S. M.;Ash, A. C.;Li, H.;Zhang, Y.; | 2024 | Links Among Attention-Deficit/Hyperactivity Disorder Symptoms and Psycholinguistic Abilities Are Different for Children With and Without Developmental Language Disorder | American Journal of Speech-Language Pathology | The population is not college students |
| 1293 | Zhao, J. L.;Song, F. X.;Chen, Q.;Li, M.;Wang, Y. H.;Kong, F.; | 2018 | Linking shyness to loneliness in Chinese adolescents: The mediating role of core self-evaluation and social support | Personality and Individual Differences | Not related wellbeing |
| 1294 | Lee, Seungyoon;Chung, Jae Eun;Park, Namkee; | 2016 | Linking Cultural Capital With Subjective Well-Being and Social Support: The Role of Communication Networks | Social Science Computer Review | Not related wellbeing |
| 1295 | Abaid Ur, Rehman;Tariq Mehmood, Bhuttah;You, Xuqun; | 2020 | Linking Burnout to Psychological Well-being: The Mediating Role of Social Support and Learning Motivation | Psychology Research and Behavior Management | Not related wellbeing |
| 1296 | Agarwal, S.;Mewafarosh, R.; | 2021 | LINKAGE OF SOCIAL MEDIA ENGAGEMENT WITH FOMO AND SUBJECTIVE WELL-BEING | Journal of Content, Community, and Communication | Not related wellbeing |
| 1297 | Barcelos, A. M.;Latham-Green, T.;Barnes, R.;Gorton, H.;Gussy, M.;Henderson, C.;Khatri, M.;Knapp, P.;Solomon, J.; | 2024 | Lifeguard Pharmacy: The co-development of a new community pharmacy response service for people in danger from domestic abuse or suicidal ideation | International Journal of Pharmacy Practice | Mental health disorders |

| | | | | | |
|---|---|---|---|---|---|
| 1298 | Zhang, J.;Zhao, S.;Lester, D.;Zhou, C.; | 2014 | Life satisfaction and its correlates among college students in China: a test of social reference theory | Asian J Psychiatr | Not related wellbeing |
| 1299 | Gonzales, G.;de Mola, E. L.;Robertson, L.;Gavulic, K. A.;McKay, T.; | 2023 | LGBTQ College student health and wellbeing at the onset of the pandemic: additional evidence and lessons learned from COVID-19 | BMC Public Health | Mental health disorders |
| 1300 | Hridoy, M. T. A.;Saha, S. R.;Islam, M. M.;Uddin, M. A.;Mahmud, M. Z.; | 2024 | Leveraging web scraping and stacking ensemble machine learning techniques to enhance the detection of major depressive disorder from social media posts | Social Network Analysis and Mining | Mental health disorders |
| 1301 | Caporale-Berkowitz, N. A.; | 2022 | Let's teach peer support skills to all college students: Here's how and why | J Am Coll Health | Not related wellbeing |
| 1302 | Anghel, E.; | 2022 | Let’s do this together: Do the quantity and the quality of collaborative learning predict achievement among college students? | Active Learning in Higher Education | Not related wellbeing |
| 1303 | Travers, A.;Armour, C.;Hansen, M.;Cunningham, T.;Langdon, S.;Hyland, P.;Vallières, F.;McCarthy, A.;Walshe, C.; | 2020 | Lesbian, gay, or bisexual identity as a risk factor for trauma and mental health problems in Northern Irish students and the protective role of social support | European Journal of Psychotraumatolog y | Mental health disorders |
| 1304 | Devine, M. A.; | 2016 | Leisure-Time Physical Activity: Experiences of College Students With Disabilities | Adapt Phys Activ Q | Not related wellbeing |
| 1305 | Ha, J. Y.;Smith, A. C.; | 2019 | Legal access to alcohol and academic performance: Who is affected? | Economics of Education Review | Mental health disorders |
| 1306 | Scharf, M.;Mayseless, O.;Kivenson-Baron, I.; | 2011 | Leaving the Parental Nest: Adjustment Problems, Attachment Representations, and Social Support During the Transition from High School to Military Service | Journal of Clinical Child and Adolescent Psychology | The population is not college students |

| | | | | | |
|---|---|---|---|---|---|
| 1307 | McGregor, K. K.;Marshall, B. A.;Julian, S. K.;Oleson, J.; | 2019 | Learning While Playing: a Randomized Trial of Serious Games as a Tool for Word Mastery | Language, speech, and hearing services in schools | Not related wellbeing |
| 1308 | Hu, C. S.;Zhang, H.;Short, L. A.;Liu, M.;Huang, C.;Liang, Z.;Yang, Y.;Huang, M.;Xie, D.; | 2023 | Learning of a Classmate's Suicide Ideation Affects Emotions When Advising the Classmate and the Helpfulness of the Advice Provided | Archives of suicide research | Mental health disorders |
| 1309 | Davis, K.;Sridharan, H.;Koepke, L.;Singh, S.;Boiko, R.; | 2018 | Learning and Engagement in a Gamified Course: Investigating the Effects of Student Characteristics | Journal of Computer-Assisted Learning | Not related wellbeing |
| 1310 | Llamas, J. D.;Morgan Consoli, M. L.;Hendricks, K.;Nguyen, K.; | 2018 | Latino/ Freshman Struggles: Effects of Locus of Control and Social Support on Intragroup Marginalization and Distress | Journal of Latina/o Psychology | Mental health disorders |
| 1311 | Low, T. Q. Y.;Teerawichitchainan, B.;Zimmer, Z.;Toan, T. K.; | 2024 | Later-life Social Network Profiles of male war survivors in Vietnam: Implications for health behaviors | Social Science and Medicine | Not related wellbeing |
| 1312 | Turpin, R.;King-Marshall, E.;Dyer, T.; | 2023 | Latent syndemic profiles among sexual and gender minority college students and psychological distress amid the COVID-19 pandemic | J LGBT Youth | Mental health disorders |
| 1313 | Long, M.;Li, J.;Sun, Y.;Gai, Y.;Zhang, S.; | 2024 | Latent class analysis of post-traumatic disorder and post-traumatic growth among front-line healthcare professionals during the early outbreak of COVID-19 | Current Psychology | Mental health disorders |
| 1314 | Walters, C.;McDonald, E.;Sheers, C.;Hawkins, K.;Solich, H.;Anderson, J.;Simic, N.;Moore, D.;Stevenson, T.;Lawn, S.;Goodyear, | 2024 | Knowledge Families Hold: Co-Production and Co-Research With Mental Health Family Carers in Understanding Experiences During the COVID-19 Pandemic | Health Expectations | Not related wellbeing |

| | | | | | |
|---|---|---|---|---|---|
| | M.;Maghidman, M.;Petrakis, M.; | | | | |
| 1315 | Jiang, Q.;Kim, Y.;Choi, M.; | 2022 | Kinetic Effects of 6 Weeks' Pilates or Balance Training in College Soccer Players with Chronic Ankle Instability | International journal of environmental research and public health | Not related wellbeing |
| 1316 | Tang, W.;Shi, C.;Li, Y.;Tang, Z.;Yang, G.;Zhang, J.;He, L.; | 2024 | Keypoints-Based Multi-Cue Feature Fusion Network (MF-Net) for Action Recognition of ADHD Children in TOVA Assessment | Bioengineering | Mental health disorders |
| 1317 | Bassilios, B.;Dunt, D.;Krysinska, K.;Machlin, A.;Newton, D.;Currier, D.; | 2024 | Key informant perspectives of suicide prevention in Australia | BMC Public Health | Mental health disorders |
| 1318 | Shiroma, P. R.;Thuras, P.;Polusny, M. A.;Kehle-Forbes, S.;Disner, S.;Pardo, J. V.;Gilmore, C.;Tolly, B.;Voller, E.;McManus, E.;King, C.;Lipinski, A.;Eng, E.;Hawkinson, F.;Wang, G.; | 2024 | Ketamine-enhanced prolonged exposure therapy in veterans with PTSD: A randomized controlled trial protocol | Contemporary Clinical Trials | Mental health disorders |
| 1319 | Hovasapian, A.;Levine, L. J.; | 2018 | Keeping the magic alive: social sharing of positive life experiences sustains happiness | Cognition and Emotion | Not related wellbeing |
| 1320 | Walter, N.;Demetriades, S. Z.;Murphy, S. T.; | 2019 | Just a Spoonful of Sugar Helps the Messages Go Down: using Stories and Vicarious Self-Affirmation to Reduce E-Cigarette Use. | Health communication | Not related wellbeing |
| 1321 | Chiang, T. L.;Chen, C.;Hsu, C. H.;Lin, Y. C.;Wu, H. J.; | 2019 | Is the goal of 12,000 steps per day sufficient for improving body composition and metabolic syndrome? The necessity of combining exercise | BMC Public Health | Meta review |

| | | | | | |
|---|---|---|---|---|---|
| | | | intensity: a randomized controlled trial | | |
| 1322 | Wang, Q.;Rice, K. G.;Arana, F. G.;Wetstone, H.;Bunker, B.; | 2024 | Is social support beneficial after a breakup? A moderation model of social support, depression, emotional volatility and gender for college students during COVID-19 | Journal of Social and Personal Relationships | Mental health disorders |
| 1323 | Yichen, M.;Chuntian, L.; | 2024 | Is lifestyle a bridge between perceived social support and depression in Chinese university students? | Children and Youth Services Review | Mental health disorders |
| 1324 | Anderson, D. A.;Schaumberg, K.;Anderson, L. M.;Reilly, E. E.; | 2015 | Is the level of intuitive eating associated with plate side effects? | Eating Behaviors | Unpublished journal article |
| 1325 | Datu, J. A. D.;Yang, W. P.;Valdez, J. P. M.;Chu, S. K. W.; | 2018 | Is Facebook involvement associated with academic engagement among Filipino university students? A cross-sectional study | Computers & Education | Not related wellbeing |
| 1326 | Nishimura, R.;Menrai, K.;Kajihara, M.;Asaoka, S.; | 2023 | Is decision-making influenced by interactions between extended wakefulness and weak emotional stressors? An experimental study | Ind Health | Mental health disorders |
| 1327 | Ong, Chorng-Shyong;Lin, Michael Yu-Ching; | 2016 | Is being satisfied enough? Well-being and IT post-adoption behavior | Information Development | Not related wellbeing |
| 1328 | El-Khoury, F.;Ghezala, I. B.;Hatem, G.;Jaffal, Z.;Soares, A.;Yacini, L.;Duchesne, S.;Dommergues, M.;Bretelle, F.;Eudeline, S.;Hoffmann, P.;Masse-Navette, C.;Layachi, F.;Maurice, O.;de Careil, T. D. F.;Bardou, M.; | 2024 | IROND-L: study protocol for a French prospective, quasi-experimental, multicentre trial to examine the impact of a coordinated multidisciplinary approach for women victims of violence | BMJ Open | Mental health disorders |

| | | | | | |
|---|---|---|---|---|---|
| 1329 | Myers, A.;Halpern-Manners, A.;McLeod, J. D.; | 2024 | Invisible disabilities and college academic success: New evidence from a mediation analysis | Social Science Research | Not related wellbeing |
| 1330 | Pei, Y.;Fan, Y.;Kong, X.;Sun, H.;Zhou, J.;Wu, H.; | 2022 | Investigation of the Effectiveness of Traditional Breathing Therapy on Pulmonary Function in College Students with Obstructive Sleep Apnea | Contrast media & molecular imaging | Mental health disorders |
| 1331 | Lee, D.; | 2022 | Investigation of gender differences in multivariate associations between physical activity and psychological distress | J Am Coll Health | Mental health disorders |
| 1332 | Mao, X. L.;Chen, H. M.; | 2023 | Investigation of contemporary college student's mental health status and construction of a risk prediction model | World J Psychiatry | Unpublished journal article |
| 1333 | Shen, I. H.;Wang, W. E.;Ni, H. C.;Chen, C. L.; | 2024 | Investigating Working Memory Deficits in School-Age Children with Attention-Deficit/Hyperactivity Disorder: An Event-Related Potentials Study During Delayed-Match-to-Sample Task | Journal of Attention Disorders | The population is not college students |
| 1334 | Brandon, Laurel E.;Reis, Sally M.;McCoach, D. Betsy; | 2021 | Investigating the Success of Academically Talented Students with Financial Need: Pathways and Decisions of Jack Kent Cooke Scholars | International Journal for Talent Development and Creativity | Not related wellbeing |
| 1335 | Shen, H. J.;Ye, X. Z.;Zhang, J. L.;Huang, D. H.; | 2024 | Investigating the role of perceived emotional support in predicting learners' well-being and engagement mediated by motivation from a self-determination theory framework | Learning and Motivation | Not related wellbeing |
| 1336 | Oraibi, Omar;Somali, Mohammed;Daghriri, | 2024 | Investigating the Interrelationships Between Obesity, Academic Achievement, Physical | Materia Socio-Medica | Not related wellbeing |

| | | | | | |
|---|---|---|---|---|---|
| | Khaled;Alameer, Mohammed;Arishi, Mohamed;Sahli, Ali;Najmi, Atiah;Otaif, Faisal;Abueishah, Hanadi;Oraibi, Bassem;Alhazmi, Abdulhameed;Alhazmi, Luai; | | Activity, and Social Support Among Jazan University Students | | |
| 1337 | Cooper, B. R.;Hill, L. G.;Haggerty, K. P.;Skinner, M.;Bumpus, M. F.;Borah, P.;Casey-Goldstein, M.;Catalano, R.; | 2020 | Investigating the efficacy of a self-directed parenting intervention to reduce risky behaviors among college students: study protocol for a multi-arm hybrid type 2 randomized control trial | Contemporary clinical trial communications | Mental health disorders |
| 1338 | Lee, D.;Young, S. J.; | 2018 | Investigating the effects of behavioral change, social support, and self-efficacy in physical activity in a collectivistic culture: Application of Stages of Motivational Readiness for Change in Korean young adults | Prev Med Rep | The population is not college students |
| 1339 | Wang, S.;Esperança, J. P.;Yang, W.;Zhang, J. Z.; | 2023 | Investigating the Determinants of New Technology Entrepreneurial Performance: an Empirical Study with PLS-SEM and MGA | Journal of the Knowledge Economy | Not related wellbeing |
| 1340 | Kim, M. H.;Turecki, G.;Orri, M.; | 2024 | Investigating the contribution of childhood maltreatment to suicide attempt: A multivariable Mendelian randomization study | Psychiatry Research | Mental health disorders |
| 1341 | Uy, K. J. D.;Alenton, J. B. B.;Amparado, P. D. E.; | 2014 | Investigating Student Study Engagement among College Students across Year Levels 1 | Recoletos Multidisciplinary Research Journal | Not related wellbeing |
| 1342 | Setiawati, Y.;Hartopo, D.;Rabitho, F. D.;Chuanardi, W.; | 2024 | Investigating Attention Deficit Hyperactivity Disorder Symptoms, Emotional Dysregulation and Family Functioning in Children: A | Journal of the Korean Academy of Child and | The population is not college students |

| | | | | | |
|---|---|---|---|---|---|
| | | | Community-Based Study in Elementary Schools in Surabaya, Indonesia | Adolescent Psychiatry | |
| 1343 | Feng, C.;Qin, L.;Luo, Y.;Xu, P.; | 2020 | Intranasal vasopressin expedites dishonesty in women | Hormones and behavior | Not related wellbeing |
| 1344 | Cénat, J. M.;Jacob, G.;Guillaume, D.;Amédée, L. M.;Darius, W. P.;Farahi, S. M. M. M.;Clorméus, L. A.;Guerrier, M.;Hébert, M.; | 2024 | Intimate Partner Violence and posttraumatic stress disorder among adolescents and young adults in Haiti | Psychiatry Research | The population is not college students |
| 1345 | Muñoz, L. R.; | 2022 | Interventions to boost enrollment in nursing Doctor of Philosophy (PhD) programs | Nurs Outlook | Mental health disorders |
| 1346 | Shan, Y.;Ji, M.;Xie, W.;Li, R.;Qian, X.;Zhang, X.;Hao, T.; | 2022 | Interventions in Chinese Undergraduate Students' Mental Health: Systematic Review | Interact, J Med Res, | Mental health disorders |
| 1347 | Pardavila-Belio, M. I.;García-Vivar, C.;Pimenta, A. M.;Canga-Armayor, A.;Pueyo-Garrigues, S.;Canga-Armayor, N.; | 2015 | Intervention study for smoking cessation in Spanish college students: pragmatic randomized controlled trial | Addiction (Abingdon, England) | Mental health disorders |
| 1348 | Ezeudu, F. O.;Nwoji, I. H. N.;Dave-Ugwu, P. O.;Abaeme, D. O.;Ikegbunna, N. R.;Agugu, C. V.;Muoneke, M. N.;Alabi, A. O.;Nwefuru, B. C.; | 2020 | Intervention for burnout among Chemistry Education Undergraduates in Nigeria | Journal of International Medical Research | Mental health disorders |
| 1349 | Li, M.;Ren, Y.; | 2019 | Intervention Effects of Motivation Interviewing Chinese Modified on the Mental Health of College Students with Exercise Dependence | Psychiatric quarterly | Mental health disorders |
| 1350 | Zhang, Z.;Li, Y.;Sun, S.;Tang, Z.; | 2022 | Intervention Effect of Group Counseling Based on Positive Psychology on Psychological Crisis of | Computational intelligence and | Mental health disorders |

| | | | | | |
|---|---|---|---|---|---|
| | | | College Student | neuroscience | |
| 1351 | Santacrose, L. B.;Laurita, A. C.;Marchell, T. C.; | 2020 | Intervene: modeling Pro-Social Bystander Behavior in College Students through Online Video. | Health communication | Mental health disorders |
| 1352 | García-López, D.;Izquierdo, M.;Rodríguez, S.;González-Calvo, G.;Sainz, N.;Abadía, O.;Herrero, A. J.; | 2010 | Interset stretching does not influence the kinematic profile of consecutive bench-press sets. | Journal of strength and conditioning research | Published not from January 2010 to 31 March 2024 |
| 1353 | Zhou, Q.;Li, N.;Li, C. A.;Zhang, J.; | 2024 | Interpersonal Relationship and Suicide Attempt: The Role of Family and Social Relationship | Journal of nervous and mental disease | Mental health disorders |
| 1354 | Crocker, J.;Canevello, A.;Breines, J. G.;Flynn, H.; | 2010 | Interpersonal Goals and Change in anxiety and dysphoria in first-semester college students | J Pers Soc Psychol | Published not from January 2010 to 31 March 2024 |
| 1355 | Muehlenkamp, J.;Brausch, A.;Quigley, K.;Whitlock, J.; | 2013 | Interpersonal features and functions of nonsuicidal self-injury | Suicide Life Threat Behav | Mental health disorders |
| 1356 | Rafaeli, A. K.;Bar-Kalifa, E.;Verdeli, H.;Miller, L.; | 2021 | Interpersonal Counseling for College Students: pilot Feasibility and Acceptability Study | American journal of psychotherapy | Not related wellbeing |
| 1357 | Mailey, E. L.;Wójcicki, T. R.;Motl, R. W.;Hu, L.;Strauser, D. R.;Collins, K. D.;McAuley, E.; | 2010 | Internet-delivered physical activity intervention for college students with mental health disorders: A Randomized Pilot trial | Psychology, health & medicine | Published not from January 2010 to 31 March 2024 |
| 1358 | Kass, A. E.;Trockel, M.;Safer, D. L.;Sinton, M. M.;Cunning, D.;Rizk, M. T.;Genkin, B. H.;Weisman, H. L.;Bailey, J. O.;Jacobi, C.;et al.,; | 2014 | Internet-based preventive intervention for reducing eating disorder risk: a randomized controlled trial comparing guided with unguided self-help | Behavior research and therapy | Mental health disorders |
| 1359 | Guerrero, G.;Avila, D.;da Silva, F. J. M.;Pereira, A.;Fernández-Caballero, | 2023 | Internet-based identification of anxiety in university students using text and facial emotion | Internet Interv | Mental health disorders |

| | | | | | |
|---|---|---|---|---|---|
| | A.; | | analysis | | |
| 1360 | Wekullo, C. S.; | 2019 | International Undergraduate Student Engagement: Implications for Higher Education Administrators | Journal of International Students | Mental health disorders |
| 1361 | Mbous, Y. P. V.;Mohamed, R.;Rudisill, T. M.; | 2022 | International students challenges during the COVID-19 pandemic in a university in the United States: A focus group study | Curr Psychol | Not related wellbeing |
| 1362 | Chentsova, V. O.;Bravo, A. J.;Mezquita, L.;Pilatti, A.;Hogarth, L.; | 2023 | Internalizing symptoms, rumination, and problematic social networking site use: A cross-national examination among young adults in seven countries | Addict Behav | The population is not college students |
| 1363 | Finley, J. C. A.;Robinson, A. D.;VanLandingham, H. B.;Ulrich, D. M.;Phillips, M. S.;Soble, J. R.; | 2024 | Internalizing and somatic symptoms influence the discrepancy between subjective and objective cognitive difficulties in adults with ADHD who have valid and invalid test scores. | Journal of the International Neuropsychological Society | Mental health disorders |
| 1364 | Datta, N.;Bidopia, T.;Datta, S.;Mittal, G.;Alphin, F.;Herbert, B. M.;Marsh, E. J.;Fitzsimons, G. J.;Strauman, T. J.;Zucker, N. L.; | 2021 | Internal states and interoception along a spectrum of eating disorder symptomology | Physiology & behavior | Mental health disorders |
| 1365 | Sari, Winda Liftiana;Fakhruddiana, Fuadah; | 2019 | Internal Locus of Control, Social Support, and Academic Procrastination among Students in Completing the Thesis | International Journal of Evaluation and Research in Education | Not related wellbeing |
| 1366 | Joshi, V.;Nanavati, N.; | 2024 | Inter-class Correlation-based EEG Channel Selection for ADHD Classification | IAENG International | Mental health disorders |

| | | | | | |
|---|---|---|---|---|---|
| | | | | Journal of Computer Science | |
| 1367 | Jenkins, L.;Jeske, D.; | 2017 | Interactive Support Effects on Career Agency and Occupational Engagement Among Young Adults | Journal of Career Assessment | The population is not college students |
| 1368 | Fang, B. B.;Lu, F. J. H.;Gill, D. L.;Chiu, Y. H.;Cheng, Y. C.;Hsieh, M. H.;Zhang, Z.; | 2023 | Interactive effects of dispositional mindfulness and PETTLEP imagery training on basketball shooting performance: a randomized controlled trial | Psychology of Sport and Exercise | Mental health disorders |
| 1369 | Hoffman, H. G.; | 2021 | Interacting with virtual objects via embodied avatar hands reduces pain intensity and diverts attention. | Scientific Reports | Mental health disorders |
| 1370 | Hodis, M. A.;Sriramachandramurthy, R.;Sashittal, H. C.; | 2015 | Interact with me on my terms: a four-segment Facebook engagement framework for marketers. | Journal of Marketing Management | Not related wellbeing |
| 1371 | Seyfi, F.;Poudel, K. C.;Yasuoka, J.;Otsuka, K.;Jimba, M.; | 2013 | Intention to seek professional psychological help among college students in Turkey: influence of help-seeking attitudes | BMC Res Notes | Not related wellbeing |
| 1372 | Boisselier, N.;Soubelet, A.; | 2024 | Intellectual giftedness and early adversity: searching for the hidden factor | Current Psychology | Not related wellbeing |
| 1373 | Stadtfeld, C.;Vörös, A.;Elmer, T.;Boda, Z.;Raabe, I. J.; | 2019 | Integration in emerging social networks explains academic failure and success. | Proceedings of the National Academy of Sciences of the United States of America | Not related wellbeing |
| 1374 | Miranda, O.;Qi, X.;Brannock, M. D.;Whitworth, R.;Kosten, T. R.;Ryan, N. D.;Haas, G. L.;Kirisci, | 2024 | Integrating Drug Target Information in Deep Learning Models to Predict the Risk of Adverse Events in Patients with Comorbid Post-Traumatic | Biomedicines | Mental health disorders |

| | | | | | |
|---|---|---|---|---|---|
| | L.;Wang, L.; | | Stress Disorder and Alcohol Use Disorder | | |
| 1375 | Medenblik, A. M.;Moore, T. M.;Stuart, G. L.; | 2024 | Integrating brief exposure exercises to support cognitive processing therapy for treatment of PTSD: A case study | Clinical Case Studies | Mental health disorders |
| 1376 | Maqableh, M.;Hmoud, H. Y.;Jaradat, M.;Masa'deh, R.; | 2021 | Integrating an information systems success model with perceived privacy, perceived security, and trust: the moderating role of Facebook addiction | Heliyon | Mental health disorders |
| 1377 | Shewail, F.;Abdelmajeed, S.;Farouk, M.;Abdelmegeed, M.; | 2023 | Instrument-assisted soft tissue mobilization versus myofascial release therapy in the treatment of chronic neck pain: a randomized clinical trial | BMC musculoskeletal disorders | Mental health disorders |
| 1378 | Schultz, Beth E.;Corbett, Cynthia F.;Hughes, Ronda G.; | 2022 | Instrumental support: A conceptual analysis | Nursing Forum | Unpublished journal article |
| 1379 | George, A. J.;Johnson, V. N.;Emmons, E. M.;Wellum, M.;Rhea, E.; | 2023 | Institutional and Network Social support during COVID-19: A case study of one university's students and their support-seeking behaviors | Review of Communication | Not related wellbeing |
| 1380 | Aydin, T.;Arabaci, G.;Kilintari, M.;Taylor, J.;Parris, B. A.; | 2024 | Inhibitory control ability moderates the relationship between internet addiction and inattention in ADHD in a community sample. | Personality and Individual Differences | Mental health disorders |
| 1381 | Bai, S.;Ying, Z. M.;Ying, J. K.;Zhang, Q. Y.;Lv, Y. H.;Wu, Z. M.; | 2024 | Inhibition of 5-HT alleviates PTSD-like behaviors and promotes hippocampal neuroplasticity by modulating hippocampal autophagy in rats. | Journal of Neurophysiology | Mental health disorders |
| 1382 | McKinley, C. J.;Wright, P. J.; | 2014 | Informational social support and online health information seeking: Examining the association between factors contributing to healthy eating behavior | Computers in Human Behavior | Not related wellbeing |

| | | | | | |
|---|---|---|---|---|---|
| 1383 | Voss, R. P.;Corser, R.;McCormick, M.;Jasper, J. D.; | 2018 | Influencing health decision-making: a study of color and message framing | Psychology & Health | Not related wellbeing |
| 1384 | Yin, Xiangju;Huang, Yiming;Zhang, Xin;Chen, Yuqian;Wang, Mingyue;Qian, Hongwei; | 2022 | Influencing Factors and Improvement Path of Academic Engagement among College Students in the Context of Epidemic Prevention and Control | International journal of environmental research and public health | Not related wellbeing |
| 1385 | Leavens, E. L. S.;Miller, M. B.;Brett, E. I.;Baraldi, A.;Leffingwell, T. R.; | 2020 | Influencing college students' normative perceptions of protective behavioral strategies: a pilot randomized trial | Addictive behaviors | Not related wellbeing |
| 1386 | Yamaguchi, A.; | 2013 | Influences of social capital on health and well-being from a qualitative approach | Glob J Health Sci | Not related wellbeing |
| 1387 | Metts, A. V.;Craske, M. G.; | 2023 | Influence of social support on cognitive reappraisal in young adults elevated neuroticism | Behavior research and therapy | The population is not college students |
| 1388 | Zhou, J.;Yang, J.;Yu, Y.;Wang, L.;Han, D.;Zhu, X.;He, J.;Qiu, X.;Yang, X.;Qiao, Z.;Sui, H.;Yang, Y.; | 2017 | Influence of school-level and family-level variables on Chinese college students' aggression | Psychol Health Med | Not related wellbeing |
| 1389 | Liang, B.;Li, B.;Fan, X.;Mu, Y.;Wang, J.; | 2024 | Influence of perceived social support on detection of social norm violation: evidence from N1 and N400 | Front Psychol | Not related wellbeing |
| 1390 | Almalki, S. A.; | 2019 | Influence of Motivation on Academic Performance among Dental College Students | Open Access Maced J Med Sci | Not related wellbeing |
| 1391 | Sun, J.;Cheng, W.;Fan, Z.;Zhang, X.; | 2020 | Influence of high-intensity intermittent training on glycolipid metabolism in obese male college students | Annals of Palliative Medicine | Meta review |

| | | | | | |
|---|---|---|---|---|---|
| 1392 | Li, X. Y.;Yang, P. C.;Jiang, Y. J.;Gao, D. D.; | 2023 | Influence of fear of COVID-19 on depression: The mediating effects of anxiety and the moderating effects of perceived social support and stress perception | Frontiers in Psychology | Mental health disorders |
| 1393 | Kubota, T.;Mori, H.;Morisawa, T.;Hanyu, K.;Kuge, H.;Watanabe, M.;Tanaka, T. H.; | 2020 | Influence of electroacupuncture stimulation on skin temperature, skin blood flow, muscle blood volume, and pupil diameter | Acupuncture in medicine | Not related wellbeing |
| 1394 | Huynh, K. V.;Glass, I. V.;Zanarini, M. C.; | 2024 | INCREASING PREVALENCE OF ATTENTION-DEFICIT/HYPERACTIVITY DISORDER IN PATIENTS WITH BORDERLINE PERSONALITY DISORDER | Journal of Personality Disorders | Mental health disorders |
| 1395 | Naseem, U.;Thapa, S.;Zhang, Q.;Hu, L.;Rashid, J.;Nasim, M.; | 2024 | Incorporating historical information by disentangling hidden representations of mental health surveillance on social media | Social Network Analysis and Mining | Not related wellbeing |
| 1396 | Joubert, L.;Kilgas, M.;Riley, A.;Gautam, Y.;Donath, L.;Drum, S.; | 2017 | In-Class Cycling to Augment College Student Academic Performance and Reduce Physical Inactivity: results from an RCT | International journal of environmental research and public health | Not related wellbeing |
| 1397 | Mastroleo, N. R.;Murphy, J. G.;Colby, S. M.;Monti, P. M.;Barnett, N. P.; | 2011 | Incident-specific and individual-level moderators of brief intervention effects with mandated college students | Psychology of addictive behaviors | Mental health disorders |
| 1398 | González-Barriga, F.;Orduña, V.; | 2024 | Incentive-salience attribution is attenuated in spontaneously hypertensive rats, an animal model of ADHD. | Behavioural processes | Mental health disorders |
| 1399 | Zhang, Y.;Liu, Z.;Zhao, Y. H.; | 2021 | Impulsivity, Social Support, and Depression Are | Frontiers in | Mental health disorders |

| | | | | | |
|---|---|---|---|---|---|
| | | | Associated With Latent Profiles of Internet Addiction Among Male College Freshmen | Psychiatry | |
| 1400 | Lamont, K.;van Woerden, H. C.;King, E.;Wendelboe-Nelson, C.;Humphry, R. W.;Stark, C.;Williams, C.;Maxwell, M.; | 2024 | Improving the mental health of farmers: what types of remote support are acceptable, feasible, and improve outcomes? A feasibility RCT | Discover Mental Health | Not related wellbeing |
| 1401 | Poon, K.;Ho, M. S. H.;Wang, L. C.;Lee, H. M.;Lau, W. K. W.;Chan, W. W. L.; | 2024 | Improving cognitive function in Chinese children with ADHD and/or RD through computerized working memory training | BMC Psychology | Mental health disorders |
| 1402 | Cheng, D.;Yang, Y.;Yan, X.;Chen, Q.; | 2024 | Improved effect of EEG-biofeedback intervention on cognitive function in childhood idiopathic epilepsy with ADHD: A retrospective study | Heliyon | Mental health disorders |
| 1403 | Ahmadi Moghadam, E.;Abedinzadeh Torghabeh, F.;Hosseini, S. A.;Moattar, M. H.; | 2024 | Improved ADHD Diagnosis Using EEG Connectivity and Deep Learning through Combining Pearson Correlation Coefficient and Phase-locking Value | Neuroinformatics | Mental health disorders |
| 1404 | Doughty, K. N.;Martin-Parchment, M.; | 2023 | Imposter phenomenon and experiences of discrimination among students at a predominantly White institution | J Am Coll Health | Not related wellbeing |
| 1405 | Mana, A.;Saka, N.;Dahan, O.;Ben-Simon, A.;Margalit, M.; | 2022 | Implicit Theories, Social Support, and Hope as Serial Mediators for Predicting Academic Self-Efficacy Among Higher Education Students | Learning Disability Quarterly | Unpublished journal article |
| 1406 | Painter, J. T.;Pyne, J.;Curran, G.;Raciborski, R. A.;Russell, S.;Fortney, J.;Gifford, A. L.;Ohl, M.;Woodward, E. N.; | 2024 | Implementation of collaborative care for depression in VA HIV clinics: Translating Initiatives for Depression into Effective Solutions (TIDES): protocol for a cluster-randomized type 3 | Implementation Science Communications | Mental health disorders |

| | | | | | |
|---|---|---|---|---|---|
| | | | hybrid effectiveness-implementation trial | | |
| 1407 | Calancie, O. G.;Parr, A. C.;Brien, D. C.;Coe, B. C.;Booij, L.;Khalid-Khan, S.;Munoz, D. P.; | 2024 | Impairment of Visual Fixation and Preparatory Saccade Control in Borderline Personality Disorder With and Without Comorbid Attention-Deficit/Hyperactivity Disorder | Biological Psychiatry: Cognitive Neuroscience and Neuroimaging | Mental health disorders |
| 1408 | Huang, Y.;Liu, Y.;Hu, Q.;Zhang, Q.; | 2024 | Impaired Reactive Control But Preserved Proactive Control in Hyperactive Children | Journal of Attention Disorders | The population is not college students |
| 1409 | Aksu, M. H.;Baltaci, N. N.;Yigman, F.;Yilmaz, O.; | 2023 | 'Impaired Autonomy and Performance' Predicts Instagram Addiction Among Instagram Users: A Cross-Sectional Study | Gazi Medical Journal | Mental health disorders |
| 1410 | Liang, P.;Li, Z.;Li, J.;Wei, J.;Li, J.;Zhang, S.;Xu, S.;Liu, Z.;Wang, J.; | 2023 | Impacts of complex electromagnetic radiation and low-frequency noise exposure conditions on the cognitive function of operators | Frontiers in Public Health | Not related wellbeing |
| 1411 | Houser, C.;Cavallo-Medved, D.;Bondy, M.; | 2023 | Impact of the Preparation for Academic Success in Science (PASS) High School to University Transition Program | Canadian Journal for the Scholarship of Teaching and Learning | The population is not college students |
| 1412 | Alshehri, M. A.;Kruse-Diehr, A. J.;McDaniel, J.;Partridge, J. A.;Null, D.; | 2021 | Impact of Social Support on the Physical Activity Behaviors of International College Students in the United States | Int J Exerc Sci | Not related wellbeing |
| 1413 | Alshehri, Mohammed;Kruse-Diehr, Aaron J.;McDaniel, Justin T.;Partridge, Julie;Null, Dawn B.; | 2023 | Impact of social support on the dietary behaviors of international college students in the United States | Journal of American College Health | Not related wellbeing |
| 1414 | Mao, X.;Hou, T.;Zhang, Y.;Zhang, J.;Zhang, F.;Liu, W.; | 2024 | Impact of social support on PTSD: Chain mediating effects of insomnia and anxiety | Global Mental Health | Mental health disorders |

| | | | | | |
|---|---|---|---|---|---|
| 1415 | He, T. B.;Tu, C. C.;Bai, X.; | 2022 | Impact of Social Support on College Students' Anxiety Due to COVID-19 Isolation: Mediating Roles of perceived risk and resilience in the post-pandemic period | Front Psychol | Mental health disorders |
| 1416 | Lahiri, S.;Choudhury, S.;Chatterjee, S.;Hazra, A.; | 2019 | Impact of social media on academic performance and interpersonal relation: A cross-sectional Study among students at a tertiary medical center in East India | Journal of Education and Health Promotion | Not related wellbeing |
| 1417 | Ning, W.;Inan, F. A.; | 2023 | Impact of social media addiction on college student's academic performance: an interdisciplinary perspective | Journal of Research on Technology in Education | Mental health disorders |
| 1418 | Yang, W.;Hu, W.;Morita, N.;Ogai, Y.;Saito, T.;Wei, Y.; | 2022 | Impact of Short-Term Intensive-Type Cognitive Behavioral Therapy Intervention on Internet Addiction among Chinese College Students: a Randomized Controlled Trial | International journal of environmental research and public health | Mental health disorders |
| 1419 | Chen, C.;Li, H.;Niu, Y.;Liu, C.;Lin, Z.;Cai, J.;Li, W.;Ge, W.;Chen, R.;Kan, H.; | 2019 | Impact of short-term exposure to fine particulate matter air pollution on urinary metabolome: a randomized, double-blind, crossover trial | Environment International | Meta review |
| 1420 | Wang, J.;Lin, L.;Huang, J.;Zhang, J.;Duan, J.;Guo, X.;Wu, S.;Sun, Z.; | 2022 | Impact of PM2.5 exposure on plasma metabolome in healthy adults during air pollution waves: a randomized, crossover trial | Journal of Hazardous Materials | Meta review |
| 1421 | Karawekpanyawong, Nuntaporn;Wongpakaran, Tinakon;Wongpakaran, Nahathai;Boonnag, | 2021 | Impact of Perceived Social Support on the Relationship between ADHD and Depressive Symptoms among First-Year Medical Students: A Structural Equation Model Approach | Children | Mental health disorders |

| | | | | | |
|---|---|---|---|---|---|
| | Chiraphat;Siritikul, Sirinut;Chalanunt, Sirikorn;Kuntawong, Pimolpun; | | | | |
| 1422 | Hussein, R. S.;Yousef, S. S.; | 2024 | Impact of perceived social support on suicidal ideation among students at Ain Shams University | Middle East Current Psychiatry | Mental health disorders |
| 1423 | Dou, F.;Li, Q.;Li, X.;Li, Q.;Wang, M.; | 2023 | Impact of Perceived Social Support on fear of missing out (FoMO): A moderated mediation model | Current Psychology | Not related wellbeing |
| 1424 | Dou, F.;Li, Q. L.;Li, X. A.;Li, Q. F.;Wang, M. H.; | 2023 | Impact of perceived social support on Fear of missing out (FoMO): A moderated mediation model | Current Psychology | Not related wellbeing |
| 1425 | Sheng, W.;Fang, S. H.; | 2024 | Impact of Moral Elevation on College Students Sense of Meaning of Life: The Mediating Roles of Gratitude and Perceived Social Support | Psychology Research and Behavior Management | Not related wellbeing |
| 1426 | Shauran, B.;Jain, R.;Jain, N.; | 2021 | Impact of Mentoring on Academic Success of students in similar and cross-gender mentoring relationships | International Journal of Indian Culture and Business Management | Not related wellbeing |
| 1427 | Antúnez, Z.;Vergara, R. C.;Langer, A. I.;Santander, J.;Baader, T.;Alamo, C.;Arce, M.;Delgado, B.;Laurin, K.;Moncada, C.; | 2023 | Impact of mental health problems and social support on sleep quality: Follow-up before and during the first eight months of the COVID-19 pandemic in Chilean university students | Anales De Psicologia | Mental health disorders |
| 1428 | Kemmler, W.;von Stengel, S.;Kohl, M.;Bauer, J.; | 2016 | Impact of exercise changes on body composition during the college years--a five-year randomized | BMC Public Health | Not related wellbeing |

| | | | | | |
|---|---|---|---|---|---|
| | | | controlled study | | |
| 1429 | Zhao, Y.;Wang, R.;Li, H.;Chen, C.;Zhou, B.;Weng, X.;Hua, Y.;Jia, Y.;Wu, Y.;Li, Y.; | 2023 | Impact of Easing COVID-19 Restrictions on Fear of COVID-19 and Social Support Among Chinese Students: A Longitudinal Analysis | Psychol Res Behav Manag | Not related wellbeing |
| 1430 | Zareipour, M. A.;Mahmoodi, H.;Valizadeh, R.;Ghelichi Ghojogh, M.;Rezaie Moradali, M.;Zare, F.; | 2018 | Impact of an Educational Intervention Based on the BASNEF Model on Skin Cancer Preventive Behavior of College Students | Asian Pacific journal of cancer prevention | Mental health disorders |
| 1431 | Milan, J. E.;White, A. A.; | 2010 | Impact of a stage-tailored, web-based intervention on folic acid-containing multivitamin use by college women | American journal of health promotion | Published not from January 2010 to 31 March 2024 |
| 1432 | Grim, M.;Hortz, B.;Petosa, R.; | 2011 | Impact evaluation of a pilot web-based intervention to increase physical activity | Am J Health Promot | Mental health disorders |
| 1433 | Ward, J.;Coats, J.;Tyer, K.;Weigand, S.;Williams, G.; | 2013 | Immediate effects of anterior upper thoracic spine manipulation on cardiovascular response | Journal of manipulative and physiological therapeutics | Not related wellbeing |
| 1434 | Bavarian, N.;Flay, B. R.;Ketcham, P. L.;Smit, E.; | 2013 | Illicit use of prescription stimulants in a college student sample: a theory-guided analysis | Drug and alcohol dependence | Not related wellbeing |
| 1435 | Patterson, M. S.;Francis, A. N.;Gagnon, L. R.;Prochnow, T.; | 2023 | I'll be there for you: The effects of exercise engagement on social support provision within undergraduate students' networks. | J Am Coll Health | Not related wellbeing |
| 1436 | Cullum, Jerry;O'Grady, Megan;Sandoval, Patricia;Armeli, Stephen;Tennen, Howard; | 2013 | Ignoring norms with a little help from my friends: social support reduces normative influence on drinking behavior. | Journal of Social and Clinical Psychology | Not related wellbeing |
| 1437 | Luyckx, K.;Klimstra, T. A.;Duriez, | 2012 | Identity processes and coping strategies in college | J Youth Adolesc | Not related wellbeing |

| | | | | | |
|---|---|---|---|---|---|
| | B.;Schwartz, S. J.;Vanhalst, J.; | | students: short-term longitudinal dynamics and the role of personality | | |
| 1438 | García-Valdez, A. A.;Román-Godínez, I.;Salido-Ruiz, R. A.;Torres-Ramos, S.; | 2024 | Identifying PTSD sex-based patterns through explainable artificial intelligence in biometric data | Network Modeling Analysis in Health Informatics and Bioinformatics | Mental health disorders |
| 1439 | Kornbluh, M.;Withers, M. C.;Ades, J.;Grennan, G.;Mishra, J.; | 2022 | Identifying Protective socio-ecological Factors for College Students in California's deadliest wildfire | J Am Coll Health | Not related wellbeing |
| 1440 | Gomes, K. D.;Moore, B. A.;Straud, C. L.;Baker, M. T.;Isler, W. C.;McNally, R. J.;Litz, B. T.;Peterson, A. L.; | 2024 | Identifying Predictors of Positive and Negative Affect at Mid-Deployment Among Military Medical Personnel | Military Medicine | Not related wellbeing |
| 1441 | Sano, A.;Taylor, S.;McHill, A. W.;Phillips, A. J.;Barger, L. K.;Klerman, E.;Picard, R.; | 2018 | Identifying Objective Physiological Markers and Modifiable Behaviors for Self-Reported Stress and Mental Health Status Using Wearable Sensors and Mobile Phones: Observational Study | J Med Internet Res | Mental health disorders |
| 1442 | Alaslani, K.;Alandejani, M.; | 2020 | Identifying factors that Influence students performance through social networking sites: An exploratory case study | Heliyon | Not related wellbeing |
| 1443 | Tran, T.;Ickes, M. J.;Hester, J. W.;Kavuluru, R.; | 2021 | Identifying current Juul users among emerging adults through Twitter feeds | Int J Med Inform | The population is not college students |
| 1444 | Niitsu, K.;Lee, C.;Rice, M. J.; | 2023 | Identification of Relationships Among Resilience Factors Using Network Analysis: A Pilot Study | J Am Psychiatr Nurses Assoc | Not related wellbeing |
| 1445 | Qi, P.;Huang, M.;Ren, X.;Zhai, Y.;Qiu, C.;Zhu, H.; | 2024 | Identification of potential biomarkers and therapeutic targets related to post-traumatic stress disorder due to traumatic brain injury | European Journal of Medical Research | Mental health disorders |

| | | | | | |
|---|---|---|---|---|---|
| 1446 | Shan, Y.;Sun, Y.;Xie, J.;Li, T.;Chen, K.; | 2024 | Identification of central symptoms in problematic WeChat use and depression among Chinese college students: a network analysis | BMC Psychiatry | Mental health disorders |
| 1447 | Prause, N.;Binnie, J.; | 2024 | Iatrogenic effects of Reboot/NoFap on public health: A preregistered survey study | Sexualities | Not related wellbeing |
| 1448 | Steers, M. L.;Quist, M. C.;Bryan, J. L.;Foster, D. W.;Young, C. M.;Neighbors, C.; | 2016 | I Want You to Like Me: Extraversion, Need for Approval, and Time on Facebook as Predictors of Anxiety | Transl Issues Psychol Sci | Mental health disorders |
| 1449 | Tsuneyoshi, N.;Hosoya, T.;Takeno, Y.;Saitoh, K.;Murai, H.;Amimoto, N.;Tatsumi, R.;Watanabe, S.;Hasegawa, Y.;Kikkawa, E.;Goto, K.;Nishigaki, F.;Tamura, K.;Kimura, H.; | 2024 | Hypoimmunogenic human iPSCs expressing HLA-G, PD-L1, and PD-L2 evade innate and adaptive immunity. | Stem Cell Research and Therapy | Not related wellbeing |
| 1450 | Gloede, M. E.;Sapp, M.;Van Susteren, W.; | 2021 | Hypnosis and Mindfulness Meditation: The Power of Suggestibility | International journal of clinical and experimental hypnosis | Mental health disorders |
| 1451 | McLaren, V.;Gallagher, M.;Hopwood, C. J.;Sharp, C.; | 2022 | Hypermentalizing and Borderline Personality Disorder: a Meta-Analytic Review | American journal of psychotherapy | Meta review |
| 1452 | Sajdlowska, J.;Shenasan, P.;Clarke, N.;Bhattacharyya, N.; | 2024 | Hydrosalpinx leading to fallopian tube torsion in a 10-year-old female: A case report | Journal of Pediatric Surgery Case Reports | Not related wellbeing |
| 1453 | McEwan, B.; | 2011 | HYBRID ENGAGEMENT: HOW FACEBOOK HELPS AND HINDERS STUDENTS' SOCIAL INTEGRATION | Higher Education Administration with Social Media: | Not related wellbeing |

| | | | | | |
|---|---|---|---|---|---|
| | | | | Including Applications in Student Affairs, Enrollment Management, Alumni Relations, and Career Centers | |
| 1454 | Espeleta, H. C.;Witcraft, S. M.;Raffa, T.;Kartiko, S.;Dawson, D.;Becerra, G.;Roisman, H.;Hughes-Halbert, C.;Mueller, M.;Powell, E.;Brock, T.;Sarani, B.;Ruggiero, K. J.; | 2024 | Hybrid 1 randomized controlled trial of an integrated stepped-care mental health intervention for traumatic injury patients | Contemporary Clinical Trials | Mental health disorders |
| 1455 | Hecht, M. L.;BeLue, R.;Ray, A.;Hopfer, S.;Miller-Day, M.;McKee, F.; | 2022 | HPV Vaccine Intent among Adult Women Receiving Care at Community Health Centers | Journal of Cancer Education | Not related wellbeing |
| 1456 | Whelan, E.;Golden, W.;Tarafdar, M.; | 2022 | How technostress and self-control of social networking sites affect academic achievement and wellbeing | Internet Research | Mental health disorders |
| 1457 | Shu, Y.;Lin, W.;Yang, J.;Huang, P.;Li, B.;Zhang, X.; | 2022 | How social support predicts anxiety among university students during COVID-19 control phase: Mediating roles of self-esteem and resilience | Anal Soc Issues Public Policy | Mental health disorders |
| 1458 | Zhang, Y.;Farina, R. E.;Lawrence, S. E.;Walters, T. L.;Clark, A. N.;Hanna-Walker, V.;Lefkowitz, E. | 2022 | How Social Support and Parent-Child Relationship Quality Relate to LGBTQ Plus College Students' Well-Being During COVID-19 | Journal of Family Psychology | The population is not college students |

| | | | | | |
|---|---|---|---|---|---|
| | S.; | | | | |
| 1459 | Shen, Y. N.;Sun, X. J.;Xin, T.; | 2019 | How social support affects moral disengagement: The role of anger and hostility | Social Behavior and Personality | Not related wellbeing |
| 1460 | Lake, J. S.;Alston, A. T.;Kahn, K. B.; | 2021 | How Social Networking Use and Beliefs About Inequality Affect Engagement With Racial Justice Movements | Race and Justice | Not related wellbeing |
| 1461 | Chang'an, Zhang;Tang, Lingjie;Liu, Zhifang; | 2023 | How social media usage affects psychological and subjective well-being: testing a moderated mediation model | BMC Psychology | Not related wellbeing |
| 1462 | Roper, L. D.; | 2023 | How self-advocacy strategies help students with 'invisible disabilities' flourish beyond high school | Journal of Human Behavior in the Social Environment | The population is not college students |
| 1463 | Almeida, D. J.;Byrne, A. M.;Smith, R. M.;Ruiz, S.; | 2021 | How Relevant Is Grit? The Importance of Social Capital in First-Generation College Students' Academic Success | Journal of College Student Retention: Research, Theory and Practice | Not related wellbeing |
| 1464 | Kenny, Rachel;Dooley, Barbara;Fitzgerald, Amanda; | 2016 | How psychological resources mediate and perceived social support moderates the relationship between depressive symptoms and help-seeking intentions in college students. | British Journal of Guidance & Counselling | Mental health disorders |
| 1465 | Robbins, R.;Niederdeppe, J.; | 2017 | How Online Peer-to-Peer Conversation Shapes the Effects of a Message About Healthy Sleep | Prevention science | Mental health disorders |
| 1466 | Hu, H. H.;Yang, X.;Mo, P. K. H.;Zhao, C. J.;Kuang, B. B.;Zhang, G. H.;Lin, G. Y.; | 2022 | How mobile phone addiction is associated with suicidal ideation in university students in China: Roles of depression and online social support | Frontiers in Psychology | Mental health disorders |

| | | | | | |
|---|---|---|---|---|---|
| 1467 | Mutuyimana, C.;Maercker, A.; | 2024 | How meaning in life and vitality are associated with posttrauma outcomes: A systematic review. | Journal of Traumatic Stress | Not related wellbeing |
| 1468 | Kong, F.;Gong, X. Y.;Sajjad, S.;Yang, K. R.;Zhao, J. J.; | 2019 | How Is Emotional Intelligence Linked to Life Satisfaction? The Mediating Role of Social Support, Positive Affect, and Negative Affect | Journal of Happiness Studies | Not related wellbeing |
| 1469 | Chen, B.;Sun, J.;Feng, Y.; | 2020 | How Have COVID-19 Isolation Policies Affected Young People's Mental Health? - Evidence From Chinese College Students | Front Psychol | Not related wellbeing |
| 1470 | Park, K. G.;Kim, J.;Kim, H.; | 2022 | How exhibitionism and voyeurism contribute to engagement in SNS use: The mediating effects of content production and consumption | Telematics and Informatics | Not related wellbeing |
| 1471 | Huang, J.;Qiao, T.;Song, Z.;Yan, J.; | 2022 | How Does Social Support Influence Junior College Students' Occupational Identity in Pre-school Education? | Front Psychol | Not related wellbeing |
| 1472 | Feng, Q.;Chen, X.;Guo, Z.; | 2023 | How does role accumulation enhance career adaptability? A dual mediation analysis | Curr Psychol | Not related wellbeing |
| 1473 | Ubara, A.;Tanizawa, N.;Harata, M.;Suh, S.;Yang, C. M.;Li, X.;Okajima, I.; | 2022 | How Does E-mail-Delivered Cognitive Behavioral Therapy Work for Young Adults (18-28 Years) with Insomnia? Mediators of Changes in Insomnia, Depression, Anxiety, and Stress | International journal of environmental research and public health | The population is not college students |
| 1474 | Liu, S. S.;Shteynberg, G.;Morris, M. W.;Yang, Q.;Galinsky, A. D.; | 2021 | How Does Collectivism Affect Social Interactions? A Test of Two Competing Accounts | Pers Soc Psychol Bull | Not related wellbeing |
| 1475 | Butler, S. J.;Ramsey-Wade, C.; | 2024 | How do clients experience intensive EMDR for post-traumatic stress? An interpretative phenomenological analysis | European Journal of Trauma and Dissociation | Mental health disorders |

| | | | | | |
|---|---|---|---|---|---|
| 1476 | Ajjan, H.;Cao, Y.;Hartshorne, R.; | 2019 | How compulsive social media use influences college students' performance: A structural equation analysis with gender comparison | International Journal of Learning Technology | Not related wellbeing |
| 1477 | Wang, H.;Yang, J.;Li, P.; | 2022 | How and When goal-oriented self-regulation improves college students' well-being: A weekly diary study | Curr Psychol | Not related wellbeing |
| 1478 | Maier, Karl J.;James, Ashley E.; | 2014 | Hostility and Social Support Explain Physical Activity Beyond Negative Affect among Young Men, but not Women, in College. | Behavioral Medicine | Not related wellbeing |
| 1479 | Wedgeworth, M. L.;Eyer, J. C.;March, A. L.;Feldman, D. B.; | 2021 | Hoping to Pass: Randomized Trial of a One-Time Hope Intervention on Standardized Exam Passing Rates in BSN Students | Journal of the American Psychiatric Nurses Association | Mental health disorders |
| 1480 | Hollis, B.;Sheehan, B. E.;Kelley, M. L.;Stevens, L.; | 2022 | Hookups Among U.S. College Students: Examining the Association Between Hookup Motives and Personal Affect | Arch Sex Behav | Not related wellbeing |
| 1481 | Castañeda, G.;Barnett, T. E.;Soule, E. K.;Young, M. E.; | 2016 | Hookah smoking behavior initiation in the context of Millennials | Public Health | Not related wellbeing |
| 1482 | Cai, M.;Park, H. R.;Yang, E. J.; | 2024 | Hominis Placenta modulates PTSD-like behaviors in SPSS-induced PTSD mice: Regulating energy metabolism and neuronal activity. | Biomedicine and Pharmacotherapy | Mental health disorders |
| 1483 | Hong, W.;Wang, Q.;Hou, Q.;Zhao, N.;Wang, R.;Bai, Y.;Hu, C.;Liu, W.; | 2023 | Home quarantine during COVID-19 blunted childhood trauma-related psychiatric symptoms in Chinese college students. | Front Public Health | The population is not college students |
| 1484 | Palmer, L.;Busuttil, W.;Simms, A.;Fear, N. T.;Stevelink, S. A. M.; | 2024 | Holding and rupture: Describing posttraumatic stress among former UK Army and Royal Marine | PLoS ONE | Mental health disorders |

| | | | | | |
|---|---|---|---|---|---|
| | | | personnel deployed to Iraq and Afghanistan | | |
| 1485 | Castro-Figueroa, E. M.;Peña-Vargas, C.;Rodríguez-Santiago, M.;Figueroa, J. I.;Hernández, R.;Rodríguez, Z.;Jim, H.;Pereira, C.;Torres-Blasco, N.;Flores, I.;Costas-Muñiz, R.;Armaiz-Pena, G. N.; | 2024 | Hispanic Cancer Survivors Exposed to Multiple Natural Disasters: Pre–Post-Disaster Changes in Anxiety, Depression, PTSD, Perceived Stress, and Physical Symptom Burden | International journal of environmental research and public health | Mental health disorders |
| 1486 | Salomon, A.;Kolikant, Y. B.; | 2016 | High-school students' perceptions of the effects of non-academic usage of ICT on their academic achievements | Computers in Human Behavior | The population is not college students |
| 1487 | Sobaih, A. E.;Hasanein, A.;Elshaer, I. A.; | 2022 | Higher Education in and after COVID-19: The Impact of Using Social Network Applications for E-Learning on Students' Academic Performance | Sustainability | Not related wellbeing |
| 1488 | Zhang, L.;Xuan, R.;Chen, Q.;Zhao, Q.;Shi, Z.;Du, J.;Zhu, C.;Yu, F.;Ji, G.;Wang, K.; | 2022 | High-definition transcranial direct current stimulation modulates eye gaze on emotional faces in college students with alexithymia: an eye-tracking study. | Progress in neuro-psychopharmacology & biological psychiatry | Not related wellbeing |
| 1489 | Selai, C.;Lee, C. H.;Simeoni, S.;Pakzad, M.;Joyce, E.;Petrochilos, P.;Rantell, K. R.;Boico, V.;Panicker, J. N.; | 2024 | High Prevalence of Psychological Comorbidities and Functional Neurological Symptoms in Women With Urinary Retention | Journal of Urology | Not related wellbeing |
| 1490 | McMillan, W.;Stice, E.;Rohde, P.; | 2011 | High- and low-level dissonance-based eating disorder prevention programs with young women with body image concerns: an experimental trial | Journal of consulting and clinical psychology | Mental health disorders |

| | | | | | |
|---|---|---|---|---|---|
| 1491 | Yisa, V.;Orji, R.; | 2024 | Hidden desires echoed distress: Dissecting Nigeria's sexting landscape and its ties to depression. | Computers in Human Behavior | Mental health disorders |
| 1492 | Kulick, A.;Wernick, L. J.;Woodford, M. R.;Renn, K.; | 2017 | Heterosexism, Depression, and Campus Engagement Among LGBTQ College Students: Intersectional Differences and Opportunities for Healing | Journal of Homosexuality | Mental health disorders |
| 1493 | Cloutier, R. M.;Kearns, N. T.;Knapp, A. A.;Contractor, A. A.;Blumenthal, H.; | 2019 | Heterogeneous Patterns of Marijuana Use Motives Using Latent Profile Analysis | Subst Use Misuse | Not related wellbeing |
| 1494 | Kim, A. S.;Choi, S.;Park, S.; | 2020 | Heterogeneity in first-generation college students influencing academic success and adjustment to higher education | Social Science Journal | Not related wellbeing |
| 1495 | Yaklin, S.;Jain, R.;Cole, S. P.;Raison, C.;Rolin, D.;Jain, S.; | 2020 | HERO Wellness Scale: examining a new mental wellness scale | Annals of Clinical Psychiatry | Not related wellbeing |
| 1496 | Park, H. R.;Cai, M.;Yang, E. J.; | 2024 | Herbal Formula Extract Ameliorates Anxiety and Cognitive Impairment via Regulation of the Reelin/Dab-1 Pathway in a Murine Model of Post-Traumatic Stress Disorder | Pharmaceutics | Mental health disorders |
| 1497 | Shi, W.;Hall, B. J.; | 2020 | Help-seeking preferences among Chinese college students exposed to a natural disaster: a person-centered approach | Eur J Psychotraumatol | Not related wellbeing |
| 1498 | Dong, H.;Dai, J.;Lipson, S. K.;Curry, L.; | 2022 | Help-seeking for mental health services in Asian American college students: an exploratory qualitative study | J Am Coll Health | Not related wellbeing |
| 1499 | Tureluren, E.;Claes, L.;Andriessen, | 2022 | Help-seeking behavior in bereaved university and | Front Psychol | Mental health disorders |

| | | | | | |
|---|---|---|---|---|---|
| | K.; | | college students: Associations with grief, mental health distress, and personal growth | | |
| 1500 | Ray, E. C.;Arpan, L.;Oehme, K.;Perko, A.;Clark, J.; | 2021 | Helping students cope with adversity: the influence of a web-based intervention on students' self-efficacy and intentions to use wellness-related resources | Journal of American College Health | Mental health disorders |
| 1501 | Ginsburg, H. J.;Cameron, R.;Mendez, R. V.;Westhoff, M.; | 2016 | Helping others use social media: Age stereotypes when estimating learner's success | Psychology, Society, and Education | Not related wellbeing |
| 1502 | Downs, M. F.;Eisenberg, D.; | 2012 | Help-seeking and treatment use among suicidal college students | J Am Coll Health | Mental health disorders |
| 1503 | Lin, G.;Xiang, Q.;Fu, X.;Wang, S.;Wang, S.;Chen, S.;Shao, L.;Zhao, Y.;Wang, T.; | 2012 | Heart rate variability biofeedback decreases blood pressure in prehypertensive subjects by improving autonomic function and baroreflex. | Journal of alternative and complementary medicine (New York, N.Y.) | Not related wellbeing |
| 1504 | Schwartz, E. K. C.;Palmisano, A. N.;Petrakis, I. L.;Pietrzak, R. H.;Sofuoglu, M.; | 2024 | Health correlates of experiential and behavioral avoidance among trauma-exposed veterans | Journal of Psychiatric Research | Not related wellbeing |
| 1505 | Yubero, S.;Navarro, R.;Larrañaga, E.;Esteban, M.;Gutiérrez, J.;Elche, M.; | 2018 | Health Contributing Factors in Higher Education Students: The Importance of Family and Friends | Healthcare (Basel) | Not related wellbeing |
| 1506 | Wright, R. R.;Nelson, R.;Garcia, S.;Butler, A.; | 2020 | Health Behavior Change in the Classroom: A Means to a Healthy End? | J Prim Prev | Not related wellbeing |
| 1507 | Wallin, K.;Wallin-Lundell, I.;Alehagen, S.;Hanberger, | 2024 | Having Reliable Support: A Prerequisite to Promote Sexual and Reproductive Health in | Archives of sexual behavior | Mental health disorders |

| | | | | | |
|---|---|---|---|---|---|
| | L.;Hultsjö, S.; | | Young Women with ADHD | | |
| 1508 | Fitzsimmons-Craft, E. E.;Taylor, C. B.;Newman, M. G.;Zainal, N. H.;Rojas-Ashe, E. E.;Lipson, S. K.;Firebaugh, M. L.;Ceglarek, P.;Topooco, N.;Jacobson, N. C.;et al.,; | 2021 | Harnessing mobile technology to reduce mental health disorders in college populations: a randomized controlled trial study protocol. | Contemporary Clinical Trials | Mental health disorders |
| 1509 | Yu, S. C.; | 2015 | Happiness or addiction: An example of Taiwanese college students' use of Facebook | International Journal of Technology and Human Interaction | Mental health disorders |
| 1510 | Kumar, D.;Yadav, P.;Joshy, V. M.;Thomas, B.; | 2023 | Happiness index of medical students and related factors in Andaman and Nicobar Islands, India | J Educ Health Promot | Not related wellbeing |
| 1511 | Salem, Alteneiji;Nizar Mohammad, Alsharari;Rasha Mohamed, AbouSamra;Houjeir, Roudaina; | 2023 | Happiness and Positivity in the Higher Education Context: An Empirical Study | The International Journal of Educational Management | Not related wellbeing |
| 1512 | Karyotaki, E.;Klein, A. M.;Ciharova, M.;Bolinski, F.;Krijnen, L.;de Koning, L.;de Wit, L.;van der Heijde, C. M.;Ebert, D. D.;Riper, H.;et al.,; | 2022 | Guided internet-based transdiagnostic individually tailored Cognitive Behavioral Therapy for symptoms of depression and/or anxiety in college students: a randomized controlled trial. | Behavior research and therapy | Mental health disorders |
| 1513 | Wayment, H. A.;Silver, R. C.; | 2021 | Grief and Solidarity Reactions 1 Week After an On-Campus Shooting | J Interpers Violence | Not related wellbeing |
| 1514 | Joseph, H. M.;Santosa, H.;Fisher, N.;Huppert, T.;Morgan, J. K.; | 2024 | Greater Frontoparietal Connectivity During Task Engagement Among Toddlers With | Developmental Psychobiology | Mental health disorders |

| | | | | | |
|---|---|---|---|---|---|
| | | | Parent-Reported Inattention | | |
| 1515 | Gabana, N. T.;Steinfeldt, J. A.;Wong, Y. J.;Chung, Y. B.; | 2017 | Gratitude, burnout, and sport satisfaction among college student-athletes: The mediating role of perceived social support | Journal of Clinical Sport Psychology | Not related wellbeing |
| 1516 | Fujitani, T.;Ohara, K.;Kouda, K.;Mase, T.;Miyawaki, C.;Momoi, K.;Okita, Y.;Nakamura, H.; | 2017 | Gratitude Predicts Well-being Mediated by Social Support and Sense of Coherence in Women | Health Behavior and Policy Review | Not related wellbeing |
| 1517 | Kaniuka, A. R.;Kelliher Rabon, J.;Brooks, B. D.;Sirois, F.;Kleiman, E.;Hirsch, J. K.; | 2021 | Gratitude and suicide risk among college students: Substantiating the protective benefits of being thankful | J Am Coll Health | Mental health disorders |
| 1518 | Zhang, Q. Y.;Tsai, W.; | 2023 | Gratitude and psychological distress among first-year college students: The mediating roles of perceived social support and support provision | Journal of Counseling Psychology | Mental health disorders |
| 1519 | Bassilios, B.;Currier, D.;Krysinska, K.;Dunt, D.;Machlin, A.;Newton, D.;Williamson, M.;Pirkis, J.; | 2024 | Government-funded suicide prevention in Australia – an environmental scan | BMC Public Health | Mental health disorders |
| 1520 | Koestner, R.;Powers, T. A.;Milyavskaya, M.;Carbonneau, N.;Hope, N.; | 2015 | Goal internalization and persistence as a function of autonomous and directive forms of goal support | J Pers | Not related wellbeing |
| 1521 | Pranckeviciene, E.;Kasperiuniene, J.; | 2024 | Global Suicide Mortality Rates (2000–2019): Clustering, Themes, and Causes Analyzed through Machine Learning and Bibliographic Data | International journal of environmental research and public health | Mental health disorders |
| 1522 | Mudiyanselage, S. P. K.;Tsai, Y. T.;Tsai, Y. J.;Yang, Y. H.;Lu, Z. | 2024 | Global overview of suicidal behavior and risk factors among the general population during the | BMC Psychology | Mental health disorders |

| | | | | | |
|---|---|---|---|---|---|
| | T.;Ko, N. Y.; | | COVID-19 pandemic: a scoping review | | |
| 1523 | Wang, P.;Huang, Y.; | 2020 | Give Me What I Want: Identifying the Support Needs of College Student Entrepreneurs | Front Psychol | Not related wellbeing |
| 1524 | Song, X.;Zhang, Y.;Tang, Z.;Dai, J.;Wu, Y.;Huang, G.;Niu, H.;Wang, Y.;Jin, X.;Du, L.; | 2024 | Ginger oil-loaded transdermal adhesive patch treats post-traumatic stress disorder. | Journal of Traditional Chinese Medical Sciences | Mental health disorders |
| 1525 | Keels, M.; | 2013 | Getting Them Enrolled Is Only Half the Battle: College Success as a Function of Race or Ethnicity, Gender, and Class | American Journal of Orthopsychiatry | Not related wellbeing |
| 1526 | Cramer, S. C.;Parodi, L.;Moslemi, Z.;Braun, R. G.;Aldridge, C. M.;Shahbaba, B.;Rosand, J.;Holman, E. A.;Griessenauer, C. J.;Patel, N.;Anderson, C.;Henry, J.;Kourkoulis, C.;Lin, D. J.;Zaba, N.;Gee, J.;Moon, J.;Schwertfeger, J.;Jayaraman, A.;Lee, R.;Lansberg | 2024 | Genetic Variation and Stroke Recovery: The STRONG Study | Stroke | Unpublished journal article |
| 1527 | Niitsu, K.;Houfek, J. F.;Rice, M. J.;Stoltenberg, S. F.;Kupzyk, K.;Barron, C.; | 2022 | Genetic associations with resilience to potentially traumatic events and vantage sensitivity to social support | Arch Psychiatr Nurs | Not related wellbeing |
| 1528 | Twenge, J. M.;Exline, J. J.;Grubbs, J. B.;Sastry, R.;Campbell, W. K.; | 2015 | Generational and period differences in American adolescents' religious orientation, 1966-2014 | PLoS ONE | Not related wellbeing |
| 1529 | Zeng, X.;Chen, Y.;Li, Y.; | 2023 | Generate Greater Gratitude When Being Help? A Study of the Psychological Mechanism of Gratitude for Chinese Poor College Students | Appl Res Qual Life | Not related wellbeing |

| | | | | | |
|---|---|---|---|---|---|
| 1530 | Bryan, J. L.;Quist, M. C.;Young, C. M.;Steers, M. L. N.;Lu, Q.; | 2016 | General Needs Satisfaction as a Mediator of the Relationship Between Ambivalence Over Emotional Expression and Perceived Social Support | Journal of Social Psychology | Not related wellbeing |
| 1531 | Goulet, Carol L.;Wells, Courtney K.;Szymanski, Lynda A.;Thieman, Thomas J.; | 2023 | Gender-specific social support and resilience in nontraditional female college students | Journal of American College Health | Not related wellbeing |
| 1532 | Xu, Y.;Yue, L. Z.;Wang, W.;Wu, X. J.;Liang, Z. Y.; | 2021 | Gender-Specific Impact of Self-Monitoring and Social Norm Information on Walking Behavior Among Chinese College Students Assessed Using WeChat: longitudinal Tracking Study | Journal of medical Internet research | Not related wellbeing |
| 1533 | Li, S.;Wang, L.;Xiong, J.;Xiao, D.; | 2022 | Gender-Specific Effects of 8-Week Multi-Modal Strength and Flexibility Training on Hamstring Flexibility and Strength | International journal of environmental research and public health | Not related wellbeing |
| 1534 | Jones, M. K.;Leath, S.;Settles, I. H.;Doty, D.;Conner, K.; | 2022 | Gendered Racism and Depression Among Black Women: Examining the Roles of Social Support and Identity | Cultural Diversity and Ethnic Minority Psychology | Mental health disorders |
| 1535 | Stark, A. M.;Tousignant, O.;Fireman, G. D.; | 2019 | Gender-Based Effects of Frames on Bullying Outcomes | Journal of Psychology | Not related wellbeing |
| 1536 | Razavi, M. R.; | 2021 | Gender differences in the effect of virtual social networks use on students' academic performance. | Current Psychology | Not related wellbeing |
| 1537 | Tifferet, S.; | 2020 | Gender Differences in Social Support on Social Network Sites: A Meta-Analysis | Cyberpsychology Behavior and Social Networking | Meta review |

| | | | | | |
|---|---|---|---|---|---|
| 1538 | Graves, B. S.;Hall, M. E.;Dias-Karch, C.;Haischer, M. H.;Apter, C.; | 2021 | Gender Differences in perceived stress and coping among college students | PLoS ONE | Mental health disorders |
| 1539 | Wilson, O. W. A.;Colinear, C.;Guthrie, D.;Bopp, M.; | 2022 | Gender differences in college student physical activity, campus recreational facility use, and comfort | J Am Coll Health | Not related wellbeing |
| 1540 | Sun, P.;Huang, Y.;Yu, H.;Wu, X.;Chen, J.;Fang, Y.;Zhang, X.; | 2024 | Gender differences in clinical characteristics and influencing factors of suicide attempts in first-episode and drug-naïve major depressive disorder patients with comorbid metabolic syndrome | BMC Psychiatry | Mental health disorders |
| 1541 | Tinajero, C.;Martínez-López, Z.;Rodríguez, M. S.;Guisande, M. A.;Páramo, M. F.; | 2015 | Gender and socioeconomic status differences in university students' perception of social support | European Journal of Psychology of Education | Not related wellbeing |
| 1542 | Lipson, S. K.;Speer, N.;Brunwasser, S.;Hahn, E.;Eisenberg, D.; | 2014 | Gatekeeper training and access to mental health care at universities and colleges | Journal of Adolescent Health | Not related wellbeing |
| 1543 | Xue, L. L.;Yan, Y.;Fan, H.;Zhang, L. P.;Wang, S. Y.;Chen, L. P.; | 2023 | Future self-continuity and depression among college students: The role of the presence of meaning and perceived social support | Journal of Adolescence | Mental health disorders |
| 1544 | Wright, Kevin B.;King, Shawn;Rosenberg, Jenny; | 2014 | Functions of social support and Self-verification in association with loneliness, depression, and stress | Journal of Health Communication | Mental health disorders |
| 1545 | Feng, A.;Zhi, D.;Feng, Y.;Jiang, R.;Fu, Z.;Xu, M.;Zhao, M.;Yu, S.;Stevens, M.;Sun, L.;Calhoun, V.;Sui, J.; | 2024 | Functional imaging derived ADHD biotypes based on deep clustering: a study on personalized medication therapy guidance | clinical medicine | Mental health disorders |
| 1546 | Hui, B. P. H.;Au, A. K. Y.;Ng, J. C. | 2022 | From Social Networking Site Use to Subjective | Int J Environ Res | Not related wellbeing |

| | | | | | |
|---|---|---|---|---|---|
| | K.;Song, X.; | | Well-Being: The Interpersonal and Intrapersonal Mediating Pathways of Prosocial Behavior among Vocational College Students in China | Public Health | |
| 1547 | Rousseau, C.;Hassan, G.;Miconi, D.;Lecompte, V.;Mekki-Berrada, A.;El Hage, H.;Oulhote, Y.; | 2019 | From social adversity to sympathy for violent radicalization: the role of depression, religiosity, and social support | Arch Public Health | Mental health disorders |
| 1548 | Vignery, K.; | 2022 | From networked students centrality to student networks density: What matters for student performance? | Social Networks | Not related wellbeing |
| 1549 | Demir, M.;Özen, A.;Dogan, A.; | 2012 | Friendship, Perceived Mattering and Happiness: A Study of American and Turkish College Students | Journal of Social Psychology | Not related wellbeing |
| 1550 | Igler, E. C.;Austin, J. E.;Sejkora, E. K. D.;Davies, W. H.; | 2024 | Friends' Perspective: Young Adults' Reaction to Disclosure of Chronic Illness | J Clin Psychol Med Settings | The population is not college students |
| 1551 | Muraleetharan, V.;Brault, M. A.; | 2023 | Friends as Informal Educators: The Role of Peer Relationships in Promotion of Sexual Health Services among College Students | Community Health Equity Res Policy | Mental health disorders |
| 1552 | Brannan, D.;Biswas-Diener, R.;Mohr, C. D.;Mortazavi, S.;Stein, N.; | 2013 | Friends and family: A cross-cultural investigation of social support and subjective well-being among college students | Journal of Positive Psychology | Not related wellbeing |
| 1553 | McArthur, L. H.;Fasczewski, K. S.;Wartinger, E.;Miller, J.; | 2018 | Freshmen at a University in Appalachia Experience a Higher Rate of Campus than Family Food Insecurity | J Community Health | Not related wellbeing |
| 1554 | Seo, M.;Kim, J.;Yang, H.; | 2016 | Frequent Interaction and Fast Feedback Predict Perceived Social Support: Using Crawled and Self-Reported Data of Facebook Users | Journal of Computer-Mediated Communication | Not related wellbeing |

| | | | | | |
|---|---|---|---|---|---|
| 1555 | Yang, F.;Oka, T.; | 2023 | Free from your experiences to grow: belief in free will moderates the relationship between attachment avoidance and personal growth initiative. | BMC Psychol | Not related wellbeing |
| 1556 | Seon, J.;Prock, K. A.;Bishop, J. D.;Hughes, A. K.;Woodward, A. T.;MacLean, M.; | 2019 | Formal and Informal Social Support and Academic Achievement among College Students with Unstable Childhood Experiences | Child Welfare | The population is not college students |
| 1557 | Webb, Jon R.;Hirsch, Jameson K.;Conway-Williams, Elizabeth;Brewer, Kenneth G.; | 2013 | Forgiveness and alcohol problems: Indirect associations involving mental health and social support | Addiction Research & Theory | Mental health disorders |
| 1558 | Azanaw, J.;Dagne, H.;Andualem, Z.;Adane, T.; | 2021 | Food Safety Knowledge, Attitude, and Practice of College Students, Ethiopia, 2019: a Cross-Sectional Study | BioMed research international | Not related wellbeing |
| 1559 | Cockerham, M.;Camel, S.;James, L.;Neill, D.; | 2021 | Food insecurity in baccalaureate nursing students: A cross-sectional survey | J Prof Nurs | Not related wellbeing |
| 1560 | Henry, L.;Ellis, D.;Ellis, S.;Fleck, M. J.;Migdol, S.;Rodriguez, N.;Delgado, V.;Esmonde, S.;Islam, M. I.;Kazaoka, K.;Sun, W.;Tajallipour, P.; | 2023 | Food insecurity among LGBTQIA+ college students in North Texas: Meaning, experiences, and recommendations for inclusive solutions | Journal of Agriculture, Food Systems, and Community Development | Mental health disorders |
| 1561 | Kaur, P.;Dhir, A.;Chen, S.;Rajala, R.; | 2016 | Flow in context: Development and validation of the flow experience instrument for social networking. | Computers in Human Behavior | Not related wellbeing |
| 1562 | Wu, J.;Xie, M.;Lai, Y.;Mao, Y. H.;Harmat, L.; | 2021 | Flow as a Key Predictor of Subjective Well-Being Among Chinese University Students: A Chain Mediating Model | Frontiers in Psychology | Not related wellbeing |

| | | | | | |
|---|---|---|---|---|---|
| 1563 | Pu, Y.;Liu, Y.;Qi, Y.;Yan, Z.;Zhang, X.;He, Q.; | 2023 | Five weeks of solution-focused group counseling successfully reduces internet addiction among college students: a pilot study. | Journal of Behavioral Addictions | Mental health disorders |
| 1564 | Wei, C.;Ma, Y.;Ye, J. H.;Nong, L.; | 2022 | First-Year College Students' Mental Health in the Post-COVID-19 Era in Guangxi, China: A Study Demands-Resources Model Perspective | Front Public Health | Not related wellbeing |
| 1565 | Karaman, Mehmet A.;Watson, Joshua;Freeman, Paula;Haktanır, Abdulkadir; | 2021 | First-Year College Students at a Hispanic Serving Institution: Academic Self-Concept, Social Support, and Adjustment | International Journal for the Advancement of Counselling | Not related wellbeing |
| 1566 | Hou, Z.;Zhao, X.;Ding, J.; | 2024 | First promising non-stimulant (guanfacine) transdermal patch for long-acting treatment of ADHD by solid dispersion technique | Journal of Drug Delivery Science and Technology | Mental health disorders |
| 1567 | Aase, D. M.;McManimen, S.;Hay, J.;Long, C.;Bryan, C. J.; | 2024 | Firearm ownership factors and cognitive functioning: A preliminary study | Journal of Psychiatric Research | Not related wellbeing |
| 1568 | Halperin, D. T.;Laux, J.;LeFranc-García, C.;Araujo, C.;Palacios, C.; | 2019 | Findings From a Randomized Trial of Weight Gain Prevention Among Overweight Puerto Rican Young Adults | Journal of nutrition education and behavior | The population is not college students |
| 1569 | Vignery, Kristel; | 2021 | Findings and methodologies about student networks, learning, performance, and academic achievement in higher education: A literature review on quantitative studies | Review of Education | Not related wellbeing |
| 1570 | Tran, A. G. T. T.;Lam, C. K.;Legg, E.; | 2018 | Financial Stress, Social Supports, Gender, and Anxiety During College: A Stress-Buffering Perspective ψ | Counseling Psychologist | Mental health disorders |

| | | | | | |
|---|---|---|---|---|---|
| 1571 | Lawley, K. A.;Caley, T. C. S.;Lehman, B. J.; | 2023 | Financial strain and the health and well-being of college students during the COVID-19 pandemic | J Am Coll Health | Not related wellbeing |
| 1572 | Merritt, V. C.;Rabinowitz, A. R.;Guty, E.;Meyer, J. E.;Greenberg, L. S.;Arnett, P. A.; | 2019 | Financial incentives influence ImPACT validity indices but not cognitive composite scores. | Journal of clinical and experimental neuropsychology | Not related wellbeing |
| 1573 | Walsh, J. L.;Fielder, R. L.;Carey, K. B.;Carey, M. P.; | 2013 | Female College Students' Media Use and Academic Outcomes: Results from a Longitudinal Cohort Study | Emerg Adulthood | Not related wellbeing |
| 1574 | Korovina, L.;Zaporozhets, T.;Boyechko, F.; | 2021 | FEATURES OF BEHAVIOR, DIET, ALCOHOL CONSUMPTION, SMOKING, AND PSYCHOLOGICAL STATE AND THEIR RELATIONSHIPS WITH THE ACADEMIC PERFORMANCE OF JUNIOR MEDICINE STUDENTS | Health Problems of Civilization | Mental health disorders |
| 1575 | Melnyk, B. M.;Amaya, M.;Szalacha, L. A.;Hoying, J.;Taylor, T.;Bowersox, K.; | 2015 | Feasibility, Acceptability, and Preliminary Effects of the COPE Online Cognitive-Behavioral Skill-Building Program on Mental Health Outcomes and Academic Performance in Freshmen College Students: a Randomized Controlled Pilot Study | Journal of child and adolescent psychiatric nursing | Not related wellbeing |
| 1576 | Romaniuk, M.;Saunders-Dow, E.;Brown, K.;Batterham, P. J.; | 2024 | Feasibility, acceptability, and initial outcomes of a psychological adjustment and reintegration program for transitioned military veterans | BMC Psychology | Not related wellbeing |
| 1577 | Chung, S.;Lai, J.;Hawkey, E. J.;Dvorsky, M. R.;Owens, E.;Huston, E.;Pfiffner, L. J.; | 2024 | Feasibility study of a telehealth school-based behavioral parent training group program for attention-deficit/hyperactivity disorder | Journal of Pediatric Psychology | Mental health disorders |

| | | | | | |
|---|---|---|---|---|---|
| 1578 | Lin, B.;Prickett, C.;Woltering, S.; | 2021 | Feasibility of using a biofeedback device in mindfulness training - a pilot randomized controlled trial | Pilot and Feasibility Studies | Mental health disorders |
| 1579 | Camenga, D. R.;Bernstein, S. L.;Dziura, J.;Fiellin, L.;Krishnan-Sarin, S.; | 2021 | Feasibility of text messaging to augment brief advice and nicotine replacement therapy for smoking cessation in college students | Journal of American College Health | Not related wellbeing |
| 1580 | Pope, Z. C.;Gao, Z.; | 2022 | Feasibility of smartphone application- and social media-based intervention on college students' health outcomes: a pilot randomized trial | Journal of American College Health | Mental health disorders |
| 1581 | Castro, O.;Vergeer, I.;Bennie, J.;Biddle, S. J. H.; | 2021 | Feasibility of Reducing and Breaking Up University Students' Sedentary Behaviour: Pilot Trial and Process Evaluation | Front Psychol | Not related wellbeing |
| 1582 | What all, M. C.;Patterson, A. J.;Chiu, S.;Oldmeadow, C.;Hutchesson, M. J.; | 2019 | Feasibility and Preliminary Efficacy of the Eating Advice to Students (EATS) Brief Web-Based Nutrition Intervention for Young Adult University Students: a Pilot Randomized Controlled Trial | Nutrients | Mental health disorders |
| 1583 | Marenus, M. W.;Murray, A.;Friedman, K.;Sanowski, J.;Ottensoser, H.;Cahuas, A.;Kumaravel, V.;Chen, W.; | 2021 | Feasibility and Effectiveness of the Web-Based WeActive and WeMindful Interventions on Physical Activity and Psychological Well-Being | BioMed research international | Mental health disorders |
| 1584 | Moreno, M. A.;Kerr, B.;Fairlie, A. M.;Lewis, M.; | 2023 | Feasibility and Acceptability of the Social Media-Brief Alcohol Screening and Intervention for College Students Intervention | Journal of Adolescent Health | Mental health disorders |
| 1585 | Wang, X.;Jiang, N.;Chen, S.;Tuerdi, S.;Yang, J.;Yan, R.;He, L.;Wang, J.;Li, Y.; | 2024 | Fear of progression in patients with acute myocardial infarction: a cross-sectional study | BMC Nursing | Not related wellbeing |

| | | | | | |
|---|---|---|---|---|---|
| 1586 | Mauer, V. A.;Littleton, H.;Lim, S.;Sall, K. E.;Siller, L.;Edwards, K. M.; | 2022 | Fear of COVID-19, anxiety, and social support among college students | J Am Coll Health | Mental health disorders |
| 1587 | Causey, Shakiera T.;Livingston, Jonathan;High, Benyetta; | 2015 | Family Structure, Racial Socialization, Perceived Parental Involvement, and Social Support as Predictors of Self-Esteem in African American College Students | Journal of black studies | Not related wellbeing |
| 1588 | Ko, A.;Pick, C. M.;Kwon, J. Y.;Barlev, M.;Krems, J. A.;Varnum, M. E. W.;Neel, R.;Peysha, M.;Boonyasiriwat, W.;Brandstätter, E.;Crispim, A. C.;Cruz, J. E.;David, D.;David, O. A.;de Felipe, R. P.;Fetvadjiev, V. H.;Fischer, R.;Galdi, S.;Galindo, O.;Golovina, | 2020 | Family Matters: Rethinking the Psychology of Human Social Motivation | Perspect Psychol Sci | Not related wellbeing |
| 1589 | Shi, J.;Wang, L.;Yao, Y.;Su, N.;Zhao, X.;Chen, F.; | 2017 | Family Impacts on Self-Esteem in Chinese College Freshmen | Front Psychiatry | Not related wellbeing |
| 1590 | Shi, J. Y.;Wang, L.;Yao, Y. H.;Su, N.;Zhao, X. D.;Zhan, C. Y.; | 2017 | Family Function and Self-esteem among Chinese University Students with and without Grandparenting Experience: Moderating Effect of Social Support | Frontiers in Psychology | Not related wellbeing |
| 1591 | Hussey, J. E.;Donohue, B.;Barchard, K. A.;Allen, D. N.; | 2019 | Family contributions to sports performance and their utility in predicting appropriate referrals to mental health optimization programs | European journal of sport science | Not related wellbeing |
| 1592 | Thomas, A. L.;Brausch, A. M.; | 2022 | Family and peer support moderates the relationship between distress tolerance and suicide | J Am Coll Health | Mental health disorders |

| | | | | | |
|---|---|---|---|---|---|
| | | | risk in black college students. | | |
| 1593 | Zhai, H.;Chen, L.;Yang, Y.;Sun, H.;Pan, H.;He, J.;Zhu, X.;Sui, H.;Wang, W.;Qiu, X.;Qiao, Z.;Yang, X.;Yang, J.;Yu, Y.;Ban, B.;He, C.; | 2016 | Family and College Environmental Exposures Mediate the Relationship between Parental Education and Depression among College Students | PLoS ONE | Mental health disorders |
| 1594 | Campbell, F.;Blank, L.;Cantrell, A.;Baxter, S.;Blackmore, C.;Dixon, J.;Goyder, E.; | 2022 | Factors that influence the mental health of university and college students in the UK: a systematic review | BMC Public Health | Not related wellbeing |
| 1595 | Farren, G. L.;Zhang, T.;Martin, S. B.;Thomas, K. T.; | 2017 | Factors related to meeting physical activity guidelines in active college students: A social cognitive perspective | J Am Coll Health | Not related wellbeing |
| 1596 | Brandy, J. M.;Penckofer, S.;Solari-Twadell, P. A.;Velsor-Friedrich, B.; | 2015 | Factors predictive of depression in first-year college students | J Psychosoc Nurs Ment Health Serv | Mental health disorders |
| 1597 | Johnson, N. L.;Johnson, D. M.; | 2013 | Factors influencing the relationship between sexual trauma and risky sexual behavior in college students | J Interpers Violence | Mental health disorders |
| 1598 | Wang, Z. Y.;Wong, K.; | 2022 | Factors influencing the career intentions of music performance students: An integrated model analysis | International Journal of Music Education | Not related wellbeing |
| 1599 | Alsarayreh, M.;Aljaafreh, A.; | 2023 | Factors influencing students' academic performance in universities: Mediated behavior | Knowledge Management & E-Learning-an International Journal | Not related wellbeing |

| | | | | | |
|---|---|---|---|---|---|
| 1600 | Kim, B.;Kim, H. R.;Yoo, J. Y.;Han, M. A.; | 2024 | Factors Influencing Post-Traumatic Stress Disorder in Hospital Clinical Nurses during COVID-19 in Korea: Resilience, Social Support, and Professional Pride in Nursing | Healthcare (Switzerland) | Mental health disorders |
| 1601 | Han, B.;Rideout, C.; | 2022 | Factors Associated with University Students' Development and Success: Insights from Senior Undergraduates | Canadian Journal for the Scholarship of Teaching and Learning | Not related wellbeing |
| 1602 | Richardson, S. C.;Gunn, L. H.; | 2024 | Factors Associated With Suicide Risk Behavior Outcomes Among Black Middle School Adolescents | Journal of the American Academy of Child and Adolescent Psychiatry | Mental health disorders |
| 1603 | Mao, Z.;Qin, T.;Fan, L.;Li, N.;Wang, Y.; | 2023 | Factors associated with psychological distress among college students under COVID-19 pandemic: the moderating role of coping styles and social support | Current Psychology | Mental health disorders |
| 1604 | Bell, C.;Sulaiman-Hill, R.;Tanveer, S.;Porter, R.;Dean, S.;Schluter, P. J.;Beaglehole, B.;Boden, J. M.; | 2024 | Factors associated with mental health outcomes in a Muslim community following the Christchurch terrorist attack | BJPsych Open | Not related wellbeing |
| 1605 | Liu, F.;Ye, J.;Wei, Y.;Pan, Y.;Wang, W.;Chen, J.;Zhou, T.;Wu, S.;Li, Z.;Guo, J.;Xiao, A.; | 2024 | Factors associated with a high level of suicide risk among patients with late-life depression: a cross-sectional study from a tertiary psychiatric hospital in Guangzhou China | BMC Geriatrics | Mental health disorders |
| 1606 | Abdullah, N. A.;Abu Shamsi, N.;Jenatabadi, H. S.;Ng, B. | 2022 | Factors Affecting Undergraduates' Academic Performance during COVID-19: Fear, Stress and | Sustainability | Mental health disorders |

| | | | | | |
|---|---|---|---|---|---|
| | K.;Mentri, K. A. C.; | | Teacher-Parents' Support | | |
| 1607 | Yang, Z.;Ding, Y.;Song, S.;Zhang, Y.;Li, A.;Su, M.;Xu, Y.; | 2023 | Factors Affecting the Breastfeeding Duration of Infants and Young Children in China: A Cross-Sectional Study | Nutrients | The population is not college students |
| 1608 | Hasan, S.;Fatima, M.; | 2018 | Factors affecting the Academic Performance of university students residing in Student Housing Facility | Khazar Journal of Humanities and Social Sciences | Not related wellbeing |
| 1609 | Casale, S.;Fioravanti, G.; | 2020 | Factor structure and psychometric properties of the Italian version of the fear of missing out scale in emerging adults and adolescents | Addict Behav | The population is not college students |
| 1610 | Keinert, M.;Schindler-Gmelch, L.;Rupp, L. H.;Sadeghi, M.;Capito, K.;Hager, M.;Rahimi, F.;Richer, R.;Egger, B.;Eskofier, B. M.;Berking, M.; | 2024 | Facing depression: evaluating the efficacy of the EmpkinS-EKSpression reappraisal training augmented with facial expressions – protocol of a randomized controlled trial. | BMC Psychiatry | Mental health disorders |
| 1611 | Stifter, M.;Goklish, N.;Watchman, C.;Mitchell, K.;Duncan, J.;Miller, M.;HorseChief, M.;Kemp, C. G.;Cwik, M.;Haroz, E. E.; | 2024 | Facilitators and Barriers to Implementing a Community Suicide Database and Prevention Program in Diverse Tribal Communities | International journal of environmental research and public health | Mental health disorders |
| 1612 | Do, P. T.;Moreland, J. R.; | 2014 | Facilitating the role of 3D multimodal visualization and learning rehearsal in memory recall | Psychological reports | Mental health disorders |
| 1613 | Costello, M. A.;Nagel, A. G.;Hunt, G. L.;Rivens, A. J.;Hazelwood, O. A.;Pettit, C.;Allen, J. P.; | 2022 | Facilitating connection to enhance college student well-being: evaluation of an experiential group program | American Journal of Community Psychology | Not related wellbeing |

| | | | | | |
|---|---|---|---|---|---|
| 1614 | van Twillert, E.;Hulsman, M. M. C.;Tak, L. M.; | 2024 | Facilitating and hindering factors in the treatment of persistent somatic symptoms in migrants: A scoping review | Journal of Psychosomatic Research | Not related wellbeing |
| 1615 | Julie Newman, Kingery;Bodenlos, Jamie S.;Lathrop, Jessica A.; | 2020 | Facets of dispositional mindfulness versus sources of social support predicting college students' psychological adjustment | Journal of American College Health | Mental health disorders |
| 1616 | Kingery, Julie Newman;Bodenlos, Jamie S.;Lathrop, Jessica A.; | 2020 | Facets of Dispositional Mindfulness versus Sources of Social Support Predicting College Students' Psychological Adjustment | Journal of American College Health | Mental health disorders |
| 1617 | Merchant, G.;Weibel, N.;Pina, L.;Griswold, W. G.;Fowler, J. H.;Ayala, G. X.;Gallo, L. C.;Hollan, J.;Patrick, K.; | 2017 | Face-to-Face and Online Networks: college Students' Experiences in a Weight-Loss Trial | Journal of Health Communication | Not related wellbeing |
| 1618 | Phu, B.;Gow, A. J.; | 2019 | Facebook use and its association with subjective happiness and loneliness | Computers in Human Behavior | Not related wellbeing |
| 1619 | Michikyan, M.;Subrahmanyam, K.;Dennis, J.; | 2015 | Facebook use and academic performance among college students: A mixed-methods study with a multi-ethnic sample | Computers in Human Behavior | Not related wellbeing |
| 1620 | Conley, Q.;Sadauskas, J.;Christopherson, R.;Lin, L.;Ilgaz, H.;Seto, C.;Kula, I.;Dalal, M.;Atkinson, R. K.; | 2023 | Facebook usage patterns looking into the mind via the ICAP engagement framework | Behavior and Information Technology | Not related wellbeing |
| 1621 | Billedo, C. J.;Kerkhof, P.;Finkenauer, C.;Ganzeboom, H.; | 2019 | Facebook and Face-to-Face: Examining the Short- and Long-Term Reciprocal Effects of Interactions, Perceived Social Support, and Depression among International Students | Journal of Computer-Mediated Communication | Mental health disorders |

| | | | | | |
|---|---|---|---|---|---|
| 1622 | Chan, A. S.;Leung, P. Y.;Pang, T. W. Y.;Sze, S. L.; | 2024 | Eye-tracking training improves the visuospatial working memory of children with attention-deficit/hyperactivity disorder and autism spectrum disorder. | Autism Research | The population is not college students. |
| 1623 | Yu, T.;Hu, J.; | 2022 | Extraversion and Neuroticism on College Freshmen's Depressive Symptoms During the COVID-19 Pandemic: The Mediating Role of Social Support | Frontiers in Psychiatry | Mental health disorders |
| 1624 | Fila, M. J.;Eatough, E.; | 2018 | Extending knowledge of illegitimate tasks: Student satisfaction, anxiety, and emotional exhaustion | Stress Health | Mental health disorders |
| 1625 | Grygierczyk, A. T.; | 2024 | Extending Durkheim's Sociology of suicide to healthcare decision-making: Towards a Sociology of choice as a social phenomenon of integration and Regulation | Social Theory and Health | Mental health disorders |
| 1626 | Abdulaziz Alkhalaf, S. A.; | 2022 | Expressive writing in a Saudi university English foreign language (EFL) classroom: evaluating gains in syntactic complexity | F1000Research | Not related wellbeing |
| 1627 | Pachankis, J. E.;Goldfried, M. R.; | 2010 | Expressive writing for gay-related stress: psychosocial benefits and mechanisms underlying improvement | Journal of consulting and clinical psychology | Published not from January 2010 to 31 March 2024 |
| 1628 | Kreniske, P.;Pala, A. N.;Milman, R.;Sanchez, C.; | 2019 | Expressive innovation: How first-year college students introduced emoticons to the curriculum | Cogn Dev | Not related wellbeing |
| 1629 | Teixeira, L.;Lowden, A.;Luz, A. A.;Turte, S. L.;Moreno, C. R.;Valente, D.;Nagai-Manelli, R.;Louzada, F. M.;Fischer, F. M.; | 2013 | Exposure to bright light during evening class hours increases alertness among working college students. | Sleep medicine | Not related wellbeing |

| | | | | | |
|---|---|---|---|---|---|
| 1630 | Clauss, N.;Byrd-Craven, J.; | 2019 | Exposure to a sex-specific stressor mitigates sex differences in stress-induced eating. | Physiology & behavior | Mental health disorders |
| 1631 | Dai, C. L.;Chen, C. C.;Sharma, M.; | 2023 | Exploring Yoga Behaviors among College Students Based on the Multi-Theory Model (MTM) of Health Behavior Change | Int J Environ Res Public Health | Not related wellbeing |
| 1632 | Li, N.;Zhao, S.;Liu, C.;Dai, K.;Huang, W.; | 2022 | Exploring the relationship between perceived social support and college students' autonomous fitness behavior: Chain mediating effect test | Front Psychol | Not related wellbeing |
| 1633 | Wade, J. M.;Frederick, H.;Lowe, S.;Yarrell, E.;Taylor, A.;Parker, S.; | 2024 | Exploring the racial gradient in reproductive health: an examination of challenges to sexual health care faced by black female college students | Ethn Health | Mental health disorders |
| 1634 | Kasap, Z.;Çobanoğlu Osmanlı, C.;Sarı, İ F.;Er, E.;Şahin, B.;Kulaklı, F.; | 2024 | Exploring the quantity and quality of symptoms of attention deficit hyperactivity disorder and intelligence in children with cerebral palsy: A case-control study | European Journal of Pediatrics | The population is not college students |
| 1635 | Hu, X.;Zhang, J.;Shen, S.; | 2022 | Exploring the pathway from seeking to sharing social support in e-learning: an investigation based on the norm of reciprocity and expectation confirmation theory | Curr Psychol | Not related wellbeing |
| 1636 | Zhao, J.;Wang, Y.;Kong, F.; | 2014 | Exploring the mediation effect of social support and self-esteem on the relationship between humor style and life satisfaction in Chinese college students | Personality and Individual Differences | Not related wellbeing |
| 1637 | Hoffman, Rebecca K.;Cook, Maya N.;Balaguer, Matthew T.;Gee, Christina B.; | 2023 | Exploring the Interaction Between Social Strain and Support and its Association with College Students’ Psychological Well-Being | Emerging Adulthood | Not related wellbeing |

| | | | | | |
|---|---|---|---|---|---|
| 1638 | Levy Schwartz, M.;Magzal, F.;Yehuda, I.;Tamir, S.; | 2024 | Exploring the impact of probiotics on adult ADHD management through a double-blind RCT | Scientific Reports | Mental health disorders |
| 1639 | Han, J.;Lee, H.;Kim, T.;Lee, S.; | 2024 | Exploring the Impact of Positive Psychology-Based Virtual Music Therapy on Mental Health in Stressed College Students during COVID-19: A Pilot Investigation | Healthcare (Switzerland) | Mental health disorders |
| 1640 | Kedia, P.;Mishra, L.; | 2023 | Exploring the factors influencing the effectiveness of online learning: A study on college students | Soc Sci Humanit Open | Not related wellbeing |
| 1641 | McCullock, S. P.;Perrault, E. K.; | 2020 | Exploring the Effects of Source Credibility and Message Framing on STI Screening Intentions: an Application of Prospect and Protection Motivation Theory | Journal of Health Communication | Not related wellbeing |
| 1642 | Polizzi, C. P.;Wachen, J. S.;Straud, C. L.;Mintz, J.;Baier, A. L.;Dondanville, K. A.;Young-McCaughan, S.;Litz, B. T.;Yarvis, J. S.;Peterson, A. L.;Resnick, P. A.; | 2024 | Exploring the Associations of Emotion Regulation and Trait Resilience with the Efficacy of Cognitive Processing Therapy for Active Duty Military Personnel with PTSD | Cognitive Therapy and Research | Mental health disorders |
| 1643 | Waterman, E. A.;Dworkin, E. R.;Dardis, C. M.;Ullman, S. E.;Edwards, K. M.;Rodriguez, L. M.; | 2021 | Exploring the association between anticipated and actual responses to disclosures of intimate partner violence and sexual assault | J Soc Pers Relat | Mental health disorders |
| 1644 | Benbow, R. J.;Lee, Y. G.; | 2022 | Exploring Student Service Member/Veteran Social Support and Campus Belonging in University STEMM Fields | Journal of College Student Development | Not related wellbeing |
| 1645 | Aljawarneh, Y. M.;Ghader, N.;Al-Bashaireh, A. M.;Dalky, H. | 2024 | Exploring Risk Perception, Mental Health, Mental Fatigue, Stigma, and the Quality of Life among | International journal of | Not related wellbeing |

| | | | | | |
|---|---|---|---|---|---|
| | F.;Al-Omari, H.;Alkouri, O.;Sanad, S. R.;Mheiri, N. A.;Gopakumar, A.;AlShaya, S.;Blatch, G. L.;Ghunaim, H. Y.; | | UAE Healthcare Workers during the COVID-19 Pandemic: A National Multicentric Cross-Sectional Study | environmental research and public health | |
| 1646 | Oh, Ahrim;Han, Meekyung;Choi, Young;Lau, Sing;Shum Michelle, S. W.; | 2019 | Exploring the relationship among child maltreatment experience in childhood and behavior problems as young adults: Role of social support among college students in Hong Kong | International Social Work | The population is not college students |
| 1647 | Garcia, C.;Amador Ayala, J.;Diaz Roldan, K.;Bavarian, N.; | 2022 | Exploring Reddit conversations about mental health difficulties among college students during the COVID-19 pandemic | J Am Coll Health | Not related wellbeing |
| 1648 | Zhou, H. Y.;Luo, Y. H.;Shi, L. J.;Gong, J.; | 2023 | Exploring psychological and psychosocial correlates of non-suicidal self-injury and suicide in college students using network analysis | J Affect Disord | Mental health disorders |
| 1649 | Kornbluh, M.;Wilking, J.;Roll, S.;Donatello, R.; | 2022 | Exploring housing insecurity about student success | J Am Coll Health | Not related wellbeing |
| 1650 | Painter, J. E.;DiClemente, R. J.;Jimenez, L.;Stuart, T.;Sales, J. M.;Mulligan, M. J.; | 2017 | Exploring evidence for behavioral risk compensation among participants in an HIV vaccine clinical trial | Vaccine | Mental health disorders |
| 1651 | Begum, Z.;Shaik, K.; | 2024 | Exploring Diverse Approaches for Detecting and Diagnosing Attention Deficit Hyperactivity Disorder: A Comprehensive Survey | Mathematical Modelling of Engineering Problems | Mental health disorders |
| 1652 | Poh, X. W. W.;Lim, S. S. Y.;Chew, Y. Y.;Lim-Ashworth, N. S. J.;Lim, C. G.; | 2024 | Exploring correlations between Conners' Continuous Performance Test and subjective measures of attention deficit hyperactivity disorder | Singapore medical journal | Mental health disorders |

| | | | | | |
|---|---|---|---|---|---|
| | | | symptoms in a pediatric clinical sample | | |
| 1653 | Otanga, H.;Tanhan, A.;Musili, P. M.;Arslan, G.;Bulus, M.; | 2022 | Exploring College Students' Biopsychosocial Spiritual Wellbeing and Problems during COVID-19 through a Contextual and Comprehensive Framework | International Journal of Mental Health and Addiction | Not related to research questions |
| 1654 | Adiukwu, F. N.;Adesokun, O.;Amuta-Igwe, C. M.;Metu, I.;Jack, I. C.; | 2024 | Exploring cognitive characteristics and impairments in bipolar disorder: Insights from the BiDiLoS-Ng pilot study | Global Mental Health | Mental health disorders |
| 1655 | Luca, S. D.;Yan, Y.;Schueller, D.;O'Donnell, K.; | 2024 | Exploring adolescent suicidal trajectories: The intersection of race/ethnicity, gender, and social connectedness | Journal of Adolescence | Mental health disorders |
| 1656 | Tapia, V.;Isralowitz, E. B.;Deng, K.;Nguyen, N. T.;Young, M.;Como, D. H.;Martinez, M.;Valente, T.;Cermak, S. A.; | 2022 | Exploratory analysis of college students' occupational engagement during COVID-19 | Journal of Occupational Science | Not related wellbeing |
| 1657 | Uryga, A.;Mataczyński, C.;Pelah, A. I.;Burzyńska, M.;Robba, C.;Czosnyka, M.;Zeiler, F. A.;Younsi, A.;Wolf, S.;Vilcinis, R.;Vargiolu, A.;Vajkoczy, P.;Unterberg, A.;Tenovuo, O.;Tamosuitis, T.;Takala, R.;Sundstrom, N.;Stocchetti, N.;Smielewski, P.;Sakowitz, O | 2024 | Exploration of simultaneous transients between cerebral hemodynamics and the autonomic nervous system using windowed time-lagged cross-correlation matrices: a CENTER-TBI study | Acta Neurochirurgica | Not related wellbeing |
| 1658 | Story, C. R.;Smith, E. A.;Harvey, I. S.;Thareja, G.;Hayes, J.; | 2022 | Exploration of how emotional social support predicts food insecurity among college students | J Am Coll Health | Not related wellbeing |

| | | | | | |
|---|---|---|---|---|---|
| 1659 | Varadharajan, R.;Anbalagan, P.;Saravanan, M. S.; | 2024 | EXPLOITING THE EFFICACY OF THE DRUGS USED FOR ATTENTION-DEFICIT/HYPERACTIVITY DISORDER (ADHD) TREATMENT IN ADULTS AND CHILDREN USING A NOVEL BIG DATA-DRIVEN TIME-DEPENDENT FLEXIBLE DEEP RECURRENT NETWORK MODEL (TDF-DRN) | Journal of Theoretical and Applied Information Technology | Mental health disorders |
| 1660 | Griffin, V. W.;Wentz, E.;Meinert, E.; | 2022 | Explaining the Why in #WhyIDidntReport: An Examination of Common Barriers to Formal Disclosure of Sexual Assault in College Students | J Interpers Violence | Mental health disorders |
| 1661 | Lian, S.; | 2022 | Experimental Research on Badminton Teaching of Physical Education Major Based on Deep Learning in the Multimedia Environment | Computational intelligence and neuroscience | Not related wellbeing |
| 1662 | Arigo, D.;Brown, M. M.;DiBisceglie, S.; | 2021 | Experimental effects of fitspiration messaging on body satisfaction, exercise motivation, and exercise behavior among college women and men | Translational Behavioral Medicine | Not related wellbeing |
| 1663 | Green, D.;Loprinzi, P. D.; | 2019 | Experimental Effects of Acute Exercise on Prospective Memory and False Memory | Psychological reports | Mental health disorders |
| 1664 | Frith, E.;Loprinzi, P. D.; | 2018 | Experimental effects of acute exercise and music listening on cognitive creativity | Physiology & behavior | Not related wellbeing |
| 1665 | Williamson, V.;Murphy, D.;Katona, C.;Curry, C.;Weldon, E.;Greenberg, N.; | 2024 | Experiences and impact of moral injury in human trafficking survivors: a qualitative study | BMC Psychology | Mental health disorders |
| 1666 | Kodzo, L. D.;Danso, N. A. A.;Budu, J. T.;Akriti, K. B.;Hussain, | 2024 | Experience of psychosocial rehabilitation; perspectives of depressed adolescents | European Child and Adolescent | Mental health disorders |

| | | | | | |
|---|---|---|---|---|---|
| | A.;Zhang, R.; | | | Psychiatry | |
| 1667 | Looby, A.;Earleywine, M.; | 2011 | Expectation to receive methylphenidate enhances subjective arousal but not cognitive performance. | Experimental and clinical psychopharmacolog y | Not related wellbeing |
| 1668 | Looby, A.;Zimmerman, L.;Livingston, N. R.; | 2022 | Expectation for stimulant type modifies caffeine's effects on mood and cognition among college students. | Experimental and clinical psychopharmacolog y | Not related wellbeing |
| 1669 | Looby, A.;Piccorelli, A. V.;Zimmerman, L.;Falco, C.;Livingston, N. R.;Akin, C.;Benton, S.;Juliano, L. M.; | 2024 | Expectancy for Adderall influences subjective mood and drug effects regardless of concurrent caffeine ingestion: a randomized controlled trial. | Psychopharmacolog y | Mental health disorders |
| 1670 | Toth, S. E.;Highfill, M. C.;Jenkins, I. K.;Battle, R. D.; | 2022 | Exercise stages of change and barriers among undergraduates at a historically black university | J Am Coll Health | Not related wellbeing |
| 1671 | Guo, S. Q.;Guo, K. L.;Fu, H. Y.; | 2023 | Exercise adherence and meaning in life: The chain mediating effect of perceived social support and optimism. | Social Behavior and Personality | Not related wellbeing |
| 1672 | Pardo-Salamanca, A.;Paoletti, D.;Pastor-Cerezuela, G.;De Stasio, S.;Berenguer, C.; | 2024 | Executive Functioning Profiles in Neurodevelopmental Disorders: Parent–Child Outcomes | Children | The population is not college students |
| 1673 | Bjornsdottir, E. A.;Sigurdardottir, S.;Halldorsdottir, S.; | 2024 | Excruciating existential suffering and complicated grief: The essence of surviving the suicide of a son or daughter | Scandinavian Journal of Caring Sciences | Mental health disorders |
| 1674 | Pornsakulvanich, V.; | 2018 | Excessive use of Facebook: The influence of self-monitoring and Facebook usage on social | Kasetsart Journal of Social Sciences | Not related wellbeing |

| | | | | | |
|---|---|---|---|---|---|
| | | | support | | |
| 1675 | Saleem, S.;Feng, Y.;Luqman, A.; | 2021 | Excessive SNS use at work, technological conflicts and employee performance: A social-cognitive-behavioral perspective. | Technology in Society | Not related wellbeing |
| 1676 | Medenblik, A. M.;Garner, A. R.;Basting, E. J.;Sullivan, J. A.;Jensen, M. C.;Shorey, R. C.;Stuart, G. L.; | 2024 | Examining trauma, anxiety, and depression as predictors of dropout from residential treatment for substance use disorders | Journal of Clinical Psychology | Mental health disorders |
| 1677 | Koç, T.;Turan, A. H.; | 2020 | Examining the Relationships between Gender-Specific Social Network Sites (SNSs) Addiction Patterns and Student Academic Performance | Addicta: the Turkish Journal on Addictions | Mental health disorders |
| 1678 | Leino, T.;Finserås, T. R.;Skogen, J. C.;Pallesen, S.;Kristensen, J. H.;Mentzoni, R. A.;Sivertsen, B.; | 2024 | Examining the relationship between non-suicidal self-harm and suicidality within the past 12 months and gaming problems in Norwegian full-time students | BMC Psychiatry | Mental health disorders |
| 1679 | Zielinski, M. J.;Veilleux, J. C.; | 2014 | Examining the relation between borderline personality features and social support: The mediating role of rejection sensitivity | Personality and Individual Differences | Not related wellbeing |
| 1680 | Cody, K.;Scott, J. M.;Simmer-Beck, M.; | 2022 | Examining the mental health of university students: A quantitative and qualitative approach to identifying prevalence, associations, stressors, and interventions | J Am Coll Health | Mental health disorders |
| 1681 | Rensi, M.;Barta, M.;Moreno, J.;McCullough, R.;Glaus, R.;Lundblad, R.;Ni, C. F.;Dykeman, | 2024 | Examining the Key Topics in Research Articles on Burnout Among Firefighters, Police Officers, and First Responders: A Topic Modeling Analysis | Journal of Police and Criminal Psychology | Not related wellbeing |

| | | | | | |
|---|---|---|---|---|---|
| | C.; | | | | |
| 1682 | Johnson, E. A.;Survase, S.;Gray, P. B.; | 2023 | Examining the Impact of Virtual Animal Stimuli on College Students' Affect and Perception of their Academic Advising Experience | Animals (Basel) | Not related wellbeing |
| 1683 | Menon, C. V.;Harter, S. L.; | 2012 | Examining the impact of acculturative stress on body image disturbance among Hispanic college students | Cultur Divers Ethnic Minor Psychol | Mental health disorders |
| 1684 | Scull, T. M.;Kupersmidt, J. B.;Malik, C. V.;Keefe, E. M.; | 2018 | Examining the efficacy of a mHealth media literacy education program for sexual health promotion in older adolescents attending community college | Journal of American College Health | Mental health disorders |
| 1685 | Celio, M. A.;Lisman, S. A.; | 2014 | Examining the efficacy of a personalized normative feedback intervention to reduce college student gambling | Journal of American College Health | Mental health disorders |
| 1686 | Leupold, C. R.;Lopina, E. C.;Erickson, J.; | 2020 | Examining the Effects of Core Self-Evaluations and Perceived Organizational Support on Academic Burnout Among Undergraduate Students | Psychol Rep | Not related wellbeing |
| 1687 | Alothman, A. A.;Gadelrab, H. F.;Ebrahim, M. T.;Abo-Eid, N. F.; | 2024 | Examining the effectiveness of a social-play-based program to reduce symptoms of Attention-Deficit/Hyperactivity Disorder in Saudi elementary school children | Research in Developmental Disabilities | The population is not college students |
| 1688 | Sen, K.;Laheji, N.;Ramamonjiarivelo, Z.;Renick, C.;Osborne, R.;Beauvais, B.; | 2024 | Examining the Effect of Contactless Intergenerational Befriending Intervention on Social Isolation Among Older Adults and Students' Attitude Toward Companionship: Content | JMIR Aging | The population is not college students |

| | | | | | |
|---|---|---|---|---|---|
| | | | Analysis | | |
| 1689 | Lahoti, A.;Berny, L. M.;Tanner-Smith, E. E.; | 2024 | Examining the association between posttraumatic stress disorder and sexual risk-taking in dually diagnosed adolescents | Journal of Traumatic Stress | Mental health disorders |
| 1690 | Braitman, A. L.;Strowger, M.;Lau-Barraco, C.;Shipley, J. L.;Kelley, M. L.;Carey, K. B.; | 2022 | Examining the added value of harm reduction strategies to emailed boosters to extend the effects of online interventions for college drinkers | Psychology of addictive behaviors | Mental health disorders |
| 1691 | Weigold, I. K.;Porfeli, E. J.;Weigold, A.; | 2013 | Examining tenets of personal growth initiative using the personal growth initiative scale-II | Psychol Assess | Not related wellbeing |
| 1692 | Gómez-Chica, P.;Rueda-Ruzafa, L.;Aparicio-Mota, A.;Rodriguez-Arrastia, M.;Ropero-Padilla, C.;Rodriguez-Valbuena, C.;Román, P.; | 2024 | Examining suicide risk in sexual and gender minority youth: A descriptive observational study on depressive symptoms, social support, and self-esteem | Journal of Clinical Nursing | Mental health disorders |
| 1693 | Mason, T. B.;Lewis, R. J.; | 2017 | Examining social support, rumination, and optimism concerning binge eating among Caucasian and African-American college women | Eat Weight Disord | Unpublished journal article |
| 1694 | Luther, Kate; | 2015 | Examining Social Support Among Adult Children of Incarcerated Parents | Family Relations | The population is not college students |
| 1695 | Chan, J. K. N.;Wong, C. S. M.; | 2020 | Examining risk and protective factors on the progression of romantic relational aggression among young adults: Parental control, parental care, and peer social support | Journal of Family Trauma, Child Custody and Child Development | The population is not college students |
| 1696 | Beasley, S. T.;McClain, S.; | 2021 | Examining Psychosociocultural Influences as Predictors of Black College Students' Academic | Journal of Black Psychology | Not related wellbeing |

| | | | | | |
|---|---|---|---|---|---|
| | | | Self-Concept and Achievement | | |
| 1697 | Kerimoğlu Yıldız, G.;Turk Delibalta, R.; | 2024 | Examining Predictors of Post-Traumatic Changes Among Mothers in Turkey Following Earthquakes | Disaster Medicine and Public Health Preparedness | Not related wellbeing |
| 1698 | Hallensleben, N.;Kraiss, J.;Glaesmer, H.;Forkmann, T.;Spangenberg, L.; | 2024 | Examining heterogeneity in the affect-regulating function of suicidal ideation: Person-specific analyses in male inpatients with depression. | Suicide and Life-Threatening Behavior | Mental health disorders |
| 1699 | Yu, E. A.;Chang, E. C.;Yu, T.;Bennett, S. C.;Fowler, E. E.; | 2017 | Examining Gender Differences in the Roles of Meaning in Life and Interpersonal Expectancies in Depressive Symptoms | Gender Issues | Mental health disorders |
| 1700 | Patrick, M. E.;Sur, A.;Arterberry, B.;Peterson, S.;Morrell, N.;Vock, D. M.; | 2023 | Examining engagement effects in an adaptive preventive intervention for college student drinking | Journal of consulting and clinical psychology | Mental health disorders |
| 1701 | Braitman, A. L.;Shipley, J. L.;Strowger, M.;Ayala Guzman, R.;Whiteside, A.;Bravo, A. J.;Carey, K. B.; | 2022 | Examining Emailed Feedback as Boosters After a College Drinking Intervention Among Fraternities and Sororities: Rationale and Protocol for a Remote Controlled Trial (Project Greek) | JMIR Res Protoc | Mental health disorders |
| 1702 | Baldwin, A. S.;Rothman, A. J.;Vander Weg, M. W.;Christensen, A. J.; | 2013 | Examining causal components and a mediating process underlying self-generated health arguments for exercise and smoking cessation | Health Psychology | Not related wellbeing |
| 1703 | Weinstein, S. M.;Lee, H. H.;Dziak, J. J.;Berbaum, M. L.;Zhang, T.;Avenetti, D.;Sandoval, A.;Martin, M. A.; | 2024 | Examining Caregiver- and Family-Level Psychosocial Influences on Child Oral Health Behavioral Outcomes in Racially and Economically Minoritized Urban Families | Children | The population is not college students |
| 1704 | Alshammari, T. K.;Rogowska, A. M.;Basharahil, R. F.;Alomar, S. | 2023 | Examining bedtime procrastination, study engagement, and study holism in undergraduate | Frontiers in Psychology | Not related wellbeing |

| | | | | | |
|---|---|---|---|---|---|
| | F.;Alseraye, S. S.;Al Juffali, L. A.;Alrasheed, N. M.;Alshammari, M. A.; | | students, and their association with insomnia. | | |
| 1705 | Kim, M. H.;Karr, J. E.; | 2024 | Examining associations between intelligence mindset, mental health symptom severity, and academic self-efficacy and performance | Current Psychology | Not related wellbeing |
| 1706 | Wei, C.;Suhr, J. A.; | 2015 | Examination of the Role of Expectancies on Task Performance in College Students Concerned about ADHD | Applied neuropsychology. Adult | Mental health disorders |
| 1707 | Hollerbach, B. S.;Jitnarin, N.;Koeppel, M. D. H.;Valenti, M.;Beitel, S.;Goodrich, J. M.;Burgess, J. L.;Jahnke, S. A.; | 2024 | Examination of Stress Among Recruit and Incumbent Women Firefighters | Safety and Health at Work | Mental health disorders |
| 1708 | Stroiney, D. A.;Mokris, R. L.;Hanna, G. R.;Ranney, J. D.; | 2020 | Examination of Self-Myofascial Release vs. Instrument-Assisted Soft-Tissue Mobilization Techniques on Vertical and Horizontal Power in Recreational Athletes | Journal of strength and conditioning research | Not related wellbeing |
| 1709 | Cruz, L. N.;Walker, N. C.;Rehman, S. S.;McNerney, M. W.;Madore, M. R.; | 2024 | Examination of Congruity between Subjective and Objective Working Memory in Veterans with Mild TBI and Relation to Psychiatric Symptoms and Childhood Trauma | Behavioral Sciences | The population is not college students |
| 1710 | Mangus, Lauren;Somers, Cheryl;Yoon, Jina;Partridge, Ty;Pernice, Francesca; | 2021 | Examination of College Student Achievement within an Ecological Framework | Journal of Adult and Continuing Education | Not related wellbeing |
| 1711 | Lutz, R.;Lakey, B.; | 2024 | Evidence that specific personal relationships evoke maladaptive personality expression | Anxiety Stress Coping | Not related wellbeing |

| | | | | | |
|---|---|---|---|---|---|
| 1712 | Hale, J. W.;Pacheco, J. A.;Lewis, C. S.;Swimmer, L.;Daley, S. M.;Nazir, N.;Daley, C. M.;Choi, W. S.; | 2023 | Everyday discrimination for American Indian tribal college students enrolled in the Internet All Nations Breath of Life program | J Am Coll Health | Not related wellbeing |
| 1713 | Su, X. M.;Yang, T. X.;Zuber, S.;Li, S. B.;Yuan, R. M.;Yuan, C. W.;Yang, H. X.;Wang, Y.;Chan, R. C. K.; | 2024 | Event-, time- and activity-based prospective memory in children with higher autistic traits | Research in Autism Spectrum Disorders | The population is not college students |
| 1714 | Pendry, P.;Kuzara, S.;Gee, N. R.; | 2019 | Evaluation of Undergraduate Students' Responsiveness to a 4-Week University-Based Animal-Assisted Stress Prevention Program | International journal of environmental research and public health | Mental health disorders |
| 1715 | Korkmaz, H.;Aydin, E.;Ocal, F. C. A.;Satar, B.; | 2024 | Evaluation of the Effects of Optokinetic Stimuli and Dual-Task Performance on Vestibulo-Ocular Reflex Function in Children With Attention Deficit and Hyperactivity Disorder | Clinical Otolaryngology | The population is not college students. |
| 1716 | Zhu, L.;Sun, S. Y.;Topoleski, L. D. T.;Eggleton, C.;Ma, R. H.;Madan, D.; | 2021 | Evaluation of STEM Engagement Activities on the Attitudes and Perceptions of Mechanical Engineering S-STEM Scholars | Journal of Biomechanical Engineering-Transactions of the Asme | Not related wellbeing |
| 1717 | Xin, W.;Zou, Y.;Ao, Y.;Cai, Y.;Huang, Z.;Li, M.;Xu, C.;Jia, Y.;Yang, Y.;Yang, Y.;et al.,; | 2020 | Evaluation of integrated modular teaching in Chinese ophthalmology trainee courses | BMC medical education | Not related wellbeing |
| 1718 | Syversen, A. M.;Schønning, V.;Fjellheim, G. S.;Elgen, I.;Wergeland, G. J.; | 2024 | Evaluation of dialectical behavior therapy for adolescents in routine clinical practice: a pre-post study | BMC Psychiatry | Mental health disorders |

| | | | | | |
|---|---|---|---|---|---|
| 1719 | La Marca, J. P.;Cruz, D.;Fandino, J.;Cacciaguerra, F. R.;Fresco, J. J.;Guerra, A. T.; | 2018 | Evaluation of artifact-corrected electroencephalographic (EEG) training: a pilot study | Journal of neural transmission (Vienna, Austria: 1996) | Not related wellbeing |
| 1720 | Kartal, D.;Jaeger, T.;Lamblin, M.;Richards, H.;Witt, K.;Occhipinti, J. A.;Mihalopoulos, C.;Chatterton, M. L.;Chanen, A.;McGorry, P.;Skinner, A.;Zbukvic, I.;Thompson, A.;Knott, J.;Flego, A.;Hamilton, C.;Webb, M.;Mullen, E.;Swingler, N.;Kenny, B.;Robinson, J | 2024 | Evaluation of a youth-focused suicide prevention HOPE aftercare service: protocol for a non-randomized hybrid effectiveness-implementation type I design | BMC Health Services Research | Mental health disorders |
| 1721 | Jormand, H.;Bashirian, S.;Barati, M.;Rezapur-Shahkolai, F.;Babamiri, M.; | 2022 | Evaluation of a web-based randomized controlled trial educational intervention based on media literacy on preventing substance abuse among college students, applying the integrated social marketing approach: a study protocol | Trials | Mental health disorders |
| 1722 | Mullen, M. G.;Thompson, J. L.;Murphy, A. A.;Malenczak, D.;Giacobbe, G.;Karyczak, S.;Holloway, K. E.;Twamley, E. W.;Silverstein, S. M.;Gill, K. J.; | 2017 | Evaluation of a cognitive remediation intervention for college students with psychiatric conditions | Psychiatric rehabilitation journal | Mental health disorders |
| 1723 | Clarke, N. C.;Field, M.;Rose, A. K.; | 2015 | Evaluation of a Brief Personalised Intervention for Alcohol Consumption in College Students | PLoS ONE | Mental health disorders |
| 1724 | Arazan, C.;Costelloe, M. | 2023 | Evaluation of a brief harm reduction intervention | Journal of | Mental health disorders |

| | | | | | |
|---|---|---|---|---|---|
| | T.;Willingham, M. T.; | | to reduce celebratory drinking among college students | American College Health | |
| 1725 | Zhang, Q. H.; | 2018 | Evaluation model of college students' sports learning engagement factors based on big data analysis | Kuram ve Uygulamada Egitim Bilimleri | Not related wellbeing |
| 1726 | Morris, N. M.;Mattera, J.;Golden, B.;Moses, S.;Ingram, P. B.; | 2022 | Evaluating the performance of the MMPI-3 over-reporting scales: sophisticated simulators and the effects of comorbid conditions | Clinical neuropsychologist | Not related wellbeing |
| 1727 | Galovski, T. E.;Street, A. E.;Cooney, C. C.;Winters, M. R.; | 2024 | Evaluating the Impact of a Peer Support Program on Participants' Well-Being Finding Belongingness Through the Women Veterans Network | Medical Care | Unpublished journal article |
| 1728 | Javed, A.;Yasir, M.;Majid, A.;Shah, H. A.;ul Islam, E.;Asad, S.;Khan, M. W.; | 2019 | Evaluating the effects of social networking site addiction, task distraction, and self-management on Nurses' performance | Journal of Advanced Nursing | Mental health disorders |
| 1729 | Levin, M. E.;Krafft, J.;Davis, C. H.;Twohig, M. P.; | 2021 | Evaluating the effects of guided coaching calls on engagement and outcomes for online acceptance and commitment therapy | Cognitive behavior therapy | Not related wellbeing |
| 1730 | Tolcher, K.;Cauble, M.;Downs, A.; | 2022 | Evaluating the effects of gratitude interventions on college student well-being | Journal of American College Health | Mental health disorders |
| 1731 | O'Brien, K. M.;Sauber, E. W.;Kearney, M. S.;Venaglia, R. B.;Lemay, E. P.; | 2021 | Evaluating the Effectiveness of an Online Intervention to Educate College Students About Dating Violence and Bystander Responses | Journal of Interpersonal Violence | Mental health disorders |
| 1732 | Pierce, S. V.;Haro, A. Y.;Ayón, C.;Enriquez, L. E.; | 2021 | Evaluating the Effect of Legal Vulnerabilities and Social Support on the Mental Health of | Journal of Latinos and Education | Not related wellbeing |

| | | | | | |
|---|---|---|---|---|---|
| | | | Undocumented College Students | | |
| 1733 | Miché, M.;Strippoli, M. P. F.;Preisig, M.;Lieb, R.; | 2024 | Evaluating the clinical utility of an easily applicable prediction model of suicide attempts, newly developed and validated with a general community sample of adults | BMC Psychiatry | Mental health disorders |
| 1734 | Kuchynka, S.;Findley-Van Nostrand, D.;Pollenz, R. S.; | 2019 | Evaluating Psychosocial Mechanisms Underlying STEM Persistence in Undergraduates: Scalability and Longitudinal Analysis of Three Cohorts from a Six-Day Pre-College Engagement STEM Academy Program | CBE-Life Sciences Education | Not related wellbeing |
| 1735 | Jansen, S.;Niyonzima, J. B.;Gerbarg, P.;Brown, R. P.;Nsengiyumva, A.;Niyonsenga, J.;Nsabimana, E.; | 2024 | Evaluating effects of community-based social healing model on Ubuntu, mental health and psychosocial functioning in post-genocide Rwanda: protocol for cluster randomized control trial | Trials | Unpublished journal article |
| 1736 | Hintz, S.;Frazier, P. A.;Meredith, L.; | 2015 | Evaluating an online stress management intervention for college students | Journal of Counseling Psychology | Mental health disorders |
| 1737 | Liu, J.;Teh, W. L.;Tan, R. H. S.;Chang, S. S. H.;Lau, B. J.;Chandwani, N.;Tor, P. C.;Subramaniam, M.; | 2024 | Evaluating a maladaptive personality-informed model of social support and post-traumatic stress disorder | Journal of Affective Disorders | Mental health disorders |
| 1738 | Wang, S. W.;Lau, A. S.; | 2018 | Ethnicity moderates the benefits of perceived support and emotional expressivity on stress reactivity for Asian Americans and Euro-Americans. | Cultur Divers Ethnic Minor Psychol | Mental health disorders |

| | | | | | |
|---|---|---|---|---|---|
| 1739 | Hachem, Zeinab A.;Toro, Rosa I.; | 2022 | Ethnic identity commitment and socioemotional well-being among Latinx-origin college students: The influence of maternal and peer relationships | Journal of Social and Personal Relationships | Not related wellbeing |
| 1740 | Zhao, Y.;Ding, Y.;Chekired, H.;Wu, Y.;Wang, Q.; | 2023 | Ethnic Differences in Response to COVID-19: A Study of American-Asian and Non-Asian College Students | Behav Sci (Basel) | Unpublished journal article |
| 1741 | Snyder, J. R.; | 2024 | Ethical Accompaniment and End-of-Life Care | Christian Bioethics | Not related wellbeing |
| 1742 | Chu, A. M.;So, M. K.;Chan, T. W.;Tiwari, A.; | 2020 | Estimating the dependence of mixed sensitive response types in randomized response technique | Statistical methods in medical research | Not related wellbeing |
| 1743 | Fan, Y.;Li, Y.;Dong, Z.;Ong, M.;Hope, J.; | 2024 | Entrepreneurial mental health in the wake of COVID-19 in China with an emphasis on attention deficit hyperactivity disorder (ADHD) and dyslexia analysis | Scientific Reports | Mental health disorders |
| 1744 | Baker, J. C.;Cacace, S.;Cramer, R. J.;Rasmussen, S.;Martin, C.;May, A. M.;Thomsen, C.;Bryan, A. O.;Bryan, C. J.; | 2024 | Entrapment in the military context: Factor structure and associations with suicidal thoughts and behaviors | Suicide and Life-Threatening Behavior | Mental health disorders |
| 1745 | Moscardini, E. H.;Oakey-Frost, D. N.;Robinson, A.;Powers, J.;Aboussouan, A. B.;Rasmussen, S.;Cramer, R. J.;Tucker, R. P.; | 2022 | Entrapment and suicidal ideation: The protective roles of the presence of life meaning and reasons for living | Suicide Life Threat Behav | Mental health disorders |
| 1746 | Rediger, K.;Dawson, C.;Victor, L. A.;Kverno, K.;Raymond, G.;Smyth, S.;Bennett, D.;Markus, R.;Kantsiper, M. E.;Siddiqui, Z. K.; | 2024 | Ensuring Safe and Effective Psychiatric Care in COVID-19 Alternate Care Sites | Disaster Medicine and Public Health Preparedness | Not related wellbeing |

| | | | | | |
|---|---|---|---|---|---|
| 1747 | Barnett, N. P.;Clark, M. A.;Kenney, S. R.;DiGuiseppi, G.;Meisel, M. K.;Balestrieri, S.;Ott, M. Q.;Light, J.; | 2019 | Enrollment and assessment of a first-year college class social network for a controlled trial of the indirect effect of a brief motivational intervention | Contemporary Clinical Trials | Mental health disorders |
| 1748 | Mercier, Cendrine;Zanna, Omar;Florin, Agnès; | 2022 | Enquêter à distance sur le bien-être des collégiens: Quand les usages sociaux des outils numériques sont source de biais de participation | Socio - Anthropologie | Not related wellbeing |
| 1749 | Dillard, A. J.;Hisler, G.; | 2015 | Enhancing the effects of a narrative message through experiential information processing: an experimental study | Psychology & Health | Not related wellbeing |
| 1750 | Krause, K. J.;Davis, S. E.;Yin, Z.;Schafer, K. M.;Rosenbloom, S. T.;Walsh, C. G.; | 2024 | Enhancing Suicide Attempt Risk Prediction Models with Temporal Clinical Note Features | Applied Clinical Informatics | Mental health disorders |
| 1751 | Lobato-Camacho, F. J.;López, J. C.;Vargas, J. P.; | 2024 | Enhancing spatial memory and pattern separation: Long-term effects of stimulant treatment in individuals with ADHD. | Behavioural Brain Research | Mental health disorders |
| 1752 | Miranda, O.;Kiehl, S. M.;Qi, X.;Brannock, M. D.;Kosten, T.;Ryan, N. D.;Kirisci, L.;Wang, Y.;Wang, L.; | 2024 | Enhancing post-traumatic stress disorder patient assessment: leveraging natural language processing for research of domain criteria identification using electronic medical records | BMC Medical Informatics and Decision-Making | Mental health disorders |
| 1753 | Friedman, K.;Marenus, M. W.;Murray, A.;Cahuas, A.;Ottensoser, H.;Sanowski, J.;Chen, W.; | 2022 | Enhancing Physical Activity and Psychological Well-Being in College Students during COVID-19 through WeActive and WeMindful Interventions | International journal of environmental research and public health | Mental health disorders |
| 1754 | Romero-Ayuso, D.;del Pino-González, A.;Torres-Jiménez, | 2024 | Enhancing Ecological Validity: Virtual Reality Assessment of Executive Functioning in Children | Children | Mental health disorders |

| | | | | | |
|---|---|---|---|---|---|
| | A.;Juan-González, J.;Celdrán, F. J.;Franchella, M. C.;Ortega-López, N.;Triviño-Juárez, J. M.;Garach-Gómez, A.;Arrabal-Fernández, L.;Medina-Martínez, I.;González, P.; | | and Adolescents with ADHD | | |
| 1755 | Oddo, L. E.;Meinzer, M. C.;Tang, A.;Murphy, J. G.;Vasko, J. M.;Lejuez, C. W.;Chronis-Tuscano, A.; | 2021 | Enhanced Brief Motivational Intervention for College Student Drinkers With ADHD: Goal-directed Activation as a Mechanism of Change | Behavior therapy | Mental health disorders |
| 1756 | Firouzi, M.;Kazemi, K.;Ahmadi, M.;Helfroush, M. S.;Aarabi, A.; | 2024 | Enhanced ADHD classification through deep learning and dynamic resting state fMRI analysis | Scientific Reports | Mental health disorders |
| 1757 | Kushlev, K.;Heintzelman, S. J.;Lutes, L. D.;Wirtz, D.;Oishi, S.;Diener, E.; | 2017 | ENHANCE: design and rationale of a randomized controlled trial for promoting enduring happiness & well-being | Contemporary Clinical Trials | Not related wellbeing |
| 1758 | Newman, C. B.; | 2011 | Engineering success: The role of faculty relationships with African American undergraduates | Journal of Women and Minorities in Science and Engineering | Not related wellbeing |
| 1759 | Christian, H.;Crasta, D.;Lloyd-Lester, G.;True, G.;Goodman, M.;Bass, B.;Coric, K.;Ruetten, T.;Lane, R.;Khazanov, G.; | 2024 | Engaging suicide prevention and firearm stakeholders in developing a workshop promoting secure firearm storage for suicide prevention | Injury Epidemiology | Mental health disorders |
| 1760 | Wolk, C. B.;Pieri, M.;Weiss, S. E.;Harrison, J.;Khazanov, G. | 2024 | Engaging primary care patients at risk for suicide in mental health treatment: user insights to inform | BMC Primary Care | Mental health disorders |

| | | | | | |
|---|---|---|---|---|---|
| | K.;Candon, M.;Oslin, D. W.;Press, M. J.;Anderson, E.;Famiglio, E.;Buttenheim, A.;Jager-Hyman, S.; | | implementation strategy design. | | |
| 1761 | Aronowitz, T.;Kim, B.;Vu, P.;Bergeron, A.; | 2018 | Engaging college students in substance misuse & sexual health intervention using social marketing principles | Appl Nurs Res | Mental health disorders |
| 1762 | Lindsey, M. A.;Mufson, L.;Vélez-Grau, C.;Grogan, T.;Wilson, D. M.;Reliford, A. O.;Gunlicks-Stoessel, M.;Jaccard, J.; | 2024 | Engaging Black youth in depression and suicide prevention treatment within urban schools: study protocol for a randomized controlled pilot | Trials | Mental health disorders |
| 1763 | Jaffar, A. A.;Eladl, M. A.; | 2016 | Engagement Patterns of High and Low Academic Performers on Facebook Anatomy Pages | Journal of Medical Education and Curricular Development | Not related wellbeing |
| 1764 | Purkayastha, S.;Addepally, S. A.;Bucher, S.; | 2020 | Engagement and Usability of a Cognitive Behavioral Therapy Mobile App Compared With Web-Based Cognitive Behavioral Therapy Among College Students: randomized Heuristic Trial | JMIR human factors | Not related wellbeing |
| 1765 | Abdullah, R.;Alrige, M.;Bitar, H.; | 2024 | Employing Elements of Virtual Reality to Enhance Social Skills Among Children With Attention-Deficit/Hyperactivity Disorder: Quasi-Experimental Study | TEM Journal | The population is not college students |
| 1766 | Qin, X. B.;Yang, F.;Jiang, Z. B.;Zhong, B.; | 2022 | Empathy Not Quarantined: Social Support via Social Media Helps Maintain Empathy During the COVID-19 Pandemic | Social Media + Society | Not related wellbeing |
| 1767 | Lobo, Joseph; | 2023 | Emotional Support, Academic Resiliency, and | Journal of Learning | Not related wellbeing |

| | | | | | |
|---|---|---|---|---|---|
| | | | School Engagement in an Online Learning Setting During COVID-19 Pandemic | for Development | |
| 1768 | Kirnan, J. P.;Shapiro, A. R.;Mistretta, A. J.;Sellet, M.;Fotinos, G.;Blair, B.; | 2022 | Emotional support animals supporting college students' mental health and well-being: A qualitative analysis exploring practices, policies, and perceptions | J Am Coll Health | Not related wellbeing |
| 1769 | Racine, S. E.;Hebert, K. R.;Benning, S. D.; | 2018 | Emotional Reactivity and Appraisal of Food about Eating Disorder Cognitions and Behaviours: evidence to Support the Motivational Conflict Hypothesis | European Eating Disorders Review | Mental health disorders |
| 1770 | Horne, M. J.;Allbright, M.;Galbraith, D. A.;Patel, A.; | 2024 | Emotional Intelligence in Medicine: An Investigation of the Significance for Physicians, Residents, and Medical Students – A Systematic Review | Journal of Surgical Education | Not related wellbeing |
| 1771 | Wang, H.;Wu, S.;Wang, W.;Wei, C.; | 2021 | Emotional Intelligence and Prosocial Behavior in College Students: A Moderated Mediation Analysis | Front Psychol | Not related wellbeing |
| 1772 | Kong, F.;Zhao, J.;You, X.; | 2012 | Emotional intelligence and life satisfaction in Chinese university students: The mediating role of self-esteem and social support | Personality and Individual Differences | Not related wellbeing |
| 1773 | Wijekoon, C. N.;Amaratunge, H.;de Silva, Y.;Senanayake, S.;Jayawardane, P.;Senarath, U.; | 2017 | Emotional intelligence and academic performance of medical undergraduates: a cross-sectional study in a selected university in Sri Lanka | BMC medical education | Not related wellbeing |
| 1774 | Ghislieri, C.;Sanseverino, D.;Dolce, V.;Spagnoli, P.;Manuti, A.;Ingusci, E.;Addabbo, T.; | 2023 | Emotional Exhaustion and Engagement in Higher Education Students during a Crisis, Lessons Learned from COVID-19 Experience in Italian | Social Sciences-Basel | Not related wellbeing |

| | | | | | |
|---|---|---|---|---|---|
| | | | Universities | | |
| 1775 | Goh, P. K.;A. Wong, A. W. W.;Suh, D. E.;Bodalski, E. A.;Rother, Y.;Hartung, C. M.;Lefler, E. K.; | 2024 | Emotional Dysregulation in Emerging Adult ADHD: A Key Consideration in Explaining and Classifying Impairment and Co-Occurring Internalizing Problems | Journal of Attention Disorders | Mental health disorders |
| 1776 | Nguyen-Feng, V. N.;Romano, F. N.;Frazier, P.; | 2019 | Emotional abuse moderates the efficacy of an ecological momentary stress management intervention for college students. | Journal of Counseling Psychology | Mental health disorders |
| 1777 | Yang, H. F.;Li, J. Q.;Qiu, X. Q.;Zheng, Y. L.;Song, X.; | 2021 | Emotion regulation mediates the relationship between college students' perceived social support and social anxiety. | Journal of Psychology in Africa | Mental health disorders |
| 1778 | Jia-Yuan, Z.;Xiang-Zi, J.;Yi-Nan, F.;Yu-Xia, C.; | 2022 | Emotion Management for College Students: effectiveness of a Mindfulness-Based Emotion Management Intervention on Emotional Regulation and Resilience of College Students | Journal of nervous and mental disease | Mental health disorders |
| 1779 | Lippmann, M.;Olwert, M. R.;Leistner, C. E.; | 2024 | Emerging Adults' Perspectives on Consensually Nonmonogamous Parenting | Archives of sexual behavior | The population is not college students |
| 1780 | Holt, L. J.;Latimer, L. J.; | 2024 | Emerging Adults' Experiences with E-Cigarette Cessation | Subst Use Misuse | The population is not college students |
| 1781 | Chen, I. C.;Chang, C. L.;Huang, I. W.;Chang, M. H.;Ko, L. W.; | 2024 | Electrophysiological functional connectivity and complexity reflecting cognitive processing speed heterogeneity in young children with ADHD | Psychiatry Research | Mental health disorders |
| 1782 | King, C. A.;Eisenberg, D.;Pistorello, J.;Coryell, W.;Albucher, R. C.;Favorite, T.;Horwitz, A.;Bonar, E. E.;Epstein, D.;Zheng, K.; | 2022 | Electronic bridge to mental health for college students: a randomized controlled intervention trial | Journal of consulting and clinical psychology | Mental health disorders |

| | | | | | |
|---|---|---|---|---|---|
| 1783 | Nakamura, Y.;Tsuruike, M.;Ellenbecker, T. S.; | 2016 | Electromyographic Activity of Scapular Muscle Control in Free-Motion Exercise | Journal of Athletic Training | Not related wellbeing |
| 1784 | Gibbons, R.; | 2024 | Eight 'truths' about suicide | BJPsych Bulletin | Mental health disorders |
| 1785 | Mitra, S.;Mitra, M.;Nandi, P.;Pandey, M.;Chakrabarty, M.;Saha, M.;Nandi, D. K.; | 2023 | Efficacy of Yoga for COVID-19 Stress Prophylaxis | Journal of physical activity & health | Mental health disorders |
| 1786 | Huberty, J.;Green, J.;Glissmann, C.;Larkey, L.;Puzia, M.;Lee, C.; | 2019 | Efficacy of the Mindfulness Meditation Mobile App "Calm" to Reduce Stress Among College Students: Randomized Controlled Trial | JMIR mHealth and uHealth | Mental health disorders |
| 1787 | Neighbors, C.;Rodriguez, L. M.;Rinker, D. V.;Gonzales, R. G.;Agana, M.;Tackett, J. L.;Foster, D. W.; | 2015 | Efficacy of personalized normative feedback as a brief intervention for college student gambling: a randomized controlled trial | Journal of consulting and clinical psychology | Mental health disorders |
| 1788 | Jiang, L.;Zhang, S.;Wang, Y.;So, K. F.;Ren, C.;Tao, Q.; | 2020 | Efficacy of light therapy for a college student sample with non-seasonal subthreshold depression: an RCT study | Journal of Affective Disorders | Mental health disorders |
| 1789 | Ishii-Takahashi, A.;Hamada, J.;Yamaguchi, R.;Kawahara, T.;Mukai, T.;Gustavo, S.;Shaw, P.;Ashida, S.;Koehly, L.;Tsujimoto, K.;Yoshimaru, Y.;Kano, Y.; | 2024 | Efficacy of behavioral parent training on attachment security in children with attention deficit hyperactivity disorder: a randomized controlled trial | Trials | The population is not college students |
| 1790 | LaCount, P. A.;Hartung, C. M.;Shelton, C. R.;Stevens, A. E.; | 2018 | Efficacy of an Organizational Skills Intervention for College Students With ADHD Symptomatology and Academic Difficulties | Journal of Attention Disorders | Mental health disorders |
| 1791 | Simmons, V. N.;Heckman, B. W.;Fink, A. C.;Small, B. J.;Brandon, | 2013 | Efficacy of an experiential, dissonance-based smoking intervention for college students | Journal of consulting and | Mental health disorders |

| | | | | | |
|---|---|---|---|---|---|
| | T. H.; | | delivered via the Internet | clinical psychology | |
| 1792 | DeTore, N. R.;Luther, L.;Deng, W.;Zimmerman, J.;Leathem, L.;Burke, A. S.;Nyer, M. B.;Holt, D. J.; | 2023 | Efficacy of a transdiagnostic, prevention-focused program for at-risk young adults: a waitlist-controlled trial | Psychological medicine | The population is not college students |
| 1793 | Kim, Y.;Kim, B.; | 2022 | Effects of young adults' smartphone use for social media on communication network heterogeneity, social capital, and civic engagement | Online Information Review | The population is not college students |
| 1794 | Machado, Y. D. C.;Oliveira, M.;Lima, J. L. F.;Bhargav, H.;Varambally, S.;de Miranda, D. M.;Romano-Silva, M. A.; | 2024 | Effects of yoga on impulsivity in patients with and without mental disorders: a systematic review | BMC Psychiatry | Mental health disorders |
| 1795 | Lu, T.;Guo, Z.;Li, H.;Zhang, X.;Ren, Z.;Yang, W.;Wei, L.;Huang, L.; | 2021 | Effects of Wise Intervention on Perceived Discrimination Among College Students Returning Home From Wuhan During the COVID-19 Outbreak | Front Psychol | Mental health disorders |
| 1796 | Pribis, P.;Bailey, R. N.;Russell, A. A.;Kilsby, M. A.;Hernandez, M.;Craig, W. J.;Grajales, T.;Shavlik, D. J.;Sabatè, J.; | 2012 | Effects of walnut consumption on cognitive performance in young adults | British journal of nutrition | The population is not college students |
| 1797 | Deng, W.; | 2022 | Effects of Vibration Training on Weight Loss and Heart Rate Variability in Obese Female College Students | BioMed research international | Not related wellbeing |
| 1798 | Wang, L. J.;Chao, H. R.;Chen, C. C.;Chen, C. M.;You, H. L.;Tsai, C. C.;Tsai, C. S.;Chou, W. J.;Li, C. | 2024 | Effects of urinary organophosphate flame retardants in susceptibility to attention-deficit/hyperactivity disorder in | Ecotoxicology and Environmental Safety | The population is not college students |

| | | | | | |
|---|---|---|---|---|---|
| | J.;Tsai, K. F.;Cheng, F. J.;Kung, C. T.;Li, S. H.;Wang, C. C.;Ou, Y. C.;Lee, W. C.;Huang, W. T.; | | school-age children | | |
| 1799 | Makaruk, H.;Winchester, J. B.;Sadowski, J.;Czaplicki, A.;Sacewicz, T.; | 2011 | Effects of unilateral and bilateral plyometric training on power and jumping ability in women | Journal of strength and conditioning research | Not related wellbeing |
| 1800 | Logue, E.;Leri, J.;Shahidullah, J. D.;Pinciotti, C. M.;Rathouz, P. J.;Cisler, J. M.;Newport, D. J.;Wagner, K. D.;Nemeroff, C. B.; | 2024 | Effects of trauma exposure and posttraumatic stress disorder on perceived social support in youth: A longitudinal investigation | Psychiatry Research | Mental health disorders |
| 1801 | Saidi, O.;Davenne, D.;Lehorgne, C.;Duché, P.; | 2020 | Effects of timing of moderate exercise in the evening on sleep and subsequent dietary intake in lean, young, healthy adults: randomized crossover study | European journal of applied physiology | The population is not college students |
| 1802 | Liu, H.;Chen, S.;Ji, H.;Dai, Z.; | 2023 | Effects of time-restricted feeding and walking exercise on the physical health of female college students with hidden obesity: a randomized trial | Frontiers in Public Health | Not related wellbeing |
| 1803 | Cheng, C. F.;Hsu, W. C.;Lee, C. L.;Chung, P. K.; | 2010 | Effects of the different frequencies of whole-body vibration during the recovery phase after exhaustive exercise | Journal of sports medicine and physical fitness | Published not from January 2010 to 31 March 2024 |
| 1804 | He, H.;Zhang, J.;Zhang, N.;Du, S.;Liu, S.;Ma, G.; | 2020 | Effects of the Amount and Frequency of Fluid Intake on Cognitive Performance and Mood among Young Adults in Baoding, Hebei, China: a Randomized Controlled Trial | International journal of environmental research and public health | The population is not college students |
| 1805 | Gan, Y.;Peng, J.; | 2024 | Effects of teacher support on math engagement | Children and Youth | Not related wellbeing |

| | | | | | |
|---|---|---|---|---|---|
| | | | among Chinese college students: A mediated moderation model of math self-efficacy and intrinsic value | Services Review | |
| 1806 | Xu, B.;Chen, N. S.;Chen, G.; | 2020 | Effects of teacher role on student engagement in WeChat-Based online discussion learning | Computers and Education | Not related wellbeing |
| 1807 | Shin, M.;Kim, Y.;Park, S.; | 2019 | Effects of State Anxiety and Ego Depletion on Performance Change in Golf Putting: a Hierarchical Linear Model Application | Perceptual and motor skills | Mental health disorders |
| 1808 | Hawn, S. E.;Lind, M. J.;Conley, A.;Overstreet, C. M.;Kendler, K. S.;Dick, D. M.;Amstadter, A. B.; | 2018 | Effects of social support on the association between precollege sexual assault and college-onset victimization | Journal of American College Health | Mental health disorders |
| 1809 | Zhang, Ni;Campo, Shelly;Yang, Jingzhen;Janz, Kathleen F.;Snetselaar, Linda G.;Eckler, Petya; | 2015 | Effects of Social Support About Physical Activity on Social Networking Sites: Applying the Theory of Planned Behavior | Health communication | Not related wellbeing |
| 1810 | Dogan, U.; | 2016 | Effects of Social Network Use on Happiness, Psychological Well-being, and Life Satisfaction of High School Students: Case of Facebook and Twitter | Egitim Ve Bilim-Education and Science | The population is not college students |
| 1811 | Jang, W. E.;Chun, J. W.;Kim, J. J.;Bucy, E.; | 2021 | Effects of Self-Presentation Strategy and Tie Strength on Facebook Users' Happiness and Subjective Vitality | Journal of Happiness Studies | Not related wellbeing |
| 1812 | Lee, M. S.;Kim, H. K.; | 2020 | Effects of Self-efficacy, Health Perception, Social Support perceived disability on Health-promoting Behavior of nursing students. | Medico-Legal Update | Not related wellbeing |
| 1813 | Jang, Hansori;Woo, Hongryun;Lee, | 2020 | Effects of Self-Compassion and Social Support on | Journal of | Mental health disorders |

| | | | | | |
|---|---|---|---|---|---|
| | Injung; | | Lesbian, Gay, and Bisexual College Students' Positive Identity and Career Decision-Making | Counseling and Development: JCD | |
| 1814 | Jang, H.;Woo, H.;Lee, I.; | 2020 | Effects of Self-Compassion and Social Support on Lesbian, Gay, and Bisexual College Students' Positive Identity and Career Decision-Making | Journal of Counseling and Development | Mental health disorders |
| 1815 | Nishiwaki, M.;Nakano, Y.;Matsumoto, N.; | 2019 | Effects of regular high-cocoa chocolate intake on arterial stiffness and metabolic characteristics during exercise | Nutrition (Burbank, Los Angeles County, Calif.) | Meta review |
| 1816 | Suarez, A.;Yakupova, V.; | 2024 | Effects of postpartum PTSD on maternal mental health and child socioemotional development - a two-year follow-up study | BMC Pediatrics | Mental health disorders |
| 1817 | Corkin, D. M. S.;Lindt, S. F.;Williams, P. S.; | 2021 | Effects of positive college classroom motivational environments on procrastination and achievement | Learning Environments Research | Not related wellbeing |
| 1818 | Erzen, E.;Ozabaci, N.; | 2023 | Effects of Personality Traits, Social Support, and Self-Efficacy on Predicting University Adjustment | Journal of Education-Us | Not related wellbeing |
| 1819 | Khallad, Yacoub;Jabr, Fares; | 2016 | Effects of Perceived Social Support and Family Demands on College Students' mental well-being: A cross-cultural investigation | International Journal of Psychology | Not related wellbeing |
| 1820 | Jung, H. L.;Kwak, H. E.;Kim, S. S.;Kim, Y. C.;Lee, C. D.;Byurn, H. K.;Kang, H. Y.; | 2011 | Effects of Panax ginseng supplementation on muscle damage and inflammation after uphill treadmill running in humans | American journal of Chinese medicine | Not related wellbeing |
| 1821 | Zhao, Chengjia;Xu, Huihui;Lai, Xinyi;Yang, Xue;Tu, Xiaolian;Ding, Nani;Lv, Yijun;Zhang, Guohua; | 2021 | Effects of Online Social Support and Perceived Social Support on the Relationship Between Perceived Stress and Problematic Smartphone Usage Among Chinese Undergraduates | Psychology Research and Behavior Management | Mental health disorders |

| | | | | | |
|---|---|---|---|---|---|
| 1822 | Ryoo, M.;Son, C.; | 2015 | Effects of Neurofeedback Training on EEG, Continuous Performance Task (CPT), and ADHD Symptoms in ADHD-prone College Students | Journal of Korean Academy of Nursing | Mental health disorders |
| 1823 | Mahler, H. I. M.; | 2018 | Effects of multiple viewings of an ultraviolet photo on sun protection behaviors | Public Health | Not related wellbeing |
| 1824 | Slavin, M. N.;Earleywine, M.; | 2019 | Effects of Messaging and Psychological Reactance on Marijuana Craving | Substance use & misuse | Not related wellbeing |
| 1825 | Chen, S.;Sun, P.;Wang, S.;Lin, G.;Wang, T.; | 2016 | Effects of heart rate variability biofeedback on cardiovascular responses and autonomic sympathovagal modulation following stressor tasks in prehypertensives | Journal of Human Hypertension | Mental health disorders |
| 1826 | Zhang, Z.;Chen, L.;Qin, Z.;He, J.;Gao, C.;Sun, J.;Chen, J.;Li, D.; | 2024 | Effects of functional correction training on movement patterns and physical fitness in male college students | PeerJ | Not related wellbeing |
| 1827 | Jeminiwa, R.;Garza, K. B.;Chou, C.;Franco-Watkins, A.;Fox, B. I.; | 2024 | Effects of Framed Mobile Messages on Beliefs, Intentions, Adherence, and Asthma Control: a Randomized Trial | Pharmacy (Basel, Switzerland) | Not related wellbeing |
| 1828 | Li, Y.;Fu, W.;Zhang, Q.;Chen, X.;Li, X.;Du, B.;Deng, X.;Ji, F.;Dong, Q.;Jaeggi, S. M.;et al.,; | 2023 | Effects of forward and backward span pieces of training on working memory: evidence from a randomized controlled trial | Psychophysiology | Mental health disorders |
| 1829 | Lukito, S.;Lam, S. L.;Criaud, M.;Westwood, S.;Kowalczyk, O. S.;Curran, S.;Barrett, N.;Abbott, C.;Liang, H.;Simonoff, E.;Barker, G. J.;Giampietro, V.;Rubia, K.; | 2024 | Effects of fMRI neurofeedback of right inferior frontal cortex on inhibitory brain activation in children with ADHD | Philosophical Transactions of the Royal Society B: Biological Sciences | Mental health disorders |
| 1830 | Wan, X.;Li, S.;Best, T. M.;Liu, | 2021 | Effects of flexibility and strength training on peak | Journal of sport and | Not related wellbeing |

| | | | | | |
|---|---|---|---|---|---|
| | H.;Li, H.;Yu, B.; | | hamstring musculotendinous strains during sprinting | health science | |
| 1831 | Li, S.;Garrett, W. E.;Best, T. M.;Li, H.;Wan, X.;Liu, H.;Yu, B.; | 2020 | Effects of Flexibility and Strength Interventions on Optimal Lengths of hamstring muscle-tendon units | Journal of science and medicine in sport | Mental health disorders |
| 1832 | Murad, A.;Gul, A.;Changezi, R.;Naz, A.;Khan, N.; | 2019 | Effects of Facebook Usage on the Academic Performance of the Undergraduate Students of Quetta City | Clinical Social Work and Health Intervention | Not related wellbeing |
| 1833 | Xiang, Y. H.;Dong, X.;Zhao, J. X.; | 2020 | Effects of Envy on Depression: The Mediating Roles of Psychological Resilience and Social Support | Psychiatry Investigation | Mental health disorders |
| 1834 | Marczinski, C. A.;Fillmore, M. T.;Bardgett, M. E.;Howard, M. A.; | 2011 | Effects of energy drinks mixed with alcohol on behavioral control: risks for college students consuming trendy cocktails | Alcoholism, clinical and experimental research | Mental health disorders |
| 1835 | Liu, X. H.;Zhou, M.;Guo, J. D.; | 2023 | Effects of EFL Learners' Perceived Social Support on Academic Burnout: The Mediating Role of Interaction Engagement | Sage Open | Not related wellbeing |
| 1836 | Hunt, M. G.;Momjian, A. J.;Wong, K. K.; | 2011 | Effects of diurnal variation and caffeine consumption on Test of Variables of Attention (TOVA) performance in healthy young adults | Psychological assessment | The population is not college students |
| 1837 | Zhang, N.;Du, S. M.;Zhang, J. F.;Ma, G. S.; | 2019 | Effects of Dehydration and Rehydration on Cognitive Performance and Mood among Male College Students in Guangzhou, China: a Self-Controlled Trial | International journal of environmental research and public health | Not related wellbeing |
| 1838 | Jo, I. H.;Kang, S.;Yoon, M.; | 2014 | Effects of communication competence and social | Educational | Not related wellbeing |

| | | | | | |
|---|---|---|---|---|---|
| | | | network centralities on learner performance | Technology and Society | |
| 1839 | Asadi, A.;Ramirez-Campillo, R.; | 2016 | Effects of cluster vs. traditional plyometric training sets on maximal-intensity exercise performance | Medicine (Kaunas, Lithuania) | Not related wellbeing |
| 1840 | Chang, J.;Choi, B.;Tjolleng, A.;Jung, K.; | 2017 | Effects of button position on a soft keyboard: muscle activity, touch time, and discomfort in two-thumb text entry | Applied ergonomics | Not related wellbeing |
| 1841 | Kim, D.;Ham, O. K.;Kang, C.;Jun, E.; | 2014 | Effects of auricular acupressure using Sinapsis alba seeds on obesity and self-efficacy in female college students | Journal of alternative and complementary medicine (New York, N.Y.) | Not related wellbeing |
| 1842 | Wang, X.;Pang, R.;Zhang, Y.;Zhang, C.;Sun, S.; | 2024 | Effects of Aromatherapy Massage Combined with TCM Emotional Release Technique on Maternal and Neonatal Physical and Mental Health and Family Relationships in Patients with Postpartum Depression | Alternative therapies in health and medicine | Mental health disorders |
| 1843 | Karatekin, C.;Ahluwalia, R.; | 2020 | Effects of Adverse Childhood Experiences, Stress, and Social Support on the Health of College Students | J Interpers Violence | The population is not college students |
| 1844 | Wemm, S. E.;Wulfert, E.; | 2017 | Effects of Acute Stress on Decision-Making | Applied psychophysiology and biofeedback | Mental health disorders |
| 1845 | Mou, H.;Fang, Q.;Tian, S.;Qiu, F.; | 2023 | Effects of acute exercise with different modalities on working memory in men with high and low aerobic fitness | Physiology & behavior | Mental health disorders |

| | | | | | |
|---|---|---|---|---|---|
| 1846 | Loprinzi, P.;Olafson, D.;Scavuzzo, C.;Lovorn, A.;Mather, M.;Frith, E.;Fujiwara, E.; | 2022 | Effects of acute exercise on emotional memory | Cognition & emotion | Mental health disorders |
| 1847 | Novak, E.;Soyturk, I.; | 2021 | Effects of Action Video Game Play on Arithmetic Performance in Adults | Perception | The population is not college students |
| 1848 | Burchert, S.;Alkneme, M. S.;Alsaod, A.;Cuijpers, P.;Heim, E.;Hessling, J.;Hosny, N.;Sijbrandij, M.;van't Hof, E.;Ventevogel, P.;Knaevelsrud, C.; | 2024 | Effects of a self-guided digital mental health self-help intervention for Syrian refugees in Egypt: A pragmatic randomized controlled trial | PLoS Medicine | Mental health disorders |
| 1849 | Derr, S.;Morrow, M. T.; | 2020 | Effects of a Growth Mindset of Personality on Emerging Adults' Defender Self-Efficacy, Moral Disengagement, and Perceived Peer Defending | Journal of Interpersonal Violence | The population is not college students |
| 1850 | Vinci, C.;Peltier, M. R.;Shah, S.;Kinsaul, J.;Waldo, K.;McVay, M. A.;Copeland, A. L.; | 2014 | Effects of a brief mindfulness intervention on negative affect and urge to drink among college student drinkers | Behavior research and therapy | Mental health disorders |
| 1851 | Rodriguez, L. M.;Lee, K. D. M.;Onufrak, J.;Dell, J. B.;Quist, M.;Drake, H. P.;Bryan, J.; | 2020 | Effects of a brief interpersonal conflict cognitive reappraisal intervention on improvements in access to emotion regulation strategies and depressive symptoms in college students | Psychology & Health | Mental health disorders |
| 1852 | Hung, K. C.;Chung, H. W.;Yu, C. C.;Lai, H. C.;Sun, F. H.; | 2019 | Effects of 8-week core training on core endurance and running economy | PLoS ONE | Not related wellbeing |
| 1853 | Zheng, G.;Lan, X.;Li, M.;Ling, K.;Lin, H.;Chen, L.;Tao, J.;Li, J.;Zheng, X.;Chen, B.;et al.,; | 2015 | Effectiveness of Tai Chi on Physical and Psychological Health of College Students: results of a Randomized Controlled Trial | PLoS ONE | Not related wellbeing |
| 1854 | Subburaman, N.;Parangimalai, D. | 2021 | Effectiveness of social media based oral health | Indian journal of | Not related wellbeing |

| | | | | | |
|---|---|---|---|---|---|
| | M.;Iyer, K.;Sukumaran, A.; | | promotion program among 18-20 year-old city college students - A comparative study | dental research | |
| 1855 | Dietrich, S. K.;Francis-Jimenez, C. M.;Knibbs, M. D.;Umali, I. L.;Truglio-Londrigan, M.; | 2016 | Effectiveness of sleep education programs to improve sleep hygiene and/or sleep quality in college students: a systematic review | JBI Database System Rev Implement Rep | Mental health disorders |
| 1856 | Farahmand, M.;Khalili, D.;Ramezani Tehrani, F.;Amin, G.;Negarandeh, R.; | 2020 | Effectiveness of Echium amoenum on premenstrual syndrome: a randomized, double-blind, controlled trial | BMC complementary medicine and therapies | Not related wellbeing |
| 1857 | Li, X.;Liu, H.;Kuang, M.;Li, H.;He, W.;Luo, J.; | 2022 | Effectiveness of Digital Cognitive Behavior Therapy for the Treatment of Insomnia: spillover Effects of CBT | International journal of environmental research and public health | Not related wellbeing |
| 1858 | Küchler, A. M.;Albus, P.;Ebert, D. D.;Baumeister, H.; | 2019 | Effectiveness of an internet-based intervention for procrastination in college students (StudiCare Procrastination): study protocol of a randomized controlled trial | Internet interventions | Mental health disorders |
| 1859 | Schroeder, S.;Burnis, J.;Denton, A.;Krasnow, A.;Raghu, T. S.;Mathis, K.; | 2017 | Effectiveness of Acupuncture Therapy on Stress in a Large Urban College Population | Journal of acupuncture and meridian studies | Mental health disorders |
| 1860 | Wong, W. C.;Sun, W. H.;Chia, S. M. C.;Tucker, J. D.;Mak, W. P.;Song, L.;Choi, K. W. Y.;Lau, S. T. H.;Wan, E. Y. F.; | 2020 | Effectiveness of a Peer-Led Web-Based Intervention to Improve General Self-Efficacy in Using Dating Apps Among Young Adults: randomized Clustered Trial | Journal of medical Internet research | The population is not college students |
| 1861 | Nadkarni, A.;Gandhi, Y.;Fernandes, | 2024 | Effectiveness and cost-effectiveness of community | Trials | Mental health disorders |

| | | | | | |
|---|---|---|---|---|---|
| | L.;Mirchandani, K.;Kamat, S.;Weiss, H. A.;Singla, D. R.;Velleman, R.;Lu, C.;Bhatia, U.;Biswal, B.;Sequeira, M.;D’souza, E.;Raikar, K.;Patel, V.; | | intervention in enhancing access to care and improving clinical outcomes for depression: a protocol for a cluster randomized controlled trial in India | | |
| 1862 | Chawla, G.;Azharuddin, M.;Ahmad, I.;Hussain, M. E.; | 2022 | Effect of Whole-body Vibration on Depression, Anxiety, Stress, and Quality of Life in College Students: a Randomized Controlled Trial | Oman medical journal | Mental health disorders |
| 1863 | Zhang, N.;Du, S.;Tang, Z.;Zheng, M.;Ma, G.; | 2017 | Effect of water supplementation on cognitive performances and mood among male college students in Cangzhou, China: study protocol of a randomized controlled trial | International journal of environmental research and public health | Not related wellbeing |
| 1864 | López-Cepero Borrego, J.;Estrada-Pineda, C.;Chan-Gamboa, E. C.;Fuente-Barrera, A.; | 2021 | Effect of Victimization and Perceived Support on Maintenance of Dating Relationships Among College Students in Guadalajara, Mexico | J Interpers Violence | Not related wellbeing |
| 1865 | Kumar, S. S.;Archana, R.;Mukkadan, J. K.; | 2017 | Effect of vestibular stimulation on spatial and verbal memory in college students | National Medical Journal of India | Mental health disorders |
| 1866 | López-Bedoya, J.;Vernetta-Santana, M.;Robles-Fuentes, A.;Ariza-Vargas, L.; | 2013 | Effect of three types of flexibility training on active and passive hip range of motion | Journal of sports medicine and physical fitness | Not related wellbeing |
| 1867 | Jia, M. Y.;Cheng, J. Q.; | 2024 | Effect of teacher social support on students' emotions and learning engagement: a US-Chinese classroom investigation | Humanities & Social Sciences Communications | Not related wellbeing |
| 1868 | Oh, J. K.;Shin, Y. O.;Yoon, J. H.;Kim, S. H.;Shin, H. C.;Hwang, H. J.; | 2010 | Effect of Supplementation with Ecklonia cava polyphenol on endurance performance of college students | International journal of sport nutrition and | Published not from January 2010 to 31 March 2024 |

| | | | | | |
|---|---|---|---|---|---|
| | | | | exercise metabolism | |
| 1869 | Wang, J.;Qiu, J.;Yi, L.;Hou, Z.;Benardot, D.;Cao, W.; | 2019 | Effect of sodium bicarbonate ingestion during 6 weeks of HIIT on anaerobic performance of college students | Journal of the International Society of Sports Nutrition | Not related wellbeing |
| 1870 | Saini, N.;Sangwan, G.;Verma, M.;Kohli, A.;Kaur, M.;Lakshmi, P. V. M.; | 2020 | Effect of Social Networking Sites on the Quality of Life of College Students: A Cross-Sectional Study from a City in North India | ScientificWorldJournal | Not related wellbeing |
| 1871 | Otundo, Joseph O.;MacGregor, Susan K.; | 2019 | Effect of Situational Interest and Social Support on College Students' Physical Activity Motivation: A Mixed Methods Analysis | Physical Educator | Not related wellbeing |
| 1872 | Ko, C. M.;Grace, F.;Chavez, G. N.;Grimley, S. J.;Dalrymple, E. R.;Olson, L. E.; | 2018 | Effect of Seminar on Compassion on student self-compassion, mindfulness, and well-being: a randomized controlled trial | Journal of American College Health | Mental health disorders |
| 1873 | He, J. W.;Tu, Z. H.;Xiao, L.;Su, T.;Tang, Y. X.; | 2020 | Effect of restricting bedtime mobile phone use on sleep, arousal, mood, and working memory: a randomized pilot trial | PLoS ONE | Mental health disorders |
| 1874 | Yu, Y.;Liu, S.;Song, M.;Fan, H.;Zhang, L.; | 2020 | Effect of Parent-Child Attachment on College Students' Social Anxiety: A Moderated Mediation Model | Psychol Rep | The population is not college students |
| 1875 | Lei, H.; | 2022 | Effect of Multivoice Chorus on Interpersonal Communication Disorder | Occupational therapy international | Mental health disorders |
| 1876 | Morales-Rivero, A.;Crail-Meléndez, D.;Reyes-Santos, L.;Bisanz, | 2024 | Effect of Motor Interference Therapy on Distress Related to Traumatic Memories: A Randomized, | Brain and Behavior | Mental health disorders |

| | | | | | |
|---|---|---|---|---|---|
| | E.;Bisanz, J.;Ruiz-Chow, A.;Chavarria-Medina, M. M.; | | Double-Blind, Controlled Feasibility Trial | | |
| 1877 | Bhagavan, C.;Glue, P.;Evans, W.;Reynolds, L.;Turner, T.;King, C.;Russell, B. R.;Morunga, E.;Mills, J. L.;Layton, G.;Menkes, D. B.; | 2024 | Effect of MDMA-assisted therapy on mood and anxiety symptoms in advanced-stage cancer (EMMAC): study protocol for a double-blind, randomized controlled trial | Trials | Mental health disorders |
| 1878 | Smith, T. J.;Rigassio-Radler, D.;Denmark, R.;Haley, T.;Touger-Decker, R.; | 2013 | Effect of Lactobacillus rhamnosus LGG® and Bifidobacterium animalis ssp. Lactic BB-12® on health-related Quality of life in college students affected by upper respiratory infections | British journal of nutrition | Not related wellbeing |
| 1879 | Abdolalipour, S.;Abbasalizadeh, S.;Mohammad-Alizadeh-Charandabi, S.;Abbasalizadeh, F.;Jahanfar, S.;Raphi, F.;Mirghafourvand, M.; | 2024 | Effect of implementation of the WHO intrapartum care model on maternal and neonatal outcomes: a randomized control trial | BMC Pregnancy and Childbirth | Not related wellbeing |
| 1880 | Li, X.;Wang, H.;Yang, Y.;Qi, C.;Wang, F.;Jin, M.; | 2015 | Effect of Height on Motor Coordination in college students participating in a dancesport program | Medical problems of performing artists | Not related wellbeing |
| 1881 | Tsai, T. W.;Chang, C. C.;Liao, S. F.;Liao, Y. H.;Hou, C. W.;Tsao, J. P.;Cheng, I. S.; | 2017 | Effect of green tea extract supplementation on glycogen replenishment in exercised human skeletal muscle | British journal of nutrition | Not related wellbeing |
| 1882 | Ni, S. G.;Yang, R. D.;Zhang, Y. F.;Dong, R.; | 2015 | Effect of gratitude on loneliness of Chinese college students: Social support as a mediator | Social Behavior and Personality | Not related wellbeing |
| 1883 | Xiang, Y. H.;Chao, X. M.;Ye, Y. Y.; | 2018 | Effect of Gratitude on Benign and Malicious Envy: The Mediating Role of Social Support | Frontiers in Psychiatry | Not related wellbeing |
| 1884 | Strong, D. R.;Pierce, J. P.;Pulvers, K.;Stone, M. D.;Villaseñor, A.;Pu, | 2021 | Effect of Graphic Warning Labels on Cigarette Packs on US Smokers' Cognitions and Smoking | JAMA network open | Mental health disorders |

| | | | | | |
|---|---|---|---|---|---|
| | M.;Dimofte, C. V.;Leas, E. C.;Oratowski, J.;Brighton, E.;et al.,; | | Behavior After 3 Months: a Randomized Clinical Trial | | |
| 1885 | Wang, J.;Guan, X.;Zhang, Y.;Li, Y.;Ahmed, M. Z.;Jobe, M. C.;Ahmed, O.; | 2023 | Effect of Family Cohesion on Depression of Chinese College Students in the COVID-19 Pandemic: Chain Mediation Effect of Perceived Social Support and Intentional Self-Regulation | International Journal of Mental Health Promotion | Mental health disorders |
| 1886 | Sheerin, C. M.;Konig, A.;Eonta, A. M.;Vrana, S. R.; | 2018 | Effect of expressive and neutral writing on respiratory sinus arrhythmia response over time | Journal of Behavior Therapy and Experimental Psychiatry | Not related wellbeing |
| 1887 | Zhang, W.;Xu, R.; | 2022 | Effect of Exercise Intervention on Internet Addiction and Autonomic Nervous Function in College Students | BioMed research international | Mental health disorders |
| 1888 | Shinohara, H.;Hosomi, R.;Sakamoto, R.;Urushihata, T.;Yamamoto, S.;Higa, C.;Oyama, S.; | 2023 | Effect of exercise devised to reduce arm tremor in the sighting phase of archery | PLoS ONE | Not related wellbeing |
| 1889 | Cao, W.;Qiu, J.;Cai, T.;Yi, L.;Benardot, D.;Zou, M.; | 2020 | Effect of D-ribose supplementation on delayed onset muscle soreness induced by plyometric exercise in college students | Journal of the International Society of Sports Nutrition | Not related wellbeing |
| 1890 | Schilling, J. F.;Murphy, J. C.;Bonney, J. R.;Thich, J. L.; | 2013 | Effect of core strength and endurance training on performance in college students: randomized pilot study | Journal of bodywork and movement therapies | Not related wellbeing |
| 1891 | Zhou, Guangya;Li, Yanna; | 2024 | Effect of childhood maltreatment on Internet Addiction among college students: The mediators of social support and self-esteem | Social Behavior and Personality | The population is not college students |

| | | | | | |
|---|---|---|---|---|---|
| 1892 | Lin, P. H.;Lin, Y. P.;Chen, K. L.;Yang, S. Y.;Shih, Y. H.;Wang, P. Y.; | 2021 | Effect of aromatherapy on autonomic nervous system regulation with treadmill exercise-induced stress among adolescents | PLoS ONE | Mental health disorders |
| 1893 | Bernstein, M. H.;Wood, M. D.; | 2017 | Effect of anticipatory stress on placebo alcohol consumption in a bar laboratory | American journal of drug and alcohol abuse | Mental health disorders |
| 1894 | Morgan, B.;Mirza, A. M.;Gimblet, C. J.;Ortlip, A. T.;Ancalmo, J.;Kalita, D.;Pellinger, T. K.;Walter, J. M.;Werner, T. J.; | 2023 | Effect of an 11-Week Resistance Training Program on Arterial Stiffness in Young Women | Journal of strength and conditioning research | Not related wellbeing |
| 1895 | Shetty, G. B.;Jacob, J.;Shetty, P.;Mooventhan, A.;Aryal, P.;Asha, B. K.; | 2020 | Effect of acupuncture on cognitive task performance of college students: a pilot study | Journal of complementary & integrative medicine | Not related wellbeing |
| 1896 | Charandabi, S. M.;Vahidi, R.;Marions, L.;Wahlström, R.; | 2010 | Effect of a peer-educational intervention on provider knowledge and reported performance in family planning services: a cluster randomized trial. | BMC medical education | Published not from January 2010 to 31 March 2024 |
| 1897 | Sandrick, J.;Tracy, D.;Eliasson, A.;Roth, A.;Bartel, J.;Simko, M.;Bowman, T.;Harouse-Bell, K.;Kashani, M.;Vernalis, M.; | 2017 | Effect of a Counseling Session Bolstered by Text Messaging on Self-Selected Health Behaviors in College Students: A Preliminary Randomized Controlled Trial | JMIR mHealth and uHealth | Not related wellbeing |
| 1898 | Liu, Z. X.;Glizer, D.;Tannock, R.;Woltering, S.; | 2016 | EEG alpha power during maintenance of information in working memory in adults with ADHD and its plasticity due to working memory training: a randomized controlled trial | Clinical Neurophysiology | Mental health disorders |

| | | | | | |
|---|---|---|---|---|---|
| 1899 | Hong, J. S.;Woodford, M. R.;Long, L. D.;Renn, K. A.; | 2016 | Ecological Covariates of Subtle and Blatant Heterosexist Discrimination Among LGBQ College Students | J Youth Adolesc | Not related wellbeing |
| 1900 | Hua, J.;Howell, J. L.;Olson, D.; | 2023 | Eating Together More but Feeling Worse: Discrepancies between observed and reported well-being of Latino(a/x) students at a Hispanic-Serving Institution | J Am Coll Health | Not related wellbeing |
| 1901 | Kokkonen, J.;Nelson, A. G.;Tarawhiti, T.;Buckingham, P.;Winchester, J. B.; | 2010 | Early-phase resistance training strength gains in novice lifters are enhanced by doing static stretching. | Journal of strength and conditioning research | Published not from January 2010 to 31 March 2024 |
| 1902 | Lin, P.;Zhou, X.;Zang, S.;Zhu, Y.;Zhang, L.;Bai, Y.;Wang, H.; | 2023 | Early neural markers for individual difference in mathematical achievement determined from rational number processing | Neuropsychologia | Not related wellbeing |
| 1903 | Jedličková, P.;Sleziaková, A.; | 2024 | Dynamic versus Static Diagnostics | Journal of Ecohumanism | Not related wellbeing |
| 1904 | Kim, G. S.;Lee, C. Y.;Kim, I. S.;Lee, T. H.;Cho, E.;Lee, H.;McCreary, L. L.;Kim, S. H.; | 2015 | Dyadic Effects of Individuals and Friends on Physical Activity in College Students | Public Health Nurs | Not related wellbeing |
| 1905 | Arria, A. M.;Garnier-Dykstra, L. M.;Cook, E. T.;Caldeira, K. M.;Vincent, K. B.;Baron, R. A.;O'Grady, K. E.; | 2013 | Drug use patterns in young adulthood and post-college employment | Drug and alcohol dependence | Mental health disorders |
| 1906 | Taşkan, M.;Tufan, A. E.;Öztürk, Y.;Kesikbaş, B. B.;Imrek, Y.;Akinci, B.;Koçak, G.; | 2024 | Drug Holidays May Attenuate Beneficial Effects of Treatment on Emotion Regulation and Recognition Among Children with ADHD: A Single-Center, Prospective Study | Psychiatry and Clinical Psychopharmacolog y | Mental health disorders |

| | | | | | |
|---|---|---|---|---|---|
| 1907 | Müller, V.;Mellor, D.;Pikó, B. F.; | 2024 | Dropout Intention among University Students with ADHD Symptoms: Exploring a Path Model for the Role of Self-Efficacy, Resilience, and Depression | Education Sciences | Mental health disorders |
| 1908 | Hong, S.;Jang, S. M.;Jeong, J.;Emory-Khenmy, C.; | 2024 | Drinking motives and alcohol use among undergraduate college students in Hawai‘i: A cross-sectional analysis on the moderating effects of ego-resiliency and social support | Asian Social Work and Policy Review | Mental health disorders |
| 1909 | Ramey, H. L.;Lawford, H. L.;Rose-Krasnor, L.; | 2017 | Doing for others: Youth's contributing behaviors and psychological engagement in youth-adult partnerships | Journal of Adolescence | Not related wellbeing |
| 1910 | Radcliffe, A. M.;Stevenson, J. K.;Lumley, M. A.;D'Souza, P.;Kraft, C.; | 2010 | Does Written Emotional Disclosure about Stress Improve College Students' Academic Performance? Results from Three Randomized, Controlled Studies | Journal of College Student Retention | Published not from January 2010 to 31 March 2024 |
| 1911 | Delli Paoli, A. G.;Smith, A. L.;Pontifex, M. B.; | 2017 | Does Walking Mitigate Affective and Cognitive Responses to Social Exclusion? | Journal of sport & exercise psychology | Not related wellbeing |
| 1912 | Sen-Chi, Yu; | 2020 | Does Using Social Network Sites Reduce Depression and Promote Happiness?: An Example of Facebook-Based Positive Interventions | International Journal of Technology and Human Interaction | Mental health disorders |
| 1913 | Yu, S. C.; | 2020 | Does using social network sites reduce depression and promote happiness? An example of Facebook-based positive interventions | International Journal of Technology and Human Interaction | Mental health disorders |
| 1914 | Andersson, C.;Bendtsen, M.;Lindfors, P.;Molander, | 2021 | Does the management of personal integrity information lead to differing participation rates | International journal of methods | The population is not college students |

| | | | | | |
|---|---|---|---|---|---|
| | O.;Lindner, P.;Topooco, N.;Engström, K.;Berman, A. H.; | | and response patterns in mental health surveys with young adults? A three-armed methodological experiment | in psychiatric research | |
| 1915 | Klapproth, F.;Brink, C.; | 2024 | Does students' ADHD diagnosis affect teachers' school-track decisions? An experimental study | European Journal of Psychology of Education | Mental health disorders |
| 1916 | Li, Yue;Peng, Jun; | 2021 | Does social support matter? The mediating links with coping strategy and anxiety among Chinese college students in a cross-sectional study of the COVID-19 pandemic | BMC Public Health | Mental health disorders |
| 1917 | Darling, Katherine E.;Fahrenkamp, Amy J.;Wilson, Shana M.;Karazsia, Bryan T.;Sato, Amy F.; | 2017 | Does Social Support Buffer the Association Between Stress Eating and Weight Gain During the Transition to College? Differences by Gender | Behavior Modification | Mental health disorders |
| 1918 | Carey, K. B.;DiBello, A. M.;Magill, M.;Mastroleo, N. R.; | 2024 | Does self-affirmation augment the effects of a mandated personalized feedback intervention? A randomized controlled trial with heavy-drinking college students | Psychology of addictive behaviors | Mental health disorders |
| 1919 | Barratt, J. M.;Duran, F.; | 2021 | Does psychological capital and social support impact engagement and burnout in online distance learning students? | Internet and Higher Education | Not related wellbeing |
| 1920 | Wang, P.;Garcia, E. R.;Chen, C.;Yim, I. S.; | 2023 | Does perceived support moderate the link between acculturative stress and problematic eating behaviors? The role of family, significant other, and friend support | Int J Eat Disord | Mental health disorders |
| 1921 | Brown, B. A.;Goodman, F. R.;Disabato, D. J.;Kashdan, T. | 2021 | Does negative emotion differentiation influence how people choose to regulate their distress after | Emotion | Mental health disorders |

| | | | | | |
|---|---|---|---|---|---|
| | B.;Armeli, S.;Tennen, H.; | | stressful events? A four-year daily diary study | | |
| 1922 | Chen, L. H.;Chen, M. Y.;Tsai, Y. M.; | 2012 | Does gratitude always work? Ambivalence over emotional expression inhibits the beneficial effect of gratitude on well-being. | Int J Psychol | Not related wellbeing |
| 1923 | Almanza, A. J.;Rapp, J. T.;Kierce, E. D.; | 2020 | Does Awareness of either Psychotropic Medication or Behavioral Interventions Bias College Students' Ratings of a Child's Behavior? | Developmental neurorehabilitation | The population is not college students. |
| 1924 | DuPont, C. M.;Pressman, S. D.;Reed, R. G.;Manuck, S. B.;Marsland, A. L.;Gianaros, P. J.; | 2023 | Does an Online Positive Psychological Intervention Improve Positive Affect in Young Adults During the COVID-19 Pandemic? | Affect Sci | The population is not college students. |
| 1925 | Igarashi, Y.;Suzuki, K.;Norii, T.;Motomura, T.;Yoshino, Y.;Kitagoya, Y.;Ogawa, S.;Yokobori, S.;Yokota, H.; | 2022 | Do Video Calls Improve Dispatcher-Assisted First Aid for Infants with Foreign Body Airway Obstruction? A Randomized Controlled Trial/Simulation Study | Journal of nippon medical school = nippon ika daigaku zasshi | Not related wellbeing |
| 1926 | Jensen-Campbell, L. A.;Dougall, A. L.;Heller, A. C.;Iyer-Eimerbrink, P.;Bland, M. K.;Hull, K.; | 2023 | Do Social Support and Loneliness Influence Emerging Adults' Mental Health during the First Year of the COVID-19 Pandemic? | Brain Sciences | The population is not college students. |
| 1927 | Santos, S.;Mateus, N.;Sampaio, J.;Leite, N.; | 2017 | Do previous sports experiences influence the effect of an enrichment program on basketball skills? | Journal of Sports Sciences | Not related wellbeing |
| 1928 | Saulnier, K. G.;King, C. A.;Ilgen, M. A.;Ganoczy, D.;Jagusch, J.;Garlick, J.;Abraham, K. M.;Lapidos, A.;Kim, H. M.;Vega, E.;Ahmedani, B. K.;Pfeiffer, P. N.; | 2024 | Do measures of social support and social distress share general factors associated with suicidal ideation and attempts? | Suicide and Life-Threatening Behavior | Mental health disorders |
| 1929 | Marley, Scott C.;Wilcox, M. Jeanne; | 2022 | Do family and peer academic social supports predict academic motivations and achievement of | Journal of Applied Research in Higher | Not related wellbeing |

| | | | | | |
|---|---|---|---|---|---|
| | | | first-year college students? | Education | |
| 1930 | Fernandez, A. C.;Yurasek, A. M.;Merrill, J. E.;Miller, M. B.;Zamboanga, B. L.;Carey, K. B.;Borsari, B.; | 2017 | Do brief motivational interventions reduce drinking game frequency in mandated students? An analysis of data from two randomized controlled trials | Psychology of addictive behaviors | Mental health disorders |
| 1931 | Wu, Z.;Wang, X.;Zhang, S.;Ding, N.;Zhang, G.;Zhao, C.;Xu, H.;Lai, X.;Tu, X.;Yang, X.; | 2022 | Do Attitudes, Mental Health Status, and Interpersonal Factors Predict COVID-19 Vaccine Hesitancy at the Early Phase of the Pandemic? A Longitudinal Study in Chinese College Students | Front Psychol | Not related wellbeing |
| 1932 | Chen, R.;Meng, X.;Zhao, A.;Wang, C.;Yang, C.;Li, H.;Cai, J.;Zhao, Z.;Kan, H.; | 2016 | DNA hypomethylation and its mediation in the effects of fine particulate air pollution on cardiovascular biomarkers: a randomized crossover trial | Environment International | Not related wellbeing |
| 1933 | Martalek, A.;Dubertret, C.;Fovet, T.;Le Strat, Y.;Tebeka, S.; | 2024 | Distressing memories: A continuum from wellness to PTSD | Journal of Affective Disorders | Mental health disorders |
| 1934 | Hong, L.;Lai, X.;Xu, D.;Zhang, W.;Wu, B.;Yu, X.;Zhao, K.;Zhang, G.; | 2022 | Distinct patterns of problematic smartphone use and related factors in Chinese college students | BMC Psychiatry | Mental health disorders |
| 1935 | Chidharom, M.;Carlisle, N. B.; | 2024 | Distinct mechanisms of attentional suppression: exploration of trait factors underlying cued- and learned-suppression | Cognitive Research: Principles and Implications | Mental health disorders |
| 1936 | Cabana-Domínguez, J.;Bosch, R.;Soler Artigas, M.;Alemany, S.;Llonga, N.;Vilar-Ribó, L.;Carabí-Gassol, P.;Arribas, L.;Macias-Chimborazo, | 2024 | Dissecting the polygenic contribution of attention-deficit/hyperactivity disorder and autism spectrum disorder on school performance by their relationship with educational attainment | Molecular Psychiatry | Mental health disorders |

| | | | | | |
|---|---|---|---|---|---|
| | V.;Español-Martín, G.;del Castillo, C.;Martínez, L.;Pagerols, M.;Pagespetit, È;Prat, R.;Puigbó, J.;Ramos-Quiroga, J. A.; | | | | |
| 1937 | Kingery, J. N.;Bodenlos, J. S.;Schneider, T. I.;Peltz, J. S.;Sindoni, M. W.; | 2023 | Dispositional mindfulness predicting psychological adjustment among college students: the role of rumination and gender | J Am Coll Health | Mental health disorders |
| 1938 | Momeni, M.;Ghorbani, A.;Arjeini, Z.; | 2020 | Disordered eating attitudes among Iranian university students of medical sciences: the role of body image perception | Nutrition and health (Berkhamsted, Hertfordshire) | Mental health disorders |
| 1939 | Sultana, E.;Shastry, N.;Kasarla, R.;Hardy, J.;Collado, F.;Aenlle, K.;Abreu, M.;Sisson, E.;Sullivan, K.;Klimas, N.;Craddock, T. J. A.; | 2024 | Disentangling the effects of PTSD from Gulf War Illness in male veterans via a systems-wide analysis of immune cell, cytokine, and symptom measures | Military Medical Research | Mental health disorders |
| 1940 | Wilson, S.;Derlega, V. J.;Woody, A.;Lewis, R.;Braitman, A. L.;Barbee, A.;Winstead, B. A.; | 2014 | Disentangling reactions to HIV disclosure: effects of HIV status, sexual orientation, and disclosure recipients' gender | J Health Psychol | Mental health disorders |
| 1941 | Vilar-Ribó, L.;Cabana-Domínguez, J.;Alemany, S.;Llonga, N.;Arribas, L.;Grau-López, L.;Daigre, C.;Cormand, B.;Fernàndez-Castillo, N.;Ramos-Quiroga, J. A.;Soler Artigas, M.;Ribasés, M.; | 2024 | Disentangling heterogeneity in substance use disorder: Insights from genome-wide polygenic scores | Translational Psychiatry | Mental health disorders |
| 1942 | Rogers-Sirin, L.;Sirin, S. R.;Gupta, T.; | 2016 | DISCRIMINATION-RELATED STRESS AND BEHAVIORAL ENGAGEMENT: THE MODERATING EFFECT OF POSITIVE | Education and Youth Today | Mental health disorders |

| | | | | | |
|---|---|---|---|---|---|
| | | | SCHOOL RELATIONSHIPS | | |
| 1943 | Billingsley, J. T.;Hurd, N. M.; | 2019 | Discrimination, mental health, and academic performance among underrepresented college students: the role of extracurricular activities at predominantly white institutions | Social Psychology of Education | Not related wellbeing |
| 1944 | Xu, H. B.;Yan, S. J.;Chang, Y. Y.;Jiang, H.;Zou, L.;Gan, Y.;Gong, Y. H.;Cao, S. Y.;Wang, C.;Liu, J. A.;Lu, Z. X.; | 2020 | The discrepancy in perceived social support and related factors among Chinese college students with and without siblings | Journal of Affective Disorders | Not related wellbeing |
| 1945 | Nazarov, A.;Roth, M. L.;Liu, A.;Wanklyn, S. G.;Dempster, K. S.;Plouffe, R. A.;Bird, B. M.;Fikretoglu, D.;Garber, B.;Don Richardson, J.; | 2024 | Discontinuation of mental health treatment among Canadian military personnel | Journal of Military, Veteran, and Family Health | Not related wellbeing |
| 1946 | Dworkin, E. R.;Pittenger, S. L.;Allen, N. E.; | 2016 | Disclosing Sexual Assault Within Social Networks: A Mixed-Method Investigation | Am J Community Psychol | Mental health disorders |
| 1947 | Klausli, Julia F.;Caudill, Carrie; | 2021 | Discerning Student Depression: Religious Coping and Social Support Mediating Attachment | Counseling and Values | Mental health disorders |
| 1948 | Minotti, B. J.;Ingram, K. M.;Forber-Pratt, A. J.;Espelage, D. L.; | 2021 | Disability community and mental health among college students with physical disabilities | Rehabil Psychol | Not related wellbeing |
| 1949 | Webb, J. R.;Hill, S. K.;Brewer, K. G.; | 2012 | Dimensions of Social Support as Mediators of the forgiveness-alcohol outcome relationship | Addictive Disorders and Their Treatment | Mental health disorders |
| 1950 | Ketvel, L.;Vuoksimaa, E.;Pulkkinen, L.;Rose, R. J.;Vedenkannas, | 2024 | Dimensional Attention-Deficit/Hyperactivity Disorder Symptoms and Executive Functioning in | Neuropsychology | Mental health disorders |

| | | | | | |
|---|---|---|---|---|---|
| | U.;Rapeli, P.;Raevuori, A.;Latvala, A.; | | Adolescence: A Multi-Informant, Population-Based Twin Study | | |
| 1951 | Simola, S.; | 2024 | Dignity-Infused and Trauma-Informed, Contemplative Pedagogy for Preventing Moral Injury and Promoting Wellbeing | Humanistic Management Journal | Mental health disorders |
| 1952 | Sharma, K.;Ghosh, A.;Krishnan, N. C.;Kathirvel, S.;Basu, D.;Kumar, A.;George, B. B.; | 2023 | Digital screening and brief intervention for illicit drug misuse in college students: mixed methods, pilot, cluster, randomized trial from India | Asian Journal of Psychiatry | Mental health disorders |
| 1953 | Ghosh, A.;Krishnan, N. C.;Kathirvel, S.;Pillai, R. R.;Basu, D.;George, B. B.;P, V. A.;Sharma, K.;Kumar, A.; | 2023 | Digital screening and brief intervention for alcohol misuse in college students: a pilot, mixed-methods, cluster randomized controlled trial from a low-resourced setting | Asia-Pacific psychiatry | Mental health disorders |
| 1954 | Tafesse, W.;Aguilar, M. P.;Sayed, S.;Tariq, U.; | 2024 | Digital Overload, Coping Mechanisms, and Student Engagement: An Empirical Investigation Based on the S-O-R Framework | Sage Open | Not related wellbeing |
| 1955 | Somogyi, S.;Kilencz, T.;Szőcs, K.;Klein, I.;Balogh, L.;Molnár, R.;Bálint, S.;Pulay, A. J.;Nemoda, Z.;Baradits, M.;Réthelyi, J. M.; | 2024 | Differential neurocognitive profiles in adult attention-deficit/hyperactivity disorder subtypes revealed by the Cambridge Neuropsychological Test Automated Battery | European Archives of Psychiatry and Clinical Neuroscience | Mental health disorders |
| 1956 | Sharp, K. M. H.;Schwartz, L. E.;Barnes, S. E.;Jamison, L. E.;Miller-Graff, L. E.;Howell, K. H.; | 2017 | Differential Influence of Social Support in Emerging Adulthood Across Sources of Support and Profiles of Interpersonal and Non-Interpersonal Potentially Traumatic Experiences | Journal of Aggression Maltreatment & Trauma | Not related wellbeing |
| 1957 | Doughty, A. H.;Soydan, J. A.; | 2019 | Differential derived stimulus relations across probe-trial versus adduction testing are not a | Behavioural processes | Not related wellbeing |

| | | | | | |
|---|---|---|---|---|---|
| | | | function of comparison-stimulus presentation. | | |
| 1958 | Ingkachotivanich, N.;Wongpakaran, T.;Wongpakaran, N.;Oon-Arom, A.;Karawekpanyawong, N.;Lohanan, T.;Leesawat, T.; | 2022 | Different Effects of Perceived Social Support on the Relationship between Perceived Stress and Depression among University Students with Borderline Personality Disorder Symptoms: A Multigroup Mediation Analysis | Healthcare | Mental health disorders |
| 1959 | Ridner, S. L.;Keith, R. J.;Walker, K. L.;Hart, J. L.;Newton, K. S.;Crawford, T. N.; | 2018 | Differences in quality of life among college student electronic cigarette users | AIMS Public Health | Unpublished journal article |
| 1960 | Murillo, A. G.;Gómez, G.;Durán-Agüero, S.;Parra-Soto, S. L.;Araneda, J.;Morales, G.;Ríos-Castillo, I.;Carpio-Arias, V.;Cavagnari, B. M.;Nava-González, E. J.;Bejarano-Roncancio, J. J.;Núñez-Martínez, B.;Cordón-Arrivillaga, K.;Meza-Miranda, E. R.;Mauricio-Al | 2022 | Dietary Patterns and Dietary Recommendations Achievement From Latin American College Students During the COVID-19 Pandemic Lockdown | Frontiers in Sustainable Food Systems | Not related wellbeing |
| 1961 | Philips, K. H.;Brintz, C. E.;Moss, K.;Gaylord, S. A.; | 2019 | Didgeridoo Sound Meditation for Stress Reduction and Mood Enhancement in Undergraduates: a Randomized Controlled Trial | Global advances in health and medicine | Mental health disorders |
| 1962 | Zukerman, G.;Yahav, G.;Ben-Itzchak, E.; | 2019 | Opposed associations between academic achievement and social anxiety among university students with and without autism spectrum disorder | Autism Research | Mental health disorders |

| | | | | | |
|---|---|---|---|---|---|
| 1963 | Singh, M.;Skippen, P.;He, J.;Thomson, P.;Fuelscher, I.;Caeyenberghs, K.;Anderson, V.;Hyde, C.;Silk, T. J.; | 2024 | Developmental patterns of inhibition and frontal-basal-ganglia white matter organization in healthy children and children with attention-deficit/hyperactivity disorder | Human Brain Mapping | The population is not college students |
| 1964 | de Jesus, M. C.;Dutra-Thomé, L.;Pereira, A. S.; | 2022 | Developmental assets and positive youth development in Brazilian university students | Front Psychol | Not related wellbeing |
| 1965 | Vidal, A. F. P.;Cervantes, J. A.;Rumbo-Morales, J. Y.;Sorcia-Vázquez, F. D. J.;Ortiz-Torres, G.;Moncada, C. A. C.;Arias, I. D. L. T.; | 2024 | Development of RelaxQuest: A Serious EEG-Controlled Game Designed to Promote Relaxation and Self-Regulation with a Potential Focus on ADHD Intervention | Applied Sciences (Switzerland) | Mental health disorders |
| 1966 | Park, C. L.;Williams, M. K.;Hernandez, P. R.;Agocha, V. B.;Lee, S. Y.;Carney, L. M.;Loomis, D.; | 2020 | Development of emotion regulation across the first two years of college | J Adolesc | Not related wellbeing |
| 1967 | Wu, Y.; | 2022 | Development of College Students' Resilience Scale | Biomed Res Int | Unpublished journal article |
| 1968 | Budman, J. R.;Maeir, A.; | 2024 | Development of a psychological health promotion intervention for ultra-orthodox Jewish mothers of children with ADHD using the intervention mapping protocol | BMC Public Health | Mental health disorders |
| 1969 | Yang, B.;Wang, X.;Wei, X.;Ma, J.; | 2024 | Development of a novel HER2-CAR monocyte cell therapy with controllable proliferation and enhanced anti-tumor efficacy | Chinese Medical Journal | Not related wellbeing |
| 1970 | Kim, S. S.;Gil, M.;Kim-Godwin, Y.; | 2021 | Development and Validation of the Family Relationship Assessment Scale in Korean College | Fam Process | Not related wellbeing |

| | | | | | |
|---|---|---|---|---|---|
| | | | Students' Families | | |
| 1971 | Razo, J. M.;Wickham, R. E.;Inocian, E. P.;Kra-Friedman, A. R.;Steers, M. N.; | 2023 | Development and validation of the alcohol-related content poster prototype scale: Exploring the impact of social media prototypes on college students' drinking | Alcohol Clin Exp Res (Hoboken) | Mental health disorders |
| 1972 | Rabanales-Sotos, J.;Guisado-Requena, I. M.;Leiton-Espinoza, Z. E.;Guerrero-Agenjo, C. M.;López-Torres-Hidalgo, J.;Martín-Conty, J. L.;Martín-Rodriguez, F.;López-Tendero, J.;López-González, A.; | 2022 | Development and Validation of a Novel Ultra-Compact and Cost-Effective Device for Basic Hands-On CPR Training: a Randomized, Sham-Controlled, Blinded Trial | International journal of environmental research and public health | Not related wellbeing |
| 1973 | Walsh, C. G.;Ripperger, M. A.;Hu, Y.;Sheu, Y. H.;Lee, H.;Wilimitis, D.;Zheutlin, A. B.;Rocha, D.;Choi, K. W.;Castro, V. M.;Kirchner, H. L.;Chabris, C. F.;Davis, L. K.;Smoller, J. W.; | 2024 | Development and multi-site external validation of a generalizable risk prediction model for bipolar disorder | Translational Psychiatry | Mental health disorders |
| 1974 | Ariati, J.;Hyoscyamina, D. E.; | 2017 | Development and initial validation of emotional support and achievement motivation scale as a part of the ride-space assessment. | Pertanika Journal of Social Sciences and Humanities | Not related wellbeing |
| 1975 | Witkiewitz, K.;Desai, S. A.;Bowen, S.;Leigh, B. C.;Kirouac, M.;Larimer, M. E.; | 2014 | Development and evaluation of a mobile intervention for heavy drinking and smoking among college students | Psychology of addictive behaviors | Mental health disorders |

| | | | | | |
|---|---|---|---|---|---|
| 1976 | Tindle, R.;Castillo, P.;Doring, N.;Grant, L.;Willis, R.; | 2022 | Developing and validating a university needs an instrument to measure the psychosocial needs of university students. | Br J Educ Psychol | Not related wellbeing |
| 1977 | Sibley, M. H.;Bickman, L.;Atkins, D.;Tanana, M.;Coxe, S.;Ortiz, M.;Martin, P.;King, J.;Monroy, J. M.;Ponce, T.;Cheng, J.;Pace, B.;Zhao, X.;Chawla, V.;Page, T. F.; | 2024 | Developing an Implementation Model for ADHD Intervention in Community Clinics: Leveraging Artificial Intelligence and Digital Technology | Cognitive and Behavioral Practice | Mental health disorders |
| 1978 | Tollabi, M.;Boroujeni, S. T.;Arabameri, E.;Shahbazi, M.;Lohse, K. R.; | 2024 | Determining the optimal challenge point for learning motor skills in children with attention-deficit/hyperactivity disorder | Human Movement Science | The population is not college students |
| 1979 | Yakşi, N.;Eroğlu, M.; | 2024 | Determinants of Posttraumatic Stress Disorder (PTSD) among children and adolescents in the subacute stage of Kahramanmaras earthquake, Turkey | Archives of Public Health | Mental health disorders |
| 1980 | Goh, C. F.;Rasli, A.;Tan, O. K.;Choi, S. L.; | 2019 | Determinants and academic achievement effect of Facebook use in educational communication among university students | Aslib Journal of Information Management | Not related wellbeing |
| 1981 | Nouri, A.;Tabanfar, Z.; | 2024 | Detection of ADHD Disorder in Children Using Layer-Wise Relevance Propagation and Convolutional Neural Network: An EEG Analysis | Frontiers in Biomedical Technologies | Mental health disorders |
| 1982 | Lattie, E. G.;Kornfield, R.;Ringland, K. E.;Zhang, R.;Winquist, N.;Reddy, M.; | 2020 | Designing Mental Health Technologies that Support the Social Ecosystem of College Students | Proc SIGCHI Conf Hum Factor Comput Syst | Not related wellbeing |
| 1983 | Wang, Z.;Yue, S.;Chen, X.;Li, J.;Zhu, P.;Chen, H.;Qiu, F.;Xie, | 2024 | Design of Murine Double Minute 2 Proteolysis Targeting Chimera Degraders with a Built-In | Journal of Medicinal | Not related wellbeing |

| | | | | | |
|---|---|---|---|---|---|
| | D.;Liang, Y.;Li, D.;Lu, A.;Liang, C.; | | Tumor-Targeting Ability | Chemistry | |
| 1984 | Baig, M. R.;Villarreal, G.;Aviles, L.;Meraj, A.;Davis, B.;Meyer, E. C.;Straud, C.;Young-McCaughan, S.;Peterson, A. L.;Roache, J. D.; | 2024 | Design and methodology of a randomized clinical trial of quetiapine to reduce central nervous system polypharmacy in veterans with postconcussive syndrome symptoms | Contemporary Clinical Trials | Mental health disorders |
| 1985 | Patrick, K.;Marshall, S. J.;Davila, E. P.;Kolodziejczyk, J. K.;Fowler, J. H.;Calfas, K. J.;Huang, J. S.;Rock, C. L.;Griswold, W. G.;Gupta, A.;Merchant, G.;Norman, G. J.;Raab, F.;Donohue, M. C.;Fogg, B. J.;Robinson, T. N.; | 2014 | Design and implementation of a randomized controlled social and mobile weight loss trial for young adults (project SMART) | Contemp Clin Trials | The population is not college students |
| 1986 | Sobeck, K.;Strand, G. R.;Hoffman, D. N.; | 2024 | Describing Medical Aid-in-Dying and Nursing “Leave-the-Room” Policies in California A Mixed Methods Study | Journal of Hospice and Palliative Nursing | Not related wellbeing |
| 1987 | Ananthanagu, U.;Agarwal, P.; | 2024 | DepXGBoot: Depression detection using a robust tuned extreme gradient boosting model generator | IAES International Journal of Artificial Intelligence | Mental health disorders |
| 1988 | Yoshizumi, T.;Mizutani, S.;Yamada, S.; | 2016 | Deprivation and Social Support in Mental Health of Welfare Recipients in Japan | Psychological reports | Not related wellbeing |
| 1989 | Lemon, Emily D.;Vu, Milkie;Roche, Kathleen M.;Hall, Kelli Stidham;Berg, Carla J.; | 2022 | Depressive Symptoms Concerning Adverse Childhood Experiences, Discrimination, Hope, and Social Support in a Diverse Sample of College Students | Journal of Racial and Ethnic Health Disparities | The population is not college students |
| 1990 | Lamis, Dorian A.;Ballard, Elizabeth D.;May, Alexis M.;Dvorak, Robert | 2016 | Depressive Symptoms and Suicidal Ideation in College Students: The Mediating and Moderating | Journal of Clinical Psychology | Mental health disorders |

| | | | | | |
|---|---|---|---|---|---|
| | D.; | | Roles of Hopelessness, Alcohol Problems, and Social Support | | |
| 1991 | Zeides Taubin, D.;Fogel-Grinvald, H.;Maeir, A.; | 2024 | Depressive Symptoms and Quality of Life Among Women Living With a Partner Diagnosed With ADHD | Journal of Attention Disorders | Mental health disorders |
| 1992 | Serag, R.;Hamed, M. A. M.;Mahmoud, D. A. M.;Elabd, E. F. M.;Kasem, R. E.; | 2024 | Depressive symptoms and coping patterns in a sample of Egyptian mothers of ADHD children | Middle East Current Psychiatry | Mental health disorders |
| 1993 | Tang, W. J.;Dai, Q.; | 2018 | Depressive symptoms among first-year Chinese undergraduates: The roles of socio-demographics, coping style, and social support | Psychiatry Research | Mental health disorders |
| 1994 | Dong, K. X.;Zhao, G. Z.; | 2023 | Depressive symptomatology and different dimensions of social support serially mediate the effect of negative automatic thoughts on suicidal ideation. | Current Psychology | Mental health disorders |
| 1995 | Noel, J. K.;Lakhan, H. A.;Sammartino, C. J.;Rosenthal, S. R.; | 2023 | Depressive and anxiety symptoms in first-generation college students | J Am Coll Health | Mental health disorders |
| 1996 | Klausli, Julia;Caudill, Carrie; | 2018 | Depression for college students in a traditional Christian culture context: the role of attachment, spirituality, and social support | Mental Health, Religion & Culture | Mental health disorders |
| 1997 | Tang, Zaili;Feng, Shuidong;Lin, Jing; | 2021 | Depression and its correlation with social support and health-promoting lifestyles among Chinese university students: a cross-sectional study | BMJ Open | Mental health disorders |
| 1998 | Ceballos, N. A.;Petrofes, C.;Bitney, C.;Graham, R.;Howard, K.; | 2024 | Denial, Attention-Seeking, and Posting Online While Intoxicated: Three Key Predictors of | Cyberpsychol Behav Soc Netw | Mental health disorders |

| | | | | | |
|---|---|---|---|---|---|
| | | | Collegiate Sadfishing | | |
| 1999 | Kamau, S. C.; | 2017 | DEMOCRATIC ENGAGEMENT IN THE DIGITAL AGE: YOUTH, SOCIAL MEDIA AND PARTICIPATORY POLITICS IN KENYA | Communication-South African Journal for Communication Theory and Research | Not related wellbeing |
| 2000 | Flori, J. N.;Schreiner, A. M.;Dunn, M. E.;Crisafulli, M. J.;Lynch, G. T.;Dvorak, R. D.;Davis, C. A.; | 2023 | Delivery of a Prevention Program in Large College Classes: effectiveness of the Expectancy Challenge Alcohol Literacy Curriculum | Substance use & misuse | Mental health disorders |
| 2001 | Dickason, C. N.;Heinrich, C.;Smith, M.; | 2024 | Delivering on the Promise: The Role of Supplemental Promise Programs in Reducing Barriers to College Success | Journal of Higher Education | Not related wellbeing |
| 2002 | Merrill, J. E.;Boyle, H. K.;Barnett, N. P.;Carey, K. B.; | 2018 | Delivering normative feedback to heavy drinking college students via text messaging: a pilot feasibility study | Addictive behaviors | Not related wellbeing |
| 2003 | Fitzpatrick, K. K.;Darcy, A.;Vierhile, M.; | 2017 | Delivering Cognitive Behavior Therapy to Young Adults With Symptoms of Depression and Anxiety Using a Fully Automated Conversational Agent (Woebot): A Randomized Controlled Trial | JMIR mental health | The population is not college students |
| 2004 | Austgulen, A.;Posserud, M. B.;Hysing, M.;Haavik, J.;Lundervold, A. J.; | 2024 | Deliberate self-harm in adolescents screening positive for attention-deficit / hyperactivity disorder: a population-based study | BMC Psychiatry | Mental health disorders |
| 2005 | Liao, Z.;Zhang, X.;Wang, Y.;Wang, T.;Li, X.;Zhao, M.;Zhuang, Q.; | 2021 | Delayed-Onset PTSD and Coping Strategies of Chinese College Students During the COVID-19 Pandemic | Front Social | Mental health disorders |

| | | | | | |
|---|---|---|---|---|---|
| 2006 | Henson, J. M.;Pearson, M. R.;Carey, K. B.; | 2015 | Defining and characterizing differences in college alcohol intervention efficacy: a growth mixture modeling application | Journal of consulting and clinical psychology | Mental health disorders |
| 2007 | Leon, C.;Kaur, S.;Sagar, R.;Tayade, P.;Sharma, R.; | 2024 | Default at fault? Exploring neural correlates of default mode network in children with ADHD, their unaffected siblings versus neurotypical controls: A quantitative EEG study | Asian Journal of Psychiatry | Mental health disorders |
| 2008 | Adekkanattu, P.;Furmanchuk, A.;Wu, Y.;Pathak, A.;Patra, B. G.;Bost, S.;Morrow, D.;Wang, G. H. M.;Yang, Y.;Forrest, N. J.;Luo, Y.;Walunas, T. L.;Lo-Ciganic, W.;Gelad, W.;Bian, J.;Bao, Y.;Weiner, M.;Oslin, D.;Pathak, J.; | 2024 | Deep learning for identifying personal and family history of suicidal thoughts and behaviors from EHRs | npj Digital Medicine | Mental health disorders |
| 2009 | Zhang, J.; | 2024 | Dealing with Service Failures and Complaints on Social Media: The Role of Online Social Support in China | Journal of Social Service Research | Not related wellbeing |
| 2010 | Ripoll-Núñez, K.;Gordon, K. C.; | 2024 | Dealing with couple infidelity in romantic relationships: A group intervention feasibility study | Family Process | Mental health disorders |
| 2011 | Kaukinen, C.; | 2014 | Dating violence among college students: the risk and protective factors | Trauma Violence Abuse | Mental health disorders |
| 2012 | Dalla Rosa, A.;Vianello, M.;Galliani, E. M.;Boffo, V.;Fedeli, M.;Lo Presti, F.;Melacarne, C.; | 2019 | Data from a three-wave complete longitudinal design survey on career calling and related constructs (N = 6368) | Data Brief | Not related wellbeing |
| 2013 | Poddar, K. H.;Hosig, K. | 2012 | Dairy intake and related self-regulation improved | Journal of the | Not related wellbeing |

| | | | | | |
|---|---|---|---|---|---|
| | W.;Anderson-Bill, E. S.;Nickols-Richardson, S. M.;Duncan, S. E.; | | in college students using online nutrition education. | Academy of Nutrition and Dietetics | |
| 2014 | Mournet, A. M.;Kellerman, J. K.;Yeager, A. L.;Rosen, R. L.;Kim, J. S.;Kleiman, E. M.; | 2022 | Daily-level assessment of the contexts under which seeking social support relates to the risk of suicidal thinking. | Suicide and Life-Threatening Behavior | Mental health disorders |
| 2015 | Aldridge-Gerry, A. A.;Roesch, S. C.;Villodas, F.;McCabe, C.;Leung, Q. K.;Da Costa, M.; | 2011 | Daily stress and alcohol consumption: modeling between-person and within-person ethnic variation in coping behavior | J Stud Alcohol Drugs | Mental health disorders |
| 2016 | Patrick, M. E.;Yeomans-Maldonado, G.;Griffin, J.; | 2016 | Daily Reports of Positive and Negative Affect and Alcohol and Marijuana Use Among College Student and Nonstudent Young Adults | Substance use & misuse | The population is not college students |
| 2017 | Zhou, Yuan;Meng, Xiao;Wang, Jiayin;Xu, Mo;Jiang, Sa;Dai, Chengjun;Liu, Mengting; | 2023 | Daily Peer Relationships and Academic Achievement among College Students: A Social Network Analysis Based on Behavioral Big Data | Sustainability | Not related wellbeing |
| 2018 | Emma, Armstrong-Carter;João, F. Guassi Moreira;Ivory, Susannah L.;Telzer, Eva H.; | 2020 | Daily Links Between Helping Behaviors and Emotional Well-Being During Late Adolescence | Journal of Research on Adolescence | Not related wellbeing |
| 2019 | Armstrong-Carter, E.;Guassi Moreira, J. F.;Ivory, S. L.;Telzer, E. H.; | 2020 | Daily Links Between Helping Behaviors and Emotional Well-Being During Late Adolescence | J Res Adolesc | Not related wellbeing |
| 2020 | Browning, Mhem;Shin, S.;Drong, G.;McAnirlin, O.;Gagnon, R. J.;Ranganathan, S.;Sindelar, K.;Hoptman, D.;Bratman, G. N.;Yuan, S.;et al.,; | 2023 | Daily exposure to virtual nature reduces symptoms of anxiety in college students. | Scientific Reports | Mental health disorders |

| | | | | | |
|---|---|---|---|---|---|
| 2021 | Kim, S. Y.;Suh, H.;Oh, W.;Daheim, J.; | 2021 | Daily change patterns in mindfulness and psychological health: a pilot intervention | Journal of Clinical Psychology | Mental health disorders |
| 2022 | Heiman, T.;Olenik-Shemesh, D.; | 2022 | Cyber-Victimization Experience among Higher Education Students: Effects of Social Support, Loneliness, and Self-Efficacy | International journal of environmental research and public health | Not related wellbeing |
| 2023 | Fang, Jie;Wang, Xingchao;Wen, Zhonglin;Huang, Jiayan; | 2020 | Cyber victimization and loneliness among Chinese college students: A moderated mediation model of rumination and online social support | Children and Youth Services Review | Not related wellbeing |
| 2024 | Tang, X.;Duan, W.; | 2023 | Cyber-ostracism mediates the relationship between perceived stress and emotional well-being among college students. | Journal of American College Health | Mental health disorders |
| 2025 | McComas, M.;Gil-Rivas, V.; | 2023 | Cyberaggression victimization on anxiety and depression in college students: the role of emotion regulation, social media, social support, and biological sex | J Am Coll Health | Mental health disorders |
| 2026 | Zheng, Y.;Zheng, X.; | 2015 | Current state and recent developments of child psychiatry in China | Child Adolesc Psychiatry Ment Health | The population is not college students |
| 2027 | Xue, F.;Suh, H. N.;Rice, K. G.;Ashby, J. S.; | 2023 | Cumulative Trauma and Trauma Symptoms: A Three-Way Interaction | Behav Sci (Basel) | Unpublished journal article |
| 2028 | Salgado García, F.;Bursac, Z.;Derefinko, K. J.; | 2020 | Cumulative Risk of Substance Use in Community College Students | Am J Addict | Not related wellbeing |
| 2029 | Guan, S. S. A.;Chiang, J. J.;Sherman, L. E.;Nguyen, J.;Tsui, Y. L.;Robles, | 2017 | Culture moderates the effect of social support across communication contexts in young adult | Computers in Human Behavior | Not related wellbeing |

| | | | | | |
|---|---|---|---|---|---|
| | T. F.; | | women in the United States. | | |
| 2030 | Hubbard, R. R.;Palmberg, A.;Lydecker, J.;Green, B.;Kelly, N. R.;Trapp, S.;Bean, M. K.; | 2016 | Culturally-Based Communication about Health, Eating, and Food: Development and validation of the CHEF scale | Appetite | Unpublished journal article |
| 2031 | Wang, S. W.;Shih, J. H.;Hu, A. W.;Louie, J. Y.;Lau, A. S.; | 2010 | Cultural differences in daily support experiences | Cultur Divers Ethnic Minor Psychol | Published not from January 2010 to 31 March 2024 |
| 2032 | Eskin, M.;Baydar, N.;Harlak, H.;Hamdan, M.;Mechri, A.;Isayeva, U.;Abdel-Khalek, A. M.;Rezaeian, M.;Asad, N.;El-Nayal, M.;Buhairan, F. A.;Noor, I. M.;Khader, Y.;Khan, A.;Sayyari, A. A.;Khader, A.;Behzadi, B.;Öztürk, CŞ;Agha, H.;Hendarmin, L. A.;Khan, M. M.; | 2021 | Cultural and interpersonal risk factors for suicide ideation and suicide attempts among Muslim college students from 11 nations | J Affect Disord | Mental health disorders |
| 2033 | Rivera-Rivera, N.;Calaf, M.;Pérez-Pedrogo, C.;Sánchez-Cardona, I.;Villegas-Adorno, Y.; | 2024 | Cultural Adaptation of Skills Training in Affective and Interpersonal Regulation Therapy for Spanish-speaking Caribbean Veterans at the VA Caribbean Healthcare System: A preliminary study of feasibility and acceptability in a Primary Care setting | Interamerican Journal of Psychology | Not related wellbeing |
| 2034 | Yu, H.;Li, X.; | 2022 | Cultivation of Positive Psychological Quality of College Students' English Learning Under the Online and Offline Teaching Mode During the Epidemic | Front Public Health | Not related wellbeing |

| | | | | | |
|---|---|---|---|---|---|
| 2035 | da Silva, C. M. F. P.;de Assis, S. G.;Avanci, J. Q.; | 2024 | Cross-sectional study about suicide ideation and attempts among Brazilian pre-adolescents | BMC Psychiatry | Mental health disorders |
| 2036 | Choi, J.;Hall, C. B.;Clouston, S. A. P.;Cleven, K. L.;Mann, F. D.;Luft, B. J.;Zammit, A. R.; | 2024 | Cross-sectional association between posttraumatic stress and cognition is moderated by pulmonary functioning in World Trade Center responders. | American Journal of Industrial Medicine | Mental health disorders |
| 2037 | Agarwal, A. K.;Southwick, L.;Pelullo, A.;McCalpin, H. J.;Gonzales, R. E.;Asch, D. A.;Livesey, C.;Bellini, L.;Kishton, R.;Beck, S.;Merchant, R. M.; | 2024 | Cross-sectional analysis of healthcare worker mental health and utilization of a digital mental health platform from 2020 to 2023 | BMJ Open Quality | Not related wellbeing |
| 2038 | Braitman, A. L.;Stamates, A.;Colangelo, M.;Ehlke, S. J.;Ortman, J.;Heron, K. E.;Carey, K. B.; | 2023 | Criterion Validity of Protective Behavioral Strategies for Alcohol Consumption among College Students: comparison across Two Measures | Substance use & misuse | Mental health disorders |
| 2039 | Roblyer, M. Z. I.;Zambrano, S. B.; | 2020 | Crime Victimization and Suicidal Ideation Among Colombian College Students: The Role of Depressive Symptoms, Familism, and Social Support | Journal of Interpersonal Violence | Mental health disorders |
| 2040 | Zevallos, Ana L.;Washburn, Mara; | 2014 | Creating a Culture of Student Success: The SEEK Scholars Peer Mentoring Program | About Campus | Unpublished journal article |
| 2041 | Zhuo, L.;Wu, Q.;Le, H.;Li, H.;Zheng, L.;Ma, G.;Tao, H.; | 2021 | COVID-19-Related Intolerance of Uncertainty and Mental Health among Back-To-School Students in Wuhan: The Moderation Effect of Social Support | Int J Environ Res Public Health | Not related wellbeing |
| 2042 | Zeng, F.;John, W. C. M.;Sun, X.;Wang, Y.; | 2023 | COVID-19-associated impact and post-traumatic stress symptoms 39 days after pandemic in a sample of home-quarantined Chinese college | BMC Psychiatry | Mental health disorders |

| | | | | | |
|---|---|---|---|---|---|
| | | | students: the mediating effecting of past stressful events, psychological resilience, and social support | | |
| 2043 | Reid, C.;Beckstead, J.;Salinas-Miranda, A.; | 2022 | COVID-19 stress, social support, and coping in international students during the COVID-19 pandemic: a moderated analysis on anxiety and depression | J Am Coll Health | Mental health disorders |
| 2044 | Gallegos, M. I.;Zaring-Hinkle, B.;Bray, J. H.; | 2022 | COVID-19 pandemic stresses and relationships in college students | Fam Relat | Mental health disorders |
| 2045 | Muyor-Rodríguez, J.;Caravaca-Sánchez, F.;Fernández-Prados, J. S.; | 2021 | COVID-19 Fear, Resilience, Social Support, Anxiety, and Suicide among College Students in Spain | Int J Environ Res Public Health | Mental health disorders |
| 2046 | Tasso, A. F.;Hisli Sahin, N.;San Roman, G. J.; | 2021 | COVID-19 disruption on college students: Academic and socioemotional implications | Psychol Trauma | Not related wellbeing |
| 2047 | Berman, N. C.;Fang, A.;Hoeppner, S. S.;Reese, H.;Siev, J.;Timpano, K. R.;Wheaton, M. G.; | 2022 | COVID-19 and obsessive-compulsive symptoms in a large multi-site college sample | J Obsessive Compulsive Relat Disord | Not related wellbeing |
| 2048 | Cui, M.;Hong, P.; | 2021 | COVID-19 and Mental Health of Young Adult Children in China: Economic Impact, Family Dynamics, and Resilience | Fam Relat | The population is not college students |
| 2049 | Brown, M. L.;Trotter, C. E.;Huang, W.;Contreras Castro, K.;DeMuth, W. D.;Bing, E. G.; | 2023 | COVID-19 and mental health among college students in the southwestern United States | J Am Coll Health | Not related wellbeing |
| 2050 | David, I.;Kehinde, O.;Tefera, G. M.;Onyeaka, K.;Harvey, I. S.;Majee, W.; | 2023 | COVID-19 and Higher Education: A Qualitative Study on Academic Experiences of African International Students in the Midwest | Appl Res Qual Life | Not related wellbeing |

| | | | | | |
|---|---|---|---|---|---|
| 2051 | Farahmand, M.;Khalili, D.;Ramezani Tehrani, F.;Amin, G.;Negarandeh, R.; | 2020 | Could Anise decrease the intensity of premenstrual syndrome symptoms in comparison to placebo? A double-blind randomized clinical trial | Journal of complementary & integrative medicine | Mental health disorders |
| 2052 | Popp, J.;Nyman, J. A.;Luo, X.;Bengtson, J.;Lust, K.;An, L.;Ahluwalia, J. S.;Thomas, J. L.; | 2018 | Cost-effectiveness of enhancing a Quit-and-Win smoking cessation program for college students | European journal of health economics | Not related wellbeing |
| 2053 | Cowell, A. J.;Brown, J. M.;Mills, M. J.;Bender, R. H.;Wedehase, B. J.; | 2012 | Cost-effectiveness analysis of motivational interviewing with feedback to reduce drinking among a sample of college students | Journal of studies on alcohol and drugs | Not related wellbeing |
| 2054 | Lambic, D.; | 2016 | Correlation between Facebook use for educational purposes and academic performance of students | Computers in Human Behavior | Not related wellbeing |
| 2055 | Xiao, T.;Zhang, Y.; | 2017 | Correlation between athlete training intensity and cardiac performance | Nigerian journal of clinical practice | Not related wellbeing |
| 2056 | Smith, M. L.;Sosa, E. T.;Hochhalter, A. K.;Covin, J.;Ory, M. G.;McKyer, E. L.; | 2011 | Correlates of family health history discussions between college students and physicians: does family cancer history make a difference? | Journal of primary prevention | Not related wellbeing |
| 2057 | Romero, D. H.;Riggs, S. A.;Ruggero, C.; | 2015 | Coping, family social support, and psychological symptoms among student veterans | Journal of Counseling Psychology | Not related wellbeing |
| 2058 | Yeung, N. C.;Chow, T. S.; | 2019 | Coping with my way: Mediating roles of emotional expression and social support seeking in the associations between individual differences and posttraumatic growth | Health Psychol Open | Not related wellbeing |
| 2059 | Villegas-Gold, R.;Yoo, H. C.; | 2014 | Coping with discrimination among Mexican American college students | J Couns Psychol | Not related wellbeing |

| | | | | | |
|---|---|---|---|---|---|
| 2060 | Edirisinghe, N. P.;Makuloluwa, P. T. R.;Amarasekara, T.;Goonewardena, C. S. E.; | 2024 | Coping with cancer pain: a qualitative study to explore pain perception and self-coping strategies of patients with cancer in Sri Lanka | BMJ Open | Not related wellbeing |
| 2061 | Paralkar Ma, U.;Knutson Ph, D. D.; | 2023 | Coping with academic stress: Ambiguity and uncertainty tolerance in college students | J Am Coll Health | Mental health disorders |
| 2062 | Jenzer, T.;Read, J. P.;Naragon-Gainey, K.;Prince, M. A.; | 2019 | Coping trajectories in emerging adulthood: The influence of temperament and gender | J Pers | Not related wellbeing |
| 2063 | Rahat, Enes;Ilhan, Tahsin; | 2016 | Coping Styles, Social Support, Relational Self-Construal, and Resilience in Predicting Students' Adjustment to University Life | Educational Sciences: Theory and Practice | Not related wellbeing |
| 2064 | van Heemstra, H.;van der Aa, N.;Mooren, T.;Medema, D.;Vink, G.;Knipscheer, J.;Moradi, A.;Kleber, R.;Heide, J. J. T.; | 2024 | Coping styles in refugees with PTSD: Results from a randomized trial comparing EMDR therapy and stabilization | PLoS ONE | Mental health disorders |
| 2065 | Akbar, Z.;Aisyawati, M. S.; | 2021 | Coping Strategy, Social Support, and Psychological Distress Among University Students in Jakarta, Indonesia During the COVID-19 Pandemic | Front Psychol | Mental health disorders |
| 2066 | Zhang, N.; Henderson, C. N. R.; | 2022 | Coping strategies and chiropractic student perceived stress | J Chiropr Educ | Mental health disorders |
| 2067 | Marenco-Escuderos, A. D.;Cervantes, D. R.;Rambal-Rivaldo, L. I.; | 2023 | Coping profiles associated with resilience, academic engagement, and social support in university students | Interdisciplinary | Not related wellbeing |
| 2068 | Jenzer, T.;Cheesman, A. J.;Shaw, R. J.;Egerton, G. A.;Read, J. P.; | 2022 | Coping Flexibility and Alcohol-Related Outcomes: Examining Coping Motives as Mediators | Subst Use Misuse | Mental health disorders |

| | | | | | |
|---|---|---|---|---|---|
| 2069 | Reed, D. E.;Fischer, I. C.;Williams, R. M.;Na, P. J.;Pietrzak, R. H.; | 2024 | Co-occurring Chronic Pain and PTSD Among US Military Veterans: Prevalence, Correlates, and Functioning | Journal of General Internal Medicine | Mental health disorders |
| 2070 | Zhou, J. H.;Yu, H. Y.; | 2021 | Contribution of social support to home-quarantined Chinese college students' well-being during the COVID-19 pandemic: the mediating role of online learning self-efficacy and moderating role of anxiety | Social Psychology of Education | Mental health disorders |
| 2071 | Aronson, P.; | 2017 | Contradictions in the American Dream: High educational aspirations and perceptions of deteriorating institutional support | Int J Psychol | Not related wellbeing |
| 2072 | Leshem, B.;Kashy-Rosenbaum, G.;Schiff, M.;Benbenishty, R.;Pat-Horenczyk, R.; | 2023 | Continuous Exposure to Terrorism during the COVID-19 Pandemic: A Moderated Mediation Model in the Israeli Context | Int J Environ Res Public Health | Not related wellbeing |
| 2073 | Cassidy, R. N.;Jackson, K. M.;Rohsenow, D. J.;Tidey, J. W.;Tevyaw, T. O. L.;Barnett, N. P.;Monti, P. M.;Miller, M. E.;Colby, S. M.; | 2018 | Contingency management for college student smokers: the role of drinking as a moderator and mediator of smoking abstinence during treatment | Addictive behaviors | Not related wellbeing |
| 2074 | Buboltz, W.;Deemer, E.;Hoffmann, R.; | 2010 | Content analysis of the Journal of Counseling Psychology: Buboltz, Miller, and Williams (1999) 11 years later | J Couns Psychol | Published not from January 2010 to 31 March 2024 |
| 2075 | Ho, Hillbun;Ito, Kenichi; | 2019 | Consumption-oriented engagement in social network sites: Undesirable influence on personal well-being | European Journal of Marketing | Not related wellbeing |
| 2076 | Murray-Kolb, L. E.;Wenger, M. | 2017 | Consumption of Iron-Biofortified Beans Positively | Journal of Nutrition | Not related wellbeing |

| | | | | | |
|---|---|---|---|---|---|
| | J.;Scott, S. P.;Rhoten, S. E.;Lung'aho, M. G.;Haas, J. D.; | | Affects Cognitive Performance in 18- to 27-Year-Old Rwandan Female College Students in an 18-Week Randomized Controlled Efficacy Trial. | | |
| 2077 | White, M.;Legg, E.;Foroughi, B.;Rose, J.; | 2019 | Constructing past, present, and future communities: Exploring the experiences of community among last-dollar scholarship students | J Community Psychol | Not related wellbeing |
| 2078 | Wen, J.;Wu, Y.;Peng, L.;Chen, S.;Yuan, J.;Wang, W.;Cong, L.; | 2022 | Constructing and Verifying an Alexithymia Risk-Prediction Model for Older Adults with Chronic Diseases Living in Nursing Homes: A Cross-Sectional Study in China | Geriatrics (Basel) | The population is not college students |
| 2079 | Albright, J. N.;Hurd, N. M.; | 2018 | Constellations of social support among underrepresented college students: Associations with mental health | Applied Developmental Science | Not related wellbeing |
| 2080 | Smith, Kevin M.;Cobb, Kate F.;Reed-Fitzke, Kayla;Ferraro, Anthony J.;Duncan, James M.;Lucier-Greer, Mallory; | 2022 | Connections Between Parental Reciprocity and Emerging Adult Depressive Symptoms and Loneliness: The Role of Peer Social Support | Canadian Journal of Behavioural Science | Mental health disorders |
| 2081 | Mahoney, C. T.;Beck, B. M.;Dixon, K. E.;Horne, S. D.;Lawyer, S. R.; | 2024 | Conceptualizing impulsivity as a construct concerning posttraumatic stress disorder symptom severity among women | Journal of Traumatic Stress | Mental health disorders |
| 2082 | Dehnabaei, Z.;Tabibi, Z.;Ouimet, M. C.;Mohammadzadeh Moghaddam, A.;Ebrahimpour Delavar, M.; | 2024 | Computerized cognitive training to improve executive functions and driving skills of adolescents with and without symptoms of attention-deficit/hyperactivity disorder | Transportation Research Part F: Traffic Psychology and Behaviour | Mental health disorders |
| 2083 | Trinczer, I. L.;Shalev, L.; | 2024 | Computerized Attention Functions Training | Journal of Clinical | The population is not college |

| | | | | | |
|---|---|---|---|---|---|
| | | | Versus Computerised Executive Functions Training for Children with Attention Deficit/Hyperactivity Disorder: A Randomised Controlled Trial | Medicine | students |
| 2084 | Fernandez-Pineda, M.;Swift, A.;Dolbier, C.;Banasiewicz, K. G.; | 2024 | Compounding stress: A mixed-methods study on the psychological experience of miscarriage amid the COVID-19 pandemic | BMC Pregnancy and Childbirth | Mental health disorders |
| 2085 | Eshel, Y.;Kimhi, S.;Marciano, H.;Adini, B.; | 2021 | Components of Unrealistic Optimism of College Students: The Case of the COVID-19 Pandemic | Front Psychol | Not related wellbeing |
| 2086 | Yang, Z.;Chen, X.;Chen, X.;Xie, Z.;Luo, D.; | 2024 | Completed suicide risk factors among people living with HIV in Hunan Province identified through a psychological autopsy case-control study | Scientific Reports | Mental health disorders |
| 2087 | Maykish, A.;Nishisaka, M. M.;Talbott, C. K.;Reaves, S. K.;Kristo, A. S.;Sikalidis, A. K.; | 2021 | Comparison of Whey Versus Almond Protein Powder on Nitrogen Balance in Female College Students; The California Almond Protein Powder Project (CAlmond-P3) | International journal of environmental research and public health | Not related wellbeing |
| 2088 | Kasahara, K.;Konrad, A.;Yoshida, R.;Murakami, Y.;Koizumi, R.;Sato, S.;Ye, X.;Thomas, E.;Nakamura, M.; | 2022 | Comparison of the Prolonged Effects of Foam Rolling and Vibration Foam Rolling Interventions on Passive Properties of Knee Extensors | Journal of sports science & medicine | Mental health disorders |
| 2089 | Ward, J.;Coats, J.; | 2017 | Comparison of the BackJoy SitSmart Relief and Spine Buddy LT1 H/C Ergonomic Chair Supports on Short-Term Neck and Back Pain | Journal of manipulative and physiological therapeutics | Not related wellbeing |
| 2090 | Lozano, B. E.;Stephens, R. S.; | 2010 | Comparison of participative set and assigned goals in the reduction of alcohol use | Psychology of addictive behaviors | Published not from January 2010 to 31 March 2024 |

| | | | | | |
|---|---|---|---|---|---|
| 2091 | Goldman, D. B.;Wade, N. G.; | 2012 | Comparison of forgiveness and anger-reduction group treatments: a randomized controlled trial | Psychotherapy research | Not related wellbeing |
| 2092 | Muraosa, H.;Shirata, T.;Saito, Y.;Noto, K.;Suzuki, A.; | 2024 | Comparison of dysfunctional attitudes, cognitive vulnerability to depression, before and during the COVID-19 pandemic in healthy participants | BMC Psychology | Mental health disorders |
| 2093 | Vanderslice-Barr, J. L.;Miele, A. S.;Jardin, B.;McCaffrey, R. J.; | 2011 | Comparison of computerized versus booklet versions of the TOMM™ | Applied neuropsychology | Not related wellbeing |
| 2094 | Lim, Y. B.;Song, H.;Lee, H.;Lim, S.;Kwon, S. Y.;Chun, J.;Kim, S.;Tosun, C.;Yoon, K. S.;Sohn, C. H.;Kim, B. N.; | 2024 | Comparison of arterial spin-labeled MRI (ASL MRI) between ADHD and control group (ages of 6–12) | Scientific Reports | Mental health disorders |
| 2095 | Douris, P. C.;McDonald, B.;Vespi, F.;Kelley, N. C.;Herman, L.; | 2012 | Comparison between Nintendo Wii Fit aerobics and traditional aerobic exercise in sedentary young adults | Journal of strength and conditioning research | The population is not college students |
| 2096 | Nagilla, J.;Kulkarni, S.;Madupu, P. R.;Doshi, D.;Bandari, S. R.;Srilatha, A.; | 2017 | Comparative evaluation of antiplaque efficacy of coconut oil pulling and a placebo, among dental college students: a randomized controlled trial | Journal of clinical and diagnostic research | Not related wellbeing |
| 2097 | Glass, C. R.;Westmont, C. M.; | 2014 | Comparative Effects of belongingness on the academic success and cross-cultural interactions of domestic and international students | International Journal of Intercultural Relations | Not related wellbeing |
| 2098 | Jaafarpour, M.;Hatefi, M.;Khani, A.;Khajavikhan, J.; | 2015 | Comparative effect of cinnamon and ibuprofen for treatment of primary dysmenorrhea: a randomized double-blind clinical trial | Journal of clinical and diagnostic research | Mental health disorders |
| 2099 | Ragsdale, K. A.;Nichols, A. A.;Mehta, M.;Maples-Keller, J. | 2024 | Comorbid treatment of traumatic brain injury and mental health disorders | NeuroRehabilitation | Mental health disorders |

| | | | | | |
|---|---|---|---|---|---|
| | L.;Yasinski, C. W.;Hyatt, C. S.;Watkins, L. E.;Loucks, L. A.;Carbone, E.;Rauch, S. A. M.;Rothbaum, B. O.; | | | | |
| 2100 | Lin, P. C.;Long, C. Y.;Ko, C. H.;Yen, J. Y.; | 2024 | Comorbid Attention Deficit Hyperactivity Disorder in Women with Premenstrual Dysphoric Disorder | Journal of Women's Health | Mental health disorders |
| 2101 | Yuan, L.;Lu, L.;Wang, X.;Qu, M.;Gao, Y.;Pan, B.; | 2023 | Comorbid anxiety and depressive symptoms and the related factors among international medical students in China during COVID-19 pandemic: a cross-sectional study | BMC Psychiatry | Mental health disorders |
| 2102 | Prokes, C.;Housel, J.; | 2021 | Community College Student Perceptions of Remote Learning Shifts Due to COVID-19 | TechTrends | Not related wellbeing |
| 2103 | Wright, Kevin B.;Rosenberg, Jenny;Egbert, Nicole;Ploeger, Nicole A.;Bernard, Daniel R.;King, Shawn; | 2013 | Communication competence, social support, and depression among college students: a model of Facebook and face-to-face support network influence | Journal of Health Communication | Mental health disorders |
| 2104 | Lorch, R. F.;Chen, H. T.;Lemarié, J.; | 2012 | Communicating headings and preview sentences in text and speech | Journal of experimental psychology. Applied | Not related wellbeing |
| 2105 | Fogaca, J. L.; | 2021 | Combining Mental Health and Performance Interventions: Coping and Social Support for Student-Athletes | Journal of Applied Sport Psychology | Mental health disorders |
| 2106 | Berdin, A. N.;Saules, K. K.; | 2019 | Combined Use of Alcohol and the Internet: associated Features | Substance use & misuse | Mental health disorders |

| | | | | | |
|---|---|---|---|---|---|
| 2107 | Thirumagal, P. G.;Madhumita, G.;Krishna Priya, V.; | 2019 | Collision of Social Network Sites among college student's academic performance | International Journal of Recent Technology and Engineering | Not related wellbeing |
| 2108 | Levine, R.;Manley, K.;Bailey, G.;Warnecke, A.;Davis, D.;Sommers, A.; | 2021 | College Success Among Students From Disadvantaged Backgrounds: “Poor” and “Rural” Do Not Spell Failure | Journal of College Student Retention: Research, Theory and Practice | Not related wellbeing |
| 2109 | LeGary, R. A.; | 2017 | College Students with Autism Spectrum Disorder: Perceptions of Social Supports that Buffer College-Related Stress and Facilitate Academic Success | Journal of Postsecondary Education and Disability | Mental health disorders |
| 2110 | Ramsey, M. A.;Gentzler, A. L.;Morey, J. N.;Oberhauser, A. M.;Westerman, D.; | 2013 | College students use of communication technology with parents: comparisons between two cohorts in 2009 and 2011 | Cyberpsychol Behav Soc Netw | Not related wellbeing |
| 2111 | Ye, Shaoyu; Ho, Kevin K. W.; | 2023 | College students' Twitter usage and psychological well-being from the perspective of generalized trust: comparing changes before and during the COVID-19 pandemic | Library Hi Tech | Unpublished journal article |
| 2112 | Corbitt-Hall, D. J.;Gauthier, J. M.;Davis, M. T.;Witte, T. K.; | 2016 | College Students' Responses to Suicidal Content on Social Networking Sites: an Examination Using a Simulated Facebook Newsfeed | Suicide & life-threatening behavior | Mental health disorders |
| 2113 | Dziewior, J.;Carr, L. J.;Pierce, G. L.;Whitaker, K.; | 2022 | College students report less physical activity and more sedentary behavior during the COVID-19 pandemic. | J Am Coll Health | Not related wellbeing |
| 2114 | Klonoff-Cohen, H.; | 2022 | College Students' Opinions About Coping | Front Psychol | Mental health disorders |

| | | | | | |
|---|---|---|---|---|---|
| | | | Strategies for Mental Health Problems, Suicide Ideation, and Self-Harm During COVID-19 | | |
| 2115 | Yakunina, E. S.;Rogers, J. R.;Waehler, C. A.;Werth, J. L., Jr.; | 2010 | College students' intentions to seek help for suicidal ideation: accounting for the help-negation effect | Suicide Life Threat Behav | Published not from January 2010 to 31 March 2024 |
| 2116 | Lewine, Rich;Warnecke, Ashlee;Sommers, Alison; | 2022 | College Students from Poverty: Academic Success and Authenticity | Journal of Poverty | Not related wellbeing |
| 2117 | Huang, X.;Deng, Y.;Ge, P.;Sun, X.;Huang, M.;Chen, H.;Wang, Y.;Suo, B.;Song, Z.;Wu, Y.; | 2022 | College Students' Degree of Support for Online Learning during the COVID-19 Pandemic and Associated Factors: A Cross-Sectional Study | Int J Environ Res Public Health | Not related wellbeing |
| 2118 | Shermeyer, L.;Morrow, M. T.;Mediate, N.; | 2019 | College students' daily coping, mood, and quality of life: Benefits of problem-focused engagement | Stress Health | Unpublished journal article |
| 2119 | Slawson, D. L.;Dalton, W. T., 3rd;Dula, T. M.;Southerland, J.;Wang, L.;Littleton, M. A.;Mozen, D.;Relyea, G.;Schetzina, K.;Lowe, E. F.;Stoots, J. M.;Wu, T.; | 2015 | College students as facilitators in reducing adolescent obesity disparity in Southern Appalachia: Team Up for Healthy Living | Contemp Clin Trials | Not related wellbeing |
| 2120 | Guan, J. M.;Xiang, P.;Keating, X.;Land, W.; | 2020 | College Students' Achievement Goals, Social Goals, and Self-reported Persistence in Physical Activity Class Settings | Quest | Unpublished journal article |
| 2121 | Alt, D.; | 2015 | College students' academic motivation, media engagement, and fear of missing out | Computers in Human Behavior | Not related wellbeing |
| 2122 | Perrault, E. K.;McCullock, S. P.;Lee, D. G.;Hildenbrand, G. M.;Mikkelsen, D. G.; | 2023 | College student gratitude: A silver lining while evaluating a yearlong bathroom stall messaging campaign | J Am Coll Health | Not related wellbeing |

| | | | | | |
|---|---|---|---|---|---|
| 2123 | Strauser, C. M.;Chavez, V.;Lindsay, K. R.;Figgins, M. M.;DeShaw, K. J.; | 2023 | College student-athlete versus nonathlete mental and social health factors during the COVID-19 pandemic | J Am Coll Health | Not related wellbeing |
| 2124 | Apker, J.; | 2022 | College student accounts of coping and social support during COVID-19 impacted learning | Communication Quarterly | Not related wellbeing |
| 2125 | Hooker, S.;Brand, B.; | 2010 | College knowledge: a critical component of college and career readiness | New Dir Youth Dev | Published not from January 2010 to 31 March 2024 |
| 2126 | Chrisman, M. S.;Wright, R.;Purdy, W.; | 2021 | College Classroom Instructors Can Effectively Promote Standing among Students Provided with Standing Desks | International journal of environmental research and public health | Not related wellbeing |
| 2127 | Accardo, A. L.;Bean, K.;Cook, B.;Gillies, A.;Edgington, R.;Kuder, S. J.;Bomgardner, E. M.; | 2019 | College Access, Success, and Equity for Students on the Autism Spectrum | Journal of Autism and Developmental Disorders | Not related wellbeing |
| 2128 | Salimova, L.;Rusnáková, M.; | 2024 | Collaborative strategies for adolescent suicide prevention: insights from Slovakia and Kyrgyzstan | Discover Mental Health | Mental health disorders |
| 2129 | Murphy, P.;Garavan, H.; | 2011 | Cognitive predictors of problem drinking and AUDIT scores among college students | Drug and alcohol dependence | Not related wellbeing |
| 2130 | Buckenmeyer, P. J.;Bauer, J. A.;Hokanson, J. F.;Hendrick, J. L.; | 2015 | Cognitive influence of a 5-h ENERGY® shot: are effects perceived or real? | Physiology & behavior | Not related wellbeing |
| 2131 | Kanevski, M.;Booth, J. N.;Stewart, T. M.;Rhodes, S. M.; | 2024 | Cognitive heterogeneity in Attention Deficit Hyperactivity Disorder: Implications for maths | British Journal of Developmental Psychology | Mental health disorders |
| 2132 | Conway, L. G.;Harris, K. J.;Catley, D.;Gornick, L. J.;Conway, K. | 2017 | Cognitive complexity of clients and counselors during motivation-based treatment for smoking | BMJ Open | Not related wellbeing |

| | | | | | |
|---|---|---|---|---|---|
| | R.;Repke, M. A.;Houck, S. C.; | | cessation: an observational study on occasional smokers in a US college sample | | |
| 2133 | Stephens, N. M.;Hamedani, M. G.;Destin, M.; | 2014 | Closing the social-class Achievement gap: a difference-education Intervention Improves first-generation Students' Academic Performance and All Students' College Transition | Psychological science | Mental health disorders |
| 2134 | Parnes, McKenna F.;Kanchewa, Stella S.;Marks, Amy K.;Sarah, E. O. Schwartz; | 2020 | Closing the college achievement gap: Impacts and processes of a help-seeking intervention | Journal of Applied Developmental Psychology | Mental health disorders |
| 2135 | Harackiewicz, J. M.;Canning, E. A.;Tibbetts, Y.;Priniski, S. J.;Hyde, J. S.; | 2016 | Closing Achievement Gaps with a utility-value Intervention: Disentangling Race and social class | Journal of personality and social psychology | Mental health disorders |
| 2136 | Gilbertson, M. K.;Brady, S. T.;Ablorh, T.;Logel, C.;Schnitker, S. A.; | 2022 | Closeness to God, Spiritual Struggles, and Wellbeing in the First Year of College | Frontiers in Psychology | Not related to research questions |
| 2137 | Wisk, L. E.;Nelson, E. B.;Magane, K. M.;Weitzman, E. R.; | 2019 | Clinical Trial Recruitment and Retention of College Students with Type 1 Diabetes via Social Media: an Implementation Case Study | Journal of diabetes science and technology | Mental health disorders |
| 2138 | Ruud, T.;Selle, M. L.;Clausen, H. K.;Heiervang, K. S.;Odden, S.;Stuen, H. K.;Landheim, A.; | 2024 | Clinical outcomes and outcome predictors of two-year assertive community treatment in Norway: an explorative prospective pre-post study | BMC Psychiatry | Mental health disorders |
| 2139 | Langberg, J. M.;Dvorsky, M. R.;Silvia, P.;Labban, J.;Anastopoulos, A. D.; | 2023 | Clinical Change Mechanisms in the Treatment of College Students With ADHD: trajectories and Associations With Outcomes | Behavior therapy | Mental health disorders |
| 2140 | Lopes, J.; | 2024 | Clinical and demographic factors influencing suicide, hospitalizations, and work disability in a | Minerva Psychiatry | Mental health disorders |

| | | | | | |
|---|---|---|---|---|---|
| | | | group of bipolar II Portuguese women | | |
| 2141 | Merchant, Gina;Fowler, James H.;Greg, J. Norman;Anjali, Gupta;Christina, Servetas;Karen, Calfas;Ketaki, Raste;Laura, Pina;Mike, Donohue;William, G. Griswold;Simon, Marshall; | 2014 | Click “Like” to Change Your Behavior: A Mixed Methods Study of College Students’ Exposure to and Engagement With Facebook Content Designed for Weight Loss | Journal of medical Internet research | Not related wellbeing |
| 2142 | Merchant, G.;Weibel, N.;Patrick, K.;Fowler, J. H.;Norman, G. J.;Gupta, A.;Servetas, C.;Calfas, K.;Raste, K.;Pina, L.;et al.,; | 2014 | Click "like" to change your behavior: a mixed methods study of college students' exposure to and engagement with Facebook content designed for weight loss. | Journal of medical Internet research | Not related wellbeing |
| 2143 | Zhong, Z. J.; | 2014 | Civic engagement among educated Chinese youth: The role of SNS (Social Networking Services), bonding and bridging social capital | Computers and Education | Not related wellbeing |
| 2144 | Berg, C. J.;Haardörfer, R.;Vu, M.;Getachew, B.;Lloyd, S. A.;Lanier, A.;Childs, D.;Sandridge, Y.;Bierhoff, J.;Li, J.;Dossantos, E.;Windle, M.; | 2018 | Cigarette use trajectories in young adults: Analyses of predictors across system levels | Drug Alcohol Depend | The population is not college students |
| 2145 | Wills, C.;Ghani, S.;Tubbs, A.;Fernandez, F. X.;Athey, A.;Turner, R.;Robbins, R.;Patterson, F.;Warlick, C.;Alfonso-Miller, P.;Killgore, W. D. S.;Grandner, M. A.; | 2021 | Chronotype and social support among student-athletes: impact on depressive symptoms | Chronobiology International | Mental health disorders |

| | | | | | |
|---|---|---|---|---|---|
| 2146 | Vidal, C.;Reinert, M.;Nguyen, T.;Jun, H. J.; | 2024 | Chronic stress and lack of social support: Role in adolescent depression and suicide-related behaviors in the context of the COVID-19 pandemic | Journal of Affective Disorders | Mental health disorders |
| 2147 | Gage, M.;Phillips, K.;Noh, B.;Yoon, T.; | 2021 | Choline-Based Multi-Ingredient Supplementation Can Improve Explosive Strength during a Fatiguing Task | International journal of environmental research and public health | Not related wellbeing |
| 2148 | Interian, A.;Myers, C. E.;Brenner, L. A.;Sweeney, R.;Osterberg, T.;Reddy, V.;Barnhart, M.;Hill, L. S.;Miller, R. B.;Beck, K. D.;Cominski, T. P.;Chan, C. C.;Shafritz, K. M.;Goodman, M. S.;Hazlett, E. A.; | 2024 | Choice and motor impulsivity in Veterans with mild traumatic brain injury with and without a history of suicide attempt | Psychiatry Research | Mental health disorders |
| 2149 | Tan, Fei;Luo, Bingquan;Lin, Shaozhu;Guo, Kelei; | 2023 | Chinese college students' physical activity motivation and social development: Chain mediation effect of perceived social support and subjective well-being | Social Behavior and Personality | Not related wellbeing |
| 2150 | Wang, Z.;Hua, L.;Huang, Y.;Deng, X.;Zhao, Y.;Xiao, Y.;Li, J.; | 2023 | Chinese calligraphic handwriting practice promotes positive affect in adolescents: converging evidence from correlational and experimental designs. | Acta Psychologica | Not related wellbeing |
| 2151 | Hall, J.; | 2010 | Childhood perceptions of family, social support, parental alcoholism, and later alcohol use among African American college students | Journal of Substance Use | Published not from January 2010 to 31 March 2024 |

| | | | | | |
|---|---|---|---|---|---|
| 2152 | Zhao, J. X.;Peng, X.;Chao, X. M.;Xiang, Y. H.; | 2019 | Childhood Maltreatment Influences Mental Symptoms: The Mediating Roles of Emotional Intelligence and Social Support | Frontiers in Psychiatry | The population is not college students |
| 2153 | Zeng, Y. W.;Chiu, S. H.;Yeh, C. B.; | 2024 | Childhood Maltreatment Associated with Anxiety Depression and Complex PTSD Symptoms | Psychiatry and Clinical Psychopharmacology | Mental health disorders |
| 2154 | Li, C.;Wang, R.;Zhu, N.;Kong, F.; | 2024 | Childhood maltreatment and depressed mood in female college students: A daily diary analysis | Child Abuse Negl | The population is not college students |
| 2155 | You, Z.;Chen, M.;Yang, S.;Zhou, Z.;Qin, P.; | 2014 | Childhood adversity, recent life stressors and suicidal behavior in Chinese college students | PLoS ONE | The population is not college students |
| 2156 | Lee, J. K.;Lee, J.;Chung, M. K.;Shin, T.;Park, J. Y.;Lee, K. J.;Lim, H. S.;Hwang, S.;Urtnasan, E.;Jo, Y.;Kim, M. H.; | 2024 | Childhood adversity and suicidal ideation in older Korean Adults: unraveling the mediating mechanisms of mental Health, physical health, and social relationships | BMC Psychiatry | The population is not college students |
| 2157 | Yates, T. M.;Gregor, M. A.;Haviland, M. G.; | 2012 | Child maltreatment, alexithymia, and problematic internet use in young adulthood | Cyberpsychol Behav Soc Netw | The population is not college students |
| 2158 | Kim, L.;Huh, D. A.;Kang, M. S.;Park, K.;Lee, J.;Hwang, S. H.;Choi, H. J.;Lim, W.;Moon, K. W.;Lee, Y. J.; | 2024 | Chemical exposure from the Hebei Spirit oil spill accident and its long-term effects on mental health | Ecotoxicology and Environmental Safety | Not related wellbeing |
| 2159 | Colditz, Jason B.;Chu, Kar-Hai;Hsiao, Lily;Barrett, Erica;Kraemer, Kevin L.;Pedersen, Sarah L.; | 2023 | Characterizing online social support for alcohol use disorder: A mixed-methods approach | Alcohol, Clinical and Experimental Research | Mental health disorders |
| 2160 | Du, X.;Zhang, Q.;Jiang, Y.;Zhu, | 2022 | Characterization of plasma-derived exosomal | Environment | Not related wellbeing |

| | | | | | |
|---|---|---|---|---|---|
| | X.;Zhang, Y.;Liu, C.;Niu, Y.;Cai, J.;Chen, R.;Kan, H.; | | miRNA changes following traffic-related air pollution exposure: a randomized, crossover trial based on small RNA sequencing | International | |
| 2161 | Sun, Y.;Zhou, J.;Zhu, H.;Liu, P.;Lin, H.;Xiao, Z.;Yu, X.;Qian, J.;Tong, M.;Chi, X.;Hong, Q.; | 2024 | Characteristics of Speech Auditory Brainstem Response in Preschool Children With Attention-Deficit/Hyperactivity Disorder | Journal of Speech, Language, and Hearing Research | The population is not college students |
| 2162 | Li, D. J.;Huang, J. J.;Hsu, S. T.;Hsieh, K. Y.;Lin, G. G.;Wu, P. J.;Liu, C. L.;Wu, H. C.;Chou, F. H. C.; | 2024 | Characteristics of Sleep Disturbance and Comparison Across Three Waves of the COVID-19 Pandemic Among Healthcare Workers | Psychiatry Investigation | Mental health disorders |
| 2163 | Warburton, K. M.;Yost, J. S.;Bajo, S. D.;Martindale, J. R.;Parsons, A. S.;Ryan, M. S.; | 2024 | Characteristics of ADHD in Struggling Residents and Fellows | Journal of Graduate Medical Education | Mental health disorders |
| 2164 | Fagioli, L.;Rios-Aguilar, C.;Deil-Amen, R.; | 2015 | Changing the context of student engagement: Using Facebook to increase community college student persistence and success. | Teachers College Record | Not related wellbeing |
| 2165 | Huang, S.;Wang, D.;Zhao, J.;Chen, H.;Ma, Z.;Pan, Y.;Liu, X.;Fan, F.; | 2022 | Changes in suicidal ideation and related influential factors in college students during the COVID-19 lockdown in China | Psychiatry Res | Mental health disorders |
| 2166 | Mirabito, G.;Verhaeghen, P.; | 2023 | Changes in State Mindfulness are the Key to Success in Mindfulness Interventions: ecological Momentary Assessments of Predictors, Mediators, and Outcomes in a Four-Week Koru Mindfulness Intervention. | Psychological reports | Mental health disorders |
| 2167 | Rowland, D. L.;Kamran Ehsan, M.;Cooper, S. E.; | 2024 | Changes in Spousal Intimacy in Women Suffering Trauma Symptoms from Domestic Abuse: A | International journal of | Mental health disorders |

| | | | | | |
|---|---|---|---|---|---|
| | | | Culturally Embedded Intervention Study in Pakistan | environmental research and public health | |
| 2168 | Kaj, M.;Tékus, É;Juhász, I.;Stomp, K.;Wilhelm, M.; | 2015 | Changes in physical fitness of Hungarian college students in the last fifteen years | Acta biologica Hungarica | Not related wellbeing |
| 2169 | Li, K.;Haynie, D.;Lipsky, L.;Iannotti, R. J.;Pratt, C.;Simons-Morton, B.; | 2016 | Changes in Moderate-to-Vigorous Physical Activity Among Older Adolescents | Pediatrics | Not related wellbeing |
| 2170 | Caldwell, K.;Emery, L.;Harrison, M.;Greeson, J.; | 2011 | Changes in mindfulness, well-being, and sleep quality in college students through taijiquan courses: a cohort control study | Journal of alternative and complementary medicine (New York, N.Y.) | Mental health disorders |
| 2171 | Dennhardt, A. A.;Yurasek, A. M.;Murphy, J. G.; | 2015 | Change in delay discounting and substance reward value following a brief alcohol and drug use intervention | Journal of the experimental analysis of behavior | Mental health disorders |
| 2172 | Mohn, C.;Haga, E.;Nilsson, H. S. W.;Pirkis, J.;Mehlum, L.; | 2024 | Change in attitudes after a suicide prevention media campaign in the Mid-Norway region. | BMC Psychiatry | Mental health disorders |
| 2173 | Looby, A.;De Young, K. P.;Earleywine, M.; | 2013 | Challenging expectancies to prevent nonmedical prescription stimulant use: a randomized, controlled trial | Drug and alcohol dependence | Not related wellbeing |
| 2174 | Maddah, Z.;Negarandeh, R.;Rahimi, S.;Pashaeypoor, S.; | 2024 | Challenges of living with veterans with post-traumatic stress disorder from the perspective of spouses: A Qualitative Content Analysis Study | BMC Psychiatry | Mental health disorders |
| 2175 | Roberti, E.;Clavenna, A.;Basso, E.;Bravaccio, C.;Riccio, M. P.;Pincherle, M.;Duca, M.;Giordani, | 2024 | Challenges in transitioning from adolescent to Adult Mental Health Services for young adults with ADHD in Italy: an observational study | Epidemiology and Psychiatric Sciences | Mental health disorders |

| | | | | | |
|---|---|---|---|---|---|
| | C.;Scarpellini, F.;Campi, R.;Giardino, M.;Zanetti, M.;Tessarollo, V.;Costantino, I.;Bonati, M.;Cartabia, M.;Calati, M. G.;Graziani, V.;Marchetti, F.;Suprani, T. | | | | |
| 2176 | Mesfin, W.;Habtamu, K.; | 2024 | Challenges and coping mechanisms of parents of children with attention deficit hyperactivity disorder in Addis Ababa, Ethiopia: a qualitative study | BMC Psychology | The population is not college students |
| 2177 | Blithikioti, C.;Duek, O.;Gordon, C.;Krystal, J. H.;Levy, I.;Harpaz-Rotem, I.;Schiller, D.;Perl, O.; | 2024 | Cerebellar Contributions to Traumatic Autobiographical Memory in People with Post-Traumatic Stress Disorder | Cerebellum | Mental health disorders |
| 2178 | Biehl, S. A.;Kahn, J. H.; | 2016 | Causal Effects of Language on the Exchange of Social Support in an Online Community | Cyberpsychol Behav Soc Netw | Not related wellbeing |
| 2179 | Winderman, Kate;Martin, Colleen E.;Smith, Nathan Grant; | 2018 | Career Indecision among LGB College Students: The Role of Minority Stress, Perceived Social Support, and Community Affiliation | Journal of Career Development | Mental health disorders |
| 2180 | Lent, R. W.;do Céu Taveira, M.;Soares, J.;Marques, C.;Cardoso, B.;Oliveira, Í; | 2022 | Career decision-making in unemployed Portuguese adults: Test of the social cognitive model of career self-management | J Couns Psychol | The population is not college students |
| 2181 | Jeong, C. Y.;Hong, A. J.; | 2023 | Career adaptability and mediated social network process linking achievement goal orientation to behavior | Journal of Employment Counseling | Not related wellbeing |
| 2182 | Stergiopoulos, V.;Bastidas-Bilbao, | 2024 | Care considerations in medical assistance in dying | BMC Psychiatry | Not related wellbeing |

| | | | | | |
|---|---|---|---|---|---|
| | H.;Gupta, M.;Buchman, D. Z.;Stewart, D. E.;Rajji, T.;Simpson, A. I. F.;van Kesteren, M. R.;Cappe, V.;Castle, D.;Shields, R.;Hawke, L. D.; | | for persons with mental illness as the sole underlying medical condition: a qualitative study of patient and family perspectives | | |
| 2183 | Rydell, R. J.;Boucher, K. L.; | 2010 | Capitalizing on multiple social identities to prevent stereotype threat: the moderating role of self-esteem | Personality & Social Psychology Bulletin | Published not from January 2010 to 31 March 2024 |
| 2184 | Xie, G.;Gao, X.;Guo, Q.;Liang, H.;Yao, L.;Li, W.;Ma, B.;Wu, N.;Han, X.;Li, J.; | 2024 | Cannabidiol ameliorates PTSD-like symptoms by inhibiting neuroinflammation through its action on CB2 receptors in the brains of male mice. | Brain, Behavior, and Immunity | Mental health disorders |
| 2185 | Kivlen, C.;Winston, K.;Mills, D.;DiZazzo-Miller, R.;Davenport, R.;Binfet, J. T.; | 2022 | Canine-Assisted Intervention Effects on the Well-Being of Health Science Graduate Students: a Randomized Controlled Trial | The American Journal of Occupational Therapy: official publication of the American Occupational Therapy Association | Mental health disorders |
| 2186 | Frith, E.;Loprinzi, P.; | 2017 | Can Facebook Reduce Perceived Anxiety Among College Students? Randomized Controlled Exercise Trial Using the Transtheoretical Model of Behavior Change | JMIR mental health | Mental health disorders |
| 2187 | Faro, E. Z.;Jones, D.;Adeagbo, M.;Cho, H.;Swartzendruber, | 2024 | Can an evidence-based mental health intervention be implemented into preexisting home visiting | Implementation Science | Mental health disorders |

| | | | | | |
|---|---|---|---|---|---|
| | G.;Tabb, K. M.;Tandon, S. D.;Ryckman, K.; | | programs using implementation facilitation? Study protocol for a three-variable implementation effectiveness context hybrid trial | | |
| 2188 | Forster, M.;Rogers, C. J.;Sussman, S.;Watts, J.;Rahman, T.;Yu, S.;Benjamin, S. M.; | 2021 | Can Adverse Childhood Experiences Heighten Risk for Problematic Internet and Smartphone Use? Findings from a College Sample | Int J Environ Res Public Health | The population is not college students |
| 2189 | Thomas, S.; | 2024 | Call for Manuscripts-Special Issue on Caregiver Suicide and Promotion of Well-Being | Issues in mental health nursing | Mental health disorders |
| 2190 | Hernandez-Tejada, M. A.;Little, D. M.;Bruce, M. J.;Butte, S.;Burnett, J.;Wood, L.;Acierno, R.; | 2024 | Building resilience: A specialty clinic tailored to older adults at risk for violence and abuse | International Journal of Psychiatry in Medicine | The population is not college students |
| 2191 | Weisz, E.;Ong, D. C.;Carlson, R. W.;Zaki, J.; | 2021 | Building empathy through motivation-based interventions | Emotion (Washington, D.C.) | Mental health disorders |
| 2192 | Finitsis, D. J.;Cruess, D. G.;Pinnamaraju, S.;Robinson, P.;Woodruff, T. A.; | 2022 | Brief, peer-delivered motivational interview promotes help-seeking behavior among college students with needle anxiety: a randomized controlled trial. | Journal of American College Health | Mental health disorders |
| 2193 | Vázquez, F. L.;Torres, Á J.;Blanco, V.;Bouza, Q.;Otero, P.;Andrade, E.;Simón, M. Á;Bueno, A. M.;Arrojo, M.;Páramo, M.;Fernández, A.; | 2024 | Brief psychological intervention for suicide prevention based on problem-solving applied in different formats to people over 50 years old: protocol for a randomized controlled trial | BMC Psychiatry | Mental health disorders |
| 2194 | Martin-Perez, C.;Navas, J. F.;Perales, J. C.;Lopez-Martin, A.;Cordovilla-Guardia, S.;Portillo, | 2019 | Brief group-delivered motivational interviewing is equally effective as brief group-delivered cognitive-behavioral therapy at reducing alcohol | PLoS ONE | Mental health disorders |

| | | | | | |
|---|---|---|---|---|---|
| | M.;Maldonado, A.;Vilar-Lopez, R.; | | use in risky college drinkers. | | |
| 2195 | Church, D.;De Asis, M. A.;Brooks, A. J.; | 2012 | Brief group intervention using emotional freedom techniques for depression in college students: a randomized controlled trial | Depression research and treatment | Mental health disorders |
| 2196 | Ramirez, J. J.;Monti, P. M.;Colwill, R. M.; | 2015 | Brief and extended alcohol-cue-exposure effects on craving and attentional bias | Experimental and clinical psychopharmacology | Mental health disorders |
| 2197 | Lekkas, D.;Jacobson, N. C.; | 2024 | Breaking the silence: leveraging social interaction data to identify high-risk suicide users online using network analysis and machine learning. | Scientific Reports | Mental health disorders |
| 2198 | Tahmin, C. I.;Tahsin, C. T.;Wattero, R.;Ahmed, Z.;Corbin, C.;Carter, J. R.;Park, J.;Racette, S. B.;Sullivan, S. S.;Herr, M. D.;Fonkoue, I. T.; | 2024 | Blunted brachial blood flow velocity response to acute mental stress in PTSD females | Physiological Reports | Mental health disorders |
| 2199 | Wani, A. H.;Katrinli, S.;Zhao, X.;Daskalakis, N. P.;Zannas, A. S.;Aiello, A. E.;Baker, D. G.;Boks, M. P.;Brick, L. A.;Chen, C. Y.;Dalvie, S.;Fortier, C.;Geuze, E.;Hayes, J. P.;Kessler, R. C.;King, A. P.;Koen, N.;Liberzon, I.;Lori, A.;Luykx, J. J.;Maihofer, | 2024 | Blood-based DNA methylation and exposure risk scores predict PTSD with high accuracy in military and civilian cohorts. | BMC Medical Genomics | Mental health disorders |
| 2200 | Short, N. A.;Allan, N. P.;Ashrafioun, L.;Stecker, T.; | 2024 | Beliefs about mental health treatment, treatment initiation, and suicidal behaviors among veterans and service members at risk for suicide and not in | Suicide and Life-Threatening Behavior | Mental health disorders |

| | | | | | |
|---|---|---|---|---|---|
| | | | treatment | | |
| 2201 | Thieux, M.;Lioret, J.;Bouet, R.;Guyon, A.;Lachaux, J. P.;Herbillon, V.;Franco, P.; | 2024 | Behavioral and Electrophysiological Markers of Attention Fluctuations in Children with Hypersomnolence | Journal of Clinical Medicine | The population is not college students |
| 2202 | Ilagan, Guy;Ilagan, Jill;Jocius, Robin;Hornor, Tara;Shealy, Todd;Simpson, Annie;Cavaliere, Greg;Pollen, Brett;Brooks, Jesse; | 2022 | Backpacking Veterans: Exploring Sense of Belonging, Happiness, and Stress-Coping | Journal of Outdoor Recreation, Education, and Leadership | Mental health disorders |
| 2203 | Knouse, L. E.;Ziegler, M.;Lavine, I.;Zhang, J.;Cheng, Y.;Ul Ain, H.; | 2024 | Avoidant Automatic Thoughts are Associated with Task Avoidance and Inattention in the Moment | Cognitive Therapy and Research | Mental health disorders |
| 2204 | Liu, Z.;Li, J.;Zhang, Y.;Wu, D.;Huo, Y.;Yang, J.;Zhang, M.;Dong, C.;Jiang, L.;Sun, R.;Zhou, R.;Li, F.;Yu, X.;Zhu, D.;Guo, Y.;Chen, J.; | 2024 | Auxiliary Diagnosis of Children With Attention-Deficit/Hyperactivity Disorder Using Eye-Tracking and Digital Biomarkers: Case-Control Study | JMIR mHealth and uHealth | The population is not college students |
| 2205 | Gehdu, B. K.;Press, C.;Gray, K. L. H.;Cook, R.; | 2024 | Autistic adults have insight into their relative face recognition ability | Scientific Reports | The population is not college students |
| 2206 | Boyraz, G.;Waits, J. B.;Felix, V. A.; | 2014 | Authenticity, life satisfaction, and distress: a longitudinal analysis | J Couns Psychol | Mental health disorders |
| 2207 | Aristodemou, M. E.;Rommelse, N.;Kievit, R.; | 2024 | Attentiveness Modulates Reaction-Time Variability: Findings From a Population-Based Sample of 1032 Children | Collabra: Psychology | The population is not college students |
| 2208 | Paller, A. S.;Gonzalez, M. E.;Barnum, S.;Jaeger, J.;Shao, L.;Ozturk, Z. E.;Korotzer, A.; | 2024 | Attentiveness and mental health in adolescents with moderate-to-severe atopic dermatitis without ADHD | Archives of Dermatological Research | Mental health disorders |
| 2209 | Milligan, M. A.;Deyo, A. G.;Vrabec, | 2024 | Attention-deficit/hyperactivity symptoms and loss | Eating Behaviors | The population is not college |

| | | | | | |
|---|---|---|---|---|---|
| | A.;Snyder, M.;Kidwell, K. M.; | | of control eating in emerging adults: Role of psychological resilience | | students |
| 2210 | Namimi-Halevi, C.;Dor, C.;Dichtiar, R.;Bromberg, M.;Sinai, T.; | 2024 | Attention-deficit hyperactivity disorder is associated with risky and unhealthy behaviors among adolescents. | Public Health | Mental health disorders |
| 2211 | Vaucheret Paz, E.;Ortolá, R.;Cestari, M.;Cordoba, F.;Leist, M.;Petracca, L.;Chirilla, C.;Appendino, I.; | 2024 | Attentional Differences Between Isolated Attention Deficit Disorder and Attention Deficit Disorder Associated with Neurofibromatosis Type 1 | Revista Colombiana de Psiquiatria | Mental health disorders |
| 2212 | Combs, D. R.;Chapman, D.;Waguspack, J.;Basso, M. R.;Penn, D. L.; | 2011 | Attention shaping as a means to improve emotion perception deficits in outpatients with schizophrenia and impaired controls. | Schizophrenia research | Mental health disorders |
| 2213 | Khodarahimi, S.;Hashim, I. H. M.;Mohd-Zaharim, N.; | 2016 | Attachment Styles, Perceived Stress and Social Support in a Malaysian Young Adults Sample | Psychologica Belgica | The population is not college students |
| 2214 | So, Christine;Fiori, Katherine; | 2022 | Attachment anxiety and loneliness during the first year of college: Self-esteem and social support as mediators | Personality and Individual Differences | Mental health disorders |
| 2215 | Li, H.;Tong, J.;Wang, X.;Lu, M.;Yang, F.;Gao, H.;Gan, H.;Yan, S.;Gao, G.;Huang, K.;Cao, Y.;Tao, F.; | 2024 | Associations of prenatal exposure to individual and mixed organophosphate esters with ADHD symptom trajectories in preschool children: The modifying effects of maternal Vitamin D | Journal of Hazardous Materials | Mental health disorders |
| 2216 | Wang, W.;Wu, M.;Zhu, Z.;Ma, L.;Zhang, L.;Li, H.; | 2023 | Associations of mobile phone addiction with suicide ideation and suicide attempt: findings from six universities in China | Front Public Health | Mental health disorders |
| 2217 | Kearns, A.;Whitley, E.; | 2019 | Associations of Internet Access with Social Integration, wellbeing and physical activity among | BMC Public Health | The population is not college students |

| | | | | | |
|---|---|---|---|---|---|
| | | | adults in deprived communities: evidence from a household survey | | |
| 2218 | Panneh, M.;Ding, Q.;Kabuti, R.;Bradley, J.;Ngurukiri, P.;Kungu, M.;Abramsky, T.;Pollock, J.;Beksinska, A.;Shah, P.;Irungu, E.;Gafos, M.;Seeley, J.;Weiss, H. A.;Elzagallaai, A. A.;Rieder, M. J.;Kaul, R.;Kimani, J.;Beattie, T.; | 2024 | Associations of hair cortisol levels with violence, poor mental health, and harmful alcohol and other substance use among female sex workers in Nairobi, Kenya | Discover Mental Health | Mental health disorders |
| 2219 | Verfaellie, M.;Patt, V.;Lafleche, G.;Vasterling, J. J.; | 2024 | Associations between PTSD and temporal discounting: The role of future thinking | Journal of Behavior Therapy and Experimental Psychiatry | Mental health disorders |
| 2220 | Foroughi, B.;Griffiths, M. D.;Iranmanesh, M.;Salamzadeh, Y.; | 2022 | Associations Between Instagram Addiction, Academic Performance, Social Anxiety, Depression, and Life Satisfaction Among University Students | International Journal of Mental Health and Addiction | Mental health disorders |
| 2221 | Boyes, M. E.;Mah, M. A.;Hasking, P.; | 2023 | Associations between Family Functioning, Emotion Regulation, Social Support, and Self-injury among Emerging Adult University Students | Journal of Child and Family Studies | Mental health disorders |
| 2222 | Zhang, Z.;Tan, J.;Luo, Q.; | 2024 | Associations between breakfast skipping and outcomes in neuropsychiatric disorders, cognitive performance, and frailty: a Mendelian randomization study | BMC Psychiatry | Mental health disorders |
| 2223 | Turgoose, D.;Murphy, D.; | 2024 | Associations between Adverse Childhood | European Journal of | Mental health disorders |

| | | | | | |
|---|---|---|---|---|---|
| | | | Experiences (ACEs) and Complex-PTSD, moral injury, and perceived social support: A latent class analysis | Trauma and Dissociation | |
| 2224 | Guo, T. Y.;Zhang, Z. H.;Taylor, A.;Hall, D. L.;Yeung, A. S.;Kramer, A. F.;Zou, L. Y.; | 2022 | Association of social support with negative emotions among Chinese adolescents during Omicron-related lockdown of Shenzhen City: The roles of rumination and sleep quality | Frontiers in Psychiatry | Mental health disorders |
| 2225 | Zhou, L.;Li, Q.;Liu, S.;Wang, L.;Yu, M.;Lu, X.;Yang, L.;Lei, W.;Chen, G.; | 2024 | Association of inflammatory cytokines with magnetic resonance imaging features of the brain in patients with depression | Brain Research Bulletin | Mental health disorders |
| 2226 | Yang, M.;Li, J.;Fu, Y.;Wang, G.;Liu, M.;Chen, J.;Liu, J.; | 2024 | Association of childhood trauma, social support, cognition, and suicidality in females with bipolar disorder | BMC Psychiatry | Mental health disorders |
| 2227 | Kim, J. H.;Kim, J.;Jang, S. Y.;Park, E. C.; | 2024 | Association between watching eating broadcasts like mukbang and cooking and generalized anxiety disorder among Korean adolescents | BMC Psychiatry | Mental health disorders |
| 2228 | Borges, C. C.;Dos Santos, P. R.;Alves, P. M.;Borges, R. C. M.;Lucchetti, G.;Barbosa, M. A.;Porto, C. C.;Fernandes, M. R.; | 2021 | Association between spirituality/religiousness and quality of life among healthy adults: a systematic review | Health Qual Life Outcomes | The population is not college students |
| 2229 | Xian, X.;Zhang, Y.;Bai, A.;Zhai, X.;Hu, H.;Zhang, J.;Ye, M.; | 2022 | Association between Family Support, Stress, and Sleep Quality among College Students during the COVID-19 Online Learning Period | Int J Environ Res Public Health | Mental health disorders |
| 2230 | Laurene, K. R.;Kodukula, G.;Lechner, W. V.;Grega, C.;Lumpkin, E.;Kenne, D. R.; | 2024 | Assessment of psychological distress as a function of positive psychological variables during the COVID-19 pandemic: A university longitudinal | J Am Coll Health | Mental health disorders |

| | | | | | |
|---|---|---|---|---|---|
| | | | study | | |
| 2231 | Bölte, S.;Alehagen, L.;Black, M. H.;Hasslinger, J.;Wessman, E.;Remnélius, K. L.;Marschik, P. B.;D'Arcy, E.;Seidel, A.;Girdler, S.;Zander, E.; | 2024 | Assessment of functioning in ADHD according to World Health Organization standards: First revision of the International Classification of Functioning, Disability and Health Core Sets | Developmental Medicine and Child Neurology | Mental health disorders |
| 2232 | Awad, M. S. A.;Alrahim, M. M. A. A.;Awadelkareem, R. A. M.;Khalafallah Elhaj, M. A.;Abdelrahman Ibrahim, M. M.; | 2024 | Assessment of displaced Sudanese school-age children's mental health at Ad-Damar, River Nile, Sudan, 2024: a descriptive cross-sectional study | BMC Public Health | The population is not college students |
| 2233 | Almahmoud, O. H.;Abdallah, H. S.;Ahmad, A. A.;Judieh, I. M.;Kayed, D. N.;Abed, A. Y.; | 2024 | Assessment of attention-deficit / hyperactivity disorder signs among Palestinian school-age children | Journal of Pediatric Nursing | The population is not college students |
| 2234 | Çalışkan Pala, S.;Eyüboğlu, D.; | 2024 | Assessment of Alcohol Use Disorder Risk Profile, Suicide Probability, and Cognitions in University Students | Addicta: the Turkish Journal on Addictions | Mental health disorders |
| 2235 | Shahsavar, Y.;Choudhury, A.; | 2024 | Assessing user acceptance of a mental health app & its impact on depression and attention deficit hyperactivity disorder-related knowledge: A mixed method experimental study. | Journal of Behavioral and Cognitive Therapy | Mental health disorders |
| 2236 | Smith, S. K.;Manschot, C.;Kuhn, E.;Laber, E.;Somers, T. J.;Syrjala, K. L.;Applebaum, A. J.; | 2024 | Assessing the utility of the PC-PTSD-5 as a screening tool among a cancer survivor sample | Cancer | Mental health disorders |
| 2237 | Bhuiyan, Nishat;Kang, Jamie H.;Papalia, Zack;Bopp, Christopher M.;Bopp, Melissa;Mama, | 2022 | Assessing the stress-buffering effects of social support for exercise on physical activity, sitting time, and blood lipid profiles | Journal of American College Health | Mental health disorders |

| | | | | | |
|---|---|---|---|---|---|
| | Scherezade K.; | | | | |
| 2238 | Lagdon, S.;Ross, J.;Robinson, M.;Contractor, A. A.;Charak, R.;Armour, C.; | 2021 | Assessing the Mediating Role of Social Support in Childhood Maltreatment and Psychopathology Among College Students in Northern Ireland | J Interpers Violence | The population is not college students |
| 2239 | Enrique, A.;Mooney, O.;Salamanca-Sanabria, A.;Lee, C. T.;Farrell, S.;Richards, D.; | 2019 | Assessing the efficacy and acceptability of an internet-delivered intervention for resilience among college students: a pilot randomized control trial protocol | Internet interventions | Mental health disorders |
| 2240 | Enrique Roig, A.;Mooney, O.;Salamanca-Sanabria, A.;Lee, C. T.;Farrell, S.;Richards, D.; | 2020 | Assessing the Efficacy and Acceptability of a Web-Based Intervention for Resilience Among College Students: Pilot Randomized Controlled Trial | JMIR formative research | Mental health disorders |
| 2241 | Killaspy, H.;Dalton-Locke, C.;Clarke, C. S.;Leavey, G.;Igoumenou, A.;Arbuthnott, M.;Barrett, K.;Omar, R.; | 2024 | Assessing the clinical and cost-effectiveness of inpatient mental health rehabilitation services provided by the NHS and independent sector (ACER): protocol | BMC Psychiatry | Mental health disorders |
| 2242 | Hoare, F.;O'Donoghue, A.;Sweeney, C.;McCarthy, G.;Kavalidou, K.;Russell, V.;Norton, M. J.;Doherty, A. M.; | 2024 | Assessing the characteristics of suicidal ideation and self-harm in a national older adult population attending emergency departments across Ireland: Cohort study protocol | BMJ Open | Mental health disorders |
| 2243 | Kim, B. S. K.;Suh, H. N.;Subica, A.; | 2023 | Asian American child-parent cultural value discrepancies, family conflict, life satisfaction, and self-esteem | J Couns Psychol | The population is not college students |
| 2244 | Kirkland, A. E.;Langan, M. T.;Holton, K. F.; | 2022 | Artificial food coloring affects EEG power and ADHD symptoms in college students with ADHD: a pilot study. | Nutritional Neuroscience | Mental health disorders |

| | | | | | |
|---|---|---|---|---|---|
| 2245 | Stea, T. H.;Bonsaksen, T.;Smith, P.;Kleppang, A. L.;Steigen, A. M.;Leonhardt, M.;Lien, L.;Vettore, M. V.; | 2024 | Are social pressure, bullying, and low social support associated with depressive symptoms, self-harm, and self-directed violence among adolescents? A cross-sectional study using a structural equation modeling approach | BMC Psychiatry | Mental health disorders |
| 2246 | McCloskey, W.;Iwanicki, S.;Lauterbach, D.;Giammittorio, D. M.;Maxwell, K.; | 2015 | Are Facebook "Friends" Helpful? Development of a Facebook-Based Measure of Social Support and Examination of Relationships Among Depression, Quality of Life, and Social Support | Cyberpsychology Behavior and Social Networking | Mental health disorders |
| 2247 | Enns, K.;Ferdous, K. T.;Balasubramanian, S.;Ghosh, S.;Srinivasan, V.;Thomo, A.; | 2024 | Are brain networks classifiable? | Network Modeling Analysis in Health Informatics and Bioinformatics | Mental health disorders |
| 2248 | Shapiro, J. R.;Williams, A. M.;Hambarchyan, M.; | 2013 | Are all interventions created equal? A multi-threat approach to tailoring stereotype threat interventions | Journal of personality and social psychology | Mental health disorders |
| 2249 | Lovett, B. J.;Jordan, A. H.; | 2019 | Are ADHD Screeners Safe to Use? | Journal of Attention Disorders | Mental health disorders |
| 2250 | Beck-Hiestermann, F. M. L.;Hartung, L. K.;Richert, N.;Miethe, S.;Wiegand-Grefe, S.; | 2024 | Are 6 more accurate than 4? The influence of different modes of delivery on postpartum depression and PTSD | BMC Pregnancy and Childbirth | Mental health disorders |
| 2251 | Hurd, N. M.;Albright, J.;Wittrup, A.;Negrete, A.;Billingsley, J.; | 2018 | Appraisal Support from Natural Mentors, Self-worth, and Psychological Distress: Examining the Experiences of Underrepresented Students Transitioning Through College | J Youth Adolesc | Mental health disorders |
| 2252 | Zavala, E.;Kurtz, D. L.; | 2021 | Applying Differential Coercion and Social Support | Journal of | Mental health disorders |

| | | | | | |
|---|---|---|---|---|---|
| | | | Theory to Intimate Partner Violence | Interpersonal Violence | |
| 2253 | Flett, J. A. M.;Conner, T. S.;Riordan, B. C.;Patterson, T.;Hayne, H.; | 2020 | App-based mindfulness meditation for psychological distress and adjustment to college in incoming university students: a pragmatic, randomized, waitlist-controlled trial | Psychology & Health | Mental health disorders |
| 2254 | Jehi, T.;Mulvey, M.;Shulgan, E.;Burke, E.;Dean, M.;Betancourt, J.;Carliss, G.;Khan, R.;Majzoub, N.;Halawani, R.;Beeson, L.;Zeman, C.; | 2024 | Anxiety, Depression, Stress, and Test Anxiety are Inversely Associated with Academic Performance Among Undergraduate Students Post-COVID-19 Confinement | American Journal of Health Education | Mental health disorders |
| 2255 | Hou, X.;Elhai, J. D.;Hu, T.;She, Z.;Xi, J.; | 2023 | Anxiety symptoms and problematic smartphone use severity among Chinese college students: The moderating role of social support | Current Psychology | Mental health disorders |
| 2256 | Díaz-Jiménez, R. M. PhD;Caravaca-Sánchez, F. PhD;Martín-Cano, M. C. PhD;De la Fuente-Robles, Y. M. PhD; | 2020 | Anxiety levels among social work students during the COVID-19 lockdown in Spain | Soc Work Health Care | Mental health disorders |
| 2257 | Zhang, Q.;Wen, F.;Li, B.; | 2024 | Anxiety and depression in Chinese cataract patients: a network analysis | BMC Psychology | Mental health disorders |
| 2258 | Grineski, S. E.;Morales, D. X.;Collins, T. W.;Nadybal, S.;Trego, S.; | 2024 | Anxiety and depression among US college students engaging in undergraduate research during the COVID-19 pandemic | J Am Coll Health | Mental health disorders |
| 2259 | Chaliawala, K. S.;Vidourek, R. A.;King, K. A.; | 2024 | Anxiety among Asian international college students in the US: a systematic literature review | J Am Coll Health | Mental health disorders |
| 2260 | Al-Nawaiseh, H. K.;McIntosh, W. | 2022 | An-m-Health Intervention Using Smartphone App | International | Mental health disorders |

| | | | | | |
|---|---|---|---|---|---|
| | A.;McKyer, L. J.; | | to Improve Physical Activity in College Students: a Randomized Controlled Trial | journal of environmental research and public health | |
| 2261 | Wang, Y.;Liu, Q.;Liu, M.;Wang, W.;Ye, S.;Liu, X.;Liang, H.;Xue, X.;Gao, H.; | 2024 | Analysis of the therapeutic effect of pestle needle and EEG biofeedback and methylphenidate in the treatment of attention-deficit/hyperactivity disorder | Journal of Neurophysiology | Mental health disorders |
| 2262 | Fu, Y.;Chen, Y.;Wang, J.;Tang, X.;He, J.;Jiao, M.;Yu, C.;You, G.;Li, J.; | 2013 | Analysis of the prevalence of PTSD and its influencing factors among college students after the Wenchuan earthquake | Child Adolesc Psychiatry Ment Health | Mental health disorders |
| 2263 | Șipoș, R.;Văidean, T.;Răpciuc, A. M.;Poetar, C. R.;Predescu, E.; | 2024 | Analysing Digital Engagement Patterns: A Machine Learning Investigation into Social Anxiety Among Adolescents with ADHD | Journal of Clinical Medicine | Mental health disorders |
| 2264 | Liu, L.;Chen, J.;Liang, S.;Peng, X.;Yang, W.;Huang, A.;Wang, X.;Fan, F.;Zhao, J.; | 2023 | An Unusual College Experience: 16-Month Trajectories of Depressive Symptoms and Anxiety among Chinese New Undergraduate Students of 2019 during the COVID-19 Pandemic | Int J Environ Res Public Health | Mental health disorders |
| 2265 | Eddy, L. D.;Anastopoulos, A. D.;Dvorsky, M. R.;Silvia, P. J.;Labban, J. D.;Langberg, J. M.; | 2021 | An RCT of a CBT Intervention for Emerging Adults with ADHD Attending College: functional Outcomes | Journal of Clinical Child and Adolescent Psychology | Mental health disorders |
| 2266 | Pedersen, E. R.;D'Amico, E. J.;LaBrie, J. W.;Farris, C.;Klein, D. J.;Griffin, B. A.; | 2019 | An online alcohol and risky sex prevention program for college students studying abroad: study protocol for a randomized controlled trial | Addiction science & clinical practice | Mental health disorders |
| 2267 | Lee, D. J.;Crowe, M. L.;Weathers, F. | 2024 | An Item Response Theory Analysis of the | Assessment | Mental health disorders |

| | | | | | |
|---|---|---|---|---|---|
| | W.;Bovin, M. J.;Ellickson, S.;Sloan, D. M.;Schnurr, P.;Keane, T. M.;Marx, B. P.; | | Clinician-Administered PTSD Scale for DSM-5 Among Veterans | | |
| 2268 | Kuo, H. I.;Sun, J. L.;Nitsche, M.;Chang, J. C.; | 2024 | An investigation of the acute effects of aerobic exercise on executive function and cortical excitability in adolescents with attention deficit hyperactivity disorder (ADHD) | European Child and Adolescent Psychiatry | Mental health disorders |
| 2269 | Black, N.;Mullan, B.; | 2015 | An intervention to decrease heavy episodic drinking in college students: the effect of executive function training | Journal of American College Health | Mental health disorders |
| 2270 | Herrero, R.;Mira, A.;Cormo, G.;Etchemendy, E.;Banos, R.;Garcia-Palacios, A.;Ebert, D. D.;Franke, M.;Berger, T.;Schaub, M. P.;et al.,; | 2019 | An Internet-based intervention for improving resilience and coping strategies in university students: study protocol for a randomized controlled trial | Internet interventions | Mental health disorders |
| 2271 | Shafiq, S.; | 2024 | An exploration of psychological and socio-cultural facets in perinatal distress of Pakistani couples: a triangulated qualitative study | BMC Pregnancy and Childbirth | Mental health disorders |
| 2272 | Piszczor, J.;Kamm, J. M.;Jones, M.;Rubright, C.;Soble, J. R.; | 2024 | An Examination of WAIS-IV Digit Span Performance Inconsistency as a Novel Embedded Performance Validity Test Among Adults Clinically Referred for Attention-Deficit/Hyperactivity Disorder | Journal of Psychoeducational Assessment | The population is not college students |
| 2273 | Szlyk, H.;Motley, R.;Joe, S.;Nonas-Barnes, L.;Azasu, E.; | 2022 | An Examination of Suicidal Behavior among Black College Students with Exposure to Police Violence | Soc Work | Mental health disorders |

| | | | | | |
|---|---|---|---|---|---|
| 2274 | Flynn, M.;Kecmanovic, J.;Alloy, L. B.; | 2010 | An Examination of Integrated Cognitive-Interpersonal Vulnerability to Depression: The Role of Rumination, Perceived Social Support, and Interpersonal Stress Generation | Cognitive Therapy and Research | Published not from January 2010 to 31 March 2024 |
| 2275 | Lewis, M. A.;Neighbors, C.; | 2015 | An examination of college student activities and attentiveness during a web-delivered personalized normative feedback intervention | Psychology of addictive behaviors | Mental health disorders |
| 2276 | Wilkes-Gillan, S.;Parsons, L.;Parsons, D.;Mahoney, N.;Hancock, N.;Cordier, R.;Lincoln, M.;Chen, Y. W.;Bundy, A.; | 2024 | An evaluation of intervention appropriateness from the perspective of parents: A peer-mediated, play-based intervention for children with ADHD | Australian Occupational Therapy Journal | Mental health disorders |
| 2277 | Kumar, K.;Chaturvedi, R.; | 2018 | An empirical study of social support, stress, and life satisfaction among engineering graduates: the mediating role of perceived work/study life balance | International Journal of Happiness and Development | Mental health disorders |
| 2278 | Yan, Z.;Peacock, J.;Cohen, J. F. W.;Kurdziel, L.;Benes, S.;Oh, S.;Bowling, A.; | 2023 | An 8-Week Peer Health Coaching Intervention among College Students: a Pilot Randomized Study | Nutrients | Mental health disorders |
| 2279 | MacQueen, D. A.;Minassian, A.;Kenton, J. A.;Geyer, M. A.;Perry, W.;Brigman, J. L.;Young, J. W.; | 2018 | Amphetamine improves mouse and human attention in the 5-choice continuous performance test. | Neuropharmacolog y | Mental health disorders |
| 2280 | Zenker, M. L.;Bubeck, P.;Thieken, A. H.; | 2024 | Always on my mind: indications of post-traumatic stress disorder among those affected by the 2021 flood event in the Ahr Valley, Germany. | Natural Hazards and Earth System Sciences | Mental health disorders |
| 2281 | Saltz, R. F.;Paschall, M. | 2010 | Alcohol risk management in college settings: the | American journal of | Published not from January |

| | | | | | |
|---|---|---|---|---|---|
| | J.;McGaffigan, R. P.;Nygaard, P. M.; | | safer California universities randomized trial | preventive medicine | 2010 to 31 March 2024 |
| 2282 | Cain, S. M.;Rooney, E. A.;Cacace, S.;Post, A.;Russell, K.;Rasmussen, S.;Baker, J. C.;Cramer, R. J.; | 2024 | Adverse and benevolent Childhood experiences among adults in the United Kingdom: a latent class analysis | BMC Public Health | The population is not college students |
| 2283 | Lyvers, M.;Dark, S.;Jaguru, I.;Thorberg, F. A.; | 2024 | Adult symptoms of ASD and ADHD about alcohol use: Potential roles of transdiagnostic features | Alcohol | Mental health disorders |
| 2284 | Zhu, W.;Wang, C. D.;Chong, C. C.; | 2016 | Adult attachment, perceived social support, cultural orientation, and depressive symptoms: A moderated mediation model | J Couns Psychol | Mental health disorders |
| 2285 | Liu, C.;Ma, J. L.; | 2019 | Adult Attachment Orientations and Social Networking Site Addiction: The Mediating Effects of Online Social Support and the Fear of Missing Out | Front Psychol | Mental health disorders |
| 2286 | Li, X.;Sun, P.;Li, L.; | 2022 | Adult attachment and trait anxiety among Chinese college students: A multiple mediation model | Front Public Health | Mental health disorders |
| 2287 | Kim, S.;Liu, Z.;Glizer, D.;Tannock, R.;Woltering, S.; | 2014 | Adult ADHD and working memory: neural evidence of impaired encoding | Clinical Neurophysiology | Mental health disorders |
| 2288 | Suorsa, K. I.;Cushing, C. C.;Mullins, A. J.;Meier, E.;Tackett, A. P.;Junghans, A.;Chaney, J. M.;Mullins, L. L.; | 2016 | Adolescents and young adults with asthma and allergies: Physical activity, self-efficacy, social support, and subsequent psychosocial outcomes | Childrens Health Care | The population is not college students |
| 2289 | von Keyserlingk, L.;Moeller, J.;Heckhausen, J.;Eccles, J. S.;Arum, R.; | 2024 | Adjusting to college—Do ability beliefs and confidence in getting support matter for performance and mental health? | Zeitschrift fur Erziehungswissenschaft | Unpublished journal article |
| 2290 | Cunniff, P. J.;Ahsan, A.;McCrary, | 2024 | ADHD prescription patterns and medication | BMC Psychiatry | Mental health disorders |

| | | | | | |
|---|---|---|---|---|---|
| | C.;Dien, T.;Kuhn, T. H.;Vazifedan, T.;Harrington, J. W.; | | adherence in children and adolescents during the COVID-19 pandemic in an urban academic setting | | |
| 2291 | Swanepoel, A.; | 2024 | ADHD AND ASD ARE NORMAL BIOLOGICAL VARIATIONS AS PART OF HUMAN EVOLUTION AND ARE NOT "DISORDERS" | Clinical Neuropsychiatry | Mental health disorders |
| 2292 | da Silva Leandro, S. I.;Alves, K. C. M.;de Oliveira, F. Í S.;de Aquino Matoso, L. Y.;Zamoner, B. M.;Romano, T. M.;de Lima Nardin, K.;Cavalcante, F. S. B. M.;Müller, P. G.;Gervásio, B. C.;Gusmão, C. T. P.;dos Santos, J. C. C.; | 2024 | Addressing treatment hurdles in adults with late-onset attention-deficit/hyperactivity disorder: a detailed case report | Egyptian Journal of Neurology, Psychiatry and Neurosurgery | The population is not college students |
| 2293 | Valencia, P. D.;de la Rosa-Gómez, A.;Barba-Sánchez, A. E.;Hernández-Posadas, A.;Hernández-Jiménez, M. J.; | 2024 | Adaptation of the Posttraumatic Stress Disorder Checklist for DSM-5: A Short Version in a Help-seeking Sample | Avances en Psicologia Latinoamericana | Mental health disorders |
| 2294 | Perrino, T.;Lozano, A.;Estrada, Y.;Tapia, M. I.;Brown, C. H.;Horigian, V. E.;Beardslee, W. R.;Prado, G.; | 2024 | Adaptation of an evidence-based, preventive intervention to promote mental health in Hispanic adolescents: eHealth Familias Unidas Mental Health | Translational Behavioral Medicine | Mental health disorders |
| 2295 | Huang, Z.;Xiao, X.;Liu, C.;Cai, Q.;Liu, C.;Tan, Q.;Zhan, Y.; | 2023 | Acute Social Stress Influences Moral Decision-Making Under Different Social Distances in Young Healthy Men | Experimental psychology | Mental health disorders |
| 2296 | Huang, J.;Deng, F.;Wu, S.;Zhao, Y.;Shima, M.;Guo, B.;Liu, Q.;Guo, | 2016 | Acute effects on pulmonary function in young healthy adults exposed to traffic-related air | Environmental health and | The population is not college students |

| | | | | | |
|---|---|---|---|---|---|
| | X.; | | pollution in semi-closed transport hub in Beijing | preventive medicine | |
| 2297 | Su, H.;Chang, N. J.;Wu, W. L.;Guo, L. Y.;Chu, I. H.; | 2017 | Acute Effects of Foam Rolling, Static Stretching, and Dynamic Stretching During Warm-ups on Muscular Flexibility and Strength in Young Adults | Journal of Sport Rehabilitation | The population is not college students |
| 2298 | Howard, M. A.;Marczinski, C. A.; | 2010 | Acute effects of a glucose energy drink on behavioral control | Experimental and clinical psychopharmacology | Published not from January 2010 to 31 March 2024 |
| 2299 | Kuo, H. I.;Nitsche, M. A.;Wu, Y. T.;Chang, J. C.;Yang, L. K.; | 2024 | Acute aerobic exercise modulates cognition and cortical excitability in adults with attention-deficit hyperactivity disorder (ADHD) and healthy controls. | Psychiatry Research | Mental health disorders |
| 2300 | Hendricks, D. J.;Sampson, E.;Rumrill, P.;Leopold, A.;Elias, E.;Jacobs, K.;Nardone, A.;Scherer, M.;Stauffer, C.; | 2015 | Activities and interim outcomes of a multi-site development project to promote cognitive support technology use and employment success among postsecondary students with traumatic brain injuries | NeuroRehabilitation | Mental health disorders |
| 2301 | Albright, J. N.;Hurd, N. M.; | 2023 | Activism, Social Support, and Trump-Related Distress: Exploring Associations With Mental Health | Journal of Diversity in Higher Education | Mental health disorders |
| 2302 | Tamir, Y.;Novak, A. M.;Cohen, I.;Adini, B.;Lev-Ari, S.; | 2024 | Active Engagement: The Impact of Group-Based Physical Activities on the Resilience of Israeli Adolescents with ADHD | Journal of Clinical Medicine | Mental health disorders |
| 2303 | Craig, D. W.;Perkins, H. W.; | 2018 | Accuracy of Estimated Blood Alcohol Concentration Norms From College Student Drinking Survey Data: verification Using Matched | Journal of studies on alcohol and drugs | Mental health disorders |

| | | | | | |
|---|---|---|---|---|---|
| | | | Late-Night Breath Measurements | | |
| 2304 | Zang, C.;Hou, Y.;Lyu, D.;Jin, J.;Sacco, S.;Chen, K.;Aseltine, R.;Wang, F.; | 2024 | Accuracy and transportability of machine learning models for adolescent suicide prediction with longitudinal clinical records | Translational Psychiatry | Mental health disorders |
| 2305 | Franco, M.;Hsiao, Y. S.;Gnilka, P. B.;Ashby, J. S.; | 2019 | Acculturative stress, social support, and career outcome expectations among international students | International Journal for Educational and Vocational Guidance | Mental health disorders |
| 2306 | Wähnke, L.;Plück, J.;Bodden, M.;Ernst, A.;Klemp, M. T.;Mühlenmeister, J.;Döpfner, M.; | 2024 | Acceptance and utilization of web-based self-help for caregivers of children with externalizing disorders | Child and Adolescent Psychiatry and Mental Health | The population is not college students |
| 2307 | Underwood, S. B.;White, S. V.;Forsyth, J. P.; | 2024 | Acceptance and Commitment Therapy (ACT) and Self-Compassion for Generalized Anxiety Disorder and Comorbid Major Depressive Disorder: A Case Study | Clinical Case Studies | Mental health disorders |
| 2308 | Schipani-McLaughlin, A. M.;Salazar, L. F.;Leone, R. M.;Balser, D.;Hunley, K.;Quinn-Echevarria, K.; | 2024 | Acceptability of RealConsent: a Sexual Violence Web-Based Risk Reduction Program for College Women | Health education & behavior | Mental health disorders |
| 2309 | Restrepo, J. E.;Cardona, E. Y. B.;Montoya, G. P. C.;Cassaretto, M.;Vilela, P.; | 2023 | Academic stress and adaptation to university life: mediation of cognitive-emotional regulation and social support | Anales De Psicologia | Mental health disorders |
| 2310 | Cheng, J.;Zhao, Y. Y.;Wang, J.;Sun, Y. H.; | 2020 | Academic burnout and depression of Chinese medical students in the pre-clinical years: the | Psychology Health & Medicine | Mental health disorders |

| | | | | | |
|---|---|---|---|---|---|
| | | | buffering hypothesis of resilience and social support | | |
| 2311 | Chen, S.;Xue, B.;Zhou, R.;Qian, A.;Tao, J.;Yang, C.;Huang, X.;Wang, M.; | 2024 | Abnormal stability of dynamic functional architecture in drug-naïve children with attention-deficit/hyperactivity disorder | BMC Psychiatry | The population is not college students |
| 2312 | Liu, Q.;Liao, W.;Yang, L.;Cao, L.;Liu, N.;Gu, Y.;Wang, S.;Xu, X.;Wang, H.; | 2024 | The aberrant amplitude of low-frequency fluctuation and functional connectivity in children with different subtypes of ADHD: a resting-state fNIRS study | BMC Psychiatry | Mental health disorders |
| 2313 | Han, J.;Slade, A.;Fujimoto, H.;Zheng, W. Y.;Shvetcov, A.;Hoon, L.;Funke Kupper, J.;Senadeera, M.;Gupta, S.;Venkatesh, S.;Mouzakis, K.;Gu, Y.;Bilgrami, A.;Saba, N.;Cutler, H.;Batterham, P.;Boydell, K.;Shand, F.;Whitton, A.;Christensen, H.; | 2024 | A web-based video messaging intervention for suicide prevention in men: study protocol for a five-armed randomized controlled trial | Trials | Mental health disorders |
| 2314 | Salazar, L. F.;Schipani-McLaughlin, A. M.;Sebeh, Y.;Nizam, Z.;Hayat, M.; | 2023 | A Web-Based Sexual Violence, Alcohol Misuse, and Bystander Intervention Program for College Women (RealConsent): Randomized Controlled Trial | Journal of medical Internet research | Mental health disorders |
| 2315 | Salazar, L. F.;Vivolo-Kantor, A.;Hardin, J.;Berkowitz, A.; | 2014 | A web-based sexual violence bystander intervention for male college students: randomized controlled trial | Journal of medical Internet research | Mental health disorders |
| 2316 | Liang, W.;Duan, Y. P.;Shang, B. R.;Wang, Y. P.;Hu, C.;Lippke, S.; | 2019 | A web-based lifestyle intervention program for Chinese college students: study protocol and | BMC Public Health | Mental health disorders |

| | | | | | |
|---|---|---|---|---|---|
| | | | baseline characteristics of a randomized placebo-controlled trial | | |
| 2317 | Haws, K.;Mak, S.;Greer, S.;Kussin, C. A.;Sacra, E.;Carlson, C. J.;McManus, P.;Varon, S.;Chandler, H.;Osinubi, O.; | 2024 | A Virtual Functional Medicine-Based Interdisciplinary and Integrative Intervention for Gulf War Illness | Military Medicine | Mental health disorders |
| 2318 | Howell, R. T.;Rodzon, K. S.;Kurai, M.;Sanchez, A. H.; | 2010 | A validation of well-being and happiness surveys for administration via the internet | Behavior Research Methods | Published not from January 2010 to 31 March 2024 |
| 2319 | Ceglarek, P. J. D.;Ward, L. M.; | 2016 | A tool for help or harm? How associations between social networking use, social support, and mental health differ for sexual minority and heterosexual youth | Computers in Human Behavior | Mental health disorders |
| 2320 | West, D. S.;Monroe, C. M.;Turner-McGrievy, G.;Sundstrom, B.;Larsen, C.;Magradey, K.;Wilcox, S.;Brandt, H. M.; | 2016 | A Technology-Mediated Behavioral Weight Gain Prevention Intervention for College Students: controlled, Quasi-Experimental Study | Journal of medical Internet research | Mental health disorders |
| 2321 | Ashraf, I.;Jung, S.;Hur, S.;Park, Y.; | 2024 | A systematic literature review of neuroimaging coupled with machine learning approaches for diagnosis of attention deficit hyperactivity disorder. | Journal of Big Data | Mental health disorders |
| 2322 | Zhan, N.;Li, F.;Fung, H. W.;Zhang, K.;Wang, J.;Geng, F.; | 2024 | A symptom-level perspective on irritability, PTSD, and depression in children and adults | Journal of Affective Disorders | Mental health disorders |
| 2323 | Beneria, A.;Motger-Albertí, A.;Quesada-Franco, M.;Arteaga-Henríquez, G.;Santesteban-Echarri, O.;Ibáñez, | 2024 | A Suicide Attempt Multicomponent Intervention Treatment (SAMIT Program): study protocol for a multicentric randomized controlled trial | BMC Psychiatry | Mental health disorders |

| | | | | | |
|---|---|---|---|---|---|
| | P.;Parramon-Puig, G.;Sanz-Correcher, P.;Galynker, I.;Ramos-Quiroga, J. A.;Pintor, L.;Bruguera, P.;Braquehais, M. D.; | | | | |
| 2324 | Ji, Lei;Chen, Changfeng;Hou, Binyin;Ren, Decheng;Fan, Yuan;Liu, Liangjie;Bi, Yan;Guo, Zhenming;Yang, Fengping;Wu, Xi;Li, Xingwang;Liu, Chuanxin;Zuo, Zhen;Zhang, Rong;Yi, Zhenghui;Xu, Yifeng;He, Lin;Shi, Yi;Yu, Tao;He, Guang; | 2021 | A study of negative life events driven depressive symptoms and academic engagement in Chinese college students | Scientific Reports (Nature Publisher Group) | Mental health disorders |
| 2325 | Terui, S.;Goldsmith, J. V.;Daugherty, C. D.;Briscoe, J. R.; | 2021 | A student-driven HIV/PrEP communication intervention using a modified social network strategy | J Am Coll Health | Mental health disorders |
| 2326 | Rajabi, M.;Bagian Kulehmarzi, M. J.;Dastnaei, T. M.; | 2024 | A Structural Model of Cognitive Reactivity and Early Life Experiences with Suicide Resiliency: The Mediation of Psychological Pain | International Journal of High-Risk Behaviors and Addiction | Mental health disorders |
| 2327 | de Oliveira Assumpcao, C.;Barreto, R. V.;de Lima, L. C. R.;Cardozo, A. C.;de Lima Montebelo, M. I.;Catarino, H. R. C.;Greco, C. C.;Denadai, B. S.; | 2020 | A single bout of downhill running attenuates subsequent level running-induced fatigue. | Scientific Reports | Not related wellbeing |
| 2328 | Patrick, M. E.;Boatman, J. | 2020 | A sequential multiple assignment randomized trial | Contemporary | Mental health disorders |

| | | | | | |
|---|---|---|---|---|---|
| | A.;Morrell, N.;Wagner, A. C.;Lyden, G. R.;Nahum-Shani, I.;King, C. A.;Bonar, E. E.;Lee, C. M.;Larimer, M. E.;et al.,; | | (SMART) protocol for empirically developing an adaptive preventive intervention for college student drinking reduction | Clinical Trials | |
| 2329 | Wang, S.;Cannata, B.;Vallurupalli, M.;Yenikomshian, H. A.;Gillenwater, J.;Stoycos, S. A.; | 2024 | A Scoping Review of PTSD and Depression in Adult Burn Patients: A Call for Standardized Screening and Intervention Research | Journal of Burn Care and Research | Mental health disorders |
| 2330 | Glass, N.;Clough, A.;Case, J.;Hanson, G.;Barnes-Hoyt, J.;Waterbury, A.;Alhusen, J.;Ehrensaft, M.;Grace, K. T.;Perrin, N.; | 2015 | A safety app to respond to dating violence for college women and their friends: the MyPlan study randomized controlled trial protocol | BMC Public Health | Mental health disorders |
| 2331 | Brown, J.; | 2013 | A review of the evidence on technology-based interventions for the treatment of tobacco dependence in college health | Worldviews Evid-Based Nurs | Mental health disorders |
| 2332 | Maymon, R.;Hall, N. C.; | 2021 | A Review of First-Year Student Stress and Social Support | Social Sciences-Basel | Mental health disorders |
| 2333 | Gex, K. S.;Mun, E. Y.;Barnett, N. P.;McDevitt-Murphy, M. E.;Ruggiero, K. J.;Thurston, I. B.;Olin, C. C.;Voss, A. T.;Withers, A. J.;Murphy, J. G.; | 2023 | A randomized pilot trial of a mobile delivered brief motivational interviewing and behavioral economic alcohol intervention for emerging adults | Psychology of addictive behaviors | The population is not college students |
| 2334 | Richman, A. R.;Maddy, L.;Torres, E.;Goldberg, E. J.; | 2016 | A randomized intervention study to evaluate whether electronic messaging can increase human papillomavirus vaccine completion and knowledge among college students | Journal of American College Health | Mental health disorders |

| | | | | | |
|---|---|---|---|---|---|
| 2335 | Levin, M. E.;Krafft, J.;Hicks, E. T.;Pierce, B.;Twohig, M. P.; | 2020 | A randomized dismantling trial of the open and engaged components of acceptance and commitment therapy in an online intervention for distressed college students | Behavior research and therapy | Mental health disorders |
| 2336 | Gilmore, A. K.;Lewis, M. A.;George, W. H.; | 2015 | A randomized controlled trial targeting alcohol use and sexual assault risk among college women at high risk for victimization | Behavior research and therapy | Mental health disorders |
| 2337 | Wen, A.;Wolitzky-Taylor, K.;Gibbons, R. D.;Craske, M.; | 2023 | A randomized controlled trial on using a predictive algorithm to adapt the level of psychological care for community college students: STAND triaging and adapting to the level of care study protocol | Trials | Unpublished journal article |
| 2338 | Baumgartner PhD, J. N.;Schneider PhD, T. R.; | 2023 | A randomized controlled trial of mindfulness-based stress reduction on academic resilience and performance in college students | Journal of American College Health | Mental health disorders |
| 2339 | Liu, W.;Yuan, J.;Wu, Y.;Xu, L.;Wang, X.;Meng, J.;Wei, Y.;Zhang, Y.;Kang, C. Y.;Yang, J. Z.; | 2024 | A randomized controlled trial of mindfulness-based cognitive therapy for major depressive disorder in undergraduate students: dose-response effect, inflammatory markers and BDNF | Psychiatry Research | Mental health disorders |
| 2340 | Gu, Y.;Xu, G.;Zhu, Y.; | 2018 | A Randomized Controlled Trial of Mindfulness-Based Cognitive Therapy for College Students With ADHD | Journal of Attention Disorders | Mental health disorders |
| 2341 | Murphy, J. G.;Dennhardt, A. A.;Skidmore, J. R.;Borsari, B.;Barnett, N. P.;Colby, S. M.;Martens, M. P.; | 2012 | A randomized controlled trial of a behavioral economic supplement to brief motivational interventions for college drinking | Journal of consulting and clinical psychology | Mental health disorders |

| | | | | | |
|---|---|---|---|---|---|
| 2342 | Anastopoulos, A. D.;Langberg, J. M.;Eddy, L. D.;Silvia, P. J.;Labban, J. D.; | 2021 | A randomized controlled trial examining CBT for college students with ADHD | Journal of consulting and clinical psychology | Mental health disorders |
| 2343 | Dvorak, R. D.;Troop-Gordon, W.;Stevenson, B. L.;Kramer, M. P.;Wilborn, D.;Leary, A. V.; | 2018 | A randomized control trial of a deviance regulation theory intervention to increase alcohol protective strategies | Journal of consulting and clinical psychology | Mental health disorders |
| 2344 | Sullivan, A. J.;Anderson, J.;Beatty, M.;Choi, J.;Jaccard, J.;Hawkins, K.;Pearlson, G.;Stevens, M. C.; | 2024 | A randomized clinical trial to evaluate the feasibility, tolerability, and preliminary target engagement for a novel executive working memory training in adolescents with ADHD | Behavior research and therapy | Mental health disorders |
| 2345 | Murphy, J. G.;Dennhardt, A. A.;Martens, M. P.;Borsari, B.;Witkiewitz, K.;Meshesha, L. Z.; | 2019 | A randomized clinical trial evaluating the efficacy of a brief alcohol intervention supplemented with a substance-free activity session or relaxation training | Journal of consulting and clinical psychology | Mental health disorders |
| 2346 | James, Leon; | 2016 | A psychobiological model for managing student engagement in online courses using gamification principles | Webology | Unpublished journal article |
| 2347 | Plettinckx, E.;Berndt, N.;Seixas, R.;De Smet, S.;Antoine, J.;Bruggeman, H.;Harth, N.;Papadopoulou, A.;Bafi, I.;Fotiou, A.;Pridotkienė, E.;Kalinauskaitė, R.;Rašimaitė, B.;Tsiakkirou, M.;Balcaen, M.;Fernandez, K.;Gremeaux, L.;Dirkx, N.;De Ridder, K.;Yiasemi, | 2024 | A protocol for identifying the needs related to drug use, health, and social (re)integration of people living in prison within five European countries | Archives of Public Health | Mental health disorders |

| | | | | | |
|---|---|---|---|---|---|
| 2348 | Sit, H. F.;Li, G.;Chen, W.;Sou, E. K. L.;Wong, M.;Burchert, S.;Hong, I. W.;Sit, H. Y.;Lam, A. I. F.;Hall, B. J.; | 2022 | A protocol for a type 1 effectiveness-implementation randomized controlled trial of the WHO digital mental health intervention Step-by-Step to address depression among Chinese young adults in Macao (SAR), China | Internet interventions | The population is not college students |
| 2349 | Rasmussen, S.;Chandler, J. F.;Russell, K.;Cramer, R. J.; | 2024 | A prospective examination of sleep chronotype and future suicide intent among adults in the United Kingdom: A test of the integrated motivational volitional model of suicide | Sleep medicine | Mental health disorders |
| 2350 | Harackiewicz, J. M.;Hecht, C. A.;Asher, M. W.;Beymer, P. N.;Lamont, L. B.;Wheeler, N. S.;Else-Quest, N. M.;Priniski, S. J.;Smith, J. L.;Hyde, J. S.;et al.,; | 2023 | A prosocial value intervention in Gateway STEM courses | Journal of personality and social psychology | Mental health disorders |
| 2351 | Edwards, K. M.;Waterman, E. A.;Dardis, C. M.;Ullman, S. E.;Rodriguez, L. M.;Dworkin, E. R.; | 2021 | A program to improve social reactions to sexual and dating violence disclosures reduces posttraumatic stress in subsequently victimized participants. | Psychol Trauma | Mental health disorders |
| 2352 | Sleath, B.;Beznos, B.;Carpenter, D.;Thomas, K.;Annis, I.;Tudor, G.;Garcia, N.;Adjei, A.;Anastopoulos, A.;Leslie, L.;Coyne, I.; | 2024 | A pre-visit video/question prompt list intervention to increase youth question-asking about attention deficit hyperactivity disorder during pediatric visits | Patient Education and Counseling | Mental health disorders |
| 2353 | Goshvarpour, A.;Goshvarpour, A.;Abbasi, A.; | 2022 | A Predictive Model for Emotion Recognition Based on Individual Characteristics and | Basic Clin Neurosci | Unpublished journal article |

| | | | | | |
|---|---|---|---|---|---|
| | | | Autonomic Changes | | |
| 2354 | Ruehlman, L.;Karoly, P.; | 2023 | A pilot test of Internet-delivered brief interactive training sessions for depression: evaluating dropout, uptake, adherence, and outcome | Journal of American College Health | Mental health disorders |
| 2355 | Blow, J.;Sagaribay, R.;Cooper, T. V.; | 2022 | A pilot study examining the impact of a brief health education intervention on food choices and exercise in a Latinx college student sample | Appetite | Mental health disorders |
| 2356 | Dale, S. K.;Wright, I. A.;Madhu, A.;Reid, R.;Shahid, N. N.;Wright, M.;Sanders, J.;Phillips, A.;Rodriguez, A.;Safren, S. A.; | 2024 | A Pilot Randomized Control Trial of the Striving Towards EmPowerment and Medication Adherence (STEP-AD) Intervention for Black Women Living with HIV | AIDS and Behavior | Mental health disorders |
| 2357 | Holt, L. J.;Armeli, S.;Tennen, H.;Austad, C. S.;Raskin, S. A.;Fallahi, C. R.;Wood, R.;Rosen, R. I.;Ginley, M. K.;Pearlson, G. D.; | 2013 | A person-centered approach to understanding negative reinforcement drinking among first-year college students | Addict Behav | Unpublished journal article |
| 2358 | Newcomb-Anjo, S. E.;Barker, E. T.;Howard, A. L.; | 2017 | A Person-Centered Analysis of Risk Factors that Compromise Wellbeing in Emerging Adulthood | Journal of Youth and Adolescence | Not related to research questions |
| 2359 | Sun, W. H.;Wong, C. K. H.;Wong, W. C. W.; | 2017 | A Peer-Led, Social Media-Delivered, Safer Sex Intervention for Chinese College Students: Randomized Controlled Trial | Journal of medical Internet research | Mental health disorders |
| 2360 | LaBrie, J. W.;Earle, A. M.;Boyle, S. C.;Hummer, J. F.;Montes, K.;Turrisi, R.;Napper, L. E.; | 2016 | A parent-based intervention reduces heavy episodic drinking among first-year college students. | Psychology of addictive behaviors | Mental health disorders |
| 2361 | Liu, L.;Ji, Y.;Gao, Y.;Li, T.;Xu, W.; | 2022 | A Novel Stress State Assessment Method for College Students Based on EEG | Comput Intell Neurosci | Mental health disorders |

| | | | | | |
|---|---|---|---|---|---|
| 2362 | Mei, X.;Wang, X.;Huang, W.;Zhu, J.;Liu, K.;Wang, X.;Cai, W.;He, R.; | 2024 | A novel polycaprolactone/polypyrrole/β-cyclodextrin electrochemical flexible sensor for dinotefuran pesticide detection | Food Chem | Unpublished journal article |
| 2363 | Özaslan, A.;Sevri, M.;İşeri, E.;Karacan, B.;Cengiz, M.;Karacan, H.;Sarıpınar, E. G.;Dikmen, A. U.;Güney, E.; | 2024 | A New Objective Diagnostic Tool for Attention-Deficit Hyper-Activity Disorder (ADHD): Development of the Distractor-Embedded Auditory Continuous Performance Test | Journal of Clinical Medicine | Mental health disorders |
| 2364 | Esmaeili Rad, M.;Ahmadi, F.; | 2018 | A new method to measure and decrease online social networking addiction | Asia-Pacific psychiatry | Mental health disorders |
| 2365 | Karr, J. E.;Rodriguez, J. E.;Rast, P.;Goh, P. K.;Martel, M. M.; | 2024 | A Network Analysis of Executive Functions in Children and Adolescents With and Without Attention-Deficit/Hyperactivity Disorder | Child Psychiatry and Human Development | The population is not college students |
| 2366 | Pedersen, E. R.;Hummer, J. F.;Davis, J. P.;Fitzke, R. E.;Christie, N. C.;Witkiewitz, K.;Clapp, J. D.; | 2022 | A mobile-based pregaming drinking prevention intervention for college students: study protocol for a randomized controlled trial | Addiction science & clinical practice | Mental health disorders |
| 2367 | Pedersen, E. R.;Hummer, J. F.;Davis, J. P.;Fitzke, R. E.;Tran, D. D.;Witkiewitz, K.;Clapp, J. D.; | 2023 | A mobile-based pregaming drinking prevention intervention for college students: a pilot randomized controlled trial | Psychology of addictive behaviors | Mental health disorders |
| 2368 | Luo, J.;Li, F.;Wu, Y.;Liu, X.;Zheng, Q.;Qi, Y.;Huang, H.;Xu, G.;Liu, Z.;He, F.;Zheng, Y.; | 2024 | A mobile device-based game prototype for ADHD: development and preliminary feasibility testing | Translational Psychiatry | Mental health disorders |
| 2369 | Cecchin, H. F. G.;da Costa, H. E. R.;Pacheco, G. R.;de Valencia, G. B.;Murta, S. G.; | 2024 | A mixed methods study of suicide protective factors in college students | Psicologia: Reflexao e Critica | Mental health disorders |

| | | | | | |
|---|---|---|---|---|---|
| 2370 | Kirlic, N.;Akeman, E.;DeVille, D. C.;Yeh, H. W.;Cosgrove, K. T.;McDermott, T. J.;Touthang, J.;Clausen, A.;Paulus, M. P.;Aupperle, R. L.; | 2023 | A machine learning analysis of risk and protective factors of suicidal thoughts and behaviors in college students | J Am Coll Health | Mental health disorders |
| 2371 | Wu, J.;Liu, Q.; | 2023 | A longitudinal study on college students' depressive symptoms during the COVID-19 pandemic: The trajectories, antecedents, and outcomes | Psychiatry Res | Mental health disorders |
| 2372 | Price, M.;Hidalgo, J. E.;Bird, Y. M.;Bloomfield, L. S. P.;Buck, C.;Cerutti, J.;Dodds, P. S.;Fudolig, M. I.;Gehman, R.;Hickok, M.;et al.,; | 2023 | A large clinical trial to improve well-being during the transition to college using wearables: the lived experiences measured using rings study | Contemporary Clinical Trials | Mental health disorders |
| 2373 | Tanner, A. E.;Guastaferro, K. M.;Rulison, K. L.;Wyrick, D. L.;Milroy, J. J.;Bhandari, S.;Thorpe, S.;Ware, S.;Miller, A. M.;Collins, L. M.; | 2021 | A Hybrid Evaluation-Optimization Trial to Evaluate an Intervention Targeting the Intersection of Alcohol and Sex in College Students and Simultaneously Test an Additional Component Aimed at Preventing Sexual Violence | Annals of Behavioral Medicine | Mental health disorders |
| 2374 | Ghosh, A.;Cohen, K. A.;Jans, L.;Busch, C. A.;McDanal, R.;Yang, Y.;Cooper, K. M.;Schleider, J. L.; | 2023 | A Digital Single-Session Intervention (Project Engage) to Address Fear of Negative Evaluation Among College Students: Pilot Randomized Controlled Trial | JMIR mental health | Mental health disorders |
| 2375 | White, M. E.;Shih, J. H.; | 2012 | A daily diary study of co-rumination, stressful life events, and depressed mood in late adolescents | J Clin Child Adolesc Psychol | Mental health disorders |
| 2376 | Salamanca-Sanabria, A.;Richards, D.;Timulak, L.;Connell, S.;Mojica | 2020 | A culturally adapted cognitive behavioral internet-delivered intervention for depressive | JMIR mental health | Mental health disorders |

| | | | | | |
|---|---|---|---|---|---|
| | Perilla, M.;Parra-Villa, Y.;Castro-Camacho, L.; | | symptoms: randomized controlled trial | | |
| 2377 | Schaefer, L. M.;Howell, K. H.;Schwartz, L. E.;Bottomley, J. S.;Crossnine, C. B.; | 2018 | A concurrent examination of protective factors associated with resilience and posttraumatic growth following childhood victimization | Child Abuse Negl | The population is not college students |
| 2378 | Weaver, C. C.;Leffingwell, T. R.;Lombardi, N. J.;Claborn, K. R.;Miller, M. E.;Martens, M. P.; | 2014 | A computer-based feedback-only intervention with and without a moderation skills component | Journal of Substance Abuse Treatment | Mental health disorders |
| 2379 | Karambelas, G. J.;Allott, K. A.;Byrne, L. K.;Jayasinghe, A.;Hasty, M.;Macneil, C.;Watson, A. E.;Filia, K.;Cotton, S. M.; | 2024 | A comparison of challenging and positive caregiving experiences for caregivers of individuals with schizophrenia spectrum and bipolar disorders | Journal of Affective Disorders Reports | Mental health disorders |
| 2380 | King, S. C.;Richner, K. A.;Tuliao, A. P.;Kennedy, J. L.;McChargue, D. E.; | 2020 | A comparison between telehealth and face-to-face delivery of a brief alcohol intervention for college students | Substance abuse | Mental health disorders |
| 2381 | Bornheimer, L. A.;Verdugo, J. L.;Brdar, N. M.;Im, V.;Jeffers, N.;Bushnell, C. B.;Hoener, K.;Tasker, M.;DeWeese, K.;Florence, T.;Jester, J. M.;King, C. A.;Taylor, S. F.;Himle, J. A.; | 2024 | A cognitive-behavioral treatment for suicide prevention among adults with schizophrenia spectrum disorders in community mental health: Study protocol for a pilot feasibility and acceptability randomized clinical trial | Pilot and Feasibility Studies | Mental health disorders |
| 2382 | Lee, Catherine;Dickson, Daniel A.;Conley, Colleen S.;Holmbeck, Grayson N.; | 2014 | A Closer Look at Self-Esteem, Perceived Social Support, and Coping Strategy: A Prospective Study of Depressive Symptomatology Across the Transition to College | Journal of Social and Clinical Psychology | Mental health disorders |
| 2383 | Yen, J. Y.;Király, O.;Griffiths, M. | 2024 | A case-control study for psychiatric comorbidity | Journal of | Mental health disorders |

| | | | | | |
|---|---|---|---|---|---|
| | D.;Demetrovics, Z.;Ko, C. H.; | | and associative factors of gaming disorder and hazardous gaming based on ICD-11 criteria: Cognitive control, emotion regulation, and reinforcement sensitivity | Behavioral Addictions | |
| 2384 | Mermelstein, L. C.;Garske, J. P.; | 2015 | A Brief Mindfulness Intervention for College Student Binge Drinkers: a Pilot Study | Psychology of addictive behaviors | Mental health disorders |
| 2385 | Klaeth, J. R.;Jensen, A. G.;Auren, T. J. B.;Solem, S.; | 2024 | 12-month follow-up of intensive outpatient treatment for PTSD combining prolonged exposure therapy, EMDR, and physical activity | BMC Psychiatry | Mental health disorders |
| 2386 | Avella, H.; | 2024 | "TikTok ≠ therapy": Mediating mental health and algorithmic mood disorders | New Media and Society | Mental health disorders |
| 2387 | Hooyer, K.;Hamblen, J.;Kehle-Forbes, S. M.;Larsen, S. E.; | 2024 | "Pitching" posttraumatic stress disorder treatment: A qualitative study of how providers discuss evidence-based psychotherapies with patients | Journal of Traumatic Stress | Mental health disorders |
| 2388 | Axame, W. K.;Kpodo, L.;Bilabam, J. K.;Assiam, R.;Kugbey, N.;Gbogbo, S.; | 2024 | "I should kill myself and rest in peace": a qualitative study on suicide attempts among adolescents in Ghana. | BMC Psychiatry | Mental health disorders |
| 2389 | Higgins, M.;Littleton, H.;Zamundu, A.;Dolezal, M.; | 2024 | "It Makes Me Feel Harder to Kill": A Qualitative Study of the Perceived Benefits of Recreational Martial Arts Practice Among Women Sexual Assault Survivors | Sex Roles | Mental health disorders |
| 2390 | Zolli, N.;Loubeau, J. K.;Sikov, J.;Baul, T. D.;Hasan, S.;Rosen, K.;Buonocore, O.;Rabin, M.;Duncan, A.;Fortuna, L.;Borba, C. P. C.;Silverstein, M.;Spencer, A. E.; | 2024 | "If he doesn't buy in, it's a waste of time": Perspectives from diverse parents and adolescents on engaging children in ADHD treatment. | Child Psychiatry and Human Development | Mental health disorders |

| | | | | | |
|---|---|---|---|---|---|
| 2391 | Yang, Y. J.;Qiu, X. H.;Yang, X. X.;Qiao, Z. X.;Han, D.;Wang, K.;Liu, J. P.; | 2010 | [Study on the influencing factors of suicidal ideation among university students in Harbin] | Zhonghua Liu Xing Bing Xue Za Zhi | Published not from January 2010 to 31 March 2024 |
| 2392 | Yang, L.;Zhang, Z.;Sun, L.;Wu, H.;Sun, Y.; | 2014 | [Risk and risk factors of suicide attempt after first onset of suicide ideation: findings from medical students in grades 1 and 2] | Wei Sheng Yan Jiu | Unpublished journal article |
| 2393 | Xiao, R.;Du, J.; | 2023 | [Reliability and validity of the 6-item UCLA Loneliness Scale(ULS-6)for application in adults] | Nan Fang Yi Ke Da Xue Xue Bao | The population is not college students. |
| 2394 | Chen, J. P.;Wang, H.;Liu, L.; | 2013 | [Quality of life and related social support for men who have sex with men among university students in Chongqing, China] | Zhonghua Liu Xing Bing Xue Za Zhi | Unpublished journal article |
| 2395 | Wang, H.;Si, M. Y.;Su, X. Y.;Huang, Y. M.;Xiao, W. J.;Wang, W. J.;Gu, X. F.;Ma, L.;Li, J.;Zhang, S. K.;Ren, Z. F.;Qiao, Y. L.; | 2022 | [Mental Health Status and Its Influencing Factors among College Students during the Epidemic of Coronavirus Disease 2019 Multi-center Cross-sectional Study] | Zhongguo Yi Xue Ke Xue Yuan Xue Bao | Unpublished journal article |
| 2396 | Kim, H. K.;Lee, M. H.; | 2011 | [Factors influencing resilience of adult children of alcoholics among college students] | J Korean Acad Nurs | The population is not college students |
| 2397 | Kreider, C. M.;Medina, S.;Koedam, H. M.; | 2021 | (Dis)ability-informed mentors support occupational performance for college students with learning disabilities and attention-deficit/hyperactivity disorders through problem-solving and a focus on strengths | British Journal of Occupational Therapy | Mental health disorders |
| 2398 | Suárez-Orozco, C.;López Hernández, G.; | 2020 | "Waking Up Every Day With the Worry": A Mixed-Methods Study of Anxiety in Undocumented Latinx College Students | Front Psychiatry | Mental health disorders |
| 2399 | Chang, P. F.;Whitlock, J.;Bazarova, | 2018 | "To Respond or not to Respond, that is the | Social Media + | Mental health disorders |

| | | | | | |
|---|---|---|---|---|---|
| | N. N.; | | Question": The Decision-Making Process of Providing Social Support to Distressed Posters on Facebook. | Society | |
| 2400 | Nguyen, R. L.;Cope, C. E.;Wiedbusch, E. K.;Guerrero, M.;Jason, L. A.; | 2021 | "This Program Helped Save Our Lives so We All Can Bond over That": A Preliminary Study of the First Oxford House Collegiate Recovery Home | Alcohol Treat Q | Unpublished journal article |
| 2401 | Oh, Christine J.;Kim, Nadia Y.; | 2016 | "Success Is Relative": Comparative Social Class and Ethnic Effects in an Academic Paradox | Sociological perspectives | Not related wellbeing |
| 2402 | Hill, L. G.;Bumpus, M.;Haggerty, K. P.;Catalano, R. F.;Cooper, B. R.;Skinner, M. L.; | 2023 | "Letting Go and Staying Connected": substance Use Outcomes from a Developmentally Targeted Intervention for Parents of College Students. | Prevention science | Mental health disorders |
| 2403 | Holland, K. J.;Cortina, L. M.; | 2017 | "It Happens to Girls All the Time": Examining Sexual Assault Survivors' Reasons for Not Using Campus Supports | Am J Community Psychol | Mental health disorders |
| 2404 | Gillespie-Lynch, K.;Bublitz, D.;Donachie, A.;Wong, V.;Brooks, P. J.;D'Onofrio, J.; | 2017 | "For a Long Time Our Voices Have been Hushed": Using Student Perspectives to Develop Supports for Neurodiverse College Students. | Front Psychol | Not related wellbeing |
| 2405 | Hoyle, J. N.;Laditka, J. N.;Laditka, S. B.; | 2022 | "Eventually I'm gonna need people": Social capital among college students with developmental disability. | Res Dev Disabil | Not related wellbeing |
| 2406 | Enriquez, Laura E.; | 2011 | "Because We Feel the Pressure and We Also Feel the Support": Examining the Educational Success of Undocumented Immigrant Latina/o Students | Harvard Educational Review | Not related wellbeing |
| 2407 | Jones, Phil; | 2021 | Work without the worker: Labour in the age of platform capitalism | 未分配 | Unpublished journal article |

| | | | | | |
|---|---|---|---|---|---|
| 2408 | Liberati, A.;Altman, D. G.;Tetzlaff, J.;Mulrow, C.;Gotzsche, P. C.;Ioannidis, J. P. A.;Clarke, M.;Devereaux, P. J.;Kleijnen, J.;Moher, D.; | 2009 | The PRISMA statement for reporting systematic reviews and meta-analyses of studies that evaluate healthcare interventions: explanation and elaboration | Bmj-British Medical Journal | Published not from January 2010 to 31 March 2024 |
| 2409 | Zhao, H.;Seibert, S. E.;Hills, G. E.; | 2005 | The mediating role of self-efficacy in the development of entrepreneurial intentions | Journal of Applied Psychology | Published not from January 2010 to 31 March 2024 |
| 2410 | Li, P.;Yang, J.;Zhou, Z.;Zhao, Z.;Liu, T.; | 2022 | The influence of college students' academic stressors on mental health during COVID-19: The mediating effect of social support, social well-being, and self-identity | Frontiers in Public Health | Mental health disorders |
| 2411 | Iram, A.;Mustafa, M.;Ahmad, S.;Maqsood, S.;Maqsood, F.; | 2021 | The Effects of Provision of Instrumental, Emotional, and Informational Support on Psychosocial Adjustment of Involuntary Childless Women in Pakistan | Journal of Family Issues | The population is not college students |
| 2412 | Hofstadler, N.;Babic, S.;Lämmerer, A.;Mercer, S.;Oberdorfer, P.; | 2021 | The ecology of CLIL teachers in Austria - an ecological perspective on CLIL teachers' wellbeing | Innovation in Language Learning and Teaching | Not related to research questions |
| 2413 | Wang, C.;Hatzigianni, M.;Shahaeian, A.;Murray, E.;Harrison, L. J.; | 2016 | The combined effects of teacher-child and peer relationships on children's social-emotional adjustment | Journal of School Psychology | The population is not college students |
| 2414 | Diener, E.;Suh, E. M.;Lucas, R. E.;Smith, H. L.; | 1999 | Subjective well-being: Three decades of progress | Psychological Bulletin | Published not from January 2010 to 31 March 2024 |
| 2415 | Diener, E.; | 1984 | Subjective wellbeing | Psychological Bulletin | Published not from January 2010 to 31 March 2024 |
| 2416 | Beaumont, J.;Putwain, D. | 2023 | Students' Emotion Regulation and School-Related | Journal of | Not related wellbeing |

| | | | | | |
|---|---|---|---|---|---|
| | W.;Gallard, D.;Malone, E.;Marsh, H. W.;Pekrun, R.; | | Well-Being: Longitudinal Models Juxtaposing Between- and Within-Person Perspectives | Educational Psychology | |
| 2417 | Strzelecki, A.; | 2024 | Students' Acceptance of ChatGPT in Higher Education: An Extended Unified Theory of Acceptance and Use of Technology | Innovative Higher Education | Not related wellbeing |
| 2418 | Cohen, S.;Wills, T. A.; | 1985 | Stress, social support, and the buffering hypothesis | Psychological Bulletin | Published not from January 2010 to 31 March 2024 |
| 2419 | Preacher, Kristopher J;Hayes, Andrew F; | 2004 | SPSS and SAS procedures for estimating indirect effects in simple mediation models | Behavior research methods, instruments, & computers | Published not from January 2010 to 31 March 2024 |
| 2420 | Chen, Y. X.;Feeley, T. H.; | 2014 | Social support, social strain, loneliness, and well-being among older adults: An analysis of the Health and Retirement Study | Journal of Social and Personal Relationships | The population is not college students |
| 2421 | Cheung, H. S.;Sim, T. N.; | 2017 | Social Support From Parents and Friends for Chinese Adolescents in Singapore | Youth & Society | Not related wellbeing |
| 2422 | Väisänen, S.;Pietarinen, J.;Pyhältö, K.;Toom, A.;Soini, T.; | 2017 | Social support as a contributor to student teachers' experienced well-being | Research Papers in Education | Not related wellbeing |
| 2423 | Yıldırım, Murat;Green, Zane Asher; | 2023 | Social support and resilience mediate the relationship of stress with satisfaction with life and the flourishing of youth. | British Journal of Guidance & Counselling | Mental health disorders |
| 2424 | Shin, H.;Park, C.; | 2022 | Social support and psychological well-being in younger and older adults: The mediating effects of basic psychological need satisfaction | Frontiers in Psychology | The population is not college students |
| 2425 | Bandura, A.; | 2001 | Social cognitive theory: An agentic perspective | Annual Review of Psychology | Published not from January 2010 to 31 March 2024 |

| | | | | | |
|---|---|---|---|---|---|
| 2426 | Kalaitzaki, A.;Tsouvelas, G.;Koukouli, S.; | 2021 | Social capital, social support and perceived stress in college students: The role of resilience and life satisfaction | Stress and Health | Mental health disorders |
| 2427 | Bandura, A.; | 1982 | Self-efficacy mechanism in human agency | American Psychologist | Published not from January 2010 to 31 March 2024 |
| 2428 | Lent, R. W.;Brown, S. D.;Larkin, K. C.; | 1986 | Self-efficacy in the prediction of academic performance and perceived career options | Journal of Counseling Psychology | Published not from January 2010 to 31 March 2024 |
| 2429 | Bandura, A.;Barbaranelli, C.;Caprara, G. V.;Pastorelli, C.; | 2001 | Self-efficacy beliefs as shapers of children's aspirations and career trajectories | Child Development | Published not from January 2010 to 31 March 2024 |
| 2430 | Bandura, A.; | 1977 | Self-efficacy - toward a unifying theory of behavioral change | Psychological Review | Published not from January 2010 to 31 March 2024 |
| 2431 | Li, Yue;Peng, Jun;Tao, Yanqiang; | 2023 | Relationship between social support, coping strategy against COVID-19, and anxiety among home-quarantined Chinese university students: A path analysis modeling approach | Current Psychology: A Journal for Diverse Perspectives on Diverse Psychological Issues | Mental health disorders |
| 2432 | Hayes, A. F.;Rockwood, N. J.; | 2017 | Regression-based statistical mediation and moderation analysis in clinical research: Observations, recommendations, and implementation | Behavior Research and Therapy | Mental health disorders |
| 2433 | Li, Ruihua;Che Hassan, Norlizah;Saharuddin, Norzihani; | 2023 | Psychological capital related to academic outcomes among university students: a systematic literature review | Psychology Research and Behavior | Review article |

| | | | | | |
|---|---|---|---|---|---|
| | | | | Management | |
| 2434 | Pashak, T. J.;Handal, P. J.;Scales, P. C.; | 2018 | Protective Factors for the College Years: Establishing the Appropriateness of the Developmental Assets Model for Emerging Adults | Current Psychology | The population is not college students |
| 2435 | Kline, Rex B; | 2023 | Principles and practice of structural equation modeling | | Unpublished journal article |
| 2436 | Cobb, S.; | 1976 | Presidential Address-1976. Social support as a moderator of life stress | Psychosom Med | Published not from January 2010 to 31 March 2024 |
| 2437 | O'Connor, E.;Prebble, K.;Waterworth, S.; | 2024 | Organizational factors to optimize mental health nurses' well-being in the workplace: An integrative literature review | International Journal of Mental Health Nursing | Not related to research questions |
| 2438 | World Health Organization（WHO）; | | Mental health | | Unpublished journal article |
| 2439 | Shrout, P. E.;Bolger, N.; | 2002 | Mediation in experimental and nonexperimental studies: New procedures and recommendations | Psychological Methods | Published not from January 2010 to 31 March 2024 |
| 2440 | Krasnov, V.; | 1988 | Marxism - philosophy and economics - so well,t | International Journal on World Peace | Published not from January 2010 to 31 March 2024 |
| 2441 | Zhang, X. Q.;Wong, G. T. F.;Liu, C. H.;Hahm, H. C.;Chen, J. A.; | 2022 | International student stressors and mental health during the COVID-19 pandemic: a qualitative study | Journal of American College Health | Mental health disorders |
| 2442 | Waters, S. K.;Cross, D.;Shaw, T.; | 2010 | How important are school and interpersonal student characteristics in determining later adolescent school connectedness, by school sector? | Australian Journal of Education | Published not from January 2010 to 31 March 2024 |
| 2443 | Van Ryn, Michelle;Vinokur, | 1992 | How did it work? An examination of the | American Journal | Published not from January |

| | | | | | |
|---|---|---|---|---|---|
| | Amiram D; | | mechanisms through which an intervention for the unemployed promoted job-search behavior | of Community Psychology | 2010 to 31 March 2024 |
| 2444 | Richards, B. N.; | 2022 | Help-Seeking Behaviors as Cultural Capital: Cultural Guides and the Transition from High School to College among Low-Income First-Generation Students | Social Problems | The population is not college students |
| 2445 | Ryff, C. D.; | 1989 | HAPPINESS IS EVERYTHING, OR IS IT - EXPLORATIONS ON THE MEANING OF PSYCHOLOGICAL WELL-BEING | Journal of Personality and Social Psychology | Published not from January 2010 to 31 March 2024 |
| 2446 | Popay, Jennie;Roberts, Helen;Sowden, Amanda;Petticrew, Mark;Arai, Lisa;Rodgers, Mark;Britten, Nicky;Roen, Katrina;Duffy, Steven; | 2006 | Guidance on the conduct of narrative synthesis in systematic reviews | 未分配 | Published not from January 2010 to 31 March 2024 |
| 2447 | Yang, Kairong;Zhang, Linting;Li, Wenjie;Jia, Ning;Kong, Feng; | 2024 | Gratitude predicts well-being via resilience and social support in emerging adults: A daily diary study. | The Journal of Positive Psychology | The population is not college students |
| 2448 | Jenkins, Sharon Rae;Belanger, Aimee;Connally, Melissa Londoño;Boals, Adriel;Durón, Kelly M.; | 2013 | First-generation undergraduate students' social support, depression, and life satisfaction | Journal of College Counseling | Mental health disorders |
| 2449 | Fan, C.;Liu, S.; | 2022 | Exploring the associations among perceived Teacher emotional support, resilience, COVID-19 anxiety, and mental well-being: evidence from Chinese vocational college students | Curr Psychol | Mental health disorders |
| 2450 | Popat, A.;Tarrant, C.; | 2023 | Exploring adolescents' perspectives on social | Clinical Child | Review article |

| | | | | | |
|---|---|---|---|---|---|
| | | | media and mental health and well-being - A qualitative literature review | Psychology and Psychiatry | |
| 2451 | Dittmann, C.;Forstmeier, S.; | 2022 | Experiences with Teachers in Childhood and their Association with wellbeing in adulthood | BMC Psychology | The population is not college students |
| 2452 | Scott, A. B.;Ciani, K. D.; | 2008 | Effects of an undergraduate career class on men's and women's career decision-making self-efficacy and vocational identity | Journal of Career Development | Published not from January 2010 to 31 March 2024 |
| 2453 | Lee, Geon Min;Shin, Hyun Kyun; | 2022 | Effects of Acceptance and Commitment Therapy on Job-Seeking Stress, Self-Efficacy, Experiential Avoidance, Depression, and Anxiety | Korean Journal of Stress Research | Mental health disorders |
| 2454 | Richards, M.;Huppert, F. A.; | 2011 | Do positive children become positive adults? Evidence from a longitudinal birth cohort study | Journal of Positive Psychology | The population is not college students |
| 2455 | Ross, L. T.;Ross, T. P.; | 2023 | Distress, Social Support, and Self-Compassion: Relationships With Mental Health Among College Students | Psychological Reports | Mental health disorders |
| 2456 | Roaming, Sinjin;Howard, Krista; | 2019 | Coping with stress in college: An examination of spirituality, social support, and quality of life | Mental Health, Religion & Culture | Mental health disorders |
| 2457 | Schiavo, G.;Businaro, S.;Zancanaro, M.; | 2024 | Comprehension, apprehension, and acceptance: Understanding the influence of literacy and anxiety on acceptance of artificial Intelligence | Technology in Society | Mental health disorders |
| 2458 | Waters, L.;Cameron, K.;Nelson-Coffey, S. K.;Crone, D. L.;Kern, M. L.;Lomas, T.;Oades, L.;Owens, R. L.;Pawelski, J. O.;Rashid, T.;Warren, M. A.;White, M. A.;Williams, P.; | 2022 | Collective well-being and posttraumatic growth during COVID-19: how positive psychology can help families, schools, workplaces and marginalized communities | Journal of Positive Psychology | Not related to research questions |

| | | | | | |
|---|---|---|---|---|---|
| 2459 | Lent, R. W.;Hackett, G.; | 1987 | Career self-efficacy - empirical status and future directions | Journal of Vocational Behavior | Published not from January 2010 to 31 March 2024 |
| 2460 | Baigi, S. F. M.;Sarbaz, M.;Ghaddaripouri, K.;Ghaddaripouri, M.;Mousavi, A. S.;Kimiafar, K.; | 2023 | Attitudes, knowledge, and skills towards artificial intelligence among healthcare students: A systematic review | Health Science Reports | Review article |
| 2461 | Ghotbi, N.;Ho, M. T.;Mantello, P.; | 2022 | The attitude of college students towards ethical issues of artificial intelligence in an international university in Japan | Ai & Society | Unpublished journal article |
| 2462 | Sindermann, C.;Sha, P.;Zhou, M.;Wernicke, J.;Schmitt, H. S.;Li, M.;Sariyska, R.;Stavrou, M.;Becker, B.;Montag, C.; | 2021 | Assessing the Attitude Towards Artificial Intelligence: Introduction of a Short Measure in German, Chinese, and English Language | Kunstliche Intelligenz | Unpublished journal article |
| 2463 | Guo, Kun;Zhang, Xiaoye;Bai, Simin;Minhat, Halimatus Sakdiah;Mohd Nazan, Ahmad Iqmer Nashriq;Feng, Jianan;Li, Xiuqin;Luo, Guihua;Zhang, Xiaoping;Feng, Jujun;Li, Yingbo;Si, Mingyu;Qiao, Youlin;Ouyang, Jing;Saliluddin, Suhainizam; | 2021 | Assessing Social support Impact on depression, anxiety, and Stress among Undergraduate students in Shaanxi Province during the COVID-19 pandemic in China | PLoS ONE | Mental health disorders |
| 2464 | Pandya, S. S.;Wang, J.; | 2024 | Artificial intelligence in career development: a scoping review | Human Resource Development International | Review article |
| 2465 | George, A Shaji; | 2024 | Artificial Intelligence and the Future of Work: Job | Partners Universal | The population is not college |

| | | | | | |
|---|---|---|---|---|---|
| | | | Shifting Not Job Loss | Innovative Research Publication | students |
| 2466 | Dwivedi, Y. K.;Hughes, L.;Ismagilova, E.;Aarts, G.;Coombs, C.;Crick, T.;Duan, Y. Q.;Dwivedi, R.;Edwards, J.;Eirug, A.;Galanos, V.;Ilavarasan, P. V.;Janssen, M.;Jones, P.;Kar, A. K.;Kizgin, H.;Kronemann, B.;Lal, B.;Lucini, B.;Medaglia, R.;Le Meunier-FitzHug | 2021 | Artificial Intelligence (AI): Multidisciplinary perspectives on emerging challenges, opportunities, and agenda for research, practice and policy | International Journal of Information Management | The population is not college students |
| 2467 | Lin, H. Z.;Chen, Q.; | 2024 | Artificial intelligence (AI) -Integrated Educational Applications and College students' creativity and academic emotions: students and teachers' perceptions and attitudes | BMC Psychology | The population is not college students |
| 2468 | Collier, Joel; | 2020 | Applied structural equation modeling using AMOS: Basic to advanced techniques | 未分配 | Unpublished journal article |
| 2469 | Taylor, K. M.;Betz, N. E.; | 1983 | Applications of self-efficacy theory to the understanding and treatment of career indecision | Journal of Vocational Behavior | Published not from January 2010 to 31 March 2024 |
| 2470 | Deer, LillyBelle K.;Gohn, Kelsey;Kanaya, Tomoe; | 2018 | Anxiety and Self-Efficacy as Sequential Mediators in US College Students’ Career Preparation | Education + Training | Mental health disorders |
| 2471 | Belle, M. A.;Antwi, C. O.;Ntim, S. Y.;Affum-Osei, E.;Ren, J.; | 2022 | Am I Gonna Get a Job? Graduating Students' Psychological Capital, Coping Styles, and Employment Anxiety | Journal of Career Development | Mental health disorders |
| 2472 | Pisarik, C. T.;Rowell, P. | 2017 | A Phenomenological Study of Career Anxiety | Career | Mental health disorders |

| | | | | | |
|---|---|---|---|---|---|
| | C.;Thompson, L. K.; | | Among College Students | Development Quarterly | |
| 2473 | Martins, A.;Ramalho, N.;Morin, E.; | 2010 | A comprehensive meta-analysis of the relationship between Emotional Intelligence and health | Personality and Individual Differences | Published not from January 2010 to 31 March 2024 |
| 2474 | Voss, C.;Shorter, P.;Weatrowski, G.;Mueller-Coyne, J.;Turner, K.; | 2023 | A comparison of anxiety levels before and during the COVID-19 pandemic | Psychological Reports | Mental health disorders |
| 2475 | Mortenson, S.;Liu, M. N.;Burleson, B. R.;Liu, Y.; | 2006 | A fluency of feeling - Exploring cultural and individual differences (and similarities) related to skilled emotional support | Journal of Cross-Cultural Psychology | Published not from January 2010 to 31 March 2024 |
| 2476 | Lindenberg, C. S.;Strickland, O.;Solorzano, R.;Galvis, C.;Dreher, M.;Darrow, V. C.; | 1999 | Correlates of alcohol and drug use among low-income Hispanic immigrant childbearing women living in the USA | International Journal of Nursing Studies | Published not from January 2010 to 31 March 2024 |
| 2477 | Goldstein, L. H.;Atkins, L.;Leigh, P. N.; | 2002 | Correlates of quality of life in people with motor neuron disease (MND) | Amyotrophic Lateral Sclerosis | Published not from January 2010 to 31 March 2024 |
| 2478 | Llácer, A.;Zunzunegui, M. V.;Gutierrez-Cuadra, P.;Béland, F.;Zarit, S. H.; | 2002 | Correlates of the well-being of spousal and children carers of disabled people over 65 in Spain | European Journal of Public Health | Published not from January 2010 to 31 March 2024 |
| 2479 | Kingsley, J.;Townsend, M.;Henderson-Wilson, C.; | 2009 | Cultivating health and wellbeing: members' perceptions of the health benefits of a Port Melbourne community garden. | Leisure Studies | Published not from January 2010 to 31 March 2024 |
| 2480 | Morgan, K.;Bath, P. A.; | 1998 | Customary physical activity and psychological well-being: a longitudinal study | Age and Ageing | Published not from January 2010 to 31 March 2024 |
| 2481 | Kanis, M.;Brinkman, W. P.;Perry, M.; | 2009 | Designing for positive disclosure: What do you like today? | International Journal of Industrial Ergonomics | Published not from January 2010 to 31 March 2024 |

| | | | | | |
|---|---|---|---|---|---|
| 2482 | Hodgson, N. A.;Given, C. W.; | 2004 | Determinants of functional recovery in older adults surgically treated for cancer. | Cancer Nursing | Published not from January 2010 to 31 March 2024 |
| 2483 | Cummins, R. A.;Eckersley, R.;Pallant, J.;Van Vugt, J.;Misajon, R.; | 2003 | Developing a national index of subjective wellbeing: The Australian Unity Wellbeing Index | Social Indicators Research | Published not from January 2010 to 31 March 2024 |
| 2484 | Katz, M. R.;Irish, J. C.;Devins, G. M.; | 2004 | Development and pilot testing of a psychoeducational intervention for oral cancer patients | Psycho-Oncology | Published not from January 2010 to 31 March 2024 |
| 2485 | Ghahari, S.;Packer, T. L.;Passmore, A. E.; | 2009 | Development, standardization, and pilot testing of an online fatigue self-management program | Disability and Rehabilitation | Published not from January 2010 to 31 March 2024 |
| 2486 | Derluyn, I.;Broekaert, E.; | 2007 | Different perspectives on emotional and behavioral problems in unaccompanied refugee children and adolescents | Ethnicity & Health | Published not from January 2010 to 31 March 2024 |
| 2487 | Ashida, S.;Heaney, C. A.; | 2008 | Differential associations of social support and social connectedness with structural features of social networks and the health status of older adults | Journal of Aging and Health | Published not from January 2010 to 31 March 2024 |
| 2488 | Simon, A. E.;Thompson, M. R.;Flashman, K.;Wardle, J.; | 2009 | Disease stage and psychosocial outcomes in colorectal cancer | Colorectal Disease | Published not from January 2010 to 31 March 2024 |
| 2489 | Meadows, G.;Burgess, P.;Bobevski, I.; | 2002 | Distributing mental health care resources: strategic implications from the National Survey of Mental Health and Wellbeing | Australian and New Zealand Journal of Psychiatry | Published not from January 2010 to 31 March 2024 |
| 2490 | Clémence, A.;Karmaniola, A.;Green, E. G. T.;Spini, D.; | 2007 | Disturbing life events and well-being after 80 years of age:: a longitudinal comparison of survivors and the deceased over five years | Ageing & Society | Published not from January 2010 to 31 March 2024 |

| | | | | | |
|---|---|---|---|---|---|
| 2491 | Martin, M.;Westerhof, G. J.; | 2003 | Do you need to have them or should you believe you have them? Resources, their appraisal, and well-being in adulthood | Journal of Adult Development | Published not from January 2010 to 31 March 2024 |
| 2492 | Montorio, I.;Losada, A.;Izal, M.;Márquez, O.; | 2009 | Dysfunctional Thoughts about Caregiving Questionnaire: psychometric properties of a new measure | International Psychogeriatrics | Published not from January 2010 to 31 March 2024 |
| 2493 | Lee, Y.; | 2009 | Early motherhood and harsh parenting: The role of human, social, and cultural capital | Child Abuse & Neglect | Published not from January 2010 to 31 March 2024 |
| 2494 | Knapp, M.;Romeo, R.;Beecham, J.; | 2009 | The economic cost of autism in the UK | Autism | Published not from January 2010 to 31 March 2024 |
| 2495 | Mistry, R. S.;Vandewater, E. A.;Huston, A. C.;McLoyd, V. C.; | 2002 | Economic well-being and children's social adjustment: The role of family process in an ethnically diverse low-income sample | Child Development | Published not from January 2010 to 31 March 2024 |
| 2496 | Jones, R. B.;Pearson, J.;Cawsey, A. J.;Bental, D.;Barrett, A.;White, J.;White, C. A.;Gilmour, W. H.; | 2006 | Effect of different forms of information produced for cancer patients on their use of the information, social support, and anxiety: randomized trial | British Medical Journal | Published not from January 2010 to 31 March 2024 |
| 2497 | Kocken, P. L.;Voorham, A. J. J.; | 1998 | Effects of a peer-led senior health education program | Patient Education and Counseling | Published not from January 2010 to 31 March 2024 |
| 2498 | Schultz, J.;Corman, H.;Noonan, K.;Reichman, N. E.; | 2009 | Effects of child health on parents' social capital | Social Science & Medicine | Published not from January 2010 to 31 March 2024 |
| 2499 | Langer, A.;Campero, L.;Garcia, C.;Reynoso, S.; | 1998 | Effects of psychosocial support during labor and childbirth on breastfeeding, medical interventions, and mothers' wellbeing in a Mexican public hospital: a randomized clinical trial | British Journal of Obstetrics and Gynaecology | Published not from January 2010 to 31 March 2024 |
| 2500 | Samarel, N.;Tulman, L.;Fawcett, J.; | 2002 | Effects of two Types of Social Support and Education on adaptation to early-stage breast | Research in Nursing & Health | Published not from January 2010 to 31 March 2024 |

| | | | | | |
|---|---|---|---|---|---|
| | | | cancer | | |
| 2501 | Strongman, K. T.;Overton, A. E.; | 1999 | Emotion in late adulthood | Australian Psychologist | Published not from January 2010 to 31 March 2024 |
| 2502 | Wallis, H.;Renneberg, B.;Ripper, S.;Germann, G.;Wind, G.;Jester, A.; | 2006 | Emotional distress and psychosocial resources in patients recovering from severe burn injury | Journal of Burn Care & Research | Published not from January 2010 to 31 March 2024 |
| 2503 | Burton, N. W.;Pakenham, K. I.;Brown, W. J.; | 2009 | Evaluating the effectiveness of psychosocial resilience training for heart health, and the added value of promoting physical activity: a cluster randomized trial of the READY program | Bmc Public Health | Published not from January 2010 to 31 March 2024 |
| 2504 | Ashing-Giwa, K. T.;Tejero, J. S.;Kim, J.;Padilla, G. V.;Hellemann, G.; | 2007 | Examining predictive models of HRQOL in a population-based, multiethnic sample of women with breast carcinoma | Quality of Life Research | Published not from January 2010 to 31 March 2024 |
| 2505 | Lee, C.; | 2001 | Experiences of family caregiving among older Australian women | Journal of Health Psychology | Published not from January 2010 to 31 March 2024 |
| 2506 | Stansfeld, S. A.;Head, J.;Marmot, M. G.; | 1998 | Explaining social class differences in depression and well-being | Social Psychiatry and Psychiatric Epidemiology | Published not from January 2010 to 31 March 2024 |
| 2507 | Cant, B.; | 2004 | Facilitating social networks among gay men | Sociological Research Online | Published not from January 2010 to 31 March 2024 |
| 2508 | Mikolajczyk, R. T.;Brzoska, P.;Maier, C.;Ottova, V.;Meier, S.;Dudziak, U.;Ilieva, S.;El Ansari, W.; | 2008 | Factors associated with self-rated health status in university students: a cross-sectional study in three European countries | Bmc Public Health | Published not from January 2010 to 31 March 2024 |
| 2509 | Ferrell, B.;Ervin, K.;Smith, S.;Marek, T.;Melancon, C.; | 2002 | Family perspectives on ovarian cancer | Cancer Practice | Published not from January 2010 to 31 March 2024 |

| | | | | | |
|---|---|---|---|---|---|
| 2510 | Meadows, S. O.; | 2009 | Family Structure and Fathers' Well-Being: Trajectories of Mental Health and Self-Rated Health | Journal of Health and Social Behavior | Published not from January 2010 to 31 March 2024 |
| 2511 | Decker, S.;Cary, P.;Krautscheid, L.; | 2006 | From the streets to assisted living: Perceptions of a vulnerable population | Journal of Psychosocial Nursing and Mental Health Services | Published not from January 2010 to 31 March 2024 |
| 2512 | Lai, T. J.;Chang, C. M.;Connor, K. M.;Lee, L. C.;Davidson, J. R. T.; | 2004 | Full and partial PTSD among earthquake survivors in rural Taiwan | Journal of Psychiatric Research | Published not from January 2010 to 31 March 2024 |
| 2513 | O'Hare, T.;Sherrer, M. V.;Connery, H. S.;Thornton, J.;LaButti, A.;Emrick, K.; | 2003 | Further validation of the Psycho-Social Well-Being Scale (PSWS) with community clients | Community Mental Health Journal | Published not from January 2010 to 31 March 2024 |
| 2514 | Sobieszczyk, T.;Knodel, J.;Chayovan, N.; | 2003 | Gender and wellbeing among older people: evidence from Thailand | Aging and Society | Published not from January 2010 to 31 March 2024 |
| 2515 | Fuller, T. D.;Edwards, J. N.;Vorakitphokatorn, S.;Sermsri, S.; | 2004 | Gender differences in the psychological well-being of married men and women: An Asian case | Sociological Quarterly | Published not from January 2010 to 31 March 2024 |
| 2516 | Friel, S.;Marmot, M.;McMichael, A. J.;Kjellstrom, T.;Vågerö, D.; | 2008 | Global health equity and climate stabilization: a common agenda | Lancet | Published not from January 2010 to 31 March 2024 |
| 2517 | Mikelson, K. S.; | 2008 | He said she said: Comparing mother and father reports of father involvement. | Journal of Marriage and Family | Published not from January 2010 to 31 March 2024 |
| 2518 | Lau, R.;Morse, C. A.; | 2008 | Health and well-being of older people in Anglo-Australian and Italian-Australian communities: A rural-urban comparison | Australian Journal of Rural Health | Published not from January 2010 to 31 March 2024 |
| 2519 | Schofield, H. L.;Bloch, S.;Nankervis, J.;Murphy, B.;Singh, B. S.;Herrman, | 1999 | Health and well-being of women family carers: a comparative study with a generic focus | Australian and New Zealand Journal of | Published not from January 2010 to 31 March 2024 |

| | | | | | |
|---|---|---|---|---|---|
| | H. E.; | | | Public Health | |
| 2520 | Batniji, R.;Rabaia, Y.;Nguyen-Gillham, V.;Giacaman, R.;Sarraj, E.;Punamaki, R. L.;Saab, H.;Boyce, W.; | 2009 | Health in the Occupied Palestinian Territory 4 Health as human security in the occupied Palestinian territory | Lancet | Published not from January 2010 to 31 March 2024 |
| 2521 | Parkerson, G. R.;Gutman, R. A.; | 2000 | Health-related quality of life predictors of survival and hospital utilization | Health Care Financing Review | Published not from January 2010 to 31 March 2024 |
| 2522 | Fertig, A. R.;Reingold, D. A.; | 2008 | Homelessness among At-Risk Families with Children in Twenty American Cities | Social Service Review | Published not from January 2010 to 31 March 2024 |
| 2523 | Butterworth, P.;Fairweather, A. K.;Anstey, K. J.;Windsor, T. D.; | 2006 | Hopelessness, demoralization, and suicidal behavior: the backdrop to welfare reform in Australia | Australian and New Zealand Journal of Psychiatry | Published not from January 2010 to 31 March 2024 |
| 2524 | Mamdani, M.;Rajani, R.;Leach, V.; | 2008 | How Best to Enable Support for Children Affected by HIV/AIDS? A Policy Case Study in Tanzania | Ids Bulletin-Institute of Development Studies | Published not from January 2010 to 31 March 2024 |
| 2525 | Blazer, D. G.; | 2008 | How do you feel about ...? Health Outcomes in late life and self-perceptions of health and well-being | Gerontologist | Published not from January 2010 to 31 March 2024 |
| 2526 | Juth, V.;Smyth, J. M.;Santuzzi, A. M.; | 2008 | How do you feel? - Self-esteem predicts effect, stress, social interaction, and symptom severity during daily life in patients with chronic illness | Journal of Health Psychology | Published not from January 2010 to 31 March 2024 |
| 2527 | Bray, R.; | 2009 | How does AIDS illness affect women's residential decisions? Findings from an ethnographic study in a Cape Town township | Ajar-African Journal of Aids Research | Published not from January 2010 to 31 March 2024 |

| | | | | | |
|---|---|---|---|---|---|
| 2528 | Carver, C. S.;Harris, S. D.;Lehman, J. M.;Durel, L. A.;Antoni, M. H.;Spencer, S. M.;Pozo-Kaderman, C.; | 2000 | How important is the perception of personal control? Studies of early-stage breast cancer patients | Personality and Social Psychology Bulletin | Published not from January 2010 to 31 March 2024 |
| 2529 | Salewski, C.; | 2003 | Illness Representations in families with a chronically ill adolescent: Differences between family members and impact on patients' outcome variables | Journal of Health Psychology | Published not from January 2010 to 31 March 2024 |
| 2530 | Ghasemi, M.; | 2009 | Impact of Domestic Violence on the psychological wellbeing of children in Iran | Journal of Family Studies | Published not from January 2010 to 31 March 2024 |
| 2531 | Wallhagen, M. I.;Strawbridge, W. J.;Shema, S. J.;Kaplan, G. A.; | 2004 | Impact of self-assessed hearing loss on a spouse: A longitudinal analysis of couples | Journals of Gerontology Series B-Psychological Sciences and Social Sciences | Published not from January 2010 to 31 March 2024 |
| 2532 | Afifi, T. D.;McManus, T.;Hutchinson, S.;Baker, B.; | 2007 | Inappropriate parental divorce disclosures, the factors that prompt them, and their impact on parents' and adolescents' well-being | Communication Monographs | Published not from January 2010 to 31 March 2024 |
| 2533 | Kärner, A.;Tingström, P.;Abrandt-Dahlgren, M.;Bergdahl, B.; | 2005 | Incentives for lifestyle changes in patients with coronary heart disease | Journal of Advanced Nursing | Published not from January 2010 to 31 March 2024 |
| 2534 | Taris, T. W.;Van Horn, J. E.;Schaufeli, W. B.;Schreurs, P. J. G.; | 2004 | Inequity, burnout and psychological withdrawal among teachers: A dynamic exchange model | Anxiety Stress and Coping | Published not from January 2010 to 31 March 2024 |
| 2535 | Raina, P.;Waltner-Toews, D.;Bonnett, B.;Woodward, | 1999 | Influence of companion animals on the physical and psychological health of older people: An | Journal of the American Geriatrics | Published not from January 2010 to 31 March 2024 |

| | | | | | |
|---|---|---|---|---|---|
| | C.;Abernathy, T.; | | analysis of a one-year longitudinal study | Society | |
| 2536 | Orbell, S.; | 1996 | Informal care in social context: A social psychological analysis of participation, impact, and intervention in care of the elderly | Psychology & Health | Published not from January 2010 to 31 March 2024 |
| 2537 | Dodman, D.;Satterthwaite, D.; | 2008 | Institutional Capacity, Climate Change Adaptation, and the Urban Poor | Ids Bulletin-Institute of Development Studies | Published not from January 2010 to 31 March 2024 |
| 2538 | Strazdins, L. M.; | 2000 | Integrating emotions: Multiple role measurement of emotional work | Australian Journal of Psychology | Published not from January 2010 to 31 March 2024 |
| 2539 | Noblet, A. J.;Rodwell, J. J.; | 2009 | Integrating Job Stress and Social Exchange Theories to Predict Employee Strain in Reformed Public Sector Contexts | Journal of Public Administration Research and Theory | Published not from January 2010 to 31 March 2024 |
| 2540 | Sum, S.;Mathews, M. R.;Pourghasem, M.;Hughes, I.; | 2008 | Internet Technology and Social Capital: How the Internet Affects Seniors' Social Capital and Wellbeing | Journal of Computer-Mediated Communication | Published not from January 2010 to 31 March 2024 |
| 2541 | Mazalin, D.;Moore, S.; | 2004 | Internet use, identity development and social anxiety among young adults | Behaviour Change | Published not from January 2010 to 31 March 2024 |
| 2542 | Kerry, S.; | 2009 | Intersex individuals' religiosity and their journey to wellbeing | Journal of Gender Studies | Published not from January 2010 to 31 March 2024 |
| 2543 | Findlay, R. A.; | 2003 | Interventions to reduce social isolation amongst older people: where is the evidence? | Ageing & Society | Published not from January 2010 to 31 March 2024 |
| 2544 | Feldman, S.;Byles, J. E.;Beaumont, R.; | 2000 | 'Is anybody listening?' The experiences of widowhood for older Australian women | Journal of Women & Aging | Published not from January 2010 to 31 March 2024 |

| | | | | | |
|---|---|---|---|---|---|
| 2545 | Ridge, T.; | 2007 | It's a family affair: Low-income children's perspectives on maternal work | Journal of Social Policy | Published not from January 2010 to 31 March 2024 |
| 2546 | Robertson, S.; | 2006 | 'I've been like a coiled spring this last week': embodied masculinity and health. | Sociology of Health & Illness | Published not from January 2010 to 31 March 2024 |
| 2547 | Lindfors, P. M.;Meretoja, O. A.;Töyry, S. M.;Luukkonen, R. A.;Elovainio, M. J.;Leino, T. J.; | 2007 | Job Satisfaction, Work Ability, and life satisfaction among Finnish anaesthesiologists | Acta Anaesthesiologica Scandinavica | Published not from January 2010 to 31 March 2024 |
| 2548 | Radey, M.;Padilla, Y. C.; | 2009 | Kin Financial Support: Receipt and Provision Among Unmarried Mothers | Journal of Social Service Research | Published not from January 2010 to 31 March 2024 |
| 2549 | Sastre, M. T. M.; | 1999 | Lay conceptions of well-being and rules used in well-being judgments among young, middle-aged, and elderly adults | Social Indicators Research | Published not from January 2010 to 31 March 2024 |
| 2550 | Bowling, A.;Gabriel, Z.; | 2007 | Lay theories of quality of life in older age | Ageing & Society | Published not from January 2010 to 31 March 2024 |
| 2551 | Hipkins, R.; | 2005 | Learning to "be" in a new century: reflections on a curriculum in transition | Curriculum Matters | Published not from January 2010 to 31 March 2024 |
| 2552 | Short, L.; | 2007 | Lesbian mothers living well in the context of heterosexism and discrimination: Resources, strategies and legislative change | Feminism & Psychology | Published not from January 2010 to 31 March 2024 |
| 2553 | Biggs, S.;Bernard, M.;Kingston, P.;Nettleton, H.; | 2000 | Lifestyles of belief: narrative and culture in a retirement community | Aging and Society | Published not from January 2010 to 31 March 2024 |
| 2554 | Cohen-Mansfield, J.;Shmotkin, D.;Goldberg, S.; | 2009 | Loneliness in old age: longitudinal changes and their determinants in an Israeli sample | International Psychogeriatrics | Published not from January 2010 to 31 March 2024 |
| 2555 | Golden, J.;Conroy, R. M.;Bruce, I.;Denihan, A.;Greene, E.;Kirby, M.;Lawlor, B. A.; | 2009 | Loneliness, social support networks, mood and wellbeing in community-dwelling elderly | International Journal of Geriatric Psychiatry | Published not from January 2010 to 31 March 2024 |

| | | | | | |
|---|---|---|---|---|---|
| 2556 | Drory, Y.;Kravetz, S.;Hirschberger, G.; | 2002 | Long-term mental health of men after a first acute myocardial infarction | Archives of Physical Medicine and Rehabilitation | Published not from January 2010 to 31 March 2024 |
| 2557 | Michael, J. H.;Evans, D. D.;Jansen, K. J.;Haight, J. M.; | 2005 | Management commitment to safety as organizational support: Relationships with non-safety outcomes in wood manufacturing employees | Journal of Safety Research | Published not from January 2010 to 31 March 2024 |
| 2558 | Johnson, H.;Douglas, J.;Bigby, C.;Iacono, T.; | 2009 | Maximizing community inclusion through mainstream communication services for adults with severe disabilities | International Journal of Speech-Language Pathology | Published not from January 2010 to 31 March 2024 |
| 2559 | van Rensburg, M. S. J.; | 2009 | Measuring the quality of life of residents in SADC communities affected by HIV | Aids Care-Psychological and Socio-Medical Aspects of Aids/Hiv | Published not from January 2010 to 31 March 2024 |
| 2560 | Price, S. L.;Storey, S.;Lake, M.; | 2008 | Menopause experiences of women in rural areas | Journal of Advanced Nursing | Published not from January 2010 to 31 March 2024 |
| 2561 | Mak, A. S.;Nesdale, D.; | 2001 | Migrant Distress: The role of perceived racial discrimination and coping resources | Journal of Applied Social Psychology | Published not from January 2010 to 31 March 2024 |
| 2562 | Ballinger, M. L.;Talbot, L. A.;Verrinder, G. K.; | 2009 | More than a place to do woodwork: a case study of a community-based Men's Shed | Journal of Mens Health | Published not from January 2010 to 31 March 2024 |
| 2563 | Taft, A. J.;Small, R.;Hegarty, K. L.;Lumley, J.;Watson, L. F.;Gold, L.; | 2009 | MOSAIC (MOthers' Advocates In the Community): protocol and sample description of a cluster randomized trial of mentor-mother support to reduce intimate partner violence among pregnant or recent mothers | Bmc Public Health | Published not from January 2010 to 31 March 2024 |

| | | | | | |
|---|---|---|---|---|---|
| 2564 | Bromley, J.;Hare, D. J.;Davison, K.;Emerson, E.; | 2004 | Mothers supporting children with autistic spectrum disorders - Social support, mental health status, and satisfaction with services. | Autism | Published not from January 2010 to 31 March 2024 |
| 2565 | Sixsmith, A.;Gibson, G.; | 2007 | Music and the wellbeing of people with dementia | Ageing & Society | Published not from January 2010 to 31 March 2024 |
| 2566 | Buckley, C.;Angel, J. L.;Donahue, D.; | 2000 | Nativity and older women's health: Constructed reliance in the health and retirement study | Journal of Women & Aging | Published not from January 2010 to 31 March 2024 |
| 2567 | Mellor, D.;Stokes, M.;Firth, L.;Hayashi, Y.;Cummins, R.; | 2008 | Need for belonging, relationship satisfaction, loneliness, and life satisfaction. | Personality and Individual Differences | Published not from January 2010 to 31 March 2024 |
| 2568 | Van Groenou, M. I. B.;Van Tilburg, T.; | 2003 | Network size and support in old age: differentials by socio-economic status in childhood and adulthood | Aging and Society | Published not from January 2010 to 31 March 2024 |
| 2569 | Williams, B. R.;Baker, P. S.;Allman, R. M.; | 2005 | Nonspousal family loss among community-dwelling older adults | Omega-Journal of Death and Dying | Published not from January 2010 to 31 March 2024 |
| 2570 | Chen, Z. H.;Davey, G.; | 2008 | Normative Life Satisfaction in Chinese Societies | Social Indicators Research | Published not from January 2010 to 31 March 2024 |
| 2571 | Dobreva-Martinova, T.;Villeneuve, M.;Strickland, L.;Matheson, K.; | 2002 | Occupational role stress in the Canadian forces: Its association with individual and organizational well-being | Canadian Journal of Behavioural Science-Revue Canadienne Des Sciences Du Comportement | Published not from January 2010 to 31 March 2024 |
| 2572 | Snyder, L. A.;Krauss, A. D.;Chen, P. Y.;Finlinson, S.;Huang, Y. H.; | 2008 | Occupational safety: Application of the job demand-control-support model | Accident Analysis and Prevention | Published not from January 2010 to 31 March 2024 |

| | | | | | |
|---|---|---|---|---|---|
| 2573 | Wiles, J. L.;Allen, R. E. S.;Palmer, A. J.;Hayman, K. J.;Keeling, S.;Kerse, N.; | 2009 | Older people and their social spaces: A study of well-being and attachment to place in Aotearoa New Zealand | Social Science & Medicine | Published not from January 2010 to 31 March 2024 |
| 2574 | Furukawa, T. A.;Hori, S.;Azuma, H.;Nakano, Y.;Oshima, M.;Kitamura, T.;Sugiura, M. O.;Aoki, K.; | 2002 | Parents, personality, or partner? Correlates of marital relationships | Social Psychiatry and Psychiatric Epidemiology | Published not from January 2010 to 31 March 2024 |
| 2575 | Shek, D. I. L.; | 2005 | Paternal and Maternal influences on the Psychological well-being of Substance Abuse, and Delinquency of Chinese Adolescents Experiencing Economic Disadvantage | Journal of Clinical Psychology | Published not from January 2010 to 31 March 2024 |
| 2576 | Thornton, A. A.;Perez, M. A.;Meyerowitz, B. E.; | 2004 | Patient and partner quality of life and psychosocial adjustment following radical prostatectomy | Journal of Clinical Psychology in Medical Settings | Published not from January 2010 to 31 March 2024 |
| 2577 | Fleishman, J. A.;Sherbourne, C. D.;Cleary, P. D.;Wu, A. W.;Crystal, S.;Hays, R. D.; | 2003 | Patterns of coping among persons with HIV infection: Configurations, correlates, and change | American Journal of Community Psychology | Published not from January 2010 to 31 March 2024 |
| 2578 | Davey, A.;Eggebeen, D. J.; | 1998 | Patterns of intergenerational exchange and mental health | Journals of Gerontology Series B-Psychological Sciences and Social Sciences | Published not from January 2010 to 31 March 2024 |
| 2579 | Narushima, M.; | 2005 | 'Payback time': community volunteering among older adults as a transformative mechanism | Aging and Society | Published not from January 2010 to 31 March 2024 |
| 2580 | Fornara, F.;Bonaiuto, M.;Bonnes, M.; | 2006 | Perceived hospital environment quality indicators: A study of orthopedic | Journal of Environmental | Published not from January 2010 to 31 March 2024 |

| | | | | | |
|---|---|---|---|---|---|
| | | | | Psychology | |
| 2581 | Coventry, W. L.;Gillespie, N. A.;Heath, A. C.;Martin, N. G.; | 2004 | Perceived social support in a large community sample - Age and sex differences | Social Psychiatry and Psychiatric Epidemiology | Published not from January 2010 to 31 March 2024 |
| 2582 | Skok, A.;Harvey, D.;Reddihough, D.; | 2006 | Perceived stress, perceived social support, and well-being among mothers of school-aged children with cerebral palsy | Journal of Intellectual & Developmental Disability | Published not from January 2010 to 31 March 2024 |
| 2583 | Hatmaker, D. D.;Kemp, V. H.; | 1998 | Perception of threat and subjective well-being in low-risk and high-risk pregnant women | Journal of Perinatal & Neonatal Nursing | Published not from January 2010 to 31 March 2024 |
| 2584 | Chang, J. K.;Krantz, M.; | 1996 | Personal and environmental factors concerning adjustment of offspring of alcoholics | Substance Use & Misuse | Published not from January 2010 to 31 March 2024 |
| 2585 | O'Rourke, N.; | 2005 | Personality, cognitive adaptation, and marital satisfaction as predictors of well-being among older married adults | Canadian Journal on Aging-Revue Canadienne Du Vieillissement | Published not from January 2010 to 31 March 2024 |
| 2586 | Walker, R. B.;Hiller, J. E.; | 2007 | Places and health: A qualitative study to explore how older women living alone perceive the social and physical dimensions of their neighborhoods | Social Science & Medicine | Published not from January 2010 to 31 March 2024 |
| 2587 | Hatzidimitriadou, E.; | 2002 | Political ideology, helping mechanisms, and empowerment of mental health self-help/mutual aid groups | Journal of Community & Applied Social Psychology | Published not from January 2010 to 31 March 2024 |
| 2588 | Wang, X. D.;Gao, L.;Zhang, H. B.;Zhao, C. Z.;Shen, Y. C.;Shinfuku, N.; | 2000 | Post-earthquake quality of Life and psychological well-being: Longitudinal evaluation in a rural community sample in northern China | Psychiatry and Clinical Neurosciences | Published not from January 2010 to 31 March 2024 |

| | | | | | |
|---|---|---|---|---|---|
| 2589 | Caron, J.;Corbière, M.;Mercier, C.;Diaz, P.;Ricard, N.;Lesage, A.; | 2003 | The construct validity of the client questionnaire of the Wisconsin Quality of Life Index -: a cross-validation study | International Journal of Methods in Psychiatric Research | Published not from January 2010 to 31 March 2024 |
| 2590 | Miller, S. M.; | 2008 | The Effect of Frequency and Type of Internet Use on Perceived Social Support and Sense of Well-Being in Individuals With Spinal Cord Injury | Rehabilitation Counseling Bulletin | Published not from January 2010 to 31 March 2024 |
| 2591 | Iwasaki, Y.;Zuzanek, J.;Mannell, R. C.; | 2001 | The effects of physically active leisure on stress-health relationships | Canadian Journal of Public Health-Revue Canadienne De Sante Publique | Published not from January 2010 to 31 March 2024 |
| 2592 | Greaves, M.;Rogers-Clark, C.; | 2009 | The experience of socially isolated older people in accessing and navigating the healthcare system | Australian Journal of Advanced Nursing | Published not from January 2010 to 31 March 2024 |
| 2593 | Harknett, K.;Hardman, L.;Garfinkel, I.;McLanahan, S. S.; | 2001 | The fragile families study: Social policies and labor markets in seven cities. | Children and Youth Services Review | Published not from January 2010 to 31 March 2024 |
| 2594 | Vozoris, N. T.;Tarasuk, V. S.; | 2004 | The health of Canadians on welfare | Canadian Journal of Public Health-Revue Canadienne De Sante Publique | Published not from January 2010 to 31 March 2024 |
| 2595 | Janzen, B. L.;Green, K.;Muhajarine, N.; | 2006 | The health of single fathers - Demographic, economic and social correlates | Canadian Journal of Public Health-Revue Canadienne De | Published not from January 2010 to 31 March 2024 |

| | | | | | |
|---|---|---|---|---|---|
| | | | | Sante Publique | |
| 2596 | Beekman, A. T. F.;Penninx, Bwjh;Deeg, D. J. H.;de Beurs, E.;Geerlings, S. W.;van Tilburg, W.; | 2002 | The impact of depression on the well-being, disability, and use of services in older adults: a longitudinal perspective | Acta Psychiatrica Scandinavica | Published not from January 2010 to 31 March 2024 |
| 2597 | Beaudoin, C. E.; | 2007 | The impact of news use and social capital on youth wellbeing: An aggregate-level analysis | Journal of Community Psychology | Published not from January 2010 to 31 March 2024 |
| 2598 | Guo, M.;Aranda, M. P.;Silverstein, M.; | 2009 | The Impact of out-migration on the inter-generational support and psychological well-being of older adults in rural China | Ageing & Society | Published not from January 2010 to 31 March 2024 |
| 2599 | Theron, L. C.; | 2007 | The impact of the HIV epidemic on the composite well-being of educators in South Africa: a qualitative study | Ajar-African Journal of Aids Research | Published not from January 2010 to 31 March 2024 |
| 2600 | Feldman, B.;Broussard, C. A.; | 2005 | The Influence of Relational Factors on Men's adjustment to their partners' newly-diagnosed breast cancer | Journal of Psychosocial Oncology | Published not from January 2010 to 31 March 2024 |
| 2601 | Van der Doef, M.;Maes, S.; | 1999 | The Job Demand-Control(-Support) model and psychological well-being: a review of 20 years of empirical research | Work and Stress | Published not from January 2010 to 31 March 2024 |
| 2602 | Langer, A.;Farnot, U.;Garcia, C.;Barros, F.;Victora, C.;Belizan, J. M.;Villar, J.; | 1996 | The Latin American trial of psychosocial support during pregnancy: Effects on mother's well-being and satisfaction | Social Science & Medicine | Published not from January 2010 to 31 March 2024 |
| 2603 | van Wel, F.;Linssen, H.;Abma, R.; | 2000 | The parental bond and the well-being of adolescents and young adults | Journal of Youth and Adolescence | Published not from January 2010 to 31 March 2024 |
| 2604 | Butterworth, P.; | 2003 | The prevalence of mental disorders among income | Australian and New | Published not from January |

| | | | | | |
|---|---|---|---|---|---|
| | | | support recipients: An important issue for welfare reform | Zealand Journal of Public Health | 2010 to 31 March 2024 |
| 2605 | Cook, K.;Davis, E.;Smyth, P.;McKenzie, H.; | 2009 | The Quality of Life of Single Mothers Making the Transition from Welfare to Work | Women & Health | Published not from January 2010 to 31 March 2024 |
| 2606 | Goldner, L.;Mayseless, O.; | 2009 | The Quality of Mentoring Relationships and Mentoring Success | Journal of Youth and Adolescence | Published not from January 2010 to 31 March 2024 |
| 2607 | Tuncay, T.;Musabak, I.;Gok, D. E.;Kutlu, M.; | 2008 | The relationship between anxiety, coping strategies, and characteristics of patients with diabetes | Health and Quality of Life Outcomes | Published not from January 2010 to 31 March 2024 |
| 2608 | Suurmeijer, Tpbm;Van Sonderen, F. L. P.;Krol, B.;Doeglas, D. M.;Van den Heuvel, W. J. A.;Sanderman, R.; | 2005 | The relationship between personality, supportive transactions and support satisfaction, and mental health of patients with early rheumatoid arthritis. Results from the Dutch part of the Euridiss study | Social Indicators Research | Published not from January 2010 to 31 March 2024 |
| 2609 | Zank, S.;Leipold, B.; | 2001 | The relationship between the severity of dementia and subjective well-being | Aging & Mental Health | Published not from January 2010 to 31 March 2024 |
| 2610 | Foroughi, E.;Misajon, R.;Cummins, R. A.; | 2001 | The relationships between migration, social support, and social integration on quality of life | Behaviour Change | Published not from January 2010 to 31 March 2024 |
| 2611 | Lawler-Row, K. A.;Elliott, J.; | 2009 | The Role of Religious Activity and Spirituality in the Health and Well-being of Older Adults | Journal of Health Psychology | Published not from January 2010 to 31 March 2024 |
| 2612 | Kliewer, W.;Lepore, S. J.;Oskin, D.;Johnson, P. D.; | 1998 | The role of social and cognitive processes in children's adjustment to community violence | Journal of Consulting and Clinical Psychology | Published not from January 2010 to 31 March 2024 |
| 2613 | McNiff, J.; | 2008 | The significance of 'I' in educational research and the responsibility of intellectuals | South African Journal of Education | Published not from January 2010 to 31 March 2024 |

| | | | | | |
|---|---|---|---|---|---|
| 2614 | Powell, J.;Robison, J.;Roberts, H.;Thomas, G.; | 2007 | The single assessment process in primary care: Older people's accounts of the process | British Journal of Social Work | Published not from January 2010 to 31 March 2024 |
| 2615 | Cox, B. J.;Clara, I. P.;Sareen, J.;Stein, M. B.; | 2008 | The structure of feared social situations among individuals with a lifetime diagnosis of social anxiety disorder in two independent nationally representative mental health surveys | Behavior Research and Therapy | Published not from January 2010 to 31 March 2024 |
| 2616 | Fry, P. S.; | 2001 | The unique contribution of key existential factors to the prediction of psychological well-being of older adults following spousal loss | Gerontologist | Published not from January 2010 to 31 March 2024 |
| 2617 | Dykstra, P. A.;Keizer, R.; | 2009 | The wellbeing of childless men and fathers in mid-life | Ageing & Society | Published not from January 2010 to 31 March 2024 |
| 2618 | Wilcox, W. B.;Wolfinger, N. H.; | 2007 | Then comes marriage. Religion, race, and marriage in urban America | Social Science Research | Published not from January 2010 to 31 March 2024 |
| 2619 | Huberman, M.; | 2008 | Ticket to trade: Belgian labor and globalization before 1914 | Economic History Review | Published not from January 2010 to 31 March 2024 |
| 2620 | Tinghög, P.;Hemmingsson, T.;Lundberg, I.; | 2007 | To what extent may the association between immigrant status and mental illness be explained by socioeconomic factors? | Social Psychiatry and Psychiatric Epidemiology | Published not from January 2010 to 31 March 2024 |
| 2621 | Compton, W. C.; | 2001 | Toward a tripartite factor structure of mental health: Subjective well-being, personal growth, and religiosity | Journal of Psychology | Published not from January 2010 to 31 March 2024 |
| 2622 | Knappe, S.;Pinquart, M.; | 2009 | Tracing criteria of successful aging? Health locus of control and well-being in older patients with internal diseases | Psychology Health & Medicine | Published not from January 2010 to 31 March 2024 |
| 2623 | Bradby, H.; | 2002 | Translating culture and language: a research note on multilingual settings | Sociology of Health & Illness | Published not from January 2010 to 31 March 2024 |

| | | | | | |
|---|---|---|---|---|---|
| 2624 | Poole, D. L.;Negi, N.; | 2008 | Transnational community enterprises for social welfare in global civil society | International Journal of Social Welfare | Published not from January 2010 to 31 March 2024 |
| 2625 | Prince-Paul, M.; | 2008 | Understanding the meaning of social well-being at the end of life | Oncology Nursing Forum | Published not from January 2010 to 31 March 2024 |
| 2626 | White, P.;Smith, S. M.;Hevey, D.;O'Dowd, T.; | 2009 | Understanding Type 2 Diabetes Including the Family Member's Perspective | Diabetes Educator | Published not from January 2010 to 31 March 2024 |
| 2627 | Fuller, J.;Kelly, B.;Sartore, G.;Fragar, L.;Tonna, A.;Pollard, G.;Hazell, T.; | 2007 | Use of social network analysis to describe service links for farmers' mental health | Australian Journal of Rural Health | Published not from January 2010 to 31 March 2024 |
| 2628 | Windle, G.;Woods, R. T.; | 2004 | Variations in subjective wellbeing: the mediating role of a psychological resource | Ageing & Society | Published not from January 2010 to 31 March 2024 |
| 2629 | Lin, C. C.; | 2016 | The roles of social support and coping style in the relationship between gratitude and well-being | Personality and Individual Differences | Included |
| 2630 | Mahasneh, A. M.; | 2022 | The Relationship between Subjective Well-being and Social Support among Jordanian University Students | Psychology in Russia: State of the Art | Included |
| 2631 | Ma, C. M. S.; | 2020 | The Relationship Between Social Support and Life Satisfaction Among Chinese and Ethnic Minority Adolescents in Hong Kong: the Mediating Role of Positive Youth Development | Child Indicators Research | Included |
| 2632 | Shuo, Z.;Xuyang, D.;Xin, Z.;Xuebin, C.;Jie, H.; | 2022 | The Relationship Between Postgraduates' Emotional Intelligence and Well-Being: The Chain Mediating Effect of Social Support and Psychological Resilience | Frontiers in Psychology | Included |

| | | | | | |
|---|---|---|---|---|---|
| 2633 | Li, P.;Yang, J.;Zhou, Z.;Zhao, Z.;Liu, T.; | 2022 | The influence of college students' academic stressors on mental health during COVID-19: The mediating effect of social support, social well-being, and self-identity | Front Public Health | Included |
| 2634 | Qian, L.;Hu, W.;Jiang, M.; | 2023 | The Impact of Online Social Behavior on College Student's Life Satisfaction: Chain-Mediating Effects of Perceived Social Support and Core Self-Evaluation | Psychol Res Behav Manag | Included |
| 2635 | Kim, J.;Lee, J. E. R.; | 2011 | The Facebook paths to happiness: Effects of the number of Facebook friends and self-presentation on subjective well-being | Cyberpsychology, Behavior, and Social Networking | Included |
| 2636 | Brunsting, N. C.;Zachry, C.;Liu, J. T.;Bryant, R.;Fang, X. Y.;Wu, S. Y.;Luo, Z. D.; | 2021 | Sources of Perceived Social Support, Social-Emotional Experiences, and Psychological Well-Being of International Students | Journal of Experimental Education | Included |
| 2637 | Kalaitzaki, Argyroula;Tsouvelas, George;Koukouli, Sofia; | 2021 | Social capital, social support and perceived stress in college students: The role of resilience and life satisfaction | Stress and Health | Included |
| 2638 | Haleiwa, I.;Spalding, R.;Smith, K.;Chappell, A.;Strough, J.; | 2022 | Risk and protective factors for college students' psychological health during the COVID-19 pandemic | J Am Coll Health | Included |
| 2639 | Johnson, Ba Bs; Riley Jb Ms, Msn Fnp-Bc Faan; | 2021 | Psychosocial impacts on college students providing mental health peer support | J Am Coll Health | Included |
| 2640 | Cinalioglu, E. A.;Gazioglu, E. I.; | 2022 | Psychological Well-Being in Emerging Adulthood: The Role of Loneliness, Social Support, and Sibling Relationships in Turkey | Canadian Journal of Family and Youth | Included |
| 2641 | Guan, S. A.;Jimenez, G.;Cabrera, | 2022 | Providing Support Differentially Affects Asian | Front Psychol | Included |

| | | | | | |
|---|---|---|---|---|---|
| | J.;Cho, A.;Ullah, O.;Den Broeder, R.; | | American and Latinx Psychosocial and Physiological Well-Being: A Pilot Study | | |
| 2642 | Huang, Liang;Zhang, Ting; | 2022 | Perceived Social Support, Psychological Capital, and Subjective Well-Being among College Students in the Context of Online Learning during the COVID-19 Pandemic | The Asia - Pacific Education Researcher | Included |
| 2643 | Cahuas, A.;Marenus, M. W.;Kumaravel, V.;Murray, A.;Friedman, K.;Ottensoser, H.;Chen, W.; | 2023 | Perceived social support and COVID-19 impact on quality of life in college students: an observational study | Ann Med | Included |
| 2644 | Lee, Seungyoon;Chung, Jae Eun;Park, Namkee; | 2018 | Network Environments and Well-Being: An Examination of Personal Network Structure, Social Capital, and Perceived Social Support | Health communication | Included |
| 2645 | Kong, Feng;You, Xuqun; | 2013 | Loneliness and Self-Esteem as Mediators Between Social Support and Life Satisfaction in Late Adolescence | Social Indicators Research | Included |
| 2646 | Deichert, Nathan T.;Fekete, Erin M.;Craven, Michael; | 2021 | Gratitude enhances the beneficial effects of social support on psychological well-being. | The Journal of Positive Psychology | Included |
| 2647 | Shangguan, C. Y.;Zhang, L. H.;Wang, Y. L.;Wang, W.;Shan, M. X.;Liu, F.; | 2022 | Expressive Flexibility and Mental Health: The Mediating Role of Social Support and Gender Differences | International journal of environmental research and public health | Included |
| 2648 | Fan, C.;Liu, S.; | 2022 | Exploring the associations among perceived Teacher emotional support, resilience, COVID-19 anxiety, and mental well-being: evidence from | Curr Psychol | Included |

| | | | | | |
|---|---|---|---|---|---|
| | | | Chinese vocational college students | | |
| 2649 | Ross, L. T.;Ross, T. P.; | 2023 | Distress, Social Support, and Self-Compassion: Relationships With Mental Health Among College Students | Psychol Rep | Included |
| 2650 | Arroyo, Analisa;Curran, Timothy;Ruppel, Erin K.; | 2022 | Direct and indirect associations among self-disclosure skills, social support, and psychosocial outcomes during the transition to college | Journal of Social and Personal Relationships | Included |
| 2651 | Kuczynski, A. M.;Kanter, J. W.;Robinaugh, D. J.; | 2020 | Differential Associations Between Interpersonal Variables and quality-of-life in a sample of college students | Qual Life Res | Included |
| 2652 | Roaming, Sinjin;Howard, Krista; | 2019 | Coping with stress in college: an examination of spirituality, social support, and quality of life | Mental Health, Religion & Culture | Included |
| 2653 | Hossain, M. M.;Islam, K. M. Z.;Al Masud, A.;Hossain, M. A.;Jahan, N.; | 2023 | Antecedents and Consequences of Self-Disclosure in Subjective Well-Being: A Facebook Case With a Social Support Mediator | Sage Open | Included |
| 2654 | Siewert K, Antoniw K, Kubiak T, et al. | 2011 | The more, the better. The relationship between mismatches in social support and subjective well-being in daily life | Journal of Health Psychology | Included |
| 2655 | Peng L, Zhang J, Li M, et al | 2012 | Adverse life events and mental health of Chinese medical students: The effect of resilience, personality, and social support | Psychiatry research | Included |
| 2656 | Kong F, Ding K, Zhao J | 2015 | The relationships among gratitude, self-esteem, social support, and life satisfaction among undergraduate students | Journal of Happiness Studies | Included |

| | | | | | |
|---|---|---|---|---|---|
| 2657 | Wang H, Chua V, Stefanone M A | 2015 | Social Ties, Communication Channels, and Personal Well-Being: A Study of the Networked Lives of College Students in Singapore | American Behavioral Scientist | Included |
| 2658 | Zeidner M, Matthews G | 2016 | Ability to emotional intelligence and mental health: Social support as a mediator | Personality and individual differences | Included |
| 2659 | Tan C S, Krishnan S A P, Lee Q W | 2017 | The role of self-esteem and social support in the relationship between extraversion and happiness: A serial mediation model | Current Psychology | Included |
| 2660 | Alorani O I, Alradaydeh M F. | 2018 | Spiritual Well-being Perceived Social Support, and life satisfaction among university students | International Journal of Adolescence and Youth | Included |
| 2661 | Yıldırım M, Tanrıverdi F Ç | 2021 | Social Support, Resilience, and Subjective Well-being in College Students | Journal of Positive School Psychology | Included |
| 2662 | Arslan G | 2021 | Psychological Maltreatment and Spiritual Well-being in Turkish College Young Adults: Exploring the Mediating Effect of College Belonging and Social Support | Journal of religion and health | Included |
| 2663 | Holliman A J, Waldeck D, Jay B, et al | 2021 | Adaptability and Social Support: Examining Links with Psychological Well-being Among UK Students and Non-students | Frontiers in Psychology | Included |
| 2664 | Liu J. | 2021 | Social support mediates the effect of forgiveness on subjective well-being in college students. | Social Behavior and Personality: An International Journal | Included |

| | | | | | |
|---|---|---|---|---|---|
| 2665 | Asghar M, Minichiello A, Iqbal A. | 2022 | Perceived Factors Contributing to the Subjective Well-being of Undergraduate Engineering Students: An Exploratory Study | International Journal of Environmental Research and Public Health | Included |
| 2666 | Galián M D, Ato E | 2023 | The mediating role of negative affect in the relationship between family functioning and subjective happiness in Spanish college students | | Included |
| 2667 | Tariq H, Bilqees U, Fatima S, et al | 2023 | Relationship Between Social Support, Social Media Usage, and Psychological Well-being among Undergraduates in Different Institutions of Punjab | Annals of King Edward Medical University Lahore Pakistan | Included |
| 2668 | Xin Z | 2023 | The association between social support provision, psychological capital, subjective well-being, and sense of indebtedness among undergraduates with low socioeconomic status | BMC psychology | Included |
| 2669 | Yıldırım M, Green Z A | 2024 | Social support and resilience mediate the relationship of stress with satisfaction with life and the flourishing of youth. | British Journal of Guidance & Counselling | Included |
| 2670 | Yang K, Zhang L, Li W, et al | 2025 | Gratitude predicts well-being via resilience and social support in emerging adults: A daily diary study. | The Journal of Positive Psychology | Included |
| 2671 | Mo, P. K. H., Ma, L., Li, J., Xie, L., Liu, X., Jiang, H., ... & Lau, J. T. F | 2024 | Communication in social networking sites on offline and online social support and life satisfaction among university students: Tie strength matters | Journal of Adolescent Health | Included |

| | | | | | |
|---|---|---|---|---|---|
| 2672 | Fiset, J., & Robertson, M. C. S. | 2023 | Navigating the support landscape: Bridging the divide between social support in business schools and student mental health | *The International Journal of Management Education* | Included |
| 2673 | Kuczynski, A. M., Kanter, J. W., & Robinaugh, D. J. | 2020 | Differential associations between interpersonal variables and quality-of-life in a Sample of College Students | Quality of Life Research | Included |
| 2674 | Kase, T., Endo, S., & Oishi, K. | 2016 | Process linking social support to mental health through a sense of coherence in Japanese university students | Mental Health & Prevention | Included |
| 2675 | Sun, P., Jiang, H., Chu, M., & Qian, F. | 2014 | Gratitude and school well-being among Chinese university students: Interpersonal relationships and social support as mediators | Social Behavior and Personality: An International Journal | Included |
| 2676 | Oh, H. J., Ozkaya, E., & LaRose, R. | 2014 | How does online social networking enhance life satisfaction? The relationships among online supportive interaction, effect, perceived social support, sense of community, and life satisfaction | Computers in Human Behavior | Included |
| 2677 | Matsuda, T., Tsuda, A., Kim, E., & Deng, K. | 2014 | Association between perceived social support and subjective well-being among Japanese, Chinese, and Korean college students | Psychology | Included |
| 2678 | Kong, F., Zhao, J., & You, X. | 2013 | Self-esteem as mediator and moderator of the relationship between social support and subjective well-being among Chinese university students | Social indicators research | Included |
| 2679 | İlhan Yalçın | 2011 | Social Support and Optimism as Predictors of Life Satisfaction of College Students | International Journal for the | Included |

| | | | | | |
|---|---|---|---|---|---|
| | | | | Advancement of Counselling | |
