# Supplementary material for: A systematic review on the impact of social support on college students’ wellbeing and mental health
Source: PLoS One. 2025 Jul 11;20(7):e0325212. doi: 10.1371/journal.pone.0325212 (PMC12250717; doi:10.1371/journal.pone.0325212)
Supplement: S4 File — (PDF) [file pone.0325212.s004.pdf]

Supporting information

S4 File: Crowe Critical Appraisal Tool (CCAT) form

Supplementary Table 1: Crowe Critical Appraisal Tool (CCAT) form

**Category item**	**Item descriptors**	**Description**	**Score (1-5)**
	**1. Preliminaries**		
Title	1. Includes study aims and designs		
**Abstract**	1. Key information<br>2. Balanced and informative		
Last	1. Sufficient detail others could reproduce<br>2. Clear/concise writing, table(s), diagram(s) and figure(s)		
		**Preliminaries (/5)**	
	**2. Introduction**		
Background	1. Summary of current knowledge<br>2. Specific problem(s) addressed and reason(s) for addressing		
Objective	1. Primary objective(s), hypothesis(es), or aim(s)<br>2. Secondary question(s)		
	**Is it worth continuing?**	**Introduction (/5)**	
	**3. Design**		
Research design	1. Research design was chosen and why<br>2. Suitability of research design(s)		

Intervention, treatment, exposure	1. Intervention(s)/ treatment(s)/ exposure(s) chosen and why<br>2. Precise details of intervention(s)/ treatment(s)/ exposure(s) for each group<br>3. Intervention(s)/ treatment(s)/ exposure(s) valid and reliable	
The outcome, output, predictor, measure	1. Outcome(s)/ output(s)/ predictor(s)/ measure(s) chosen and why<br>2. Clearly define outcome(s)/ output(s)/ predictor(s)/ measure(s)<br>3. Outcome(s)/ output(s)/ predictor(s)/ measure(s) valid and reliable	
Bias, etc	1. Potential bias, confounding variables, effect modifiers, interactions<br>2. Sequence generation, group allocation, group balance, and by whom<br>3. Equivalent treatment of participants/ cases/ groups	
	**Is it worth continuing?**	**Design (/5)**
	**4. Sampling**	
Sampling method	1. Sampling method(s) chosen and why<br>2. Suitability of sampling method	
Sampling size	1. Sampling size, how chosen, and why<br>2. Suitability of sample size	
Sampling protocol	1. Target/actual/sample population(s): description and suitability<br>2. Participants/cases/groups: inclusion and exclusion criteria<br>3. Recruitment of participants/cases/groups	
	**Is it worth continuing?**	**Sampling (/5)**
	**5. Data collection**	
Collection method	1. Collection method(s) chosen and why<br>2. Suitability of collection method(s)	

Collection protocol	1. Include date(s), location(s), setting(s), personnel, material(s), process(es)<br>2. Methods to ensure/ enhance the quality of measurement/ instrumentation<br>3. Manage non-participation, withdrawal, incomplete/ lost data	
	**Is it worth continuing?**	**Data collection (/5)**
	**6. Ethical matters**	
Participant ethics	1. Informed consent, equity<br>2. Privacy, confidentiality/ anonymity	
Researcher ethics	1. Ethical approval, funding, conflict(s) of interest<br>2. Subjectivities, relationship(s) with participants/ cases	
	**Is it worth continuing?**	**Ethical matters (/5)**
	**7. Results**	
Analysis, Integration, Interpretation method	1. A.I.I. method(s) for primary outcome(s)/ output(s)/ predictor(s) chosen and why<br>2. Additional A.I.I. methods (e.g., subgroup analysis) chosen and why<br>3. Suitability of analysis/ integration/ interpretation method	
Essential analysis	1. Flow of participants/ cases/ groups through each stage of research<br>2. Demographic and other characteristics of participants/ cases/ groups<br>3. Analyze raw data, response rate, non-participation/ withdrawal/ incomplete/lost data	
The outcome, output, predictor analysis	1. Summary of results and precision for each outcome/ output/ predictor/ measure<br>2. Consideration of benefits/ harms, unexpected results, problems/ failures<br>3. Description of outlying data (e.g., diverse cases, adverse effects, minor themes)	
		**Results (/5)**
	**8. Discussion**	

Interpretation	1. Interpretation of results in the context of current evidence and objectives<br>2. Draw inferences consistent with the strength of the data<br>3. Consideration of alternative explanations for observed results<br>4. Account for bias, confounding/ effect modifiers/ interactions/ imprecision
Generalization	1. Consideration of the overall practical usefulness of the study<br>2. Description of generalizability (external validity) of the study
Concluding remarks	1. Highlight the study's particular strength<br>2. Suggest steps that may improve future results (e.g., limitations)<br>3. Suggest further studies
	**Discussion (/5)**
**9. Total**	
Total score	1. Add all scores for categories 1-8

Note: Scoring for each category is based on the guiding principles recommended in the Crowe Critical
Crowe Critical Appraisal Tool (CCAT): Version 1.4 (19 November 2013): Michael Crowe (michael.crowe@my.jcu.edu.au)
