# Supplementary material for: A systematic review on the impact of social support on college students’ wellbeing and mental health
Source: PLoS One. 2025 Jul 11;20(7):e0325212. doi: 10.1371/journal.pone.0325212 (PMC12250717; doi:10.1371/journal.pone.0325212)
Supplement: S5 File — (PDF) [file pone.0325212.s005.pdf]

Supporting information

S5 File: Critical appraisalof included studies

**Critical appraisal of included studies.**										
**Scores**										
**Category Items**		**1) Preliminaries**	**2) Introduction**	**3) Design**	**4) Sampling**	**5) Data Collection**	**6) Ethical matters (participant ethics and researcher ethics)**	**7) Results**	**8) Discussion**	**9) Aggregate Scores (/40)**
1	Chao (2011) American	5	5	4	3	3	3	5	4	32
2	Kim (2011) American	5	5	4	4	5	4	5	5	37
3	Siewert (2011) Germany	5	5	3	4	4	5	5	4	35
4	Yalçin (2011) Turkey	5	5	5	4	4	5	5	5	38
5	Peng (2012) China	5	5	4	5	5	4	4	4	36

6	Kong (2013) China	5	5	4	4	5	4	5	5	37
7	Kong (2013) China	5	5	4	5	4	4	5	5	37
8	Matsuda (2014) Japan	5	5	4	3	4	4	4	4	33
9	Oh (2014) American	5	5	4	4	5	4	4	5	36
10	Sun (2014) China	5	5	4	4	4	4	5	5	36
11	Kong (2015) China	5	5	4	4	5	3	4	5	35
12	Wang (2015) American	5	5	4	5	5	4	5	5	38
13	Kase (2016) Japan	5	5	4	4	5	3	5	5	36
14	Lin (2016) China	5	5	4	4	4	4	5	4	35
15	Zeidner (2016) Israel	3	4	4	4	4	4	5	5	33

16	Tan (2017) Malaysia	5	5	4	4	4	5	4	4	35
17	Alorani (2018) Jordan	5	5	5	5	5	4	4	4	37
18	Lee (2018) Republic of Korea	5	5	4	4	3	4	5	4	34
19	Roming (2019) American	5	5	3	3	4	4	5	5	34
20	Kuczynski (2020) American	5	5	5	5	4	4	5	5	38
21	Ma (2020) China	5	5	4	3	4	4	4	5	34
22	Yildirim (2020) United Kingdom	5	5	3	4	3	4	4	5	33
23	Arslan (2021) Turkey	5	5	3	4	4	4	5	5	35
24	Brunsting (2021) American	5	5	5	5	4	5	4	4	37
25	Deichert (2021)	5	5	3	3	3	4	5	4	32

	American									
26	Holliman (2021) United Kingdom	5	5	4	4	4	4	5	5	36
27	Johnson (2021) American	5	5	3	3	3	4	4	5	32
28	Kalaitzaki (2021) Greece	5	5	4	4	4	4	5	5	36
29	Liu (2021) China	5	5	5	5	5	4	4	5	38
30	Arroyo (2022) American	5	5	4	4	5	5	4	5	37
31	Asghar (2022) American	5	5	4	5	4	4	5	4	36
32	Cinalioglu (2022) Turkey	5	5	5	5	4	4	4	5	37
33	Fan (2022) China	5	5	4	5	4	4	4	5	36
34	Guan (2022) American	5	5	4	5	4	4	4	5	36

35	Haliwa (2022) American	5	5	5	4	4	4	4	5	36
36	Huang (2022) China	5	5	3	3	3	4	5	4	32
37	Li (2022) China	5	5	3	3	3	4	4	5	32
38	Mahasneh (2022) Jordan	5	5	4	4	4	4	5	5	36
39	Shangguan (2022) China	5	5	3	3	3	4	5	4	32
40	Shuo (2022) China	5	5	4	4	5	4	5	5	37
41	Cahuas (2023) American	5	5	4	4	5	5	5	5	38
42	Fiset (2023) American	5	5	3	3	3	4	4	4	31
43	Galián (2023) Spain	5	5	4	4	4	4	5	5	36
44	Hossain (2023) Bangladesh	5	5	4	4	5	4	4	5	36

45	Qian (2023) China	5	5	3	3	3	5	5	4	33
46	Ross (2023) American	5	5	4	4	4	5	5	5	37
47	Saeed (2023) Pakistan	5	5	4	4	4	5	5	5	37
48	Xin (2023) China	5	5	3	3	3	4	4	4	31
49	Yıldırım (2023) Pakistan	5	5	3	3	4	5	5	5	35
50	Han Mo (2024) China	5	5	5	5	3	5	4	4	36
51	Yang (2024) China	5	5	4	4	4	5	5	5	37
