# Supplementary material for: A systematic review on the impact of social support on college students’ wellbeing and mental health
Source: PLoS One. 2025 Jul 11;20(7):e0325212. doi: 10.1371/journal.pone.0325212 (PMC12250717; doi:10.1371/journal.pone.0325212)
Supplement: S6 File — (PDF) [file pone.0325212.s006.pdf]

Supporting information

S6 File: Risk_Bias_Quality_Table

CHECKLIST FOR ANALYTICAL CROSS SECTIONAL STUDIES

Critical Appraisal tools for use in JBI Systematic Reviews

INTRODUCTION

JBI is JBI is an international research organization based in the Faculty of Health and Medical Sciences at the University of Adelaide, South Australia. JBI develops and delivers unique evidence-based information, software, education, and training designed to improve healthcare practice and health outcomes. With over 70 Collaborating Entities, servicing over 90 countries, JBI is a recognised global leader in evidence-based healthcare.

JBI Systematic Reviews

The core of evidence synthesis is the systematic review of literature on a particular intervention, condition, or issue. The systematic review is essentially an analysis of the available literature (that is, evidence) and a judgment of the effectiveness or otherwise of a practice, involving a series of complex steps. JBI takes a particular view on what counts as evidence and the methods utilized to synthesize those different types of evidence. In line with this broader view of evidence, JBI has developed theories, methodologies, and rigorous processes for the critical appraisal and synthesis of these diverse forms of evidence to aid in clinical decision-making in healthcare. There now exists JBI guidance for conducting reviews of effectiveness research, qualitative research, prevalence/incidence, etiology/risk, economic evaluations, text/opinion, diagnostic test accuracy, mixed methods, umbrella reviews, and scoping reviews. Further information regarding JBI systematic reviews can be found in the JBI Evidence Synthesis Manual.

JBI Critical Appraisal Tools

All systematic reviews incorporate a process of critique or appraisal of the research evidence. The purpose of this appraisal is to assess the methodological quality of a study and to determine the extent to which a study has addressed the possibility of bias in its design, conduct, and analysis. All papers selected for inclusion in the systematic review (that is – those that meet the inclusion criteria described in the protocol) need to be subjected to rigorous appraisal by two critical appraisers. The results of this appraisal can then be used to inform the synthesis and interpretation of the results of the study. JBI Critical appraisal tools have been developed by the JBI and collaborators and approved by the JBI Scientific Committee following extensive peer review. Although designed for

© JBI, 2020. All rights reserved. JBI grants use of these tools for research purposes only. All other enquiries should be sent to jbisynthesis@adelaide.edu.au.

use in systematic reviews, JBI critical appraisal tools can also be used when creating Critically Appraised Topics (CAT), in journal clubs, and as an educational tool.

© JBI, 2020. All rights reserved. JBI grants use of these tools for research purposes only. All other enquiries should be sent to jbisynthesis@adelaide.edu.au.

JBI CRITICAL APPRAISAL CHECKLIST FOR ANALYTICAL CROSS SECTIONAL STUDIES

Reviewer_ _

Date_ _

Author_ _Year_________ Record Number_________

	Yes	No	Unclear	Not applicable
1. Were the criteria for inclusion in the sample clearly defined?	□	□	□	□
2. Were the study subjects and the setting described in detail?	□	□	□	□
3. Was the exposure measured validly and reliably?	□	□	□	□
4. Were objective, standard criteria used for measurement of the condition?	□	□	□	□
5. Were confounding factors identified?	□	□	□	□
6. Were strategies to deal with confounding factors stated?	□	□	□	□
7. Were the outcomes measured validly and reliably?	□	□	□	□
8. Was appropriate statistical analysis used?	□	□	□	□

Overall appraisal: Include □ Exclude □ Seek further info □

Comments (Including reason for exclusion)

____

____

____

____

© JBI, 2020. All rights reserved. JBI grants use of these tools for research purposes only. All other enquiries should be sent to jbisynthesis@adelaide.edu.au.

EXPLANATION OF ANALYTICAL CROSS SECTIONAL STUDIES CRITICAL APPRAISAL

How to cite: Moola S, Munn Z, Tufanaru C, Aromataris E, Sears K, Sfetcu R, Currie M, Qureshi R, Mattis P, Lisy K, Mu P-F. Chapter 7: Systematic reviews of etiology and risk. In: Aromataris E, Munn Z (Editors). *JBI Manual for Evidence Synthesis.* JBI, 2020. Available from https://synthesismanual.jbi.global

Analytical cross sectional studies Critical Appraisal Tool

Answers: Yes, No, Unclear or Not/Applicable

1. Were the criteria for inclusion in the sample clearly defined?

The authors should provide clear inclusion and exclusion criteria that they developed before the recruitment of the study participants. The inclusion/exclusion criteria should be specified (e.g., risk, stage of disease progression) with sufficient detail and all the necessary information critical to the study.

2. Were the study subjects and the setting described in detail?

The study sample should be described in sufficient detail so that other researchers can determine if it is comparable to the population of interest to them. The authors should provide a clear description of the population from which the study participants were selected or recruited, including demographics, location, and period.

3. Was the exposure measured validly and reliably?

The study should clearly describe the method of measurement of exposure. Assessing validity requires that a 'gold standard' is available to which the measure can be compared. The validity of exposure measurement usually relates to whether a current measure is appropriate or whether a measure of past exposure is needed.
Reliability refers to the processes included in an epidemiological study to check the repeatability of measurements of the exposures. These usually include intra-observer reliability and inter-observer reliability.

4. Were objective, standard criteria used for measurement of the condition?

It is useful to determine if patients were included in the study based on either a specified diagnosis or definition. This is more likely to decrease the risk of bias. Characteristics are another useful approach to matching groups, and studies that did not use specified diagnostic methods or definitions should provide evidence on matching by key characteristics.

5. Were confounding factors identified?

Confounding has occurred where the estimated intervention exposure effect is biased by the presence of some difference between the comparison groups (apart from the exposure investigated/of interest). Typical confounders include baseline characteristics, prognostic factors, or concomitant exposures (e.g. smoking). A confounder is a difference between the comparison groups and it influences the direction of the study results. A high-quality study at the level of cohort design

© JBI, 2020. All rights reserved. JBI grants use of these tools for research purposes only. All other enquiries should be sent to jbisynthesis@adelaide.edu.au.

will identify the potential confounders and measure them (where possible). This is difficult for studies where behavioral, attitudinal, or lifestyle factors may impact the results.

6. Were strategies to deal with confounding factors stated?

Strategies to deal with the effects of confounding factors may be dealt with in the study design or data analysis. By matching or stratifying the sampling of participants, the effects of confounding factors can be adjusted. When dealing with adjustments in data analysis, assess the statistics used in the study. Most will be some form of multivariate regression analysis to account for the confounding factors measured.

7. Were the outcomes measured validly and reliably?

Read the methods section of the paper. If e.g. lung cancer is assessed based on existing definitions or diagnostic criteria, then the answer to this question is likely to be yes. If lung cancer is assessed using observer-reported, or self-reported scales, the risk of over- or under-reporting is increased, and objectivity is compromised. Importantly, determine if the measurement tools used were validated instruments as this has a significant impact on outcome assessment validity.

Having established the objectivity of the outcome measurement (e.g. lung cancer) instrument, it's important to establish how the measurement was conducted. Were those involved in collecting data trained or educated in the use of the instrument/s? (e.g. radiographers). If there was more than one data collector, were they similar in terms of level of education, clinical or research experience, or level of responsibility in the piece of research being appraised?

8. Was appropriate statistical analysis used?

As with any consideration of statistical analysis, consideration should be given to whether there was a more appropriate alternate statistical method that could have been used. The methods section should be detailed enough for reviewers to identify which analytical techniques were used (in particular, regression or stratification) and how specific confounders were measured.

For studies utilizing regression analysis, it is useful to identify if the study identified which variables were included and how they related to the outcome. If stratification was the analytical approach used, were the strata of analysis defined by the specified variables? Additionally, it is also important to assess the appropriateness of the analytical strategy in terms of the assumptions associated with the approach as differing methods of analysis are based on differing assumptions about the data and how it will respond.

© JBI, 2020. All rights reserved. JBI grants use of these tools for research purposes only. All other enquiries should be sent to jbisynthesis@adelaide.edu.au.

© JBI, 2020. All rights reserved. JBI grants use of these tools for research purposes only. All other enquiries should be sent to jbisynthesis@adelaide.edu.au.

Table 1 JBI Risk of Bias Assessment for Included Studies

Study (Author, Year)	Q1	Q2	Q3	Q4	Q5	Q6	Q7	Q8	Overall Risk
Chao (2011)	Y	Y	Y	Y	N	N	Y	Y	Moderate
Kim & Lee (2011)	Y	Y	Y	Y	N	N	Y	Y	Moderate
Siewert et al. (2011)	Y	Y	Y	Y	N	N	Y	Y	Moderate
Yalรงฤฑn (2011)	Y	Y	Y	Y	N	N	Y	Y	Moderate
Peng et al. (2012)	Y	Y	Y	Y	Y	Y	Y	Y	Low
Kong & You (2013)	Y	Y	Y	Y	Y	Y	Y	Y	Low
Kong et al. (2013)	Y	Y	Y	Y	N	N	Y	Y	Moderate
Matsuda et al. (2014)	Y	Y	Y	Y	N	N	Y	Y	Moderate
Oh et al. (2014)	Y	Y	Y	Y	N	N	Y	Y	Moderate
Sun et al. (2014)	Y	Y	Y	Y	N	N	Y	Y	Moderate
Kong et al. (2015)	Y	Y	Y	Y	Y	Y	Y	Y	Low
Wang et al. (2015)	Y	Y	Y	Y	N	N	Y	Y	Moderate
Kase et al. (2016)	Y	Y	Y	Y	Y	Y	Y	Y	Low
Lin (2016)	Y	Y	Y	Y	Y	Y	Y	Y	Low
Zeidner & Matthews (2016)	Y	Y	Y	Y	N	N	Y	Y	Moderate
Tan et al. (2017)	Y	Y	Y	Y	N	N	Y	Y	Moderate
Alorani & Alradaydeh (2018)	Y	Y	Y	Y	N	N	Y	Y	Moderate
Lee et al. (2018)	Y	Y	Y	Y	N	N	Y	Y	Moderate
Roming & Howard (2019)	Y	Y	Y	Y	N	N	Y	Y	Moderate
Kuczynski et al. (2020)	Y	Y	Y	Y	N	N	Y	Y	Moderate
Ma (2020)	Y	Y	Y	Y	Y	Y	Y	Y	Low
Yฤฑldฤฑrฤฑm & Tanrฤฑverdi (2020)	Y	Y	Y	Y	N	N	Y	Y	Moderate
Arslan (2021)	Y	Y	Y	Y	N	N	Y	Y	Moderate
Brunsting et al. (2021)	Y	Y	Y	Y	N	N	Y	Y	Moderate
Deichert et al. (2021)	Y	Y	Y	Y	Y	Y	Y	Y	Low

Holliman et al. (2021)	Y	Y	Y	Y	N	N	Y	Y	Moderate
Johnson & Riley (2021)	Y	Y	Y	Y	Y	Y	Y	Y	Low
Kalaitzaki et al. (2021)	Y	Y	Y	Y	Y	Y	Y	Y	Low
Liu (2021)	Y	Y	Y	Y	N	N	Y	Y	Moderate
Arroyo et al. (2022)	Y	Y	Y	Y	N	N	Y	Y	Moderate
Cinalioglu & Gazioglu (2022)	Y	Y	Y	Y	N	N	Y	Y	Moderate
Fan & Liu (2022)	Y	Y	Y	Y	N	N	Y	Y	Moderate
Guan et al. (2022)	Y	Y	Y	Y	Y	Y	Y	Y	Low
Haliwa et al. (2022)	Y	Y	Y	Y	N	N	Y	Y	Moderate
Huang & Zhang (2022a)	Y	Y	Y	Y	N	N	Y	Y	Moderate
Huang & Zhang (2022b)	Y	Y	Y	Y	N	N	Y	Y	Moderate
Mahasneh (2022)	Y	Y	Y	Y	Y	Y	Y	Y	Low
Shangguan et al. (2022)	Y	Y	Y	Y	Y	Y	Y	Y	Low
Shuo et al. (2022)	Y	Y	Y	Y	N	N	Y	Y	Moderate
Cahuas et al. (2023)	Y	Y	Y	Y	N	N	Y	Y	Moderate
Fiset & Robertson (2023)	Y	Y	Y	Y	N	N	Y	Y	Moderate
Galisรกn & Ato (2023)	Y	Y	Y	Y	N	N	Y	Y	Moderate
Hossain et al. (2023)	Y	Y	Y	Y	N	N	Y	Y	Moderate
Qian et al. (2023)	Y	Y	Y	Y	N	N	Y	Y	Moderate
Van Petegem et al. (2008)	Y	Y	Y	Y	N	N	Y	Y	Moderate
Saeed et al. (2023)	Y	Y	Y	Y	N	N	Y	Y	Moderate
Xin (2023)	Y	Y	Y	Y	N	N	Y	Y	Moderate
Yฤฑldฤฑrฤฑm & Green (2023)	Y	Y	Y	Y	N	N	Y	Y	Moderate
Han Mo et al. (2024)	Y	Y	Y	Y	N	N	Y	Y	Moderate
Yang et al. (2024)	Y	Y	Y	Y	Y	Y	Y	Y	Low

Y = Yes (criterion met); UN = Unclear; N No (criterion not met). Overall risk: Green = Low, Yellow = Moderate, Red = High.

Table 2: GRADE Certainty of Evidence Assessment for Each Study and Outcome

Study (Author, Year)	Outcome	Risk of Bias	Inconsistency	Indirectness	Imprecision	Publication Bias	Overall Certainty	Summary Comment
Chao (2011)	Well-being	Moderate	Not Serious	Not Serious	Not Serious	Likely	Low	Consistent effects; observational design limits causal inference.
Kim & Lee (2011)	Subjective Well-being	Moderate	Not Serious	Not Serious	Not Serious	Likely	Low	Social media and support link to well-being; moderate confidence due to design.
Siewert et al. (2011)	Subjective Well-being	Moderate	Not Serious	Not Serious	Serious	Likely	Very Low	Support mismatch; small sample raises uncertainty.
Yalçın (2011)	Life Satisfaction	Moderate	Not Serious	Not Serious	Not Serious	Likely	Low	Support predicts life satisfaction; consistent results but moderate bias.
Peng et al. (2012)	Mental Health	Low	Not Serious	Not Serious	Not Serious	Likely	Moderate	High-quality evidence linking resilience and mental health outcomes.
Kong & You (2013)	Life Satisfaction	Low	Not Serious	Not Serious	Not Serious	Likely	Moderate	Validated structural paths for satisfaction; strong statistical modeling.
Kong et al. (2013)	Self-esteem	Moderate	Not Serious	Not Serious	Serious	Likely	Low	Confounding limits confidence in the self-esteem pathway.

Matsuda et al. (2014)	Quality of Life	Moderate	Not Serious	Not Serious	Not Serious	Likely	Low	Support linked to quality of life; clear direction but moderate design bias.
Oh et al. (2014)	Stress	Moderate	Not Serious	Not Serious	Not Serious	Likely	Low	Support is inversely related to stress; design limits causal conclusions.
Sun et al. (2014)	School Well-being	Moderate	Not Serious	Not Serious	Not Serious	Likely	Low	School climate and gratitude impact well-being; consistent but observational.
Kong et al. (2015)	Life Satisfaction	Low	Not Serious	Not Serious	Not Serious	Likely	Moderate	Support predicts satisfaction; high methodological rigor.
Wang et al. (2015)	Gratitude	Moderate	Not Serious	Not Serious	Not Serious	Likely	Low	Gratitude correlated with life satisfaction; moderate design bias.
Kase et al. (2016)	Mental Health	Low	Not Serious	Not Serious	Not Serious	Likely	Moderate	Support and coherence linked with mental health outcomes; solid control.
Lin (2016)	Optimism	Low	Not Serious	Not Serious	Not Serious	Likely	Moderate	Positive outlook linked to well-being; consistent across populations.
Zeidner & Matthews (2016)	Emotional Intelligence	Moderate	Not Serious	Not Serious	Serious	Likely	Low	Emotion regulation and well-being are connected; a small sample limits precision.
Tan et	Happiness	Moderate	Not Serious	Not Serious	Not Serious	Likely	Low	Support and personality traits

al. (2017)								affect happiness; clear associations.
Alorani & Alradaydeh (2018)	Spiritual Well-being	Moderate	Not Serious	Not Serious	Not Serious	Likely	Low	Spiritual well-being is driven by community and support; and consistent patterns.
Lee et al. (2018)	Coping Strategies	Moderate	Not Serious	Not Serious	Serious	Likely	Low	Stress coping strategies aligned with perceived support; inconsistent sample.
Roming & Howard (2019)	Hope	Moderate	Not Serious	Not Serious	Not Serious	Likely	Low	Hope mediates stress and supports relationships; strong structure.
Kuczynski et al. (2020)	School Belonging	Moderate	Not Serious	Not Serious	Not Serious	Likely	Low	Belonging mediated by support and fairness; strong link.
Ma (2020)	Life Satisfaction	Low	Not Serious	Not Serious	Not Serious	Likely	Moderate	Academic satisfaction relates to gratitude and support.
Yıldırım & Tanrıverdi (2020)	Perceived Stress	Moderate	Not Serious	Not Serious	Serious	Likely	Low	Stress is perceived as lower with social resources and; a small sample size.
Arslan (2021)	Psychological	Moderate	Not Serious	Not Serious	Not Serious	Likely	Low	Psychological flexibility buffers stress; consistent

	Flexibility							direction.
Brunsting et al. (2021)	Support Needs	Moderate	Not Serious	Not Serious	Not Serious	Likely	Low	Students identified unmet support needs; directionally valid.
Deichert et al. (2021)	Social Connectedness	Low	Not Serious	Not Serious	Not Serious	Likely	Moderate	Connectedness predicts well-being; moderate risk due to single-site sample.
Holliman et al. (2021)	Academic Support	Moderate	Not Serious	Not Serious	Not Serious	Likely	Low	Support systems predict academic success; and moderate confidence.
Johnson & Riley (2021)	Teacher Support	Low	Not Serious	Not Serious	Not Serious	Likely	Moderate	High teacher support tied to satisfaction; consistent model.
Kalaitzaki et al. (2021)	Resilience	Low	Not Serious	Not Serious	Not Serious	Likely	Moderate	Resilience improves well-being; generalizable results.
Liu (2021)	Anxiety	Moderate	Not Serious	Not Serious	Serious	Likely	Low	Anxiety moderated by support perception; unclear causality.
Arroyo et al. (2022)	Social Identity	Moderate	Not Serious	Not Serious	Not Serious	Likely	Low	Group identity is linked with emotional security.
Cinalioglu & Gazioglu (2022)	Social Comparison	Moderate	Not Serious	Not Serious	Not Serious	Likely	Low	Self-evaluation predicts emotional outcomes; small N.

Fan & Liu (2022)	Self-control	Moderate	Not Serious	Not Serious	Not Serious	Likely	Low	Self-control predicts adjustment; observed reliably.
Guan et al. (2022)	Subjective Well-being	Low	Not Serious	Not Serious	Not Serious	Likely	Moderate	Well-being enhanced by optimism and gratitude.
Haliwa et al. (2022)	Gratitude	Moderate	Not Serious	Not Serious	Not Serious	Likely	Low	Gratitude improved coping capacity; design limits directionality.
Huang & Zhang (2022a)	Academic Emotion	Moderate	Not Serious	Not Serious	Not Serious	Likely	Low	Positive emotions are linked to academic outcomes.
Huang & Zhang (2022b)	Stress Management	Low	Not Serious	Not Serious	Not Serious	Likely	Moderate	Stress regulation through support systems; moderate findings.
Mahasneh (2022)	Social Inclusion	Low	Not Serious	Not Serious	Not Serious	Likely	Moderate	Inclusion predicted satisfaction; reliable effects.
Shangguan et al. (2022)	Mindfulness	Moderate	Not Serious	Not Serious	Serious	Likely	Low	Mindfulness reduces academic tension; small sample.
Shuo et al. (2022)	Academic Stress	Moderate	Not Serious	Not Serious	Not Serious	Likely	Low	The support reduces academic stress; the general direction is positive.

Cahuas et al. (2023)	Student Motivation	Moderate	Not Serious	Not Serious	Not Serious	Likely	Low	Motivation enhanced by peer and teacher support.
Fiset & Robertson (2023)	Goal Orientation	Moderate	Not Serious	Not Serious	Serious	Likely	Low	Goal direction linked to emotional well-being.
Galian & Ato (2023)	Adjustment	Moderate	Not Serious	Not Serious	Not Serious	Likely	Low	Support predicts academic adjustment; low design bias.
Hossain et al. (2023)	Mental Health	Moderate	Not Serious	Not Serious	Not Serious	Likely	Low	Mental health is enhanced by resilience and trust.
Qian et al. (2023)	School Climate	Moderate	Not Serious	Not Serious	Not Serious	Likely	Low	Climate quality links with outcomes; observational limit.
Van Petegem et al. (2008)	Family Support	Moderate	Not Serious	Not Serious	Not Serious	Likely	Low	Family support boosts resilience; robust path analysis.
Saeed et al. (2023)	Gratitude	Moderate	Not Serious	Not Serious	Not Serious	Likely	Low	Support influences gratitude and motivation.
Xin (2023)	Study Engagement	Moderate	Not Serious	Not Serious	Not Serious	Likely	Low	Support fosters engagement and happiness.

Yıldırım & Green (2023)	Well-being	Moderate	Not Serious	Not Serious	Not Serious	Likely	Low	Support and a positive attitude enhance well-being.
Han Mo et al. (2024)	Self-efficac y	Moderate	Not Serious	Not Serious	Not Serious	Likely	Moderate	Self-efficacy mediates success; robust SEM.
Yang et al. (2024)	Growth Mindset	Low	Not Serious	Not Serious	Not Serious	Likely	Moderate	Support boosts confidence and goal pursuit.
