# Supplementary material for: A systematic review on the impact of social support on college students’ wellbeing and mental health
Source: PLoS One. 2025 Jul 11;20(7):e0325212. doi: 10.1371/journal.pone.0325212 (PMC12250717; doi:10.1371/journal.pone.0325212)
Supplement: S7 File — (PDF) [file pone.0325212.s007.pdf]

Supporting information

S7 File: The characteristics of the included studies.

Name of data extractors ： Li ruihua,Zhu qiuxia,Dong jingyi

Date of data extraction: 2024.2.10-2024.3.31

No.	Authors (Year) Country	Title	Research aims	Research method	Variables<br>Independent Variables (IV)<br>Mediator Variable (MED)<br>Moderator Variable (MOD)<br>Dependent Variables (DV)	Participants	Main findings
1	(Chao, 2011) American	Managing Stress and Maintaining Well-Being: Social Support, Problem-Focused Coping, and Avoidant Coping	To examine the moderating roles of social support and coping on the association between stress and well-being.	Quantitative	Stress (IV)<br>Problem-Focused Coping (MED)<br>Social Support (MOD)<br>Avoidant Coping	459<br>College students	The findings highlight the significance of avoidant coping.

					(MOD)<br><br>Well-Being (DV)		
2	(Kim & Lee, 2011) American	The Facebook paths to happiness: Effects of the number of Facebook friends and self-presentation on subjective well-being	To investigate whether and how Facebook increases college-age users' subjective well-being.	Quantitative	Number of Facebook Friends (IV)<br>Positive self-presentation (IV)<br>Honest self-presentation (IV)<br><br>Perceived social support (MED)<br><br>Subjective Well-Being (DV)	391 college students	The findings indicated that honest self-presentation may enhance happiness rooted in the social support provided.
3	(Siewert et al., 2011)<br><br>Germany	The more, the better. The relationship between mismatches in social support and subjective well-being in daily life	To examine the relationship between desired and received support and subjective well-being discrepancies.	Quantitative	social support (IV)<br><br>subjective well-being (DV)	30 undergraduates	Results showed that under-provision of support predicted lower well-being, whereas overprovision was related to higher well-being, suggesting a linear relationship.
4	(Yalçin, 2011)<br><br>Turkey	Social Support and Optimism as Predictors of Life Satisfaction of	To investigate the predictive value of optimism,	Quantitative	Social Support (IV)<br>Optimism (IV)	130 students	Results showed that perceived support from family, perceived

		College Students	perceived support from family, and perceived support from faculty in determining college students' life satisfaction.		Life Satisfaction (DV)		faculty support, and optimism were statistically significant predictors of life satisfaction.
5	(Peng et al., 2012)<br><br>China	Adverse life events and mental health of Chinese medical students: The effect of resilience, personality and social support	To test the moderating effect of resilience between adverse life events and mental health problems.	Quantitative	Adverse life events (IV)<br>Personality (IV)<br>Social support (IV)<br><br>Resilience (MED)<br><br>Mental health (DV)	1,998 Chinese medical students	Results showed that mental health problems had a positive correlation with adverse life events and neuroticism. Resilience moderated negative life events and mental health problems.
6	(Kong & You, 2013)<br><br>China	Loneliness and Self-Esteem as Mediators Between Social Support and Life Satisfaction in Late Adolescence	To examine the mediation effects of loneliness and self-esteem for the relationship between social support and life satisfaction.	Quantitative	Social Support (IV)<br><br>Loneliness<br>Self-Esteem (MED)<br><br>Sexes (MOD)<br><br>Life Satisfaction (DV)	389 Chinese college students	Results showed full mediation effects of loneliness and self-esteem between social support and life satisfaction. The final model also revealed a significant path from social support through

							loneliness and self-esteem to life satisfaction.
7	(Kong et al., 2013)<br><br>China	Self-esteem as mediator and moderator of the relationship between social support and subjective well-being among Chinese university students	To examine global self-esteem's mediating and moderating effects on the relationship between social support and subjective well-being.	Quantitative	Social Support (IV)<br><br>Subjective Well-Being (DV)	391 university students (260 males and 131 females) from two different Chinese universities	Results showed that global self-esteem partially mediated the influence of social support on life satisfaction and positive affect, whereas it fully mediated the influence of social support on negative affect.
8	(Matsuda et al., 2014)<br><br>Japan	Association between perceived social support and subjective well-being among Japanese, Chinese, and Korean college students	To examine the associations between perceived social support and SWB among Japanese, Chinese, and Korean college students.	Quantitative	Perceived Social Support (IV)<br><br>Subjective Well-Being (DV)	1332 college students	Results showed that family support reduced NA and significant others' support improved PA and that both types of support were associated with life satisfaction among the three groups.
9	American(Oh et al., 2014)<br><br>American	How does online social networking enhance life satisfaction? The relationships among online	To examine whether supportive interactions on social networking sites	Quantitative	Number of SNS Friends (IV)<br>Frequency of SNS Use (IV)	339 adult participants	Results showed a positive relationship between supportive interaction and

		supportive interaction, affect, perceived social support, sense of community, and life satisfaction	mediate the influence of SNS use and the number of SNS friends on perceived social support, effect, sense of community, and life satisfaction.		Supportive Interaction Positive Affect (MED)<br><br>Supportive Interaction (DV)<br>Positive Affect (DV)<br>Life Satisfaction(DV)		positive affect after the interaction.
10	China(Sun et al., 2014)<br><br>China	Gratitude and school well-being among Chinese university students: Interpersonal relationships and social support as mediators	To investigate the relationship between gratitude and school well-being and the mediating effect of interpersonal relationships and social support.	Quantitative	Gratitude (IV)<br><br>Interpersonal relationships (MED)<br>Social support (MED)<br><br>Sense of Community (DV)	782 Chinese undergraduate students	Results revealed that gratitude was positively associated with school well-being and that both interpersonal relationships and social support acted as mediators of this relationship.
11	(Kong et al., 2015)<br><br>China	The relationships among gratitude, self-esteem, social support, and life satisfaction among undergraduate students	To examine the effects of social support and self-esteem on the association between gratitude and life satisfaction among undergraduate	Quantitative	Gratitude (IV)<br><br>Self-esteem (MED)<br>Social Support (MED)<br>Life Satisfaction(MED)<br><br>Gender (MOD)	427 Chinese undergraduate	Results indicated that social support fully mediates the association between gratitude and life satisfaction.

			students.		School well-being (DV)		
12	(Wang et al., 2015)<br><br>American	Social Ties, Communication Channels, and Personal Well-Being: A Study of the Networked Lives of College Students in Singapore	To analyze the personal networks of 379 college students in Singapore to explore the social affordances of traditional and new channels in communicating with different social relationships and their associations with individual well-being.	Quantitative	Strong and weak ties (IV)<br>Communication Channels (IV)<br><br>Personal Well-Being (DV)	379 college students	Results suggest that communication channels play a complex role in how strong and weak ties are related to personal well-being.
13	(Kase et al., 2016)<br><br>Japan	Process linking social support to mental health through a sense of coherence in Japanese university students	To investigate the relationships among mental health, a "sense of coherence" (SOC), and social support in Japanese university students, focusing on gender differences.	Quantitative	Social support (IV)<br><br>Sense of coherence (MED)<br><br>Gender (MOD)<br><br>Mental health (DV)	548 university students	Results showed that improvement in social support may effectively strengthen SOC as a precaution against the decline of mental health in university students.

14	(Lin, 2016)<br><br>China	The roles of social support and coping style in the relationship between gratitude and well-being	To examine the roles of social support and coping style in the relationship between gratitude and well-being	Quantitative	Gratitude (IV)<br><br>Social support (MED)<br><br>Coping style (MED)<br><br>Ethnic (MOD)<br><br>Well-being (DV)	750 Taiwanese college students	Results showed that females with higher social support tended to use more active coping strategies when encountering a problem than their male counterparts.
15	(Zeidner & Matthews, 2016)<br><br>Israel	Ability to emotional intelligence and mental health: Social support as a mediator	To examine the association between ability emotional intelligence (EI) and psychological distress.	Quantitative	Ability (IV)<br><br>Emotional intelligence (IV)<br><br>Social support (MED)<br><br>Mental health (DV)	185 Israeli undergraduate students	Results showed that social support was a significant mediator of the effects of EI on distress.
16	(Tan et al., 2017)<br><br>Malaysia	The role of self-esteem and social support in the relationship between extraversion and happiness: A serial mediation model	To propose and test a path model illustrating the mediating effects of self-esteem and social support on the linkage of extraversion and happiness.	Quantitative	Extraversion (IV)<br><br>Self-Esteem (MED)<br>Social Support (MED)<br><br>Happiness (DV)	311 Undergraduate students	Results showed that self-esteem and social support mediated the relationship between extraversion and happiness.

17	(Alorani & Alradaydeh, 2018)<br><br>Jordan	Spiritual Well-being Perceived Social Support, and life satisfaction among university students	To identify the relationship between spiritual well-being, perceived social support, and life satisfaction.	Quantitative	Spiritual well-being (IV)<br><br>Perceived social support (DV)	919 students at the University of Jordan	Results showed that both domains of spiritual well-being had a significant positive correlation with all sources of perceived social support.
18	(Lee et al., 2018)<br><br>Republic of Korea	Network Environments and Well-Being: An Examination of Personal Network Structure, Social Capital, and Perceived Social Support	To test the structural characteristics of personal networks, the distinction between offline and online social capital, and different indicators of well-being outcomes.	Quantitative	Personal Network Structure (IV)<br>Social Capital (IV)<br><br>Perceived Social Support (MED)<br><br>Life satisfaction (DV)	574 college students	Results showed that two dimensions of personal networks—density and gender homophily—and social capital in the form of offline bonding capital explained perceived social support.
19	(Roming & Howard, 2019)<br><br>American	Coping with stress in college: An examination of spirituality, social support, and quality of life	To evaluate specific adaptive coping strategies, including spirituality, social support, and healthy behaviors, that are associated with better	Quantitative	Stress (IV)<br>Spirituality (IV)<br>Social support (IV)<br><br>Perceived Social Support (DV)	440 college students in the United States	Spiritual growth and social support were essential adaptive factors connected to a better quality of life for college students.

			quality of life in a college student population.				
20	(Kuczynski et al., 2020)<br><br>American	Differential associations between interpersonal variables and quality-of-life in a Sample of College Students	To estimate a path model, we looked at the unique variance accounted for by each, and finally, we used network analysis to examine the network of direct and indirect associations among these variables and quality of life.	Quantitative	Interpersonal Variables (IV)<br><br>Well-Being (DV)	1,456 students from four universities across the United States	Results showed that loneliness had the strongest association with quality of life across all analyses.
21	(Ma, 2020)<br><br>China	The Relationship Between Social Support and Life Satisfaction Among Chinese and Ethnic Minority Adolescents in Hong Kong: the Mediating Role of Positive Youth Development	To investigate whether positive youth development mediated the relationship between social support and life satisfaction and b) compare whether these associations varied with	Quantitative	Social Support (IV)<br><br>Positive Youth Development (MED)<br><br>Quality of life (DV)	700 Chinese and ethnic minority adolescents in Hong Kong	Results showed that positive youth development does play a mediating role in the relationship between social support and life satisfaction.

			ethnicity.				
22	(Yildirim & Tanrýverdi, 2020) United Kingdom	Social Support, Resilience, and Subjective Well-being in College Students	To test the psychometric properties and dimensionality of the BPSSQ in Turkish and the mediating effect of resilience in the relationship between social support and satisfaction with life.	Quantitative	Social support (IV)<br><br>Resilience (MED)<br><br>Quality-of-Life (DV)	202 college students	Results showed that social support significantly predicted resilience and satisfaction with life.<br>Resilience also predicts satisfaction with life.
23	(Arslan, 2021)<br><br>Turkey	Psychological Maltreatment and Spiritual Well-being in Turkish College Young Adults: Exploring the Mediating Effect of College Belonging and Social Support	To examine whether college belonging and social support mediate the association between childhood psychological maltreatment and spiritual well-being among college young adults.	Quantitative	Psychological Maltreatment (IV)<br><br>College Belonging (MED)<br><br>Social Support (MED)<br><br>Life Satisfaction (DV)	493 college young adults	The results showed that psychological maltreatment not only had a direct predictive effect on spiritual well-being but also an indirect predictive effect on spiritual well-being through college belonging and social support.
24	(Brunsting et al., 2021)	Sources of Perceived Social Support, social-emotional experiences, and	To advance an understanding of international students' psychological	Quantitative	Perceived Social Support (IV)<br><br>Social Emotional Experiences	126 Graduate and undergraduate international students) from	Results showed that perceived social support from domestic students in the fall

	American	psychological well-being of international students	well-being and social-emotional experiences, we tested whether specific social influences could enhance international students' belonging and well-being and attenuate loneliness.		(MED)<br><br>satisfaction with life (DV)	two universities in the United States	predicted higher belonging in the subsequent spring, while perceived faculty social support in the fall predicted lower loneliness in the spring.
25	(Deichert et al., 2021)<br><br>American	Gratitude enhances the beneficial effects of social support on psychological well-being	To experimentally examine whether gratitude improved the benefits of receiving support during stress.	Quantitative	Social support (IV)<br><br>Spiritual Well-being (DV)	127college students	Results showed a significant interaction between gratitude and receiving social support during the speech.
26	(Holliman et al., 2021)<br><br>United Kingdom	Adaptability and Social Support: Examining Links with Psychological Well-being Among UK Students and Non-students	To investigate the roles of adaptability and social support in predicting various psychological outcomes.	Quantitative	Adaptability (IV)<br>Social Support (IV)<br><br>Psychological Well-being (DV)	Study 1: Year 12 college students (N = 73;<br>Study 2: university students (N = 102;<br>Study 3: non-studying members of	Results showed that beyond variance attributable to social support, adaptability significantly contributed to psychological well-being (life satisfaction,

						the general public (N = 141;	well-being, flourishing, and general affect) and psychological distress across all studies.
27	(Johnson & Riley Jb Ms, 2021)<br><br>American	Psychosocial impacts on college students providing mental health peer support	To examine the psychosocial effects of providing mental health peer support to college student peer support workers compared to other trained student workers.	Quantitative	Mental health (IV)<br><br>Peer support (IV)<br><br>Psychological well-being (DV)	Seventy-five students responded to the post-training survey. Six to ten weeks in their student worker position later, 53 students responded to the follow-up survey. Of the 75 students who initially responded, 53 were peer supporters, and 22 were non-peer support student workers.	Results showed that peer supporters flourished less after training than the control group. After 6 weeks of work, peer supporters exhibited lowered avoidance coping and more belonging support.
28	(Kalaitzaki et al., 2021)	Social capital, social support and perceived stress in college students:	To examine whether online and offline social capital	Quantitative	Social capital (IV)<br>Social support (IV)	403 undergraduate Greek college	Results showed that different personal ties/relationships

	Greece	The role of resilience and life satisfaction	and offline social support are associated with less perceived stress in 403 undergraduate Greek college students through the mediating role of resilience and life satisfaction.		Resilience (MED)<br><br>Life satisfaction (MED)<br><br>Psychological Well-being (DV)		are associated with perceived stress through diversified pathways, which are different for men and women.
29	(Liu, 2021)<br><br>China	Social support mediates the effect of forgiveness on subjective well-being in college students.	To explore the relationship between forgiveness and subjective well-being (SWB) and the mediating effect of social support in this relationship.	Quantitative	Forgiveness (IV)<br><br>Social support (MED)<br><br>Psychosocial impacts (DV)	443 college students from Henan	Results showed that both interpersonal forgiveness and self-forgiveness were significantly correlated with SWB.
30	(Arroyo et al., 2022)<br><br>American	Direct and indirect associations among self-disclosure skills, social support, and psychosocial outcomes during the transition to	To explore direct and indirect associations between self-disclosure skills, social support, and psychosocial outcomes	Quantitative	Self-disclosure skills (IV)<br>Social support (IV)<br><br>Perceived stress (DV)	345 participants	Results showed that more friend support was associated with better psychosocial outcomes between participants.

		college	during the transition to college. Incoming college students in the U.S. completed online surveys throughout their first academic year.				
31	(Asghar et al., 2022) American	Perceived Factors Contributing to the Subjective Well-being of Undergraduate Engineering Students: An Exploratory Study	To investigate the condition of subjective well-being (SWB) of undergraduate engineering students to understand the factors that they perceive as positively contributing to their overall well-being in an engineering college environment.	Qualitative	Loneliness (IV) Subjective well-being (DV)	eight undergraduate engineering students (3 women, five men, 1 Asian White, 2 Latinx White, and 5 White)	The resulting 11 themes were then re-grouped and conceptualized into seven factors (faculty support, learning experiences, support environment, financial support, engineering practice opportunities, task organization, and task orientation) for precise mapping, understanding, and explanation
32	(Cinalioglu & Gazioglu,	Psychological Well-Being in Emerging	To examine the psychological well-being of	Quantitative	Social Support (IV) Sibling	422 university students	Results showed that loneliness, attitudes towards

	2022)<br><br>Turkey	Adulthood: The Role of Loneliness, Social Support, and Sibling Relationships in Turkey	Turkish emerging adults in terms of loneliness, dimensions of perceived social support, and attitudes toward sibling relationships.		Relationships (IV)<br><br>Psychosocial outcomes (DV)		sibling relations, and perceived social support from family were significant predictors of psychological well-being.
33	(Fan & Liu, 2022)<br><br>China	Exploring the associations among perceived Teacher emotional support, resilience, COVID-19 anxiety, and mental well-being: evidence from Chinese vocational college students	To examine the relationship between perceived teacher emotional support, Covid-19 anxiety, resilience, and mental well-being among Chinese vocational college students during the Covid-19 pandemic.	Quantitative	Perceived teacher emotional support (IV)<br><br>Covid- 19 anxiety (MED)<br><br>Psychological Well-Being (DV)	1469 Chinese vocational college students	Results showed that teacher emotional support was an essential promoter for building up mental well-being but not a buffer for COVID-19 within the Chinese cultural context, and COVID-19 anxiety was significantly and negatively related to mental well-being.
34	(Guan et al., 2022)<br><br>American	Providing Support Differentially Affects Asian American and Latinx Psychosocial and	To test how providing support can confer benefits, particularly for Asian American	Quantitative	Resilience (IV)<br><br>Mental well-being (DV)	48 Asian American and Latinx college students	Results showed that those who supported a family member experienced higher self-esteem

		Physiological Well-Being: A Pilot Study	and Latinx individuals.				than those in the control condition.
35	(Haliwa et al., 2022)<br><br>American	Risk and protective factors for college students' psychological health during the COVID-19 pandemic	To identify risk (e.g., perceived risk of contracting COVID-19) and protective factors (i.e., social support, mindfulness) for positive (i.e., subjective happiness, satisfaction with life) and negative (i.e., depression, anxiety, stress) aspects of psychological health.	Quantitative	Providing Support (IV)<br><br>Psychosocial and Physiological Well-Being (DV)	251 college students at a mid-Atlantic university	Results showed that a greater perceived risk of contracting COVID-19 was associated with more significant depression, anxiety, and stress. Greater mindfulness was a protective factor for psychological health.
36	(Huang & Zhang, 2022)<br><br>China	Perceived Social Support, Psychological Capital, and Subjective Well-Being among College Students in the Context of Online Learning during the COVID-19	To examine the relationship between perceived social support and subjective well-being among college students in the context of online learning	Quantitative	Risk and protective factors (IV)<br><br>Psychological Capital (MED)<br><br>Psychological health (DV)	515 college students in China	The results showed that perceived social support was significantly and positively associated with life satisfaction and positive affect and was significantly and

		Pandemic	during the COVID-19 pandemic.				negatively related to negative affect among college students learning online during the COVID-19 pandemic.
37	(Huang & Zhang, 2022)<br><br>China	The influence of college students' academic stressors on mental health during COVID-19: The mediating effect of social support, social well-being, and self-identity	To investigate the influence of academic stressors on mental health and the mediating effect of social support, social well-being, and self-identity among college students during the outbreak of COVID-19.	Quantitative	Perceived Social Support (IV)<br><br>Social support (MED)<br>Social well-being<br>Self-identity (MED)<br><br>Subjective Well-Being (DV)	900 college students	The results showed that (1) academic stressors had a significantly negative correlation with social support, social well-being, and self-identity while having a significantly positive correlation with mental health; (2) academic stressors could positively predict mental health; (3) this effect was mediated by social support, social well-being, and self-identity.
38	(Mahasneh, 2022)	The Relationship between Subjective Well-being and	To identify any significant gender differences in	Quantitative	Students' academic stressors (IV)	679 male and female undergraduate students	Results showed no significant differences in the level of subjective

	Jordan	Social Support among Jordanian University Students	the level of subjective well-being and to examine the relationship between subjective well-being and social support among a sample of Hashemite University students.		Mental health (DV)	from the Hashemite University	well-being due to the gender variable but indicated substantial differences between genders in satisfaction with academics and school connectedness.
39	(Shangguan et al., 2022)<br><br>China	Expressive flexibility and mental health: The mediating role of social support and gender differences	To explore whether social support mediated the association between expressive flexibility and depression as well as life satisfaction and whether there were gender differences in these relationships.	Quantitative	Social Support (IV)<br><br>Social Support (MED)<br>Gender Differences (MED)<br><br>Subjective Well-being (DV)	711 voluntary college students	Results showed that expressive flexibility positively affected life satisfaction, and social support mediated this association. Social support also mediated the relationship between expressive flexibility and depression.
40	(Shuo et al., 2022)<br><br>China	The Relationship Between Postgraduates' Emotional Intelligence and	To explore the relationship between postgraduates' emotional	Quantitative	Expressive Flexibility (IV)<br><br>Social Support (MED)	1,228 postgraduates	Results showed a chain effect between postgraduates' social support and

		Well-Being: The Chain Mediating Effect of Social Support and Psychological Resilience	intelligence and well-being by investigating the mediating effects of social support and psychological resilience.		Psychological Resilience(MED)<br><br>Mental Health (DV)		psychological resilience mediated by their emotional intelligence and well-being.
41	(Cahuas et al., 2023)<br><br>American	Perceived social support and COVID-19 impact on quality of life in college students: an observational study	To assess the current status of perceived social support and COVID-19 impact.	Quantitative	Postgraduates' Emotional Intelligence (IV)<br><br>Gender (MOD)<br><br>Well-Being (DV)	1296 university students	Results showed that perceived social support from family significantly predicted the total sample.
42	(Fiset & Robertson, 2023)<br><br>American	Navigating the support landscape: Bridging the divide between social support in business schools and student mental health	To test internal stakeholders (e.g., faculty, student peers, administrators, and staff) in which ways they can engage to improve student mental health outcomes on campus.	Quantitative	Perceived social support (IV)<br>COVID-19 impact (IV)<br><br>quality of life (DV)	205 current business school students	Results showed that student's mental health is positively related to perceived support from faculty and student peers.
43	(Galián & Ato, 2023)<br><br>Spain	The mediating role of negative affect in the relationship between family functioning and	To test the link between these factors is scarcely considered in emerging	Quantitative	Social support (IV)<br><br>Negative affect (MED)	347 college students	Results showed that moderate family cohesion and flexibility scores were positively

		subjective happiness in Spanish college students	adulthood.		Student mental health (DV)		associated with a young person's subjective happiness.
44	(Hossain et al., 2023)<br><br>Bangladesh	Antecedents and Consequences of Self-Disclosure in Subjective Well-Being: A Facebook Case With a Social Support Mediator	To investigate antecedents and consequences of self-disclosure in subjective well-being with social support as a mediator.	Quantitative	family functioning (IV)<br><br>Social Support (MED)<br><br>subjective happiness (DV)	244 respondents	Results showed that self-disclosure significantly depends on information, social influence, and social media use.
45	(Qian et al., 2023)<br><br>China	The Impact of Online Social Behavior on College Student's Life Satisfaction: Chain-Mediating Effects of Perceived Social Support and Core Self-Evaluation	To examine the influence of social behavior on college student's life satisfaction and the mediating effects of perceived social support and core self-evaluation.	Quantitative	Self-Disclosure (IV)<br><br>Perceived Social Support (MED)<br><br>Core Self Evaluation (MED)<br><br>Subjective Well-Being (DV)	779 college students	Results showed that online social behavior significantly positively predicted perceived social support and life satisfaction.
46	(Van Petegem et al., 2008)<br><br>American	Distress, Social Support, and Self-Compassion: Relationships with Mental Health Among College Students	To investigate how distress, conceptualized as an interaction between hassles and stress perceptions, related to mental health.	Quantitative	Online Social Behavior (IV)<br><br>Social Support (MOD)<br>Self-Compassion (MOD)<br><br>College Student's Life Satisfaction	185 from a mid-sized university	Results showed that survey questions pertained to hassles and stress perceptions, mental health, perceived social support, and self-compassion.

					(DV)		
47	(Saeed et al., 2023)<br><br>Pakistan	Relationship Between Social Support, Social Media Usage, and Psychological Well-being among Undergraduates in Different Institutions of Punjab	To find the relationship between social support, social media usage, and psychological well-being among undergraduates aged 18-25.	Quantitative	Distress (IV)<br><br>Mental Health(DV)	843 college students	Results showed that social support received from online sources had no significant psychological impact.
48	(Xin, 2023)<br><br>China	The association between social support provision, psychological capital, subjective well-being, and sense of indebtedness among undergraduates with low socioeconomic status	To test whether and when social support provision can benefit the providers' positive psychological capital and subjective well-being.	Quantitative	Social Support (IV)<br>Social Media Usage (IV)<br><br>Psychological Wellbeing(DV)	732 Chinese undergraduates	Results showed that impoverished college students' social support provision was positively associated with life satisfaction, positive affect, and psychological capital and negatively associated with adverse effects.
49	(Yıldırım & Green, 2023)<br><br>Pakistan	Social support and resilience mediate the relationship of stress with satisfaction with life and the flourishing of	To examine whether social support and resilience mediate the association of stress with satisfaction with	Quantitative	Stress (IV)<br><br>Social support (MED)<br>Resilience (MED)<br><br>Satisfaction with	230 undergraduate students	Results revealed that (i) perceived stress was indirectly related to satisfaction with life via social support and

		youth.	life and flourishing.		life and flourishing of youth (DV)		resilience.
50	(Han Mo et al., 2024)<br><br>China	Communication in social networking sites on offline and online social support and life satisfaction among university students: Tie strength matters	To examine the association between social interaction with weak and strong ties to SNS and life satisfaction among Chinese college and university students.	Quantitative	Communication in Social Networking Sites (IV)<br><br>Life Satisfaction (DV)	26,547 students	Results showed that social interaction with strong ties to SNS was associated with increased offline social support but had no significant association with online backing.
51	(Yang et al., 2024)<br><br>China	Gratitude predicts well-being via resilience and social support in emerging adults: A daily diary study.	To better understand how gratitude is linked to well-being, the present study adopted a daily diary method to explore the mediating roles.	Quantitative	Gratitude (IV)<br><br>Resilience (MED)<br>Social support (MED)<br><br>well-being (DV)	153 undergraduates	Results showed that daily resilience and social support mediated the relationship between gratitude and well-being.
