# Supplementary material for: A systematic review on the impact of social support on college students’ wellbeing and mental health
Source: PLoS One. 2025 Jul 11;20(7):e0325212. doi: 10.1371/journal.pone.0325212 (PMC12250717; doi:10.1371/journal.pone.0325212)
Supplement: S8 File — (PDF) [file pone.0325212.s008.pdf]

Supporting information

S8 File: Wellbeing and Social Support Measurement.

No.	Authors (Year)	Title	Well-being instrument	Social support instrument
1	(Chao, 2011)	Managing Stress and Maintaining Well-Being: Social Support, Problem-Focused Coping, and Avoidant Coping	Mental Health Inventory (MHI): This scale consists of 38 items and uses a 5-point Likert scale to assess psychological distress and well-being among adults. It has been extensively used in nonpsychiatric samples and has well-established psychometric properties . The sample was found to have a coefficient alpha of 0.89.	The Social Support Inventory (SSI; Brown et al., 1987) is a 39-item questionnaire that assesses satisfaction with support and help received from others over the previous month. For the total score, the coefficient alpha was 0.95 in Brown et al. and .90 for the present sample.
2	(Kim & Lee, 2011)	The Facebook paths to happiness: Effects of the number of Facebook friends and self-presentation on subjective well-being	Subjective Happiness Scale (SHS): Subjective well-being (SWB) is measured using the four-item SHS, based on a 7-point Likert scale. Participants rate their overall happiness. This index was reliable, with a Cronbach's alpha of 0.86 .	Perceived Social Support: Measured by a seven-item index adapted from the Interpersonal Support Evaluation List (ISEL) scale. Items are reworded to reflect the context of Facebook use. Participants rate the availability of social support on a 5-point Likert scale. This index was reliable, with a Cronbach's alpha of 0.86 .
3	(Yalçin, 2011)	Social Support and Optimism as Predictors of Life Satisfaction of College Students	Life satisfaction is measured using the 5-item Satisfaction with Life Scale (SWLS), rated on a 7-point scale from 1 (strongly disagree) to 7 (strongly agree). Sample items include "I am	The Perceived Social Support Scale-Revised (PSSS-R) assesses support from family, friends, and teachers using 50 items rated on a 3-point scale. Developed by Yıldırım (2004), it showed high reliability, with alpha coefficients of .93 for

			satisfied with my life" and "My life is ideal." .In the present study, the Cronbach alpha coefficient was 0.74.	the total scale. In this study, alpha coefficients were .93 for the total, .93 for family support, .89 for support from friends, and .93 for support from teachers.
4	(Peng et al., 2012)	Adverse life events and mental health of Chinese medical students: The effect of resilience, personality, and social support	The Symptom Checklist-90-Revised (SCL-90-R) measures mental health across nine subscales. The scale evaluated psychiatric and somatic symptoms and achieved internal consistency coefficients ranging from 0.85 to 0.90 and test-retest correlations from 0.80 to 0.86, effectively reflecting the mental health levels of each individual.	The Social Support Rating Scale (SSRS) measures social support across three dimensions: objective, subjective, and availability, using 10 items. Higher scores indicate more support. It has high internal consistency (0.88) and test-retest reliability (0.85) in China.
5	(Kong & You, 2013)	Loneliness and Self-Esteem as Mediators Between Social Support and Life Satisfaction in Late Adolescence	The Satisfaction with Life Scale (SWLS) measures life satisfaction with five items rated on a 7-point Likert scale. Sample items include "I am satisfied with my life." In this study, the Cronbach alpha coefficient was 0.78.	The Chinese Social Support Rating Scale (CSSRS) assesses subjective support, objective support, and support utilization with 10 items. The total score measures overall social support status. It has good validity and reliability, with a Cronbach alpha of 0.71.
6	(Kong et al., 2013)	Self-esteem as mediator and moderator of the relationship between social support and subjective well-being among Chinese university students	The Satisfaction with Life Scale (SWLS) measures life satisfaction with five items on a 7-point Likert scale. Sample items: "I am satisfied with my life." It has good psychometric properties, with a Cronbach alpha of 0.78 in this study.	The Chinese Social Support Rating Scale (CSSRS) assesses subjective and objective aspects of utilizing social support, with 10 items. The total score measures overall social support status. It has been validated and is reliable, with a Cronbach alpha of 0.71.

7	(Matsuda et al., 2014)	Association between perceived social support and subjective well-being among Japanese, Chinese, and Korean college students	Satisfaction with Life Scale (SWLS): Measures life satisfaction with five items on a 7-point Likert scale. Sample items: "I am satisfied with my life." Positive and Negative Affect Schedule (PANAS): Assesses positive and negative affect with 10 items each on a 5-point scale. This study's coefficient alphas were 0.84, 0.76, and 0.86 for Japanese, Chinese, and Korean students.<br>The Positive and Negative Affect Schedule (PANAS) (Watson, Clark, & Tellegen, 1988; Kawahito, Otsuka, Kaida, & Nakata, 2011; Huang, Yang, & Ji, 2003; Lee, Kim, & Lee, 2003) consists of 20 self-report items, 10 of which measure PA (e.g., determined, inspired, enthusiastic, active) and 10 of which measure NA (e.g., jittery, afraid, nervous, ashamed). This study's alpha coefficient was 0.85, 0.88, and 0.79 for Japanese, Chinese, and Korean students.	The Multidimensional Scale of Perceived Social Support (MSPSS) consists of 12 items measuring support from family, friends, and significant others on a 7-point scale. Higher scores indicate more support. It is reliable and valid, with a coefficient alpha of 0.71.
8	(Oh et al., 2014)	How does online social networking enhance life satisfaction? The	Life Satisfaction: Measured using a four-item scale adapted from the SWLS on a 7-point Likert scale.	Perceived Social Support: Measured using a nine-item index for appraisal, companionship, and esteem support on a 7-point Likert scale.

		relationships among online supportive interaction, affect, perceived social support, sense of community, and life satisfaction	Cronbach alpha: 0.86. Affect: Positive and negative affect were measured using a modified PANAS on a 7-point Likert scale. Cronbach alphas: 0.86 (positive), 0.81 (negative).	Cronbach alphas .87 (appraisal), 0.75 (companionship), 0.72 (esteem).
9	(Sun et al., 2014)	Gratitude and school well-being among Chinese university students: Interpersonal relationships and social support as mediators	School Well-Being: Measured as a multidimensional construct including School Satisfaction: 12 items on a 5-point scale. Cronbach alpha: 0.83. Positive Affect in School: 14 items on a 6-point scale. Cronbach alpha: 0 .89. Negative Affect in School: 14 items on a 6-point scale. Cronbach alpha:0 .88.	The Multidimensional Scale of Perceived Social Support (MSPSS) measures support from significant others, family, and friends using 12 items on a 7-point scale. Example item: "My family tries to help me." Cronbach's alpha: 0.90.
10	(Kong et al., 2015) (2015)	The relationships among gratitude, self-esteem, social support, and life satisfaction among undergraduate students	The Satisfaction with Life Scale (SWLS) measures life satisfaction with five items on a 7-point Likert scale. Example items: "I am satisfied with my life." It has good reliability, with a Cronbach alpha of 0.79.	The Multidimensional Scale of Perceived Social Support (MSPSS) measures support from family, friends, and significant others with 12 items on a 7-point Likert scale. Example items: "My family tries to help me." Cronbach alpha: 0.88.
11	(Kase et al., 2016)	Process linking social support to mental health through a sense of coherence in Japanese university students	Mental health was measured using the GHQ-12, a 12-item questionnaire on a 4-point scale. Lower scores indicate better mental health. The GHQ-12 showed high reliability in this study, with Cronbach alphas of 0.82 for males and 0.79 for females.	Social support was measured using the Japanese version of the MSPSS, with 12 items across three subscales (family, friends, significant others) on a 7-point scale. The total score reflects overall support. Cronbach alphas: 0.88 (pros), 0.91 (cons).

12	(Lin, 2016)	The roles of social support and coping style in the relationship between gratitude and well-being	Well-being was measured using the Inventory of Well-Being (IW), with 10 items on a 6-point scale. It includes life satisfaction dimensions Cronbach alphas: 0.89 (LS), 0.87 (PE), 0.90 (overall).	Social support was measured using the Inventory of Social Support (ISS), with 18 items across emotional-companion support (ECS) and informational-tangible support (ITS) on a 6-point scale. Cronbach alphas: 0.94 (ECS), .086 (ITS), 0.95 (overall).
13	(Zeidner & Matthews, 2016)	Ability to emotional intelligence and mental health: Social support as a mediator	Mental health was assessed using the MHI-38, focusing on two subscales:<br>Well-Being: Measures positive mental health (e.g., happiness) with a Cronbach alpha 0.95.<br>Distress: Measures adverse mental health (e.g., anxiety) with a Cronbach alpha of 0.91.	Social support was measured using the Interpersonal Social Evaluation List (ISEL), with 40 items assessing tangible, appraisal, self-esteem, and belonging support. Each item is rated on a scale. The overall reliability (Cronbach alpha) in this study was 0.76.
14	(Tan et al., 2017)	The role of self-esteem and social support in the relationship between extraversion and happiness: A serial mediation model	Happiness was measured using the Oxford Happiness Questionnaire (OHQ), a 29-item scale on a 6-point Likert scale. Higher scores indicate greater happiness. Cronbach alpha ： 0 .88.	Social support was measured using the MSPSS, with 12 items assessing support from family, friends, and significant others on a 7-point scale. The MSPSS showed good internal consistency: overall (0.89), Family (0.83), Friends (0.84), and Significant Others (0.91).
15	(Alorani & Alradaydeh, 2018)	Spiritual Well-being Perceived Social Support, and life satisfaction among university students	Life satisfaction was measured using the SWLS, with five items on a 7-point Likert scale. Total scores range from 5 to 35, with higher scores indicating greater satisfaction. Cronbach alpha ： 0.83.	Social support was measured using the MPSSS, with 12 items on a 7-point Likert scale across three subscales: family, friends, and significant others. Total scores range from 12 to 84. Cronbach alpha ： 0.87.
16	(Lee et al., 2018)	Network Environments and Well-Being: An Examination of	Satisfaction with Life Scale (SWLS): Measures life satisfaction with five items on a 7-point scale. Higher scores	The Multidimensional Scale of Perceived Social Support (MSPSS) measures social support with 12 items on a 7-point scale from family, friends,

		Personal Network Structure, Social Capital, and Perceived Social Support	indicate greater satisfaction. Cronbach alpha: 0.88.<br><br>RAND Mental Health Inventory (MHI-5): Assesses mental health with items on a 6-point scale.<br>Cronbach alpha:0 .77.<br><br>Sense of Belonging: Measures belonging with statements/questions rated on a 1 to 5 scale. Cronbach alpha: 0.80.	and significant others. Higher scores indicate more excellent support. Cronbach alpha ： 0.92.
17	(Roming & Howard, 2019)	Coping with stress in college: An examination of spirituality, social support, and quality of life	The Health-Promoting Lifestyle Profile II (HPLP2) includes subscales for Physical Activity and Nutrition, rated on a four-point scale from "Never" to "Routinely." Higher scores indicate better habits. Reliability: Physical Activity (alpha 0.855) and Nutrition (alpha0 .814).	The Social Support from Parents and Friends Scales (SSPFS) measures received parental and peer support with 11 items on a four-point scale. Example item: "My parents ask about my school." Higher scores indicate more support. Cronbach alpha: 0 .907.
18	(Kuczynski et al., 2020)	Differential associations between interpersonal variables and quality-of-life in a Sample of College Students	The WHOQOL-BREF measures quality of life across four domains with 26 items on a 5-point scale. Domains include physical health, psychological health, social relationships, and environment. Higher scores indicate a better quality of life.<br>Cronbach alpha: 0.92.	The Medical Outcomes Study Social Support Survey (SSS) measures perceived social support with 19 items across four domains on a 5-point scale. Domains include emotional/informational, tangible, affectionate, and positive social interaction. Cronbach alpha:0 .96.

19	(Ma, 2020)	The Relationship Between Social Support and Life Satisfaction Among Chinese and Ethnic Minority Adolescents in Hong Kong: the Mediating Role of Positive Youth Development	The Satisfaction with Life Scale (SWLS) measures life satisfaction with five items on a 7-point scale. Higher scores indicate greater life satisfaction. Cronbach alpha ： 0 .89.	The MSPSS measures social support with 12 items across three subscales: family, friends, and significant others, rated on a 7-point scale. Cronbach alphas: 0.84 (family support), 0.86 (friend support), and 0.89 (significant other support).
20	(Yildirim & Tanrýverdi, 2020)	Social Support, Resilience, and Subjective Well-being in College Students	The SWLS measures subjective well-being with 5 items on a 7-point scale. Higher scores indicate greater life satisfaction. Cronbach alpha ： 0 .90.	The BPSSQ measures social support with 6 items on a 5-point scale. Higher scores indicate more excellent perceived support. Cronbach alpha： 0 .77.
21	(Arslan, 2021)	Psychological Maltreatment and Spiritual Well-being in Turkish College Young Adults: Exploring the Mediating Effect of College Belonging and Social Support	Spiritual Well-being Scale (SWS): The SWS measures spiritual well-being with 5 items on a 5-point scale. Higher scores indicate greater spiritual well-being. Cronbach alpha ： 0 .83.	Brief Perceived Social Support Questionnaire (BPSSQ): The BPSSQ measures social support with 6 items on a 5-point scale. Higher scores indicate more excellent perceived support. Cronbach alpha of 0.88.
22	(Brunsting et al., 2021)	Sources of Perceived Social Support, social-emotional experiences, and psychological well-being of international students	Ryff's 42-item Psychological Well-Being Scales measure well-being across six 5-point subscales. Example item: "Being happy with myself is more important than approval." Cronbach alpha:0 .93.	The Sources of Social Support Scale measures support from faculty, domestic students, and international students using a 5-point scale. It assesses emotional, informational, appraisal, and instrumental support.<br>Cronbach alpha: 0.89 to0 .95.

23	(Deichert et al., 2021)	Gratitude enhances the beneficial effects of social support on psychological well-being.	State Perceived Stress: Measured using the 8-item Perceived Stress Scale (PSS) on a 1-7 bipolar adjective scale. Higher scores indicate higher stress.<br>Cronbach alpha:0.89.	State Perceived Support: Measured using the 6-item Perceived Support Scale (PSP) on a 1-7 bipolar scale.<br>Cronbach alpha:0.91.
24	(Holliman et al., 2021)	Adaptability and Social Support: Examining Links With Psychological Well-being Among UK Students and Non-students	Life Satisfaction: Measured using the BMSLSS (6 items) and SWLS (5 items) on a 7-point scale.Cronbach alpha: 0.77.<br>Psychological Well-being: Measured using the ONS Personal Well-being Domain (4 items) on a 10-point scale. Cronbach alpha: 0.71.<br>Flourishing: Measured using the Flourishing Scale (8 items) on a 7-point scale. Cronbach alpha: 0.87.<br>Positive and Negative Experience (SPANE): Measures emotions over the last 4 weeks (12 items) on a 5-point scale. The negative total score (α = 0.79) is deducted from the positive total (α = 0.77) score to create a "SPANE Balance" score.<br>Psychological Distress: Measured using K10 and DASS-21 on Likert-type scales. Cronbach alpha: 0.71.	The MSPSS measures social support with 12 items on a 7-point scale from family, friends, and significant others. Example item: "I get the emotional help I need from my family."<br>Cronbach alpha: 0.92.

25	(Johnson & Riley Jb Ms, 2021)	Psychosocial impacts on college students providing mental health peer support	Well-being was measured using Ryff's 42-item Psychological Well-Being Scales on a 5-point scale. Subscales include purpose, relations, autonomy, mastery, growth, and acceptance.<br>Reliability: subscales (Alpha: 0.72 to 0.80), overall (Alpha: 0.93).	Social Support: Assessed from three sources—faculty, international students, and domestic students—likely using a structured questionnaire to measure perceived support. Specific scales or items are not detailed in the provided portion. Cronbach alpha: 0.816.
26	(Kalaitzaki et al., 2021)	Social capital, social support and perceived stress in college students: The role of resilience and life satisfaction	The SWLS measures life satisfaction (5 items) on a 7-point scale, Cronbach alpha:0 .83. The RS measures resilience (15 items) on a 7-point scale, Cronbach alpha: 0.91. The PSS-10 measures perceived stress (10 items) on a 5-point scale, Cronbach alpha:0 .75.	The SOS measures social support from significant others with 4 items on a 7-point scale. Higher scores indicate more considerable support. Cronbach alpha: 0.88 (partners), 0.88 (friends).
27	(Liu, 2021)	Social support mediates the effect of forgiveness on subjective well-being in college students.	The SWB measures life satisfaction, positive affect, and negative affect with items on a 7-point scale. Cronbach alphas: 0.88 (life satisfaction), 0.81 (positive affect), 0.76 (negative affect).	The PSSS measures social support from family, friends, and significant others with 12 items on a 7-point scale. Example items: "My family helps me." Cronbach alpha: 0.87.
28	(Cinalioglu & Gazioglu, 2022)	Psychological Well-Being in Emerging Adulthood: The Role of Loneliness, Social Support, and Sibling Relationships in Turkey	The Flourishing Scale (FS) measures psychological well-being with 8 items on a 7-point scale. Higher scores indicate greater well-being. The FS demonstrated good reliability with a Cronbach's alpha of 0.86.	The MSPSS measures perceived social support from family, friends, and significant others with 12 items on a 7-point scale.<br>Cronbach alphas: 0.83 (family), 0.89 (friends), 0.95 (significant others), 0.88 (total).
29	(Guan et	Providing Support	Mental well-being was measured	Teacher Emotional Support: Measured with a

	al., 2022)	Differentially Affects Asian American and Latinx Psychosocial and Physiological Well-Being: A Pilot Study	using the WHO-5, a 5-item scale with positively phrased items rated on a 5-point scale. The WHO-5 demonstrated good reliability with a Cronbach's alpha of 0.893.	6-item scale on a 5-point scale. Sample items: "My math teacher cares about me." The scale showed good reliability with a Cronbach's alpha of 0.907.
30	(Fan & Liu, 2022) China	Providing Support Differentially Affects Asian American and Latinx Psychosocial and Physiological Well-Being: A Pilot Study	Mental well-being (MWB) was assessed using the WHO-5, a 5-item scale with positively phrased items rated on a 5-point scale. When the WHO-5 was used in the current sample, the result of the scale showed good reliability (α=0.893).	Teacher Emotional Support (TES): Measured with a modified 6-item scale gauging students' feelings of being liked, respected, and valued by teachers during the pandemic. Based on Federici & Skaalvik (2014). In the current sample, Cronbach's alpha for the entire questionnaire was 0.907.
31	(Haliwa et al., 2022)	Risk and protective factors for college students' psychological health during the COVID-19 pandemic	DASS-21 measures depression, anxiety, and stress with 21 items on a 4-point scale. Cronbach alphas: .886 (depression), .870 (anxiety), .857 (stress). SHS measures happiness (α = .829). SWLS measures life satisfaction (α = .856).	Multidimensional Scale of Perceived Social Support (MSPSS): a 12-item scale assessing perceived social support from family, friends, and significant others. Participants rate items from 1 (very strongly disagree) to 7 (very strongly agree). Higher sum scores indicated better perceived social support (a ¼ .884).
32	(Huang & Zhang, 2022)	Perceived Social Support, Psychological Capital, and Subjective Well-Being among College Students in the Context of Online Learning during the	PANAS measures positive and negative affect with 15 items on a 4-point scale. Cronbach alphas: 0.907 (Positive Affect), 0.878 (Negative Affect).	The MSPSS measures perceived social support from family, friends, and significant others with 12 items on a 6-point scale. The calculated Cronbach is a for the overall perceived social support scale indicated good reliability (Cronbach is a = 0.910), as did its three subscales of support of family, friends, and

		COVID-19 Pandemic		significant others (Cronbach is a = 0.880, 0.885, and 0.911, respectively).
33	(Li et al., 2022)	The influence of college students' academic stressors on mental health during COVID-19: The mediating effect of social support, social well-being, and self-identity	The DASS-21 measures depression, anxiety, and stress with items rated from 0 (never) to 3 (almost always). Scores are multiplied by 2. Cronbach alpha: 0.939.	Social Support Questionnaire (SSQ): Developed by Xiao (1994), this questionnaire includes 10 items divided into three dimensions: subjective support, objective support, and utilization degree of support. Items are rated on various scales, with higher scores indicating better social support. The Cronbach's alpha of the questionnaire in this study was 0.814.
34	(Mahasneh, 2022)	The Relationship between Subjective Well-being and Social Support among Jordanian University Students	The CSSWQ measures subjective well-being with 15 items across four dimensions on a 7-point scale.<br>Cronbach alphas: 0.71 (academics), 0.72 (connectedness), 0.83 (gratitude), 0.81 (efficacy), .077 (total).	The MSPSS measures social support from family, friends, and significant others with 12 items on a 7-point scale.<br>Cronbach alphas: .084 (family), 0.79 (friends), 0.81 (significant other), 0.85 (total).
35	(Shangguan et al., 2022)	Expressive flexibility and mental health: The mediating role of social support and gender differences	BDI: 21-item Chinese depression measure. Scores 0-63, higher, indicate more severe depression.<br>Cronbach alphas:0.93.<br>SWLS: 5-item Chinese life satisfaction scale. 7-point Likert, scores 5-35, higher indicates greater satisfaction. Cronbach alphas:0.87.	PSSS: 12-item Chinese social support measure. 7-point Likert scale, statements like "There is a special person with whom I can share joys and sorrows." A total score is the sum of all items. Cronbach alphas: 0.95.
36	(Shuo et al., 2022)	The Relationship Between Postgraduates' Emotional Intelligence and Well-Being: The	Well-being was measured using the Chinese version of the Index of Well-Being (IWB) as compiled by Campbell to measure subjects' degree	Xiao's (1994) Social Support Rating Scale (SSRS) was used to measure subjects' perceived degree of social support. This scale comprises 10 items and three dimensions: subjective support,

		Chain Mediating Effect of Social Support and Psychological Resilience	of well-being experienced (Li and Zhao, 2000).<br>Cronbach alphas: 0.948.	objective support, and utilization of support. Cronbach alphas: 0.806.
37	(Arroyo et al., 2022)	Direct and indirect associations among self-disclosure skills, social support, and psychosocial outcomes during the transition to college	Well-being: Measured using the BMSLSS for life satisfaction, ONS Personal Well-being Domain for psychological well-being, and Kessler Psychological Distress Scale (K10) for psychological distress.<br>Depression:<br>Time 1 α = .89, Time 2 α = .90, Time 3 α = .91<br>Anxiety:<br>Time 1 α = .91, Time 2 α = .92, Time 3 α = .92<br>Social isolation:<br>Time 1 α = .88, Time 2 α = .92, Time 3 α = .90<br>Social inclusion:<br>Time 1 α = .91, Time 2 α = .91, Time 3 α = .91<br>Life satisfaction:<br>Time 1 α = .84, Time 2 α = .88, Time 3 α = .86<br>Positive affect:<br>Time 1 α = .81, Time 2 α = .87, Time 3 α = .86	Social Support: Measured using the Multidimensional Scale of Perceived Social Support (MSPSS), which assesses perceived social support from family, friends, and significant others.<br>Family support:<br>Time 1 α = .88, Time 2 α = .91, Time 3 α = .90<br>Significant other support:<br>Time 1 α = .91, Time 2 α = .93, Time 3 α = .92<br>Friend support:<br>Time 1 α = .90, Time 2 α = .91, Time 3 α = .92
38	(Cahuas et	Perceived social	The COVID-19 impact on quality of	Social support was measured using the

	al., 2023)	support and COVID-19 impact on quality of life in college students: an observational study	life (COVID-19 QoL): The questionnaire contains 6 questions designed for individuals to self-rate the impact of COVID-19 on quality of life in physical and mental health and personal safety. Cronbach alpha : 0.856.	Multidimensional Scale of Perceived Social Support (MSPSS) by Zimet et al., which includes items to assess perceived support from friends, family, and significant others. Cronbach alphas: 0.88.
39	(Fiset & Robertson, 2023)	Navigating the support landscape: Bridging the divide between social support in business schools and student mental health	GHQ-12: 12-item mental health measure. 4-point scale (0=never to 3=always). Sample item: "My worries have made me lose much sleep." Good reliability, Cronbach alpha：0.89.	Perceived Social Support: Adapted 6-item scale for four groups: Faculty (Cronbach alpha： 0.91), Student Peers (Cronbach alpha： 0.94), Administrators (Cronbach alpha： 0.87), Staff (Cronbach alpha ： 0.88). 5-point scale (1=strongly disagree to 5=strongly agree). Sample item: "I can count on [group] when things go wrong."
40	(Galián & Ato, 2023)	The mediating role of negative affect in the relationship between family functioning and subjective happiness in Spanish college students	SHS: 4-item global happiness measure. 7-point Likert scale (1=very unhappy to 7=very happy). Self-rating and comparison to others.<br>Cronbach alpha ： 0.81.	FACES IV (Spanish adaptation): 24-item scale assessing family dynamics. Two balanced scales (Cohesion, Flexibility) and four unbalanced scales (Enmeshed, Disengaged, Chaotic, Rigid). Measures emotional bonding and adaptability in family systems.<br>The range of reliability for the Spanish adaptation of FACES IV balanced scales was between 0.65 and 0.77, and for unbalanced scales, between 00.51 and .74.
41	(Hossain et al., 2023)	Antecedents and Consequences of Self-Disclosure in Subjective Well-Being:	Subjective Well-Being (SWB): Measures both emotional and cognitive well-being. Assesses individuals' self-evaluation of life	Perceived Social Support: Assessed support from the Facebook social network. Likely covers emotional, informational, and instrumental support dimensions. Specific scales or items are

		A Facebook Case With a Social Support Mediator	satisfaction and emotional states. Specific scales or items are not detailed. Cronbach alpha ： 0.736.	not detailed.<br>Cronbach alpha ： 0.834.
42	(Qian et al., 2023)	The Impact of Online Social Behavior on College Student's Life Satisfaction: Chain-Mediating Effects of Perceived Social Support and Core Self-Evaluation	The Life Satisfaction Questionnaire developed by Diener was used, which consists of 5 items rated on a 7-point scale from 1 (very disagree) to 7 (very agree). Higher scores indicate higher life satisfaction. Cronbach alpha ： 0.72.	Online Social Behavior Scale ： The questionnaire used the online social behaviour scale developed by Li.19 There are 22 items scored by 5 points, 1 means "very inconsistent," and 5 means "very consistent". The test-retest reliability of the scale ranges from 0.624 to 0.794; The reliability of internal consistency ranges from 0.728 to 0.900.
43	(Ross & Ross, 2023)	Distress, Social Support, and Self-Compassion: Relationships With Mental Health Among College Students	Well-being measurement:<br>CES-D (10 items, Cronbach alpha：0.82) for depression,<br>Trait Anxiety Scale (10 items, Cronbach alpha：0.85)<br>Subjective Happiness Scale (4 items, Cronbach alpha：0.86) for happiness<br>Love of Life Scale (Cronbach alpha：0.94) for life satisfaction.	Perceived Social Support: The study used an abbreviated version of the Social Provisions Scale (Cutrona & Russell, 1987). This scale assesses perceived social support across five dimensions, with higher scores indicating more perceived support.<br>Cronbach alpha ： 0.93 .
44	(Saeed et al., 2023)	Relationship Between Social Support, Social Media Usage, and Psychological Well-being among Undergraduates in Different Institutions of Punjab	Ryff's Psychological Well-Being Scale: 42 items, 6 subscales (7 items each). 6-point Likert scale (1=strongly disagree to 6=strongly agree). Higher scores indicate better psychological well-being. Considered the gold standard for measuring PWB.<br>Cronbach alpha ： 0.798.	Inventory of Social Supportive Behavior (ISSB): 40-item scale assessing students' social support. Modified for online sources and close ties. In this approach, behaviours that can be performed in an online context and live situations (informational and emotional support), were retained, and the rest of the items (instrumental) were removed from the scale as they cannot be performed in an online context. Focuses on

				informational and emotional support. 5-point Likert scale (1=not at all to 5=about every day). Instrumental support items were removed.<br>Online social support: Cronbach alpha： 0.877.<br>General social support: Cronbach alpha： 0.815.
45	(Xin, 2023)	The association between social support provision, psychological capital, subjective well-being, and sense of indebtedness among undergraduates with low socioeconomic status	Subjective Well-being Questionnaire: Measures life satisfaction (long-term well-being indicator), positive affect, and negative affect (short-term emotional states). Combines cognitive and emotional aspects of well-being.<br>The Cronbach's α of life satisfaction in the present study was 0.81.<br>Six items were used to assess the positive effect (Cronbach alpha：0.84), and six were used to evaluate the adverse impact (Cronbach alpha ： 0.84).	Social Support Provision Questionnaire: This scale, adapted for the Chinese context, has three items rated on a 5-point Likert scale (1 = never, 5 = always) to measure the frequency of providing emotional and social support to others. Higher scores indicate more frequent provision of emotional support . The Cronbach's α of the present study's emotional social support provision scale was 0.81.
46	(Yıldırım & Green, 2023)	Social support and resilience mediate the relationship of stress with satisfaction with life and the flourishing of youth.	SWLS: 5 items, 7-point scale, measure life satisfaction. Turkish version, Cronbach alpha：0.87.<br>Flourishing Scale: 8-item, 7-point scale assesses self-perceived success in relationships, self-esteem, purpose, and optimism. Turkish version, Cronbach alpha：0.91.	Brief Perceived Social Support Questionnaire: 6 items assessing support from friends, family, and significant others. 5-point scale (1=not true at all to 5=very true). Higher scores indicate greater perceived support. Turkish version, Cronbach alpha：0.86.

47	(Han Mo et al., 2024)	Communication in social networking sites on offline and online social support and life satisfaction among university students	Life Satisfaction: Measured using Diener's Satisfaction with Life Scale (SWLS). 5 items, 7-point Likert scale. Higher scores indicate greater life satisfaction. Widely validated and used in research. Cronbach's alpha：0.78.	Offline Support: MSPSS, 12-item, 7-point scale, assesses support from family, friends, and significant others. Cronbach's alpha： 0.96. Online Support: ISCS bonding subscale, 10 items, 5-point scale, measures support from social networking sites. Cronbach's alpha： 0.84.
48	(Yang et al., 2024)	Gratitude predicts well-being via resilience and social support in emerging adults: A daily diary study.	Daily well-being was assessed using scales:<br>Satisfaction with Life (SWLS, within-person omega reliability .80, between-person 0.97),<br>Scale of Positive and Negative Experience (SPANE, positive affect within 0.72, between 0.86;<br>negative affect within 0.62, between .78), and<br>Mental Health Continuum Short Form (MHC-SF, within 0.84, between 0 .98).	Daily social support was assessed using four items from the MSPSS, including "Today, my friends try to help me" and "Today, I get emotional help and support I need from my family," rated on a 7-point Likert scale (1 = strongly disagree, 7 = strongly agree). Within-person omega reliability was 0.77, and between-person omega reliability was 0.94.

No.	Authors (Year)	Title	Well-being instrument	Social support instrument
**1**	(Siewert et al., 2011)	The more, the better. The relationship between mismatches in social support and subjective well-being	State Negative Affect: Measured by the state version of the 10-item Negative Affect scale of PANAS, rated on a five-point scale from 1 (not at all) to 5 (extremely).Perceived	Perceived Availability of Social Support was measured using the Berlin Social Support Scales (BSSS). Desired and Received Support were assessed for emotional, informational, and practical support using specific items rated on a

		in daily life	Stress: Measured with a single item on a four-point scale.	four-point scale.
2	(Wang et al., 2015)	Social Ties, Communication Channels, and Personal Well-Being: A Study of the Networked Lives of College Students in Singapore	Well-being was measured using the Satisfaction with Life Scale (SWLS), with five items on a 7-point Likert scale. The total score, summing responses, indicates life satisfaction, with higher scores reflecting greater well-being. Cronbach's α ： 0 .90.	Network structures. Questions about participants' networks closely follow the definitions in the Pew Social Tie Survey (Boase et al., 2006) and were divided into two groups. Strong ties are "the people you have felt VERY CLOSE to over the past 12 months. These might include those you discussed important matters with, kept in regular contact with, and/or people who were there for you when you needed help." Weak ties are "the people you felt SOMEWHAT CLOSE to over the past 12 months.<br>These people are more than just casual acquaintances but not as close as family and good friends."
